# Energy Levels, Classified Lines, and Zeeman Effect of Neutral Thorium

**DOI:** 10.6028/jres.080A.023

**Published:** 1976-04-01

**Authors:** Romuald Zalubas

**Affiliations:** Institute for Basic Standards, National Bureau of Standards, Washington, D.C. 20234

**Keywords:** Energy levels, thorium, Th I, wavelengths, Zeeman-effect of Th I

## Abstract

A list of about 9500 classified lines of Th I in the range 2345–29 662 Å is given. Lines in the range 2345–9239 A were observed and measured at NBS. Zeeman effect data for 2281 lines are listed. Lists of 254 even and 322 odd levels including their *g* values are presented. Among them there are 72 new levels, which were not contained in earlier publications.

## 1. Introduction

A review of early research on the spectra of neutral and singly ionized thorium has been given by Zalubas [1960].^1^ In the meantime light sources and spectrographs have been improved and more standard wavelengths of thorium have become available. Their development is described by Giacchetti, Stanley, and Zalubas [1970]. Therefore, at NBS I undertook a new observation and description of Th I and Th II in order to expand the analyses of these spectra. A complete line list of about 35 000 Th I and Th II lines will be published separately. That list will include all available thorium standards and will be useful for users of thorium standards. Zalubas and Corliss have published a list of classified lines in Th II [1974].

In the present paper I will review only the work on finding the energy levels and *g* factors of Th I.

## 2. Wavelengths and Classified Lines

New observations were taken on the NBS 10.7 meter Eagle spectrograph in the range 2000–12000 Å. Electrodeless lamps and sliding sparks were used as light sources for the separation of Th I and Th II. Professor S. P. Davis supplied some spectrograms for range 2600–4500 Å produced on the Czerny-Turner spectrograph at the University of California. I measured wavelengths in the 2800–12000 Å observation range. Stronger lines were measured on 3 to 6 plates, weaker lines (below intensity 5) on 2 to 3 plates. Small numbers of lines of intensity 1 are included which were measured only once. The wavelengths in the range 2000–2800 Å were measured only once. Internal thorium standards from Giachetti [1966], and Giacchetti, Stanley, and Zalubas [1970] were used.

All classified lines fit the energy level scheme to within ±0.05 cm^−1^. More than 60 percent of them do not exceed ±0.02 cm^−1^.

The consistency of wavelengths in this list can be demonstrated by the following test. The average absolute value of the difference between observed and calculated wavelengths expressed in cm^−1^ was 0.008 for 50 lines around each of the wavelengths 3000, 4000, 5000, 6000, and 8000 Å.

At the Laboratoire Aimé Cotton, Orsay, France wavelengths of Th I and Th II lines in the range 9239–29 662 Å were recorded by means of Fourier transform spectroscopy. The list of 3100 lines, including classified lines of Th I and Th II, was published by Giacchetti, Blaise, Corliss, and Zalubas [1974]. The average deviation between the observed and calculated wavenumbers from this list is less than 0.002 cm^−1^.

These two lists combined together have around 35 000 lines and excel earlier observations in accuracy and extent. In order to have all classified lines of Th I at hand, the 1900 Th I lines measured by Fourier transform spectroscopy are taken from Giacchetti et al. [1974] and incorporated in table 4.

Table 4 contains about 9500 classified lines in the wavelength range 2345–29 662 Å. For the wavelength range 2345–9239 Å visually estimated relative intensities on an arbitrary scale from 1 to 5000 are given for electrodeless lamp and sliding spark. For the wavelength range 9239–29 662 Å only the electrodeless lamp intensity on a scale 0 to 9 is given. The letter symbols are used to describe the character of lines; *b—*blend (two wavelengths measured), *d—*double (one wavelength measured), *h—*hazy, *l*—shaded to longer wavelength, *r*—reversed, *s—*shaded to shorter wavelength, and *w—*wide. In column 4 the wavenumber is given in vacuum in units of cm^−1^. In the fifth and sixth columns the classification of a line is given by rounded-off values of the energy levels responsible for the transition, with their *J*-values as subscripts and a superscript degree symbol to indicate odd parity.

## 3. Zeeman Effect and *g*-Factors

B. E. Moore [1909] observed the splittings of thorium lines in the magnetic field in the range 3002–4721 Å. He observed mostly Th II lines and at that time of course did not derive *J* and *g* values.

Lier [1939] observed the Zeeman effect of 40 lines of Th I in the range 3427–6182 Å. Fifteen of these lines had resolved ZE patterns. These data were used by Schuurmans [1946], who derived *g* factors for all levels he found. Charles [1958] furnished ZE data for 46 lines in the range 3041–6457 Å.

In the final list I used *g* factors derived from my observations. I have measured ca. 2281 Zeeman patterns in a magnetic field of 2.4 teslas, produced by the electromagnet at Argonne National Laboratory. From completely resolved patterns I derived *J* values and *g* values, and from partly resolved patterns I obtained Δ*g*, or *p*, or *n*, or a combination of these. Δ*g* is the difference between two *g* values for that line. The distance from the no-field position to the most distant *p* component (parallel polarization) is denoted by *p*, and *n* is the distance from no-field position to the strongest *n* component (normal polarization). All these measurements are given in Lorentz units. The type of Zeeman pattern is described by the following numbers: 1 indicates that the *n* pattern shades out, 2 indicates that the *n* pattern shades in, 3 indicates that the *n* pattern is symmetrical, *J*_1_*=J*_2_, and the *p* pattern shades in, and 7 indicates that the pattern is either triplet or unresolved. The values of *g* derived from my measurements or measured Δ*g, p*, and *n* are given in table 3.

Values of *g* factors for the energy levels are derived from various numbers of patterns, from 60 for one level to only one for some levels. Therefore, the *g*-values are given with three, two, or one decimal digits. The estimated error is not greater than 2 in the last digit.

## 4. Energy Levels

In previous papers on Th I [Zalubas, 1959, 1968] I gave an account of the earlier analyses of Th I. Here I will give just the total number of levels contributed by those early investigators that have proved to be real. Schuurmans [1946] found 29 energy levels and properly identified the five lowest levels. Stukenbroeker and McNally [1953] contributed 7 levels. Charles [1958] found 2 levels. Steers [1967], using his far infrared observations, found 13 levels. Giacchetti and Blaise [1970] found 10 levels. The remaining levels were found by the present author. Now the total number of known even levels is 254, and 322 odd levels. There are 72 levels given here for the first time.

Th I has two very distinct systems of levels, which I have connected. One system starts with the ground state 6*d*^2^7*s*^2 3^F_2_. The other starts 7795 cm^−1^ above ground state with the *5f*6*d*7*s*^2^ ${\;}^{3}H_{4}^{{}^{\circ}}$ level.

For energy level searches I treated the two systems separately. In such searches, I used a set of programs called COMBO, written by J. Tech for the analysis of spectra. The numbers of combinations and the available ZE data were the critical factors in establishing new energy levels. I have not included levels which can be found by intercombinations between high odd levels, and high even levels, despite the fact that some chains are long. Some of them may be confirmed later by theoretical calculations and extension of the analysis. Even levels are given in table 1 and odd levels in table 2.

The uncertainty of the level values for levels below 15 000 is 0.001 cm^−1^, and the uncertainties of most of the high levels do not exceed 0.005 cm^−1^. Levels given with two decimal places have uncertainties of 0.01 to 0.02 cm^−1^.

I have identified the lowest levels by using g values, and by comparison of the levels with calculations for the 6*d*^2^7*s*^2^ and 6*d*^3^7*s* configurations by Trees [I960]. For the low odd levels I made preliminary identifications using Martin’s [1963] Ce I identifications, and also Klinkenberg’s [1950] Th III identifications. Later I used Sugar’s [1968] preliminary calculations for the 5*f*6*d*7*s*^2^ and 5*f*6*d*^2^7*s* configurations. Giacchetti and Blaise [1970] identified the ^5^F, ^5^G, ^5^H, ^5^I terms of the *5f*5*d*7*s*7*p* configuration. In addition, Brewer [1971] identified 14465 as ${\;}^{5}G_{2}^{{}^{\circ}}$ of *bd*^2^7*s*7*p*, and 18 431 as ^3^F_3_ of 5/7*s*^2^7*p*. No attempt has been made to identify the terms or configurations of the high energy levels.

## 5. Ionization Energy

Ionov and Mittsev [1961] derived the ionization energy of Th I as 6.95 ± 0.06 eV, and Smith and Hertel [1969] as 7.5 ± 0.3 eV by the surface ionization method. Avni and Klein [1973] got 7.4 ± 0.3 eV for the ionization energy from measurements in a d.c. arc plasma, using the Saha equations. Meggers, Corliss, and Scribner [1961] estimated this energy to be about 50 000 cm^−1^. Rauh and Ackermann [1973] obtained an ionization potential of 6.0 ± 0.1 eV from an ionization efficiency curve and from the appearance potential by electron impact. The best value of 49 000 ± 1000 cm^−1^ (6.08 ± 0.12 eV) was obtained by Sugar [1973] utilizing interpolated spectral properties of actinide atoms.

## Figures and Tables

**Table 1:** Even energy levels of Th I

Configuration	Term	*J*	Level (cm^−1^)	*g*
6*d*^2^ 7*s*^2^	^3^F	2	0.000	0.736
6*d*^2^ 7*s*^2^	^3^P	0	2558.058	
6*d*^2^ 7*s*^2^	^3^F	3	2869.260	1.084
6*d*^2^ 7*s*^2^	^3^P	2	3687.988	1.254
6*d*^2^ 7*s*^2^	^3^P	1	3865.476	1.478
6*d*^2^ 7*s*^2^	^3^F	4	4961.660	1.212
6*d*^3^(^4^F)7*s*	^5^F	1	5563.143	0.065
6*d*^3^(^4^F)7*s*	^5^F	2	6362.397	1.011
6*d*^2^ 7*s*^2^	^1^D	2	7280.125	1.188
6*d*^3^(^4^F)7*s*	^5^F	3	7502.290	1.250
6*d*^2^ 7*s*^2^	^1^G	4	8111.005	1.067
6*d*^3^(^4^F)7*s*	^5^F	4	8800.251	1.310
6*d*^3^(^4^F)7*s*	^5^F	5	9804.808	1.366
6*d*^3^(^4^P)7*s*	^5^P	1	11601.032	2.398
6*d*^3^(^4^P)7*s*	^5^P	2	11802.934	1.713
6*d*^3^(^2^G)7*s*	^3^G	3	12847.971	1.388
6*d*^3^(^4^P)7*s*	^5^P	3	13088.564	1.050
6*d*^3^(^2^G)7*s*	^3^G	4	13297.436	0.992
6*d*^3^(^4^F)7*s*	^3^F	2	13847.774	0.932
6*d*^3^(^2^D2)7*s*	^3^D	1	13962.523	0.753
6*d*^3^(^2^G)7*s*	^3^G	5	14204.268	1.144
6*d*^3^(^2^P)7*s*	^3^P	0	14226.823	
6*d*^3^(^2^H)7*s*	^3^H	4	15493.222	0.914
6*d*^3^(^2^D2)7*s*	^3^D	2	15863.892	1.05
6*d*^3^(^4^F)7*s*	^3^F	3	15970.099	1.200
6*d*^2^ 7*s*^2^	^1^S	0	16351.942	
6*d*^3^(^2^H)7*s*	^3^H	6	16554.245	1.17
6*d*^3^(^4^P)7*s*	^3^P	1	17073.814	1.28
6*d*^3^(^2^H)7*s*	^3^H	5	17166.108	1.11
6*d*^3^(^2^D2)7*s*	^3^D	3	17398.399	1.19
6*d*^3^(^4^F)7*s*	^3^F	4	17959.898	1.17
5*f*6*s*^2^ 7*p*	^3^G	3	18431.685	
6*d*^3^(^2^P)7*s*	^3^P	2	18549.409	1.2
6*d*^3^(^2^P)7*s*	^3^P	1	18574.613	1.37
		2	18699.627	
6*d*^3^(^4^P)7*s*	^3^P	2	19273.284	1.180
6*d*^3^(^2^F)7*s*	^3^F	4	19532.421	1.20
6*d*^3^(^2^F)7*s*	^3^F	3	19713.035	1.1
6*d*^3^(^2^F)7*s*	^3^F	2	20054.776	0.88
6*d*^3^(^2^H)7*s*	^1^H	5	21143.425	1.03
		2	21575.040	1.19
		3	21594.676	1.035
5*f*6*d*7*s*7*p*	^5^I	4	22098.187	0.742
		1	22401.215	1.180
		3	22637.461	1.128
		4	23032.983	1.084
5*f*6*d*7*s*7*p*	^5^G	2	22988.523	0.625
5*f*6*d*7*s*7*p*	^5^G	3	23201.985	0.946
5*f*6*d*7*s*7*p*	^5^I	5	23277.380	1.006
5*f*6*d*7*s*7*p*	^5^F	1	23769.872	0.468
5*f*6*d*7*s*7*p*	^5^F	2	23990.407	0.875
5*f*6*d*7*s*7*p*	^5^G	4	24032.222	1.091
5*f*6*d*7*s*7*p*	^5^H	3	24399.112	0.832
		1	24627.874	0.504
		3	24664.727	0.895
		2	24671.898	1.118
5*f*6*d*7*s*7*p*	^5^F	3	24915.019	1.183
5*f*6*d*7*s*7*p*	^5^H	4	25405.160	0.918
		2	25890.390	1.113
		4	25923.018	0.96
		1	26111.536	0.374
		4	26255.580	1.032
		5	26380.537	1.049
		3	26796.070	0.814
		4	26971.459	1.036
5*f*6*d*7*s*7*p*	^5^I	6	26997.242	1.11
		3	27044.218	1.17
5*f*6*d*7*s*7*p*	^5^G	5	27191.358	1.116
		3	27343.737	0.690
		4	27495.586	0.998
		4	27521.523	0.925
		2	27566.853	0.811
		5	27591.803	1.10
		3	27612.267	0.928
		3	27980.683	1.030
		5	28034.004	1.074
		4	28227.932	1.128
		2	28273.215	0.848
		5	28342.498	1.101
		3	28358.393	1.190
		2	28531.046	0.910
		4	28562.659	1.033
		2	28679.257	0.97
		4	28845.562	0.98
		2	28882.455	0.860
		3	28934.903	1.031
		4	29104.006	0.996
		5	29141.525	1.07
		2	29418.388	1.042
		1	29422.086	1.104
		6	29552.52	1.18
		2	29650.955	0.84
		5	29684.194	1.175
		4	29756.400	1.090
		3	29835.046	1.065
		4	29849.160	1.03
		1	29961.798	1.339
		3	30011.343	1.070
		4	30014.330	
		2	30132.640	0.75
		1	30286.898	0.624
		6	30372.711	1.15
		5	30508.001	1.11
		2	30544.560	0.970
		4	30552.501	1.19
		3	30651.163	1.057
5*f*6*d*7*s*7*p*	^5^I	7	30726.784	
		2	30758.008	0.984
		1	30889.931	0.734
		6	30930.435	1.2
		3	30964.652	1.15
		1	31095.84	1.327
		3	31141.795	1.096
		2	31245.890	1.336
		3	31265.632	1.075
		5	31271.000	1.084
		4	31326.971	1.15
		1	31429.253	1.294
		3	31537.237	0.900
		5	31626.821	1.111
		3	31774.590	1.05
		4	31793.697	1.090
		3	31929.499	1.14
		4	31995.652	0.92
		4	32012.954	1.2
		2	32041.231	1.08
		5	32201.997	1.052
		5	32293.919	1.186
		4	32458.177	1.132
		3	32551.611	1.25
		5	32737.014	1.136
		4	32781.597	1.083
		3	32796.121	1.156
		5	32963.392	1.20
		6	33068.085	1.07
		3	33099.412	1.337
		5	33214.534	1.028
		1	33273.846	1.380
		2	33309.040	1.002
		1	33390.746	0.704
		3	33408.661	1.113
		4	33418.121	1.121
		4	33459.180	0.95
		4	33527.562	1.15
		3	33564.851	1.1
		2	33606.291	1.417
		2	33689.043	1.0
		2	33721.308	1.159
		1	33773.617	1.410
		4	33831.881	1.15
		3	34167.473	1.041
		1	34298.062	0.931
		4	34301.432	0.905
		6	34407.566	1.19
		3	34431.335	1.047
		2	34444.594	1.102
		4	34447.172	1.11
		5	34460.873	1.175
		3	34707.730	1.165
		5	34849.054	1.111
		4	34873.350	1.11
		3	34874.059	1.095
		1	35046.030	0.4
		3	35089.394	1.184
		2	35149.75	1.09
		5	35216.987	1.177
		5	35300.91	0.87
		3	35405.303	1.067
		1	35421.028	1.164
		3	35440.891	1.07
		3	35576.514	1.192
		5	35582.288	1.15
		4	35633.866	1.120
		4	35794.375	1.144
		6	35799.978	1.075
		3	35831.292	1.190
		2	35877.81	1.412
		2	36047.30	0.83
		4	36089.691	1.09
		5	36133.494	1.041
		5	36210.789	1.0
		4	36297.296	1.128
		3	36336.196	1.10
		2	36485.388	1.275
		3	36515.98	0.834
		2	36653.567	1.203
		6	36748.997	1.13
		3	36775.361	0.97
		5	36781.317	1.118
		3	36808.30	1.094
		2	36818.110	0.95
		4	36885.289	1.048
		4	37085.86	1.20
		2	37094.849	1.155
		3	37131.059	0.88
		5	37211.68	1.03
		4	37303.438	1.02
		3	37307.14	0.995
		6	37332.502	1.00
		3	37404.631	1.3
		3	37521.509	1.127
		3	37688.21	1.238
		6	37742.28	1.1
		2	37748.043	0.99
		2	37784.402	1.02
		4	37819.119	1.04
		5	37890.46	0.97
		4	37892.823	0.86
		4	37992.254	1.01
		2	38097.10	
		5	38106.740	1.16
		3	38145.74	1.07
		4	38186.12	0.95
		4	38366.571	1.042
		3	38382.14	1.0
		3	38529.930	1.0
		2	38602.98	1.1
		4	38700.25	1.2
		3	38734.401	1.10
		4	38760.633	1.05
		2	38807.67	1.02
		5	38953.228	1.1
		4	38956.877	1.05
		3	39010.164	
		3	39174.919	1.03
		5	39252.291	1.05
		4	39283.816	1.02
		3	39321.807	1.01
		5	39351.78	1.07
		4	39520.19	1.0
		2	39680.452	0.99
		5	39694.33	1.006
		3	39837.63	0.95
		4	40052.463	0.94
		3	40200.428	1.02
		4	40426.73	1.01
		3	40493.279	1.08
		5	40582.766	
		2	40611.670	1.0
		4	40701.33	1.07
		2	40886.99	1.2
		4	41230.973	1.05
		5	41356.165	1.0
		3	41361.409	0.91
		2	41578.403	0.86
		3	41686.69	1.1
		5	41712.85	1.10
		4	41755.863	1.09
		2	42027.37	1.019
		3	42286.444	0.95
		5	42635.880	
		5	43857.368	1.05
		6	43937.23	1.16
		6	44437.81	1.12

**Table 2:** Odd eneroy levels of Th I

Configuration	Term	*J*	Level (cm^−1^)	*g*
5*f*6*d*7*s*^2^	^3^H°	4	7795.270	0.863
5*f*6*d*7*s*^2^	^3^F°	2	8243.597	0.778
5*f*6*d*7*s*^2^	^1^G°	4	10414.133	0.986
5*f*6*d*7*s*^2^	^3^G°	3	10526.540	0.869
6*d*7*s*^2^ 7*p*	^3^F°	2	10783.153	0.732
5*f*6*d*7*s*^2^	^3^H°	5	11197.027	1.034
5*f*6*d*7*s*^2^	^3^F°	3	11241.728	1.004
5*f*6*d*7*s*^2^	^3^D°	1	11877.836	0.726
5*f*6*d*7*s*^2^	^1^D°	2	12114.365	0.970
5*f*6*d*7*s*^2^	^3^G°	4	13175.113	1.084
6*d*7*s*^2^ 7*p*	^3^F°	3	13945.308	1.116
5*f*6*d*7*s*^2^	^3^D°	2	14032.084	1.125
5*f*6*d*7*s*^2^	^3^F°	4	14206.916	1.167
5*f*6*d*7*s*^2^	^3^P°	1	14243.992	1.200
5*f*6*d*7*s*^2^	^3^P°	0	14247.307	
6*d*^2^ 7*s*7*p*	^5^G°	2	14465.220	0.810
5*f*6*d*7*s*^2^	^3^H°	6	14481.873	1.170
5*f*6*d*7*s*^2^	^3^D°	3	15166.899	1.064
5*f*6*d*7*s*^2^	^3^G°	5	15490.075	1.19
5*f*6*d*^2^ 7*s*	^5^H°	3	15618.985	0.600
6*d*^2^ 7*s*7*p*	^5^F°	1	15736.968	0.388
6*d*^2^ 7*s*7*p*	^5^F°	2	16217.482	1.070
5*f*6*d*^2^ 7*s*	^5^H°	4	16346.648	0.880
5*f*6*d*^2^ 7*s*	^5^I°	4	16783.847	0.712
5*f*6*d*7*s*^2^	^3^P°	2	17224.302	1.040
6*d*^2^ 7*s*7*p*	^3^D°	1	17354.638	0.507
6*d*^2^ 7*s*7*p*	^5^F°	3	17411.223	1.134
5*f*6*d*^2^ 7*s*	^5^H°	5	17501.174	1.004
6*d*^2^ 7*s*7*p*	^3^D°	2	17847.078	1.162
5*f*6*d*^2^ 7*s*	^5^I°	5	18011.379	0.996
6*d*7*s*^2^ 7*p*	^3^F°	4	18053.617	1.2
6*d*^2^ 7*s*7*p*	^3^D°	3	18069.066	1.136
6*d*^2^ 7*s*7*p*	^3^P°	0	18382.825	
6*d*^2^ 7*s*7*p*	^3^P°	1	18614.338	1.380
6*d*^2^ 7*s*7*p*	^5^F°	4	18809.886	1.15
		3	18930.293	0.970
		2	19039.154	1.090
5*f*6*d*^2^ 7*s*	^5^H°	6	19227.334	1.08
		3	19503.144	1.086
6*d*^2^ 7*s*7*p*	^3^P°	2	19516.981	1.361
5*f*5*d*7*s*^2^	^1^H°	5	19588.362	1.16
		1	19817.182	1.572
		4	19948.3%	1.281
5*f*6*d*^2^ 7*s*	^5^I°	6	19986.166	1.2
		3	20214.931	1.182
		5	20322.267	1.06
		1	20423.495	1.433
		2	20522.715	0.815
		0	20543.845	
		4	20566.625	1.233
		0	20724.396	
		1	20737.288	1.363
5*f*6*d*^2^ 7*s*	^5^I°	7	20867.975	1.2
		2	20922.123	1.170
6*d*^2^ 7*s*7*p*	^5^F°	5	21077.152	1.252
		3	21165.099	1.306
		2	21252.603	0.668
		4	21539.593	1.154
		1	21668.958	1.585
6*d*^3^ 7*p*	^5^G°	2	21738.052	0.529
		3	21890.134	0.942
		4	21902.272	0.984
		3	22141.606	1.095
		4	22163.133	0.992
		2	22248.950	1.141
		3	22338.996	1.017
		1	22396.827	1.527
		5	22399.400	0.935
		2	22508.048	1.384
		3	22669.890	1.233
		3	22855.301	1.133
		1	22877.519	0.615
		5	22877.583	1.198
		5	23015.155	1.08
		1	23049.461	1.363
		2	23093.984	1.321
		4	23113.549	1.040
		6	23306.831	1.1
		0	23410.201	
		1	23481.375	0.843
		3	23521.050	1.078
		2	23603.522	1.379
		5	23609.172	0.99
		4	23655.141	1.2
		1	23741.076	0.769
		2	23752.688	1.112
		4	23916.899	1.25
		6	24084.943	1.211
		2	24182.432	1.252
		4	24202.565	1.376
		4	24259.777	1.044
		5	24274.716	
		2	24307.749	1.518
		2	24381.336	1.253
		3	24421.060	1.16
		3	24561.653	1.177
		5	24701.047	1.150
		3	24769.718	1.141
		1	24838.924	0.770
		6	24850.30	
		1	24880.220	0.78
		3	24981.111	1.075
		2	25306.763	1.166
		3	25321.922	1.386
		4	25355.576	0.98
		3	25442.686	1.091
		1	25526.314	1.066
		4	25575.046	1.05
		5	25690.475	1.209
		2	25703.403	1.177
		5	25753.940	1.15
		1	25809.306	1.568
		4	25877.502	1.041
		3	26036.352	0.954
		4	26048.543	1.116
		3	26096.986	1.001
		2	26113.270	0.979
		1	26287.049	0.710
		2	26363.104	1.036
		4	26384.927	1.060
		3	26508.031	1.074
		5	26645.412	1.1
		2	26651.831	1.102
		4	26790.430	1.139
		3	26878.162	0.903
		0	26882.511	
		3	26995.777	1.166
		2	27061.417	0.987
		1	27087.981	1.162
		3	27260.171	1.105
		4	27266.028	1.150
		1	27297.579	0.6
		3	27317.393	1.087
		2	27447.746	1.07
		3	27670.964	1.219
		2	27674.327	1.090
		2	27784.368	0.860
		5	27852.743	1.259
		4	27948.610	1.263
		1	28024.691	1.030
		4	28140.870	1.222
		2	28347.551	1.543
		1	28372.707	1.805
		4	28480.159	1.02
		2	28513.300	1.100
		3	28589.314	1.179
		1	28649.145	1.091
		2	28673.429	1.130
		3	28676.243	1.093
		4	28680.738	1.192
		3	28884.958	1.180
		2	28917.957	0.945
		4	28932.654	1.111
		5	29050.740	1.12
		1	29157.095	0.876
		3	29157.879	1.210
		1	29197.326	1.141
		2	29197.906	1.38
		2	29252.790	0.978
		4	29310.020	
		2	29419.232	1.680
		1	29640.260	0.952
		3	29686.370	1.260
		5	29711.622	1.2
		5	29733.325	1.18
		3	29744.522	1.148
		2	29853.148	0.910
		4	29881.457	1.165
		3	30017.100	1.129
		4	30160.006	1.11
		2	30208.582	1.095
		3	30255.452	1.082
		1	30281.011	1.471
		5	30517.108	1.18
		4	30517.390	1.455
		2	30553.291	1.042
		0	30613.62	
		1	30723.82	1.06
		3	30761.719	1.192
		5	30788.179	1.19
		2	30812.991	0.929
		1	30928.742	0.976
		3	30990.520	1.097
		4	31019.110	1.19
		2	31075.91	1.326
		5	31141.255	0.947
		4	31194.703	0.730
		3	31283.117	1.11
		3	31523.937	1.108
		2	31599.274	1.216
		4	31671.155	1.18
		1	31712.73	1.175
		5	31716.592	1.14
		3	31780.865	1.175
		2	31870.097	0.944
		4	31953.464	1.073
		5	32023.60	1.16
		1	32080.416	0.795
		2	32160.486	1.080
		3	32197.119	1.138
		3	32285.180	1.12
		4	32294.089	1.16
		4	32439.049	1.141
		2	32503.62	1.200
		2	32575.427	0.716
		1	32650.907	1.14
		1	32665.561	0.82
		3	32754.504	1.12
		4	32862.480	1.16
		2	32954.59	1.114
		3	33043.332	1.134
		1	33161.77	1.332
		4	33270.547	1.170
		3	33294.928	1.10
		2	33297.133	1.26
		2	33427.039	0.80
		4	33560.06	1.18
		3	33591.166	1.010
		1	33662.23	1.018
		2	33718.42	1.064
		2	33734.706	1.302
		4	33799.730	1.15
		3	33800.621	1.10
		5	33844.960	1.118
		3	33967.906	0.794
		4	34001.333	1.078
		1	34111.17	0.850
		5	34182.708	1.09
		2	34324.08	1.131
		4	34344.650	1.114
		2	34371.840	1.282
		3	34430.22	1.213
		4	34492.022	0.898
		3	34583.008	1.172
		1	34591.01	1.496
		3	34704.406	1.103
		3	34725.43	1.090
		2	34851.666	0.866
		1	34878.665	1.478
		5	34886.676	1.07
		1	34989.778	1.619
		5	35081.021	1.100
		4	35131.22	1.212
		3	35240.821	1.077
		5	35273.877	1.16
		1	35328.63	1.272
		4	35351.439	1.19
		3	35462.05	1.250
		2	35533.292	1.219
		1	35678.85	1.02
		4	35733.465	1.1
		3	35812.518	1.10
		2	35885.887	1.567
		4	36062.875	1.100
		3	36074.06	1.140
		5	36132.494	1.167
		4	36178.040	1.04
		2	36188.95	1.41
		5	36275.186	1.12
		1	36361.456	1.109
		4	36382.61	1.064
		3	36427.40	1.08
		2	36488.828	1.0
		1	36556.77	1.054
		5	36625.920	1.15
		2	36668.670	0.92
		3	36762.803	1.13
		4	36800.658	0.97
		1	36837.21	0.798
		5	36837.988	1.168
		2	36871.91	1.250
		5	37008.760	1.136
		4	37041.15	1.009
		2	37149.10	1.060
		1	37159.282	0.9
		3	37159.544	1.00
		2	37234.01	1.10
		5	37571.34	1.044
		4	37605.73	1.13
		1	37616.308	
		3	37656.975	1.134
		2	37736.977	
		4	37950.044	1.13
		3	37954.97	1.1
		2	37970.270	
		3	38053.028	1.08
		2	38088.99	0.9
		3	38216.95	1.13
		1	38278.878	0.866
		4	38306.376	1.05
		2	38355.312	
		5	38385.213	0.98
		3	38416.222	1.18
		4	38580.297	1.06
		5	38665.559	1.078
		2	38669.77	1.027
		3	38675.356	0.94
		3	38788.85	0.98
		2	38814.252	0.77
		1	38840.559	1.2
		3	38998.975	1.314
		5	39062.033	
		3	39264.69	1.06
		4	39311.261	1.10
		1	39411.899	
		3	39468.675	
		4	39562.09	1.12
		3	39611.52	1.26
		5	39873.585	1.08
		4	39906.930	1.10
		3	39985.716	
		2	40096.226	1.21
		3	40254.944	
		4	40270.098	1.10
		4	40425.183	1.11
		5	40724.599	
		4	40776.519	1.16
		3	40980.714	
		4	41134.911	1.04
		2	41200.38	1.35
		2	41720.570	1.13
		1	42624.127	1.29

**Table 3:** Zeeman effect of Th I

Wavelength (Å)	Intensity		Wavenumber (cm^−1^)	Type	*J*_1_	*g*_1_	*J*_2_	*g*_2_	Δ*g*	*P*	*n*
	Lamp	Spark									
9094.821	400	100	10992.25	2	1	0.498	2	1.007			
9063.948	200	10	11029.69	3	2	0.766	2	1.186			
9048.248	800b	75	11048.83	2	2	1.006	3	1.136			
9045.341	200	20	11052.38	1	2	1.710	3	1.140			
9016.581	300	20	11087.63	7							0.918
9012.517	150	8	11092.63	7							1.090
8997.862	100	10	11110.70	1	1	0.727	2	0.627			
8967.635	500	100	11148.15	3						0.111	
8949.114	100	20	11171.22	7							0.984
8910.844	75	4	11219.20	1							0.118
8892.973	150	15	11241.75	2	2	0.734	3	1.014			
8889.187	100	10	11246.54	2	1	1.387	2	1.717			
8875.223	100	8	11264.23	1	3	1.165	4	0.975			
8868.822	200	50	11272.36	3	5	1.243	5	1.358			
8854.899	10	3	11290.08	3						0.288	
8852.784	10	3	11292.78	1							0.628
8841.170	150	20	11307.62	1							0.864
8829.686	15	5	11322.32	3	3	0.895	3	1.170			
8810.240	15		11347.31	3						0.359	
8781.093	25	1	11384.98	7							0.892
8775.575	200	40	11392.14	7							1.031
8772.803	150	8	11395.74	3	3	1.001	3	1.125			
8766.743	100	15	11403.61	2	4	0.994	5	1.150			
8760.449	100	8	1 1411.81	1	3	1.392	4	1.044			
8758.247	400	75	11414.67	2	3	1.182	4	1.310			
8749.169	100	20	11426.52	3						0.288	
8748.038	400	100	11428.00	3	3	0.976	3	1.249			
8739.779	75	10	11438.80	3						0.262	
8734.023	75	8	11446.33	3						0.136	
8732.426	150	50	11448.43	3	1	1.358	1	2.398			
8730.818	15	2	11450.54	2							1.274
8724.375	40	2	11458.99	3	2	0.928	2	1.166			
8717.754	10	2	11467.70	1							0.965
8709.233	200	50	11478.91	1	2	1.254	3	1.064			
8701.117	50	3	11489.62	2					0.190		1.682
8691.333	201	1	11502.56	1							0.125
8687.841	50	4	11507.18	1							0.698
8682.216	10	1	11514.63	7							0.869
8668.118	100	20	11533.36	2	2		3		0.135		
8665.491	400	100	1 1536.86	2	2	1.088	3	1.248			
8655.873	50	8	11549.68	3						0.075	
8649.144	50	5	11558.66	2	4	0.715	5	1.115			
8645.310	150	25	11563.79	3	1	0.759	1	1.068			
8639.444	150	50	11571.64	1	3	0.870	4	0.746			
8638.360	100	5	11573.09	3						0.661	
8631.350	50	2	11582.49	7							1.567
8630.770	20h	2h	11583.27	7							1.180
8621.315	75	2	11595.97	2	2	0.813	3	1.089			
8607.724	8	2	11614.28	7							0.968
8593.103	100	10	1 1634.04	1					0.150		
8577.279	100	15	1 1655.51	2	1	0.462	2	0.968			
8575.327	75	5	1 1658.16	3						0.376	
8573.122	500	100	11661.16	2	1	0.055	2	1.036			
8560.425	100	4	11678.46	2	1	0.864	2	1.706			
8558.448	751	4	11681.15	3						0.250	
8556.324	100	20	11684.05	3	4	0.736	4	0.982			
8554.945	200	50	11685.94	7							1.209
8544.593	150	50	11700.09	2							1.455
8543.721	150	40	11701.29	3						0.390	
8539.795	150	25	11706.67	2	2	1.004	3	1.136			
8516.555	150	50	11738.61	7							1.144
8510.622	150	25	11746.80	2	2	0.626	3	1.006			
8501.438	100	5	11759.48	3						0.065	
8496.449	100b		11766.39	3						0.271	
8496.340	75b		11766.54	7							1.136
8491.159	8	1	11773.72	7							1.409
8483.298	50	2	11784.63	7							1.266
8478.505	200		11791.29	7							1.294
8478.353	500		11791.50	2	1	0.074	2	0.503			
8471.823	200	50	11800.59	3	2	1.379	2	1.721			
8465.668	100	25	11809.17	7							2.420
8464.236	150	50	11811.17	3	2	0.778	2	0.878			
8446.516	500	150	11835.95	7							0.860
8445.495	200	150	11837.38	3	4	1.064	4	1.281			
8430.586	15	1	11858.31	7							1.110
8426.379	75		11864.23	7							0.375
8424.031	100		11867.54	3	1	0.375	1	1.201			
8421.227	500	100	11871.49	3	1	0.388	1	1.483			
8418.004	200	75	11876.04	3	2	0.886	2	0.978			
8416.742	500w	150	11877.82	7							0.745
8411.913	100	5	11884.64	7							1.116
8410.532	25	25	11886.59	2							1.980
8407.010	75	3	11891.57	3						0.364	
8406.674	40	2	11892.04	3						0.269	
8399.253	100	8	11902.55	2					0.320		
8398.171	75	8	11904.08	7							0.621
8388.528	100	10	11917.77	1	3	1.096	4	0.856			
8385.726	100	10	11921.75	3	3		3			0.737	
8384.927	8	2	11922.89	7							1.101
8383.073	8	2	11925.52	7							1.239
8379.219	100	8	11931.01	1	2	1.264	3	0.598			
8369.332	200	50	11945.10	7							1.084
8367.390	150	20	11947.87	1	2	0.811	3	0.591			
8366.073	150	15	11949.76	3	2	1.121	2	1.717			
8358.728	300	200	11960.26	3						0.170	
8346.537	100	3	11977.73	7					0.130		
8345.867	100	5	11978.69	1	1	1.369	2	1.038			
8340.933	20	3	11985.77	7							1.341
8339.403	5	2	11987.97	7							1.170
8335.700	100	8	11993.30	3						0.969	
8330.455	500	300	12000.85	3	3	1.088	3	1.240			
8320.859	500	150	12014.69	1	2	1.353	3	1.241			
8316.595	8	2	12020.85	1							0.226
8311.621	100	8	12028.04	1							0.731
8304.412	75	3	12038.48	3						0.142	
8297.172	100b	5	12048.99	2	1	0.390	2	1.255			
8295.542	100	8	12051.36	2					0.170		
8292.521	100	8	12055.75	3	2	0.836	2	1.078			
8288.404	100	5	12061.73	3						1.102	
8275.628	400	150	12080.35	3						0.130	
8263.926	150	20	12097.46	2	4	0.983	5	1.359			
8261.010	100	25	12101.73	3						0.534	
8254.741	100	15	12110.92	3	3	0.870	3	1.130			
8252.398	400	50	12114.36	2	2	0.734	2	0.966			
8246.011	25	4	12123.74	7							1.009
8237.739	20	2	12135.92	1							0.739
8234.937	100	4	12140.05	3	1	0.777	1	2.399			
8227.672	75	4	12150.77	2							1.232
8227.482	100	8	12151.05	1					0.340		
8224.956	8	2	12154.78	7							1.151
8214.147	100	10	12170.77	7							1.249
8209.444	100	8	12177.75	1	3	1.200	4	0.900			
8207.475	100	10	12180.67	2							1.440
8203.199	100		12187.02	2							1.246
8202.148	100	10	12188.58	7							0.969
8198.438	10	2	12194.09	7							1.046
8195.942	8	1	12197.81	7							1.006
8194.398	150	25	12200.11	2							1.863
8186.915	500	100	12211.26	7							1.061
8169.793	400	50	12236.85	3	2	1.194	2	1.362			
8162.064	50	3	12248.44	2							1.277
8159.737	400	100	12251.93	1	1	1.402	2	1.007			
8157.540	100	8	12255.23	1							0.673
8154.176	10		12260.28	7							0.830
8143.143	500	300	12276.89	1							1.063
8137.940	100	4	12284.74	1					0.150		
8129.681	100	5	12297.22	7							1.087
8122.725	200	50	12307.75	7							1.255
8122.350	100	5	12308.32	2							1.460
8114.539	75	4	12320.17	3	4	0.715	4	1.001			
8096.253	20	3	12348.00	7							0.963
8093.631	200	75	12352.00	1	1	1.476	2	1.066			
8086.568	15	4	12362.79	7							1.301
8075.651	150	20	12379.50	3	2	1.705	2	1.253			
8070.094	25	1	12388.02	7							1.390
8066.826	75	8	12393.04	2	4	0.999	5	1.209			
8057.973	50	2	12406.66	7							1.141
8032.433	200	50	12446.11	7							1.329
8026.192	100	8	12455.78	2							1.328
8025.708	100	8	12456.53	2							1.675
8024.235	100	500	12458.82	2					0.220		
8022.310	100b	20	12461.81	3						0.211	
8022.191	150b	25	12462.00	2					0.222		
8014.499	50	3	12473.96	7							1.393
8013.499	15	2	12475.51	2					0.170		
8010.708	100	4	12479.86	2					0.148		
8006.443	15		12486.51	7							1.055
8003.446	100	5	12491.18	2							1.038
8002.179	50	5	12493.16	3						0.208	
8000.033	100	10	12496.51	2							1.370
7996.963	40	3	12501.31	7							1.287
7993.676	150	8	12506.45	2	3	0.860	4	1.084			
7991.366	50	5	12510.06	1							0.490
7978.977	500	300	12529.49	3	2	1.066	2	1.253			
7976.175	8	2	12533.89	7							0.978
7972.598	200	20	12539.51	1	4	1.215	5	1.018			
7961.994	100	8	12556.21	2							1.598
7961.158	75	4	12557.53	3						0.250	
7954.592	150	15	12567.90	7							0.935
7941.721	200	75	12588.27	1	3	1.031	4	0.886			
7937.727	100	20	12594.60	3	5	0.935	5	1.371			
7929.938	5		12606.97	3						0.236	
7929.809	25c	2	12607.18	3	1	0.487	1	1.328			
7927.546	501	3	12610.78	7							0.899
7925.734	75	3	12613.66	3						0.485	
7922.235	15		12619.23	3						0.096	
7916.790	40	5	12627.91	2					0.111		1.460
7914.493	8	2	12631.57	7							1.306
7908.148	100	5	12641.71	3	2	1.081	2	1.373			
7904.411	75	5	12647.69	3	4	0.907	4	1.231			
7903.319	8	1	12649.43	7							1.075
7900.309	200	75	12654.25	1	2	0.864	3	0.614			
7899.614	100	50	12655.36	7							1.276
7897.230	15	3	12659.18	7							1.241
7893.614	75	5	12664.98	3						0.452	
7869.771	15	2	12703.35	7							1.338
7868.924	40	10	12704.72	3						0.147	
7868.396	75	10h	12705.57	3						0.159	
7865.954	400	75	12709.52	7							1.362
7862.336	15	3	12715.37	7							1.040
7853.284	20		12730.02	7							1.384
7849.892	5	1	12735.52	7							1.146
7848.441	100	40	12737.88	2							1.455
7847.536	500b	150	12739.35	3	4	1.154	4	1.307			
7842.254	100	10	12747.93	3					0.130		
7841.781	200	50	12748.70	2	2	0.862	3	1.002			
7836.456	100	5	12757.36	3						0.475	
7823.475	50	2	12778.53	7							0.911
7822.392	100	4	12780.30	2	1	2.380	2	1.245			
7817.771	500	200	12787.85	2							1.070
7809.452	100	15	12801.47	2							1.282
7807.879	40		12804.05	3						0.327	
7804.533	100	5	12809.54	3						0.131	
7804.146	75	4	12810.18	2							1.362
7802.824	10	2	12812.35	7							1.101
7798.360	600	25	12819.68	7							0.059
7793.128	10	2	12828.29	7							1.255
7788.932	500	75	12835.20	7							0.808
7782.318	150	50	12846.11	7							1.272
7778.488	75	5	12852.43	3						0.481	
7776.671	40	2	12855.43	2	2	1.170	3	1.394			
7773.744	50	5	12860.28	7							1.100
7761.704	40	4	12880.22	2							1.357
7749.325	8	2	12900.80	7							1.099
7742.552	100	2	12912.09	1					0.320		
7734.617	8	2	12925.33	7							1.096
7728.939	100	15	12934.83	7							1.178
7721.188	100	8	12947.81	3						0.290	
7710.258	100	15	12966.17	2	4	1.072	5	1.262			
7703.679	100	10	12977.24	7							1.195
7699.765	100	5	12983.84	3						0.191	
7697.922	75	5	12986.94	3						0.472	
7693.797	100	5	12993.91	7							1.117
7691.765	75	3	12997.34	2	2	1.286	3	1.414			
7683.010	50	1	13012.15	7							1.239
7676.212	100	15	13023.67	3	2	0.892	2	1.192			
7668.955	50	1	13036.00	2	1	0.784	2	1.710			
7666.566	100	2	13040.06	7							1.279
7660.889	150	4	13049.72	1	4	1.207	5	1.017			
7654.695	100	5	13060.28	1					0.370		
7653.827	200	10	13061.76	7							1.137
7652.317	100	3	13064.34	7							1.188
7647.380	500	50	13072.78	3	5	1.198	5	1.370			
7642.873	15		13080.48	3						0.231	
7637.385	75	2	13089.88	2					0.381		2.444
7636.173	100	4	13091.96	3						0.095	
7633.767	150	2	13096.09	1					0.270		
7630.310	100	5	13102.02	3	4	0.986	4	1.306			
7627.175	150	5	13107.41	2							1.401
7620.080	100	5	13119.61	7							1.166
7616.686	50	1	13125.46	2	1	0.748	2	1.164			
7607.519	40	1	13141.27	2							1.368
7605.083	5		13145.48	2					0.485		
7602.795	40	1	13149.44	7							1.264
7598.203	200	3	13157.38	3						0.580	
7591.999	10		13168.14	7							1.109
7591.531	20		13168.95	3						0.157	
7586.988	8		13176.83	1					0.208		
7585.783	200	10	13178.93	7							0.386
7585.688	200	15	13179.09	7							1.106
7583.422	25		13183.03	1	3	1.092	4	0.920			
7580.686	40	1	13187.79	7							1.212
7577.266	20		13193.74	3						0.182	
7573.342	50	1	13200.58	1	3	1.377	4	1.111			
7571.029	8		13204.61	7							1.046
7569.509	100	2	13207.26	3	2	0.728	2	0.876			
7567.739	300	15	13210.35	3	5	1.080	5	1.372			
7557.751	50	1	13227.81	1					0.166		
7555.326	50	1	13232.05	1	2	0.986	3	0.902			
7549.316	200	8	13242.59	3	2	0.815	2	1.187			
7537.429	100	2	13263.47	1					0.270		
7531.818	50	2	13273.35	3						0.089	
7531.142	75	2	13274.54	7							1.067
7523.133	100	3	13288.67	3	4	0.998	4	1.169			
7520.694	15		13292.99	3						0.190	
7520.127	8		13293.99	2	2	1.034	3	1.174			
7517.963	100	2	13297.81	1	1	1.400	2	1.146			
7516.737	50	1	13299.98	3						0.420	
7511.788	100	4	13308.74	1	4	1.040	5	1.384			
7508.477	100	4	13314.61	3	4	0.938	4	1.176			
7505.292	75		13320.26	3						0.135	
7501.180	8		13327.57	7							1.250
7499.643	10	1	13330.30	3						0.124	
7499.000	100	2	13331.44	3	2	0.778	2	1.186			
7495.564	75	2	13337.55	2	4	0.887	5	1.176			
7493.427	100	2	13341.36	2	3	1.092	4	1.304			
7487.972	100	3	13351.07	2	2	0.780	3	1.040			
7483.624	200	10	13358.83	1	1	1.471	2	1.040			
7481.352	200	15	13362.89	3	4	0.996	4	1.306			
7474.559	10	1	13375.03	3						0.267	
7469.054	40	1	13384.89	7							0.770
7465.443	100	3	13391.36	1	2	1.439	3	1.189			
7462.990	75	1	13395.77	7							0.961
7461.290	8	1	13398.82	3	2	1.170	2	1.336			
7458.753	50	2	13403.38	2	1	0.506	2	0.984			
7457.648	25	1	13405.36	2	3	0.925	4	1.170			
7456.818	20	1	13406.85	2							1.283
7455.204	100	4	13409.76	3	4	0.878	4	1.090			
7452.516	20	1	13414.59	2	2	0.944	3	1.194			
7450.213	50w	5	13418.74	7							1.201
7447.848	150	5	13423.00	3	3	0.890	3	1.002			
7443.873	50	1	13430.17	1							0.840
7442.040	15		13433.48	3						0.463	
7430.255	500	15	13454.78	1	1	1.574	2	1.012			
7428.943	800	50	13457.16	1	1	1.363	2	1.179			
7422.013	100	2	13469.72	3						0.601	
7418.548	200		13476.02	2	1	0.066	2	1.088			
7417.789	100		13477.39	1	3	1.075	4	0.871			
7411.739	100	4	13488.39	2	3	1.050	4	0.872			
7410.968	50	1	13489.80	3						0.275	
7409.221	40		13492.98	3						0.525	
7404.719	401		13501.18	7							1.174
7403.993	100	1	13502.51	3						0.603	
7402.248	400	8	13505.69	2	3	0.864	4	1.094			
7396.895	20	1	13515.46	2							1.499
7392.637	15	1	13523.25	7							1.296
7383.712	150	8	13539.59	7							1.203
7376.879	200	8	13552.14	2	5	1.082	6	1.172			
7376.178	25		13553.42	7							1.143
7370.827	100	2	13563.26	7							0.969
7362.195	50	1	13579.16	3						0.525	
7355.179	10		13592.12	3						0.270	
7353.644	15		13594.96	7							1.084
7353.007	15		13596.13	7							0.962
7350.454	75		13600.85	2	3	1.041	4	1.233			
7341.150	500	15	13618.09	3	4	0.986	4	1.088			
7339.599	150	3	13620.97	1					0.280		
7335.568	100	15	13628.45	7							0.834
7329.488	100	5	13639.76	1	2	1.713	3	1.093			
7324.808	300	10	13648.47	3						0.590	
7323.207	100	3	13651.46	3						0.571	
7315.609	150	3	13665.64	2	1	0.734	2	1.058			
7315.064	200	3	13666.65	2	1	0.522	2	1.262			
7311.510	100	3	13673.30	3	3	0.994	3	1.174			
7309.255	100	2	13677.52	1							0.938
7302.172	50	2	13690.78	2							1.622
7298.138	200b	3	13698.35	1	3	1.162	4	0.990			
7296.265	100	3	13701.87	2					0.094		
7294.316	15	1	13705.53	3						0.239	
7284.905	500	20	13723.23	1	2	1.248	3	1.133			
7282.732	100	2	13727.33	2							1.436
7279.142	8		13734.10	3						0.579	
7277.013	75	1	13738.12	3						0.099	
7274.294	15	1	13743.25	3						0.224	
7272.550	100	2	13746.55	3						0.294	
7270.558	100	2	13750.31	2	2	0.673	3	1.255			
7268.834	100	2	13753.58	7							1.182
7267.096	50	1	13756.86	7							1.362
7264.118	50		13762.50	2							1.546
7262.190	10		13766.16	3						0.187	
7258.177	200	5	13773.77	2	3	1.034	4	1.164			
7256.981	150	4	13776.04	3	2	0.974	2	1.110			
7255.351	200	5	13779.13	2	3	0.942	4	1.074			
7247.988	25	2	13793.13	7							1.144
7247.599	40		13793.87	7							1.018
7244.689	200	8	13799.41	1	3	1.030	4	0.856			
7242.086	200	10	13804.37	3	5	0.990	5	1.370			
7235.890	50	1	13816.19	1					0.160		
7233.390	100		13820.97	7							1.184
7230.888	500	5	13825.75	1							0.472
7220.989	100	2	13844.70	2	1	0.500	2	0.720			
7219.156	300	10	13848.22	3						0.230	
7208.000	500	150	13869.65	2	3	1.233	4	1.323			
7206.476	400	8	13872.58	3	3	0.836	3	0.881			
7201.803	100	2	13881.59	2	2	0.740	3	0.900			
7201.042	50	1	13883.05	2					0.284		
7200.041	400	10	13884.98	2	2	1.188	3	1.304			
7179.725	150	4	13924.27	3						0.332	
7177.284	75	2	13929.01	2					0.154		
7173.372	500	20	13936.60	1							0.879
7170.357	100	2	13942.46	1					0.260		
7168.896	800	75	13945.31	2	2	0.747	3	1.116			
7167.202	150	4	13948.60	1					0.088		
7159.943	300b	8	13962.74	1	3	1.105	4	0.984			
7159.877	100b	2	13962.87	7							1.132
7156.936	300	15	13968.61	3	4	0.989	4	1.155			
7154.952	200	8	13972.48	3	2	0.665	2	1.186			
7153.587	200	10	13975.15	3						0.829	
7150.285	400	25	13981.60	1	1	1.475	2	1.167			
7148.557	400	15	13984.98	2	3	0.826	4	0.988			
7142.328	300	8	13997.18	2	1	0.370	2	0.970			
7138.142	20		14005.39	7							1.171
7132.603	200	4	14016.26	2							1.192
7131.353	150	40	14018.72	3						0.076	1.140
7130.718	200	5	14019.97	1					.080		
7130.179	100	4	14021.03	3						0.174	
7126.814	100	3	14027.65	3						0.051	
7125.515	150	2	14030.21	3	3	3			0.480		
7124.562	500	50	14032.08	3	2	0.737	2	1.125			
7112.917	100	4	14055.06	2					0.180		
7111.045	75	2	14058.76	7					0.190		
7102.589	100	2	14075.49	1							0.968
7088.822	150	2	14102.83	7							1.199
7084.165	500	75	14112.10	2	4	1.248	5	1.363			
7072.394	300	8	14135.59	1	4	1.172	5	1.104			
7067.513	75		14145.35	7					0.250		
7066.287	100	2	14147.80	3	3	1.164	3	1.385			
7064.451	300	10	14151.48	7							1.143
7062.418	40	1	14155.55	3						0.125	
7061.392	100	2	14157.61	1	1	1.180	2	0.770			
7060.039	300	8	14160.32	3	2	0.820	2	1.008			
7059.526	150	3	14161.35	2					0.230		
7058.488	150	5	14163.43	1	3	0.998	4	0.913			
7054.416	150	2	14171.61	3						0.189	
7038.717	75	1	14203.22	7							1.194
7036.277	400	8	14208.14	2							1.479
7033.352	100	2	14214.05	3						0.313	
7028.017	100	2	14224.84	3	1	0.401	1	1.340			
7023.523	100	3	14233.94	2							1.057
7023.145	100	3	14234.71	1	1	1.380	2	1.090			
7021.279	150	8	14238.49	2							1.389
7009.047	50	1	14263.34	7							1.274
7007.092	100	4	14267.32	3						0.202	
7002.881	200	8	14275.90	2							1.609
7000.806	500	75	14280.13	1	5	1.349	6	1.211			
6998.247	100	2	14285.35	3						0.346	
6995.141	75	2	14291.70	7							1.106
6989.657	1000	150	14302.91	3	4	0.744	4	0.874			
6986.031	150	4	14310.33	3	2	0.962	2	1.702			
6981.082	100	3	14320.48	3						0.336	
6969.301	100	3	14344.68	3						0.079	
6965.946	5001	4	14351.59	2	1	0.699	2	1.097			
6962.862	75	2	14357.95	7					0.397		
6954.655	200	5	14374.89	1	1	1.364	2	1.008			
6948.205	300	20	14388.24	3	4	0.925	4	1.169			
6945.488	200	8	14393.87	2					0.350		
6942.535	150	8	14399.99	1					0.266		
6936.651	150	4	14412.20	3	3	1.383	3	1.119			
6920.037	150	5	14446.80	1							0.596
6916.129	200	10	14454.97	2	4	1.043	5	1.376			
6911.222	800r	200	14465.22	3						0.147	
6904.578	100		14479.15	3						0.105	
6900.762	100	5	14487.16	2					0.370		
6886.404	200	10	14517.36	7							1.478
6883.311	200	10	14523.89	1	3	1.132	4	0.912			
6880.769	15	1	14529.25	7							1.173
6874.752	200	10	14541.97	3						0.143	
6870.988	150	3	14549.93	3	1	0.392	1	0.619			
6868.455	100	3	14555.30	2	4	0.989	5	1.259			
6866.756	100s	2	14558.90	7					0.140		
6866.368	150	3	14559.72	3						0.312	
6862.873	100	2	14567.14	3	2	1.099	2	1.410			
6855.315	150	4	14583.20	2					0.157		
6853.511	100	4	14587.04	2					0.220		
6852.344	100	2	14589.52	7							1.150
6850.704	100h	2	14593.01	7							1.197
6846.469	100	2	14602.04	7							0.718
6841.690	10		14612.24	7							1.043
6839.287	100	2	14617.37	7							0.864
6834.925	400	50	14626.70	1							0.928
6829.037	500	25	14639.31	3	3	1.095	3	1.249			
6824.678	400	20	14648.66	1	2	1.101	3	1.000			
6823.500	150	4	14651.19	3						1.010	
6815.604	100	2	14668.17	7							1.054
6809.311	150	4	14681.72	1					0.160		
6805.746	100	2	14689.41	2							1.050
6802.783	100	3	14695.81	2	2	0.864	3	1.049			
6798.483	150	3	14705.10	1					0.630		
6795.797	100	2	14710.92	2					0.360		
6795.046	75	2	14712.54	7							0.952
6791.232	400	25	14720.81	2	3	1.087	4	1.318			
6788.837	300	25	14726.00	2	4	0.970	5	1.042			
6787.734	300	25	14728.39	2							1.232
6780.126	400	75	14744.91	3	2	0.622	2	0.772			
6776.230	100	3	14753.40	1					0.200		
6772.176	400b	15	14762.23	1					0.165		
6765.683	200	100	14776.40	3						0.720	
6763.340	50	2	14781.51	7							1.126
6762.500	40	1	14783.35	7							1.224
6760.667	100	3	14787.36	7							1.212
6758.202	200	8	14792.75	3						0.837	
6757.105	100	5	14795.75	1	3	1.099	4	0.891			
6751.426	100	3	14807.60	2	1	0.378	2	0.970			
6749.315	50	1	14812.23	3						0.370	
6742.881	200	8	14826.36	2	2	1.090	3	1.386			
6738.179	200	5	14836.71	3	3	1.020	3	1.241			
6735.128	100	2	14843.43	3						0.899	
6733.748	150	5	14846.47	3						0.100	
6732.650	100	3	14848.89	3						1.191	
6728.757	150	5	14857.48	3						0.203	
6727.459	500	75	14860.35	3	1	0.069	1	1.434			
6719.198	200	5	14878.62	2					0.044		
6717.384	100	3	14882.64	7							0.982
6715.515	100	2	14886.78	3						0.199	
6713.967	300	8	14890.21	3	2	0.665	2	1.015			
6711.249	100	4	14896.24	3						1.252	
6697.708	200	5	14926.36	1	1	1.380	2	1.240			
6696.140	200	5	14929.86	2							1.595
6694.492	100	2	14933.53	2					0.630		
6687.521	150	3	14949.10	3						0.258	
6684.049	100	2	14956.86	1					0.294		
6683.368	200	3	14958.39	2	2	0.782	3	0.946			
6680.087	100	1	14965.73	2	2	0.750	3	1.080			
6678.707	200	8	14968.83	3						0.083	
6673.590	200	5	14980.30	3	4	0.887	4	1.157			
6668.826	100	2	14991.00	3	4	0.922	4	0.982			
6663.702	100	3	15002.53	3						0.152	
6662.270	800	200	15005.75	1	2	1.379	3	1.250			
6658.678	400	15	15013.85	7							1.054
6654.371	100	3	15023.57	2							1.536
6638.912	150	20	15058.55	7							1.051
6626.125	75	1	15087.61	3	1	1.018	1	1.378			
6613.376	200	8	15116.70	1	1	1.518	2	1.182			
6593.940	500	150	15161.25	7							0.069
6593.464	100	15	15162.35	2							1.362
6591.485	400	75	15166.90	2	2	0.736	3	1.064			
6588.540	500r	200	15173.68	1	1	1.482	2	1.092			
6583.906	400r	200	15184.36	2							1.482
6580.231	75	3	15192.84	1					0.530		
6579.482	20	2	15194.57	3						0.122	
6577.216	100	75	15199.80	3						0.163	
6564.443	150	8	15229.38	1	2	0.966	3	0.686			
6562.105	50		15234.80	3	1	0.761	1	1.141			
6558.871	100	8	15242.32	1	2	1.246	3	0.964			
6551.703	100	5	15258.99	1	2	1.524	3	1.038			
6538.301	50	4	15290.27	2	1	0.762	2	0.998			
6531.342	800r	200	15306.56	1	1	1.582	2	1.014			
6522.044	100	15	15328.38	2	1	0.376	2	0.723			
6512.366	400	15	15351.16	3	2	1.093	2	1.249			
6506.989	100	15	15363.85	2	2	1.135	3	0.866			
6501.992	100	5	15375.65	2	2	0.532	2	1.004			
6497.490	50	3	15386.31	3						0.187	
6488.882	100	4	15406.72	7							0.638
6462.606	200b	100	15469.36	7							1.184
6457.280	800r	300	15482.12	2	4	0.866	5	1.006			
6450.007	200	15	15499.58	1	2	1.542	3	1.386			
6446.771	100	8	15507.35	3						0.216	
6438.918	100	8	15526.27	2	1	0.464	2	0.794			
6437.769	200	20	15529.04	3						0.164	
6427.507	50	4	15553.83	3						0.328	
6422.106	100	8	15566.91	2							1.410
6417.908	15	2	15577.09	3						0.141	
6415.536	75	5	15582.86	2					0.264		
6413.615	400	100	15587.52	1	3	1.200	4	1.002			
6411.899	500	75	15591.69	1							1.095
6406.446	100	5	15604.96	3						0.098	
6400.696	100	8	15618.98	1							0.310
6396.951	100	8	15628.13	2							1.510
6394.045	100	8	15635.23	3						0.440	
6392.371	75	8	15639.32	7							0.995
6387.393	200	10	15651.51	1	1	1.466	2	1.356			
6376.930	500	200	15677.19	7							1.138
6369.141	200	25	15696.37	7							1.062
6362.256	100	20	15713.35	7							1.008
6359.677	75	5	15719.72	7							0.824
6355.911	800	200	15729.04	2	3	0.862	4	1.032			
6355.633	75	8	15729.72	7							1.025
6348.737	200	15	15746.81	3						0.171	
6342.860	500	150	15761.40	2					0.120		1.668
6339.663	100	15	15769.35	1	1	1.366	2	1.179			
6331.415	200	15	15789.89	1					0.220		
6327.279	500	100	15800.21	2							1.496
6326.365	200	20	15802.50	3						0.527	
6322.866	20	2	15811.24	7							1.051
6317.183	150	8	15825.47	2	2	1.135	3	1.395			
6315.774	100	8	15829.00	3						0.232	
6307.868	25	3	15848.83	7							0.953
6303.251	200	20	15860.44	1	3	1.218	4	0.995			
6301.421	200	20	15865.05	7							0.832
6300.917	150	10	15866.32	7							1.119
6298.903	100	5	15871.39	3	2	1.099	2	1.705			
6293.241	150	15	15885.67	3						0.693	
6292.891	100	8	15886.55	3						0.296	
6291.192	300	15	15890.84	3						0.100	
6271.544	75	5	15940.63	7							1.332
6261.418	400	150	15966.41	7							1.148
6246.173	150	8	16005.38	7							0.929
6240.949	150	8	16018.77	3						0.525	
6234.856	400	75	16034.42	2	1	1.527	2	1.014			
6224.527	400	40	16061.03	3						0.239	
6220.011	100	5	16072.69	7					0.325		
6208.686	100	5	16102.01	7					0.320		
6207.220	500	200	16105.82	3	1	0.071	1	1.589			
6205.861	100	8	16109.34	7					0.340		
6203.493	500	100	16115.49	7							1.359
6198.223	400	75	16129.19	1	1	1.582	2	1.262			
6191.906	400	50	16145.65	3	2	1.009	2	1.381			
6189.145	75	2	16152.85	7					0.090		
6180.703	200	8	16174.91	2	1	0.058	2	0.518			
6178.432	200	20	16180.86	2	3	1.072	4	1.304			
6169.822	500	200	16203.44	1	3	1.305	4	1.213			
6164.480	200	20	16217.48	2	2	0.739	2	1.077			
6162.968	40	3	16221.46	7							1.018
6161.353	150	8	16225.71	7							1.317
6157.093	100	8	16236.94	3						0.916	
6155.587	200	15	16240.91	1	2	1.189	3	1.077			
6154.068	100	15	16244.92	7							1.435
6151.993	150r		16250.40	2	2	1.112	3	1.250			
6124.485	200	15	16323.39	3	2	1.189	2	1.379			
6123.838	75	8	16325.11	1					0.200		
6121.413	150	50	16331.58	3					0.110	0.665	
6119.704	75	8	16336.14	1	1	1.210	2	0.976			
6116.172	100	8	16345.57	3						0.130	
6114.546	150	10	16349.92	7							1.399
6107.539	400	15	16368.68	7							1.167
6106.847	10	2	16370.53	3						0.257	
6104.572	300	200	16376.63	2					0.335		
6102.595	300	50	16381.93	2	3	0.814	4	0.982			
6101.726	100	10	16384.27	2	1	0.507	2	0.782			
6088.031	400	50	16421.13	2	2	0.786	3	0.894			
6085.375	100	75	16428.29	3	2	0.782	2	1.118			
6079.220	200	25	16444.93	2							1.546
6077.870	150	25	16448.58	3						0.185	
6055.592	100	10	16509.09	7					0.080		
6053.381	300	15	16515.12	2	1	0.616	2	1.009			
6035.194	100	5	16564.89	7							0.967
6032.876	75	8	16571.25	1					0.300		
6030.448	75s	5	16577.93	3						0.205	
6021.036	200s	75	16603.84	2	3	0.834	4	0.870			
6014.062	75	5	16623.09	7							1.194
6007.073	300s	75	16642.43	2	3	1.095	4	1.313			
6005.174	200	15	16647.70	1	2	1.367	3	1.087			
6000.766	100	8	16659.92	7							1.137
5996.630	100	4	16671.42	2					0.404		
5995.221	100	4	16675.33	3						0.996	
5994.126	500r	100	16678.38	7							1.483
5991.004	400s	50	16687.07	1	1	1.354	2	1.006			
5989.742	50	5	16690.59	3						0.391	
5982.095	100	5	16711.92	3						0.729	
5975.066	500	150	16731.58	3	2	1.010	2	1.323			
5973.665	500r	150	16735.50	1	1	1.433	2	1.257			
5969.742	150	10	16746.50	1	1	2.403	2	1.543			
5959.681	50	3	16774.77	3						1.043	
5956.263	100	8	16784.40	3						0.233	
5955.566	100	5	16786.37	1	2	1.709	3	1.179			
5953.587	100	4	16791.94	7					0.218		
5950.148	15	2	16801.65	7					0.234		
5949.373	50	5	16803.84	3						0.016	
5944.648	150	40	16817.19	3	3	0.696	3	0.875			
5942.359	40	3	16823.67	7							1.105
5940.567	50	3	16828.75	1					0.120		
5938.459	150	20	16834.72	3						0.869	
5937.665	100	10	16836.97	3						0.178	
5929.934	100	20	16858.92	7							1.481
5926.232	150	40	16869.45	7							0.798
5916.730	100	20	16896.55	3						0.735	
5914.648	100b	10	16902.49	1							0.068
5905.571	150	20	16928.48	2	3	0.932	4	1.214			
5902.602	100	2	16936.98	3						0.989	
5899.846	150	5	16944.90	2	1	0.071	2	1.387			
5899.521	75	4	16945.83	3						0.936	
5894.699	100	25	16959.70	3						0.796	
5892.434	75	5	16966.21	3						0.225	
5891.451	200	25	16969.04	2	3	0.859	4	0.997			
5886.531	100	8	16983.23	7							0.972
5885.702	200	50	16985.62	2	4	1.146	5	1.379			
5874.993	100	8	17016.58	3						0.084	
5874.040	50	3	17019.34	3						0.134	
5869.854	100	5	17031.48	2	2	0.833	3	1.011			
5868.382	150	25	17035.75	7							1.162
5866.814	75	5	17040.30	7							0.932
5863.720	100	8	17049.29	2	1	1.364	2	1.252			
5854.120	150	8	17077.26	3	4	1.041	4	1.313			
5853.474	100	5	17079.14	2					0.192		
5852.682	200	25	17081.45	3						0.050	
5843.805	100	5	17107.40	3						0.232	
5842.050	50	4	17112.54	3						0.236	
5840.639	100	5	17116.67	3	3					0.524	
5832.371	100	15	17140.94	7							1.144
5830.827	75	5	17145.47	3						0.264	
5829.876	50	5	17148.27	3						0.480	
5829.110	50	3	17150.52	3	2	1.117	2	1.339			
5822.796	100	8	17169.12	3						0.772	
5819.607	50	3	17178.53	7							1.132
5818.453	75	5	17181.93	7							1.296
5812.976	100	8	17198.13	2							1.154
5807.685	100	4	17213.79	3						0.433	
5806.201	40	2	17218.19	7					0.213		
5805.702	100	3	17219.67	7					0.186		
5804.141	500r	50	17224.30	3	2	0.734	2	1.045			
5800.830	300	20	17234.14	3	2	1.172	2	1.254			
5797.319	75	3	17244.57	3						0.282	
5796.070	200	15	17248.29	3	4	1.120	4	1.307			
5792.431	150	8	17259.12	7							1.573
5791.704	50	2	17261.29	7					0.200		
5789.644	200	15	17267.43	3	3	1.139	3	1.246			
5782.307	40b	4	17289.34	2	2	0.927	3	1.019			
5779.833	50	2	17296.74	7					0.298		
5777.398	100	8	17304.03	3						0.118	
5776.164	25	2	17307.73	7					0.128		
5773.946	150	10	17314.38	3	1	0.070	1	0.615			
5763.529	200	10	17345.67	3	3	1.085	3	1.180			
5762.796	100	10	17347.88	3						0.462	
5760.550	600r	150	17354.64	2	1	0.504	2	0.734			
5753.026	200	25	17377.34	2	3	1.016	4	1.208			
5736.031	150	8	17428.82	2	3	1.062	4	1.464			
5729.992	200d	10	17447.19	3						0.378	
5727.711	40	3	17454.14	3						0.511	
5727.021	50	3	17456.24	7							1.181
5725.390	400r	75	17461.21	2	4	1.150	5	1.368			
5724.463	75	4	17464.04	3						0.051	
5721.425	50	4	17473.32	3						0.053	
5719.625	200	50	17478.81	3	3	1.082	3	1.251			
5711.998	75	5	17502.15	7					0.080		
5708.679	50	3	17512.33	7							1.142
5705.637	50	4	17521.67	3						0.286	
5698.550	40	4	17543.46	3						0.346	
5698.292	100	5	17544.25	3	1	0.724	1	1.104			
5696.391	100	8	17550.10	1	3	1.168	4	0.921			
5685.192	100	8	17584.68	3	4	1.060	4	1.308			
5679.004	75	5	17603.84	7							0.903
5677.051	200b	8	17609.89	3						0.203	
5673.835	50	5	17619.87	2							1.436
5667.129	150	15	17640.72	2	2	0.871	3	1.016			
5665.181	200	50	17646.79	3	2	0.775	2	1.118			
5664.621	100	15	17648.53	7					0.057		
5660.662	25	3	17660.88	7					0.590		
5657.926	200	25	17669.41	2							1.632
5648.990	150	10	17697.37	2	3	1.083	4	1.233			
5646.455	100	10	17705.31	2	2	1.041	3	1.385			
5645.529	50	8	17708.22	7							1.235
5641.735	75	8	17720.13	2					0.125		
5641.562	50	5	17720.67	2					0.102		
5635.889	100	15	17738.50	7							1.233
5633.297	100	8	17746.67	7							0.907
5630.297	100	10	17756.12	3						0.754	
5629.312	50	3	17759.23	7							0.855
5624.911	50	3	17773.12	2					0.110		
5620.013	15b	5	17788.62	7					0.080		
5619.976	50b	8	17788.73	3						0.962	
5615.320	400r	15	17803.48	3	1	1.493	1	1.559			
5612.619	75	3	17812.05	7							1.126
5612.068	150	20	17813.80	3	4	0.986	4	1.128			
5610.685	200	20	17818.19	1	1	2.396	2	1.680			
5609.585	200	10	17821.68	2					0.660		
5606.390	150	10	17831.84	3	3	0.878	3	1.192			
5605.297	100b	8	17835.32	3						0.512	
5602.866	50	5	17843.05	2	2	0.789	3	0.844			
5601.604	200	8	17847.07	3	2	0.734	2	1.158			
5595.064	300	25	17867.94	2	1	0.374	2	0.775			
5587.027	500r	50	17893.64	2							1.425
5582.367	75	4	17908.58	2					0.546		
5580.756	100	10	17913.75	3						0.587	
5580.395	50	10	17914.90	3						0.054	
5579.359	300	40	17918.23	3	1	0.065	1	0.842			
5576.205	100	10	17928.36	2	4	0.992	5	1.098			
5573.355	400	20	17937.54	3						0.198	
5572.466	200	8	17940.40	3	3	1.094	3	1.248			
5570.928	50	2	17945.35	3	2	1.006	2	1.516			
5568.000	300b	150	17954.79	3						0.081	
5559.894	150	8	17980.96	1	1	1.570	2	1.252			
5558.342	400b	50	17985.98	2							1.227
5557.045	300	15	17990.18	3	4	1.132	4	1.306			
5555.534	75	4	17995.07	7					0.128		
5554.502	20	4	17998.42	7							1.276
5552.624	75	4	18004.50	1							0.791
5551.193	40	2	18009.14	3						0.092	
5548.176	400r	40b	18018.94	3	2	1.012	2	1.255			
5546.122	75	25	18025.61	3						0.131	
5542.890	300	10	18036.12	2	3	0.875	4	1.040			
5528.226	50b	5	18083.96	3	1	0.721	1	1.343			
5527.295	200	8	18087.01	7							1.217
5519.232	100	2	18113.43	7							1.270
5518.988	200b	50	18114.23	2	4	0.883	5	1.175			
5514.930	75b	3	18127.56	7							1.274
5514.873	200b	10	18127.74	3						0.354	
5509.993	400	20	18143.80	2	4	1.263	5	1.373			
5508.557	100	5	18148.53	3						0.177	
5507.542	1001	2	18151.88	3						0.752	
5501.281	100	10	18172.53	2	1	0.627	2	0.977			
5496.138	200	10	18189.54	2	1	0.068	2	1.106			
5494.330	75	10	18195.53	2	3	1.170	4	1.314			
5492.642	100	10	18201.12	2	2	1.177	3	1.263			
5492.416	50	10	18201.87	7					0.270		
5489.086	100	5	18212.91	7					0.544		
5482.498	100	8	18234.79	7							1.157
5479.072	75	75	18246.20	1					0.100		0.983
5478.742	50	3	18247.29	7					0.222		
5477.550	40	2	18251.26	3						0.084	
5474.733	50	3	18260.66	3						0.467	
5470.759	150	10	18273.94	7							1.064
5470.145	50	8	18275.97	3						0.192	
5464.205	200w	8	18295.84	3	3	1.082	3	1.310			
5463.771	50	4	18297.29	7					0.109		
5457.289	75	3	18319.02	7					0.110		
5455.149	100	8	18326.21	3						0.620	
5452.219	300	40	18336.06	2	4	1.222	5	1.364			
5441.213	100	8	18373.15	7							1.136
5440.600	75	2	18375.22	7					0.212		
5439.850	75	8	18377.75	3						0.522	
5431.112	200	40	18407.32	2	2	1.014	3	1.140			
5429.105	100	5	18414.12	3						0.305	
5424.008	300	40	18431.43	7							0.981
5419.136	100	8	18448.00	3						0.371	
5417.486	400r	40	18453.62	1	2	1.257	3	1.101			
5415.528	200b	2	18460.29	3	4	0.860	4	1.030			
5410.769	400	15	18476.52	2	1	0.770	2	1.012			
5407.652	400b	40b	18487.17	3	5	1.035	5	1.174			
5403.200	200	15	18502.41	2	4	0.884	5	1.109			
5400.145	150	5	18512.87	2					0.035		
5399.175	200	10	18516.20	3						1.208	
5398.920	400	40	18517.08	2					0.184		1.622
5398.704	150	4	18517.82	2					0.215		
5398.205	150	4	18519.53	7					0.110		
5393.972	150	8	18534.06	3						0.881	
5388.057	300	20	18554.41	1							0.907
5387.812	100	4	18555.25	7							1.195
5387.152	25	2	18557.52	7					0.213		
5386.611	500	20	18559.39	2	3	1.082	4	1.216			
5384.031	75	15	18568.28	1							0.452
5379.112	300	20	18585.26	2	4	0.877	5	1.049			
5378.837	400	40	18586.21	3						0.505	
5376.778	200	50	18593.33	3						0.134	
5374.823	150	15	18600.09	7							1.479
5372.703	200	25	18607.43	2							1.085
5369.284	200	8	18619.28	2	1	0.072	2	1.245			
5361.156	100	3	18647.51	7					0.238		
5360.150	300	5	18651.01	1	2	1.249	3	1.016			
5359.827	100	4	18652.13	7							1.032
5355.638	100	3	18666.72	2					0.260		
5351.839	150	8	18679.97	3						0.928	
5351.126	200	25	18682.46	2	3	0.590	4	0.905			
5349.003	100	3	18689.87	3						0.049	
5347.973	150	5	18693.48	3						0.160	
5343.581	500r	40	18708.84	1	1	1.526	2	1.252			
5340.501	200	10	18719.63	7							1.181
5337.018	200b	10	18731.85	3						0.580	
5330.082	200b	5	18756.22	1	2	1.186	3	0.954			
5326.976	500r	40	18767.15	2	3	0.903	4	1.068			
5326.278	200b	8	18769.61	3						0.171	
5320.767	75	20	18789.06	3						0.161	
5320.099	50	2	18791.42	7							1.111
5318.110	100	3	18798.44	7					0.430		
5317.494	200	4	18800.62	2					0.400		
5315.230	150	3	18808.63	7					0.576		
5312.904	300	15	18816.86	1	2	1.193	3	1.006			
5312.643	150b	15	18817.79	7					0.086		
5312.532	400	40	18818.18	2	1	0.077	2	1.257			
5312.001	400r	25	18820.06	3	2	1.259	2	1.386			
5311.870	75b	2	18820.53	3						0.670	
5309.616	300	5	18828.52	7					0.687		
5306.987	100	4	18837.84	3						0.453	
5303.483	100	3	18850.29	7					0.180		
5303.334	50	2	18850.82	3						0.140	
5300.524	300	5	18860.81	2					0.215	0.429	
5298.284	200	4	18868.79	2	2	0.537	3	1.089			
5297.746	300b	15	18870.70								1.587
5296.279	300	5	18875.93	2	4	1.192	5	1.372			
5295.674	50b	4b	18878.08	7							1.172
5295.089	75	1	18880.17	7					0.270		
5294.397	200	3	18882.64	1	3	1.253	4	1.073			
5291.818	200	5	18891.84	1	2	1.026	3	0.860			
5281.070	300	2	18930.29	2	2	0.728	3	0.969			
5280.346	75	2	18932.89	3						0.634	
5277.147	100	1	18944.36	3						0.324	
5274.391	100	3	18954.26	7							0.830
5274.121	300	5	18955.23	3						0.130	
5273.134	100b	10	18958.78	7					0.520		
5269.794	100	4	18970.80	3						0.067	
5262.621	100b	4	18996.65	3						0.620	
5261.474	75b	3	19000.79	7						0.106	
5258.361	400r	25	19012.41	3	1	0.615	1	1.481			
5258.212	150b	3	19012.58	7							2.402
5254.262	3001	8	19026.87	7					0.044		0.922
5238.812	75h	10	19082.98	3	2	1.047	2	1.193			
5234.107	150	1	19100.14	7					0.074		
5228.996	100	1	19118.81	2							1.460
5228.227	200	3	19121.62	3						0.198	
5220.928	100	1	19148.35	3						0.214	
5213.350	400	5	19176.18	3	4	0.863	4	1.036			
5209.724	500	3	19189.53	2	1	0.614	2	1.250			
5207.803	200	2	19196.61	7							1.078
5206.800	75	3	19200.31	3						0.158	
5205.154	200	2	19206.38	7							1.029
5203.850	500	25	19211.19	7					0.190		
5202.007	75	3	19218.00	2	1	0.724	2	1.327			
5199.323	300b	3	19227.92	1					0.100		
5198.802	500	100d	19229.85	2	3	0.869	4	1.101			
5197.236	200	5	19235.64	7					0.270		
5195.815	500	8	19240.90	3	4	1.212	4	1.380			
5194.456	400	12	19245.93	3						1.223	
5184.452	100	3	19283.07	3						0.183	
5177.621	200	3	19308.51	3						0.578	
5176.961	500	50	19310.94	3						0.354	
5175.323	200	2	19317.08	3						0.701	
5174.367	200	3	19320.65	3						0.067	
5174.242	200	75	19321.12	2							1.003
5173.671	400	2	19323.25	3						0.044	
5172.479	75	3	19327.71	3						1.439	
5161.540	400	8	19368.67	2	2	0.797	3	0.931			
5160.718	500b	150	19371.75	3						0.176	
5154.243	500r	40	19396.09	2	4	0.859	5	1.116			
5149.210	300	3	19415.05	7					0.170		
5146.059	300	10	19426.93	7					0.310		
5143.916	300b	15	19435.02	3						0.185	
5140.771	200b	5	19446.92	1					0.550		
5130.235	150	2	19486.86	7					0.324		
5125.950	300	3	19503.14	2	2	0.734	3	1.085			
5115.045	1000r	40	19544.72	7							1.481
5098.933	150	2	19606.48	7							1.093
5096.484	800	5	19615.90	3	1	0.842	1	1.480			
5090.052	400	5	19640.69	3						0.125	
5082.625	100	8	19669.39	3						0.338	
5067.974	1000r	75	19726.25	3						0.208	
5066.134	300	10	19733.42	7					0.166		
5065.190	300	5	19737.10	2	2	0.767	3	1.027			
5063.514	2001	501	19743.63	2					1.090		
5062.932	300	5	19745.90	1	3	1.136	4	0.985			
5059.861	400b	75h	19757.88	3						0.374	
5057.982	300	2	19765.22	7							1.479
5051.885	500	5	19789.08	2	3	1.182	4	1.315			
5050.780	300	5	19793.40	2	1	0.844	2	1.256			
5044.715	100	8	19817.20	1	1	1.561	2	0.731			
5043.520	75	151	19821.89	3						1.432	
5041.126	200	5	19831.31	1							0.967
5039.231	300r	81	19838.77	2							1.536
5038.306	75	3	19842.41	3						0.336	
5034.297	100	2	19858.21	3						1.178	
5029.893	200	3	19875.60	3	1	0.768	1	1.480			
5029.013	75	2	19879.07	3						0.247	
5028.656	300r	15	19880.48	3	4	1.190	4	1.310			
5023.709	50	1	19900.06	3						0.233	
5023.486	50	1	19900.95	3						0.324	
5022.174	40	2	19906.14	3						0.798	
5018.059	150	5	19922.47	3						0.376	
5017.510	100	15	19924.65	2	1	0.708	2	1.012			
5011.478	75	4	19948.63	7							1.126
5010.417	50	4	19952.85	7					0.160		
5009.938	50b		19954.76	3						0.291	
5004.001	50	3	19978.44	3						0.233	
5002.097	500r	4	19986.05	3						0.116	
4992.638	20	5	20023.91	3						0.178	
4989.309	150	10	20037.26	1							0.890
4985.948	50	3	20050.77	7							0.926
4985.373	300	10	20053.09	2	1	0.770	2	1.254			
4982.488	150	25h	20064.70	3						0.292	
4980.186	150	10	20073.97	3						0.240	
4972.754	20	2	20103.97	3						0.179	
4972.057	40	1	20106.79	7							0.771
4965.731	200	10	20132.40	3						0.808	
4961.724	100	10	20148.66	3						0.095	
4961.379	10	2	20150.06	3						0.162	
4960.421	40b	10	20153.95	7							1.083
4958.093	50	2	20163.42	2					0.360		
4957.293	75	5	20166.67	3						0.890	
4946.663	100	100	20210.02	7							1.297
4945.458	200r	10	20214.93	2	2	0.741	3	1.182			
4943.064	200b	10	20224.72	1	2	1.321	3	1.084			
4939.642	300r	15	20238.73	2	4	0.865	5	1.074			
4939.270	75	2	20240.26	3						0.252	
4938.597	15	1	20243.02	3						0.129	
4937.829	300	8	20246.16	3	1	0.072	1	1.570			
4936.775	150	100	20250.49	1					0.188		
4933.003	20	1	20265.97	7					0.186		
4928.683	50	2	20283.73	7							1.150
4927.781	200	5	20287.45	3	2	0.779	2	0.910			
4927.299	50	1	20289.43	3						0.172	
4925.951	100	8	20294.98	7							1.017
4924.246	75b	2	20302.01	3						2.026	
4920.053	75b	25s	20319.31	2					0.180		
4911.380	200	10	20355.19	2					0.254		
4909.844	50	3	20361.56	2	1	0.751	2	1.131			
4907.209	100	4	20372.49	7							1.271
4907.044	50	2	20373.18	7							1.330
4901.987	50	2	20394.20	3						0.185	
4899.336	50	3	20405.23	3						0.341	
4899.240	50	3	20405.63	7							0.936
4892.758	50	2	20432.67	3						1.122	
4891.036	10	1	20439.86	7							1.089
4887.096	50	2	20456.33	7							1.210
4886.869	50	2	20457.29	3						0.354	
4886.133	25	2	20460.37	7							1.007
4881.204	75	2	20481.03	7					0.130		
4877.809	15	5	20495.28	3						0.092	
4877.265	20	1	20497.57	7							1.051
4876.495	100	5	20500.81	7							1.178
4876.244	50	2	20501.86	7							1.088
4874.365	150	10	20509.76	3					0.270	1.085	
4872.922	300r	25b	20515.84	1	1	1.467	2	1.246			
4872.030	100	4	20519.59	3						1.298	
4868.882	150	10	20532.86	3						0.114	
4865.974	501	81	20545.13	3						0.173	
4865.477	300r	150b	20547.23	2	4	0.862	5	1.088			
4861.720	75b	20h	20563.11	7					0.312		
4861.223	300r	50	20565.21	7							1.045
4852.868	200	50	20600.62	3						0.286	
4849.861	50	15	20613.39	3						0.648	
4849.139	50	25b	20616.46	2	4	0.684	5	0.848			
4848.363	400r	150	20619.76	3	2	1.253	2	1.512			
4847.330	150	15	20624.15	2					0.386		
4843.938	100b	50b	20638.59	7							1.171
4842.166	50	40	20646.14	7					0.256		
4840.928	100b	50	20651.43	3						0.812	
4833.674	20s	4s	20682.42	3						0.289	
4833.179	200b	50	20684.54	3						0.525	
4831.597	300r	150	20691.31	2	2	0.791	3	1.035			
4826.700	200b	50b	20712.30	3						0.931	
4823.997	200r	100h	20723.91	3						0.639	
4823.604	300r	75	20725.59	1							0.860
4822.855	300r	75	20728.81	7							1.187
4821.587	150	25	20734.26	1	2	1.386	3	1.082			
4820.463	150b	40h	20739.10	3						0.630	
4819.191	150	15	20744.57	1					0.123		
4817.017	75	8	20753.94	7							0.716
4814.919	15	3	20762.98	7							1.612
4813.893	200	100	20767.41	3						0.670	
4813.719	150	20	20768.15	7					0.220		
4813.004	75	15	20771.24	7					0.200		
4809.615	200	50	20785.88	2							1.352
4808.134	500r	150	20792.28	1	4	1.220	5	1.155			
4784.038	150	20	20897.00	3						0.671	
4778.294	400r	50	20922.12	3	2	0.736	2	1.169			
4775.313	1001	5b	20935.18	7					0.370		
4766.600	300-	100	20973.45	3	1	0.760	1	1.481			
4764.344	200	300	20983.38	3						0.830	
4758.145	100	75	21010.72	7							0.878
4749.968	300b	50	21046.89	1	3	1.216	4	1.064			
4749.200	400r	150	21050.30	3	4	0.858	4	0.972			
4743.693	150w	300	21074.73	7					0.250		
4741.301	150	40	21085.36	3						0.112	
4739.674	300	40	21092.60	1	1	1.802	2	1.188			
4737.915	150	15'	21100.43	7							1.212
4734.046	200	10	21117.67	3						0.100	
4729.128	400b	100b	21139.64	1	3	1.028	4	0.865			
4723.438	800r	200	21165.10	2	2	0.728	3	1.298			
4722.089	300	50	21171.15	2	2	1.135	3	1.251			
4721.276	200	15	21174.79	3	2	0.779	2	1.049			
4712.481	300b	100	21214.31	2					0.175		2.053
4706.575	100	5	21240.93	3						0.298	
4695.456	200	50	21291.23	3						0.315	
4695.039	300r	100	21293.12	1	2	1.244	3	1.073			
4691.883	100	8	21307.44	3						0.171	
4691.631	300b	50b	21308.59	2	2	1.015	3	1.219			
4691.500	100	15	21309.18	7							1.174
4689.252	2001	50b	21319.40	1							0.033
4686.195	500r	50	21333.30	2	3	1.079	4	1.373			
4683.349	200	20	21346.27	2					0.210		
4682.229	150	15	21351.37	3						0.292	
4678.233	150	20b	21369.61	7							1.120
4676.971	150	25b	21375.38	7							1.116
4676.056	500r	150	21379.56	3						0.425	
4673.661	800r	150	21390.52	1							0.975
4673.042	200	25	21393.35	3						0.102	
4669.984	500r	100	21407.36	3						0.120	
4668.172	800r	150	21415.67	2	2	0.941	3	1.241			
4666.797	500r	150	21421.97	2	2	0.867	2	1.004			
4663.202	300r	10	21438.49	1	2	1.510	3	1.080			
4658.522	100	8	21460.03	7							1.102
4658.376	100	5b	21460.70	7							1.002
4655.210	200	20	21475.30	2							1.338
4650.233	200	5	21498.28	2	1	0.064	2	0.988			
4649.975	100	8	21499.47	3						0.153	
4647.249	200	20	21512.08	1	2	1.261	3	1.091			
4643.557	150	10	21529.19	2							1.273
4641.244	400b	150b	21539.92	2							1.284
4639.896	100	25	21546.18	3						0.118	
4639.520	300	50	21547.92	3						0.420	
4638.686	150	10	21551.79	3						0.236	
4633.765	300b	75b	21574.68	7							0.981
4631.644	200	50	21584.56	7							0.880
4624.314	100	4	21618.78	3						0.162	
4621.165	150	40	21633.51	7							0.886
4620.448	200	10	21636.86	3						0.110	
4615.334	200	15	21660.84	3	1	1.063	1	1.483			
4615.025	200	40	21662.29	7							1.004
4613.605	200	15	21668.96	1	1	1.584	2	0.744			
4610.628	300b	50	21682.95	3						0.113	
4608.620	150	8	21692.39	3						0.319	
4603.143	300b	100b	21718.20	1	1	1.335	2	0.774			
4599.704	100	10	21734.44	3						0.515	
4595.420	800r	200	21754.70	1	2	1.240	3	1.084			
4593.645	300	150	21763.11	2	2	0.863	3	1.055			
4592.668	500r	150	21767.74	2	2	0.782	3	1.077			
4570.972	500r	200	21871.05	7							1.199
4567.240	300	100	21888.92	2					0.310		
4561.348	500r	200	21917.20	7							1.208
4558.346	300	40	21931.63	2					0.280		1.966
4551.473	200	75	21964.75	2					’0.148		
4548.326	100b	10	21979.95	7							0.938
4545.915	200	300	21991.61	1					0.090		0.755
4544.512	150	200	21998.40	7							0.814
4540.999	300r	75	22015.41	3						0.207	
4535.255	300r	150	22043.30	2	1	0.621	2	0.772			
4530.319	200	100	22067.31	7							1.002
4519.259	300	75	22121.32	1	1	1.560	2	1.250			
4515.118	300r	50	22141.60	2	2	0.729	3	1.093			
4513.681	400r	150	22148.65	2	4	1.073	5	1.364			
4505.216	500	100	22190.27	2	3	1.092	4	1.306			
4499.984	500	200	22216.07	1	3	1.063	4	0.858			
4498.947	800r	200	22221.19	2	1	0.052	2	0.852			
4493.334	1000r	300	22248.95	3	2	0.738	2	1.141			
4489.664	200	100	22267.13	3						0.235	
4488.312	200	100	22273.84	2							1.621
4486.898	300r	100	22280.86	7							0.767
4485.713	150	40	22286.75	7							0.962
4482.169	400r	100	22304.37	3						0.227	
4480.823	200	200	22311.07	3						0.222	
4479.637	200	75	22316.98	2	2	0.989	3	1.051			
4478.594	150	40	22322.17	7							0.793
4475.221	200	40	22338.99	2	2	0.735	3	1.020			
4470.990	200	25	22360.14	2	1	0.956	2	1.191			
4469.526	400r	150	22367.46	3						0.395	
4468.320	200	50	22373.49	3						0.608	
4463.834	200	100	22395.98	7							1.070
4461.789	200b	100	22406.24	7							1.405
4461.528	200	150	22407.56	2	2	0.777	3	1.057			
4461.241	200	100	22409.00	1	2	1.244	3	0.994			
4459.004	100	75	22420.24	3	1	0.729	1	0.931			
4458.736	150	75	22421.59	3	1	0.723	1	1.482			
4458.002	600r	200	22425.28	3	2	0.980	2	1.250			
4457.236	200	100	22429.14	7							0.942
4452.565	200	100	22452.66	3	3	1.082	3	1.388			
4445.032	200	75	22490.71	1					0.650		
4443.665	150	100	22497.63	1	1	1.463	2	1.018			
4443.086	20	200	22500.56	3						0.272	
4438.746	400	200	22522.56	2	2	1.000	3	1.180			
4432.252	400	150	22555.56	3						0.112	
4424.836	200b	50	22593.36	2	2	0.984	3	1.187			
4423.719	400	150	22599.07	2	1	0.712	2	1.252			
4422.048	600	200	22607.61	7							0.772
4420.256	200	20	22616.77	7							1.209
4416.844	500	100	22634.24	2	4	1.141	5	1.362			
4414.486	500d	150	22646.34	7							0.804
4408.883	800r	200	22675.12	3	2	1.036	2	1.256			
4402.927	500	150	22705.79	7							0.976
4401.581	500	200	22712.73	2	4	0.885	5	1.126			
4393.759	400	150	22753.16	3	3	1.084	3	1.244			
4392.974	400	100	22757.23	3	4	0.854	4	1.194			
4391.390	150	50	22765.44	3						0.395	
4388.967	50	25	22778.00	7							1.032
4387.734	150	40	22784.40	2	1	0.071	2	1.541			
4384.656	150b	50b	22800.40	7							1.106
4380.289	200	25	22823.13	3	2		2			1.396	
4376.532	100	20	22842.73	7							1.287
4374.124	600r	150	22855.30	2	2	0.738	3	1.133			
4373.041	200	50	22860.96	1							0.771
4369.288	50b	25	22880.59	3						0.134	
4365.930	600	150	22898.19	2	2	0.778	3	1.093			
4359.372	600r	100	22932.64	2							1.151
4358.321	500	150	22938.17	3	2	0.732	2	1.159			
4354.483	400	100	22958.39	7							0.953
4353.449	300b	100b	22963.84	3	2	1.102	2	1.253			
4351.466	100	50	22974.30	7							1.177
4349.072	400	40	22986.95	3						0.142	
4348.599	400	75	22989.45	1	1	0.798	2	0.934			
4346.432	500r	200	23000.91	1	1	1.468	2	1.187			
4345.851	150	50	23003.99	3						0.582	
4343.381	100	25	23017.07	7							1.430
4342.445	500r	100	23022.03	1	2	0.777	3	1.074			
4340.896	200	40	23030.24	1	4	1.057	5	0.945			
4338.108	400	100	23045.04	3						0.130	
4337.277	800r	100	23049.45	1	1	1.356	2	0.726			
4332.339	150	40	23075.73	2	1	1.480	2	1.710			
4330.844	300	25	23083.70	3	4		4			1.307	
4328.915	300r	25	23093.98	3	2	0.733	2	1.320			
4327.715	150	25	23100.39	1	2	1.414	3	1.048			
4327.036	100	25	23104.01	7							1.135
4325.274	200	50	23113.42	3						0.639	
4320.738	75	8	23137.69	7					0.374		
4315.254	500r	150	23167.09	3	3	0.954	3	1.077			
4314.319	300	50	23172.11	1	3	1.122	4	1.072			
4312.992	800r	200	23179.24	7							1.208
4311.798	300	100	23185.66	1	1	1.294	2	0.774			
4311.625	150b	25	23186.59	7							1.396
4311.062	150	15	23189.62	3						0.139	
4308.121	300	100	23205.45	2							1.348
4307.176	600r	200	23210.54	2	5	1.041	6	1.192			
4306.365	200b	200b	23214.91	1					0.285		
4304.955	150	10	23222.51	3	1	1.164	1	1.467			
4304.417	100	25	23225.42	7							1.013
4303.096	50	8	23232.55	7							0.487
4299.839	500r	100	23250.15	1							0.796
4299.633	200	50	23251.26	7							1.570
4297.307	300b	75	23263.85	3	5	1.042	5	1.180			
4293.770	10	3	23283.01	7							1.376
4291.810	400	100	23293.64	2	2	0.790	3	0.899			
4289.655	100	40	23305.34	2	3	0.884	4	1.152			
4288.669	200	75	23310.70	2	2	0.927	3	1.248			
4288.471	200b	50b	23311.78	2							1.106
4287.082	200	40	23319.33	2							1.499
4286.229	200b	75b	23323.97	2	2	1.020	3	1.251			
4283.103	200b	50b	23340.99	7							1.340
4280.568	300r	100	23354.81	2	1	0.062	2	0.943			
4278.324	500r	200	23367.07	3	5	1.044	5	1.144			
4272.875	400r	50	23396.86	2	3	1.138	4	1.309			
4269.943	300	75	23412.92	1							1.000
4262.611	200	2	23453.20	2					0.170		
4260.984	100	2	23462.15	2	2		3		0.220		
4260.332	600r	5	23465.74	2	4	1.170	5	1.364			
4259.494	200	1	23470.36	7					0.216		
4258.521	400	2	23475.73	2	4	0.862	5	1.087			
4257.496	600r	4	23481.37	1	1	0.839	2	0.728			
4256.254	500r	8	23488.23	3	3	1.102	3	1.248			
4253.539	600r	20	23503.22	3						0.129	
4250.315	800r	75	23521.04	2	2	0.724	3	1.066			
4248.391	200	3	23531.70	3	4	0.880	4	1.160			
4235.463	600r	5	23603.52	3	2	0.732	2	1.375			
4230.427	500	5	23631.62	2					0.098		
4229.148	300r	4	23638.77	7							1.072
4228.761	150b	3	23640.93	3						0.522	
4227.387	400	8	23648.61	2	1	0.982	2	1.190			
4226.298	200	3	23654.71	2					0.107		
4221.691	75	2	23680.52	2	2	0.819	3	1.076			
4220.064	200	15	23689.65	2	1	0.064	2	0.979			
4216.068	300s	5	23712.10	1					0.080		
4214.828	200	4	23719.08	3						0.088	
4210.923	500r	10	23741.08	7							0.713
4210.765	400	10	23741.96	7							0.807
4210.455	75	2	23743.72	7							0.990
4208.412	150	4	23755.24	2					0.180		
4194.936	200	5	23831.55	2	4	0.861	5	1.111			
4187.142	50	3	23875.91	2	1	1.047	2	1.727			
4185.143	50	2	23887.31	3						0.304	
4184.603	50	3	23890.40	7							0.847
4179.314	100	8	23920.63	7					0.230		
4177.165	100	4	23932.94	3						0.256	
4170.533	500b	25	23970.99	3	4	1.111	4	1.205			
4165.766	800r	15	23998.42	3	4	0.869	4	1.090			
4162.509	400	15	24017.20	1	3	1.068	4	0.998			
4158.535	800	20	24040.15	3	5	1.119	5	1.362			
4157.280	150	5	24047.41	7							1.064
4154.719	200	5	24062.23	3						0.656	
4138.040	200	3	24159.22	3	1	1.030	1	1.478			
4136.432	200b	5b	24168.61	2	1	0.707	2	0.795			
4136.286	300	8	24169.46	2					0.104		
4134.062	200b	10b	24182.46	3	2	0.737	2	1.250			
4134.039	100b	8b	24182.60	7							2.397
4133.958	200	10	24183.07	3						0.420	
4131.207	50	2	24199.17	3						0.145	
4127.411	600b	40b	24221.43	7							0.876
4118.490	200	4	24273.90	2							1.156
4115.759	500r	50	24290.00	2	1	0.059	2	0.900			
4112.753	600b	15	24307.76	3	2	0.738	2	1.525			
4109.323	400r	5	24328.04	3						0.302	
4107.861	100	3	24336.70	2	1	1.024	2	1.250			
4107.050	40	2	24341.51	7							1.482
4102.617	400	3	24367.81	2	2	0.948	3	1.250			
4100.341	1000r	20	24381.33	3	2	0.734	2	1.252			
4099.553	100	4	24386.02	3	2	1.433	2	1.713			
4097.321	200	8	24399.30	2	2	1.014	3	1.192			
4096.077	150	4	24406.71	2	4	0.872	5	1.052			
4093.672	40	2	24421.06	2	2	0.741	3	1.172			
4091.737	40	2	24432.60	7							1.191
4087.285	200	5	24459.21	3						0.479	
4085.434	300r	10	24470.30	3						0.542	
4085.257	200	8	24471.36	2							1.472
4082.082	75	4	24490.39	2					0.085		1.413
4081.592	50	2	24493.33	3						0.197	
4081.368	800	8	24494.67	2					0.210		
4080.707	400	20	24498.64	2	4	0.866	5	1.186			
4080.359	100	4	24500.73	7							1.057
4078.875	75	4	24509.64	7							1.153
4073.856	200	3	24539.84	2	4	1.114	5	1.376			
4072.628	200s	5	24547.24	1							0.919
4071.750	200b	15	24552.53	2	2	0.758	3	1.145			
4070.756	100	2	24558.53	2	1	1.109	2	1.707			
4070.238	100	3	24561.65	2	2	0.735	3	1.189			
4067.451	400r	5	24578.48	7							1.098
4066.822	100	2	24582.28	7							1.110
4065.618	100	4	24589.56	3						0.550	
4064.332	400	10	24597.34	1	4	1.144	5	1.034			
4063.407	1500r	75	24602.94	2							1.279
4061.625	200	3	24613.73	7							1.210
4057.940	200	2	24636.08	1	2	1.405	3	0.985			
4057.327	50		24639.81	3	2	1.050	2	1.150			
4056.636	200	2	24644.01	3						0.183	
4054.302	200	8	24658.19	2	2	1.088	3	1.253			
4053.527	100	75	24662.91	3	4	0.862	4	1.133			
4050.888	800	25	24678.98	3						0.246	
4049.944	500	40	24684.72	1	1	1.802	2	1.252			
4048.432	800	50	24693.94	2	2	0.979	3	1.089			
4048.288	800	40	24694.83	3						0.322	
4047.058	50	1	24702.33	7							1.090
4046.824	200	15	24703.75	3						0.067	
4046.252	200	8	24707.25	2	1	0.760	2	1.036			
4045.226	200	2	24713.52	3						0.634	
4040.915	200	15	24739.88	2							1.067
4039.865	500	20	24746.31	1					0.060		
4038.960	100	2	24751.86	3						0.604	
4038.228	300	10	24756.34	1					0.390		
4037.562	500	15	24760.43	3	1	1.104	1	2.400			
4036.047	2000r	50	24769.72	*2*	2	0.744	3	1.143			
4035.352	150	5	24773.99	*1*	2			3	0.310		
4034.878	200	8	24776.90	3						0.789	
4033.907	600r	8	24782.86	2							1.381
4033.776	500	8	24783.67	3	1	1.082	1	1.477			
4032.595	600r	200	24790.92	2	3	1.013	4	1.312			
4029.827	400r	15	24807.95	1	1	1.481	2	1.128			
4029.658	400	15	24808.99	3	3	1.134	3	1.385			
4028.334	200	10	24817.15	3						0.258	
4027.546	1501	20	24822.00	3						0.163	
4027.007	500b	10	24825.32	3	2	1.104	2	1.248			
4026.159	150b		24830.55	7							1.042
4022.067	500b		24855.81	2	2	0.780	3	1.337			
4021.149	300	10	24861.49	7							1.297
4020.354	500	20	24866.41	3						0.172	
4019.862	200	10	24869.45	1	2	1.229	3	0.864			
4017.062	400	5	24886.78	1							0.485
4012.495	2000r	20	24915.11	2	2	0.856	3	1.074			
4011.739	500r	5b	24919.80	3						0.206	
4008.210	1500r	100	24941.74	2	4	0.860	5	1.136			
4005.960	300	8	24955.75	3	1	1.056	1	2.391			
4005.091	500	10	24961.16	2	1	1.096	2	1.255			
4003.574	400	25	24970.62	7							1.116
4001.894	300	2	24981.10	2	2	0.726	3	1.073			
4001.058	800r	40	24986.33	3	4	0.863	4	1.083			
4000.280	500	20	24991.18	1							0.767
4000.041	200b		24992.69	2					0.410		
3998.953	600	20	24999.48	3	4	1.157	4	1.322			
3998.657	300s	2	25001.33	2							1.273
3996.670	500	15	25013.76	3						0.129	
3991.731	800	40	25044.71	1	4	1.308	5	1.118			
3990.892	300	15	25049.97	3						0.991	
3990.809	300	25	25050.50	7							0.062
3990.492	800r	10	25052.48	1	1	1.476	2	0.947			
3987.219	100b	25	25073.05								0.862
3987.206	200b		25073.13	2	2	0.709	3	1.240			
3984.879	300		25087.77	7							0.941
3982.607	100	2	25102.08	1					0.268		
3975.831	150	5	25144.86	1							0.764
3975.468	200	20	25147.16	7							0.850
3974.829	200	4	25151.20	2					0.360		
3972.640	500	75	25165.06	2							1.772
3972.153	1500r	300	25168.14	2					0.350		
3971.136	100	4	25174.59	2							1.336
3969.665	300	15	25183.92	7							1.025
3967.797	100	3	25195.78	1	5	1.380	6	1.150			
3967.392	2000r	300	25198.35	3						0.393	
3966.334	200	4	25205.07	3						0.918	
3965.483	75	3	25210.48	3						0.219	
3964.329	200	3	25217.81	7							1.066
3964.030	300	15	25219.72	3						0.550	
3962.420	400r	15	25229.97	3	2	0.950	2	1.253			
3960.879	300	10	25239.78	7							1.110
3960.270	500	50	25243.66	1	2	1.275	3	1.005			
3959.300	1000r	100	25249.84	2	1	0.067	2	0.927			
3958.928	200	3	25252.22	3						0.349	
3957.798	100	2	25259.42	3						0.381	
3957.595	20h	5	25260.72	7							2.414
3957.059	100	20	25264.15	3						0.185	
3956.481	150	20	25267.83	7					0.284		
3956.005	400	10	25270.87	1							0.094
3955.891	400	50b	25271.60	3						0.570	
3955.170	800r	40	25276.21	3	5	1.095	5	1.361			
3954.727	150	2	25279.04	3						0.321	
3954.130	100	20	25282.86	3						0.228	
3953.338	400	20	25287.92	7							0.407
3952.761	500r	10	25291.62	3	1	0.876	1	1.486			
3950.804	300	8	25304.14	1					0.480		
3950.393	1000r	200	25306.77	3	2	0.742	2	1.166			
3949.975	50	2	25309.45	1							0.850
3948.133	500	40	25321.26	7					0.330		
3948.029	600r	15	25321.92	2	2	0.727	3	1.383			
3947.329	1000r	100	25326.42	2	4	1.213	5	1.359			
3946.481	400	10	25331.86	3	1	1.147	1	1.485			
3946.391	300	25	25332.44	7							1.281
3945.206	200	8	25340.04	1	3	1.118	4	0.882			
3944.253	500	20	25346.17	3	2	1.067	2	1.701			
3943.605	150	25	25350.33	7					0.170		
3942.072	500b	50	25360.19	7							0.838
3941.137	100	3	25366.21	2							1.953
3940.837	200	5	25368.14	2							1.309
3940.705	200	4	25368.99	3						0.794	
3939.538	75	2	25376.50	3						0.283	
3939.318	150	8	25377.92	2							1.150
3938.614	400b	150	25382.46	7					0.220		
3934.274	400b	50	25410.46	3						0.510	
3934.060	150	3	25411.84	1	2	1.198	3	1.008			
3933.970	150	5	25412.42	3	1	0.812	1	1.324			
3933.238	400	40	25417.15	1					0.202		
3932.911	1500r	200	25419.26	2	4	0.876	5	1.028			
3931.085	150	3	25431.07	3						1.241	
3930.658	100	3	25433.83	7							1.061
3930.331	150	20	25435.95	7							1.087
3928.865	300	50	25445.44	3						0.432	
3928.308	100	10	25449.05	3						0.476	
3927.806	400	10	25452.30	2	2	1.124	3	1.268			
3926.864	200	8	25458.40	1					0.350		
3926.056	20	1	25463.65	3						0.243	
3925.738	100	5	25465.70	3						0.247	
3925.219	600	50	25469.08	3						1.010	
3924.404	5001	10	25474.36	7							1.043
3923.799	500r	20	25478.29	1	2	1.543	3	1.083			
3922.747	50	2	25485.12	7							1.320
3922.394	100	8	25487.42	1	4	1.169	5	1.006			
3922.218	50	50	25488.56	2					0.160		1.504
3921.453	300	15	25493.53	1	1	1.248	2	0.966			
3920.442	300	15	25500.11	7							1.024
3920.058	300	10	25502.61	1					0.240		
3919.980	300	10	25503.11	7							1.145
3918.934	300	8	25509.92	3						0.243	
3918.498	300	300	25512.76	2	1	0.060	2	1.322			
3918.070	300	40	25515.54	3						0.307	
3916.417	800r	25	25526.31	1	1	1.066	2	0.738			
3915.848	200	15	25530.02	1	1	1.410	2	0.777			
3915.295	300	8	25533.63	3						0.106	
3914.291	50	3	25540.18	7							1.537
3913.865	100	10	25542.96	7							1.109
3913.823	75	20	25543.23	3						0.611	
3913.645	2001	5	25544.39	3						0.823	
3913.082	300	25	25548.07	1	1	2.398	2	1.067			
3911.950	300b	25	25555.46	7							1.101
3911.910	500r	40	25555.72	3	4	1.223	4	1.446			
3910.978	400b	10	25561.81	1					0.080		
3910.773	300	8	25563.15	2					0.210		
3910.251	50	2	25566.56	3						0.268	
3909.991	100	8	25568.26	3						0.623	
3909.139	300	20	25573.84	2	2	0.968	3	1.238			
3908.979	300	25	25574.88	1							0.626
3908.749	500	15	25576.39	7							1.118
3908.031	100	2	25581.09	7							1.002
3907.543	50	5	25584.28	3						0.420	
3907.160	200	10	25586.79	3						0.362	
3906.387	50	5	25591.85	7							1.055
3906.308	50	3	25592.37	3						0.686	
3905.298	100	2	25598.99	7							1.146
3903.916	200	5	25608.05	3						0.344	
3903.103	1000r	150	25613.39	1	3	1.113	4	0.856			
3902.788	200	10	25615.45	7							1.274
3902.171	500	50	25619.50	7							0.958
3901.662	500	15	25622.85	3	4	0.862	4	1.121			
3900.577	1000r	50	25629.97	2							1.595
3899.019	300	75	25640.21	3						0.948	
3898.795	300	100	25641.69	3						0.559	
3898.509	300b	100	25643.57	2							1.139
3897.778	200	5	25648.37	3						0.256	
3897.612	100	3	25649.47	7							1.169
3897.316	100	2	25651.41	7							0.907
3896.168	100	2	25658.98	7							1.029
3895.418	2000r	200	25663.91	3						0.305	
3894.022	40	2	25673.11	3						0.242	
3893.817	200	8	25674.47	3						0.163	
3893.651	300	40	25675.56	3						0.355	
3893.344	300	25	25677.59	2					0.268		
3892.708	200	8	25681.78	3						0.448	
3891.725	400	10	25688.27	7							0.993
3889.906	300	4	25700.28	3						0.188	
3889.542	200	8	25702.68	2							1.344
3888.242	20	1	25711.28	3						0.257	
3888.070	15	5	25712.41	7							1.041
3887.509	300	4	25716.12	7							1.208
3887.019	800r	100	25719.37	2							1.277
3886.917	800r	500	25720.04	3						0.238	
3886.256	300	8	25724.42	7							0.600
3886.064	75	25	25725.69	7					0.280		
3885.412	25	4	25730.00	3						0.548	
3885.224	200	5	25731.25	7					0.433		
3883.767	200	15	25740.90	2							1.696
3883.536	200	5	25742.44	3						0.933	
3882.853	25	1	25746.96	7							1.019
3882.324	200	8	25750.47	2							1.310
3881.792	100	2	25754.00	7					0.243		
3881.043	100	2	25758.97	1						0.485	0.662
3880.545	8	1	25762.27	3						0.126	
3880.323	100	3	25763.75	2					0.320		
3880.194	300	15	25764.61	7							1.116
3879.644	1000r	200	25768.26	1							0.987
3879.269	400	25	25770.75	2					0.266		
3878.662	400	150	25774.78	3	1	0.946	1	1.484			
3878.160	100	3	25778.12	7							1.121
3877.610	300b	15	25781.77	3						0.160	
3877.462	500	20	25782.75	2							1.720
3874.977	400	50	25799.29	7							1.208
3873.821	2000r	500	25806.99	7							1.085
3873.473	500r	50	25809.31	1	1	1.574	2	0.744			
3872.288	20	1	25817.21	7							1.040
3870.891	75		25826.53	7							1.062
3870.682	40	2	25827.92	7							1.230
3870.178	50	1	25831.28	7							0.707
3869.663	800r	50	25834.72	7							1.378
3868.389	200	2	25843.23	3						0.264	
3868.254	400	10	25844.13	2					0.191		
3866.907	500b	50	25853.13	1	2	1.151	3	1.004			
3866.771	200	2	25854.04	1					0.570		
3866.375	100	2	25856.69	3						0.136	
3864.837	200	25	25866.98	1							0.539
3862.654	200b	200	25881.28	7					0.150		
3862.419	500b	250b	25883.17	3						0.559	
3861.574	300b	40	25888.84	1							0.392
3861.502	300b	40	25889.32	3						0.371	
3861.051	200	2	25892.34	3						0.432	
3860.657	200	10	25894.98	3						0.240	
3859.466	40	5	25902.98	7							0.934
3858.358	200	150	25910.42	3	3	1.057	3	1.312			
3857.450	75	8	25916.51	7							1.161
3856.753	40	2	25921.20	3						0.350	
3856.622	200	15	25922.07	7							0.630
3856.354	200	40	25923.88	2	2	0.781	3	1.050			
3855.064	25	4	25932.55	3						0.640	
3854.289	100	5	25937.77	3						0.208	
3853.825	150b	15	25940.89	3						1.226	
3852.719	75	2	25948.34	7							1.254
3852.135	800r	50	25952.27	2	1	0.954	2	1.258			
3851.155	300	15	25958.87	1					0.410		
3850.690	50	2	25962.01	1	3	1.280	4	0.960			
3848.627	75	3	25975.92	3						0.300	
3847.617	500	10	25982.74	7							0.985
3846.886	1000r	100	25987.68	1	1	1.480	2	0.908			
3846.625	400	50	25989.44	3						0.116	
3845.092	400	10	25999.81	2							1.244
3844.933	400	15	26000.88	2							1.401
3844.571	50	1	26003.33	7							1.140
3843.507	200	8	26010.52	1					0.340		
3842.898	400r	75b	26014.65	3						0.074	
3840.613	200	75	26030.12	7					0.304		
3840.430	300	250	26031.37	7							1.284
3838.801	50b	4b	26042.42	3						0.115	
3837.343	200b	5	26052.31	7							0.979
3837.024	200	3	26054.47	7							0.604
3836.584	800r	100	26057.46	7							1.211
3836.542	1000r	150b	26057.75	7							1.009
3836.213	100	4	26059.98	3						0.189	
3835.414	200	10	26065.41	7							1.000
3834.890	100	5	26068.97	7							0.707
3834.488	100	75	26071.51	7							1.168
3833.087	400	75	26081.24	3						0.213	
3832.583	200	3	26084.67	2					0.430		
3832.323	75	3b	26086.43	7					0.240		
3831.640	300	50	26091.08	7							1.111
3830.774	800r	150	26096.98	2	2	0.738	3	1.004			
3829.040	200	4	26108.80	3						0.250	
3828.385	3000r	300	26113.27	3	2	0.734	2	0.978			
3827.502	100	2	26119.29	2	1	0.733	2	1.083			
3826.368	400r	75	26127.03	1	2	1.205	3	0.866			
3825.515	200	25	26132.85	3						0.127	
3824.553	100b	10b	26139.43	7							1.201
3824.534	400b	15b	26139.56	7							1.188
3823.989	75	3	26143.29	3	1	0.696	1	1.096			
3823.067	500r	100	26149.59	3	1	0.062	1	1.157			
3822.709	300	25	26152.04	7					0.620		
3820.792	600r	75	26165.16	3	2	0.915	2	1.248			
3820.327	200	2	26168.34	7							0.836
3819.904	500	100b	26171.24	7							0.927
3819.189	200	10	26176.14	7							1.060
3818.685	400r	15	26179.60	1	4	1.204	5	0.939			
3817.478	400	40	26187.87	7							0.990
3817.117	300	8	26190.35	1	2	1.579	3	1.139			
3816.166	300	10	26196.87	1							0.696
3815.566	500	50	26201.00	3	2	0.772	2	1.099			
3813.815	500r	50	26213.02	3	2	0.718	2	1.008			
3812.667	75	5	26220.92	7							1.272
3812.398	400	40	26222.76	3						0.404	
3812.044	40	2	26225.20	3							1.612
3811.761	75	2	26227.15	3						0.255	
3810.995	600r	150	26232.42	7							1.202
3810.295	15	5	26237.24	3						0.372	
3808.614	500	20	26248.82	3						0.267	
3807.273	400	50	26258.07	2	4	1.100	5	1.369			
3806.317	100	1	26264.66	3						0.459	
3805.010	75		26273.68	2	2	1.052	3	1.262			
3804.699	500	50	26275.83	1							0.821
3804.127	400	20	26279.78	3						0.378	
3803.985	600r	150	26280.76	1	4	1.316	5	1.106			
3803.839	400	25	26281.77	3						0.694	
3801.571	400r	100	26297.45	7							0.902
3799.386	300	8	26312.57	7							1.029
3798.432	200	75	26319.22	1							0.582
3797.205	200	10	26327.68	3	5	1.072	5	1.372			
3796.687	400b	20	26331.27	7							1.111
3796.052	200	3	26335.68	3						0.717	
3795.386	600	100	26340.30	7							1.087
3794.981	200	3	26343.11	1					0.387		
3794.697	1500r	100	26345.09	3						0.341	
3793.815	300	25	26351.21	7							1.078
3793.504	200		26353.55	7							1.424
3793.097	300	10	26356.20	3						0.181	
3792.730	4001	75	26358.74	2	3	0.873	4	1.048			
3792.371	1000r	100	26361.24	7							0.986
3791.517	300	10	26367.18	2							1.649
3790.794	1500r	200	26372.21	1	3	1.032	4	0.860			
3790.356	300	40	26375.26	7							0.668
3787.638	300	40	26394.18	1							0.689
3787.218	75	2	26397.11	7					0.400		
3784.793	300	15	26414.02	1					0.570		
3784.574	400	20	26415.55	2							1.456
3784.138	50	2	26418.60	3						0.736	
3782.338	15	1	26431.17	7							1.134
3781.318	300	75	26438.30	3						0.216	
3780.966	300r	20b	26440.76	7							0.960
3780.147	200	8	26446.48	3						0.708	
3779.561	200	15	26450.59	2	4	1.023	5	1.208			
3778.498	300	2	26458.03	1							0.270
3778.045	300	15	26461.20	7							0.978
3777.745	200	40	26463.30	1					0.282		
3777.415	300	40	26465.61	3	3	0.794	3	1.254			
3777.184	400	8	26467.23	3						0.928	
3776.622	300	40b	26471.17	3						0.242	
3776.502	300	8	26472.01	7							0.722
3776.271	600r	150	26473.63	1					0.141		
3775.423	300	15	26479.58	2							1.278
3774.732	300	10	26484.42	7							1.070
3773.306	200	8	26494.43	3						0.376	
3772.649	400	10	26499.04	1	3	1.250	4	1.078			
3771.370	1500r	100	26508.03	2	2	0.734	3	1.074			
3770.722	100	2	26512.59	3						0.590	
3770.056	1500r	200	26517.27	3	1	0.053	1	0.783			
3768.047	200	8	26531.41	1	1	1.295	2	0.769			
3767.471	100	2	26535.46	3						0.312	
3766.447	300	10	26542.68	7							1.048
3765.412	400r	50	26549.98	1	2	1.686	3	1.084			
3764.193	200	8	26558.57	2	1	0.727	2	1.101			
3763.668	300	10	26562.28	7					0.104		
3762.257	200	8	26572.24	7							1.101
3761.704	200	20	26576.14	7							1.421
3761.470	200	25	26577.80	2	4	1.062	5	1.362			
3760.271	50b	25b	26586.27	3					0.130		
3758.706	400	20	26597.34	2	1	0.062	2	1.078			
3758.467	400r	25	26599.03	7							0.879
3757.694	1000r	200	26604.51	7							0.871
3756.451	200	8	26613.30	1					0.530		
3756.293	400	25	26614.43	7							1.019
3755.986	400	50	26616.60	3						0.296	
3755.211	800r	250	26622.09	7							1.017
3754.030	500r	50	26630.47	2	2	0.780	3	1.091			
3753.241	300	40	26636.07	1	3	1.041	4	0.853			
3752.789	300	8	26639.28	7							1.149
3751.121	300	25	26651.12	7					0.150		
3750.493	400	25	26655.58	1	5	1.353	6	1.020			
3749.617	300	20	26661.81	7							1.432
3749.514	300	10	26662.55	2						0.446	
3749.083	200	25	26665.61	2	4	0.860	5	1.170			
3748.820	100	8	26667.48	3						1.030	
3745.658	500r	100	26689.99	2							1.489
3745.176	4001	20	26693.43	3						0.279	
3744.910	300	10	26695.33	2	3	1.001	4	1.172			
3742.923	1000r	100	26709.49	7							1.198
3742.275	200	5	26714.12	1					0.255		0.364
3742.019	75b	5	26715.95	3						0.268	
3741.884	200	8	26716.91	2							1.282
3739.683	150	5	26732.63	3						0.374	
3738.436	150	2	26741.55	3						0.283	
3737.935	100	10	26745.14	1					0.400		0.516
3737.514	600r	75	26748.15	7							1.486
3736.502	200	40b	26755.39	7							0.922
3735.363	100	3	26763.55	3						0.351	
3734.664	75	3	26768.56	7							1.024
3733.672	300r	25	26775.67	7							1.066
3732.985	400r	40	26780.60	3	3	0.872	3	0.995			
3732.535	150	3	26783.82	3						0.443	
3729.945	400	40	26802.43	2	1	0.409	2	0.786			
3729.836	400	25	26803.21	7							1.172
3727.903	1000r	150	26817.10	3						0.518	
3725.966	75	2	26831.05	2	1	0.858	2	1.188			
3724.672	100	10	26840.36	3						0.181	
3724.397	100	2	26842.35	7					0.140		
3723.921	300s	8	26845.78	2	2	0.772	3	1.186			
3722.655	75	2	26854.91	3						0.094	
3722.179	300b	25b	26858.34	3						0.420	
3721.219	500r	50	26865.27	3						0.416	
3719.837	400r	40	26875.26	3						0.150	
3717.895	50	2	26889.29	3						0.143	
3717.384	100	3	26892.99	7							0.960
3716.583	300	40	26898.78	1							0.912
3715.862	300	20	26904.00	3						0.171	
3715.561	400	50	26906.18	3						0.590	
3715.060	100	2	26909.81	3						0.606	
3714.696	100	3	26912.45	1	3	1.165	4	0.857			
3712.373	100	3	26929.28	7							1.062
3710.292	300	8	26944.39	1							0.819
3709.861	300	25	26947.52	1	1	1.480	2	0.928			
3709.404	75	3	26950.84	3						0.542	
3708.396	200	5	26958.17	3						0.696	
3706.402	200	75	26972.67	3						0.429	
3705.979	150h		26975.74	1							0.706
3705.665	100	40	26978.04	7							1.248
3704.862	500r	50	26983.88	2	2	0.917	3	1.086			
3704.147	200	50	26989.09	2					0.075		1.294
3703.775	500r	50	26991.80	3						0.482	
3703.230	400r	25	26995.77	2					0.436		
3702.479	100	2	27001.25	3						0.544	
3700.978	500		27012.20	7							1.327
3699.881	500r	50	27020.21	3	4	1.066	4	1.210			
3699.355	300	5	27024.05	3						0.440	
3699.182	500	75	27025.31	2						0.151	
3699.048	300	5	27026.29	2							1.518
3698.106	1000r	150	27033.17	3	5	1.168	5	1.362			
3695.288	500r	75	27053.79	2	4	0.872	5	1.113			
3694.177	400r	75	27061.93	7							1.034
3693.993	400r	200	27063.27	3	1	0.971	1	1.471			
3693.804	200b	25	27064.66	3						0.424	
3693.523	3001	25b	27066.72	3						0.824	
3693.247	200	10	27068.74	1	1	2.399	2	1.027			
3691.876	400r	100	27078.79	1	3	1.099	4	0.869			
3691.613	500r	200	27080.72	3						0.266	
3691.411	500r	50	27082.21	7							0.947
3690.624	600r	4	27087.98	1	1	1.164	2	0.734			
3690.115	200	300	27091.71	3						0.209	
3689.726	200	3	27094.57	7							1.106
3689.582	100	2	27095.63	1							0.916
3687.983	400r	75	27107.37	1	3	1.124	4	0.976			
3687.492	150b	5	27110.99	3						0.720	
3687.193	4001	20	27113.18	3						0.133	
3685.586	300b	101	27125.00	3	2	0.931	2	1.252			
3685.214	200	4	27127.75	3						0.467	
3684.932	300	20	27129.82	7							1.052
3683.493	500	25	27140.42	7							0.999
3682.179	300	50	27150.10	7							1.324
3680.605	400r	40	27161.71	2	2	0.772	3	1.072			
3680.448	500r	50	27162.87	2							1.539
3679.544	400r	150	27169.54	7							1.036
3679.134	400	50	27172.57	7							0.967
3678.478	400r	50b	27177.42	1	1	1.158	2	0.773			
3677.304	150	4	27186.10	2					0.256		
3675.960	400	50	27196.04	1	2	1.715	3	1.315			
3675.790	400r	50	27197.29	2					0.310		
3675.137	500r	50	27202.12	3						0.445	
3674.891	300r	50	27203.95	3	5	1.137	5	1.361			
3674.089	300	40	27209.88	7							0.952
3674.013	300r	50b	27210.44	1	1		2		0.149		
3672.522	500	25	27221.50	1					0.130		
3672.300	500r	50	27223.14	3	3	1.090	3	1.249			
3670.654	15b		27235.35	2							1.422
3670.520	200	4	27236.34	7					0.365		
3669.640	400	50	27242.87	7							1.022
3668.390	300b	8	27252.16	3						1.176	
3667.882	20	4	27255.93	3						0.301	
3667.621	500	40	27257.87	1							0.603
3666.981	800r	75	27262.63	3	4	1.100	4	1.308			
3665.476	100b	10b	27273.82	2					0.170		1.821
3665.443	150b	500	27274.06	1	3	1.252	4	0.992			
3664.590	200	10	27280.41	7							1.103
3664.108	300	10	27284.00	1							0.875
3663.201	800r	100	27290.75	7							1.170
3662.955	150	15	27292.59	2					0.180		
3662.750	300r	20	27294.12	1	3	1.181	4	0.849			
3662.283	100	3	27297.59	2					0.116		
3660.500	400	25	27310.89	1	1	1.496	2	1.186			
3660.091	200b	4	27313.95	3						0.523	
3659.629	600r	150	27317.39	2	2	0.737	3	1.087			
3658.808	400r	25	27323.52	2							1.468
3657.641	200s	10	27332.24	1	4	1.306	5	1.062			
3657.054	300	20	27336.63	2							1.206
3655.125	300	50	27351.05	1	3	1.250	4	1.066			
3654.461	500r	50	27356.02	3						0.084	
3653.916	20	1	27360.10	7							0.974
3653.586	40		27362.57	1					0.340		
3653.366	150	3	27364.22	7					0.050		
3651.570	100		27377.68	7							1.256
3650.204	3001	2	27387.93	3						0.136	
3649.735	1000r	100	27391.45	1	1		2		1.040		
3648.856	150	8	27398.04	3						0.165	
3647.932	300	50	27404.98	3						0.210	
3647.575	300	15	27407.67	7							1.025
3646.268	300	8b	27417.49	1	2	1.194	3	1.000			
3645.706	300	15	27421.72	2	4	0.877	5	1.177			
3645.566	200	8	27422.77	7							1.177
3644.018	100	2	27434.42	1					0.273		
3643.512	600r	50	27438.23	7							1.276
3642.573	500r	50	27445.30	1							0.870
3641.363	100b	4	27454.42	7							0.855
3640.924	200	15h	27457.73	3	1	0.823	1	1.178			
3640.390	100	3	27461.76	7					0.660		
3639.867	4001	40	27465.70	2	3	0.901	4	1.043			
3638.644	500r	50	27474.93	1					0.186		
3638.319	400r	25	27477.39	3						0.244	
3638.146	200	4	27478.69	3						0.531	
3637.772	50b		27481.52	7					0.080		
3636.834	500r	25	27488.60	7							1.481
3636.295	150	3	27492.68	3						0.277	
3635.943	2000r	150	27495.34	7							0.614
3634.902	200	4	27503.22	1					0.194		
3634.582	600r	100b	27505.64	7							0.914
3633.910	100	3	27510.73	7					0.180		
3632.830	1000r	100	27518.90	2							1.207
3632.124	100	5	27524.26	7					0.080		0.836
3631.863	150	8	27526.23	3						0.055	
3631.708	100b	2	27527.41	3						0.372	
3630.986	25	2	27532.88	7							1.003
3630.851	25		27533.90	7							0.963
3629.851	400	20	27541.49	3	1	0.716	1	1.486			
3629.017	100	4	27547.82	3						0.668	
3628.867	100	4	27548.95	3						0.276	
3628.129	75	4	27554.56	7							1.254
3628.032	100	10	27555.30	7							1.035
3627.342	50	1	27560.54	3						0.227	
3626.939	300	50	27563.60	7							0.942
3626.471	200	10	27567.16	2							1.326
3625.895	500r	400b	27571.53	5	2	0.833	2	1.177			
3625.150	300	75	27577.20	1					0.270		
3625.026	300r	100	27578.15	3						0.091	
3624.472	300	50	27582.36	3	4	1.066	4	1.305			
3624.143	100	3	27584.87	3						0.485	
3623.773	300	25	27587.68	2	2	0.778	3	1.190			
3623.297	15	1	27591.31	3						0.174	
3622.795	600r	50	27595.13	1	2		3				0.803
3622.338	500r	20b	27598.61	3		0.06		1.33			
3622.338	500r	20b	27598.61	1		1.48		1.19			
3622.290	500r	50	27598.98	2	2		3				1.550
3621.435	50		27605.49	1							0.422
3620.839	300r	50	27610.04	1	3	1.062	4	0.858			
3619.211	200	40	27622.45	3						0.090	
3617.671	300	8	27634.21	3	2	0.775	2	1.412			
3616.177	200	8	27645.63	1					0.206		
3615.849	200	4	27648.14	2					0.372		
3614.352	500r	75	27659.59	7							1.215
3614.212	500r	75	27660.66	7							0.978
3612.864	600r	5	27670.98	7					0.476		
3610.650	300	20	27687.95	2							1.485
3608.377	800r	50	27705.39	1	5	1.360	6	1.123			
3607.819	75		27709.67	1							0.749
3606.793	200	4	27717.55	7							1.295
3606.090	200	3	27722.96	7							1.471
3605.820	150	4	27725.03	1					0.300		0.274
3605.194	500	40	27729.85	1							0.770
3604.680	600r	40	27733.80	1	1	1.468	2	1.216			
3604.064	200b		27738.54	3	3	1.077	3	1.243			
3602.909	100	40	27747.43	1							0.802
3602.328	20		27751.91	7							1.040
3601.770	200	10	27756.21	3						0.313	
3601.295	300	10	27759.87	7							0.954
3600.431	300	50	27766.53	3	5	1.361	5	1.037			
3599.723	300	20	27771.99	3						0.095	
3598.691	100	5	27779.96	3						0.182	
3598.523	150	10	27781.25	1	3	1.192	4	0.861			
3597.704	20	2	27787.57	3						0.281	
3597.021	75	2	27792.86	7							1.395
3596.536	100	5	27796.60	7							1.443
3595.975	150	5	27800.94	7					0.244		
3595.756	100	10	27802.63	2					0.265		
3595.617	500r	100	27803.70	3						0.109	
3594.985	300	20	27808.59	1	2	1.723	3	1.267			
3594.721	300	10	27810.63	1	1	0.972	2	0.740			
3592.777	800r	100	27825.67	1	4				0.160		
3592.122	75		27830.76	2					0.160		1.402
3591.253	200	50	27837.49	3						0.200	
3590.924	300		27840.04	2	3	0.878	4	1.042			
3589.992	200	20	27847.27	3	1	1.175	1	1.483			
3587.831	100	5	27864.04	7							1.266
3587.102	150	40	27869.70	7							1.071
3586.703	100		27872.80	3						0.406	
3586.478	150	4	27874.55	1	1	0.986	2	0.666			
3585.244	150	5	27884.15	3						1.220	
3585.050	20		27885.65	1					0.080		0.775
3583.298	150	10	27899.29	2							1.130
3583.100	600r	25	27900.83	3						0.229	
3581.353	150	25b	27914.44	2					0.110		1.562
3581.289	100	3	27914.93	7							1.141
3579.454	200		27929.25	7					0.300		
3577.746	200	8	27942.58	1	2	1.296	3	1.064			
3574.192	50		27970.36	7							0.872
3572.067	300	20	27987.00	3						0.515	
3571.008	300	10	27995.30	3						0.201	
3570.827	100		27996.72	7							1.054
3570.523	300	10	27999.11	3						1.141	
3570.357	500	10	28000.41	3						1.404	
3569.820	1000r	25	28004.62	1	1	1.470	2	0.937			
3569.207	75		28009.43	3	2	1.014	2	1.282			
3566.099	15		28033.84	2					0.088		1.226
3565.822	300	15	28036.02	7					0.332		
3564.508	100	10	28046.35	7							1.102
3564.235	200	8	28048.50	7							1.125
3563.911	200		28051.05	7							1.130
3563.375	600	75b	28055.27	3						0.162	
3563.299	200b	25	28055.87	3						0.117	
3562.599	75	5	28061.38	7							1.155
3562.253	100	8	28064.11	7							0.994
3561.780	500r	75	28067.83	2	2	1.020	3	1.213			
3561.408	200	8	28070.77	3						0.207	
3560.896	300	40	28074.80	7							0.958
3560.688	200	5	28076.44	1					0.267		
3558.638	100		28092.61	2					0.320		
3556.973	75	10	28105.76	7							1.205
3555.013	1000r	50	28121.26	7							1.091
3554.336	75s	2	28126.61	7							1.078
3553.960	20	2	28129.59	3						0.784	
3553.110	100	200	28136.32	3						0.163	
3551.744	75	2	28147.14	7							0.948
3551.401	1000r	150	28149.86	2							1.489
3550.783	200	40	28154.76	3						0.163	
3550.718	100s		28155.28	1	1	0.064	2	1.064			
3548.664	200	8	28171.57	1	1	0.062	2	1.298			
3547.918	300	15	28177.49	3						0.136	
3547.338	200b		28182.10	3	2	0.951	2	1.251			
3546.255	20		28190.71	3						0.306	
3545.958	500b		28193.07	1							0.831
3544.250	150	3	28206.65	1					0.244		
3544.018	800r	50	28208.50	1	4	1.314	5	1.136			
3543.208	150	3	28214.95	3					0.667	0.667	
3543.023	200	4	28216.42	3						0.699	
3542.498	500r	50	28220.60	2	2	1.013	3	1.171			
3539.843	300	25	28241.77	3						1.015	
3536.976	150	4	28264.66	3						0.149	
3535.250	150b		28278.46	7							0.967
3534.675	150b		28283.06	1							0.706
3534.210	100b	10	28286.78	1	1	2.390	2	1.195			
3533.600	40	3	28291.66	7							1.089
3533.181	200b	10	28295.02	1	1	1.483	2	1.080			
3531.450	1000r	50	28308.89	3						0.186	
3531.282	200	8	28310.24	3	3	1.111	3	1.240			
3530.514	800r	50	28316.39	7							1.051
3529.385	3001	40	28325.45	1	3	1.082	4	0.732			
3528.411	300	50	28333.27	2							1.547
3527.793	200	10	28338.23	2	4	0.857	5	1.041			
3526.764	300		28346.50	3						0.222	
3526.634	500r	10	28347.54	3	2	0.736	2	1.544			
3524.708	300	8	28363.03	2							1.217
3524.178	300	25	28367.30	3						0.660	
3523.758	300	8	28370.69	7							0.996
3523.505	300	501	28372.72	1	1	1.812	2	0.738			
3523.022	75		28376.61	7							0.787
3521.059	500r	25	28 92.43	2	1	0.803	2	1.263			
3520.710	300	40	28395.24	3						1.151	
3518.885	4001	50	28409.97	3	2	0.788	2	1.206			
3517.961	2001	10	28417.43	3						0.194	
3517.257	40	3	28423.12	3						0.174	
3515.327	100	40	28438.72	7							1.025
3514.285	200	10	28447.16	7							0.974
3511.789	100	10h	28467.37	7							1.344
3511.157	800r	25	28472.50	3	2	1.075	2	1.249			
3510.729	75b		28475.96	7							1.125
3509.319	50		28487.41	7					0.060		
3509.089	500	25	28489.28	3	2	0.873	2	1.003			
3508.360	300	3	28495.20	1					1.183		
3508.100	300b		28497.30	3						0.133	
3507.520	100		28502.02	3						1.059	
3507.178	200	8	28504.80	3						0.133	
3506.345	300	10	28511.57	7							1.080
3506.132	300	3	28513.30	3	2	0.729	2	1.097			
3505.767	150	5	28516.27	1	1	1.475	2	1.025			
3504.992	300	8	28522.57	2	1	0.721	2	1.261			
3504.852	150	8	28523.71	1							0.745
3504.569	15		28526.02	3						1.254	
3503.786	500r	40	28532.40	1							0.948
3503.130	200	15	28537.74	7							1.136
3502.963	200	10	28539.10	7					0.104		
3499.821	200	5	28564.72	2	2	0.780	3	1.100			
3498.956	200b	20b	28571.78	3						0.322	
3498.621	800r	75	28574.51	3						0.358	
3496.811	500r	15	28589.31	2	2	0.746	3	1.179			
3496.060	200	10	28595.45	7					0.190		
3495.700	800r	25	28598.39	7							1.197
3494.801	100		28605.75	3	2	1.192	2	1.567			
3494.175	15	2	28610.87	3						0.334	
3492.158	25		28627.39	1					0.615		
3491.900	500	2	28629.51	2	3	1.017	4	1.210			
3490.848	50	1	28638.14	7					0.266		
3489.509	400	2	28649.13	1	1	1.076	2	0.730			
3488.834	500	3	28654.67	7							1.087
3487.991	1501	4	28661.60	3						0.539	
3486.551	1000r	10b	28673.44	3	2	0.738	2	1.123			
3486.269	50	1	28675.75	1							0.482
3486.210	50	1	28676.24	2	2	0.744	3	1.094			
3485.175	200b	2	28684.76	1							0.620
3484.732	200		28688.40	7					0.560		
3483.433	200b	1	28699.10	7							1.256
3481.620	100h		28714.04	7							1.108
3480.810	100		28720.72	7							0.940
3480.052	500	3	28726.98	7							1.581
3479.684	300	2	28730.02	1	2	1.195	3	1.072			
3478.612	150	1	28738.87	3						0.652	
3476.385	200	3	28757.28	2	3	0.860	4	1.010			
3474.530	200	3	28772.63	3						0.510	
3473.545	400	8	28780.79	3						1.515	
3472.691	150	4	28787.87	7							1.021
3471.959	500r	501	28793.94	7							1.090
3471.001	300	15	28801.89	2							1.442
3470.568	300	20	28805.48	3	4	1.120	4	1.308			
3469.345	1000r	150	28815.63	7							1.237
3465.061	200b	25	28851.25	3	2	0.776	2	1.159			
3464.882	75		28852.74	3						0.184	
3462.981	100	2	28868.58	7							1.118
3461.574	200	3	28880.32	1	3	1.240	4	1.065			
3459.907	20	2	28894.24	3						0.237	
3458.752	15		28903.88	3						1.247	
3458.142	200	8	28908.98	7							1.194
3457.829	75		28911.60	3						0.248	
3457.434	150	5	28914.90	3						0.158	
3457.069	400r	25	28917.95	3	2	0.734	2	0.949			
3456.695	200b	8	28921.08	7							1.133
3455.612	200b	10	28930.14	3						0.269	
3455.273	20	5	28932.98	3						0.193	
3454.206	200	15	28941.92	3						0.448	
3452.335	25		28957.61	7							0.970
3452.207	75	1	28958.68	7							1.014
3451.308	100		28966.22	1	1	1.276	2	1.016			
3450.948	100	15	28969.25	3						0.158	
3450.268	150	8	28974.95	3	2	1.267	2	1.707			
3448.949	300	10	28986.03	2	4	0.864	5	1.118			
3446.546	300	8	29006.24	2	3	0.793	4	1.213			
3444.795	100	3	29020.98	3	2	0.720	2	0.984			
3443.980	100	8	29027.86	3						1.435	
3443.497	1501	2	29031.93	7							0.784
3443.409	200s	3	29032.67	7						0.474	
3442.305	25	1	29041.98	3						0.167	
3441.036	100	20	29052.69	3						0.488	
3440.070	25	3	29060.84	7							0.916
3435.487	251		29099.61	7					0.231		
3434.727	200b	2	29106.05	7							0.997
3433.238	50		29118.68	7							0.822
3432.860	20	1	29121.88	7							0.590
3431.265	200	5	29135.42	7							0.816
3429.871	100b		29147.26	7							0.876
3429.574	200	10	29149.78	3						0.726	
3427.089	200s		29170.92	3	2	1.020	2	1.216			
3425.613	100	2	29183.49	3						0.966	
3425.435	150	15	29185.01	7							0.978
3425.140	200b	2	29187.52	3						0.302	
3421.678	200	4	29217.04	7							0.833
3420.372	200		29228.20	3						0.821	
3420.196	100	3	29229.70	7							1.080
3418.032	15		29248.21	3						0.462	
3417.498	400r	10	29252.78	3	2	0.733	2	0.978			
3416.595	150	3	29260.51	3						0.350	
3416.386	100b		29262.31	7							1.140
3415.885	500r	75	29266.60	3						0.282	
3414.299	75		29280.19	7							1.074
3412.181	200		29298.36	1					0.280		
3410.699	25		29311.09	3						0.236	
3410.184	150	3	29315.52	3	1	0.076	1	1.476			
3407.832	400b	10	29335.75	7							0.853
3406.978	10		29343.10	3						0.268	
3406.841	50		29344.29	7							1.066
3404.313	50		29366.08	3						0.263	
3403.903	200	8	29369.61	7							1.252
3400.680	25		29397.44	3						0.724	
3400.630	75	3	29397.88	3	1	1.109	1	2.408			
3399.041	100	4	29411.62	7							1.538
3398.545	1500r	50	29415.92	7							1.102
3395.986	200b	1	29438.08	7							0.959
3394.583	15	3	29450.24	3						0.332	
3393.993	200	2	29455.37	3						0.079	
3393.421	150	10	29460.33	7							1.036
3392.473	100	2	29468.57	7							1.168
3390.849	200	8	29482.67	7							1.043
3390.368	600r	75	29486.85	7							1.468
3389.038	300	8	29498.43	7							0.859
3388.348	50		29504.44	3						0.414	
3388.154	100b	5	29506.13	3						1.052	
3387.119	15		29515.14	7							0.949
3384.177	15		29540.80	3						0.495	
3383.586	200	8	29545.96	7							0.938
3382.829	15		29552.57	7							1.108
3381.907	25		29560.63	3						0.522	
3377.483	50	40	29599.35	1	1	2.407	2	1.351			
3375.587	200b		29615.97	2							1.623
3374.975	1500r	75	29621.34	7							1.283
3372.822	400r	100	29640.25	1							0.547
3370.887	100	5	29657.26	3						0.744	
3370.738	100	4	29658.58	7							0.482
3369.358	20		29670.73	7							1.192
3369.000	150	4	29673.87	3						0.427	
3368.318	10	3	29679.88	3						0.404	
3367.582	200r	10	29686.37	2	2	0.742	3	1.270			
3365.979	150	5b	29700.50	7							0.977
3365.337	400r	50	29706.17	2	2	0.724	3	1.084			
3365.137	300	15	29707.93	7							0.993
3364.154	20b	2	29716.62	2							1.294
3360.997	400r	150	29744.53	2	2	0.748	3	1.148			
3350.352	200	5	29839.04	3						0.193	
3348.288	40	1	29857.43	3	1	1.427	1	2.444			
3347.991	200	5	29860.08	7							1.018
3347.403	150		29865.32	1	4	1.303	5	1.078			
3345.170	150	3	29885.25	3						0.099	
3343.813	75s	3	29897.38	7							0.978
3343.499	150	5	29900.19	2							1.408
3343.344	200	10	29901.57	3						0.383	
3343.164	300	5	29903.19	2	2	1.244	3	1.004			
3342.898	20		29905.56	3						0.483	
3341.550	10	2	29917.63	3						1.118	
3341.205	50	2	29920.72	3						0.505	
3340.337	100b	1	29928.49	7							1.464
3340.021	15		29931.33	7							1.123
3339.257	15		29938.18	7							1.454
3337.275	200	5	29955.95	3						0.259	
3336.074	150		29966.74	3						0.607	
3335.694	150	4	29970.15	2	1	0.050	2	1.220			
3335.238	300	5	29974.24	2					0.210		
3332.479	100b	1	29999.06	7							0.952
3331.727	50	2	30005.83	7							1.074
3330.976	150	4	30012.60	7							0.998
3330.477	1500r	40	30017.10	2	2	0.733	3	1.129			
3329.728	300r	75	30023.85	3						0.705	
3327.527	50	2	30043.70	3						0.482	
3327.193	800r	25	30046.73	7							1.275
3322.093	400r	10	30092.85	7							1.139
3321.574	300r	40	30097.55	7							0.862
3320.879	20		30103.85	3						1.020	
3320.476	400r	25	30107.50	1							0.817
3320.301	600	500	30109.09	7							0.995
3319.572	50		30115.70	3						0.941	
3318.390	200	5	30126.43	7							0.993
3317.576	25		30133.82	7							1.051
3315.848	200	2	30149.52	2	1	0.733	2	1.027			
3314.793	400r		30159.12	7							1.044
3313.647	300r		30169.55	3						0.075	1.215
3313.437	50	4	30171.46	3						0.512	
3313.387	75b		30171.91	7							0.992
3313.073	2001	40	30174.77	2							1.575
3310.637	150d	15b	30196.98	3						0.587	
3310.446	100	10b	30198.72	7							1.298
3309.365	1000r	40	30208.58	3	2	0.744	2	1.095			
3305.303	400r	40	30245.71	3	1	0.846	1	1.481			
3304.238	3000r	200	30255.45	2	2	0.732	3	1.082			
3303.281	100	5	30264.22	7							1.048
3302.192	400r	5	30274.19	1							0.710
3301.651	1500r	150	30279.16	2							1.556
3300.775	75	4	30287.20	3						0.277	
3298.811	200	8	30305.22	1							0.809
3298.697	75	4	30306.27	3						0.178	
3296.372	150	8	30327.64	1					0.570		
3292.926	200	4	30359.39	3						0.635	
3291.410	50		30373.37	7					0.150		0.566
3291.037	401	1	30376.81	7							1.087
3289.517	100	10	30390.85	3						0.332	
3283.671	100	4	30444.95	3						0.314	
3282.837	40	2	30452.68	3						0.398	
3281.049	400r	25	30469.28	7							1.060
3280.764	150b	20	30471.92	7							0.719
3279.025	75	4	30488.09	7							0.974
3278.733	100	25	30490.80	2							1.753
3277.702	50	3	30500.39	7							1.145
3276.721	300	5	30509.52	3						0.480	
3276.225	150	8	30514.14	7							1.328
3276.045	50	5	30515.82	3						0.287	
3272.027	800r	75	30553.29	3	2	0.730	2	1.044			
3271.892	300r	100	30554.55	7							1.080
3271.546	50	3	30557.78	2					0.280		
3270.874	150	10	30564.06	3						0.496	
3270.100	100	5	30571.20	3						0.665	
3265.604	20		30613.38	3						0.401	
3263.183	400r	20	30636.09	3						0.262	
3262.316	75	4	30644.24	7							1.256
3261.497	20		30651.93	3						0.301	
3259.061	600r	50	30674.84	7							0.961
3258.103	200r		30683.84	7							1.275
3256.503	20	3	30698.93	7							0.942
3255.920	150	8	30704.43	2					0.176		
3251.916	2000r	200	30742.23	7							1.156
3249.855	800r	25	30761.73	2	2	0.741	3	1.193			
3248.697	75b		30772.69	3						0.283	
3247.331	15		30785.64	7							0.964
3247.218	75	4	30786.71	3						0.072	
3245.994	200	8	30798.32	3						1.043	
3244.043	150	10	30816.84	3						0.255	
3243.916	50	5	30818.05	3						0.290	
3242.145	50	15b	30834.88	7							0.896
3240.644	400r	40b	30849.17	7							1.121
3238.934	400r	20	30865.45	1	2	1.307	3	1.095			
3236.573	100	8b	30887.96	1					0.240		
3234.996	400r	20	30903.02	1							1.030
3231.461	75		30936.83	7							1.049
3225.007	100	5	30998.74	2	1	0.866	2	1.194			
3222.807	200	15	31019.89	7							1.215
3221.291	400b		31034.49	7							1.128
3220.986	75b		31037.43	2							0.796
3219.490	20h	4	31051.85	7							0.859
3216.998	75	3	31075.91	3						1.185	
3215.778	100	100	31087.69	7							0.915
3214.648	150	5	31098.62	3						0.871	
3214.381	400r	25	31101.20	3						0.400	
3214.076	400r	50	31104.15	7							1.010
3211.997	100r	5	31124.29	3						0.131	
3210.779	200	5	31136.09	7							1.172
3207.938	50r	5	31163.67	3	2	0.878	2	1.262			
3202.521	200r	8	31216.38	3						0.653	
3198.969	75s		31251.04	3						0.214	
3198.694	50b		31253.73	7							0.806
3195.688	400r	50	31283.12	2	2	0.750	3	1.120			
3193.781	200r	5	31301.80	1	1	1.619	2	1.259			
3192.586	400r	20	31313.52	7							0.920
3189.711	200	8	31341.74	3						0.416	
3189.562	200	3	31343.20	7							1.109
3185.628	200	3	31381.91	3						3.269	
3184.843	300	8	31389.64	3						0.320	
3184.277	300	15b	31395.22	1					0.243		
3183.384	100	2	31404.03	7							1.056
3181.670	500r	20	31420.95	3						0.546	
3180.194	1000r	1500	31435.53	7							1.077
3178.622	200	4	31451.07	3						0.241	
3178.244	600r	100	31454.82	7							1.028
3177.402	100	2	31463.15	3	1	1.268	1	1.462			
3176.167	200r	5	31475.38	7							1.130
3174.840	200	4	31488.54	3						0.660	
3174.020	300	8	31496.68	3						0.142	
3173.427	500r	50	31502.56	7					0.220		
3171.276	500r	75	31523.93	2	2	0.743	3	1.108			
3168.342	100	2	31553.11	7							0.852
3165.048	150	3	31585.95	2	1	0.076	2	1.073			
3161.364	200d	5	31622.76	1	3	1.078	4	0.898			
3161.148	100	2	31624.93	3						0.795	
3158.394	100		31652.50	3					0.456		
3157.221	300r	8	31664.25	7							0.952
3156.866	300		31667.81	1	1	1.466	2	1.221			
3152.395	100	3	31712.73	1	1	1.175	2	0.745			
3152.295	100	3	31713.74	3						0.235	
3151.184	300	5	31724.92	3						0.459	
3151.017	200		31726.60	3						0.203	
3150.536	100	8	31731.44	7							1.157
3147.449	200	25	31762.56	7							1.149
3146.310	200r	8	31774.07	7							1.256
3145.637	400r	25	31780.86	2	2	0.738	3	1.175			
3144.145	100	3	31795.94	3						0.102	
3142.422	100	5	31813.37	3						0.482	
3139.892	150	100	31839.00	3	4	0.968	4	1.220			
3138.200	100	10	31856.17	7							1.088
3136.829	500r	8	31870.09	3	2	0.730	2	0.941			
3136.216	1000r	100	31876.32	7							1.088
3134.203	400	5	31896.79	2							1.160
3132.102	300	5	31918.19	7							1.477
3126.414	200	2	31976.26	3						0.485	
3124.986	75	2	31990.87	7					0.180		
3120.881	400r	25	32032.94	7							1.498
3116.352	300r	10	32079.50	3	4	1.008	4	1.213			
3116.263	500r		32080.42	7							0.674
3111.280	300	8	32131.79	7							1.202
3108.636	200	3	32159.12	3						0.139	
3108.504	300r	10	32160.48	3					0.340		
3104.966	100r	3	32197.13	2	2	0.761	3	1.155			
3102.125	200	20	32226.61	7						0.383	
3099.184	100	2	32257.20	3						0.322	
3098.726	300r	5	32261.96	2	3	1.070	4	1.200			
3098.119	100		32268.28	3						0.600	
3093.719	200	3	32314.17	3						0.159	
3092.828	300r	8	32323.48	2							1.382
3082.507	100b		32431.71	7							1.624
3079.574	150	2	32462.60	3						0.825	
3078.625	150b	2	32472.61	7							1.017
3077.718	300r	5	32482.17	2							1.483
3076.410	150	30	32495.98	3	1	1.116	1	1.493			
3075.687	100	3	32503.61	3						0.956	
071.632	50		32546.53	7							1.225
3065.683	200r	2	32609.68	1					0.168		
3065.038	50	10	32616.54	7							1.164
3062.454	200r	2	32644.07	3						0.325	
3061.812	400r	3	32650.91	1					0.400		
3058.029	200r	2	32691.29	3						0.427	
3047.816	500r	2	32800.84	3						0.485	
3043.248	100	5	32850.07	7							1.928
3041.941	100		32864.19	7							1.121
3034.639	400r	2	32943.26	7							1.097
3032.016	100r	2	32971.76	3	1		1			0.700	
3031.195	100r	2	32980.68	3						0.654	
3030.487	400r	8	32988.39	3						0.218	
3029.477	100		32999.39	7							0.956
3027.896	150	3	33016.62	1					0.480		
3025.448	200r	2	33043.33	2	2	0.742	3	1.132			
3021.056	300	1	33091.37	7							1.401
3019.889	300		33104.15	7							1.028
3016.096	50		33145.79	7							1.297
3014.641	150r	1	33161.79	1					0.572		
3011.745	100s	1	33193.67	7							1.274
3008.924	200	2	33224.79	7							1.224
3006.634	200	2	33250.10	7							1.101
3006.164	300r	2	33255.29	7							1.455
3004.248	150r	1	33276.50	7							1.488
3003.210	200	1	33288.00	3						0.434	
3002.687	200r	2	33293.80	3						0.587	
3002.390	100r		33297.13	3						0.897	
2999.272	75	10	33331.71	3						0.329	
2998.147	200b	1	33344.21	3						0.507	
2997.196	100	8	33354.79	3						0.261	
2995.962	100	1	33368.53	7							0.803
2995.166	100	1	33377.40	7							0.862
2993.079	100		33400.67	7							0.966
2987.672	300r	2	33461.12	3						0.464	
2981.843	100	15	33526.52	7							0.788
2980.335	150	25	33543.48	7							1.399
2980.112	200r	2	33546.04	3						0.261	
2979.043	100b		33558.04	7							1.064
2973.675	100	1	33618.61	3						0.488	
2970.566	100	2	33653.79	7							1.150
2969.378	100	8	33667.26	3						0.618	
2963.608	75	8	33732.80	7					*s'*		1.178
2963.442	75		33734.69	3						0.844	
2959.854	300	25b	33775.58	7							0.881
2959.137	200	2	33783.77	3						0.466	
2958.367	100b	2	33792.57	7							1.374
2957.332	150	10	33804.39	7							1.194
2943.729	400	3	33960.59	3						0.837	
2931.783	400	4	34098.96	7							0.685
2925.526	500r	5	34171.89	7							0.843
2918.922	200	20b	34249.20	7							1.167
2916.369	500r	10	34279.18	7							0.766
2909.103	300d	8d	34364.79	7							0.990
2907.451	100		34384.32	7							1.194
2905.097	100b	2	34412.18	7							1.238
2890.078	150r	5	34591.00	1					0.703		
2889.488	50	2	34598.07	7							1.506
2888.785	200	2	34606.48	7							1.396
2888.552	200	3	34609.28	7							1.307
2886.983	150b		34628.09	3						0.318	
2883.266	400	3	34672.72	7							1.329
2881.577	200	10	34693.04	7							0.912
2876.768	300	3	34751.04	7							1.073
2862.610	300r	20	34922.90	7							1.143
2860.776	100	2	34945.29	3						0.356	
2860.491	150	3	34948.77	7					0.697		
2850.597	300b	2	35070.06	3						0.820	
2848.084	300r	2	35101.01	7							1.426
2846.036	150	2	35126.26	3						0.980	
2843.906	300r	2	35152.58	7							1.234
2841.383	150h	2	35183.79	7							1.087
2832.314	1000r	400	35296.44	7							0.962
2829.346	150	10	35333.46	7							1.196
2828.502	2001	4	35344.00	7							1.079
2825.909	100b		35376.44	7							1.011
2808.323	200	2	35597.96	7							1.191
2804.912	200	2	35641.24	3						0.684	
2799.432	100	1	35711.01	7							0.990
2762.458	50r		36188.96	3						1.301	
2747.139	150b		36390.74	7							0.665

**Table 4:** Classified lines of *Th I*

Wavelength (Å)	Intensity		Wavenumber (cm^ –1^)	Classification
	Lamp	Spark		
29662.822	0		3370.304	9804_5_ – $13175_{4}^{{}^{\circ}}$
28574.421	0		3498.679	12847_3_ – $16346_{4}^{{}^{\circ}}$
26734.663	0		3739.442	7502_3_ – $11241_{3}^{{}^{\circ}}$
26377.115	2		3790.131	$10414_{4}^{{}^{\circ}}$ – 14204_5_
25984.374	1		3847.417	18549_2_ – $22396_{1}^{{}^{\circ}}$
25971.332	0		3849.349	14204_5_ – $18053_{4}^{{}^{\circ}}$
25913.673	0		3857.914	$23113_{4}^{{}^{\circ}}$ – 26971_4_
25790.101	1		3876.399	$20522_{2}^{{}^{\circ}}$ – 24399_3_
25735.979	1		3884.551	13962_1_ – $17847_{2}^{{}^{\circ}}$
25561.646	1		3911.044	17166_5_ – $21077_{0}^{{}^{\circ}}$
25269.196	1		3956.308	$23015_{0}^{{}^{\circ}}$ – 26971_4_
25111.575	1		3981.141	$22399_{0}^{{}^{\circ}}$ – 26380_5_
25080.636	2		3986.052	$11877_{1}^{{}^{\circ}}$ – 15863_2_
25049.573	2		3990.995	$13175_{4}^{{}^{\circ}}$ – 17166_5_
25032.462	0		3993.723	$25690_{0}^{{}^{\circ}}$ – 29684_5_
24997.523	1		3999.305	13847_2_ – $17847_{2}^{{}^{\circ}}$
24973.732	1		4003.115	$26723_{6}^{{}^{\circ}}$ – 30726_7_
24931.344	1		4009.921	15493_4_ – $19503_{3}^{{}^{\circ}}$
24902.355	1		4014.589	$13945_{3}^{{}^{\circ}}$ – 17959_4_
24789.343	1		4032.891	$21890_{3}^{{}^{\circ}}$ – 25923_4_
24684.317	0		4050.050	$19227_{6}^{{}^{\circ}}$ – 23277_5_
24516.271	1		4077.811	$23113_{4}^{{}^{\circ}}$ – 27191_5_
24486.763	0		4082.725	$24259_{4}^{{}^{\circ}}$ – 28342_5_
24470.262	0		4085.478	$23481_{1}^{{}^{\circ}}$ – 27566_2_
24428.586	1		4092.448	$22163_{4}^{{}^{\circ}}$ – 26255_4_
24420.077	1		4093.874	$22877_{0}^{{}^{\circ}}$ – 26971_4_
24329.294	0		4109.150	$25575_{4}^{{}^{\circ}}$ – 29684_5_
24301.853	1		4113.790	13297_4_ – $17411_{3}^{{}^{\circ}}$
24262.390	1		4120.481	18549_2_ – $22669_{3}^{{}^{\circ}}$
24216.068	0		4128.363	21575_2_ – $25703_{2}^{{}^{\circ}}$
24172.879	2		4135.739	13088_3_ – $17224_{2}^{{}^{\circ}}$
24171.733	0		4135.935	11601_1_ – $15736_{1}^{{}^{\circ}}$
24138.665	0		4141.601	$24421_{3}^{{}^{\circ}}$ – 28562_4_
24136.264	3		4142.013	$20522_{2}^{{}^{\circ}}$ – 24664_3_
24120.715	1		4144.683	$17501_{0}^{{}^{\circ}}$ – 21645_4_
24007.982	4		4164.145	6362_2_ – $10526_{3}^{{}^{\circ}}$
23940.033	0		4175.964	$24182_{2}^{{}^{\circ}}$ – 28358_3_
23907.149	1		4181.708	17959_4_ – $22141_{3}^{{}^{\circ}}$
23865.994	0		4188.919	$22855_{3}^{{}^{\circ}}$ – 27044_3_
23781.839	4		4203.742	13297_4_ – $17501_{0}^{{}^{\circ}}$
23712.604	0		4216.016	$25336_{6}^{{}^{\circ}}$ – 29552_6_
23704.794	1		4217.405	$22163_{4}^{{}^{\circ}}$ – 26380_5_
23672.844	1		4223.097	$18809_{4}^{{}^{\circ}}$ – 23032_4_
23671.785	1		4223.286	$13175_{4}^{{}^{\circ}}$ – 17398_3_
23663.487	1		4224.767	$14206_{4}^{{}^{\circ}}$ – 18431_3_
23608.338	0		4234.636	$17411_{3}^{{}^{\circ}}$ – 21645_4_
23588.463	1		4238.204	18431_3_ – $22669_{3}^{{}^{\circ}}$
23551.636	1		4244.831	15970_3_ – $20214_{3}^{{}^{\circ}}$
23514.732	5		4251.493	$11241_{3}^{{}^{\circ}}$ – 15493_4_
23477.705	0		4258.198	$24421_{3}^{{}^{\circ}}$ – 28679_2_
23403.546	0		4271.691	$18930_{3}^{{}^{\circ}}$ – 23201_3_
23330.692	2		4285.030	$26645_{0}^{{}^{\circ}}$ – 30930_6_
23314.380	1		4288.028	$22508_{2}^{{}^{\circ}}$ – 26796_3_
23297.038	0		4291.220	$24850_{6}^{{}^{\circ}}$ – 29141_5_
23270.076	2		4296.192	$11197_{0}^{{}^{\circ}}$ – 15493_4_
23217.661	1		4305.891	18549_2_ – $22855_{3}^{{}^{\circ}}$
23175.469	3		4313.730	16554_6_ – $20867_{7}^{{}^{\circ}}$
23153.597	4		4317.805	$15736_{1}^{{}^{\circ}}$ – 20054_2_
23127.608	1		4322.657	13088_3_ – $17411_{3}^{{}^{\circ}}$
23121.211	0		4323.853	$25690_{0}^{{}^{\circ}}$ – 30014_4_
23085.081	0		4330.620	$14243_{1}^{{}^{\circ}}$ – 18574_1_
23007.044	1		4345.309	$27948_{4}^{{}^{\circ}}$ – 32293_5_
22978.346	0		4350.736	$17224_{2}^{{}^{\circ}}$ – 21575_2_
22964.759	1		4353.310	$21902_{4}^{{}^{\circ}}$ – 26255_4_
22900.906	2		4365.448	$21890_{3}^{{}^{\circ}}$ – 26255_4_
22900.565	0		4365.513	$15166_{3}^{{}^{\circ}}$ – 19532_4_
22875.089	0		4370.375	$17224_{2}^{{}^{\circ}}$ – 21594_3_
22864.133	2		4372.469	$25180_{7}^{{}^{\circ}}$ – 29552_6_
22860.040	0		4373.252	$24561_{3}^{{}^{\circ}}$ – 28934_3_
22855.180	2		4374.182	$18614_{1}^{{}^{\circ}}$ – 22988_2_
22851.632	3		4374.861	8800_4_ – $13175_{4}^{{}^{\circ}}$
22843.962	1		4376.330	12847_3_ – $17224_{2}^{{}^{\circ}}$
22834.596	2		4378.125	3865_1_ – $8243_{2}^{{}^{\circ}}$
22829.522	2		4379.098	17959_4_ – $22338_{3}^{{}^{\circ}}$
22806.940	0		4383.434	$21539_{4}^{{}^{\circ}}$ – 25923_4_
22785.721	1		4387.516	14226_0_ – $18614_{1}^{{}^{\circ}}$
22760.862	0		4392.308	$20522_{2}^{{}^{\circ}}$ – 24915_3_
22750.249	1		4394.357	18699_2_ – $23093_{2}^{{}^{\circ}}$
22723.153	0		4399.597	$14032_{2}^{{}^{\circ}}$ – 18431_3_
22710.196	3		4402.107	9804_5_ – $14206_{4}^{{}^{\circ}}$
22646.195	3		4414.548	11802_2_ – $16217_{2}^{{}^{\circ}}$
22616.700	1		4420.305	13962_1_ – $18382_{0}^{{}^{\circ}}$
22614.383	4		4420.758	6362_2_ – $10783_{2}^{{}^{\circ}}$
22544.420	1		4434.477	$25321_{3}^{{}^{\circ}}$ – 29756_4_
22537.763	3		4435.787	$15618_{3}^{{}^{\circ}}$ – 20054_2_
22518.913	0		4439.500	17959_4_ – $22399_{0}^{{}^{\circ}}$
22513.933	1		4440.482	$24701_{0}^{{}^{\circ}}$ – 29141_5_
22496.819	2		4443.860	$19588_{0}^{{}^{\circ}}$ – 24032_4_
22446.273	0		4453.867	21594_3_ – $26048_{4}^{{}^{\circ}}$
22439.688	0		4455.174	15493_4_ – $19948_{4}^{{}^{\circ}}$
22437.386	1		4455.631	$14243_{1}^{{}^{\circ}}$ – 18699_2_
22377.781	1		4467.499	$18809_{4}^{{}^{\circ}}$ – 23277_5_
22350.905	0		4472.871	$23093_{2}^{{}^{\circ}}$ – 27566_2_
22344.755	2		4474.102	$11877_{1}^{{}^{\circ}}$ – 16351_0_
22323.993	0		4478.263	$21902_{4}^{{}^{\circ}}$ – 26380_5_
22318.327	1		4479.400	19273_2_ – $23752_{2}^{{}^{\circ}}$
22283.623	0		4486.376	$13945_{3}^{{}^{\circ}}$ – 18431_3_
22215.926	1		4500.047	18549_2_ – $23049_{1}^{{}^{\circ}}$
22115.259	0		4520.531	$23752_{2}^{{}^{\circ}}$ – 28273_2_
22103.641	4		4522.907	16554_6_ – $21077_{0}^{{}^{\circ}}$
22082.481	1		4527.241	$25321_{3}^{{}^{\circ}}$ – 29849_4_
22073.514	1		4529.080	$19503_{3}^{{}^{\circ}}$ – 24032_4_
22029.034	0		4538.225	21575_2_ – $26113_{2}^{{}^{\circ}}$
21990.719	2		4546.132	$15166_{3}^{{}^{\circ}}$ – 19713_3_
21987.769	0		4546.742	19713_3_ – $24259_{4}^{{}^{\circ}}$
21987.769	0		4546.742	$26882_{0}^{{}^{\circ}}$ – 31429_1_
21986.275	0		4547.051	21143_5_ – $25690_{0}^{{}^{\circ}}$
21959.399	1		4552.616	15970_3_ – $20522_{2}^{{}^{\circ}}$
21952.080	1		4554.134	$17847_{2}^{{}^{\circ}}$ – 22401_1_
21944.958	4		4555.612	3687_2_ – $8243_{2}^{{}^{\circ}}$
21925.759	2		4559.601	15863_2_ – $20423_{1}^{{}^{\circ}}$
21883.548	0		4568.396	$18069_{3}^{{}^{\circ}}$ – 22637_3_
21862.515	1		4572.791	$23655_{4}^{{}^{\circ}}$ – 28227_4_
21809.793	0		4583.845	$18053_{4}^{{}^{\circ}}$ – 22637_3_
21756.165	1		4595.144	17073_1_ – $21668_{1}^{{}^{\circ}}$
21749.619	1		4596.527	15970_3_ – $20566_{4}^{{}^{\circ}}$
21747.329	1		4597.011	$17501_{0}^{{}^{\circ}}$ – 22098_4_
21743.999	5		4597.715	7280_2_ – $11877_{1}^{{}^{\circ}}$
21712.571	4		4604.370	$8243_{2}^{{}^{\circ}}$ – 12847_3_
21706.674	1		4605.621	14204_5_ – $18809_{4}^{{}^{\circ}}$
21706.264	1		4605.708	$23752_{2}^{{}^{\circ}}$ – 28358_3_
21683.632	0		4610.515	21143_5_ – $25753_{0}^{{}^{\circ}}$
21676.298	0		4612.075	7502_3_ – $12114_{2}^{{}^{\circ}}$
21655.755	2		4616.450	11601_1_ – $16217_{2}^{{}^{\circ}}$
21628.998	4		4622.161	$11241_{3}^{{}^{\circ}}$ – 15863_2_
21605.654	1		4627.155	$24307_{2}^{{}^{\circ}}$ – 28934_3_
21599.082	1		4628.563	22637_3_ – $27266_{4}^{{}^{\circ}}$
21580.898	0		4632.463	$22338_{3}^{{}^{\circ}}$ – 26971_4_
21556.115	0		4637.789	$21252_{2}^{{}^{\circ}}$ – 25890_2_
21527.563	0		4643.940	$22877_{0}^{{}^{\circ}}$ – 27521_4_
21519.111	0		4645.764	$23916_{4}^{{}^{\circ}}$ – 28562_4_
21472.877	1		4655.767	$25355_{4}^{{}^{\circ}}$ – 30011_3_
21442.806	1		4662.296	18431_3_ – $23093_{2}^{{}^{\circ}}$
21424.751	0		4666.225	$22855_{3}^{{}^{\circ}}$ – 27521_4_
21418.725	0		4667.538	$14032_{2}^{{}^{\circ}}$ – 18699_2_
21415.242	2		4668.297	19713_3_ – $24381_{2}^{{}^{\circ}}$
21406.777	1		4670.143	19532_4_ – $24202_{4}^{{}^{\circ}}$
21375.118	3		4677.060	9804_5_ – $14481_{6}^{{}^{\circ}}$
21353.185	3		4681.864	18431_3_ – $23113_{4}^{{}^{\circ}}$
21328.149	0		4687.360	$23655_{4}^{{}^{\circ}}$ – 28342_5_
21319.166	1		4689.335	$22877_{1}^{{}^{\circ}}$ – 27566_2_
21260.824	0		4702.203	$24850_{6}^{{}^{\circ}}$ – 29552_6_
21256.077	0		4703.253	$23655_{4}^{{}^{\circ}}$ – 28358_3_
21250.077	0		4704.581	$25306_{2}^{{}^{\circ}}$ – 30011_3_
21247.182	0		4705.222	$22338_{3}^{{}^{\circ}}$ – 27044_3_
21239.694	1		4706.881	$23521_{3}^{{}^{\circ}}$ – 28227_4_
21203.367	0		4714.945	20054_2_ – $24769_{3}^{{}^{\circ}}$
21198.664	0		4715.991	$21539_{4}^{{}^{\circ}}$ – 26255_4_
21173.002	1		4721.707	15493_4_ – $20214_{3}^{{}^{\circ}}$
21148.510	3		4727.175	$23306_{6}^{{}^{\circ}}$ – 28034_5_
21143.170	4		4728.369	$11241_{3}^{{}^{\circ}}$ – 15970_3_
21132.654	2		4730.722	$19039_{2}^{{}^{\circ}}$ – 23769_1_
21117.677	0		4734.077	21143_5_ – $25877_{4}^{{}^{\circ}}$
21108.367	1		4736.165	17166_5_ – $21902_{4}^{{}^{\circ}}$
21095.446	0		4739.066	15493_4_ – $20232_{4}^{{}^{\circ}}$
21095.446	0		4739.066	21645_4_ – $26384_{4}^{{}^{\circ}}$
21019.525	2		4756.183	13297_4_ – $18053_{4}^{{}^{\circ}}$
21011.808	2		4757.930	$21165_{3}^{{}^{\circ}}$ – 25923_4_
21009.229	1		4758.514	13088_3_ – $17847_{2}^{{}^{\circ}}$
20981.790	0		4764.737	17398_3_ – $22163_{4}^{{}^{\circ}}$
20973.743	1		4766.565	13847_2_ – $18614_{1}^{{}^{\circ}}$
20965.562	1		4768.425	21594_3_ – $26363_{2}^{{}^{\circ}}$
20951.480	1		4771.630	13297_4_ – $18069_{3}^{{}^{\circ}}$
20920.513	1		4778.693	$26363_{2}^{{}^{\circ}}$ – 31141_3_
20893.881	0		4784.784	$13175_{4}^{{}^{\circ}}$ – 17959_4_
20879.577	1		4788.062	21575_2_ – $26363_{2}^{{}^{\circ}}$
20869.452	4		4790.385	$17847_{2}^{{}^{\circ}}$ – 22637_3_
20863.115	0		4791.840	$23481_{1}^{{}^{\circ}}$ – 28273_2_
20862.579	2		4791.963	$22399_{0}^{{}^{\circ}}$ – 27191_5_
20833.885	1		4798.563	$25753_{0}^{{}^{\circ}}$ – 30552_4_
20792.737	1		4808.059	$14465_{2}^{{}^{\circ}}$ – 19273_2_
20735.100	0		4821.424	18699_2_ – $23521_{3}^{{}^{\circ}}$
20717.817	0		4825.446	20054_2_ – $24880_{1}^{{}^{\circ}}$
20716.744	2		4825.696	$22669_{3}^{{}^{\circ}}$ – 27495_4_
20715.963	0		4825.878	$25306_{2}^{{}^{\circ}}$ – 30132_2_
20702.372	0		4829.046	15493_4_ – $20322_{0}^{{}^{\circ}}$
20681.631	0		4833.889	$24850_{6}^{{}^{\circ}}$ – 29684_5_
20680.125	4		4834.241	7280_2_ – $12114_{2}^{{}^{\circ}}$
20661.764	1		4838.537	$20566_{4}^{{}^{\circ}}$ – 25405_4_
20651.478	0		4840.947	$21539_{4}^{{}^{\circ}}$ – 26380_5_
20634.364	6		4844.962	$8243_{2}^{{}^{\circ}}$ – 13088_3_
20618.818	0		4848.615	19713_3_ – $24561_{3}^{{}^{\circ}}$
20610.588	1		4850.551	17398_3_ – $22248_{2}^{{}^{\circ}}$
20605.987	2		4851.634	$22669_{3}^{{}^{\circ}}$ – 27521_4_
20584.349	0		4856.734	$24561_{3}^{{}^{\circ}}$ – 29418_2_
20575.042	0		4858.931	$21252_{2}^{{}^{\circ}}$ – 26111_1_
20562.003	0		4862.012	$16783_{4}^{{}^{\circ}}$ – 21645_4_
20561.348	2		4862.167	21645_4_ – $26508_{3}^{{}^{\circ}}$
20541.715	1		4866.814	$24274_{0}^{{}^{\circ}}$ – 29141_5_
20513.976	0		4873.395	15863_2_ – $20737_{1}^{{}^{\circ}}$
20489.032	1		4879.328	6362_2_ – $11241_{3}^{{}^{\circ}}$
20478.858	1		4881.752	$24259_{4}^{{}^{\circ}}$ – 29141_5_
20453.217	0		4887.872	$15166_{3}^{{}^{\circ}}$ – 20054_2_
20450.008	1		4888.639	19532_4_ – $24421_{3}^{{}^{\circ}}$
20428.437	1		4893.801	$21902_{4}^{{}^{\circ}}$ – 26796_3_
20421.761	3		4895.401	17959_4_ – $22855_{3}^{{}^{\circ}}$
20419.379	0		4895.972	$19503_{3}^{{}^{\circ}}$ – 24399_3_
20392.064	0		4902.530	$26363_{2}^{{}^{\circ}}$ – 31265_3_
20391.719	0		4902.613	$22141_{3}^{{}^{\circ}}$ – 27044_3_
20381.309	3		4905.117	21143_5_ – $26048_{4}^{{}^{\circ}}$
20377.894	1		4905.939	$21890_{3}^{{}^{\circ}}$ – 26796_3_
20374.476	1		4906.762	18574_1_ – $23481_{1}^{{}^{\circ}}$
20321.907	0		4919.455	$18069_{3}^{{}^{\circ}}$ – 22988_2_
20317.777	4		4920.455	$23113_{4}^{{}^{\circ}}$ – 28034_5_
20263.141	0		4933.722	$26995_{3}^{{}^{\circ}}$ – 31929_3_
20234.945	0		4940.597	17398_3_ – $22338_{3}^{{}^{\circ}}$
20205.662	0		4947.757	$25703_{2}^{{}^{\circ}}$ – 30651_3_
20191.383	3		4951.256	$19039_{2}^{{}^{\circ}}$ – 23990_2_
20183.242	0		4953.253	$27087_{1}^{{}^{\circ}}$ – 32041_2_
20158.039	2		4959.446	$12114_{2}^{{}^{\circ}}$ – 17073_1_
20139.866	1		4963.921	$18069_{3}^{{}^{\circ}}$ – 23032_4_
20128.687	3		4966.678	$10526_{3}^{{}^{\circ}}$ – 15493_4_
20122.241	0		4968.269	$20922_{2}^{{}^{\circ}}$ – 25890_2_
20108.593	2		4971.641	18549_2_ – $23521_{3}^{{}^{\circ}}$
20077.401	2		4979.365	$18053_{4}^{{}^{\circ}}$ – 23032_4_
20047.945	2		4986.681	$24769_{3}^{{}^{\circ}}$ – 29756_4_
19998.121	1		4999.105	12847_3_ – $17847_{2}^{{}^{\circ}}$
19935.599	0		5014.783	$27948_{4}^{{}^{\circ}}$ – 32963_5_
19922.803	1		5018.004	$23916_{4}^{{}^{\circ}}$ – 28934_3_
19919.437	0		5018.852	$23015_{0}^{{}^{\circ}}$ – 28034_5_
19908.520	0		5021.604	$18011_{0}^{{}^{\circ}}$ – 23032_4_
19902.706	4		5023.071	14204_5_ – $19227_{6}^{{}^{\circ}}$
19882.301	2		5028.226	$22163_{4}^{{}^{\circ}}$ – 27191_5_
19862.586	0		5033.217	$24981_{3}^{{}^{\circ}}$ – 30014_4_
19857.666	2		5034.464	19273_2_ – $24307_{2}^{{}^{\circ}}$
19852.906	2		5035.671	$23306_{6}^{{}^{\circ}}$ – 28342_5_
19850.778	1		5036.211	$25336_{6}^{{}^{\circ}}$ – 30372_6_
19847.455	0		5037.054	$24381_{2}^{{}^{\circ}}$ – 29418_2_
19829.520	1		5041.610	$23521_{3}^{{}^{\circ}}$ – 28562_4_
19797.857	0		5049.673	$23481_{1}^{{}^{\circ}}$ – 28531_2_
19786.004	5		5052.698	$7795_{4}^{{}^{\circ}}$ – 12847_3_
19784.595	1		5053.058	18699_2_ – $23752_{2}^{{}^{\circ}}$
19770.799	1		5056.584	$24084_{6}^{{}^{\circ}}$ – 29141_5_
19765.178	3		5058.022	$21738_{2}^{{}^{\circ}}$ – 26796_3_
19764.361	0		5058.231	15863_2_ – $20922_{2}^{{}^{\circ}}$
19762.115	0		5058.806	$22508_{2}^{{}^{\circ}}$ – 27566_2_
19757.006	0		5060.114	$18930_{3}^{{}^{\circ}}$ – 23990_2_
19741.428	5		5064.107	8111_4_ – $13175_{4}^{{}^{\circ}}$
19736.666	0		5065.329	$24769_{3}^{{}^{\circ}}$ – 29835_3_
19733.467	1		5066.150	$26363_{2}^{{}^{\circ}}$ – 31429_1_
19721.637	0		5069.189	$21902_{4}^{{}^{\circ}}$ – 26971_4_
19705.256	0		5073.403	15493_4_ – $20566_{4}^{{}^{\circ}}$
19696.195	1		5075.737	$23603_{2}^{{}^{\circ}}$ – 28679_2_
19692.738	1		5076.628	13962_1_ – $19039_{2}^{{}^{\circ}}$
19683.215	4		5079.084	$10414_{4}^{{}^{\circ}}$ – 15493_4_
19676.823	1		5080.734	$10783_{2}^{{}^{\circ}}$ – 15863_2_
19674.535	3		5081.325	$21890_{3}^{{}^{\circ}}$ – 26971_4_
19669.905	2		5082.521	13847_2_ – $18930_{3}^{{}^{\circ}}$
19668.461	4		5082.894	20322_0_ – 25405_4_
19643.457	1		5089.364	18431_3_ – $23521_{3}^{{}^{\circ}}$
19639.120	1		5090.488	$21165_{3}^{{}^{\circ}}$ – 26255_4_
19622.548	0		5094.787	$22248_{2}^{{}^{\circ}}$ – 27343_3_
19617.154	2		5096.188	$22399_{0}^{{}^{\circ}}$ – 27495_4_
19595.083	3		5101.928	$18930_{3}^{{}^{\circ}}$ – 24032_4_
19586.250	0		5104.229	$22508_{2}^{{}^{\circ}}$ – 27612_3_
19571.602	2		5108.049	19273_2_ – $24381_{2}^{{}^{\circ}}$
19561.688	2		5110.638	$24307_{2}^{{}^{\circ}}$ – 29418_2_
19535.499	0		5117.489	$26878_{3}^{{}^{\circ}}$ – 31995_4_
19519.076	0		5121.795	$27674_{2}^{{}^{\circ}}$ – 32796_3_
19505.411	0		5125.383	$22855_{3}^{{}^{\circ}}$ – 27980_3_
19476.782	1		5132.917	$18069_{3}^{{}^{\circ}}$ – 23201_3_
19444.483	1		5141.443	$17847_{2}^{{}^{\circ}}$ – 22988_2_
19430.829	2		5145.056	8800_4_ – $13945_{3}^{{}^{\circ}}$
19420.664	1		5147.749	$19516_{2}^{{}^{\circ}}$ – 24664_3_
19418.336	1		5148.366	$18053_{4}^{{}^{\circ}}$ – 23201_3_
19400.479	3		5153.105	$20737_{1}^{{}^{\circ}}$ – 25890_2_
19396.794	2		5154.084	$21890_{3}^{{}^{\circ}}$ – 27044_3_
19391.319	1		5155.539	$18614_{1}^{{}^{\circ}}$ – 23769_1_
19388.002	1		5156.421	$22877_{0}^{{}^{\circ}}$ – 28034_5_
19345.173	0		5167.837	22098_4_ – $27266_{4}^{{}^{\circ}}$
19342.209	2		5168.629	19532_4_ – $24701_{0}^{{}^{\circ}}$
19336.983	0		5170.026	$22396_{1}^{{}^{\circ}}$ – 27566_2_
19321.626	0		5174.135	$26363_{2}^{{}^{\circ}}$ – 31537_3_
19317.885	3		5175.137	17073_1_ – $22248_{2}^{{}^{\circ}}$
19291.493	1		5182.217	$23752_{2}^{{}^{\circ}}$ – 28934_3_
19290.328	0		5182.530	$22338_{3}^{{}^{\circ}}$ – 27521_4_
19273.916	2		5186.943	$10783_{2}^{{}^{\circ}}$ – 15970_3_
19264.749	1		5189.411	$20922_{2}^{{}^{\circ}}$ – 26111_1_
19261.710	0		5190.230	$20214_{3}^{{}^{\circ}}$ – 25405_4_
19261.001	0		5190.421	$23655_{4}^{{}^{\circ}}$ – 28845_4_
19257.450	0		5191.378	13847_2_ – $19039_{2}^{{}^{\circ}}$
19256.382	1		5191.666	18549_2_ – $23741_{1}^{{}^{\circ}}$
19244.968	0		5194.745	$24561_{3}^{{}^{\circ}}$ – 29756_4_
19244.035	1		5194.997	15970_3_ – $21165_{3}^{{}^{\circ}}$
19241.246	0		5195.750	21594_3_ – $26790_{4}^{{}^{\circ}}$
19240.420	0		5195.973	$11877_{1}^{{}^{\circ}}$ – 17073_1_
19204.660	1		5205.648	12847_3_ – $18053_{4}^{{}^{\circ}}$
19190.670	2		5209.443	$27084_{6}^{{}^{\circ}}$ – 32293_5_
19178.389	2		5212.779	$23015_{0}^{{}^{\circ}}$ – 28227_4_
19162.549	1		5217.088	$26048_{4}^{{}^{\circ}}$ – 31265_3_
19151.815	4		5220.012	5563_1_ – $10783_{2}^{{}^{\circ}}$
19147.846	3		5221.094	12847_3_ – $18069_{3}^{{}^{\circ}}$
19143.281	1		5222.339	$18809_{4}^{{}^{\circ}}$ – 24032_4_
19138.077	1		5223.759	$18053_{4}^{{}^{\circ}}$ – 23277_5_
19138.077	1		5223.759	$23049_{1}^{{}^{\circ}}$ – 28273_2_
19113.116	2		5230.581	$25321_{3}^{{}^{\circ}}$ – 30552_4_
19091.898	0		5236.394	$23609_{0}^{{}^{\circ}}$ – 28845_4_
19088.610	3		5237.296	19532_4_ – $24769_{3}^{{}^{\circ}}$
19080.012	1		5239.656	$24182_{2}^{{}^{\circ}}$ – 29422_1_
19078.887	1		5239.965	$25690_{0}^{{}^{\circ}}$ – 30930_6_
19074.406	3		5241.196	$14032_{2}^{{}^{\circ}}$ – 19273_2_
19073.289	2		5241.503	21143_5_ – $26384_{4}^{{}^{\circ}}$
19050.366	0		5247.810	$14465_{2}^{{}^{\circ}}$ – 19713_3_
19035.208	0		5251.989	20054_2_ – $25306_{2}^{{}^{\circ}}$
19033.646	1		5252.420	$24880_{1}^{{}^{\circ}}$ – 30132_2_
19018.615	2		5256.571	$13175_{4}^{{}^{\circ}}$ – 18431_3_
18984.562	0		5266.000	$18011_{0}^{{}^{\circ}}$ – 23277_5_
18964.790	1		5271.490	17398_3_ – $22669_{3}^{{}^{\circ}}$
18958.367	1		5273.276	$22338_{3}^{{}^{\circ}}$ – 27612_3_
18957.939	0		5273.395	$24561_{3}^{{}^{\circ}}$ – 29835_3_
18942.594	0		5277.667	$26651_{2}^{{}^{\circ}}$ – 31929_3_
18942.109	1		5277.802	$24274_{0}^{{}^{\circ}}$ – 29552_6_
18938.047	1		5278.934	$23603_{2}^{{}^{\circ}}$ – 28882_2_
18925.238	0		5282.507	15970_3_ – $21252_{2}^{{}^{\circ}}$
18919.776	2		5284.032	$12114_{2}^{{}^{\circ}}$ – 17398_3_
18907.334	2		5287.509	$24561_{3}^{{}^{\circ}}$ – 29849_4_
18904.263	0		5288.368	19273_2_ – $24561_{3}^{{}^{\circ}}$
18893.288	0		5291.440	$27260_{3}^{{}^{\circ}}$ – 32551_3_
18886.685	6		5293.290	$7795_{4}^{{}^{\circ}}$ – 13088_3_
18885.154	1		5293.719	$24838_{1}^{{}^{\circ}}$ – 30132_2_
18865.586	3		5299.210	$16346_{4}^{{}^{\circ}}$ – 21645_4_
18858.490	1		5301.204	15863_2_ – $21165_{3}^{{}^{\circ}}$
18850.731	2		5303.386	$21077_{0}^{{}^{\circ}}$ – 26380_5_
18840.854	0		5306.166	$21738_{2}^{{}^{\circ}}$ – 27044_3_
18824.439	0		5310.793	$22669_{3}^{{}^{\circ}}$ – 27980_3_
18811.879	7		5314.339	$16783_{4}^{{}^{\circ}}$ – 22098_4_
18802.414	3		5317.014	16351_0_ – $21668_{1}^{{}^{\circ}}$
18799.282	0		5317.900	$22248_{2}^{{}^{\circ}}$ – 27566_2_
18788.340	0		5320.997	18431_3_ – $23752_{3}^{{}^{\circ}}$
18781.224	1		5323.013	17073_1_ – $22396_{1}^{{}^{\circ}}$
18772.446	3		5325.502	$14206_{4}^{{}^{\circ}}$ – 19532_4_
18763.740	0		5327.973	$13945_{3}^{{}^{\circ}}$ – 19273_2_
18737.838	0		5335.338	$24421_{3}^{{}^{\circ}}$ – 29756_4_
18730.785	2		5337.347	$10526_{3}^{{}^{\circ}}$ – 15863_2_
18689.399	0		5349.166	16554_6_ – $21903_{7}^{{}^{\circ}}$
18685.648	0		5350.240	$26645_{0}^{{}^{\circ}}$ – 31995_4_
18685.274	1		5350.347	$22877_{0}^{{}^{\circ}}$ – 28227_4_
18664.162	2		5356.399	$20566_{4}^{{}^{\circ}}$ – 25923_4_
18657.231	2		5358.389	$22163_{4}^{{}^{\circ}}$ – 27521_4_
18651.748	0		5359.964	$19039_{2}^{{}^{\circ}}$ – 24399_3_
18625.408	0		5367.544	$26645_{0}^{{}^{\circ}}$ – 32012_4_
18595.874	3		5376.069	$18614_{1}^{{}^{\circ}}$ – 23990_2_
18557.981	3		5387.046	$18382_{0}^{{}^{\circ}}$ – 23769_1_
18546.830	1		5390.285	$25336_{6}^{{}^{\circ}}$ – 30726_7_
18520.188	0		5398.039	$19516_{2}^{{}^{\circ}}$ – 24915_3_
18509.757	1		5401.081	$23481_{1}^{{}^{\circ}}$ – 28882_2_
18490.644	4		5406.664	8800_4_ – $14206_{4}^{{}^{\circ}}$
18468.454	3		5413.160	$17224_{2}^{{}^{\circ}}$ – 22637_3_
18452.242	0		5417.916	$22855_{3}^{{}^{\circ}}$ – 28273_2_
18440.493	4		5421.368	11802_2_ – $17224_{2}^{{}^{\circ}}$
18427.858	0		5425.085	$27674_{2}^{{}^{\circ}}$ – 33099_3_
18396.840	0		5434.232	17073_1_ – $22508_{2}^{{}^{\circ}}$
18348.035	1		5448.687	19532_4_ – $24981_{3}^{{}^{\circ}}$
18339.435	0		5451.242	$25306_{2}^{{}^{\circ}}$ – 30758_2_
18335.284	2		5452.476	4961_4_ – $10414_{4}^{{}^{\circ}}$
18334.595	0		5452.681	$24561_{3}^{{}^{\circ}}$ – 30014_4_
18331.495	1		5453.603	$21890_{3}^{{}^{\circ}}$ – 27343_3_
18331.136	0		5453.710	$24381_{2}^{{}^{\circ}}$ – 29835_3_
18320.410	0		5456.903	17398_3_ – $22855_{3}^{{}^{\circ}}$
18301.976	1		5462.399	$26995_{3}^{{}^{\circ}}$ – 32458_4_
18293.550	1		5464.915	$22877_{0}^{{}^{\circ}}$ – 28342_5_
18286.901	1		5466.902	$20423_{1}^{{}^{\circ}}$ – 25890_2_
18281.471	1		5468.526	$24182_{2}^{{}^{\circ}}$ – 29650_2_
18280.488	0		5468.820	$18930_{3}^{{}^{\circ}}$ – 24399_3_
18274.319	0		5470.666	$22141_{3}^{{}^{\circ}}$ – 27612_3_
18225.855	1		5485.213	18431_3_ – $23916_{4}^{{}^{\circ}}$
18221.955	1		5486.387	$23655_{4}^{{}^{\circ}}$ – 29141_5_
18209.323	0		5490.193	$27061_{2}^{{}^{\circ}}$ – 32551_3_
18173.926	1		5500.886	$26036_{3}^{{}^{\circ}}$ – 31537_3_
18170.303	1		5501.983	21143_5_ – $26645_{0}^{{}^{\circ}}$
18169.715	1		5502.161	$7795_{4}^{{}^{\circ}}$ – 13297_4_
18166.638	1		5503.093	$22855_{3}^{{}^{\circ}}$ – 28358_3_
18161.579	2		5504.626	$21539_{4}^{{}^{\circ}}$ – 27044_3_
18156.664	0		5506.116	$14206_{4}^{{}^{\circ}}$ – 19713_3_
18135.791	1		5512.453	13297_4_ – $18809_{4}^{{}^{\circ}}$
18125.969	5		5515.440	6362_2_ – $11877_{1}^{{}^{\circ}}$
18103.118	2		5522.402	$24850_{6}^{{}^{\circ}}$ – 30372_6_
18087.083	2		5527.298	$24307_{2}^{{}^{\circ}}$ – 29835_3_
18072.340	4		5531.807	$17501_{0}^{{}^{\circ}}$ – 23032_4_
18070.540	1		5532.358	$23609_{0}^{{}^{\circ}}$ – 29141_5_
18023.692	1		5546.738	$25180_{7}^{{}^{\circ}}$ – 30726_7_
18007.579	2		5551.701	11802_2_ – $17354_{1}^{{}^{\circ}}$
18000.660	1		5553.835	$24202_{4}^{{}^{\circ}}$ – 29756_4_
17993.769	3		5555.962	$10414_{4}^{{}^{\circ}}$ – 15970_3_
17991.742	0		5556.588	$26645_{0}^{{}^{\circ}}$ – 32202_5_
17987.035	1		5558.042	$22669_{3}^{{}^{\circ}}$ – 28227_4_
17976.979	4		5561.151	17959_4_ – $23521_{3}^{{}^{\circ}}$
17969.554	0		5563.449	$24981_{3}^{{}^{\circ}}$ – 30544_2_
17964.920	6		5564.884	4961_4_ – $10526_{3}^{{}^{\circ}}$
17962.477	0		5565.641	19273_2_ – $24838_{1}^{{}^{\circ}}$
17960.037	0		5566.397	$26363_{2}^{{}^{\circ}}$ – 31929_3_
17955.860	2		5567.692	$20543_{0}^{{}^{\circ}}$ – 26111_1_
17893.451	0		5587.111	$13945_{3}^{{}^{\circ}}$ – 19532_4_
17887.982	3		5588.819	$20522_{2}^{{}^{\circ}}$ – 26111_1_
17886.677	2		5589.227	$18809_{4}^{{}^{\circ}}$ – 24399_3_
17886.181	0		5589.382	$24259_{4}^{{}^{\circ}}$ – 29849_4_
17883.051	2		5590.360	14226_0_ – $19817_{1}^{{}^{\circ}}$
17873.738	2		5593.273	$24421_{3}^{{}^{\circ}}$ – 30014_4_
17873.620	1		5593.310	$21902_{4}^{{}^{\circ}}$ – 27495_4_
17872.284	1		5593.728	19713_3_ – $25306_{2}^{{}^{\circ}}$
17854.649	2		5599.253	$24084_{6}^{{}^{\circ}}$ – 29684_5_
17849.848	1		5600.759	$20322_{0}^{{}^{\circ}}$ – 25923_4_
17838.977	1		5604.172	$8243_{2}^{{}^{\circ}}$ – 13847_2_
17834.907	1		5605.451	$21890_{3}^{{}^{\circ}}$ – 27495_4_
17827.385	1		5607.816	18574_1_ – $24182_{2}^{{}^{\circ}}$
17826.416	0		5608.121	18699_2_ – $24307_{2}^{{}^{\circ}}$
17825.875	5		5608.291	11802_2_ – $17411_{3}^{{}^{\circ}}$
17823.987	1		5608.885	19713_3_ – $25321_{3}^{{}^{\circ}}$
17788.214	2		5620.165	21645_4_ – $27266_{4}^{{}^{\circ}}$
17783.170	4		5621.759	$17411_{3}^{{}^{\circ}}$ – 23032_4_
17778.391	1		5623.270	11601_1_ – $17224_{2}^{{}^{\circ}}$
17763.368	0		5628.026	$26384_{4}^{{}^{\circ}}$ – 32012_4_
17757.108	2		5630.010	$24381_{2}^{{}^{\circ}}$ – 30011_3_
17752.753	1		5631.391	$21890_{3}^{{}^{\circ}}$ – 27521_4_
17744.898	5		5633.884	$17354_{1}^{{}^{\circ}}$ – 22988_2_
17742.618	0		5634.608	$22399_{0}^{{}^{\circ}}$ – 28034_5_
17720.358	2		5641.686	$22338_{3}^{{}^{\circ}}$ – 27980_3_
17717.673	0		5642.541	19713_3_ – $25355_{4}^{{}^{\circ}}$
17703.673	3		5647.003	21143_5_ – $26790_{4}^{{}^{\circ}}$
17688.744	2		5651.769	$21539_{4}^{{}^{\circ}}$ – 27191_5_
17683.810	3		5653.346	$15490_{0}^{{}^{\circ}}$ – 21143_5_
17683.237	0		5653.529	$22877_{1}^{{}^{\circ}}$ – 28531_2_
17677.481	3		5655.370	13847_2_ – $19503_{3}^{{}^{\circ}}$
17670.229	1		5657.691	$24850_{6}^{{}^{\circ}}$ – 30508_5_
17669.614	3		5657.888	$25306_{2}^{{}^{\circ}}$ – 30964_3_
17663.853	0		5659.733	$25877_{4}^{{}^{\circ}}$ – 31537_3_
17658.896	0		5661.322	$26113_{2}^{{}^{\circ}}$ – 31774_3_
17649.490	2		5664.339	$24880_{1}^{{}^{\circ}}$ – 30544_2_
17634.332	4		5669.208	13847_2_ – $19516_{2}^{{}^{\circ}}$
17633.725	0		5669.403	$23752_{2}^{{}^{\circ}}$ – 29422_1_
17627.118	0		5671.528	21645_4_ – $27317_{3}^{{}^{\circ}}$
17626.046	3		5671.873	15493_4_ – $21165_{3}^{{}^{\circ}}$
17623.094	6		5672.823	7502_3_ – $13175_{4}^{{}^{\circ}}$
17614.900	1		5675.462	$20214_{3}^{{}^{\circ}}$ – 25890_2_
17614.015	1		5675.747	$22855_{3}^{{}^{\circ}}$ – 28531_2_
17597.889	1		5680.948	$14032_{2}^{{}^{\circ}}$ – 19713_3_
17595.542	1		5681.706	18699_2_ – $24381_{2}^{{}^{\circ}}$
17584.517	7		5685.268	9804_5_ – $15490_{0}^{{}^{\circ}}$
17575.945	1		5688.041	$20423_{1}^{{}^{\circ}}$ – 26111_1_
17573.115	3		5688.957	$20566_{4}^{{}^{\circ}}$ – 26255_4_
17553.728	1		5695.240	17959_4_ – $23655_{4}^{{}^{\circ}}$
17552.671	4		5695.583	17398_3_ – $23093_{2}^{{}^{\circ}}$
17551.531	1		5695.953	$25575_{4}^{{}^{\circ}}$ – 31271_5_
17549.205	0		5696.708	$26096_{3}^{{}^{\circ}}$ – 31793_4_
17547.178	0		5697.366	$27266_{4}^{{}^{\circ}}$ – 32963_5_
17521.738	2		5705.638	$24838_{1}^{{}^{\circ}}$ – 30544_2_
17516.448	0		5707.361	$22855_{3}^{{}^{\circ}}$ – 28562_4_
17508.350	2		5710.001	$21902_{4}^{{}^{\circ}}$ – 27612_3_
17503.828	1		5711.476	17166_5_ – $22877_{0}^{{}^{\circ}}$
17492.573	2		5715.151	17398_3_ – $23113_{4}^{{}^{\circ}}$
17481.041	7		5718.921	$8243_{2}^{{}^{\circ}}$ – 13962_1_
17473.699	2		5721.324	13088_3_ – $18809_{4}^{{}^{\circ}}$
17441.114	0		5732.013	$23113_{4}^{{}^{\circ}}$ – 28845_4_
17437.704	1		5733.134	18574_1_ – $24307_{2}^{{}^{\circ}}$
17404.332	4		5744.127	14204_5_ – $19948_{4}^{{}^{\circ}}$
17401.230	1		5745.151	$26048_{4}^{{}^{\circ}}$ – 31793_4_
17388.612	0		5749.320	$25877_{4}^{{}^{\circ}}$ – 31626_5_
17385.370	4		5750.392	$25180_{7}^{{}^{\circ}}$ – 30930_6_
17381.909	7		5751.537	$16346_{4}^{{}^{\circ}}$ – 22098_4_
17380.610	5		5751.967	6362_2_ – $12114_{2}^{{}^{\circ}}$
17375.665	1		5753.604	11601_1_ – $17354_{1}^{{}^{\circ}}$
17372.868	1		5754.530	20054_2_ – $25809_{1}^{{}^{\circ}}$
17334.404	1		5767.299	$23916_{4}^{{}^{\circ}}$ – 29684_5_
17333.121	1		5767.726	$13945_{3}^{{}^{\circ}}$ – 19713_3_
17323.648	0		5770.880	18431_3_ – $24202_{4}^{{}^{\circ}}$
17311.753	1		5774.845	$24769_{3}^{{}^{\circ}}$ – 30544_2_
17307.662	7		5776.210	$17501_{0}^{{}^{\circ}}$ – 23277_5_
17290.627	1		5781.901	14204_5_ – $19986_{6}^{{}^{\circ}}$
17277.735	0		5786.215	$25355_{4}^{{}^{\circ}}$ – 31141_3_
17271.005	0		5788.470	$23093_{2}^{{}^{\circ}}$ – 28882_2_
17267.932	3		5789.500	19532_4_ – $25321_{3}^{{}^{\circ}}$
17264.172	3		5790.761	$17411_{3}^{{}^{\circ}}$ – 23201_3_
17261.632	0		5791.613	$21252_{2}^{{}^{\circ}}$ – 27044_3_
17227.152	0		5803.205	$25442_{3}^{{}^{\circ}}$ – 31245_2_
17225.670	3		5803.704	17073_1_ – $22877_{1}^{{}^{\circ}}$
17221.632	1		5805.065	15863_2_ – $21668_{1}^{{}^{\circ}}$
17217.782	2		5806.363	$21165_{3}^{{}^{\circ}}$ – 26971_4_
17216.726	0		5806.719	18574_1_ – $24381_{2}^{{}^{\circ}}$
17198.807	0		5812.769	$26645_{0}^{{}^{\circ}}$ – 32458_4_
17195.420	0		5813.914	$20566_{4}^{{}^{\circ}}$ – 26380_5_
17192.605	1		5814.866	$23603_{2}^{{}^{\circ}}$ – 29418_2_
17186.894	2		5816.798	$19588_{0}^{{}^{\circ}}$ – 25405_4_
17173.440	0		5821.355	$23113_{4}^{{}^{\circ}}$ – 28934_3_
17168.126	0		5823.157	19532_4_ – $25355_{4}^{{}^{\circ}}$
17165.765	2		5823.958	$22855_{3}^{{}^{\circ}}$ – 28679_2_
17163.009	1		5824.893	$24307_{2}^{{}^{\circ}}$ – 30132_2_
17153.591	2		5828.091	18431_3_ – $24259_{4}^{{}^{\circ}}$
17152.288	1		5828.534	$22399_{0}^{{}^{\circ}}$ – 28227_4_
17151.508	1		5828.799	$21738_{2}^{{}^{\circ}}$ – 27566_2_
17151.178	0		5828.911	$24182_{2}^{{}^{\circ}}$ – 30011_3_
17146.766	0		5830.411	$23015_{0}^{{}^{\circ}}$ – 28845_4_
17135.330	4		5834.302	8111_4_ – $13945_{3}^{{}^{\circ}}$
17134.167	4		5834.698	$23306_{6}^{{}^{\circ}}$ – 29141_5_
17133.189	1		5835.031	$25306_{2}^{{}^{\circ}}$ – 31141_3_
17124.268	1		5838.071	$15736_{1}^{{}^{\circ}}$ – 21575_2_
17121.318	3		5839.077	$22141_{3}^{{}^{\circ}}$ – 27980_3_
17120.840	0		5839.240	$27260_{3}^{{}^{\circ}}$ – 33099_3_
17120.083	1		5839.498	$23916_{4}^{{}^{\circ}}$ – 29756_4_
17113.542	2		5841.730	13088_3_ – $18930_{3}^{{}^{\circ}}$
17103.520	1		5845.153	16554_6_ – $22399_{0}^{{}^{\circ}}$
17088.335	2		5850.347	$22508_{2}^{{}^{\circ}}$ – 28358_3_
17085.097	0		5851.456	$24701_{0}^{{}^{\circ}}$ – 30552_4_
17078.798	3		5853.614	$16783_{4}^{{}^{\circ}}$ – 22637_3_
17075.750	3		5854.659	13962_1_ – $19817_{1}^{{}^{\circ}}$
17054.299	0		5862.023	18699_2_ – $24561_{3}^{{}^{\circ}}$
17028.599	1		5870.870	$22163_{4}^{{}^{\circ}}$ – 28034_5_
17026.334	1		5871.651	18549_2_ – $24421_{3}^{{}^{\circ}}$
17019.670	0		5873.950	$20922_{2}^{{}^{\circ}}$ – 26796_3_
17018.885	2		5874.221	$21738_{2}^{{}^{\circ}}$ – 27612_3_
17014.114	1		5875.868	$19039_{2}^{{}^{\circ}}$ – 24915_3_
17013.556	0		5876.061	18431_3_ – $24307_{2}^{{}^{\circ}}$
17012.360	0		5876.474	$24850_{6}^{{}^{\circ}}$ – 30726_7_
17008.562	2		5877.786	$24880_{1}^{{}^{\circ}}$ – 30758_2_
17005.290	2		5878.917	$27084_{6}^{{}^{\circ}}$ – 32963_5_
17004.697	3		5879.122	$21165_{3}^{{}^{\circ}}$ – 27044_3_
16997.972	0		5881.448	$24769_{3}^{{}^{\circ}}$ – 30651_3_
16989.777	1		5884.285	$25442_{3}^{{}^{\circ}}$ – 31326_4_
16976.362	0		5888.935	$22338_{3}^{{}^{\circ}}$ – 28227_4_
16964.222	0		5893.149	$26036_{3}^{{}^{\circ}}$ – 31929_3_
16960.892	1		5894.306	$21077_{0}^{{}^{\circ}}$ – 26971_4_
16952.178	0		5897.336	$23521_{3}^{{}^{\circ}}$ – 29418_2_
16949.485	0		5898.273	$23752_{2}^{{}^{\circ}}$ – 29650_2_
16945.150	0		5899.782	$26651_{2}^{{}^{\circ}}$ – 32551_3_
16938.730	3		5902.018	$19503_{3}^{{}^{\circ}}$ – 25405_4_
16918.744	0		5908.990	$26384_{4}^{{}^{\circ}}$ – 32293_5_
16915.692	0		5910.056	$25355_{4}^{{}^{\circ}}$ – 31265_3_
16915.097	1		5910.264	19532_4_ – $25442_{3}^{{}^{\circ}}$
16900.345	1		5915.423	$25355_{4}^{{}^{\circ}}$ – 31271_5_
16887.173	1		5920.037	15970_3_ – $21890_{3}^{{}^{\circ}}$
16883.454	0		5921.341	$18069_{3}^{{}^{\circ}}$ – 23990_2_
16879.312	2		5922.794	$17847_{2}^{{}^{\circ}}$ – 23769_1_
16875.970	0		5923.967	$25321_{3}^{{}^{\circ}}$ – 31245_2_
16852.625	2		5932.173	15970_3_ – $21902_{4}^{{}^{\circ}}$
16849.382	0		5933.315	$20322_{0}^{{}^{\circ}}$ – 26255_4_
16846.815	0		5934.219	$22338_{3}^{{}^{\circ}}$ – 28273_2_
16846.017	0		5934.500	$25336_{6}^{{}^{\circ}}$ – 31271_5_
16840.767	0		5936.350	$25690_{0}^{{}^{\circ}}$ – 31626_5_
16832.896	0		5939.126	$25306_{2}^{{}^{\circ}}$ – 31245_2_
16820.947	1		5943.345	$23609_{0}^{{}^{\circ}}$ – 29552_6_
16811.787	0		5946.583	$26790_{4}^{{}^{\circ}}$ – 32737_5_
16801.531	0		5950.213	$24182_{2}^{{}^{\circ}}$ – 30132_2_
16800.478	1		5950.586	13088_3_ – $19039_{2}^{{}^{\circ}}$
16785.229	2		5955.992	$21539_{4}^{{}^{\circ}}$ – 27495_4_
16785.046	2		5956.057	$15618_{3}^{{}^{\circ}}$ – 21575_2_
16782.389	1		5957.000	17959_4_ – $23916_{4}^{{}^{\circ}}$
16768.550	6		5961.916	12847_3_ – $18809_{4}^{{}^{\circ}}$
16761.533	0		5964.412	$26048_{4}^{{}^{\circ}}$ – 32012_4_
16748.433	5		5969.077	$11197_{0}^{{}^{\circ}}$ – 17166_5_
16747.499	2		5969.410	13847_2_ – $19817_{1}^{{}^{\circ}}$
16741.938	0		5971.393	$25355_{4}^{{}^{\circ}}$ – 31326_4_
16730.028	3		5975.644	17073_1_ – $23049_{1}^{{}^{\circ}}$
16724.318	1		5977.684	$17224_{2}^{{}^{\circ}}$ – 23201_3_
16720.218	0		5979.150	$24307_{2}^{{}^{\circ}}$ – 30286_1_
16712.444	3		5981.931	$21539_{4}^{{}^{\circ}}$ – 27521_4_
16707.945	0		5983.542	$24981_{3}^{{}^{\circ}}$ – 30964_3_
16704.639	3		5984.726	$18930_{3}^{{}^{\circ}}$ – 24915_3_
16694.697	0		5988.290	$24769_{3}^{{}^{\circ}}$ – 30758_2_
16691.676	1		5989.374	18431_3_ – $24421_{3}^{{}^{\circ}}$
16688.906	1		5990.368	19713_3_ – $25703_{2}^{{}^{\circ}}$
16687.560	0		5990.851	$24561_{3}^{{}^{\circ}}$ – 30552_4_
16686.678	1		5991.168	$26790_{4}^{{}^{\circ}}$ – 32781_4_
16624.607	2		6013.537	$18614_{1}^{{}^{\circ}}$ – 24627_1_
16606.290	1		6020.170	17073_1_ – $23093_{2}^{{}^{\circ}}$
16604.436	1		6020.842	$18011_{0}^{{}^{\circ}}$ – 24032_4_
16599.350	3		6022.687	$14032_{2}^{{}^{\circ}}$ – 20054_2_
16594.999	0		6024.266	$22248_{2}^{{}^{\circ}}$ – 28273_2_
16589.555	1		6026.243	15863_2_ – $21890_{3}^{{}^{\circ}}$
16587.815	3		6026.875	$15618_{3}^{{}^{\circ}}$ – 21645_4_
16587.044	0		6027.155	$22855_{3}^{{}^{\circ}}$ – 28882_2_
16569.667	0		6033.476	19273_2_ – $25306_{2}^{{}^{\circ}}$
16540.424	5		6044.143	11802_2_ – $17847_{2}^{{}^{\circ}}$
16538.399	3		6044.883	16351_0_ – $22396_{1}^{{}^{\circ}}$
16534.332	2		6046.370	15493_4_ – $21539_{4}^{{}^{\circ}}$
16531.417	1		6047.436	$23603_{2}^{{}^{\circ}}$ – 29650_2_
16521.658	1		6051.008	$24838_{1}^{{}^{\circ}}$ – 30889_1_
16503.791	4		6057.559	$18614_{1}^{{}^{\circ}}$ – 24671_2_
16501.244	0		6058.494	20054_2_ – $26113_{2}^{{}^{\circ}}$
16461.254	1		6073.212	$19817_{1}^{{}^{\circ}}$ – 25890_2_
16452.932	0		6076.284	21594_3_ – $27670_{3}^{{}^{\circ}}$
16447.180	0		6078.409	$21902_{4}^{{}^{\circ}}$ – 27980_3_
16414.399	1		6090.548	$21890_{3}^{{}^{\circ}}$ – 27980_3_
16412.823	3		6091.133	$21252_{2}^{{}^{\circ}}$ – 27343_3_
16403.906	0		6094.444	$23916_{4}^{{}^{\circ}}$ – 30011_3_
16394.343	1		6097.999	$24274_{0}^{{}^{\circ}}$ – 30372_6_
16390.870	0		6099.291	21575_2_ – $27674_{2}^{{}^{\circ}}$
16375.188	1		6105.132	$18809_{4}^{{}^{\circ}}$ – 24915_3_
16363.631	2		6109.444	$22248_{2}^{{}^{\circ}}$ – 28358_3_
16340.749	2		6117.999	14204_5_ – $20322_{0}^{{}^{\circ}}$
16329.816	2		6122.095	$20922_{2}^{{}^{\circ}}$ – 27044_3_
16326.067	2		6123.501	$24421_{3}^{{}^{\circ}}$ – 30544_2_
16310.703	3		6129.269	$20867_{7}^{{}^{\circ}}$ – 26997_6_
16308.851	2		6129.965	18431_3_ – $24561_{3}^{{}^{\circ}}$
16304.920	0		6131.443	$24421_{3}^{{}^{\circ}}$ – 30552_4_
16304.149	3		6131.733	$21902_{4}^{{}^{\circ}}$ – 28034_5_
16297.533	0		6134.222	$22396_{1}^{{}^{\circ}}$ – 28531_2_
16284.058	1		6139.298	18699_2_ – $24838_{1}^{{}^{\circ}}$
16280.282	3		6140.722	17166_5_ – $23306_{6}^{{}^{\circ}}$
16273.373	0		6143.329	$17847_{2}^{{}^{\circ}}$ – 23990_2_
16270.823	0		6144.292	$26651_{2}^{{}^{\circ}}$ – 32796_3_
16263.051	1		6147.228	$23609_{0}^{{}^{\circ}}$ – 29756_4_
16240.450	2		6155.783	$15490_{0}^{{}^{\circ}}$ – 21645_4_
16238.115	3		6156.668	$11241_{3}^{{}^{\circ}}$ – 17398_3_
16234.453	0		6158.057	19532_4_ – $25690_{0}^{{}^{\circ}}$
16220.740	3		6163.263	$22399_{0}^{{}^{\circ}}$ – 28562_4_
16217.564	0		6164.470	19713_3_ – $25877_{4}^{{}^{\circ}}$
16211.744	0		6166.683	$26384_{4}^{{}^{\circ}}$ – 32551_3_
16204.604	1		6169.400	19273_2_ – $25442_{3}^{{}^{\circ}}$
16195.248	1		6172.964	$26790_{4}^{{}^{\circ}}$ – 32963_3_
16188.147	0		6175.672	$22669_{3}^{{}^{\circ}}$ – 28845_4_
16180.368	0		6178.641	$21165_{3}^{{}^{\circ}}$ – 27343_3_
16178.472	2		6179.365	$22163_{4}^{{}^{\circ}}$ – 28342_5_
16172.466	1		6181.660	$25355_{4}^{{}^{\circ}}$ – 31537_3_
16147.600	3		6191.179	12847_3_ – $19039_{2}^{{}^{\circ}}$
16145.326	1		6192.051	$22338_{3}^{{}^{\circ}}$ – 28531_2_
16142.758	2		6193.036	15970_3_ – $22163_{4}^{{}^{\circ}}$
16140.194	0		6194.020	$23655_{4}^{{}^{\circ}}$ – 29849_4_
16137.810	0		6194.935	$24769_{3}^{{}^{\circ}}$ – 30964_3_
16125.805	0		6199.547	$25575_{4}^{{}^{\circ}}$ – 31774_3_
16109.798	5		6205.707	13297_4_ – $19503_{3}^{{}^{\circ}}$
16100.958	0		6209.114	$23752_{2}^{{}^{\circ}}$ – 29961_1_
16084.899	0		6215.313	$25321_{3}^{{}^{\circ}}$ – 31537_3_
16081.086	1		6216.787	$22141_{3}^{{}^{\circ}}$ – 28358_3_
16071.988	1		6220.306	18549_2_ – $24769_{3}^{{}^{\circ}}$
16070.913	0		6220.722	$23741_{1}^{{}^{\circ}}$ – 29961_1_
16068.852	1		6221.520	19532_4_ – $25753_{0}^{{}^{\circ}}$
16056.201	0		6226.422	$22877_{0}^{{}^{\circ}}$ – 29104_4_
16048.543	1		6229.393	$24701_{0}^{{}^{\circ}}$ – 30930_6_
16045.762	0		6230.473	$25306_{2}^{{}^{\circ}}$ – 31537_3_
16038.518	3		6233.287	$24274_{0}^{{}^{\circ}}$ – 30508_5_
16033.162	2		6235.369	4961_4_ – $11197_{0}^{{}^{\circ}}$
16014.426	2		6242.664	17959_4_ – $24202_{4}^{{}^{\circ}}$
16008.311	3		6245.049	$18382_{0}^{{}^{\circ}}$ – 24627_1_
16006.680	0		6245.685	$23306_{6}^{{}^{\circ}}$ – 29552_6_
16005.763	4		6246.043	11601_1_ – $17847_{2}^{{}^{\circ}}$
15997.841	2		6249.136	$16783_{2}^{{}^{\circ}}$ – 23032_4_
15978.393	2		6256.742	17398_3_ – $23655_{4}^{{}^{\circ}}$
15973.502	1		6258.658	$23752_{2}^{{}^{\circ}}$ – 30011_3_
15972.593	0		6259.014	$25753_{0}^{{}^{\circ}}$ – 32012_4_
15960.024	2		6263.943	$22877_{0}^{{}^{\circ}}$ – 29141_5_
15954.449	4		6266.132	11802_2_ – $18069_{3}^{{}^{\circ}}$
15941.439	0		6271.246	$25355_{4}^{{}^{\circ}}$ – 31626_5_
15936.069	3		6273.359	$20522_{2}^{{}^{\circ}}$ – 26796_3_
15925.011	1		6277.715	15863_2_ – $22141_{3}^{{}^{\circ}}$
15924.829	0		6277.787	$24274_{0}^{{}^{\circ}}$ – 30552_4_
15922.130	4		6278.851	15970_3_ – $22248_{2}^{{}^{\circ}}$
15919.042	1		6280.069	4961_4 _ – $11241_{3}^{{}^{\circ}}$
15915.448	1		6281.487	18699_2_ – $24981_{3}^{{}^{\circ}}$
15913.906	0		6282.096	$22248_{2}^{{}^{\circ}}$ – 28531_2_
15899.553	3		6287.767	$24084_{6}^{{}^{\circ}}$ – 30372_6_
15895.139	1		6289.513	18549_2_ – $24838_{1}^{{}^{\circ}}$
15893.090	1		6290.324	$25336_{6}^{{}^{\circ}}$ – 31626_5_
15891.854	3		6290.813	$16346_{4}^{{}^{\circ}}$ – 22637_3_
15891.564	4		6290.928	13297_4_ – $19588_{0}^{{}^{\circ}}$
15887.023	1		6292.726	$24259_{4}^{{}^{\circ}}$ – 30552_4_
15868.987	2		6299.878	17959_4_ – $24259_{4}^{{}^{\circ}}$
15861.758	1		6302.749	21645_4_ – $27948_{4}^{{}^{\circ}}$
15854.989	1		6305.440	$24202_{4}^{{}^{\circ}}$ – 30508_5_
15847.733	3		6308.327	20054_2_ – $26363_{2}^{{}^{\circ}}$
15833.507	0		6313.995	$23521_{3}^{{}^{\circ}}$ – 29835_3_
15832.882	0		6314.244	$21252_{2}^{{}^{\circ}}$ – 27566_2_
15831.752	7		6314.695	5563_1_ – $11877_{1}^{{}^{\circ}}$
15825.176	2		6317.319	$12114_{2}^{{}^{\circ}}$ – 18431_3_
15810.170	1		6323.315	19713_3_ – $26036_{3}^{{}^{\circ}}$
15807.445	0		6324.405	$23093_{2}^{{}^{\circ}}$ – 29418_2_
15804.308	1		6325.660	$21902_{4}^{{}^{\circ}}$ – 28227_4_
15793.353	0		6330.048	$18069_{3}^{{}^{\circ}}$ – 24399_3_
15792.250	0		6330.490	$21165_{3}^{{}^{\circ}}$ – 27495_4_
15791.447	1		6330.812	18549_2_ – $24880_{1}^{{}^{\circ}}$
15788.721	0		6331.905	$25442_{3}^{{}^{\circ}}$ – 31774_3_
15781.849	1		6334.662	$19588_{0}^{{}^{\circ}}$ – 25923_4_
15754.904	1		6345.496	$18053_{4}^{{}^{\circ}}$ – 24399_3_
15743.881	2		6349.939	$24202_{4}^{{}^{\circ}}$ – 30552_4_
15733.987	0		6353.932	21594_3_ – $27948_{4}^{{}^{\circ}}$
15732.702	0		6354.451	$25575_{4}^{{}^{\circ}}$ – 31929_3_
15728.365	0		6356.203	$23655_{4}^{{}^{\circ}}$ – 30011_3_
15727.808	0		6356.428	$21165_{3}^{{}^{\circ}}$ – 27521_4_
15720.977	1		6359.190	$23655_{4}^{{}^{\circ}}$ – 30014_4_
15719.798	1		6359.667	$21252_{2}^{{}^{\circ}}$ – 27612_3_
15713.149	4		6362.358	14204_5_ – $20566_{4}^{{}^{\circ}}$
15702.559	1		6366.649	8800_4_ – $15166_{3}^{{}^{\circ}}$
15701.306	3		6367.157	13847_2_ – $20214_{3}^{{}^{\circ}}$
15697.019	1		6368.896	15970_3_ – $22338_{3}^{{}^{\circ}}$
15696.943	1		6368.927	$23049_{1}^{{}^{\circ}}$ – 29418_2_
15676.166	3		6377.368	$23306_{6}^{{}^{\circ}}$ – 29684_5_
15662.128	0		6383.084	$21890_{3}^{{}^{\circ}}$ – 28273_2_
15641.777	0		6391.389	$24259_{4}^{{}^{\circ}}$ – 30651_3_
15641.344	3		6391.566	$23741_{1}^{{}^{\circ}}$ – 30132_2_
15634.482	2		6394.371	$19986_{6}^{{}^{\circ}}$ – 26380_5_
15628.272	3		6396.912	15493_4_ – $21890_{3}^{{}^{\circ}}$
15621.888	0		6399.526	$22163_{4}^{{}^{\circ}}$ – 28562_4_
15620.165	2		6400.232	19713_3_ – $26113_{2}^{{}^{\circ}}$
15616.447	1		6401.756	$21165_{3}^{{}^{\circ}}$ – 27566_2_
15608.939	2		6404.835	$20566_{4}^{{}^{\circ}}$ – 26971_4_
15608.145	1		6405.161	$23609_{0}^{{}^{\circ}}$ – 30014_4_
15602.299	0		6407.561	17073_1_ – $23481_{1}^{{}^{\circ}}$
15598.808	4		6408.995	$7795_{4}^{{}^{\circ}}$ – 14204_5_
15598.676	4		6409.049	15493_4_ – $21902_{4}^{{}^{\circ}}$
15585.229	3		6414.579	13088_3_ – $19503_{3}^{{}^{\circ}}$
15583.635	2		6415.235	$17354_{1}^{{}^{\circ}}$ – 23769_1_
15576.584	6		6418.139	$16783_{4}^{{}^{\circ}}$ – 23201_3_
15575.870	1		6418.433	$21077_{0}^{{}^{\circ}}$ – 27495_4_
15574.463	1		6419.013	$25355_{4}^{{}^{\circ}}$ – 31774_3_
15572.352	2		6419.883	$19503_{3}^{{}^{\circ}}$ – 25923_4_
15572.117	4		6419.980	$16217_{2}^{{}^{\circ}}$ – 22637_3_
15569.510	3		6421.055	$22141_{3}^{{}^{\circ}}$ – 28562_4_
15568.155	0		6421.614	$20922_{2}^{{}^{\circ}}$ – 27343_3_
15565.585	0		6422.674	$26645_{0}^{{}^{\circ}}$ – 33068_6_
15553.233	2		6427.775	$15166_{3}^{{}^{\circ}}$ – 21594_3_
15551.679	4		6428.417	13088_3_ – $19516_{2}^{{}^{\circ}}$
15547.561	1		6430.120	19273_2_ – $25703_{2}^{{}^{\circ}}$
15542.668	2		6432.144	$19948_{4}^{{}^{\circ}}$ – 26380_5_
15537.904	0		6434.116	$22669_{3}^{{}^{\circ}}$ – 29104_4_
15523.156	1		6440.229	$21902_{4}^{{}^{\circ}}$ – 28342_5_
15516.436	6		6443.018	7502_3_ – $13945_{3}^{{}^{\circ}}$
15513.174	1		6444.373	$21077_{0}^{{}^{\circ}}$ – 27521_4_
15508.866	1		6446.163	$22399_{0}^{{}^{\circ}}$ – 28845_4_
15505.455	0		6447.581	$26651_{2}^{{}^{\circ}}$ – 33099_3_
15503.005	0		6448.600	$24202_{4}^{{}^{\circ}}$ – 30651_3_
15499.015	1		6450.260	$24307_{2}^{{}^{\circ}}$ – 30758_2_
15475.060	1		6460.245	$12114_{2}^{{}^{\circ}}$ – 18574_1_
15473.472	1		6460.908	16554_6_ – $23015_{0}^{{}^{\circ}}$
15473.326	1		6460.969	13962_1_ – $20423_{1}^{{}^{\circ}}$
15472.864	3		6461.162	17959_4_ – $24421_{3}^{{}^{\circ}}$
15456.917	1		6467.828	$25306_{2}^{{}^{\circ}}$ – 31774_3_
15455.882	0		6468.261	$21890_{3}^{{}^{\circ}}$ – 28358_3_
15454.759	0		6468.731	$24182_{2}^{{}^{\circ}}$ – 30651_3_
15440.113	4		6474.867	$18930_{3}^{{}^{\circ}}$ – 25405_4_
15439.546	2		6475.105	15863_2_ – $22338_{3}^{{}^{\circ}}$
15429.783	7		6479.202	$15618_{3}^{{}^{\circ}}$ – 22098_4_
15395.715	2		6493.539	$16783_{4}^{{}^{\circ}}$ – 23277_5_
15390.079	0		6495.917	$24769_{3}^{{}^{\circ}}$ – 31265_3_
15373.158	0		6503.067	$26048_{4}^{{}^{\circ}}$ – 32551_3_
15364.893	1		6506.565	$22338_{3}^{{}^{\circ}}$ – 28845_4_
15355.694	5		6510.463	14226_0_ – $20737_{1}^{{}^{\circ}}$
15342.358	1		6516.122	19532_4_ – $26048_{4}^{{}^{\circ}}$
15336.766	0		6518.498	17398_3_ – $23916_{4}^{{}^{\circ}}$
15320.133	0		6525.575	16351_0_ – $22877_{1}^{{}^{\circ}}$
15307.300	6		6531.046	$17501_{0}^{{}^{\circ}}$ – 24032_4_
15297.656	0		6535.163	$21738_{2}^{{}^{\circ}}$ – 28273_2_
15295.651	0		6536.020	19273_2_ – $25809_{1}^{{}^{\circ}}$
15292.511	1		6537.362	$23015_{0}^{{}^{\circ}}$ – 29552_6_
15291.203	2		6537.921	$13175_{4}^{{}^{\circ}}$ – 19713_3_
15284.311	0		6540.869	$22877_{1}^{{}^{\circ}}$ – 29418_2_
15282.038	2		6541.842	9804_5_ – $16346_{4}^{{}^{\circ}}$
15278.264	1		6543.458	$22338_{3}^{{}^{\circ}}$ – 28882_2_
15273.332	2		6545.571	$17224_{2}^{{}^{\circ}}$ – 23769_1_
15265.258	0		6549.033	$24880_{1}^{{}^{\circ}}$ – 31429_1_
15264.344	2		6549.425	18431_3_ – $24981_{3}^{{}^{\circ}}$
15260.157	2		6551.222	5563_1_ – $12114_{2}^{{}^{\circ}}$
15246.115	1		6557.256	$24769_{3}^{{}^{\circ}}$ – 31326_4_
15239.289	4		6560.193	13962_1_ – $20522_{2}^{{}^{\circ}}$
15215.038	0		6570.649	$23113_{4}^{{}^{\circ}}$ – 29684_5_
15203.638	0		6575.576	$24182_{2}^{{}^{\circ}}$ – 30758_2_
15195.303	0		6579.183	$17411_{3}^{{}^{\circ}}$ – 23990_2_
15190.777	3		6581.143	$20214_{3}^{{}^{\circ}}$ – 26796_3_
15190.369	2		6581.320	13962_1_ – $20543_{0}^{{}^{\circ}}$
15167.182	0		6591.381	$26508_{3}^{{}^{\circ}}$ – 33099_3_
15158.232	1		6595.273	$18809_{4}^{{}^{\circ}}$ – 25405_4_
15157.340	0		6595.661	$18069_{3}^{{}^{\circ}}$ – 24664_3_
15156.775	1		6595.907	$22338_{3}^{{}^{\circ}}$ – 28934_3_
15143.355	2		6601.752	17959_4_ – $24561_{3}^{{}^{\circ}}$
15137.607	0		6604.259	$21668_{1}^{{}^{\circ}}$ – 28273_2_
15112.474	2		6615.242	$10783_{2}^{{}^{\circ}}$ – 17398_3_
15100.835	2		6620.341	$21738_{2}^{{}^{\circ}}$ – 28358_3_
15099.336	4		6620.998	$17411_{3}^{{}^{\circ}}$ – 24032_4_
15095.376	1		6622.735	$25306_{2}^{{}^{\circ}}$ – 31929_3_
15090.817	2		6624.736	$20566_{4}^{{}^{\circ}}$ – 27191_5_
15065.723	1		6635.770	$17354_{1}^{{}^{\circ}}$ – 23990_2_
15051.950	2		6641.842	$24084_{6}^{{}^{\circ}}$ – 30726_7_
15049.671	2		6642.848	$23113_{4}^{{}^{\circ}}$ – 29756_5_
15045.413	3		6644.728	$20922_{2}^{{}^{\circ}}$ – 27566_2_
15037.137	0		6648.385	15493_4_ – $22141_{3}^{{}^{\circ}}$
15035.312	0		6649.192	$20322_{0}^{{}^{\circ}}$ – 26971_4_
15031.317	1		6650.959	13297_4_ – $19948_{4}^{{}^{\circ}}$
15021.804	3		6655.171	12847_3_ – $19503_{3}^{{}^{\circ}}$
15017.893	0		6656.904	$24307_{2}^{{}^{\circ}}$ – 30964_3_
15010.031	3		6660.391	$21902_{4}^{{}^{\circ}}$ – 28562_4_
15007.406	0		6661.556	$14481_{6}^{{}^{\circ}}$ – 21143_5_
14994.654	3		6667.221	$19588_{0}^{{}^{\circ}}$ – 26255_4_
14994.569	2		6667.259	17073_1_ – $23741_{1}^{{}^{\circ}}$
14990.632	1		6669.010	12847_3_ – $19516_{2}^{{}^{\circ}}$
14988.603	4		6669.913	15493_4_ – $22163_{4}^{{}^{\circ}}$
14984.882	4		6671.569	$11877_{1}^{{}^{\circ}}$ – 18549_2_
14982.726	1		6672.529	$21890_{3}^{{}^{\circ}}$ – 28562_4_
14980.034	0		6673.728	$25321_{3}^{{}^{\circ}}$ – 31995_4_
14977.332	3		6674.932	$22877_{0}^{{}^{\circ}}$ – 29552_6_
14968.497	2		6678.872	17073_1_ – $23752_{2}^{{}^{\circ}}$
14960.529	0		6682.429	$22163_{4}^{{}^{\circ}}$ – 28845_4_
14956.476	2		6684.240	$24561_{3}^{{}^{\circ}}$ – 31245_2_
14955.641	1		6684.613	$26096_{3}^{{}^{\circ}}$ – 32781_4_
14951.792	1		6686.334	$16346_{4}^{{}^{\circ}}$ – 23032_4_
14947.310	2		6688.339	$21539_{4}^{{}^{\circ}}$ – 28227_4_
14947.021	1		6688.468	$26048_{4}^{{}^{\circ}}$ – 32737_5_
14943.987	6		6689.826	8800_4_ – $15490_{0}^{{}^{\circ}}$
14928.485	4		6696.773	$11877_{1}^{{}^{\circ}}$ – 18574_1_
14921.758	0		6699.792	15970_3_ – $22669_{3}^{{}^{\circ}}$
14911.028	2		6704.613	$22399_{0}^{{}^{\circ}}$ – 29104_4_
14880.943	0		6718.168	$11241_{3}^{{}^{\circ}}$ – 17959_4_
14875.259	1		6720.735	$24421_{3}^{{}^{\circ}}$ – 31141_3_
14873.575	1		6721.496	$23113_{4}^{{}^{\circ}}$ – 29835_3_
14850.034	0		6732.151	18574_1_ – $25306_{2}^{{}^{\circ}}$
14842.404	0		6735.612	$23113_{4}^{{}^{\circ}}$ – 29849_4_
14830.215	3		6741.148	17959_4_ – $24701_{0}^{{}^{\circ}}$
14830.001	1		6741.245	$23015_{0}^{{}^{\circ}}$ – 29756_4_
14809.031	2		6750.791	17166_5_ – $23916_{4}^{{}^{\circ}}$
14806.456	5		6751.965	7280_2_ – $14032_{2}^{{}^{\circ}}$
14805.415	3		6752.440	$19503_{3}^{{}^{\circ}}$ – 26255_4_
14805.099	1		6752.584	16554_6_ – $23306_{6}^{{}^{\circ}}$
14796.452	0		6756.530	$20214_{3}^{{}^{\circ}}$ – 26971_4_
14789.356	1		6759.772	$26036_{3}^{{}^{\circ}}$ – 32796_3_
14784.758	1		6761.874	13962_1_ – $20724_{0}^{{}^{\circ}}$
14781.114	0		6763.541	$23609_{0}^{{}^{\circ}}$ – 30372_6_
14777.907	1		6765.009	$22338_{3}^{{}^{\circ}}$ – 29104_4_
14777.230	1		6765.319	$24561_{3}^{{}^{\circ}}$ – 31326_4_
14772.419	0		6767.522	$24769_{3}^{{}^{\circ}}$ – 31537_3_
14772.031	3		6767.700	$25690_{0}^{{}^{\circ}}$ – 32458_4_
14764.746	2		6771.039	$16217_{2}^{{}^{\circ}}$ – 22988_2_
14756.630	3		6774.763	13962_1_ – $20737_{1}^{{}^{\circ}}$
14751.515	0		6777.112	$20566_{4}^{{}^{\circ}}$ – 27343_3_
14741.329	5		6781.795	11601_1_ – $18382_{0}^{{}^{\circ}}$
14740.403	0		6782.221	$24182_{2}^{{}^{\circ}}$ – 30964_3_
14736.470	0		6784.031	17398_3_ – $24182_{2}^{{}^{\circ}}$
14719.449	1		6791.876	$23752_{2}^{{}^{\circ}}$ – 30544_2_
14718.799	5		6792.176	$19588_{0}^{{}^{\circ}}$ – 26380_5_
14716.370	2		6793.297	$22141_{3}^{{}^{\circ}}$ – 28934_3_
14695.579	3		6802.908	$21539_{4}^{{}^{\circ}}$ – 28342_5_
14690.666	0		6805.183	21143_5_ – $27948_{4}^{{}^{\circ}}$
14689.932	0		6805.523	$23481_{1}^{{}^{\circ}}$ – 30286_1_
14688.909	0		6805.997	15863_2_ – $22669_{3}^{{}^{\circ}}$
14687.576	0		6806.615	$22877_{0}^{{}^{\circ}}$ – 29684_5_
14680.667	0		6809.818	17959_4_ – $24769_{3}^{{}^{\circ}}$
14677.249	5		6811.404	11802_2_ – $18614_{1}^{{}^{\circ}}$
14661.334	0		6818.798	$21539_{4}^{{}^{\circ}}$ – 28358_3_
14654.914	7		6821.785	$11877_{1}^{{}^{\circ}}$ – 18699_2_
14651.482	3		6823.383	20054_2_ – $26878_{3}^{{}^{\circ}}$
14644.393	1		6826.686	18699_2_ – $25526_{1}^{{}^{\circ}}$
14638.811	0		6829.289	$20214_{3}^{{}^{\circ}}$ – 27044_3_
14638.217	1		6829.566	$20737_{1}^{{}^{\circ}}$ – 27566_2_
14628.703	2		6834.008	$23015_{0}^{{}^{\circ}}$ – 29849_4_
14618.976	7		6838.555	3687_2_ – $10526_{3}^{{}^{\circ}}$
14615.924	1		6839.983	19273_2_ – $26113_{2}^{{}^{\circ}}$
14604.155	2		6845.495	$24084_{6}^{{}^{\circ}}$ – 30930_6_
14603.558	3		6845.775	15493_4_ – $22338_{3}^{{}^{\circ}}$
14603.174	0		6845.955	$18069_{3}^{{}^{\circ}}$ – 24915_3_
14599.499	1		6847.678	$19948_{4}^{{}^{\circ}}$ – 26796_3_
14591.907	1		6851.241	$19039_{2}^{{}^{\circ}}$ – 25890_2_
14573.635	2		6859.831	13088_3_ – $19948_{4}^{{}^{\circ}}$
14570.349'	3		6861.378	17398_3_ – $24259_{4}^{{}^{\circ}}$
14563.603	0		6864.556	$24381_{2}^{{}^{\circ}}$ – 31245_2_
14553.980	1		6869.095	$20322_{0}^{{}^{\circ}}$ – 27191_5_
14548.134	2		6871.855	$10526_{3}^{{}^{\circ}}$ – 17398_3_
14545.954	2		6872.885	14204_5_ – $21077_{0}^{{}^{\circ}}$
14541.314	1		6875.078	18431_3_ – $25306_{2}^{{}^{\circ}}$
14533.419	4		6878.813	$22877_{0}^{{}^{\circ}}$ – 29756_4_
14519.935	2		6885.201	15970_3_ – $22855_{3}^{{}^{\circ}}$
14510.851	0		6889.511	13847_2_ – $20737_{1}^{{}^{\circ}}$
14502.932	1		6893.273	18549_2_ – $25442_{3}^{{}^{\circ}}$
14493.424	2		6897.795	$23113_{4}^{{}^{\circ}}$ – 30011_3_
14491.248	1		6898.831	$23609_{0}^{{}^{\circ}}$ – 30508_5_
14476.391	0		6905.911	$24421_{3}^{{}^{\circ}}$ – 31326_4_
14475.836	4		6906.176	15493_4_ – $22399_{0}^{{}^{\circ}}$
14469.188	2		6909.349	17398_3_ – $24307_{2}^{{}^{\circ}}$
14467.109	0		6910.342	$22508_{2}^{{}^{\circ}}$ – 29418_2_
14452.427	0		6917.362	$23093_{2}^{{}^{\circ}}$ – 30011_3_
14452.147	2		6917.496	13297_4_ – $20214_{3}^{{}^{\circ}}$
14451.758	4		6917.682	3865_1_ – $10783_{2}^{{}^{\circ}}$
14449.799	1		6918.620	$25877_{4}^{{}^{\circ}}$ – 32796_3_
14449.350	2		6918.835	17166_5_ – $24084_{6}^{{}^{\circ}}$
14438.799	3		6923.891	18431_3_ – $25355_{4}^{{}^{\circ}}$
14424.537	7		6930.737	$16346_{4}^{{}^{\circ}}$ – 23277_5_
14423.392	2		6931.287	$15166_{3}^{{}^{\circ}}$ – 22098_4_
14412.536	4		6936.508	$14206_{4}^{{}^{\circ}}$ – 21143_5_
14407.785	1		6938.795	19713_3_ – $26651_{2}^{{}^{\circ}}$
14403.474	1		6940.872	$22163_{4}^{{}^{\circ}}$ – 29104_4_
14403.132	0		6941.037	$23603_{2}^{{}^{\circ}}$ – 30544_2_
14398.454	2		6943.292	$21902_{4}^{{}^{\circ}}$ – 28845_4_
14374.424	2		6954.899	$20566_{4}^{{}^{\circ}}$ – 27521_4_
14373.329	1		6955.429	$21890_{3}^{{}^{\circ}}$ – 28845_4_
14370.393	0		6956.850	$21077_{0}^{{}^{\circ}}$ – 28034_5_
14364.711	2		6959.602	13962_1_ – $20922_{2}^{{}^{\circ}}$
14363.687	2		6960.098	$18930_{3}^{{}^{\circ}}$ – 25890_2_
14358.938	2		6962.400	$22141_{3}^{{}^{\circ}}$ – 29104_4_
14357.839	3		6962.933	7502_3_ – $14465_{2}^{{}^{\circ}}$
14355.909	0		6963.869	7280_2_ – $14243_{1}^{{}^{\circ}}$
14340.036	0		6971.577	$22877_{0}^{{}^{\circ}}$ – 29849_4_
14326.032	1		6978.392	$22163_{4}^{{}^{\circ}}$ – 29141_5_
14324.702	0		6979.040	9804_5_ – $16783_{4}^{{}^{\circ}}$
14323.255	1		6979.745	$22855_{3}^{{}^{\circ}}$ – 29835_3_
14313.992	4		6984.262	$10414_{4}^{{}^{\circ}}$ – 17398_3_
14306.560	2		6987.890	$7411_{3}^{{}^{\circ}}$ – 24399_3_
14297.496	0		6992.320	$21890_{3}^{{}^{\circ}}$ – 28882_2_
14296.656	2		6992.731	$18930_{3}^{{}^{\circ}}$ – 25923_4_
14294.346	0		6993.861	$22855_{3}^{{}^{\circ}}$ – 29849_4_
14283.487	1		6999.178	$23015_{0}^{{}^{\circ}}$ – 30014_4_
14271.876	1		7004.872	$24769_{3}^{{}^{\circ}}$ – 31774_3_
14270.962	1		7005.321	$23752_{2}^{{}^{\circ}}$ – 30758_2_
14259.243	5		7011.078	$19986_{6}^{{}^{\circ}}$ – 26997_6_
14254.713	5		7013.306	11601_1_ – $18614_{1}^{{}^{\circ}}$
14254.065	0		7013.625	15863_2_ – $22877_{1}^{{}^{\circ}}$
14253.783	1		7013.764	19273_2_ – $26287_{1}^{{}^{\circ}}$
14244.211	2		7018.477	$15618_{3}^{{}^{\circ}}$ – 22637_3_
14239.881	1		7020.611	$21252_{2}^{{}^{\circ}}$ – 28273_2_
14238.660	2		7021.213	17959_4_ – $24981_{3}^{{}^{\circ}}$
14237.955	1		7021.561	$22396_{1}^{{}^{\circ}}$ – 29418_2_
14235.729	2		7022.659	17398_3_ – $24421_{3}^{{}^{\circ}}$
14234.902	2		7023.067	$19948_{4}^{{}^{\circ}}$ – 26971_4_
14234.902	2		7023.067	$21539_{4}^{{}^{\circ}}$ – 28562_4_
14233.051	0		7023.980	$24769_{3}^{{}^{\circ}}$ – 31793_4_
14231.327	4		7024.831	13297_4_ – $20322_{0}^{{}^{\circ}}$
14230.620	0		7025.180	$20566_{4}^{{}^{\circ}}$ – 27591_5_
14230.460	0		7025.259	$22396_{1}^{{}^{\circ}}$ – 29422_1_
14217.138	1		7031.842	$24981_{3}^{{}^{\circ}}$ – 32012_4_
14214.389	0		7033.202	20054_2_ – $27087_{1}^{{}^{\circ}}$
14192.323	4		7044.137	$20522_{2}^{{}^{\circ}}$ – 27566_2_
14191.050	2		7044.769	$21890_{3}^{{}^{\circ}}$ – 28934_3_
14189.283	0		7045.646	$20566_{4}^{{}^{\circ}}$ – 27612_3_
14187.489	2		7046.537	$25690_{0}^{{}^{\circ}}$ – 32737_5_
14170.625	0		7054.923	16554_6_ – $23609_{0}^{{}^{\circ}}$
14168.671	7		7055.896	8111_4_ – $15166_{3}^{{}^{\circ}}$
14160.194	1		7060.120	$24981_{3}^{{}^{\circ}}$ – 32041_2_
14154.750	0		7062.835	$21165_{3}^{{}^{\circ}}$ – 28227_4_
14148.646	2		7065.882	$23306_{6}^{{}^{\circ}}$ – 30372_6_
14107.467	1		7086.507	$22669_{3}^{{}^{\circ}}$ – 29756_4_
14101.396	3		7089.558	$20522_{2}^{{}^{\circ}}$ – 27612_3_
14100.879	2		7089.818	19273_2_ – $26363_{2}^{{}^{\circ}}$
14095.249	1		7092.650	$24701_{0}^{{}^{\circ}}$ – 31793_4_
14090.246	7		7095.168	3687_2_ – $10783_{2}^{{}^{\circ}}$
14079.818	5		7100.423	12847_3_ – $19948_{4}^{{}^{\circ}}$
14063.614	2		7108.604	17166_5_ – $24274_{0}^{{}^{\circ}}$
14063.614	2		7108.604	17073_1_ – $24182_{2}^{{}^{\circ}}$
14061.486	0		7109.680	18699_2_ – $25809_{1}^{{}^{\circ}}$
14061.215	1		7109.817	$14465_{2}^{{}^{\circ}}$ – 21575_2_
14054.652	1		7113.137	$18809_{4}^{{}^{\circ}}$ – 25923_4_
14038.143	0		7121.502	$24307_{2}^{{}^{\circ}}$ – 31429_1_
14032.419	1		7124.407	$24202_{4}^{{}^{\circ}}$ – 31326_4_
14028.560	4		7126.367	13088_3_ – $20214_{3}^{{}^{\circ}}$
14026.605	5		7127.360	11802_2_ – $18930_{3}^{{}^{\circ}}$
14008.158	0		7136.746	$22877_{0}^{{}^{\circ}}$ – 30014_4_
14007.173	0		7137.248	$23752_{2}^{{}^{\circ}}$ – 30889_1_
13995.011	0		7143.450	15970_3_ – $23113_{4}^{{}^{\circ}}$
13993.145	1		7144.403	$21738_{2}^{{}^{\circ}}$ – 28882_2_
13976.090	2		7153.121	$22399_{0}^{{}^{\circ}}$ – 29552_6_
13975.932	4		7153.202	$19227_{6}^{{}^{\circ}}$ – 26380_5_
13974.383	1		7153.995	18549_2_ – $25703_{2}^{{}^{\circ}}$
13970.381	2		7156.044	$22855_{3}^{{}^{\circ}}$ – 30011_3_
13964.777	2		7158.916	$12114_{2}^{{}^{\circ}}$ – 19273_2_
13936.736	5		7173.320	$20322_{0}^{{}^{\circ}}$ – 27495_4_
13930.234	3		7176.668	15493_4_ – $22669_{3}^{{}^{\circ}}$
13904.491	6		7189.955	$11241_{3}^{{}^{\circ}}$ – 18431_3_
13898.031	1		7193.297	$21165_{3}^{{}^{\circ}}$ – 28358_3_
13886.527	4		7199.256	$20322_{0}^{{}^{\circ}}$ – 27521_4_
13882.834	1		7201.171	$23306_{6}^{{}^{\circ}}$ – 30508_5_
13872.468	0		7206.552	$25575_{4}^{{}^{\circ}}$ – 32781_4_
13862.050	0		7211.968	$23752_{2}^{{}^{\circ}}$ – 30964_3_
13859.106	1		7213.500	$21668_{1}^{{}^{\circ}}$ – 28882_2_
13844.566	0		7221.076	$25575_{4}^{{}^{\circ}}$ – 32796_3_
13818.508	1		7234.693	18574_1_ – $25809_{1}^{{}^{\circ}}$
13815.598	1		7236.217	11802_2_ – $19039_{2}^{{}^{\circ}}$
13828.453	1		7229.490	$24307_{2}^{{}^{\circ}}$ – 31537_3_
13814.181	0		7236.959	$23521_{3}^{{}^{\circ}}$ – 30758_2_
13813.265	0		7237.439	$23049_{1}^{{}^{\circ}}$ – 30286_1_
13809.796	1		7239.257	$21902_{4}^{{}^{\circ}}$ – 29141_5_
13799.139	0		7244.848	$25306_{2}^{{}^{\circ}}$ – 32551_3_
13795.378	1		7246.823	$24182_{2}^{{}^{\circ}}$ – 31429_1_
13792.422	5		7248.376	$16783_{4}^{{}^{\circ}}$ – 24032_4_
13786.380	1		7251.553	$15736_{1}^{{}^{\circ}}$ – 22988_2_
13779.592	1		7255.125	$22877_{1}^{{}^{\circ}}$ – 30132_2_
13774.118	1		7258.008	19532_4_ – $26790_{4}^{{}^{\circ}}$
13760.203	2		7265.348	$21077_{0}^{{}^{\circ}}$ – 28342_5_
13752.928	2		7269.191	13297_4_ – $20566_{4}^{{}^{\circ}}$
13752.277	0		7269.535	$20322_{0}^{{}^{\circ}}$ – 27591_5_
13748.535	0		7271.514	$24769_{3}^{{}^{\circ}}$ – 32041_2_
13748.149	2		7271.718	18431_3_ – $25703_{2}^{{}^{\circ}}$
13745.894	0		7272.911	$25690_{0}^{{}^{\circ}}$ – 32963_5_
13739.956	5		7276.054	$18614_{1}^{{}^{\circ}}$ – 25890_2_
13738.583	3		7276.781	$22141_{3}^{{}^{\circ}}$ – 29418_2_
13735.446	2		7278.443	$21252_{2}^{{}^{\circ}}$ – 28531_2_
13734.218	2		7279.094	$19516_{2}^{{}^{\circ}}$ – 26796_3_
13731.271	2		7280.656	$20214_{3}^{{}^{\circ}}$ – 27495_4_
13727.340	1		7282.741	19713_3_ – $26995_{3}^{{}^{\circ}}$
13723.460	0		7284.800	$22399_{0}^{{}^{\circ}}$ – 29684_5_
13680.790	0		7307.521	17073_1_ – $24381_{2}^{{}^{\circ}}$
13680.494	1		7307.679	$11241_{3}^{{}^{\circ}}$ – 18549_2_
13677.064	2		7309.512	$23655_{4}^{{}^{\circ}}$ – 30964_2_
13672.580	0		7311.909	$24701_{0}^{{}^{\circ}}$ – 32012_4_
13672.490	0		7311.957	$22338_{3}^{{}^{\circ}}$ – 29650_2_
13668.402	0		7314.144	$25753_{0}^{{}^{\circ}}$ – 33068_6_
13662.574	2		7317.264	$17354_{1}^{{}^{\circ}}$ – 24671_2_
13662.466	1		7317.322	13847_2_ – $21165_{3}^{{}^{\circ}}$
13647.606	5		7325.289	$18930_{3}^{{}^{\circ}}$ – 26255_4_
13628.932	1		7335.326	14204_5_ – $21539_{4}^{{}^{\circ}}$
13612.012	0		7344.444	$22669_{3}^{{}^{\circ}}$ – 30014_4_
13609.614	2		7345.738	19532_4_ – $26878_{3}^{{}^{\circ}}$
13599.702	3		7351.092	$20922_{2}^{{}^{\circ}}$ – 28273_2_
13598.599	3		7351.688	$24850_{6}^{{}^{\circ}}$ – 32202_5_
13598.167	2		7351.922	$20214_{3}^{{}^{\circ}}$ – 27566_2_
13597.830	1		7352.104	$24274_{0}^{{}^{\circ}}$ – 31626_5_
13595.363	0		7353.438	$25442_{3}^{{}^{\circ}}$ – 32796_3_
13592.829	0		7354.809	$24182_{2}^{{}^{\circ}}$ – 31537_3_
13588.783	4		7356.999	$22399_{0}^{{}^{\circ}}$ – 29756_4_
13587.748	0		7357.559	$23015_{0}^{{}^{\circ}}$ – 30372_6_
13579.410	4		7362.077	15493_4_ – $22855_{3}^{{}^{\circ}}$
13572.266	0		7365.952	$21165_{3}^{{}^{\circ}}$ – 28531_2_
13570.411	3		7366.959	12847_3_ – $20214_{3}^{{}^{\circ}}$
13565.665	7		7369.536	$15618_{3}^{{}^{\circ}}$ – 22988_2_
13562.388	2		7371.317	17398_3_ – $24769_{3}^{{}^{\circ}}$
13549.102	2		7378.545	19273_2_ – $26651_{2}^{{}^{\circ}}$
13540.748	3		7383.097	$19588_{0}^{{}^{\circ}}$ – 26971_4_
13538.429	2		7384.362	15493_4_ – $22877_{0}^{{}^{\circ}}$
13532.204	2		7387.759	$14206_{4}^{{}^{\circ}}$ – 21594_3_
13529.689	1		7389.132	16351_0_ – $23741_{1}^{{}^{\circ}}$
13518.143	6		7395.443	$11877_{1}^{{}^{\circ}}$ – 19273_2_
13517.712	2		7395.679	17959_4_ – $25355_{4}^{{}^{\circ}}$
13514.671	1		7397.343	$20214_{3}^{{}^{\circ}}$ – 27612_3_
13508.886	0		7400.511	$25336_{6}^{{}^{\circ}}$ – 32737_5_
13501.001	4		7404.833	13847_2_ – $21252_{2}^{{}^{\circ}}$
13491.454	1		7410.073	$23916_{4}^{{}^{\circ}}$ – 31326_4_
13484.963	1		7413.640	18699_2_ – $26113_{2}^{{}^{\circ}}$
13484.304	3		7414.002	$15618_{3}^{{}^{\circ}}$ – 23032_4_
13478.127	0		7417.400	$22338_{3}^{{}^{\circ}}$ – 29756_4_
13465.671	0		7424.261	$24202_{4}^{{}^{\circ}}$ – 31626_5_
13449.197	4		7433.355	$10526_{3}^{{}^{\circ}}$ – 17959_4_
13448.036	1		7433.997	$24561_{3}^{{}^{\circ}}$ – 31995_4_
13447.755	4		7434.152	13088_3_ – $20522_{2}^{{}^{\circ}}$
13443.925	4		7436.270	$20922_{2}^{{}^{\circ}}$ – 28358_3_
13440.583	4		7438.119	11601_1_ – $19039_{2}^{{}^{\circ}}$
13439.085	1		7438.948	$14206_{4}^{{}^{\circ}}$ – 21645_4_
13436.414	0		7440.427	$17224_{2}^{{}^{\circ}}$ – 24664_3_
13433.332	3		7442.134	14226_0_ – $21668_{1}^{{}^{\circ}}$
13426.907	3		7445.695	$18809_{4}^{{}^{\circ}}$ – 26255_4_
13426.686	2		7445.818	18431_3_ – $25877_{4}^{{}^{\circ}}$
13423.480	2		7447.596	$17224_{2}^{{}^{\circ}}$ – 24671_2_
13419.576	4		7449.763	$22399_{0}^{{}^{\circ}}$ – 29849_4_
13418.102	0		7450.581	$23093_{2}^{{}^{\circ}}$ – 30544_2_
13404.943	4		7457.895	$11241_{3}^{{}^{\circ}}$ – 18699_2_
13395.136	1		7463.355	19532_4_ – $26995_{3}^{{}^{\circ}}$
13387.914	0		7467.381	$20566_{4}^{{}^{\circ}}$ – 28034_5_
13386.236	2		7468.317	$19503_{3}^{{}^{\circ}}$ – 26971_4_
13382.214	4		7470.562	$15166_{3}^{{}^{\circ}}$ – 22637_3_
13370.573	1		7477.066	$24981_{3}^{{}^{\circ}}$ – 32458_4_
13368.792	6		7478.062	13088_3_ – $20566_{4}^{{}^{\circ}}$
13360.349	0		7482.788	17959_4_ – $25442_{3}^{{}^{\circ}}$
13355.490	0		7485.510	$21077_{0}^{{}^{\circ}}$ – 28562_4_
13353.446	0		7486.656	$23655_{4}^{{}^{\circ}}$ – 31141_3_
13352.939	0		7486.940	18549_2_ – $26036_{3}^{{}^{\circ}}$
13348.625	1		7489.360	$25306_{2}^{{}^{\circ}}$ – 32796_3_
13342.411	1		7492.848	$23015_{0}^{{}^{\circ}}$ – 30508_5_
13336.712	3		7496.050	$22338_{3}^{{}^{\circ}}$ – 29835_3_
13334.671	1		7497.197	$18614_{1}^{{}^{\circ}}$ – 26111_1_
13322.946	0		7503.795	$17411_{3}^{{}^{\circ}}$ – 24915_3_
13315.518	1		7507.981	8111_4_ – $15618_{3}^{{}^{\circ}}$
13314.704	1		7508.440	$24421_{3}^{{}^{\circ}}$ – 31929_3_
13303.409	1		7514.815	$24259_{4}^{{}^{\circ}}$ – 31774_3_
13296.036	1		7518.982	$24274_{0}^{{}^{\circ}}$ – 31793_4_
13292.355	0		7521.064	$22163_{4}^{{}^{\circ}}$ – 29684_5_
13290.823	1		7521.931	15493_4_ – $23015_{0}^{{}^{\circ}}$
13286.526	0		7524.364	$25575_{4}^{{}^{\circ}}$ – 33099_3_
13281.453	4		7527.238	$19516_{2}^{{}^{\circ}}$ – 27044_3_
13279.662	0		7528.253	$21890_{3}^{{}^{\circ}}$ – 29418_2_
13275.351	5		7530.698	16554_6_ – $24084_{6}^{{}^{\circ}}$
13269.677	1		7533.918	$24259_{4}^{{}^{\circ}}$ – 31793_4_
13267.880	2		7534.938	17166_5_ – $24701_{0}^{{}^{\circ}}$
13263.633	2		7537.351	$23015_{0}^{{}^{\circ}}$ – 30552_4_
13261.338	0		7538.655	18574_1_ – $26113_{2}^{{}^{\circ}}$
13257.081	3		7541.076	$19503_{3}^{{}^{\circ}}$ – 27044_3_
13253.863	5		7542.907	$15490_{0}^{{}^{\circ}}$ – 23032_4_
13250.406	1		7544.875	2869_3_ – $10414_{4}^{{}^{\circ}}$
13248.848	2		7545.762	$10414_{4}^{{}^{\circ}}$ – 17959_4_
13247.730	4		7546.399	8800_4_ – $16346_{4}^{{}^{\circ}}$
13246.336	0		7547.193	$19948_{4}^{{}^{\circ}}$ – 27495_4_
13245.664	2		7547.576	18549_2_ – $26096_{3}^{{}^{\circ}}$
13239.744	4		7550.951	15970_3_ – $23521_{3}^{{}^{\circ}}$
13236.164	3		7552.993	19713_3_ – $27266_{4}^{{}^{\circ}}$
13234.852	2		7553.742	3687_2_ – $11241_{3}^{{}^{\circ}}$
13219.366	0		7562.591	$14032_{2}^{{}^{\circ}}$ – 21594_3_
13217.149	0		7563.859	18549_2_ – $26113_{2}^{{}^{\circ}}$
13216.178	1		7564.415	$21539_{4}^{{}^{\circ}}$ – 29104_4_
13215.207	0		7564.971	$22396_{1}^{{}^{\circ}}$ – 29961_1_
13205.290	6		7570.652	$18809_{4}^{{}^{\circ}}$ – 26380_5_
13202.889	1		7572.029	$24202_{4}^{{}^{\circ}}$ – 31774_3_
13200.969	1		7573.130	$19948_{4}^{{}^{\circ}}$ – 27521_4_
13184.286	2		7582.713	17398_3_ – $24981_{3}^{{}^{\circ}}$
13183.941	3		7582.911	6362_2_ – $13945_{3}^{{}^{\circ}}$
13183.783	3		7583.002	$15618_{3}^{{}^{\circ}}$ – 23201_3_
13176.103	2		7587.422	18699_2_ – $26287_{1}^{{}^{\circ}}$
13169.665	0		7591.131	$24202_{4}^{{}^{\circ}}$ – 31793_4_
13168.345	0		7591.892	$24421_{3}^{{}^{\circ}}$ – 32012_4_
13167.879	0		7592.161	$24182_{2}^{{}^{\circ}}$ – 31774_3_
13156.601	1		7598.669	$12114_{2}^{{}^{\circ}}$ – 19713_3_
13150.947	1		7601.936	$21539_{4}^{{}^{\circ}}$ – 29141_5_
13146.765	1		7604.354	19713_3_ – $27317_{3}^{{}^{\circ}}$
13146.229	1		7604.664	18431_3_ – $26036_{3}^{{}^{\circ}}$
13144.547	1		7605.637	$19986_{6}^{{}^{\circ}}$ – 27591_5_
13136.162	0		7610.492	$23655_{4}^{{}^{\circ}}$ – 31265_3_
13128.501	2		7614.933	$22399_{0}^{{}^{\circ}}$ – 30014_4_
13127.923	5		7615.268	$16783_{4}^{{}^{\circ}}$ – 24399_3_
13126.337	0		7616.188	20054_2_ – $27670_{3}^{{}^{\circ}}$
13124.108	1		7617.482	15863_2_ – $23481_{1}^{{}^{\circ}}$
13120.544	1		7619.551	20054_2_ – $27674_{2}^{{}^{\circ}}$
13119.476	1		7620.171	$24421_{3}^{{}^{\circ}}$ – 32041_2_
13116.755	1		7621.752	$24307_{2}^{{}^{\circ}}$ – 31929_3_
13111.862	0		7624.596	$22508_{2}^{{}^{\circ}}$ – 30132_2_
13107.912	1		7626.894	$25336_{6}^{{}^{\circ}}$ – 32963_5_
13103.036	0		7629.732	$13945_{3}^{{}^{\circ}}$ – 21575_2_
13102.832	1		7629.851	$21252_{2}^{{}^{\circ}}$ – 28882_2_
13101.858	1		7630.418	$22877_{0}^{{}^{\circ}}$ – 30508_5_
13079.586	1		7643.411	$19948_{4}^{{}^{\circ}}$ – 27591_5_
13070.834	6		7648.529	$10783_{2}^{{}^{\circ}}$ – 18431_3_
13069.399	3		7649.369	$13945_{3}^{{}^{\circ}}$ – 21594_3_
13056.097	2		7657.162	15863_2_ – $23521_{3}^{{}^{\circ}}$
13055.889	6		7657.284	2869_3_ – $10526_{3}^{{}^{\circ}}$
13049.034	2		7661.307	$20566_{4}^{{}^{\circ}}$ – 28227_4_
13048.139	1		7661.832	$23609_{0}^{{}^{\circ}}$ – 31271_5_
13045.340	1		7663.476	18699_2_ – $26363_{2}^{{}^{\circ}}$
13043.407	4		7664.612	7502_3_ – $15166_{3}^{{}^{\circ}}$
13034.773	2		7669.689	6362_2_ – $14032_{2}^{{}^{\circ}}$
13031.133	3		7671.831	$23655_{4}^{{}^{\circ}}$ – 31326_4_
13030.257	0		7672.347	$22338_{3}^{{}^{\circ}}$ – 30011_3_
13026.187	6		7674.744	12847_3_ – $20522_{2}^{{}^{\circ}}$
13023.092	1		7676.568	$23752_{2}^{{}^{\circ}}$ – 31429_1_
13010.439	1		7684.034	$21738_{2}^{{}^{\circ}}$ – 29422_1_
13010.154	3		7684.202	17166_5_ – $24850_{6}^{{}^{\circ}}$
13008.742	0		7685.036	15970_3_ – $23655_{4}^{{}^{\circ}}$
13007.832	5		7685.574	$16346_{4}^{{}^{\circ}}$ – 24032_4_
12994.532	3		7693.440	$22141_{3}^{{}^{\circ}}$ – 29835_3_
12989.585	1		7696.370	9804_5_ – $17501_{0}^{{}^{\circ}}$
12988.177	1		7697.204	$22855_{3}^{{}^{\circ}}$ – 30552_4_
12986.920	4		7697.949	$7795_{4}^{{}^{\circ}}$ – 15493_4_
12983.105	0		7700.211	11802_2_ – $19503_{3}^{{}^{\circ}}$
12982.529	1		7700.553	$13945_{3}^{{}^{\circ}}$ – 21645_4_
12970.735	2		7707.555	$22141_{3}^{{}^{\circ}}$ – 29849_4_
12966.746	0		7709.926	$23916_{4}^{{}^{\circ}}$ – 31626_5_
12963.697	2		7711.739	$20322_{0}^{{}^{\circ}}$ – 28034_5_
12959.819	7		7714.047	11802_2_ – $19516_{2}^{{}^{\circ}}$
12952.082	5		7718.655	12847_3_ – $20566_{4}^{{}^{\circ}}$
12949.037	1		7720.470	16554_6_ – $24274_{0}^{{}^{\circ}}$
12948.255	0		7720.936	$24274_{0}^{{}^{\circ}}$ – 31995_4_
12938.933	2		7726.499	$8243_{2}^{{}^{\circ}}$ – 15970_3_
12936.840	1		7727.749	19532_4_ – $27260_{3}^{{}^{\circ}}$
12935.233	3		7728.709	$18382_{0}^{{}^{\circ}}$ – 26111_1_
12933.760	1		7729.589	20054_2_ – $27784_{2}^{{}^{\circ}}$
12932.107	2		7730.577	17959_4_ – $25690_{0}^{{}^{\circ}}$
12930.425	1		7731.583	$25336_{6}^{{}^{\circ}}$ – 33068_6_
12925.197	1		7734.710	19713_3_ – $27447_{2}^{{}^{\circ}}$
12923.256	0		7735.872	$24259_{4}^{{}^{\circ}}$ – 31995_4_
12920.304	0		7737.639	18549_2_ – $26287_{1}^{{}^{\circ}}$
12919.299	3		7738.241	$24274_{0}^{{}^{\circ}}$ – 32012_4_
12916.981	4		7739.630	15863_2_ – $23603_{2}^{{}^{\circ}}$
12900.246	2		7749.670	$19817_{1}^{{}^{\circ}}$ – 27566_2_
12898.865	4		7750.500	$20522_{2}^{{}^{\circ}}$ – 28273_2_
12888.182	2		7756.924	$19039_{2}^{{}^{\circ}}$ – 26796_3_
12881.709	0		7760.822	$21890_{3}^{{}^{\circ}}$ – 29650_2_
12874.595	1		7765.110	17073_1_ – $24838_{1}^{{}^{\circ}}$
12873.533	1		7765.751	$20214_{3}^{{}^{\circ}}$ – 27980_3_
12872.702	3		7766.252	$10783_{2}^{{}^{\circ}}$ – 18549_2_
12866.644	7		7769.909	$19227_{6}^{{}^{\circ}}$ – 26997_6_
12861.651	4		7772.925	$16217_{2}^{{}^{\circ}}$ – 23990_2_
12854.102	1		7777.490	$25321_{3}^{{}^{\circ}}$ – 33099_3_
12846.823	1		7781.897	$24769_{3}^{{}^{\circ}}$ – 32551_3_
12845.690	1		7782.583	15970_3_ – $23752_{2}^{{}^{\circ}}$
12841.750	2		7784.971	19532_4_ – $27317_{3}^{{}^{\circ}}$
12837.894	2		7787.309	$15490_{0}^{{}^{\circ}}$ – 23277_5_
12836.536	1		7788.133	19273_2_ – $27061_{2}^{{}^{\circ}}$
12831.063	0		7791.455	$10783_{2}^{{}^{\circ}}$ – 18574_1_
12830.547	3		7791.768	$20566_{4}^{{}^{\circ}}$ – 28358_3_
12828.379	1		7793.085	$24202_{4}^{{}^{\circ}}$ – 31995_4_
12827.456	0		7793.646	$22338_{3}^{{}^{\circ}}$ – 30132_2_
12826.806	3		7794.041	17959_4_ – $25753_{0}^{{}^{\circ}}$
12799.956	2		7810.390	$24202_{4}^{{}^{\circ}}$ – 32012_4_
12782.294	1		7821.182	13847_2_ – $21668_{1}^{{}^{\circ}}$
12782.059	2		7821.326	$18069_{3}^{{}^{\circ}}$ – 25890_2_
12781.578	4		7821.620	$15166_{3}^{{}^{\circ}}$ – 22988_2_
12773.189	1		7826.757	$19516_{2}^{{}^{\circ}}$ – 27343_3_
12762.096	6		7833.560	13088_3_ – $20922_{2}^{{}^{\circ}}$
12750.649	0		7840.593	$19503_{3}^{{}^{\circ}}$ – 27343_3_
12738.275	0		7848.209	$22163_{4}^{{}^{\circ}}$ – 30011_3_
12733.582	3		7851.102	$23113_{4}^{{}^{\circ}}$ – 30964_3_
12709.820	4		7865.780	$18930_{3}^{{}^{\circ}}$ – 26796_3_
12709.330	5		7866.083	$15166_{3}^{{}^{\circ}}$ – 23032_4_
12703.962	0		7869.407	$18053_{4}^{{}^{\circ}}$ – 25923_4_
12703.428	1		7869.738	$22141_{3}^{{}^{\circ}}$ – 30011_3_
12691.421	4		7877.183	15863_2_ – $23741_{1}^{{}^{\circ}}$
12685.466	1		7880.881	$16783_{4}^{{}^{\circ}}$ – 24664_3_
12684.317	0		7881.595	6362_2_ – $14243_{1}^{{}^{\circ}}$
12683.507	1		7882.098	$23655_{4}^{{}^{\circ}}$ – 31537_3_
12682.679	0		7882.613	$22669_{3}^{{}^{\circ}}$ – 30552_4_
12680.944	1		7883.691	$22248_{2}^{{}^{\circ}}$ – 30132_2_
12673.959	2		7888.036	$25180_{7}^{{}^{\circ}}$ – 33068_6_
12670.355	1		7890.280	13847_2_ – $21738_{2}^{{}^{\circ}}$
12668.767	2		7891.269	$14206_{4}^{{}^{\circ}}$ – 22098_4_
12648.388	1		7903.983	$17501_{0}^{{}^{\circ}}$ – 25405_4_
12646.536	8		7905.141	$10526_{3}^{{}^{\circ}}$ – 18431_3_
12645.697	4		7905.665	$20322_{0}^{{}^{\circ}}$ – 28227_4_
12641.383	3		7908.363	17398_3_ – $25306_{2}^{{}^{\circ}}$
12636.142	5		7911.643	$18011_{0}^{{}^{\circ}}$ – 25923_4_
12632.545	3		7913.896	2869_3_ – $10783_{2}^{{}^{\circ}}$
12629.269	0		7915.949	11601_1_ – $19516_{2}^{{}^{\circ}}$
12617.200	1		7923.521	17398_3_ – $25321_{3}^{{}^{\circ}}$
12611.214	0		7927.282	$24274_{0}^{{}^{\circ}}$ – 32202_5_
12604.640	2		7931.416	18431_3_ – $26363_{2}^{{}^{\circ}}$
12587.889	4		7941.971	$20737_{1}^{{}^{\circ}}$ – 28679_2_
12583.229	1		7944.912	$21890_{3}^{{}^{\circ}}$ – 29835_3_
12580.239	1		7946.800	15970_3_ – $23916_{4}^{{}^{\circ}}$
12580.100	1		7946.888	$21902_{4}^{{}^{\circ}}$ – 29849_4_
12571.690	2		7952.204	18699_2_ – $26651_{2}^{{}^{\circ}}$
12563.833	0		7957.177	17398_3_ – $25355_{4}^{{}^{\circ}}$
12562.651	2		7957.926	19713_3_ – $27670_{3}^{{}^{\circ}}$
12561.555	3		7958.620	18549_2_ – $26508_{3}^{{}^{\circ}}$
12561.165	1		7958.867	14204_5_ – $22163_{4}^{{}^{\circ}}$
12558.855	1		7960.331	$20922_{2}^{{}^{\circ}}$ – 28882_2_
12553.032	5		7964.024	$19227_{6}^{{}^{\circ}}$ – 27191_5_
12552.801	4		7964.170	$23306_{6}^{{}^{\circ}}$ – 31271_5_
12540.971	2		7971.683	$23655_{4}^{{}^{\circ}}$ – 31626_5_
12522.256	1		7983.597	8800_4_ – $16783_{4}^{{}^{\circ}}$
12518.195	3		7986.187	$18809_{4}^{{}^{\circ}}$ – 26796_3_
12508.396	2		7992.443	$19503_{3}^{{}^{\circ}}$ – 27495_4_
12502.774	2		7996.037	$20566_{4}^{{}^{\circ}}$ – 28562_4_
12488.669	1		8005.068	$19039_{2}^{{}^{\circ}}$ – 27044_3_
12477.297	7		8012.364	3865_1_ – $11877_{1}^{{}^{\circ}}$
12476.305	3		8013.001	$20214_{3}^{{}^{\circ}}$ – 28227_4_
12474.364	2		8014.248	11802_2_ – $19817_{1}^{{}^{\circ}}$
12471.346	1		8016.187	$23521_{3}^{{}^{\circ}}$ – 31537_3_
12469.228	4		8017.549	$10414_{4}^{{}^{\circ}}$ – 18431_3_
12466.651	0		8019.206	$24274_{0}^{{}^{\circ}}$ – 32293_3_
12465.053	3		8020.234	$20322_{0}^{{}^{\circ}}$ – 28342_5_
12460.967	4		8022.864	$10526_{3}^{{}^{\circ}}$ – 18549_2_
12455.465	0		8026.408	$24769_{3}^{{}^{\circ}}$ – 32796_3_
12453.262	3		8027.828	15493_4_ – $23521_{3}^{{}^{\circ}}$
12447.488	5		8031.552	$11241_{3}^{{}^{\circ}}$ – 19273_2_
12446.347	1		8032.288	$19948_{4}^{{}^{\circ}}$ – 27980_3_
12445.393	3		8032.904	$15736_{1}^{{}^{\circ}}$ – 23769_1_
12442.013	5		8035.086	$15166_{3}^{{}^{\circ}}$ – 23201_3_
12440.650	2		8035.966	$24701_{0}^{{}^{\circ}}$ – 32737_5_
12437.583	0		8037.948	$22248_{2}^{{}^{\circ}}$ – 30286_1_
12430.756	1		8042.362	13847_2_ – $21890_{3}^{{}^{\circ}}$
12429.285	1		8043.314	$17847_{2}^{{}^{\circ}}$ – 25890_2_
12422.300	4		8047.837	$19986_{6}^{{}^{\circ}}$ – 28034_5_
12419.161	2		8049.871	$19516_{2}^{{}^{\circ}}$ – 27566_2_
12415.160	5		8052.465	$16346_{4}^{{}^{\circ}}$ – 24399_3_
12406.195	1		8058.284	$20214_{3}^{{}^{\circ}}$ – 28273_2_
12397.849	4		8063.709	$19503_{3}^{{}^{\circ}}$ – 27566_2_
12396.828	0		8064.373	$21077_{0}^{{}^{\circ}}$ – 29141_5_
12381.813	3		8074.152	12847_3_ – $20922_{2}^{{}^{\circ}}$
12378.451	1		8076.345	18431_3_ – $26508_{3}^{{}^{\circ}}$
12378.290	2		8076.450	17959_4_ – $26036_{3}^{{}^{\circ}}$
12377.115	0		8077.217	18574_1_ – $26651_{2}^{{}^{\circ}}$
12372.007	2		8080.552	$24701_{0}^{{}^{\circ}}$ – 32781_4_
12364.267	1		8085.610	$19948_{4}^{{}^{\circ}}$ – 28034_5_
12359.625	2		8088.647	17959_4_ – $26048_{4}^{{}^{\circ}}$
12355.491	0		8091.353	$24202_{4}^{{}^{\circ}}$ – 32293_5_
12349.478	2		8095.293	$19516_{2}^{{}^{\circ}}$ – 27612_3_
12348.314	0		8096.056	$23916_{4}^{{}^{\circ}}$ – 32012_4_
12346.884	0		8096.994	$21738_{2}^{{}^{\circ}}$ – 29835_3_
12338.612	0		8102.422	18549_2_ – $26651_{2}^{{}^{\circ}}$
12337.998	7		8102.825	6362_2_ – $14465_{2}^{{}^{\circ}}$
12330.800	1		8107.555	$20423_{1}^{{}^{\circ}}$ – 28531_2_
12329.207	2		8108.603	$22399_{0}^{{}^{\circ}}$ – 30508_5_
12328.492	1		8109.073	$21902_{4}^{{}^{\circ}}$ – 30011_3_
12328.408	0		8109.128	$19503_{3}^{{}^{\circ}}$ – 27612_3_
12323.962	0		8112.054	$21902_{4}^{{}^{\circ}}$ – 30014_4_
12322.400	1		8113.082	$24850_{6}^{{}^{\circ}}$ – 32963_5_
12321.120	3		8113.925	$18930_{3}^{{}^{\circ}}$ – 27044_3_
12318.050	3		8115.947	15493_4_ – $23609_{0}^{{}^{\circ}}$
12316.913	2		8116.696	7502_3_ – $15618_{3}^{{}^{\circ}}$
12316.372	1		8117.053	$24084_{6}^{{}^{\circ}}$ – 32202_5_
12312.736	2		8119.450	$23655_{4}^{{}^{\circ}}$ – 31774_3_
12310.069	3		8121.209	$21890_{3}^{{}^{\circ}}$ – 30011_3_
12295.924	0		8130.551	$24421_{3}^{{}^{\circ}}$ – 32551_3_
12294.985	1		8131.172	$16783_{4}^{{}^{\circ}}$ – 24915_3_
12283.851	1		8138.542	19532_4_ – $27670_{3}^{{}^{\circ}}$
12283.851	1		8138.542	$23655_{4}^{{}^{\circ}}$ – 31793_4_
12276.430	0		8143.462	$20214_{3}^{{}^{\circ}}$ – 28358_3_
12273.859	2		8145.168	$20737_{1}^{{}^{\circ}}$ – 28882_2_
12271.398	2		8146.801	16554_6_ – $24701_{0}^{{}^{\circ}}$
12263.449	1		8152.082	$23113_{4}^{{}^{\circ}}$ – 31265_3_
12262.250	1		8152.879	$13945_{3}^{{}^{\circ}}$ – 22098_4_
12261.910	2		8153.105	$22399_{0}^{{}^{\circ}}$ – 30552_4_
12256.743	2		8156.542	$20522_{2}^{{}^{\circ}}$ – 28679_2_
12249.188	4		8161.573	$18809_{4}^{{}^{\circ}}$ – 26971_4_
12245.483	3		8164.042	13088_3_ – $21252_{2}^{{}^{\circ}}$
12242.874	0		8165.782	$21252_{2}^{{}^{\circ}}$ – 29478_2_
12237.332	1		8169.480	$21252_{2}^{{}^{\circ}}$ – 29422_1_
12236.549	2		8170.003	14226_0_ – $22396_{1}^{{}^{\circ}}$
12234.949	1		8171.071	$23603_{2}^{{}^{\circ}}$ – 31774_3_
12233.199	5		8172.240	$14465_{2}^{{}^{\circ}}$ – 22637_3_
12231.942	8		8173.080	$10526_{3}^{{}^{\circ}}$ – 18699_2_
12229.332	1		8174.824	$7795_{4}^{{}^{\circ}}$ – 15970_3_
12226.604	1		8176.648	17398_3_ – $25575_{4}^{{}^{\circ}}$
12226.177	4		8176.934	$11877_{1}^{{}^{\circ}}$ – 20054_2_
12223.788	2		8178.532	18699_2_ – $26878_{3}^{{}^{\circ}}$
12219.156	0		8181.632	$16217_{2}^{{}^{\circ}}$ – 24399_3_
12216.424	1		8183.462	$24274_{0}^{{}^{\circ}}$ – 32458_4_
12211.865	2		8186.517	$18069_{3}^{{}^{\circ}}$ – 26255_4_
12206.894	7		8189.851	3687_2_ – $11877_{1}^{{}^{\circ}}$
12188.866	1		8201.964	$18053_{4}^{{}^{\circ}}$ – 26255_4_
12182.019	3		8206.574	9804_5_ – $18011_{0}^{{}^{\circ}}$
12178.456	2		8208.975	$24084_{6}^{{}^{\circ}}$ – 32293_5_
12171.815	4		8213.454	4961_4_ – $13175_{4}^{{}^{\circ}}$
12167.821	3		8216.150	11601_1_ – $19817_{1}^{{}^{\circ}}$
12166.851	0		8216.805	$21539_{4}^{{}^{\circ}}$ – 29756_4_
12161.908	0		8220.144	18431_3_ – $26651_{2}^{{}^{\circ}}$
12143.705	0		8232.466	15970_3_ – $24202_{4}^{{}^{\circ}}$
12140.955	3		8234.331	$18809_{4}^{{}^{\circ}}$ – 27044_3_
12140.751	1		8234.469	$24561_{3}^{{}^{\circ}}$ – 32796_3_
12139.017	1		8235.645	8111_4_ – $16346_{4}^{{}^{\circ}}$
12132.020	3		8240.395	$20322_{0}^{{}^{\circ}}$ – 28562_4_
12129.427	5		8242.157	13297_4_ – $21539_{4}^{{}^{\circ}}$
12128.913	1		8242.506	$21890_{3}^{{}^{\circ}}$ – 30132_2_
12127.302	8		8243.601	0_2_ – $8243_{2}^{{}^{\circ}}$
12126.918	1		8243.862	$24307_{2}^{{}^{\circ}}$ – 32551_3_
12126.419	5		8244.201	$18011_{0}^{{}^{\circ}}$ – 26255_4_
12119.644	3		8248.810	9804_5_ – $18053_{4}^{{}^{\circ}}$
12117.950	0		8249.963	$22508_{2}^{{}^{\circ}}$ – 30758_2_
12113.062	0		8253.292	$21165_{3}^{{}^{\circ}}$ – 29418_2_
12109.655	0		8255.614	$24202_{4}^{{}^{\circ}}$ – 32458_4_
12109.434	3		8255.765	$20423_{1}^{{}^{\circ}}$ – 28679_2_
12109.313	1		8255.847	$23015_{0}^{{}^{\circ}}$ – 31271_5_
12099.785	2		8262.348	$24701_{0}^{{}^{\circ}}$ – 32963_5_
12083.777	0		8273.294	$21738_{2}^{{}^{\circ}}$ – 30011_3_
12082.221	2		8274.359	$23655_{4}^{{}^{\circ}}$ – 31929_3_
12075.537	1		8278.939	$20566_{4}^{{}^{\circ}}$ – 28845_4_
12074.667	2		8279.536	$19948_{4}^{{}^{\circ}}$ – 28227_4_
12066.559	0		8285.099	$23916_{4}^{{}^{\circ}}$ – 32202_5_
12059.895	0		8289.677	15970_3_ – $24259_{4}^{{}^{\circ}}$
12058.422	0		8290.690	$11241_{3}^{{}^{\circ}}$ – 19532_4_
12055.291	0		8292.843	$21668_{1}^{{}^{\circ}}$ – 29961_1_
12051.267	1		8295.612	$22248_{2}^{{}^{\circ}}$ – 30544_2_
12050.486	0		8296.150	18699_2_ – $26995_{3}^{{}^{\circ}}$
12038.242	1		8304.588	$19039_{2}^{{}^{\circ}}$ – 27343_3_
12037.639	4		8305.004	17398_3_ – $25703_{2}^{{}^{\circ}}$
12020.095	1		8317.125	12847_3_ – $21165_{3}^{{}^{\circ}}$
12018.718	7		8318.078	$16346_{4}^{{}^{\circ}}$ – 24664_3_
12018.054	4		8318.538	15863_2_ – $24182_{2}^{{}^{\circ}}$
12015.950	4		8319.994	$23306_{6}^{{}^{\circ}}$ – 31626_5_
12015.478	1		8320.321	19532_4_ – $27852_{0}^{{}^{\circ}}$
12007.313	0		8325.979	$23603_{2}^{{}^{\circ}}$ – 31929_3_
12005.953	1		8326.922	$18053_{4}^{{}^{\circ}}$ – 26380_5_
11986.392	0		8340.511	$23655_{4}^{{}^{\circ}}$ – 31995_4_
11980.135	1		8344.867	$22163_{4}^{{}^{\circ}}$ – 30508_5_
11976.025	1		8347.731	$20214_{3}^{{}^{\circ}}$ – 28562_4_
11974.134	1		8349.049	$24202_{4}^{{}^{\circ}}$ – 32551_3_
11961.578	1		8357.813	$23655_{4}^{{}^{\circ}}$ – 32012_4_
11956.759	0		8361.182	$22396_{1}^{{}^{\circ}}$ – 30758_2_
11955.885	0		8361.793	18699_2_ – $27061_{2}^{{}^{\circ}}$
11952.063	4		8364.467	$19227_{6}^{{}^{\circ}}$ – 27591_5_
11946.618	1		8368.279	$20566_{4}^{{}^{\circ}}$ – 28934_3_
11945.362	6		8369.159	$18011_{0}^{{}^{\circ}}$ – 26380_5_
11942.130	5		8371.424	$15618_{3}^{{}^{\circ}}$ – 23990_2_
11940.638	7		8372.470	2869_3_ – $11241_{3}^{{}^{\circ}}$
11927.809	0		8381.475	$18809_{4}^{{}^{\circ}}$ – 27191_5_
11916.586	3		8389.369	$22163_{4}^{{}^{\circ}}$ – 30552_4_
11910.840	0		8393.416	$22877_{0}^{{}^{\circ}}$ – 31271_5_
11909.862	1		8394.105	$19948_{4}^{{}^{\circ}}$ – 28342_5_
11904.795	2		8397.678	19273_2_ – $27670_{3}^{{}^{\circ}}$
11899.837	4		8401.177	13847_2_ – $22248_{2}^{{}^{\circ}}$
11894.942	3		8404.634	12847_3_ – $21252_{2}^{{}^{\circ}}$
11887.357	1		8409.997	$19948_{4}^{{}^{\circ}}$ – 28358_3_
11886.085	2		8410.897	$22141_{3}^{{}^{\circ}}$ – 30552_4_
11884.530	5		8411.997	11802_2_ – $20214_{3}^{{}^{\circ}}$
11882.776	2		8413.239	$15618_{3}^{{}^{\circ}}$ – 24032_4_
11878.611	2		8416.189	19532_4_ – $27948_{4}^{{}^{\circ}}$
11874.629	2		8419.011	$22338_{3}^{{}^{\circ}}$ – 30758_2_
11873.851	2		8419.563	$13175_{4}^{{}^{\circ}}$ – 21594_3_
11870.629	1		8421.848	$17501_{0}^{{}^{\circ}}$ – 25923_4_
11868.045	0		8423.682	15493_4_ – $23916_{4}^{{}^{\circ}}$
11864.247	7		8426.378	3687_2_ – $12114_{2}^{{}^{\circ}}$
11858.382	4		8430.546	$14206_{4}^{{}^{\circ}}$ – 22637_3_
11853.098	1		8434.304	13962_1_ – $22396_{1}^{{}^{\circ}}$
11848.313	1		8437.710	$23603_{2}^{{}^{\circ}}$ – 32041_2_
11840.529	0		8443.257	$23093_{2}^{{}^{\circ}}$ – 31537_3_
11839.689	1		8443.856	15863_2_ – $24307_{2}^{{}^{\circ}}$
11837.186	2		8445.642	$19588_{0}^{{}^{\circ}}$ – 28034_5_
11836.168	0		8446.368	18549_2_ – $26995_{3}^{{}^{\circ}}$
11834.938	1		8447.246	$16217_{2}^{{}^{\circ}}$ – 24664_3_
11831.941	0		8449.386	$22877_{0}^{{}^{\circ}}$ – 31326_4_
11829.730	4		8450.965	15970_3_ – $24421_{3}^{{}^{\circ}}$
11829.640	5		8451.029	13088_3_ – $21539_{4}^{{}^{\circ}}$
11827.583	4		8452.499	17073_1_ – $25526_{1}^{{}^{\circ}}$
11824.901	1		8454.416	$16217_{2}^{{}^{\circ}}$ – 24671_2_
11822.640	4		8456.033	$19817_{1}^{{}^{\circ}}$ – 28273_2_
11821.839	1		8456.606	$22508_{2}^{{}^{\circ}}$ – 30964_3_
11821.500	0		8456.848	7280_2_ – $15736_{1}^{{}^{\circ}}$
11818.546	1		8458.962	$20423_{1}^{{}^{\circ}}$ – 28882_2_
11811.926	2		8463.703	$19516_{2}^{{}^{\circ}}$ – 27980_3_
11811.055	0		8464.327	$20214_{3}^{{}^{\circ}}$ – 28679_2_
11804.617	6		8468.943	5563_1_ – $14032_{2}^{{}^{\circ}}$
11804.116	3		8469.303	$21903_{7}^{{}^{\circ}}$ – 30372_6_
11802.102	1		8470.748	$13175_{4}^{{}^{\circ}}$ – 21645_4_
11801.327	0		8471.304	$11241_{3}^{{}^{\circ}}$ – 19713_3_
11793.069	1		8477.236	$24259_{4}^{{}^{\circ}}$ – 32737_5_
11792.645	1		8477.541	$19503_{3}^{{}^{\circ}}$ – 27980_3_
11790.382	1		8479.168	$17411_{3}^{{}^{\circ}}$ – 25890_2_
11779.528	2		8486.981	16351_0_ – $24838_{1}^{{}^{\circ}}$
11775.163	4		8490.127	$10783_{2}^{{}^{\circ}}$ – 19273_2_
11772.698	1		8491.905	$23521_{3}^{{}^{\circ}}$ – 32012_4_
11761.537	1		8499.963	$20922_{2}^{{}^{\circ}}$ – 29422_1_
11746.174	1		8511.080	19273_2_ – $27784_{2}^{{}^{\circ}}$
11729.339	1		8523.296	$14465_{2}^{{}^{\circ}}$ – 22988_2_
11729.339	1		8523.296	$20322_{0}^{{}^{\circ}}$ – 28845_4_
11723.279	2		8527.702	$19039_{2}^{{}^{\circ}}$ – 27566_2_
11722.487	1		8528.278	16351_0_ – $24880_{1}^{{}^{\circ}}$
11712.220	3		8535.754	$17354_{1}^{{}^{\circ}}$ – 25890_2_
11709.471	0		8537.758	$24561_{3}^{{}^{\circ}}$ – 33099_3_
11704.645	1		8541.278	$23916_{4}^{{}^{\circ}}$ – 32458_4_
11703.457	7		8542.145	$15490_{0}^{{}^{\circ}}$ – 24032_4_
11698.831	3		8545.523	13962_1_ – $22508_{2}^{{}^{\circ}}$
11695.263	2		8548.130	17959_4_ – $26508_{3}^{{}^{\circ}}$
11694.285	3		8548.845	$21738_{2}^{{}^{\circ}}$ – 30286_1_
11694.000	2		8549.053	13847_2_ – $22396_{1}^{{}^{\circ}}$
11690.334	0		8551.734	$22877_{1}^{{}^{\circ}}$ – 31429_1_
11682.909	1		8557.169	15863_2_ – $24421_{3}^{{}^{\circ}}$
11678.305	1		8560.543	18699_2_ – $27260_{3}^{{}^{\circ}}$
11673.465	1		8564.092	18431_3_ – $26995_{3}^{{}^{\circ}}$
11667.638	5		8568.369	$16346_{4}^{{}^{\circ}}$ – 24915_3_
11661.167	3		8573.124	$19039_{2}^{{}^{\circ}}$ – 27612_3_
11657.259	1		8575.998	$22669_{3}^{{}^{\circ}}$ – 31245_2_
11653.134	1		8579.034	$24202_{4}^{{}^{\circ}}$ – 32781_4_
11648.509	1		8582.440	$21252_{2}^{{}^{\circ}}$ – 29835_3_
11641.195	1		8587.832	17166_5_ – $25753_{0}^{{}^{\circ}}$
11636.590	2		8591.231	$18930_{3}^{{}^{\circ}}$ – 27521_4_
11636.496	1		8591.300	$21165_{3}^{{}^{\circ}}$ – 29756_4_
11636.155	3		8591.552	15970_3_ – $24561_{3}^{{}^{\circ}}$
11634.601	4		8592.699	13297_4_ – $21890_{3}^{{}^{\circ}}$
11630.479	0		8595.745	$22669_{3}^{{}^{\circ}}$ – 31265_3_
11618.190	3		8604.837	13297_4_ – $21902_{4}^{{}^{\circ}}$
11617.462	5		8605.376	$14032_{2}^{{}^{\circ}}$ – 22637_3_
11615.211	1		8607.044	$21077_{0}^{{}^{\circ}}$ – 29684_5_
11609.914	2		8610.971	8800_4_ – $17411_{3}^{{}^{\circ}}$
11606.246	1		8613.692	$24182_{2}^{{}^{\circ}}$ – 32796_3_
11605.473	3		8614.266	$19948_{4}^{{}^{\circ}}$ – 28562_4_
11602.596	0		8616.402	$22141_{3}^{{}^{\circ}}$ – 30758_2_
11600.761	0		8617.765	18699_2_ – $27317_{3}^{{}^{\circ}}$
11600.523	0		8617.942	$21668_{1}^{{}^{\circ}}$ – 30286_1_
11597.002	1		8620.558	11802_2_ – $20423_{1}^{{}^{\circ}}$
11595.985	6		8621.314	$16783_{4}^{{}^{\circ}}$ – 25405_4_
11589.953	4		8625.801	16554_6_ – $25180_{7}^{{}^{\circ}}$
11584.866	0		8629.589	17073_1_ – $25703_{2}^{{}^{\circ}}$
11584.675	1		8629.731	18431_3_ – $27061_{2}^{{}^{\circ}}$
11583.465	0		8630.633	$20214_{3}^{{}^{\circ}}$ – 28845_4_
11577.991	1		8634.713	$23916_{4}^{{}^{\circ}}$ – 32551_3_
11575.518	4		8636.558	$18930_{3}^{{}^{\circ}}$ – 27566_2_
11573.652	1		8637.950	17398_3_ – $26036_{3}^{{}^{\circ}}$
11571.485	5		8639.568	$19588_{0}^{{}^{\circ}}$ – 28227_4_
11558.214	3		8649.488	13088_3_ – $21738_{2}^{{}^{\circ}}$
11557.337	3		8650.144	17398_3_ – $26048_{4}^{{}^{\circ}}$
11557.225	0		8650.228	$21902_{4}^{{}^{\circ}}$ – 30552_4_
11554.767	0		8652.068	$24084_{6}^{{}^{\circ}}$ – 32737_5_
11551.616	0		8654.428	$21890_{3}^{{}^{\circ}}$ – 30544_2_
11543.824	2		8660.270	13847_2_ – $22508_{2}^{{}^{\circ}}$
11541.028	0		8662.368	$21890_{3}^{{}^{\circ}}$ – 30552_4_
11536.071	2		8666.090	$17224_{2}^{{}^{\circ}}$ – 25890_2_
11530.938	3		8669.948	$21165_{3}^{{}^{\circ}}$ – 29835_3_
11526.460	4		8673.316	14204_5_ – $22877_{0}^{{}^{\circ}}$
11517.393	1		8680.144	$23113_{4}^{{}^{\circ}}$ – 31793_4_
11516.456	5		8680.850	5563_1_ – $14243_{1}^{{}^{\circ}}$
11514.959	4		8681.979	$18930_{3}^{{}^{\circ}}$ – 27612_3_
11512.063	3		8684.163	5563_1_ – $14247_{0}^{{}^{\circ}}$
11511.566	6		8684.538	$20867_{7}^{{}^{\circ}}$ – 29552_6_
11511.286	1		8684.749	$23609_{0}^{{}^{\circ}}$ – 32293_5_
11510.028	3		8685.698	$18809_{4}^{{}^{\circ}}$ – 27495_4_
11506.079	1		8688.679	$24274_{0}^{{}^{\circ}}$ – 32963_5_
11502.185	3		8691.621	12847_3_ – $21539_{4}^{{}^{\circ}}$
11501.478	4		8692.155	$13945_{3}^{{}^{\circ}}$ – 22637_3_
11494.068	2		8697.759	15863_2_ – $24561_{3}^{{}^{\circ}}$
11489.885	5		8700.925	8800_4_ – $17501_{0}^{{}^{\circ}}$
11478.781	1		8709.342	15493_4_ – $24202_{4}^{{}^{\circ}}$
11476.910	0		8710.762	18549_2_ – $27260_{3}^{{}^{\circ}}$
11476.074	1		8711.396	17166_5_ – $25877_{4}^{{}^{\circ}}$
11475.757	3		8711.637	$18809_{4}^{{}^{\circ}}$ – 27521_4_
11472.823	2		8713.865	$19817_{1}^{{}^{\circ}}$ – 28531_2_
11471.073	0		8715.194	7502_3_ – $16217_{2}^{{}^{\circ}}$
11465.039	5		8719.781	11802_2_ – $20522_{2}^{{}^{\circ}}$
11464.786	2		8719.973	$20214_{3}^{{}^{\circ}}$ – 28934_3_
11458.461	1		8724.787	$19503_{3}^{{}^{\circ}}$ – 28227_4_
11455.545	Id		8727.008	$18069_{3}^{{}^{\circ}}$ – 26796_3_
11444.419	2		8735.492	17073_1_ – $25809_{1}^{{}^{\circ}}$
11442.751	1		8736.765	$14465_{2}^{{}^{\circ}}$ – 23201_3_
11432.593	4		8744.528	$14243_{1}^{{}^{\circ}}$ – 22988_2_
11429.704	6		8746.738	$10526_{3}^{{}^{\circ}}$ – 19273_2_
11427.901	1		8748.118	18699_2_ – $27447_{2}^{{}^{\circ}}$
11426.433	0		8749.242	$22877_{0}^{{}^{\circ}}$ – 31626_5_
11420.044	1		8754.137	$19588_{0}^{{}^{\circ}}$ – 28342_5_
11419.693	1		8754.406	$17501_{0}^{{}^{\circ}}$ – 26255_4_
11417.309	3		8756.234	$19516_{2}^{{}^{\circ}}$ – 28273_2_
11415.542	1		8757.589	$22508_{2}^{{}^{\circ}}$ – 31265_3_
11411.321	1		8760.829	$24202_{4}^{{}^{\circ}}$ – 32963_5_
11402.009	0		8767.984	18549_2_ – $27317_{3}^{{}^{\circ}}$
11386.235	5		8780.130	$15618_{3}^{{}^{\circ}}$ – 24399_3_
11384.471	3		8781.491	15493_4_ – $24274_{0}^{{}^{\circ}}$
11383.920	3		8781.916	$18809_{4}^{{}^{\circ}}$ – 27591_5_
11371.299	1		8791.663	$24307_{2}^{{}^{\circ}}$ – 33099_3_
11369.091	2		8793.370	$24274_{0}^{{}^{\circ}}$ – 33068_6_
11366.315	2		8795.518	$14481_{6}^{{}^{\circ}}$ – 23277_5_
11358.564	1		8801.520	$22163_{4}^{{}^{\circ}}$ – 30964_3_
11358.499	3		8801.570	13088_3_ – $21890_{3}^{{}^{\circ}}$
11357.450	0		8802.383	$18809_{4}^{{}^{\circ}}$ – 27612_3_
11356.910	0		8802.802	$22338_{3}^{{}^{\circ}}$ – 31141_3_
11356.608	1		8803.036	$23655_{4}^{{}^{\circ}}$ – 32458_4_
11354.715	8		8804.503	6362_2_ – $15166_{3}^{{}^{\circ}}$
11352.128	2		8806.510	$21738_{2}^{{}^{\circ}}$ – 30544_2_
11351.924	6		8806.668	$19227_{6}^{{}^{\circ}}$ – 28034_5_
11346.494	1		8810.883	14204_5_ – $23015_{0}^{{}^{\circ}}$
11343.709	4		8813.046	$11241_{3}^{{}^{\circ}}$ – 20054_2_
11342.857	3		8813.708	13088_3_ – $21902_{4}^{{}^{\circ}}$
11335.713	0		8819.262	$20322_{0}^{{}^{\circ}}$ – 29141_5_
11332.045	4		8822.117	13847_2_ – $22669_{3}^{{}^{\circ}}$
11331.384	1		8822.632	14226_0_ – $23049_{1}^{{}^{\circ}}$
11326.973	3		8826.067	$14206_{4}^{{}^{\circ}}$ – 23032_4_
11323.876	0		8828.481	18431_3_ – $27260_{3}^{{}^{\circ}}$
11321.659	2		8830.210	$8243_{2}^{{}^{\circ}}$ – 17073_1_
11321.249	2		8830.530	17959_4_ – $26790_{4}^{{}^{\circ}}$
11307.314	3		8841.412	$19516_{2}^{{}^{\circ}}$ – 28358_3_
11303.787	5		8844.171	13297_4_ – $22141_{3}^{{}^{\circ}}$
11303.544	4		8844.361	7502_3_ – $16346_{4}^{{}^{\circ}}$
11303.544	4		8844.361	$17411_{3}^{{}^{\circ}}$ – 26255_4_
11301.136	1		8846.246	$21165_{3}^{{}^{\circ}}$ – 30011_3_
11297.608	1		8849.008	$23609_{0}^{{}^{\circ}}$ – 32458_4_
11289.645	2		8855.250	$19503_{3}^{{}^{\circ}}$ – 28358_3_
11277.612	0		8864.698	$23916_{4}^{{}^{\circ}}$ – 32781_4_
11276.818	4		8865.322	$15166_{3}^{{}^{\circ}}$ – 24032_4_
11276.338	2		8865.700	13297_4_ – $22163_{4}^{{}^{\circ}}$
11273.574	4		8867.873	$21890_{3}^{{}^{\circ}}$ – 30758_2_
11260.143	1		8878.451	$24084_{6}^{{}^{\circ}}$ – 32963_5_
11258.986	4		8879.363	$17501_{0}^{{}^{\circ}}$ – 26380_5_
11258.130	3		8880.038	$21252_{2}^{{}^{\circ}}$ – 30132_2_
11255.513	0		8882.103	$23113_{4}^{{}^{\circ}}$ – 31995_4_
11255.092	3		8882.435	17166_5_ – $26048_{4}^{{}^{\circ}}$
11249.018	0		8887.231	$17224_{2}^{{}^{\circ}}$ – 26111_1_
11246.685	1		8889.075	$20214_{3}^{{}^{\circ}}$ – 29104_4_
11245.412	2		8890.081	12847_3_ – $21738_{2}^{{}^{\circ}}$
11238.982	2		8895.167	$23306_{6}^{{}^{\circ}}$ – 32202_5_
11238.344	0		8895.672	$20522_{2}^{{}^{\circ}}$ – 29418_2_
11236.858	1		8896.849	$24202_{4}^{{}^{\circ}}$ – 33099_3_
11233.671	2		8899.373	$20522_{2}^{{}^{\circ}}$ – 29422_1_
11230.255	9		8902.080	5563_1_ – $14465_{2}^{{}^{\circ}}$
11225.532	3		8905.825	15863_2_ – $24769_{3}^{{}^{\circ}}$
11221.176	1		8909.282	14204_5_ – $23113_{4}^{{}^{\circ}}$
11215.651	2		8913.671	$20737_{1}^{{}^{\circ}}$ – 29650_2_
11212.583	1		8916.110	$22877_{0}^{{}^{\circ}}$ – 31793_4_
11211.484	0		8916.984	$24182_{2}^{{}^{\circ}}$ – 33099_3_
11205.579	4		8921.683	11601_1_ – $20522_{2}^{{}^{\circ}}$
11203.831	1		8923.075	$13175_{4}^{{}^{\circ}}$ – 22098_4_
11195.293	1		8929.880	$10783_{2}^{{}^{\circ}}$ – 19713_3_
11189.689	2		8934.352	11802_2_ – $20737_{1}^{{}^{\circ}}$
11185.925	5		8937.359	7280_2_ – $16217_{2}^{{}^{\circ}}$
11184.628	0		8938.395	$22855_{3}^{{}^{\circ}}$ – 31793_4_
11179.105	3		8942.811	11601_1_ – $20543_{0}^{{}^{\circ}}$
11171.377	1		8948.997	$17847_{2}^{{}^{\circ}}$ – 26796_3_
11162.099	3		8956.436	$14032_{2}^{{}^{\circ}}$ – 22988_2_
11157.560	1		8960.079	$18011_{0}^{{}^{\circ}}$ – 26971_4_
11151.802	0		8964.706	17398_3_ – $26363_{2}^{{}^{\circ}}$
11148.272	0		8967.544	$21165_{3}^{{}^{\circ}}$ – 30132_2_
11147.197	0		8968.409	$21539_{4}^{{}^{\circ}}$ – 30508_5_
11139.881	3		8974.299	$19588_{0}^{{}^{\circ}}$ – 28562_4_
11138.971	1		8975.032	15863_2_ – $24838_{1}^{{}^{\circ}}$
11138.821	4		8975.153	$18069_{3}^{{}^{\circ}}$ – 27044_3_
11134.469	1		8978.661	$22163_{4}^{{}^{\circ}}$ – 31141_3_
11128.287	5		8983.649	4961_4_ – $13945_{3}^{{}^{\circ}}$
11125.541	4		8985.866	$18011_{0}^{{}^{\circ}}$ – 26997_6_
11124.745	2		8986.509	$19948_{4}^{{}^{\circ}}$ – 28934_3_
11124.026	3		8987.090	$23306_{6}^{{}^{\circ}}$ – 32293_5_
11114.372	0		8994.896	$20423_{1}^{{}^{\circ}}$ – 29418_2_
11114.157	3		8995.070	$14206_{4}^{{}^{\circ}}$ – 23201_3_
11101.804	5		9005.079	9804_5_ – $18809_{4}^{{}^{\circ}}$
11098.788	2		9007.526	13847_2_ – $22855_{3}^{{}^{\circ}}$
11094.494	2		9011.012	15970_3_ – $24981_{3}^{{}^{\circ}}$
11092.159	1		9012.909	$21539_{4}^{{}^{\circ}}$ – 30552_4_
11090.735	3		9014.066	$19516_{2}^{{}^{\circ}}$ – 28531_2_
11073.735	3		9027.904	$19503_{3}^{{}^{\circ}}$ – 28531_2_
11071.478	1		9029.745	13847_2_ – $22877_{1}^{{}^{\circ}}$
11057.009	4		9041.561	13297_4_ – $22338_{3}^{{}^{\circ}}$
11054.989	4		9043.213	$13945_{3}^{{}^{\circ}}$ – 22988_2_
11051.898	7		9045.742	$15618_{3}^{{}^{\circ}}$ – 24664_3_
11050.978	1		9046.495	$23916_{4}^{{}^{\circ}}$ – 32963_5_
11046.224	4		9050.389	$18930_{3}^{{}^{\circ}}$ – 27980_3_
11043.146	3		9052.911	$15618_{3}^{{}^{\circ}}$ – 24671_2_
11042.986	2		9053.042	13088_3_ – $22141_{3}^{{}^{\circ}}$
11036.319	4		9058.511	$16346_{4}^{{}^{\circ}}$ – 25405_4_
11035.091	0		9059.519	$19503_{3}^{{}^{\circ}}$ – 28562_4_
11031.608	1		9062.380	$21902_{4}^{{}^{\circ}}$ – 30964_3_
11028.089	1		9065.271	$19817_{1}^{{}^{\circ}}$ – 28882_2_
11021.768	3		9070.470	$14206_{4}^{{}^{\circ}}$ – 23277_5_
11016.787	4		9074.571	13088_3_ – $22163_{4}^{{}^{\circ}}$
11001.800	3		9086.933	13962_1_ – $23049_{1}^{{}^{\circ}}$
11000.900	1		9087.676	$13945_{3}^{{}^{\circ}}$ – 23032_4_
10999.236	0		9089.051	$21668_{1}^{{}^{\circ}}$ – 30758_2_
10983.633	5		9101.963	13297_4_ – $22399_{0}^{{}^{\circ}}$
10982.982	3		9102.502	$22163_{4}^{{}^{\circ}}$ – 31265_3_
10982.910	4		9102.562	14204_5_ – $23306_{6}^{{}^{\circ}}$
10976.518	0		9107.863	$22163_{4}^{{}^{\circ}}$ – 31271_5_
10974.389	1		9109.630	17398_3_ – $26508_{3}^{{}^{\circ}}$
10965.252	1		9117.220	15863_2_ – $24981_{3}^{{}^{\circ}}$
10964.829	1		9117.572	$20566_{4}^{{}^{\circ}}$ – 29684_5_
10963.975	1		9118.282	$10414_{4}^{{}^{\circ}}$ – 19532_4_
10962.885	5		9119.189	11802_2_ – $20922_{2}^{{}^{\circ}}$
10956.004	1		9124.916	18549_2_ – $27674_{2}^{{}^{\circ}}$
10948.154	4		9131.459	13962_1_ – $23093_{2}^{{}^{\circ}}$
10942.432	3		9136.234	16554_6_ – $25690_{0}^{{}^{\circ}}$
10942.432	3		9136.234	11601_1_ – $20737_{1}^{{}^{\circ}}$
10938.909	1		9139.176	$16783_{4}^{{}^{\circ}}$ – 25923_4_
10923.725	2		9151.880	$21738_{2}^{{}^{\circ}}$ – 30889_1_
10913.581	3		9160.386	13088_3_ – $22248_{2}^{{}^{\circ}}$
10911.329	2		9162.277	$19516_{2}^{{}^{\circ}}$ – 28679_2_
10901.193	4		9170.796	$18809_{4}^{{}^{\circ}}$ – 27980_3_
10894.875	1		9176.114	$19503_{3}^{{}^{\circ}}$ – 28679_2_
10890.287	4		9179.980	$18011_{0}^{{}^{\circ}}$ – 27191_5_
10887.285	0		9182.511	$23916_{4}^{{}^{\circ}}$ – 33099_3_
10883.902	1		9185.365	$22141_{3}^{{}^{\circ}}$ – 31326_4_
10883.233	0		9185.930	$22855_{3}^{{}^{\circ}}$ – 32041_2_
10882.569	1		9186.490	$10526_{3}^{{}^{\circ}}$ – 19713_3_
10882.153	0		9186.842	$23015_{0}^{{}^{\circ}}$ – 32202_5_
10869.967	4		9197.141	$17847_{2}^{{}^{\circ}}$ – 27044_3_
10866.949	1		9199.695	16554_6_ – $25753_{0}^{{}^{\circ}}$
10864.601	2		9201.683	13847_2_ – $23049_{1}^{{}^{\circ}}$
10862.508	4		9203.456	$20214_{3}^{{}^{\circ}}$ – 29418_2_
10857.355	1		9207.824	15493_4_ – $24701_{0}^{{}^{\circ}}$
10853.458	3		9211.130	8800_4_ – $18011_{0}^{{}^{\circ}}$
10844.405	4		9218.820	17166_5_ – $26384_{4}^{{}^{\circ}}$
10837.713	0		9224.512	$20737_{1}^{{}^{\circ}}$ – 29961_1_
10826.499	0		9234.067	$19039_{2}^{{}^{\circ}}$ – 28273_2_
10813.573	4		9245.105	2869_3_ – $12114_{2}^{{}^{\circ}}$
10813.395	6		9245.257	4961_4_ – $14206_{4}^{{}^{\circ}}$
10812.281	5		9246.209	13847_2_ – $23093_{2}^{{}^{\circ}}$
10807.344	3		9250.433	13088_3_ – $22338_{3}^{{}^{\circ}}$
10803.918	4		9253.367	8800_4_ – $18053_{4}^{{}^{\circ}}$
10802.535	1		9254.551	14226_0_ – $23481_{1}^{{}^{\circ}}$
10800.157	4		9256.589	6362_2_ – $15618_{3}^{{}^{\circ}}$
10800.053	5		9256.678	$13945_{3}^{{}^{\circ}}$ – 23201_3_
10788.554	1		9266.544	$22508_{2}^{{}^{\circ}}$ – 31774_3_
10786.371	3		9268.420	$20566_{4}^{{}^{\circ}}$ – 29835_3_
10785.912	4		9268.814	8800_4_ – $18069_{3}^{{}^{\circ}}$
10779.100	2		9274.672	$18069_{3}^{{}^{\circ}}$ – 27343_3_
10778.635	1		9275.072	$23521_{3}^{{}^{\circ}}$ – 32796_3_
10776.984	4		9276.493	15493_4_ – $24769_{3}^{{}^{\circ}}$
10774.344	1		9278.766	$23015_{0}^{{}^{\circ}}$ – 32293_5_
10759.049	1		9291.956	$21252_{2}^{{}^{\circ}}$ – 30544_2_
10754.878	0		9295.560	$21077_{0}^{{}^{\circ}}$ – 30372_6_
10754.328	4		9296.035	$15618_{3}^{{}^{\circ}}$ – 24915_3_
10752.475	2		9297.637	$18930_{3}^{{}^{\circ}}$ – 28227_4_
10749.434	3d		9300.268	17959_4_ – $27260_{3}^{{}^{\circ}}$
10744.370	2		9304.651	$14465_{2}^{{}^{\circ}}$ – 23769_1_
10732.245	5		9315.163	12847_3_ – $22163_{4}^{{}^{\circ}}$
10726.926	8		9319.782	2558_0_ – $11877_{1}^{{}^{\circ}}$
10725.418	6		9321.092	11601_1_ – $20922_{2}^{{}^{\circ}}$
10720.051	0		9325.759	$22669_{3}^{{}^{\circ}}$ – 31995_4_
10707.531	1		9336.663	15970_3_ – $25306_{2}^{{}^{\circ}}$
10700.359	1		9342.921	$18930_{3}^{{}^{\circ}}$ – 28273_2_
10700.194	0		9343.065	$22669_{3}^{{}^{\circ}}$ – 32012_4_
10690.175	1		9351.822	15970_3_ – $25321_{3}^{{}^{\circ}}$
10678.631	1		9361.931	$20322_{0}^{{}^{\circ}}$ – 29684_5_
10678.369	3		9362.161	11802_2_ – $21165_{3}^{{}^{\circ}}$
10668.486	2		9370.834	$7795_{4}^{{}^{\circ}}$ – 17166_5_
10666.640	0		9372.456	13297_4_ – $22669_{3}^{{}^{\circ}}$
10664.767	0		9374.102	$22163_{4}^{{}^{\circ}}$ – 31537_3_
10664.234	5		9374.570	6362_2_ – $15736_{1}^{{}^{\circ}}$
10663.178	1		9375.499	$21890_{3}^{{}^{\circ}}$ – 31265_3_
10651.842	0		9385.476	15970_3_ – $25355_{4}^{{}^{\circ}}$
10649.651	1		9387.407	$21165_{3}^{{}^{\circ}}$ – 30552_4_
10646.516	2		9390.171	8111_4_ – $17501_{0}^{{}^{\circ}}$
10640.329	1		9395.631	$22141_{3}^{{}^{\circ}}$ – 31537_3_
10629.842	2		9404.901	14204_5_ – $23609_{0}^{{}^{\circ}}$
10615.008	4		9418.044	$18809_{4}^{{}^{\circ}}$ – 28227_4_
10613.388	4		9419.481	13088_3_ – $22508_{2}^{{}^{\circ}}$
10609.955	1		9422.529	9804_5_ – $19227_{6}^{{}^{\circ}}$
10607.108	1		9425.058	$21539_{4}^{{}^{\circ}}$ – 30964_3_
10605.464	4		9426.519	$18069_{3}^{{}^{\circ}}$ – 27495_4_
10601.345	2		9430.182	$23306_{6}^{{}^{\circ}}$ – 32737_5_
10600.597	1		9430.847	$21077_{0}^{{}^{\circ}}$ – 30508_5_
10596.909	1		9434.129	$20322_{0}^{{}^{\circ}}$ – 29756_4_
10595.264	1		9435.594	$22338_{3}^{{}^{\circ}}$ – 31774_3_
10594.780	1		9436.025	$20214_{3}^{{}^{\circ}}$ – 29650_2_
10592.495	0		9438.061	$23113_{4}^{{}^{\circ}}$ – 32551_3_
10591.350	2		9439.081	$20522_{2}^{{}^{\circ}}$ – 29961_1_
10587.100	3		9442.870	15863_2_ – $25306_{2}^{{}^{\circ}}$
10586.930	1		9443.022	$23015_{0}^{{}^{\circ}}$ – 32458_4_
10585.524	0		9444.276	$23655_{4}^{{}^{\circ}}$ – 33099_3_
10585.028	2		9444.718	$20566_{4}^{{}^{\circ}}$ – 30011_3_
10581.711	1		9447.679	13962_1_ – $23410_{0}^{{}^{\circ}}$
10579.481	1		9449.670	11802_2_ – $21252_{2}^{{}^{\circ}}$
10578.136	1		9450.872	14204_5_ – $23655_{4}^{{}^{\circ}}$
10576.362	2		9452.457	$18069_{3}^{{}^{\circ}}$ – 27521_4_
10570.129	0		9458.031	15863_2_ – $25321_{3}^{{}^{\circ}}$
10569.146	0		9458.911	$23609_{0}^{{}^{\circ}}$ – 33068_6_
10567.177	1		9460.673	$12114_{2}^{{}^{\circ}}$ – 21575_2_
10565.306	6		9462.349	$13175_{4}^{{}^{\circ}}$ – 22637_3_
10559.107	0		9467.904	$18053_{4}^{{}^{\circ}}$ – 27521_4_
10556.454	7		9470.283	$17501_{0}^{{}^{\circ}}$ – 26971_4_
10554.837	4		9471.734	$16783_{4}^{{}^{\circ}}$ – 26255_4_
10553.890	1		9472.584	15970_3_ – $25442_{3}^{{}^{\circ}}$
10545.902	1		9479.759	17398_3_ – $26878_{3}^{{}^{\circ}}$
10545.290	2		9480.309	$12114_{2}^{{}^{\circ}}$ – 21594_3_
10540.958	5		9484.205	$18011_{0}^{{}^{\circ}}$ – 27495_4_
10536.866	2		9487.888	15493_4_ – $24981_{3}^{{}^{\circ}}$
10533.385	3		9491.024	12847_3_ – $22338_{3}^{{}^{\circ}}$
10527.789	5		9496.069	$17501_{0}^{{}^{\circ}}$ – 26997_6_
10527.134	1		9496.660	$17847_{2}^{{}^{\circ}}$ – 27343_3_
10518.190	2		9504.735	$20867_{7}^{{}^{\circ}}$ – 30372_6_
10512.209	5		9510.143	$18011_{0}^{{}^{\circ}}$ – 27521_4_
10502.592	4		9518.851	13962_1_ – $23481_{1}^{{}^{\circ}}$
10498.496	5		9522.565	$14247_{0}^{{}^{\circ}}$ – 23769_1_
10495.610	1		9525.183	$14465_{2}^{{}^{\circ}}$ – 23990_2_
10494.843	6		9525.879	$14243_{1}^{{}^{\circ}}$ – 23769_1_
10492.255	6		9528.229	$10526_{3}^{{}^{\circ}}$ – 20054_2_
10487.427	1		9532.615	$18809_{4}^{{}^{\circ}}$ – 28342_5_
10459.723	4		9557.864	13297_4_ – $22855_{3}^{{}^{\circ}}$
10469.974	1		9548.506	$18809_{4}^{{}^{\circ}}$ – 28358_3_
10477.700	1		9541.465	$20214_{3}^{{}^{\circ}}$ – 29756_4_
10481.173	0		9538.303	$20423_{1}^{{}^{\circ}}$ – 29961_1_
10450.448	3		9566.346	$19986_{6}^{{}^{\circ}}$ – 29552_6_
10439.505	3		9576.374	$16346_{4}^{{}^{\circ}}$ – 25923_4_
10436.870	2		9578.792	15863_2_ – $25442_{3}^{{}^{\circ}}$
10435.094	3		9580.422	$18011_{0}^{{}^{\circ}}$ – 27591_5_
10434.109	1		9581.326	13088_3_ – $22669_{3}^{{}^{\circ}}$
10424.126	1		9590.502	$22338_{3}^{{}^{\circ}}$ – 31929_3_
10421.511	0		9592.909	$21165_{3}^{{}^{\circ}}$ – 30758_2_
10417.881	1		9596.251	$22399_{0}^{{}^{\circ}}$ – 31995_4_
10412.996	4		9600.753	$18930_{3}^{{}^{\circ}}$ – 28531_2_
10412.507	0		9601.204	$19817_{1}^{{}^{\circ}}$ – 29418_2_
10410.424	2		9603.125	$7795_{4}^{{}^{\circ}}$ – 17398_3_
10408.497	1		9604.903	$19817_{1}^{{}^{\circ}}$ – 29422_1_
10403.056	1		9609.927	$20522_{2}^{{}^{\circ}}$ – 30132_2_
10401.401	1		9611.456	$22163_{4}^{{}^{\circ}}$ – 31774_3_
10392.041	0		9620.113	$20214_{3}^{{}^{\circ}}$ – 29835_3_
10387.498	1		9624.320	17166_5_ – $26790_{4}^{{}^{\circ}}$
10378.821	1		9632.366	$18930_{3}^{{}^{\circ}}$ – 28562_4_
10378.146	1		9632.993	$17411_{3}^{{}^{\circ}}$ – 27044_3_
10377.490	2		9633.601	13847_2_ – $23481_{1}^{{}^{\circ}}$
10376.812	0		9634.231	$20214_{3}^{{}^{\circ}}$ – 29849_4_
10376.020	1		9634.966	$21902_{4}^{{}^{\circ}}$ – 31537_3_
10369.528	4		9640.998	13962_1_ – $23603_{2}^{{}^{\circ}}$
10362.966	1		9647.103	$21890_{3}^{{}^{\circ}}$ – 31537_3_
10358.167	3		9651.573	11601_1_ – $21252_{2}^{{}^{\circ}}$
10357.615	0		9652.087	$22141_{3}^{{}^{\circ}}$ – 31793_4_
10350.334	1		9658.877	$18614_{1}^{{}^{\circ}}$ – 28273_2_
10349.051	5		9660.074	12847_3_ – $22508_{2}^{{}^{\circ}}$
10346.540	3		9662.419	15863_2_ – $25526_{1}^{{}^{\circ}}$
10345.899	0		9663.017	17398_3_ – $27061_{2}^{{}^{\circ}}$
10334.926	3		9673.277	13847_2_ – $23521_{3}^{{}^{\circ}}$
10316.894	4		9690.184	$17501_{0}^{{}^{\circ}}$ – 27191_5_
10314.894	2		9692.063	$20322_{0}^{{}^{\circ}}$ – 30014_4_
10308.549	5		9698.028	$19986_{6}^{{}^{\circ}}$ – 29684_5_
10304.082	2		9702.233	$22338_{3}^{{}^{\circ}}$ – 32041_2_
10294.714	0		9711.062	17959_4_ – $27670_{3}^{{}^{\circ}}$
10293.052	4		9712.630	14204_5_ – $23916_{4}^{{}^{\circ}}$
10285.485	0		9719.775	$17847_{2}^{{}^{\circ}}$ – 27566_2_
10283.118	4		9722.012	7502_3_ – $17224_{2}^{{}^{\circ}}$
10278.860	1		9726.040	$21539_{4}^{{}^{\circ}}$ – 31265_3_
10275.694	0		9729.036	$20922_{2}^{{}^{\circ}}$ – 30651_3_
10271.191	2		9733.302	15970_3_ – $25703_{2}^{{}^{\circ}}$
10268.554	1		9735.801	$19948_{4}^{{}^{\circ}}$ – 29684_5_
10257.374	5		9746.413	$14243_{1}^{{}^{\circ}}$ – 23990_2_
10255.580	2		9748.118	$15166_{3}^{{}^{\circ}}$ – 24915_3_
10250.684	4		9752.773	$18809_{4}^{{}^{\circ}}$ – 28562_4_
10247.561	4		9755.746	13847_2_ – $23603_{2}^{{}^{\circ}}$
10241.779	2		9761.253	$23306_{6}^{{}^{\circ}}$ – 33068_6_
10236.031	4		9766.735	13088_3_ – $22855_{3}^{{}^{\circ}}$
10223.662	3		9778.551	13962_1_ – $23741_{1}^{{}^{\circ}}$
10218.434	5		9783.554	9804_5_ – $19588_{0}^{{}^{\circ}}$
10214.442	1		9787.377	$21539_{4}^{{}^{\circ}}$ – 31326_4_
10211.538	2		9790.161	13962_1_ – $23752_{2}^{{}^{\circ}}$
10205.020	1		9796.414	$20214_{3}^{{}^{\circ}}$ – 30011_3_
10202.133	1		9799.186	$21738_{2}^{{}^{\circ}}$ – 31537_3_
10193.720	0		9807.273	$20737_{1}^{{}^{\circ}}$ – 30544_2_
10192.965	0		9808.000	$19948_{4}^{{}^{\circ}}$ – 29756_4_
10184.539	2		9816.114	13297_4_ – $23113_{4}^{{}^{\circ}}$
10180.596	2		9819.916	$17224_{2}^{{}^{\circ}}$ – 27044_3_
10178.522	5		9821.917	12847_3_ – $22669_{3}^{{}^{\circ}}$
10175.012	4		9825.305	$14206_{4}^{{}^{\circ}}$ – 24032_4_
10160.324	0		9839.509	15863_2_ – $25703_{2}^{{}^{\circ}}$
10156.406	0		9843.305	$19039_{2}^{{}^{\circ}}$ – 28882_2_
10144.266	4		9855.084	6362_2_ – $16217_{2}^{{}^{\circ}}$
10141.399	6		9857.870	$13175_{4}^{{}^{\circ}}$ – 23032_4_
10140.434	4		9858.808	$20867_{7}^{{}^{\circ}}$ – 30726_7_
10137.390	2		9861.769	17398_3_ – $27260_{3}^{{}^{\circ}}$
10136.787	0		9862.355	15493_4_ – $25355_{4}^{{}^{\circ}}$
10131.371	2		9867.628	17398_3_ – $27266_{4}^{{}^{\circ}}$
10127.552	0		9871.349	$22141_{3}^{{}^{\circ}}$ – 32012_4_
10126.557	1		9872.318	$21902_{4}^{{}^{\circ}}$ – 31774_3_
10117.993	2		9880.675	14204_5_ – $24084_{6}^{{}^{\circ}}$
10111.877	1		9886.651	$19948_{4}^{{}^{\circ}}$ – 29835_3_
10107.000	0		9891.421	$21902_{4}^{{}^{\circ}}$ – 31793_4_
10105.549	0		9892.842	17959_4_ – $27852_{0}^{{}^{\circ}}$
10105.080	2		9893.301	13847_2_ – $23741_{1}^{{}^{\circ}}$
10104.307	1		9894.058	$16217_{2}^{{}^{\circ}}$ – 26111_1_
10102.579	2		9895.750	$19039_{2}^{{}^{\circ}}$ – 28934_3_
10098.624	1		9899.625	$22141_{3}^{{}^{\circ}}$ – 32041_2_
10097.463	1		9900.764	$19948_{4}^{{}^{\circ}}$ – 29849_4_
10089.136	7		9908.935	7502_3_ – $17411_{3}^{{}^{\circ}}$
10089.136	7		9908.935	$16346_{4}^{{}^{\circ}}$ – 26255_4_
10086.406	2		9911.617	$18069_{3}^{{}^{\circ}}$ – 27980_3_
10083.788	5		9914.190	$19227_{6}^{{}^{\circ}}$ – 29141_5_
10082.880	4		9915.083	$15490_{0}^{{}^{\circ}}$ – 25405_4_
10082.719	1		9915.242	$19503_{3}^{{}^{\circ}}$ – 29418_2_
10082.719	1		9915.242	$18930_{3}^{{}^{\circ}}$ – 28845_4_
10081.227	1		9916.709	$18614_{1}^{{}^{\circ}}$ – 28531_2_
10078.906	1		9918.993	17398_3_ – $27317_{3}^{{}^{\circ}}$
10065.187	1		9932.512	$17411_{3}^{{}^{\circ}}$ – 27343_3_
10056.214	2		9941.375	$20566_{4}^{{}^{\circ}}$ – 30508_5_
10054.960	1		9942.615	8111_4_ – $18053_{4}^{{}^{\circ}}$
10053.375	0		9944.182	7280_2_ – $17224_{2}^{{}^{\circ}}$
10048.041	3		9949.461	15493_4_ – $25442_{3}^{{}^{\circ}}$
10045.316	2		9952.160	$18930_{3}^{{}^{\circ}}$ – 28882_2_
10039.364	7		9958.060	8111_4_ – $18069_{3}^{{}^{\circ}}$
10039.101	2		9958.321	$14032_{2}^{{}^{\circ}}$ – 23990_2_
10033.226	2		9964.152	$19588_{0}^{{}^{\circ}}$ – 29552_6_
10029.545	1		9967.809	$20922_{0}^{{}^{\circ}}$ – 30889_1_
10011.398	3		9985.877	$20566_{4}^{{}^{\circ}}$ – 30552_4_
9999.614	0		9997.645	$21539_{4}^{{}^{\circ}}$ – 31537_3_
9998.963	2		9998.296	14204_5_ – $24202_{4}^{{}^{\circ}}$
9992.656	3		10004.607	$18930_{3}^{{}^{\circ}}$ – 28934_3_
9991.846	0		10005.418	13088_3_ – $23093_{2}^{{}^{\circ}}$
9989.939	2		10007.327	12847_3_ – $22855_{3}^{{}^{\circ}}$
9987.636	6		10009.635	8800_4_ – $18809_{4}^{{}^{\circ}}$
9985.052	5		10012.225	$16783_{4}^{{}^{\circ}}$ – 26796_3_
9974.693	3		10022.623	$18011_{0}^{{}^{\circ}}$ – 28034_5_
9970.467	4		10026.872	$13175_{4}^{{}^{\circ}}$ – 23201_3_
9963.495	3		10033.888	$16346_{4}^{{}^{\circ}}$ – 26380_5_
9958.059	1		10039.365	$21890_{3}^{{}^{\circ}}$ – 31929_3_
9952.375	3		10045.099	$13945_{3}^{{}^{\circ}}$ – 23990_2_
9948.171	1		10049.344	17398_3_ – $27447_{2}^{{}^{\circ}}$
9947.082	1		10050.444	$20322_{0}^{{}^{\circ}}$ – 30372_6_
9938.839	1		10058.779	$22399_{0}^{{}^{\circ}}$ – 32458_4_
9935.203	2		10062.461	$20867_{7}^{{}^{\circ}}$ – 30930_6_
9934.723	1		10062.947	$19948_{4}^{{}^{\circ}}$ – 30011_3_
9932.777	0		10064.918	$18614_{1}^{{}^{\circ}}$ – 28679_2_
9929.814	3		10067.922	11601_1_ – $21668_{1}^{{}^{\circ}}$
9927.326	2		10070.445	14204_5_ – $24274_{0}^{{}^{\circ}}$
9923.310	1		10074.520	18574_1_ – $28649_{1}^{{}^{\circ}}$
9919.448	0		10078.443	15970_3_ – $26048_{4}^{{}^{\circ}}$
9916.122	1		10081.823	15493_4_ – $25575_{4}^{{}^{\circ}}$
9913.628	1		10084.360	$17411_{3}^{{}^{\circ}}$ – 27495_4_
9912.205	2		10085.807	$22877_{0}^{{}^{\circ}}$ – 32963_5_
9911.116	4		10086.915	$13945_{3}^{{}^{\circ}}$ – 24032_4_
9910.837	2		10087.199	11802_2_ – $21890_{3}^{{}^{\circ}}$
9907.464	1q		10090.634	$17501_{0}^{{}^{\circ}}$ – 27591_5_
9906.948	1		10091.159	16554_6_ – $26645_{0}^{{}^{\circ}}$
9904.771	1		10093.377	$21902_{4}^{{}^{\circ}}$ – 31995_4_
9902.362	4		10095.833	$19588_{0}^{{}^{\circ}}$ – 29684_5_
9898.356	2		10099.918	17166_5_ – $27266_{4}^{{}^{\circ}}$
9896.050	3		10102.272	$13175_{4}^{{}^{\circ}}$ – 23277_5_
9873.821	3		10125.015	$18809_{4}^{{}^{\circ}}$ – 28934_3_
9872.635	1		10126.231	$22669_{3}^{{}^{\circ}}$ – 32796_3_
9871.998	2		10126.885	15970_3_ – $26096_{3}^{{}^{\circ}}$
9868.922	2		10130.041	8800_4_ – $18930_{3}^{{}^{\circ}}$
9867.890	2		10131.101	7280_2_ – $17411_{3}^{{}^{\circ}}$
9865.451	1		10133.605	$17847_{2}^{{}^{\circ}}$ – 27980_3_
9862.127	0		10137.021	11601_1_ – $21738_{2}^{{}^{\circ}}$
9855.745	3		10143.585	9804_5_ – $19948_{4}^{{}^{\circ}}$
9840.923	1		10158.863	$18069_{3}^{{}^{\circ}}$ – 28227_4_
9838.008	1		10161.873	$21165_{3}^{{}^{\circ}}$ – 31326_4_
9837.258	0		10162.648	$14465_{2}^{{}^{\circ}}$ – 24627_1_
9833.424	8		10166.610	3865_1_ – $14032_{2}^{{}^{\circ}}$
9826.452	7		10173.823	5563_1_ – $15736_{1}^{{}^{\circ}}$
9819.178	1		10181.360	9804_5_ – $19986_{6}^{{}^{\circ}}$
9814.962	1		10185.733	$20322_{0}^{{}^{\circ}}$ – 30508_5_
9813.153	0		10187.611	$16783_{4}^{{}^{\circ}}$ – 26971_4_
9812.698	7		10188.083	$8243_{2}^{{}^{\circ}}$ – 18431_3_
9801.710	0		10199.505	$14465_{2}^{{}^{\circ}}$ – 24664_3_
9797.252	1		10204.145	$18069_{3}^{{}^{\circ}}$ – 28273_2_
9796.200	5		10205.241	4961_4_ – $15166_{3}^{{}^{\circ}}$
9789.508	2		10212.218	$17354_{1}^{{}^{\circ}}$ – 27566_2_
9785.356	0		10216.551	$18011_{0}^{{}^{\circ}}$ – 28227_4_
9782.141	0		10219.908	13962_1_ – $24182_{2}^{{}^{\circ}}$
9769.538	2		10233.092	15863_2_ – $26096_{3}^{{}^{\circ}}$
9764.606	0		10238.261	$15166_{3}^{{}^{\circ}}$ – 25405_4_
9757.222	1		10246.009	12847_3_ – $23093_{2}^{{}^{\circ}}$
9754.019	1		10249.374	15863_2_ – $26113_{2}^{{}^{\circ}}$
9753.597	2		10249.817	$21077_{0}^{{}^{\circ}}$ – 31326_4_
9746.464	7		10257.318	3687_2_ – $13945_{3}^{{}^{\circ}}$
9743.565	4		10260.370	$16783_{4}^{{}^{\circ}}$ – 27044_3_
9738.624	1		10265.576	12847_3_ – $23113_{4}^{{}^{\circ}}$
9736.216	1		10268.115	$18614_{1}^{{}^{\circ}}$ – 28882_2_
9716.146	2		10289.325	$18069_{3}^{{}^{\circ}}$ – 28.358_3_
9702.272	3		10304.038	$15618_{3}^{{}^{\circ}}$ – 25923_4_
9701.580	1		10304.773	$18053_{4}^{{}^{\circ}}$ – 28358_3_
9700.564	7		10305.852	2869_3_ – $13175_{4}^{{}^{\circ}}$
9695.033	2		10311.732	13297_4_ – $23609_{0}^{{}^{\circ}}$
9678.235	0		10329.629	$20214_{3}^{{}^{\circ}}$ – 30544_2_
9676.940	0		10331.012	$8243_{2}^{{}^{\circ}}$ – 18574_1_
9676.840	3		10331.118	$18011_{0}^{{}^{\circ}}$ – 28342_5_
9676.107	2		10331.901	$19503_{3}^{{}^{\circ}}$ – 29835_3_
9674.790	2		10333.308	$11241_{3}^{{}^{\circ}}$ – 21575_2_
9664.700	6		10344.096	3687_2_ – $14032_{2}^{{}^{\circ}}$
9663.647	1		10345.223	13962_1_ – $24307_{2}^{{}^{\circ}}$
9656.439	1		10352.945	$11241_{3}^{{}^{\circ}}$ – 21594_3_
9652.004	0		10357.702	13297_4_ – $23655_{4}^{{}^{\circ}}$
9643.322	1		10367.027	$14032_{2}^{{}^{\circ}}$ – 24399_3_
9643.147	0		10367.215	$20522_{2}^{{}^{\circ}}$ – 30889_1_
9636.906	1		10373.929	17073_1_ – $27447_{2}^{{}^{\circ}}$
9632.647	6		10378.516	3865_1_ – $14243_{1}^{{}^{\circ}}$
9630.745	1		10380.565	$14247_{0}^{{}^{\circ}}$ – 24627_1_
9629.572	6		10381.830	3865_1_ – $14247_{0}^{{}^{\circ}}$
9629.231	0		10382.198	$22399_{0}^{{}^{\circ}}$ – 32781_4_
9627.672	1		10383.879	$14243_{1}^{{}^{\circ}}$ – 24627_1_
9625.204	3		10386.541	$19986_{6}^{{}^{\circ}}$ – 30372_6_
9623.414	0		10388.473	$22163_{4}^{{}^{\circ}}$ – 32551_3_
9619.222	1		10393.001	15970_3_ – $26363_{2}^{{}^{\circ}}$
9608.933	1		10404.129	$11241_{3}^{{}^{\circ}}$ – 21645_4_
9605.809	0		10407.513	$16783_{4}^{{}^{\circ}}$ – 27191_5_
9595.395	4		10418.808	13962_1_ – $24381_{2}^{{}^{\circ}}$
9588.809	0		10425.964	$19588_{0}^{{}^{\circ}}$ – 30014_4_
9587.027	1		10427.902	$14243_{1}^{{}^{\circ}}$ – 24671_2_
9582.816	4		10432.484	13088_3_ – $23521_{3}^{{}^{\circ}}$
9571.505	1		10444.813	$19516_{2}^{{}^{\circ}}$ – 29961_1_
9570.405	1		10446.013	11802_2_ – $22248_{2}^{{}^{\circ}}$
9567.826	2		10448.829	$11197_{0}^{{}^{\circ}}$ – 21645_4_
9567.283	1		10449.422	$16346_{4}^{{}^{\circ}}$ – 26796_3_
9561.245	5		10456.021	$8243_{2}^{{}^{\circ}}$ – 18699_2_
9510.950	0		10511.313	$17847_{2}^{{}^{\circ}}$ – 28358_3_
9507.656	2		10514.955	13088_3_ – $23603_{2}^{{}^{\circ}}$
9505.395	5		10517.456	9804_5_ – $20322_{0}^{{}^{\circ}}$
9501.443	0		10521.831	$19986_{6}^{{}^{\circ}}$ – 30508_5_
9500.302	2		10523.095	$12114_{2}^{{}^{\circ}}$ – 22637_3_
9497.191	7		10526.541	0_2_ – $10526_{3}^{{}^{\circ}}$
9495.500	7		10528.416	4961_4_ – $15490_{0}^{{}^{\circ}}$
9486.929	3		10537.928	15970_3_ – $26508_{3}^{{}^{\circ}}$
9474.882	7		10551.327	7502_3_ – $18053_{4}^{{}^{\circ}}$
9470.684	6		10556.003	3687_2_ – $14243_{1}^{{}^{\circ}}$
9467.200	5		10559.888	$16783_{4}^{{}^{\circ}}$ – 27343_3_
9461.208	3		10566.576	13088_3_ – $23655_{4}^{{}^{\circ}}$
9461.031	6		10566.774	7502_3_ – $18069_{3}^{{}^{\circ}}$
9460.871	2		10566.952	7280_2_ – $17847_{2}^{{}^{\circ}}$
9458.629	0		10569.457	$17411_{3}^{{}^{\circ}}$ – 27980_3_
9455.205	2q		10573.284	13847_2_ – $24421_{3}^{{}^{\circ}}$
9450.464	1		10578.589	$16217_{2}^{{}^{\circ}}$ – 26796_3_
9436.815	2		10593.889	11802_2_ – $22396_{1}^{{}^{\circ}}$
9431.600	5		10599.747	3865_1_ – $14465_{2}^{{}^{\circ}}$
9430.921	0		10600.510	17073_1_ – $27674_{2}^{{}^{\circ}}$
9422.317	0		10610.190	$18069_{3}^{{}^{\circ}}$ – 28679_2_
9420.621	2u		10612.100	14226_0_ – $24838_{1}^{{}^{\circ}}$
9417.463	0		10615.659	$19516_{2}^{{}^{\circ}}$ – 30132_2_
9414.090	2		10619.462	13297_4_ – $23916_{4}^{{}^{\circ}}$
9409.353	4		10624.808	$16346_{4}^{{}^{\circ}}$ – 26971_4_
9399.091	7		10636.408	$7795_{4}^{{}^{\circ}}$ – 18431_3_
9392.268	0		10644.135	15863_2_ – $26508_{3}^{{}^{\circ}}$
9390.588	2		10646.040	14204_5_ – $24850_{6}^{{}^{\circ}}$
9388.933	4		10647.916	11601_1_ – $22248_{2}^{{}^{\circ}}$
9388.933	4		10647.916	18549_2_ – $29197_{1}^{{}^{\circ}}$
9384.103	0		10653.397	14226_0_ – $24880_{1}^{{}^{\circ}}$
9383.275	5		10654.337	5563_1_ – $16217_{2}^{{}^{\circ}}$
9380.642	0		10657.327	4961_4_ – $15618_{3}^{{}^{\circ}}$
9366.797	0u		10673.079	12847_3_ – $23521_{3}^{{}^{\circ}}$
9357.253	0		10683.966	$17847_{2}^{{}^{\circ}}$ – 28531_2_
9344.207	1u		10698.882	8111_4_ – $18809_{4}^{{}^{\circ}}$
9344.096	1d		10699.009	$20566_{4}^{{}^{\circ}}$ – 31265_3_
9340.708	4		10702.890	8800_4_ – $19503_{3}^{{}^{\circ}}$
9336.161	1q		10708.102	$14206_{4}^{{}^{\circ}}$ – 24915_3_
9320.071	0		10726.589	$13945_{3}^{{}^{\circ}}$ –24671_2_
9317.727	3		10729.287	$10414_{4}^{{}^{\circ}}$ – 21143_5_
9310.448	2		10737.675	$16783_{4}^{{}^{\circ}}$ – 27521_4_
9307.899	4		10740.616	$19986_{6}^{{}^{\circ}}$ – 30726_7_
9300.018	1		10749.718	$20214_{3}^{{}^{\circ}}$ – 30964_3_
9294.976	1		10755.549	12847_3_ – $23603_{2}^{{}^{\circ}}$
9289.564	5		10761.815	9804_5_ – $20566_{4}^{{}^{\circ}}$
9276.273	5		10777.234	3687_2_ – $14465_{2}^{{}^{\circ}}$
9271.181	1		10783.153	0_2_ – $10783_{2}^{{}^{\circ}}$
9270.155	2		10784.347	$19588_{0}^{{}^{\circ}}$ – 30372_6_
9266.920	4		10788.111	8800_4_ – $19588_{0}^{{}^{\circ}}$
9266.208	6		10788.941	7280_2_ – $18069_{3}^{{}^{\circ}}$
9263.682	0		10791.882	$10783_{3}^{{}^{\circ}}$ – 21575_2_
9260.327	2		10795.792	11601_1_ – $22396_{1}^{{}^{\circ}}$
9250.579	0		10807.168	12847_3_ – $23655_{4}^{{}^{\circ}}$
9245.257	1		10813.389	$18069_{3}^{{}^{\circ}}$ – 28882_2_
9239.328	1		10820.329	15970_3_ – $26790_{4}^{{}^{\circ}}$
9237.550	2		10822.411	$20423_{1}^{{}^{\circ}}$ – 31245_2_
9234.396	3		10826.108	$18930_{3}^{{}^{\circ}}$ – 29756_4_
9233.858	50	5	10826.738	$16217_{2}^{{}^{\circ}}$ – 27044_3_
9232.493	50	4	10828.339	13088_3_ – $23916_{4}^{{}^{\circ}}$
9230.616	1		10830.541	$21165_{3}^{{}^{\circ}}$ – 31995_4_
9229.218	1		10832.181	$17847_{2}^{{}^{\circ}}$ – 28679_2_
9227.870	1		10833.764	$22855_{3}^{{}^{\circ}}$ – 33689_2_
9227.517	300	50	10834.178	$18011_{0}^{{}^{\circ}}$ – 28845_4_
9227.057	2		10834.718	$21902_{4}^{{}^{\circ}}$ – 32737_5_
9222.345	20	5	10840.254	19713_3_ – $30553_{2}^{{}^{\circ}}$
9221.439	40	5	10841.319	$17501_{0}^{{}^{\circ}}$ – 28342_5_
9218.851	2		10844.363	21594_3_ – $32439_{4}^{{}^{\circ}}$
9218.670	1		10844.576	18574_1_ – $29419_{2}^{{}^{\circ}}$
9218.612	1		10844.644	18574_1_ – $29419_{2}^{{}^{\circ}}$
9218.612	1		10844.644	18574_1_ – $29419_{2}^{{}^{\circ}}$
9208.593	50		10856.443	$11241_{3}^{{}^{\circ}}$ – 22098_4_
9208.035	8	2	10857.101	$13175_{4}^{{}^{\circ}}$ – 24032_4_
9203.988	500	75	10861.874	6362_2_ – $17224_{2}^{{}^{\circ}}$
9200.638	1h		10865.829	$18069_{3}^{{}^{\circ}}$ – 28934_3_
9199.676	8		10866.966	11802_2_ – $22669_{3}^{{}^{\circ}}$
9197.246	3		10869.837	18549_2_ – $29419_{2}^{{}^{\circ}}$
9191.681	2		10876.418	13962_1_ – $24838_{1}^{{}^{\circ}}$
9188.503	2		10880.179	21143_5_ – $32023_{0}^{{}^{\circ}}$
9186.364	1		10882.713	$22508_{2}^{{}^{\circ}}$ – 33390_1_
9179.820	2		10890.471	$15490_{0}^{{}^{\circ}}$ – 26380_5_
9178.777	200	15	10891.708	15493_4_ – $26384_{4}^{{}^{\circ}}$
9176.967	2		10893.856	$21902_{4}^{{}^{\circ}}$ – 32796_3_
9175.085	2		10896.091	$22877_{1}^{{}^{\circ}}$ – 33773_1_
9171.262	2		10900.633	$22508_{2}^{{}^{\circ}}$ – 33408_3_
9170.810	100	4	10901.170	$11197_{0}^{{}^{\circ}}$ – 22098_4_
9167.793	20	2	10904.758	$18930_{3}^{{}^{\circ}}$ – 29835_3_
9167.469	1		10905.143	13297_4_ – $24202_{4}^{{}^{\circ}}$
9166.738	1		10906.013	$21890_{3}^{{}^{\circ}}$ – 32796_3_
9165.890	400	25	10907.022	11601_1_ – $22508_{2}^{{}^{\circ}}$
9165.008	5	1	10908.071	15970_3_ – $26878_{3}^{{}^{\circ}}$
9164.261	4		10908.961	21594_3_ – $32503_{2}^{{}^{\circ}}$
9156.909	4		10917.719	13962_1_ – $24880_{1}^{{}^{\circ}}$
9155.929	4	1	10918.888	$18930_{3}^{{}^{\circ}}$ – 29849_4_
9153.352	8	2	10921.962	13847_2_ – $24769_{3}^{{}^{\circ}}$
9152.771	3		10922.655	$19039_{2}^{{}^{\circ}}$ – 29961_1_
9147.790	3	1	10928.602	21575_2_ – $32503_{2}^{{}^{\circ}}$
9141.782	8	2	10935.785	$21077_{0}^{{}^{\circ}}$ – 32012_4_
9141.337	2h		10936.317	$22163_{4}^{{}^{\circ}}$ – 33099_3_
9141.245	1		10936.427	$22669_{3}^{{}^{\circ}}$ – 33606_2_
9137.725	8	2	10940.640	18699_2_ – $29640_{1}^{{}^{\circ}}$
9134.679	150	10	10944.288	$19986_{6}^{{}^{\circ}}$ – 30930_6_
9132.261	40	8	10947.186	$17411_{3}^{{}^{\circ}}$ – 28358_3_
9129.173	20	5	10950.889	17073_1_ – $28024_{1}^{{}^{\circ}}$
9126.321	20	4	10954.311	$22877_{1}^{{}^{\circ}}$ – 33831_4_
9123.393	1		10957.827	$22141_{3}^{{}^{\circ}}$ – 33099_3_
9118.125	50	3	10964.158	$19588_{0}^{{}^{\circ}}$ – 30552_4_
9113.239	1		10970.036	$22338_{3}^{{}^{\circ}}$ – 33309_2_
9110.964	2		10972.775	17959_4_ – $28932_{4}^{{}^{\circ}}$
9107.788	2		10976.601	$22855_{3}^{{}^{\circ}}$ – 33831_4_
9107.216	100		10977.291	13297_4_ – $24274_{0}^{{}^{\circ}}$
9103.165	2		10982.176	19273_2_ – $30255_{3}^{{}^{\circ}}$
9101.064	20	3	10984.711	19532_4_ – $30517_{0}^{{}^{\circ}}$
9099.364	2		10986.763	18699_2_ – $29686_{3}^{{}^{\circ}}$
9095.713	4		10991.173	13847_2_ – $24838_{1}^{{}^{\circ}}$
9094.821	400	100	10992.251	6362_2_ – $17354_{1}^{{}^{\circ}}$
9090.809	200	10	10997.103	$16346_{4}^{{}^{\circ}}$ – 27343_3_
9084.511	3		11004.726	$20322_{0}^{{}^{\circ}}$ – 31326_4_
9082.305	3	1	11007.399	$20922_{2}^{{}^{\circ}}$ – 31929_3_
9076.185	8	1	11014.821	15493_4_ – $26508_{3}^{{}^{\circ}}$
9074.980	1		11016.284	$19948_{4}^{{}^{\circ}}$ – 30964_3_
9072.954	1		11018.744	$22399_{0}^{{}^{\circ}}$ – 33418_4_
9067.890	1		11024.897	$22248_{2}^{{}^{\circ}}$ – 33273_1_
9067.657	2		11025.181	$18809_{4}^{{}^{\circ}}$ – 29835_3_
9067.234	5	1	11025.695	15970_3_ – $26995_{3}^{{}^{\circ}}$
9063.948	200	10	11029.692	$8243_{2}^{{}^{\circ}}$ – 19273_2_
9062.879	2		11030.993	$20214_{3}^{{}^{\circ}}$ – 31245_2_
9061.672	2		11032.463	13847_2_ – $24880_{1}^{{}^{\circ}}$
9059.264	3		11035.395	$17847_{2}^{{}^{\circ}}$ – 28882_2_
9056.071	15	2	11039.286	$18382_{0}^{{}^{\circ}}$ – 29422_1_
9054.306	2		11041.438	$19503_{3}^{{}^{\circ}}$ – 30544_2_
9048.503	50		11048.519	$10526_{3}^{{}^{\circ}}$ – 21575_2_
9048.248	800b	75	11048.830	6362_2_ – $17411_{3}^{{}^{\circ}}$
9046.960	3		11050.403	$18053_{4}^{{}^{\circ}}$ – 29104_4_
9046.137	4		11051.409	$22163_{4}^{{}^{\circ}}$ – 33214_5_
9046.137	4		11051.409	$22669_{3}^{{}^{\circ}}$ – 33721_2_
9045.341	200	20	11052.381	11802_2_ – $22855_{3}^{{}^{\circ}}$
9041.717	2		11056.811	$22508_{2}^{{}^{\circ}}$ – 33564_3_
9039.277	2		11059.796	$22399_{0}^{{}^{\circ}}$ – 33459_4_
9038.935	2		11060.214	$20566_{4}^{{}^{\circ}}$ – 31626_5_
9037.880	200	10	11061.505	$17501_{0}^{{}^{\circ}}$ – 28562_4_
9034.490	2		11065.656	18574_1_ – $29640_{1}^{{}^{\circ}}$
9032.457	3		11068.146	$10526_{3}^{{}^{\circ}}$ – 21594_3_
9031.811	150	10	11068.938	12847_3_ – $23916_{4}^{{}^{\circ}}$
9026.157	2		11075.872	21575_2_ – $32650_{1}^{{}^{\circ}}$
9026.004	2		11076.059	2869_3_ – $13945_{3}^{{}^{\circ}}$
9016.581	300	20	11087.634	$12114_{2}^{{}^{\circ}}$ – 23201_3_
9016.382	3		11087.879	$18053_{4}^{{}^{\circ}}$ – 29141_5_
9013.959	5	1	11090.860	17959_4_ – $29050_{0}^{{}^{\circ}}$
9013.959	5	1	11090.860	18549_2_ – $29640_{0}^{{}^{\circ}}$
9013.581	1		11091.325	15970_3_ – $27061_{2}^{{}^{\circ}}$
9012.517	150	8	11092.634	$18011_{0}^{{}^{\circ}}$ – 29104_4_
9011.500	4	1	11093.886	13088_3_ – $24182_{2}^{{}^{\circ}}$
8997.862	100	10	11110.701	$11877_{1}^{{}^{\circ}}$ – 22988_2_
8995.178	100	10	11114.016	13088_3_ – $24202_{4}^{{}^{\circ}}$
8994.447	2		11114.919	17398_3_ – $28513_{2}^{{}^{\circ}}$
8991.072	1		11119.092	$20922_{2}^{{}^{\circ}}$ – 32041_2_
8990.879	10	2	11119.330	$10526_{3}^{{}^{\circ}}$ – 21645_4_
8987.396	75	10	11123.640	13297_4_ – $24421_{3}^{{}^{\circ}}$
8985.270	15	4	11126.271	$16217_{2}^{{}^{\circ}}$ – 27343_3_
8983.722	2		11128.189	$22399_{0}^{{}^{\circ}}$ – 33527_4_
8982.127	2		11130.165	$18011_{0}^{{}^{\circ}}$ – 29141_5_
8980.723	10	3	11131.905	15863_2_ – $26995_{3}^{{}^{\circ}}$
8978.886	3		11134.182	$19516_{2}^{{}^{\circ}}$ – 30651_3_
8976.639	3	1	11136.969	18549_2_ – $29686_{3}^{{}^{\circ}}$
8969.853	100	15	11145.395	$19227_{6}^{{}^{\circ}}$ – 30372_6_
8967.635	500	100	11148.151	8800_4_ – $19948_{4}^{{}^{\circ}}$
8965.074	2		11151.336	14204_5_ – $25355_{4}^{{}^{\circ}}$
8956.495	2		11162.017	$22669_{3}^{{}^{\circ}}$ – 33831_4_
8955.834	200	15	11162.841	2869_3_ – $14032_{2}^{{}^{\circ}}$
8952.133	2		11167.456	$22141_{3}^{{}^{\circ}}$ – 33309_2_
8949.114	100	20	11171.223	13088_3_ – $24259_{4}^{{}^{\circ}}$
8944.447	1		11177.052	$15618_{3}^{{}^{\circ}}$ – 26796_3_
8942.502	2		11179.483	21575_2_ – $32754_{3}^{{}^{\circ}}$
8941.641	8	2	11180.560	$10414_{4}^{{}^{\circ}}$ – 21594_3_
8935.225	1		11188.588	$22338_{3}^{{}^{\circ}}$ – 33527_4_
8933.357	1		11190.928	17398_3_ – $28589_{3}^{{}^{\circ}}$
8930.006	2		11195.127	18549_2_ – $29744_{3}^{{}^{\circ}}$
8928.384	2		11197.161	$21902_{4}^{{}^{\circ}}$ – 33099_3_
8928.078	25	5	11197.545	15863_2_ – $27061_{2}^{{}^{\circ}}$
8927.723	15	5	11197.990	17959_4_ – $29157_{3}^{{}^{\circ}}$
8924.217	3w		11202.389	$18930_{3}^{{}^{\circ}}$ – 30132_2_
8922.566	2		11204.462	$18809_{4}^{{}^{\circ}}$ – 30014_4_
8918.698	1		11209.321	$21890_{3}^{{}^{\circ}}$ – 33099_3_
8918.584	1		11209.465	$22396_{1}^{{}^{\circ}}$ – 33606_2_
8912.867	2		11216.655	21645_4_ – $32862_{4}^{{}^{\circ}}$
8910.844	75	4	11219.201	13088_3_ – $24307_{2}^{{}^{\circ}}$
8907.016	40	2	11224.023	$13175_{4}^{{}^{\circ}}$ – 24399_3_
8905.566	2		11225.850	$22338_{3}^{{}^{\circ}}$ – 33564_3_
8902.815	2–		11229.319	19532_4_ – $30761_{3}^{{}^{\circ}}$
8900.891	2		11231.746	$10414_{4}^{{}^{\circ}}$ – 21645_4_
8893.533	15	5	11241.039	$19516_{2}^{{}^{\circ}}$ – 30758_2_
8892.973	150	15	11241.747	0_2_ – $11241_{3}^{{}^{\circ}}$
8892.778	2		11241.993	$21539_{4}^{{}^{\circ}}$ – 32781_4_
8889.970	2		11245.544	$22163_{4}^{{}^{\circ}}$ – 33408_3_
8889.187	100	10	11246.535	11802_2_ – $23049_{1}^{{}^{\circ}}$
8882.601	1		11254.873	$19503_{3}^{{}^{\circ}}$ – 30758_2_
8877.739	1		11261.037	22401_1_ – $33662_{1}^{{}^{\circ}}$
8875.223	100	8	11264.229	13297_4_ – $24561_{3}^{{}^{\circ}}$
8874.156	2		11265.584	$22508_{2}^{{}^{\circ}}$ – 33773_1_
8872.787	1		11267.322	$22338_{3}^{{}^{\circ}}$ – 33606_2_
8872.409	1		11267.802	21594_3_ – $32862_{4}^{{}^{\circ}}$
8868.822	200	50	11272.359	9804_5_ – $21077_{0}^{{}^{\circ}}$
8867.740	1		11273.735	17073_1_ – $28347_{2}^{{}^{\circ}}$
8865.566	100	8	11276.499	11611_1_ – $22877_{1}^{{}^{\circ}}$
8862.284	2		11280.675	$19227_{6}^{{}^{\circ}}$ – 30508_5_
8860.973	4	1	11282.344	17398_3_ – $28680_{4}^{{}^{\circ}}$
8857.869	4	1	11286.298	$23015_{0}^{{}^{\circ}}$ – 34301_4_
8854.899	10	3	11290.083	15970_3_ – $27260_{3}^{{}^{\circ}}$
8854.136	1		11291.056	11802_2_ – $23093_{2}^{{}^{\circ}}$
8852.784	10	3	11292.781	13088_3_ – $24381_{2}^{{}^{\circ}}$
8852.552	1		11293.077	$21165_{3}^{{}^{\circ}}$ – 32458_4_
8850.543	2		11295.640	21143_5_ – $32439_{4}^{{}^{\circ}}$
8848.294	8	3	11298.511	16554_6_ – $27852_{0}^{{}^{\circ}}$
8847.513	2		11299.508	14226_0_ – $25526_{1}^{{}^{\circ}}$
8843.549	2		11304.573	$20322_{0}^{{}^{\circ}}$ – 31626_5_
8842.359	1		11306.095	19713_3_ – $31019_{4}^{{}^{\circ}}$
8841.841	4		11306.757	$17224_{2}^{{}^{\circ}}$ – 28531_2_
8841.170	150	20	11307.615	7502_3_ – $18809_{4}^{{}^{\circ}}$
8836.139	3		11314.053	17166_5_ – $28480_{4}^{{}^{\circ}}$
8833.471	2		11317.471	18699_2_ – $30017_{3}^{{}^{\circ}}$
8829.686	15	5	11322.322	$20214_{3}^{{}^{\circ}}$ – 31537_3_
8829.470	2		11322.599	$19948_{4}^{{}^{\circ}}$ – 31271_5_
8827.897	2		11324.616	$17354_{1}^{{}^{\circ}}$ – 28679_2_
8821.739	4	1	11332.521	13088_3_ – $24421_{3}^{{}^{\circ}}$
8820.409	50b	10	11334.230	7280_2_ – $18614_{1}^{{}^{\circ}}$
8820.214	20	3	11334.481	12847_3_ – $24182_{2}^{{}^{\circ}}$
8812.507	4		11344.393	$17501_{0}^{{}^{\circ}}$ – 28845_4_
8810.240	15		11347.312	15970_3_ – $27317_{3}^{{}^{\circ}}$
8808.667	2		11349.339	$18069_{3}^{{}^{\circ}}$ – 29418_2_
8806.220	2		11352.492	$15618_{3}^{{}^{\circ}}$ – 26971_4_
8804.580	75	3	11354.607	12847_3_ – $24202_{4}^{{}^{\circ}}$
8800.465	3		11359.916	21594_3_ – $32954_{2}^{{}^{\circ}}$
8799.353	1		11361.352	$21738_{2}^{{}^{\circ}}$ – 33099_3_
8798.171	10	3	11362.878	19713_3_ – $31075_{2}^{{}^{\circ}}$
8792.058	100	5	11370.779	14204_5_ – $25575_{4}^{{}^{\circ}}$
8790.372	10	2	11372.960	$19516_{2}^{{}^{\circ}}$ – 30889_1_
8786.029	2		11378.581	$19948_{4}^{{}^{\circ}}$ – 31326_4_
8784.139	3	1	11381.029	$21077_{0}^{{}^{\circ}}$ – 32458_4_
8781.093	25	1	11384.977	496_4_ – $16346_{4}^{{}^{\circ}}$
8780.337	1		11385.958	$22141_{3}^{{}^{\circ}}$ – 33527_4_
8779.898	1		11386.527	$21165_{3}^{{}^{\circ}}$ – 32551_3_
8775.575	200	40	11392.136	811I_4_ – $19503_{3}^{{}^{\circ}}$
8773.524	5	1	11394.799	$16217_{2}^{{}^{\circ}}$ – 27612_3_
8772.803	150	8	11395.736	$11241_{3}^{{}^{\circ}}$ – 22637_3_
8772.384	8	1	11396.280	15863_2_ – $27260_{3}^{{}^{\circ}}$
8771.465	1		11397.474	21645_4_ – $33043_{3}^{{}^{\circ}}$
8768.195	5	1	11401.725	$22163_{4}^{{}^{\circ}}$ – 33564_3_
8766.743	100	15	11403.613	13297_4_ – $24701_{0}^{{}^{\circ}}$
8760.449	100	8	11411.806	12847_3_ – $24259_{4}^{{}^{\circ}}$
8758.247	400	75	11414.675	8800_4_ – $20214_{3}^{{}^{\circ}}$
8755.001	3	1	11418.907	$21890_{3}^{{}^{\circ}}$ – 33309_2_
8753.738	2h		11420.555	$22877_{1}^{{}^{\circ}}$ – 34298_1_
8751.202	10	2	11423.864	$22877_{0}^{{}^{\circ}}$ – 34301_4_
8750.176	1		11425.204	$14465_{2}^{{}^{\circ}}$ – 25890_2_
8748.038	400	100	11427.996	7502_3_ – $18930_{3}^{{}^{\circ}}$
8747.498	2		11428.701	$19817_{1}^{{}^{\circ}}$ – 31245_2_
8747.258	2		11429.015	$20566_{4}^{{}^{\circ}}$ – 31995_4_
8744.965	2	1	11432.012	$23015_{0}^{{}^{\circ}}$ – 34447_4_
8744.598	5	1	11432.492	$22399_{0}^{{}^{\circ}}$ – 33831_4_
8743.269	15	2	11434.229	$23655_{4}^{{}^{\circ}}$ – 35089_3_
8739.251	1		11439.486	17073_1_ – $28513_{2}^{{}^{\circ}}$
8735.735	2		11444.091	$16783_{4}^{{}^{\circ}}$ – 28227_4_
8734.482	5	1	11445.732	$23015_{0}^{{}^{\circ}}$ – 34460_5_
8734.023	75	8	11446.334	$20566_{4}^{{}^{\circ}}$ – 32012_4_
8732.992	2		11447.685	$19516_{2}^{{}^{\circ}}$ – 30964_3_
8732.426	150	50	11448.427	11601_1_ – $23049_{1}^{{}^{\circ}}$
8730.818	15	2	11450.536	19273_2_ – $30723_{1}^{{}^{\circ}}$
8728.550	2		11453.511	15863_2_ – $27317_{1}^{{}^{\circ}}$
8727.446	4		11454.960	$17224_{2}^{{}^{\circ}}$ – 28679_2_
8724.375	40	2	11458.992	13847_2_ – $25306_{2}^{{}^{\circ}}$
8723.718	15	2	11459.855	$13945_{3}^{{}^{\circ}}$ – 25405_4_
8722.459	15	2	11461.509	$19503_{3}^{{}^{\circ}}$ – 30964_3_
8717.754	10	2	11467.695	18549_2_ – $30017_{3}^{{}^{\circ}}$
8716.427	2		11469.441	$8243_{2}^{{}^{\circ}}$ – 19713_3_
8715.065	1		11471.233	$17411_{3}^{{}^{\circ}}$ – 28882_2_
8714.917	1		11471.428	$20322_{0}^{{}^{\circ}}$ – 31793_4_
8714.257	8	1	11472.297	13297_4_ – $24769_{3}^{{}^{\circ}}$
8713.654	20	2	11473.090	13088_3_ – $24561_{3}^{{}^{\circ}}$
8712.838	10	2	11474.165	13847_2_ – $25321_{3}^{{}^{\circ}}$
8710.410	50	2	11477.363	8111_4_ – $19588_{0}^{{}^{\circ}}$
8709.233	200	50	11478.914	3687_2_ – $15166_{3}^{{}^{\circ}}$
8707.356	75	3	11481.389	$15490_{0}^{{}^{\circ}}$ – 26971_4_
8704.855	25	3	11484.688	6362_2_ – $17847_{2}^{{}^{\circ}}$
8703.697	25	2	11486.216	14204_5_ – $25690_{0}^{{}^{\circ}}$
8703.340	3		11486.687	19532_4_ – $31019_{4}^{{}^{\circ}}$
8702.021	8	1	11488.428	19273_2_ – $30761_{3}^{{}^{\circ}}$
8701.117	50	3	11489.622	$13175_{4}^{{}^{\circ}}$ – 24664_3_
8693.669	2		11499.465	$19227_{6}^{{}^{\circ}}$ – 30726_7_
8691.333	201	1	11502.556	15493_4_ – $26995_{3}^{{}^{\circ}}$
8689.176	8	3	11505.411	$19039_{2}^{{}^{\circ}}$ – 30544_2_
8688.429	2		11506.400	$21902_{4}^{{}^{\circ}}$ – 33408_3_
8687.841	50	4	11507.179	$15490_{0}^{{}^{\circ}}$ – 26997_6_
8682.216	10	1	11514.634	17166_5_ – $28680_{4}^{{}^{\circ}}$
8681.305	2		11515.842	$21902_{4}^{{}^{\circ}}$ – 33418_4_
8679.439	15	1	11518.318	$18614_{1}^{{}^{\circ}}$ – 30132_2_
8679.303	1		11518.499	$20522_{2}^{{}^{\circ}}$ – 32041_2_
8679.303	1		11518.499	$21890_{3}^{{}^{\circ}}$ – 33408_3_
8678.490	10	1	11519.578	17398_3_ – $28917_{2}^{{}^{\circ}}$
8676.629	2s		11522.048	8800_4_ – $20322_{0}^{{}^{\circ}}$
8675.392	15	2	11523.691	$17411_{3}^{{}^{\circ}}$ – 28934_3_
8674.634	1		11524.698	$22248_{2}^{{}^{\circ}}$ – 33773_1_
8672.281	1		11527.825	$17354_{1}^{{}^{\circ}}$ – 28882_2_
8672.159	3		11527.987	$21890_{3}^{{}^{\circ}}$ – 33418_4_
8668.118	100	20	11533.362	12847_3_ – $24381_{2}^{{}^{\circ}}$
8667.444	15	2	11534.258	17398_3_ – $28932_{4}^{{}^{\circ}}$
8665.491	400	100	11536.858	7502_3_ – $19039_{2}^{{}^{\circ}}$
8663.348	4		11539.712	19273_2_ – $30812_{2}^{{}^{\circ}}$
8662.270	2		11541.148	$18011_{0}^{{}^{\circ}}$ – 29552_6_
8660.494	1		11543.515	$21252_{2}^{{}^{\circ}}$ – 32796_3_
8659.754	2		11544.501	20054_2_ – $1599_{2}^{{}^{\circ}}$
8657.541	3		11547.452	$22141_{3}^{{}^{\circ}}$ – 33689_2_
8655.873	50	8	11549.677	14204_5_ – $25753_{0}^{{}^{\circ}}$
8651.256	3		11555.841	18699_2_ – $30255_{3}^{{}^{\circ}}$
8650.455	2		11556.911	$21902_{4}^{{}^{\circ}}$ – 33459_4_
8649.144	50	5	11558.663	$16783_{4}^{{}^{\circ}}$ – 28342_5_
8648.386	40	4	11559.676	$20214_{3}^{{}^{\circ}}$ – 31774_3_
8648.282	5		11559.815	$21539_{4}^{{}^{\circ}}$ – 33099_3_
8645.310	150	25	11563.789	13962_1_ – $25526_{1}^{{}^{\circ}}$
8642.849	1		11567.081	$22877_{1}^{{}^{\circ}}$ – 34444_2_
8641.383	3	1	11569.044	$21890_{3}^{{}^{\circ}}$ – 33459_4_
8640.972	4		11569.594	$22877_{1}^{{}^{\circ}}$ – 34447_4_
8639.655	8		11571.358	$17847_{2}^{{}^{\circ}}$ – 29418_2_
8639.444	150	5	11571.640	$10526_{3}^{{}^{\circ}}$ – 22098_4_
8638.360	100	5	11573.092	12847_3_ – $24421_{3}^{{}^{\circ}}$
8637.271	5	1	11574.551	$16783_{4}^{{}^{\circ}}$ – 28358_3_
8636.928	2		11575.011	$17847_{2}^{{}^{\circ}}$ – 29422_1_
8636.680	2		11575.343	17073_1_ – $28649_{1}^{{}^{\circ}}$
8634.114	3		11578.784	$20214_{3}^{{}^{\circ}}$ – 31793_4_
8634.022	5h	1	11578.907	$19516_{2}^{{}^{\circ}}$ – 31095_1_
8633.966	3		11578.982	$18382_{0}^{{}^{\circ}}$ – 2996_1_
8633.455	3	1	11579.667	$22141_{3}^{{}^{\circ}}$ – 33721_2_
8632.169	3	1	11581.393	18699_2_ – $30281_{1}^{{}^{\circ}}$
8631.788	2		11581.904	$18069_{3}^{{}^{\circ}}$ – 29650_2_
8631.350	50	2	11582.491	14226_0_ – $25809_{1}^{{}^{\circ}}$
8630.770	20h	2h	11583.270	$22877_{0}^{{}^{\circ}}$ – 34460_5_
8630.330	5	1	11583.860	15863_2_ – $27447_{2}^{{}^{\circ}}$
8626.611	1		11588.854	$19948_{4}^{{}^{\circ}}$ – 31537_3_
8626.254	1		11589.334	$22855_{3}^{{}^{\circ}}$ – 34444_2_
8622.093	5	1	11594.927	13847_2_ – $25442_{3}^{{}^{\circ}}$
8621.315	75	2	11595.973	2869_3_ – $14465_{2}^{{}^{\circ}}$
8618.598	5	1	11599.629	17073_1_ – $28673_{2}^{{}^{\circ}}$
8616.218	100	10	11602.833	$17501_{0}^{{}^{\circ}}$ – 29104_4_
8614.687	5	1	11604.895	$21668_{1}^{{}^{\circ}}$ – 33273_1_
8611.754	3		11608.847	19532_4_ – $31141_{0}^{{}^{\circ}}$
8609.370	4	1	11612.062	$19817_{1}^{{}^{\circ}}$ – 31429_1_
8607.724	8	2	11614.282	$18930_{3}^{{}^{\circ}}$ – 30544_2_
8605.146	2		11617.762	$20423_{1}^{{}^{\circ}}$ – 32041_2_
8604.922	4		11618.064	$10783_{2}^{{}^{\circ}}$ – 22401_1_
8601.844	1		11622.221	$18930_{3}^{{}^{\circ}}$ – 30552_4_
8600.016	3		11624.692	21645_4_ – $33270_{4}^{{}^{\circ}}$
8596.686	5		11629.195	$15166_{3}^{{}^{\circ}}$ – 26796_3_
8596.466	8	1	11629.492	$20922_{2}^{{}^{\circ}}$ – 32551_3_
8595.655	1		11630.590	$18053_{4}^{{}^{\circ}}$ – 29684_5_
8595.359	1		11630.990	$21165_{3}^{{}^{\circ}}$ – 32796_3_
8595.307	1		11631.060	$21165_{3}^{{}^{\circ}}$ – 32796_3_
8594.965	1		11631.523	$22669_{3}^{{}^{\circ}}$ – 34301_4_
8593.103	100	10	11634.044	$16346_{4}^{{}^{\circ}}$ – 27980_3_
8589.687	3		11638.670	$19503_{3}^{{}^{\circ}}$ – 31141_3_
8588.435	50	2	11640.367	$17501_{0}^{{}^{\circ}}$ – 29141_5_
8588.221	8		11640.657	$19986_{6}^{{}^{\circ}}$ – 31626_5_
8583.974	2		11646.416	$14243_{1}^{{}^{\circ}}$ – 25890_2_
8582.016	4		11649.074	21645_4_ – $33294_{3}^{{}^{\circ}}$
8579.355	3		11652.687	$21738_{2}^{{}^{\circ}}$ – 33390_1_
8577.279	100	15	11655.507	$12114_{2}^{{}^{\circ}}$ – 23769_1_
8575.327	75	5	11658.160	$17224_{2}^{{}^{\circ}}$ – 28882_2_
8574.577	2		11659.180	18549_2_ – $30208_{2}^{{}^{\circ}}$
8574.081	50	10	11659.854	$21077_{0}^{{}^{\circ}}$ – 32737_5_
8573.122	500	100	11661.159	5563_1_ – $17224_{2}^{{}^{\circ}}$
8572.075	4	1	11662.583	$21902_{4}^{{}^{\circ}}$ – 33564_3_
8564.748	50	4	11672.560	$18614_{1}^{{}^{\circ}}$ – 30286_1_
8564.570	5		11672.803	$18011_{0}^{{}^{\circ}}$ – 29684_5_
8564.141	25	3	11673.387	$20322_{0}^{{}^{\circ}}$ – 31995_4_
8563.151	15	2	11674.737	$21890_{3}^{{}^{\circ}}$ – 33564_3_
8563.003	3		11674.939	$21539_{4}^{{}^{\circ}}$ – 33214_5_
8562.314	5	1	11675.878	21594_3_ – $33270_{4}^{{}^{\circ}}$
8560.425	100	4	11678.454	11802_2_ – $23481_{1}^{{}^{\circ}}$
8558.448	751	4	11681.152	13088_3_ – $24769_{3}^{{}^{\circ}}$
8556.589	10b		11683.690	13297_4_ – $24981_{3}^{{}^{\circ}}$
8556.324	100	20	11684.052	$10414_{4}^{{}^{\circ}}$ – 22098_4_
8554.945	200	50	11685.935	2558_0_ – $14243_{1}^{{}^{\circ}}$
8553.895	1		11687.370	$16346_{4}^{{}^{\circ}}$ – 28034_5_
8551.763	8	1	11690.284	$22141_{3}^{{}^{\circ}}$ – 3383_4_
8551.460	2		11690.698	$20322_{0}^{{}^{\circ}}$ – 32012_4_
8549.932	1		11692.787	$17411_{3}^{{}^{\circ}}$ – 29104_4_
8543.721	150	40	11701.287	$15490_{0}^{{}^{\circ}}$ – 27191_5_
8542.618	3h		11702.798	$18053_{4}^{{}^{\circ}}$ – 29756_4_
8540.232	4	1	11706.068	18549_2_ – $30255_{3}^{{}^{\circ}}$
8539.795	150	25	11706.667	6362_2_ – $18069_{3}^{{}^{\circ}}$
8537.379	3		11709.979	22401_1_ – $34111_{1}^{{}^{\circ}}$
8534.674	50	2	11713.691	12847_3_ – $24561_{3}^{{}^{\circ}}$
8532.907	10	1	11716.117	$14206_{4}^{{}^{\circ}}$ – 25923_4_
8532.096	8	1	11717.230	19273_2_ – $30990_{3}^{{}^{\circ}}$
8531.445	50	2	11718.124	11802_2_ – $23521_{3}^{{}^{\circ}}$
8530.911	40	3	11718.858	$19039_{2}^{{}^{\circ}}$ – 30758_2_
8530.159	2		11719.891	21575_2_ – $33294_{3}^{{}^{\circ}}$
8528.762	3		11721.811	$21668_{1}^{{}^{\circ}}$ – 33390_1_
8528.548	2		11722.105	21575_2_ – $33297_{2}^{{}^{\circ}}$
8526.603	3		11724.779	$15618_{3}^{{}^{\circ}}$ – 27343_3_
8525.651	4		11726.088	20054_2_ – $31780_{3}^{{}^{\circ}}$
8525.386	3		11726.452	17959_4_ – $29686_{3}^{{}^{\circ}}$
8524.785	2		11727.279	$20566_{4}^{{}^{\circ}}$ – 32293_5_
8521.620	4		11731.635	18549_2_ – $30281_{1}^{{}^{\circ}}$
8519.327	50	3	11734.792	9804_5_ – $21539_{4}^{{}^{\circ}}$
8516.555	150	50	11738.612	$19588_{0}^{{}^{\circ}}$ – 31326_4_
8515.615	2		11739.907	$13175_{4}^{{}^{\circ}}$ – 24915_3_
8514.901	8	1	11740.892	13962_1_ – $25703_{2}^{{}^{\circ}}$
8513.624	2		11742.653	18809d_4_ – 30552_4_
8511.906	50	3	11745.023	$18011_{0}^{{}^{\circ}}$ – 29756_4_
8510.622	150	25	11746.795	$11241_{3}^{{}^{\circ}}$ – 22988_2_
8509.273	2		11748.657	$19516_{2}^{{}^{\circ}}$ – 31265_3_
8508.186	5	1	11750.158	$23655_{4}^{{}^{\circ}}$ – 35405_3_
8501.756	8		11759.045	7280_2_ – $19039_{2}^{{}^{\circ}}$
8501.438	50	5	11759.485	17398_3_ – $29157_{3}^{{}^{\circ}}$
8500.011	10	2	11761.459	$22669_{3}^{{}^{\circ}}$ – 34431_3_
8498.731	2		11763.230	$16217_{2}^{{}^{\circ}}$ – 27980_3_
8496.449	50b		11766.390	8800_4_ – $20566_{4}^{{}^{\circ}}$
8496.340	25b		11766.541	17166_5_ – $28932_{4}^{{}^{\circ}}$
8496.044	4		11766.951	15493_4_ – $27260_{3}^{{}^{\circ}}$
8491.811	10		11772.816	15493_4_ – $27266_{4}^{{}^{\circ}}$
8491.368	2		11773.430	17959_4_ – $29733_{0}^{{}^{\circ}}$
8488.564	1		11777.320	$22669_{3}^{{}^{\circ}}$ – 34447_4_
8487.478	15	1	11778.826	$16783_{4}^{{}^{\circ}}$ – 28562_4_
8486.561	50	2	11780.099	$18069_{3}^{{}^{\circ}}$ – 29849_4_
8486.108	2		11780.728	$20214_{3}^{{}^{\circ}}$ – 31995_4_
8485.600	3		11781.433	$18053_{4}^{{}^{\circ}}$ – 29835_3_
8483.298	50	2	11784.630	17959_4_ – $29744_{3}^{{}^{\circ}}$
8482.478	1		11785.769	$23655_{4}^{{}^{\circ}}$ – 35440_3_
8478.505	200		11791.292	$11241_{3}^{{}^{\circ}}$ – 23032_4_
8478.353	500		11791.504	5563_1_ – $17354_{1}^{{}^{\circ}}$
8473.657	1		11798.038	$20214_{3}^{{}^{\circ}}$ – 32012_4_
8472.593	40	3	11799.520	17398_3_ – $29197_{2}^{{}^{\circ}}$
8471.823	200	50	11800.592	11802_2_ – $23603_{2}^{{}^{\circ}}$
8470.358	5		11802.633	19273_2_ – $31075_{2}^{{}^{\circ}}$
8469.451	3		11803.897	$17847_{2}^{{}^{\circ}}$ – 29650_2_
8468.973	8	2	11804.563	$15166_{3}^{{}^{\circ}}$ – 26971_4_
8467.164	5	1	11807.086	15863_2_ – $27670_{3}^{{}^{\circ}}$
8465.668	100	25	11809.172	11601_1_ – $23410_{0}^{{}^{\circ}}$
8464.753	50	3	11810.448	15863_2_ – $27674_{2}^{{}^{\circ}}$
8464.236	150	50	11811.170	$8243_{2}^{{}^{\circ}}$ – 20054_2_
8462.015	4		11814.270	15970_3_ – $27784_{2}^{{}^{\circ}}$
8456.341	50	2	11822.197	4961_4_ – $16783_{4}^{{}^{\circ}}$
8455.209	2		11823.780	$19503_{3}^{{}^{\circ}}$ – 31326_4_
8454.924	50	4	11824.178	15493_4_ – $27317_{3}^{{}^{\circ}}$
8453.419	5	1	11826.283	$20214_{3}^{{}^{\circ}}$ – 32041_2_
8451.839	2		11828.494	$22338_{3}^{{}^{\circ}}$ – 34167_3_
8450.836	15b		11829.898	$15736_{1}^{{}^{\circ}}$ – 27566_2_
8449.925	2		11831.173	$21890_{3}^{{}^{\circ}}$ – 33721_2_
8447.968	4	1	11833.914	$23015_{0}^{{}^{\circ}}$ – 34849_5_
8446.516	500	150	11835.948	$11197_{0}^{{}^{\circ}}$ – 23032_4_
8445.495	200	150	11837.379	8111_4_ – $19948_{4}^{{}^{\circ}}$
8445.214	100b	8	11837.773	$18011_{0}^{{}^{\circ}}$ – 29849_4_
8440.671	25		11844.145	17073_1_ – $28917_{2}^{{}^{\circ}}$
8440.660	50w	4	11844.160	17073_1_ – $28917_{2}^{{}^{\circ}}$
8440.578	25		11844.275	14204_5_ – $26048_{4}^{{}^{\circ}}$
8439.849	1		11845.298	$19948_{4}^{{}^{\circ}}$ – 31793_4_
8438.789	8		11846.786	13962_1_ – $25809_{1}^{{}^{\circ}}$
8434.780	2		11852.417	$22855_{3}^{{}^{\circ}}$ – 34707_3_
8433.885	8	1	11853.674	18699_2_ – $30553_{2}^{{}^{\circ}}$
8432.485	10	2	11855.643	13847_2_ – $25703_{2}^{{}^{\circ}}$
8430.586	15	1	11858.313	$14032_{2}^{{}^{\circ}}$ – 25890_2_
8426.379	75		11864.233	$14247_{0}^{{}^{\circ}}$ – 26111_1_
8424.031	100		11867.540	$14243_{1}^{{}^{\circ}}$ – 26111_1_
8423.529	3		11868.247	$21738_{2}^{{}^{\circ}}$ – 33606_2_
8422.942	2h		11869.075	$21539_{4}^{{}^{\circ}}$ – 33408_3_
8422.464	1		11869.748	22098_4_ – $33967_{3}^{{}^{\circ}}$
8421.227	500	100	11871.492	3865_1_ – $15736_{1}^{{}^{\circ}}$
8419.431	2		11874.024	$20922_{2}^{{}^{\circ}}$ – 32796_3_
8418.004	200	75	11876.037	$12114_{2}^{{}^{\circ}}$ – 23990_2_
8417.605	8	2	11876.600	$15618_{3}^{{}^{\circ}}$ – 27495_4_
8417.071	75	3	11877.353	$15166_{3}^{{}^{\circ}}$ – 27044_3_
8416.742	500w	150	11877.818	0_2_ – $11877_{1}^{{}^{\circ}}$
8415.375	75	4	11879.747	$20322_{0}^{{}^{\circ}}$ – 32202_5_
8414.938	50	1	11880.364	11601_1_ – $23481_{1}^{{}^{\circ}}$
8414.273	4		11881.303	$16346_{4}^{{}^{\circ}}$ – 28227_4_
8414.005	2		11881.681	$21645_{4}^{{}^{\circ}}$ – 33527_4_
8411.913	100	5	11884.636	17166_5_ – $29050_{0}^{{}^{\circ}}$
8410.763	5		11886.261	19713_3_ – $31599_{2}^{{}^{\circ}}$
8407.010	75	3	11891.567	$20566_{4}^{{}^{\circ}}$ – 32458_4_
8406.674	40	2	11892.043	$11877_{1}^{{}^{\circ}}$ – 23769_1_
8406.314	20	1	11892.552	13088_3_ – $24981_{3}^{{}^{\circ}}$
8401.986	100	8	11898.678	$14481_{6}^{{}^{\circ}}$ – 26380_5_
8400.154	2		11901.273	$22396_{1}^{{}^{\circ}}$ – 34298_1_
8399.253	100	8	11902.550	$15618_{3}^{{}^{\circ}}$ – 27521_4_
8398.171	100	8	11904.083	$18382_{0}^{{}^{\circ}}$ – 30286_1_
8392.847	50	3	11911.634	17398_3_ – $29310_{4}^{{}^{\circ}}$
8392.397	40		11912.273	$19516_{2}^{{}^{\circ}}$ – 31429_1_
8388.528	100	10	11917.767	$7795_{4}^{{}^{\circ}}$ – 19713_3_
8385.726	100	10	11921.750	12847_3_ – $24769_{3}^{{}^{\circ}}$
8384.927	8	2	11922.886	22401_1_ – $34324_{2}^{{}^{\circ}}$
8384.639	1		11923.295	$22508_{2}^{{}^{\circ}}$ – 34431_3_
8383.073	8	2	11925.522	$19039_{2}^{{}^{\circ}}$ – 30964_3_
8379.754	75	4	11930.246	$18614_{1}^{{}^{\circ}}$ – 30544_2_
8379.219	100	8	11931.008	3687_2_ – $15618_{3}^{{}^{\circ}}$
8376.896	3		11934.316	$21165_{3}^{{}^{\circ}}$ – 33099_3_
8375.333	1		11936.543	$22508_{2}^{{}^{\circ}}$ – 34444_2_
8374.766	50	2	11937.351	$21668_{1}^{{}^{\circ}}$ – 33606_2_
8374.202	50	3	11938.155	11802_2_ – $23741_{1}^{{}^{\circ}}$
8371.658	2		11941.783	$21890_{3}^{{}^{\circ}}$ – 33831_4_
8371.303	1		11942.290	$18069_{3}^{{}^{\circ}}$ – 30011_3_
8369.332	200	50	11945.102	$13945_{3}^{{}^{\circ}}$ – 25890_2_
8367.390	150	20	11947.874	$15618_{3}^{{}^{\circ}}$ – 27566_2_
8366.073	150	15	11949.755	11802_2_ – $23752_{2}^{{}^{\circ}}$
8365.198	2		11951.005	$21738_{2}^{{}^{\circ}}$ – 33689_2_
8360.487	75	5	11957.739	$18053_{4}^{{}^{\circ}}$ – 30011_3_
8358.728	150	200	11960.256	$11241_{3}^{{}^{\circ}}$ – 23201_3_
8357.829	50	3	11961.542	13847_2_ – $25809_{1}^{{}^{\circ}}$
8357.192	1		11962.454	$22338_{3}^{{}^{\circ}}$ – 34301_4_
8355.142	50	4	11965.389	21594_3_ – $33560_{4}^{{}^{\circ}}$
8350.865	2		11971.517	$22877_{1}^{{}^{\circ}}$ – 34849_5_
8346.537	50	3	11977.725	$13945_{3}^{{}^{\circ}}$ – 25923_4_
8345.867	50	5	11978.686	18574_1_ – $30553_{2}^{{}^{\circ}}$
8344.174	8	2	11981.117	$19948_{4}^{{}^{\circ}}$ – 31929_3_
8341.475	50	8	11984.993	$20566_{4}^{{}^{\circ}}$ – 32551_3_
8339.403	5	2	11987.971	$17847_{2}^{{}^{\circ}}$ – 29835_3_
8337.330	2h	1	11990.952	$21077_{0}^{{}^{\circ}}$ – 33068_6_
8336.934	20	2	11991.521	19532_4_ – $31523_{3}^{{}^{\circ}}$
8335.700	50	8	11993.297	$15618_{3}^{{}^{\circ}}$ – 27612_3_
8333.441	50	5	11996.548	21594_3_ – $33591_{3}^{{}^{\circ}}$
8330.455	500	300	12000.848	7502_3_ – $19503_{3}^{{}^{\circ}}$
8329.299	2		12002.513	11601_1_ – $23603_{2}^{{}^{\circ}}$
8328.990	15	2	12002.959	$18011_{0}^{{}^{\circ}}$ – 30014_4_
8328.338	15	2	12003.898	18549_2_ – $30553_{2}^{{}^{\circ}}$
8327.213	15	2	12005.520	$1549_{0}^{{}^{\circ}}$ – 27495_4_
8326.068	5	1	12007.171	$17411_{3}^{{}^{\circ}}$ – 29418_2_
8322.891	5	1	12011.754	$16346_{4}^{{}^{\circ}}$ – 28358_3_
8320.859	500	150	12014.688	7502_3_ – $19516_{2}^{{}^{\circ}}$
8317.106	2		12020.109	$21668_{1}^{{}^{\circ}}$ – 33689_2_
8316.595	8	2	12020.848	17398_3_ – $29419_{2}^{{}^{\circ}}$
8314.281	4	1	12024.193	18699_2_ – $30723_{1}^{{}^{\circ}}$
8314.067	5	1	12024.503	13297_4_ – $25321_{3}^{{}^{\circ}}$
8313.281	2		12025.640	20054_2_ – $32080_{1}^{{}^{\circ}}$
8311.019	2		12028.913	$20522_{2}^{{}^{\circ}}$ – 32551_3_
8309.257	15		12031.463	$15490_{0}^{{}^{\circ}}$ – 27521_4_
8307.245	1		12034.377	$18930_{3}^{{}^{\circ}}$ – 30964_3_
8306.411	1		12035.586	$21738_{2}^{{}^{\circ}}$ – 33773_1_
8304.844	5	1	12037.857	$22669_{3}^{{}^{\circ}}$ – 34707_3_
8304.412	50	3	12038.483	$19588_{0}^{{}^{\circ}}$ – 31626_5_
8304.030	8	1	12039.037	18574_1_ – $30613_{0}^{{}^{\circ}}$
8298.353	10	2	12047.273	$19948_{4}^{{}^{\circ}}$ – 31995_4_
8297.984	3		12047.808	$22399_{0}^{{}^{\circ}}$ – 34447_4_
8297.377	25	2	12048.690	$14206_{4}^{{}^{\circ}}$ – 26255_4_
8297.172	50b	5	12048.987	3687_2_ – $15736_{1}^{{}^{\circ}}$
8295.542	100	8	12051.355	$17501_{0}^{{}^{\circ}}$ – 29552_6_
8294.880	8c		12052.317	$21668_{1}^{{}^{\circ}}$ – 33721_2_
8292.521	100	8	12055.745	$16217_{2}^{{}^{\circ}}$ – 28273_2_
8292.039	4		12056.446	$21252_{2}^{{}^{\circ}}$ – 33309_2_
8291.869	1		12056.693	$19039_{2}^{{}^{\circ}}$ – 31095_1_
8291.523	2		12057.196	17959_4_ – $30017_{3}^{{}^{\circ}}$
8290.864	2		12058.155	13297_4_ – $25355_{4}^{{}^{\circ}}$
8289.447	2		12060.216	14226_0_ – $26287_{1}^{{}^{\circ}}$
8288.404	50	5	12061.733	$16783_{4}^{{}^{\circ}}$ – 28845_4_
8287.110	25	3	12063.617	$18069_{3}^{{}^{\circ}}$ – 30132_2_
8287.023	8		12063.744	$17354_{1}^{{}^{\circ}}$ – 29418_2_
8284.481	3		12067.445	$17354_{1}^{{}^{\circ}}$ – 29422_1_
8276.232	20	2	12079.473	$14032_{2}^{{}^{\circ}}$ – 26111_1_
8275.628	400	150	12080.354	$11197_{0}^{{}^{\circ}}$ – 23277_5_
8273.609	8	1	12083.302	17073_1_ – $29157_{1}^{{}^{\circ}}$
8270.940	3	1	12087.202	21575_2_ – $33662_{1}^{{}^{\circ}}$
8267.412	2		12092.360	$22338_{3}^{{}^{\circ}}$ – 34431_3_
8263.926	150	20	12097.460	9804_5_ – $21902_{4}^{{}^{\circ}}$
8261.010	100	25	12101.731	$15490_{0}^{{}^{\circ}}$ – 27591_5_
8260.391	1		12102.638	$19039_{2}^{{}^{\circ}}$ – 31141_3_
8259.505	100	8	12103.936	8111_4_ – $20214_{3}^{{}^{\circ}}$
8258.339	8	2	12105.645	$22338_{3}^{{}^{\circ}}$ – 34444_2_
8254.741	100	15	12110.921	$10526_{3}^{{}^{\circ}}$ – 22637_3_
8253.613	75	8	12112.576	$11877_{1}^{{}^{\circ}}$ – 23990_2_
8252.398	400	50	12114.360	0_2_ – $12114_{2}^{{}^{\circ}}$
8246.170	2h		12123.509	17073_1_ – $29197_{1}^{{}^{\circ}}$
8246.011	25	4	12123.743	21594_3_ – $33718_{2}^{{}^{\circ}}$
8245.781	2		12124.081	17073_1_ – $29197_{2}^{{}^{\circ}}$
8243.712	2		12127.124	21143_5_ – $33270_{4}^{{}^{\circ}}$
8239.619	5	1	12133.148	12847_3_ – $24981_{3}^{{}^{\circ}}$
8237.739	20	2	12135.917	$20322_{0}^{{}^{\circ}}$ – 32458_4_
8236.739	4		12137.390	$21077_{0}^{{}^{\circ}}$ – 33214_5_
8235.819	4	1	12138.746	19532_4_ – $31671_{4}^{{}^{\circ}}$
8235.484	1		12139.240	$23655_{4}^{{}^{\circ}}$ – 35794_4_
8234.937	50	4	12140.046	11601_1_ – $23741_{1}^{{}^{\circ}}$
8234.937	50	4	12140.046	21594_3_ – $33734_{2}^{{}^{\circ}}$
8234.346	25	3	12140.918	$16217_{2}^{{}^{\circ}}$ – 28358_3_
8233.377	2		12142.347	20054_2_ – $32197_{3}^{{}^{\circ}}$
8232.673	1		12143.385	21575_2_ – $33718_{2}^{{}^{\circ}}$
8232.451	3		12143.712	$18614_{1}^{{}^{\circ}}$ – 30758_2_
8232.310	2		12143.920	$21165_{3}^{{}^{\circ}}$ – 33309_2_
8231.410	100	15	12145.248	13297_4_ – $25442_{3}^{{}^{\circ}}$
8228.725	1		12149.211	18574_1_ – $30723_{1}^{{}^{\circ}}$
8227.672	50	4	12150.766	13962_1_ – $26113_{2}^{{}^{\circ}}$
8227.482	50	8	12151.046	$16783_{4}^{{}^{\circ}}$ –28934_3_
8227.096	3		12151.617	11601_1_ – $23752_{2}^{{}^{\circ}}$
8224.956	8	2	12154.778	21645_4_ – $33800_{3}^{{}^{\circ}}$
8223.407	8		12157.068	19713_3_ – $31870_{2}^{{}^{\circ}}$
8221.666	1		12159.642	21575_2_ – $33734_{2}^{{}^{\circ}}$
8220.872	3		12160.817	15863_2_ – $28024_{1}^{{}^{\circ}}$
8214.147	100	10	12170.773	15970_3_ – $28140_{4}^{{}^{\circ}}$
8212.219	5	1	12173.630	$14206_{4}^{{}^{\circ}}$ – 26380_5_
8210.525	2		12176.142	$23655_{4}^{{}^{\circ}}$ – 35831_3_
8210.047	4		12176.851	$15166_{3}^{{}^{\circ}}$ – 27343_3_
8209.444	100	8	12177.745	15493_4_ – $27670_{3}^{{}^{\circ}}$
8208.596	2		12179.003	17073_1_ – $29252_{2}^{{}^{\circ}}$
8208.596	2		12179.003	17073_1_ – $29252_{2}^{{}^{\circ}}$
8207.475	100	10	12180.666	14204_5_ – $26384_{4}^{{}^{\circ}}$
8205.892	8		12183.016	$17501_{0}^{{}^{\circ}}$ – 29684_5_
8205.103	20	2	12184.188	19532_4_ – $31716_{0}^{{}^{\circ}}$
8202.148	100	10	12188.577	13847_2_ – $26036_{3}^{{}^{\circ}}$
8201.354	1		12189.757	22401_1_ – $34591_{1}^{{}^{\circ}}$
8198.438	10	2	12194.093	$17224_{2}^{{}^{\circ}}$ – 29418_2_
8195.942	8	1	12197.806	$17224_{2}^{{}^{\circ}}$ – 29422_1_
8194.698	3		12199.658	$22508_{2}^{{}^{\circ}}$ – 34707_3_
8194.398	100	25	12200.105	17959_4_ – $30160_{4}^{{}^{\circ}}$
8194.398	100	25	12200.105	$20867_{7}^{{}^{\circ}}$ – 33068_6_
8193.239	5	1	12201.831	$23015_{0}^{{}^{\circ}}$ – 35216_5_
8192.136	3		12203.473	$22669_{3}^{{}^{\circ}}$ – 34873_4_
8191.069	10	1	12205.063	21594_3_ – $33799_{4}^{{}^{\circ}}$
8190.889	50	8	12205.331	$19588_{0}^{{}^{\circ}}$ – 31793_4_
8190.479	2		12205.942	21594_3_ – $33800_{3}^{{}^{\circ}}$
8189.938	4		12206.749	$19039_{2}^{{}^{\circ}}$ – 31245_2_
8186.915	500	100	12211.256	8111_4_ – $20322_{0}^{{}^{\circ}}$
8186.217	8		12212.297	18549_2_ – $30761_{3}^{{}^{\circ}}$
8183.838	10	2	12215.847	$19986_{6}^{{}^{\circ}}$ – 32202_5_
8183.723	2		12216.019	$16346_{4}^{{}^{\circ}}$ – 28562_4_
8179.030	8	1	12223.028	7280_2_ – $19503_{3}^{{}^{\circ}}$
8178.829	2		12223.329	$10414_{4}^{{}^{\circ}}$ – 22637_3_
8178.318	2		12224.092	$19817_{1}^{{}^{\circ}}$ –32041_2_
8174.704	2		12229.496	$20566_{4}^{{}^{\circ}}$ – 32796_3_
8174.328	2		12230.059	$13175_{4}^{{}^{\circ}}$ – 25405_4_
8174.086	4		12230.421	20054_2_ – $32285_{3}^{{}^{\circ}}$
8172.122	50	3	12233.360	13088_3_ – $25321_{3}^{{}^{\circ}}$
8171.652	2		12234.064	$22855_{3}^{{}^{\circ}}$ – 35089_3_
8169.793	400	50	12236.848	7280_2_ – $19516_{2}^{{}^{\circ}}$
8168.775	5		12238.373	18574_1_ – $30812_{2}^{{}^{\circ}}$
8167.865	3		12239.736	$17411_{3}^{{}^{\circ}}$ – 29650_2_
8163.355	1		12246.498	22098_4_ – $34344_{4}^{{}^{\circ}}$
8162.064	50	3	12248.435	19532_4_ – $31780_{3}^{{}^{\circ}}$
8161.552	2		12249.204	13847_2_ – $26096_{3}^{{}^{\circ}}$
8159.737	400	100	12251.928	6362_2_ – $18614_{4}^{{}^{\circ}}$
8159.016	2		12253.011	$21165_{3}^{{}^{\circ}}$ – 33418_4_
8157.540	50	8	12255.228	$17501_{0}^{{}^{\circ}}$ – 29756_4_
8155.949	5	1	12257.619	$19516_{2}^{{}^{\circ}}$ – 31774_3_
8151.973	2		12263.597	18549_2_ – $30812_{2}^{{}^{\circ}}$
8150.878	1		12265.245	$21902_{4}^{{}^{\circ}}$ – 34167_3_
8150.703	25		12265.508	13847_2_ – $26113_{2}^{{}^{\circ}}$
8149.704	50	4	12267.011	13088_3_ – $25355_{4}^{{}^{\circ}}$
8148.901	3		12268.220	$22163_{4}^{{}^{\circ}}$ – 34431_3_
8146.748	5		12271.462	$19503_{3}^{{}^{\circ}}$ – 31774_3_
8146.237	5		12272.232	$22877_{1}^{{}^{\circ}}$ – 35149_2_
8144.017	4		12275.577	$18614_{1}^{{}^{\circ}}$ – 30889_1_
8143.143	500	300	12276.895	8800_4_ – $21077_{0}^{{}^{\circ}}$
8142.679	20	3	12277.595	13297_4_ – $25575_{4}^{{}^{\circ}}$
8138.474	200	100	12283.938	5563_1_ – $17847_{2}^{{}^{\circ}}$
8137.940	50	4	12284.744	$12114_{2}^{{}^{\circ}}$ – 24399_3_
8137.367	4		12285.609	$17847_{2}^{{}^{\circ}}$ – 30132_2_
8137.256	2		12285.777	$23015_{0}^{{}^{\circ}}$ – 35300_5_
8135.798	2		12287.979	17398_3_ – $29686_{3}^{{}^{\circ}}$
8134.633	5		12289.738	$22141_{3}^{{}^{\circ}}$ – 34431_3_
8134.085	2		12290.566	$19503_{3}^{{}^{\circ}}$ – 31793_4_
8133.855	1		12290.914	18699_2_ – $30990_{3}^{{}^{\circ}}$
8132.964	50	1	12292.260	$21539_{4}^{{}^{\circ}}$ – 33831_4_
8131.517	5	1	12294.448	$22855_{3}^{{}^{\circ}}$ – 35149_2_
8129.681	50	5	12297.224	16351_0_ – $28649_{1}^{{}^{\circ}}$
8129.405	150	25	12297.642	2869_3_ – $15166_{3}^{{}^{\circ}}$
8125.877	4		12302.981	$22141_{3}^{{}^{\circ}}$ – 34444_2_
8122.725	200	50	12307.755	$19986_{6}^{{}^{\circ}}$ – 32293_5_
8121.058	15	1	12310.282	$13945_{3}^{{}^{\circ}}$ – 26255_4_
8117.557	5		12315.591	$18930_{3}^{{}^{\circ}}$ – 31245_2_
8114.539	50	4	12320.171	$16783_{4}^{{}^{\circ}}$ – 29104_4_
8108.929	4	1	12328.695	$15166_{3}^{{}^{\circ}}$ – 27495_4_
8107.508	3		12330.855	$14465_{2}^{{}^{\circ}}$ – 26796_3_
8106.805	2		12331.925	$18809_{4}^{{}^{\circ}}$ – 31141_3_
8104.536	2		12335.377	$18930_{3}^{{}^{\circ}}$ – 31265_3_
8103.669	3	1	12336.697	$20214_{3}^{{}^{\circ}}$ – 32551_3_
8101.873	2		12339.432	$22877_{1}^{{}^{\circ}}$ – 35216_5_
8100.839	1		12341.007	$21077_{0}^{{}^{\circ}}$ – 33418_4_
8097.857	4		12345.551	$19948_{4}^{{}^{\circ}}$ – 32293_5_
8097.483	4		12346.122	17398_3_ – $29744_{3}^{{}^{\circ}}$
8096.253	20	3	12347.997	$17501_{0}^{{}^{\circ}}$ – 29849_4_
8093.631	200	75	12351.997	3865_1_ – $16217_{2}^{{}^{\circ}}$
8092.238	75	5	12354.124	13088_3_ – $25442_{3}^{{}^{\circ}}$
8092.238	75	5	12354.124	18574_1_ – $30928_{1}^{{}^{\circ}}$
8091.356	2		12355.470	21645_4_ – $34001_{4}^{{}^{\circ}}$
8089.896	4		12357.700	$16783_{4}^{{}^{\circ}}$ – 29141_5_
8089.481	75	8	12358.334	9804_5_ – $22163_{4}^{{}^{\circ}}$
8088.701	4		12359.526	15493_4_ – $27852_{0}^{{}^{\circ}}$
8087.495	5	2	12361.369	$18011_{0}^{{}^{\circ}}$ – 30372_6_
8087.263	2		12361.723	$15618_{3}^{{}^{\circ}}$ – 27980_3_
8085.228	200	50	12364.835	8800_4_ – $21165_{3}^{{}^{\circ}}$
8084.451	1		12366.023	$22508_{2}^{{}^{\circ}}$ – 34874_3_
8076.980	5	1	12377.461	15970_3_ – $28347_{2}^{{}^{\circ}}$
8075.651	150	20	12379.498	11802_2_ – $24182_{2}^{{}^{\circ}}$
8074.472	3		12381.306	18431_3_ – $30812_{2}^{{}^{\circ}}$
8070.785	2		12386.962	$20922_{2}^{{}^{\circ}}$ – 33309_2_
8068.729	5	1	12390.118	$19039_{2}^{{}^{\circ}}$ – 31429_1_
8066.826	75	8	12393.041	13297_4_ – $25690_{0}^{{}^{\circ}}$
8064.380	2		12396.800	$20566_{4}^{{}^{\circ}}$ – 32963_5_
8062.640	200	50	12399.475	$19227_{6}^{{}^{\circ}}$ – 31626_5_
8062.345	4		12399.929	$15166_{3}^{{}^{\circ}}$ – 27566_2_
8061.918	3		12400.586	13962_1_ – $26363_{2}^{{}^{\circ}}$
8057.973	50	2	12406.657	21594_3_ – $34001_{4}^{{}^{\circ}}$
8054.929	2		12411.345	$21890_{3}^{{}^{\circ}}$ – 34301_4_
8052.715	5		12414.758	$20322_{0}^{{}^{\circ}}$ – 32737_5_
8051.486	1		12416.653	21143_5_ – $33560_{4}^{{}^{\circ}}$
8050.067	5	1	12418.841	$10783_{2}^{{}^{\circ}}$ – 23201_3_
8048.611	2		12421.088	19532_4_ – $31953_{4}^{{}^{\circ}}$
8046.838	5		12423.825	$17411_{3}^{{}^{\circ}}$ – 29835_3_
8046.331	8	3	12424.608	$19588_{0}^{{}^{\circ}}$ – 32012_4_
8045.182	5	1	12426.382	$19503_{3}^{{}^{\circ}}$ – 31929_3_
8044.989	4b		12426.680	$17224_{2}^{{}^{\circ}}$ – 29650_2_
8043.239	1		12429.384	$21738_{2}^{{}^{\circ}}$ – 34167_3_
8038.691	2		12436.416	$21252_{2}^{{}^{\circ}}$ – 33689_2_
8037.699	3		12437.951	$17411_{3}^{{}^{\circ}}$ – 29849_4_
8035.618	50		12441.172	$21165_{3}^{{}^{\circ}}$ – 33606_2_
8032.883	10		12445.408	$15166_{3}^{{}^{\circ}}$ – 27612_3_
8032.433	200	50	12446.105	7502_3_ – $19948_{4}^{{}^{\circ}}$
8031.574	1		12447.436	19713_3_ – $32160_{2}^{{}^{\circ}}$
8030.188	50	4	12449.585	4961_4_ – $17411_{3}^{{}^{\circ}}$
8027.103	3		12454.369	$18053_{4}^{{}^{\circ}}$ – 30508_5_
8026.438	10	1	12455.401	15493_4_ – $27948_{4}^{{}^{\circ}}$
8026.192	50	8	12455.783	$18809_{4}^{{}^{\circ}}$ – 31265_3_
8025.708	50	8	12456.534	13297_4_ – $25753_{0}^{{}^{\circ}}$
8024.235	100	100	12458.821	12847_3_ – $25306_{2}^{{}^{\circ}}$
8023.886	2		12459.363	$20322_{0}^{{}^{\circ}}$ – 32781_4_
8022.733	3		12461.153	$18809_{4}^{{}^{\circ}}$ – 31271_5_
8022.191	75b	25	12461.995	$10526_{3}^{{}^{\circ}}$ – 22988_2_
8014.499	50	3	12473.955	12847_3_ – $25321_{3}^{{}^{\circ}}$
8013.499	15	2	12475.512	$18069_{3}^{{}^{\circ}}$ – 30544_2_
8010.708	50	4	12479.859	$22669_{3}^{{}^{\circ}}$ – 35149_2_
8008.645	2		12483.073	17398_3_ – $29881_{4}^{{}^{\circ}}$
8008.397	5		12483.460	$18069_{3}^{{}^{\circ}}$ – 30552_4_
8008.272	2		12483.655	15863_2_ – $28347_{2}^{{}^{\circ}}$
8007.988	1		12484.098	19713_3_ – $32197_{3}^{{}^{\circ}}$
8006.477	25		12486.454	13088_3_ – $25575_{4}^{{}^{\circ}}$
8006.443	15		12486.507	$20922_{2}^{{}^{\circ}}$ – 33408_3_
8003.446	50	5	12491.182	19532_4_ – $32023_{0}^{{}^{\circ}}$
8002.589	4		12492.520	$19503_{3}^{{}^{\circ}}$ – 31995_4_
8000.033	100	10	12496.511	16554_6_ – $29050_{0}^{{}^{\circ}}$
7999.045	2		12498.055	$19039_{2}^{{}^{\circ}}$ – 31537_3_
7999.009	4		12498.111	$19039_{2}^{{}^{\circ}}$ – 31537_3_
7998.488	2		12498.925	$16346_{4}^{{}^{\circ}}$ – 28845_4_
7996.963	40	3	12501.309	18574_1_ – $31075_{2}^{{}^{\circ}}$
7994.709	3		12504.833	11802_2_ – $24307_{2}^{{}^{\circ}}$
7993.676	150	8	12506.449	$10526_{3}^{{}^{\circ}}$ – 23032_4_
7993.253	15	1	12507.111	$18382_{0}^{{}^{\circ}}$ – 30889_1_
7992.936	10	2	12507.607	12847_3_ – $25355_{4}^{{}^{\circ}}$
7992.936	10	2	12507.607	19273_2_ – $31780_{3}^{{}^{\circ}}$
7992.156	50	3	12508.828	15863_2_ – $28372_{1}^{{}^{\circ}}$
7991.515	4h		12509.831	$19503_{3}^{{}^{\circ}}$ – 32012_4_
7991.515	4h		12509.831	$19948_{4}^{{}^{\circ}}$ – 32458_4_
7991.366	50	5	12510.064	15970_3_ – $28480_{4}^{{}^{\circ}}$
7989.163	8	1	12513.514	$12114_{2}^{{}^{\circ}}$ – 24627_1_
7987.974	200	50	12515.377	$14481_{6}^{{}^{\circ}}$ – 26997_6_
7986.904	1		12517.053	$18809_{4}^{{}^{\circ}}$ – 31326_4_
7984.603	1		12520.660	20054_2_ – $32575_{2}^{{}^{\circ}}$
7984.032	1		12521.556	$21645_{4}^{{}^{\circ}}$ – 34167_3_
7982.495	10	2	12523.967	$21165_{3}^{{}^{\circ}}$ – 33689_2_
7982.289	8	2	12524.290	$19516_{2}^{{}^{\circ}}$ – 32041_2_
7980.878	2		12526.504	18549_2_ – $31075_{2}^{{}^{\circ}}$
7978.977	500	300	12529.489	3687_2_ – $16217_{2}^{{}^{\circ}}$
7976.870	3		12532.798	$20566_{4}^{{}^{\circ}}$ – 33099_3_
7975.874	2		12534.363	$22338_{3}^{{}^{\circ}}$ – 34873_4_
7974.743	3		12536.141	21575_2_ – $34111_{1}^{{}^{\circ}}$
7974.459	4		12536.587	$20737_{1}^{{}^{\circ}}$ – 33273_1_
7974.164	100	8	12537.051	7280_2_ – $19817_{1}^{{}^{\circ}}$
7972.598	200	20	12539.514	4961_4_ – $17501_{0}^{{}^{\circ}}$
7970.254	50	5	12543.201	15970_3_ – $28513_{2}^{{}^{\circ}}$
7969.785	10	1	12543.940	$15490_{0}^{{}^{\circ}}$ – 28034_5_
7969.359	4		12544.610	$22163_{4}^{{}^{\circ}}$ – 34707_3_
7968.784	2		12545.515	17166_5_ – $29711_{0}^{{}^{\circ}}$
7966.264	2		12549.484	$20724_{0}^{{}^{\circ}}$ – 33273_1_
7965.711	8	1	12550.355	$12114_{2}^{{}^{\circ}}$ – 24664_3_
7961.994	100	8	12556.214	$21165_{3}^{{}^{\circ}}$ – 33721_2_
7961.343	50	8	12557.241	17959_4_ – $30517_{0}^{{}^{\circ}}$
7961.158	50	4	12557.533	$12114_{2}^{{}^{\circ}}$ – 24671_2_
7960.474	1		12558.612	$21902_{4}^{{}^{\circ}}$ – 34460_5_
7955.709	4	1	12566.133	$22141_{3}^{{}^{\circ}}$ – 34707_3_
7955.491	2		12566.478	17073_1_ – $29640_{1}^{{}^{\circ}}$
7955.066	75	4	12567.149	$23015_{0}^{{}^{\circ}}$ – 35582_5_
7954.592	150	15	12567.898	6362_2_ – $18930_{3}^{{}^{\circ}}$
7952.148	3		12571.760	$20737_{1}^{{}^{\circ}}$ – 33309_2_
7951.885	8	1	12572.176	19713_3_ – $32285_{3}^{{}^{\circ}}$
7949.019	3	1	12576.709	$20522_{2}^{{}^{\circ}}$ – 33099_3_
7947.930	4		12578.432	11802_2_ – $24381_{2}^{{}^{\circ}}$
7946.113	100	8	12581.309	$22508_{2}^{{}^{\circ}}$ – 35089_3_
7946.074	2		12581.370	11601_1_ – $24182_{2}^{{}^{\circ}}$
7945.611	3		12582.103	$18069_{3}^{{}^{\circ}}$ – 30651_3_
7944.708	2		12583.534	18699_2_ – $31283_{3}^{{}^{\circ}}$
7943.393	2		12585.617	$22855_{3}^{{}^{\circ}}$ – 35440_3_
7943.050	20	2	12586.160	14204_5_ – $26790_{4}^{{}^{\circ}}$
7941.721	200	75	12588.266	$16346_{4}^{{}^{\circ}}$ – 28934_3_
7941.164	2		12589.149	$14206_{4}^{{}^{\circ}}$ – 26796_3_
7937.727	100	20	12594.600	9804_5_ – $22399_{0}^{{}^{\circ}}$
7936.762	4		12596.132	20054_2_ – $32650_{1}^{{}^{\circ}}$
7932.280	3	1	12603.249	$19948_{4}^{{}^{\circ}}$ – 32551_3_
7929.938	5		12606.971	$18930_{3}^{{}^{\circ}}$ – 31537_3_
7929.809	25c	2	12607.176	$17354_{1}^{{}^{\circ}}$ – 29961_1_
7928.680	3		12608.971	$15618_{3}^{{}^{\circ}}$ – 28227_4_
7927.546	25	3	12610.775	20054_2_ – $32665_{1}^{{}^{\circ}}$
7925.734	50	3	12613.658	$19588_{0}^{{}^{\circ}}$ – 32202_5_
7924.977	50	4	12614.863	13088_3_ – $25703_{2}^{{}^{\circ}}$
7922.912	8	2	12618.151	11802_2_ – $24421_{3}^{{}^{\circ}}$
7922.549	50	5	12618.729	17398_3_ – $30017_{3}^{{}^{\circ}}$
7922.235	15		12619.229	15970_3_ – $28589_{3}^{{}^{\circ}}$
7917.236	15	1	12627.197	22098_4_ – $34725_{3}^{{}^{\circ}}$
7916.790	40	5	12627.908	$21539_{4}^{{}^{\circ}}$ – 34167_3_
7914.493	8	2	12631.573	$18614_{1}^{{}^{\circ}}$ – 31245_2_
7908.484	8	2	12641.171	$20322_{0}^{{}^{\circ}}$ – 32963_5_
7908.148	50	5	12641.708	$22508_{2}^{{}^{\circ}}$ – 35149_2_
7907.856	4		12642.175	$23655_{4}^{{}^{\circ}}$ – 36297_4_
7904.411	50	5	12647.685	15493_4_ – $28140_{4}^{{}^{\circ}}$
7904.294	4		12647.872	$20566_{4}^{{}^{\circ}}$ – 33214_5_
7903.319	8	1	12649.432	15863_2_ – $28513_{2}^{{}^{\circ}}$
7900.778	5		12653.500	$20737_{1}^{{}^{\circ}}$ – 33390_1_
7900.309	200	75	12654.251	$15618_{3}^{{}^{\circ}}$ – 28273_2_
7899.018	8	1	12656.320	21143_5_ – $33799_{4}^{{}^{\circ}}$
7896.541	5		12660.290	13847_2_ – $26508_{3}^{{}^{\circ}}$
7893.614	50	5	12664.984	$16217_{2}^{{}^{\circ}}$ – 28882_2_
7892.770	8		12666.338	$20724_{0}^{{}^{\circ}}$ – 33390_1_
7886.273	4001	50	12676.773	6362_2_ – $19039_{2}^{{}^{\circ}}$
7883.595	4		12681.080	$23655_{4}^{{}^{\circ}}$ – 36336_3_
7881.667	2		12684.182	$20922_{2}^{{}^{\circ}}$ – 33606_2_
7880.577	5		12685.936	$22163_{4}^{{}^{\circ}}$ – 34849_5_
7878.483	5		12689.308	13962_1_ – $26651_{2}^{{}^{\circ}}$
7869.771	15	2	12703.355	15970_3_ – $28673_{2}^{{}^{\circ}}$
7868.924	40	10	12704.722	$22877_{1}^{{}^{\circ}}$ – 35582_5_
7868.396	75	10h	12705.575	$19588_{0}^{{}^{\circ}}$ – 32293_5_
7867.683	401	3	12706.726	11601_1_ – $24307_{2}^{{}^{\circ}}$
7865.954	400	75	12709.519	$14481_{6}^{{}^{\circ}}$ – 27191_5_
7865.248	10	2	12710.660	15970_3_ – $28680_{4}^{{}^{\circ}}$
7865.077	8		12710.936	$22163_{4}^{{}^{\circ}}$ — 34874_3_
7864.009	100	8	12712.663	7502_3_ – $20214_{3}^{{}^{\circ}}$
7862.336	15	3	12715.368	17166_5_ – $29881_{4}^{{}^{\circ}}$
7858.703	2		12721.246	$22855_{3}^{{}^{\circ}}$ – 35576_3_
7858.583	2		12721.440	$17411_{3}^{{}^{\circ}}$ – 30132_2_
7855.074	3		12727.123	12847_3_ – $25575_{4}^{{}^{\circ}}$
7854.919	8		12727.374	$18809_{4}^{{}^{\circ}}$ – 31537_3_
7853.645	2		12729.439	21594_3_ – $34324_{2}^{{}^{\circ}}$
7853.284	20		12730.024	$20543_{0}^{{}^{\circ}}$ – 33273_1_
7852.227	1		12731.738	$22141_{3}^{{}^{\circ}}$ – 34873_4_
7851.006	3		12733.718	18549_2_ – $31283_{3}^{{}^{\circ}}$
7848.645	5h		12737.548	$17224_{2}^{{}^{\circ}}$ – 29961_1_
7848.441	100	40	12737.879	$15490_{0}^{{}^{\circ}}$ – 28227_4_
7847.784	100b	40	12738.946	13297_4_ – $26036_{3}^{{}^{\circ}}$
7847.536	500b	150	12739.348	8800_4_ – $21539_{4}^{{}^{\circ}}$
7843.545	4	1	12745.830	$20322_{0}^{{}^{\circ}}$ – 33068_6_
7842.254	100	10	12747.928	$13175_{4}^{{}^{\circ}}$ – 25923_4_
7841.781	200	50	12748.697	$11241_{3}^{{}^{\circ}}$ – 23990_2_
7841.596	5		12748.998	21575_2_ – 34324_2_
7841.134	8		12749.749	2869_3_ – $15618_{3}^{{}^{\circ}}$
7840.992	8		12749.980	21594_3_ – 34344_1_
7840.445	100	75	12750.870	$19986_{6}^{{}^{\circ}}$ – 32737_5_
7840.290	100	50	12751.122	13297_4_ – $26048_{4}^{{}^{\circ}}$
7839.276	10	2	12752.771	19532_4_ – $32285_{3}^{{}^{\circ}}$
7838.062	2		12754.746	$21077_{0}^{{}^{\circ}}$ – 3383 ***U***
7836.456	100	5	12757.360	16346_1_ – 29104_4_
7835.620	100		12758.721	11802_2_ – $24561_{3}^{{}^{\circ}}$
7828.073	3		12771.022	$22669_{0}^{{}^{\circ}}$ – 35440_3_
7825.245	2		12775.637	$21668_{1}^{{}^{\circ}}$ – 34444_2_
7824.291	1		12777.195	21594_3_ – $34371_{2}^{{}^{\circ}}$
7823.787	2		12778.018	$17354_{1}^{{}^{\circ}}$ – 30132_2_
7823.475	50	2	12778.528	$22855_{3}^{{}^{\circ}}$ – 35633_4_
7822.392	100	4	12780.297	11601_1_ – $24381_{2}^{{}^{\circ}}$
7819.888	2		12784.389	21645_4_ – $34430_{3}^{{}^{\circ}}$
7819.350	3		12785.269	15863_2_ – 28649!
7818.694	2		12786.342	$20522_{2}^{{}^{\circ}}$ – 33309_2_
7817.771	500	200	12787.851	$10414_{4}^{{}^{\circ}}$ – 23201_3_
7817.130	4		12788.900	13088_3_ – $25877_{4}^{{}^{\circ}}$
7816.135	50	4	12790.528	$11241_{3}^{{}^{\circ}}$ – 24032_4_
7813.970	50	5	12794.072	$11877_{1}^{{}^{\circ}}$ – 24671_2_
7813.970	50	5	12794.072	$15736_{1}^{{}^{\circ}}$ – 28531_2_
7813.481	50	8	12794.872	$16346_{4}^{{}^{\circ}}$ – 29141_5_
7810.627	100	10	12799.547	13297_4_ – $26096_{3}^{{}^{\circ}}$
7809.950	2		12800.657	$12114_{2}^{{}^{\circ}}$ – 24915_3_
7809.245	25b	2	12801.813	17959_4_ – $30761_{3}^{{}^{\circ}}$
7807.879	40		12804.052	13847_2_ – $26651_{3}^{{}^{\circ}}$
7804.533	50	5	12809.542	15863_2_ – $28673_{2}^{{}^{\circ}}$
7804.146	50	4	12810.177	17398_3_ – $30208_{2}^{{}^{\circ}}$
7803.811	3	1	12810.727	$22338_{3}^{{}^{\circ}}$ – 35149_2_
7802.824	10	2	12812.347	15863_2_ – $28676_{3}^{{}^{\circ}}$
7801.945	2		12813.791	$15166_{3}^{{}^{\circ}}$ – 27980_3_
7800.029	3		12816.938	$18809_{4}^{{}^{\circ}}$ – 31626_5_
7799.629	8	1	12817.596	$21890_{3}^{{}^{\circ}}$ – 34707_3_
7799.629	8	1	12817.596	$22399_{0}^{{}^{\circ}}$ – 35216_5_
7798.360	300	25	12819.681	5563_1_ – $18382_{0}^{{}^{\circ}}$
7795.533	1		12824.330	18699_2_ – $31523_{3}^{{}^{\circ}}$
7793.128	10	2	12828.288	17959_4_ – $30788_{0}^{{}^{\circ}}$
7790.132	10		12833.221	$19948_{4}^{{}^{\circ}}$ – 32781_4_
7788.932	500	75	12835.199	$11197_{0}^{{}^{\circ}}$ – 24032_4_
7787.659	25		12837.297	$14206_{4}^{{}^{\circ}}$ – 27044_3_
7785.771	2h		12840.410	$22248_{2}^{{}^{\circ}}$ – 35089_3_
7784.779	3		12842.046	$20566_{4}^{{}^{\circ}}$ – 33408_3_
7783.417	5	1	12844.293	$18930_{3}^{{}^{\circ}}$ – 31774_3_
7782.742	4	1	12845.407	16351_0_ – $29197_{1}^{{}^{\circ}}$
7782.318	150	50	12846.107	21645_4_ – $34492_{4}^{{}^{\circ}}$
7781.843	100	4	12846.891	$20543_{0}^{{}^{\circ}}$ – 33390_1_
7781.329	2		12847.740	$19948_{4}^{{}^{\circ}}$ – 32796_3_
7779.741	8	2	12850.362	$20423_{1}^{{}^{\circ}}$ – 33273_1_
7779.472	2		12850.806	$13945_{3}^{{}^{\circ}}$ – 26796_3_
7778.488	50	5	12852.432	$15490_{0}^{{}^{\circ}}$ – 28342_5_
7776.804	4		12855.215	21575_2_ – $34430_{3}^{{}^{\circ}}$
7776.671	40	2	12855.435	12847_3_ – $25703_{2}^{{}^{\circ}}$
7775.688	2		12857.060	17398_3_ – $30255_{3}^{{}^{\circ}}$
7773.389	3		12860.863	$23655_{4}^{{}^{\circ}}$ – 36515_3_
7772.457	2		12862.405	19713_3_ – $32575_{2}^{{}^{\circ}}$
7771.942	100	50	12863.257	$10414_{4}^{{}^{\circ}}$ – 23277_5_
7769.052	2		12868.042	$20522_{2}^{{}^{\circ}}$ – 33390_1_
7768.470	2		12869.006	$20737_{1}^{{}^{\circ}}$ – 33606_2_
7767.963	3		12869.846	$19588_{0}^{{}^{\circ}}$ – 32458_4_
7766.941	4		12871.540	$17501_{0}^{{}^{\circ}}$ – 30372_6_
7762.722	50	3	12878.535	$14465_{2}^{{}^{\circ}}$ – 27343_3_
7759.127	2		12884.502	$20214_{3}^{{}^{\circ}}$ – 33099_3_
7758.486	3		12885.566	$20423_{1}^{{}^{\circ}}$ – 33309_2_
7758.251	3h		12885.957	$20522_{2}^{{}^{\circ}}$ – 33408_3_
7755.600	4		12890.362	$19039_{2}^{{}^{\circ}}$ – 31929_3_
7754.748	1		12891.778	$21539_{4}^{{}^{\circ}}$ – 34431_3_
7754.433	1		12892.301	$20322_{0}^{{}^{\circ}}$ – 33214_5_
7754.271	2		12892.571	$20566_{4}^{{}^{\circ}}$ – 33459_4_
7752.440	3		12895.616	$18069_{3}^{{}^{\circ}}$ – 30964_3_
7751.435	5		12897.288	$22508_{2}^{{}^{\circ}}$ – 35405_3_
7749.578	5		12900.378	$16783_{4}^{{}^{\circ}}$ – 29684_5_
7749.325	8	2	12900.799	$22248_{2}^{{}^{\circ}}$ – 35149_2_
7748.880	3		12901.540	$22399_{0}^{{}^{\circ}}$ – 35300_5_
7745.813	15	3	12906.649	19532_4_ – $32439_{4}^{{}^{\circ}}$
7745.235	3		12907.612	$21539_{4}^{{}^{\circ}}$ – 34447_4_
7744.786	4		12908.360	$17224_{2}^{{}^{\circ}}$ – 30132_2_
7742.552	100	2	12912.085	$15618_{3}^{{}^{\circ}}$ – 28531_2_
7742.019	2		12912.974	$22508_{2}^{{}^{\circ}}$ – 35421_1_
7740.867	15	1	12914.895	$21252_{2}^{{}^{\circ}}$ – 34167_3_
7739.704	2		12916.836	$22877_{1}^{{}^{\circ}}$ – 35794_4_
7738.356	5	1	12919.086	$18011_{0}^{{}^{\circ}}$ – 30930_6_
7737.779	4		12920.049	13962_1_ – $26882_{0}^{{}^{\circ}}$
7736.364	2		12922.412	$22877_{1}^{{}^{\circ}}$ – 35799_6_
7735.479	2		12923.891	19273_2_ – $32197_{3}^{{}^{\circ}}$
7733.362	2		12927.429	22401_1_ – $35328_{1}^{{}^{\circ}}$
7730.107	5	1	12932.872	$22508_{2}^{{}^{\circ}}$ – 35440_3_
7728.939	100	15	12934.827	7280_2_ – $20214_{3}^{{}^{\circ}}$
7727.552	2		12937.148	21645_4_ – $34583_{3}^{{}^{\circ}}$
7726.384	2		12939.104	$22855_{3}^{{}^{\circ}}$ – 35794_4_
7723.641	3		12943.699	$15618_{3}^{{}^{\circ}}$ – 28562_4_
7721.780	2		12946.819	$21902_{4}^{{}^{\circ}}$ – 34849_5_
7721.188	100	8	12947.811	$22141_{3}^{{}^{\circ}}$ – 35089_3_
7718.812	3		12951.797	$20737_{1}^{{}^{\circ}}$ – 33689_2_
7713.337	4		12960.990	$20566_{4}^{{}^{\circ}}$ – 33527_4_
7712.394	50	4	12962.575	15970_3_ – $28932_{4}^{{}^{\circ}}$
7711.113	40	3	12964.728	$18809_{4}^{{}^{\circ}}$ – 31774_3_
7710.258	100	15	12966.166	8111_4_ – $21077_{0}^{{}^{\circ}}$
7709.882	50		12966.798	11802_2_ – $24769_{3}^{{}^{\circ}}$
7708.149	2		12969.714	$21738_{2}^{{}^{\circ}}$ – 34707_3_
7706.909	1		12971.800	$21902_{4}^{{}^{\circ}}$ – 34874_3_
7706.448	3		12972.576	$16783_{4}^{{}^{\circ}}$ – 29756_4_
7705.268	2		12974.563	18549_2_ – $31523_{3}^{{}^{\circ}}$
7703.679	100	10	12977.239	$19986_{6}^{{}^{\circ}}$ – 32963_5_
7699.765	100	5	12983.836	$18809_{4}^{{}^{\circ}}$ – 31793_4_
7699.660	10	1	12984.013	$20737_{1}^{{}^{\circ}}$ – 33721_2_
7699.399	75	1	12984.453	$14206_{4}^{{}^{\circ}}$ – 27191_5_
7698.035	20b		12986.754	$10783_{2}^{{}^{\circ}}$ – 23769_1_
7697.922	75		12986.944	15493_4_ – $28480_{4}^{{}^{\circ}}$
7697.077	2		12988.370	21594_3_ – $34583_{3}^{{}^{\circ}}$
7693.797	100	5	12993.907	17166_5_ – $30160_{4}^{{}^{\circ}}$
7690.658	5		12999.211	$18930_{3}^{{}^{\circ}}$ – 31929_3_
7689.993	1		13000.335	$22877_{1}^{{}^{\circ}}$ – 35877_2_
7688.949	3		13002.100	$19039_{2}^{{}^{\circ}}$ – 32041_2_
7686.149	50	1	13006.836	$17501_{0}^{{}^{\circ}}$ – 30508_5_
7683.150	3b		13011.913	19273_2_ – $32285_{3}^{{}^{\circ}}$
7683.010	50	1	13012.150	$14032_{2}^{{}^{\circ}}$ – 27044_3_
7682.438	3		13013.119	18699_2_ – $31712_{1}^{{}^{\circ}}$
7681.318	3		13015.017	$19948_{4}^{{}^{\circ}}$ – 32963_5_
7678.123	150	5	13020.432	7502_3_ – $20522_{2}^{{}^{\circ}}$
7676.888	2		13022.527	$22855_{3}^{{}^{\circ}}$ – 35877_2_
7675.925	2		13024.161	$22396_{1}^{{}^{\circ}}$ – 35421_1_
7675.621	3		13024.677	13088_3_ – $26113_{2}^{{}^{\circ}}$
7675.621	3		13024.677	18574_1_ – $31599_{2}^{{}^{\circ}}$
7672.749	2		13029.552	12847_3_ – $25877_{4}^{{}^{\circ}}$
7672.252	50	2	13030.396	13847_2_ – $26878_{3}^{{}^{\circ}}$
7672.123	1		13030.615	17959_4_ – $30990_{3}^{{}^{\circ}}$
7669.750	8		13034.647	$19516_{2}^{{}^{\circ}}$ – 32551_3_
7668.955	50	1	13035.998	11802_2_ – $24838_{1}^{{}^{\circ}}$
7667.018	2		13039.291	21143_5_ – $34182_{0}^{{}^{\circ}}$
7665.734	8		13041.475	19713_3_ – $32754_{3}^{{}^{\circ}}$
7665.344	2		13042.139	$20522_{2}^{{}^{\circ}}$ – 33564_3_
7661.621	8	1	13048.476	$19503_{3}^{{}^{\circ}}$ – 32551_3_
7661.162	2		13049.258	$20724_{0}^{{}^{\circ}}$ – 33773_1_
7660.889	150	4	13049.723	4961_4_ – $18011_{0}^{{}^{\circ}}$
7660.029	75	1	13051.188	5563_1_ – $18614_{1}^{{}^{\circ}}$
7660.029	75	1	13051.188	$16783_{4}^{{}^{\circ}}$ – 29835_3_
7658.323	150	5	13054.096	8111_4_ – $21165_{3}^{{}^{\circ}}$
7655.702	8		13058.565	21645_4_ – $34704_{3}^{{}^{\circ}}$
7655.307	2		13059.239	17959_4_ – $31019_{4}^{{}^{\circ}}$
7654.695	100	5	13060.283	$15618_{3}^{{}^{\circ}}$ – 28679_2_
7653.827	200	10	13061.764	14204_5_ – $27266_{4}^{{}^{\circ}}$
7653.327	1		13062.617	$17224_{2}^{{}^{\circ}}$ – 30286_1_
7652.317	100	3	13064.341	7502_3_ – $20566_{4}^{{}^{\circ}}$
7651.739	100	2	13065.328	$16783_{4}^{{}^{\circ}}$ – 29849_4_
7651.143	1		13066.346	$22338_{3}^{{}^{\circ}}$ – 35405_3_
7650.996	75	1	13066.597	$19227_{6}^{{}^{\circ}}$ – 32293_5_
7649.891	2		13068.484	$22508_{2}^{{}^{\circ}}$ – 35576_3_
7648.545	4		13070.784	14226_0_ – $27297_{1}^{{}^{\circ}}$
7647.380	500	50	13072.775	9804_5_ – $22877_{0}^{{}^{\circ}}$
7647.380	500	50	13072.775	$18069_{3}^{{}^{\circ}}$ – 31141_3_
7646.337	2		13074.558	$23015_{0}^{{}^{\circ}}$ – 36089_4_
7644.737	25	1	13077.295	11802_2_ – $24880_{1}^{{}^{\circ}}$
7643.413	1		13079.560	21645_4_ – $34725_{3}^{{}^{\circ}}$
7642.873	15		13080.484	$13175_{4}^{{}^{\circ}}$ – 26255_4_
7642.425	2		13081.251	18699_2_ – $31780_{3}^{{}^{\circ}}$
7642.028	10	1	13081.931	$19986_{6}^{{}^{\circ}}$ – 33068_6_
7641.605	2		13082.655	$18930_{3}^{{}^{\circ}}$ – 32012_4_
7641.066	1		13083.578	$20522_{2}^{{}^{\circ}}$ – 33606_2_
7638.777	100	3	13087.498	13297_4_ – $26384_{4}^{{}^{\circ}}$
7637.385	75	2	13089.883	8800_4_ – $21890_{3}^{{}^{\circ}}$
7636.173	100	4	13091.961	4961_4_ – $18053_{4}^{{}^{\circ}}$
7634.919	3		13094.111	$20214_{3}^{{}^{\circ}}$ – 33309_2_
7633.767	75	2	13096.087	15493_4_ – $28589_{3}^{{}^{\circ}}$
7632.129	50	2	13098.898	13962_1_ – $27061_{2}^{{}^{\circ}}$
7632.129	50	2	13098.898	$13945_{3}^{{}^{\circ}}$ – 27044_3_
7630.536	50	2	13101.633	$14465_{2}^{{}^{\circ}}$ – 27566_2_
7630.310	100	5	13102.021	8800_4_ – $21902_{4}^{{}^{\circ}}$
7627.428	3		13106.971	20054_2_ – $33161_{1}^{{}^{\circ}}$
7627.428	3		13106.971	20054_2_ – $33161_{1}^{{}^{\circ}}$
7627.175	150	5	13107.406	4961_4_ – $18069_{3}^{{}^{\circ}}$
7625.705	150	5	13109.933	$14481_{6}^{{}^{\circ}}$ – 27591_5_
7625.120	50	2	13110.938	$18930_{3}^{{}^{\circ}}$ – 32041_2_
7620.815	5		13118.345	$23015_{0}^{{}^{\circ}}$ – 36133_5_
7620.443	3		13118.985	17398_3_ – $30517_{4}^{{}^{\circ}}$
7620.080	100	5	13119.610	$18809_{4}^{{}^{\circ}}$ – 31929_3_
7617.255	2		13124.476	$22669_{3}^{{}^{\circ}}$ – 35794_4_
7616.686	50	1	13125.456	13962_1_ – $27087_{1}^{{}^{\circ}}$
7614.421	1		13129.360	21575_2_ – $34704_{3}^{{}^{\circ}}$
7612.122	4	1	13133.326	$17411_{3}^{{}^{\circ}}$ – 30544_2_
7611.293	8		13134.756	17073_1_ – $30208_{2}^{{}^{\circ}}$
7610.378	2		13136.335	$21165_{3}^{{}^{\circ}}$ – 34301_4_
7610.101	5		13136.813	$14206_{4}^{{}^{\circ}}$ – 27343_3_
7609.546	2		13137.772	$22163_{4}^{{}^{\circ}}$ – 35300_5_
7609.359	3		13138.094	18574_1_ – $31712_{1}^{{}^{\circ}}$
7607.824	100	4	13140.745	6362_2_ – $19503_{3}^{{}^{\circ}}$
7607.519	40	1	13141.272	$17411_{3}^{{}^{\circ}}$ – 30552_4_
7606.314	5		13143.354	7280_2_ – $20423_{1}^{{}^{\circ}}$
7605.083	5		13145.481	$15736_{1}^{{}^{\circ}}$ – 28882_2_
7604.177	3		13147.048	$14465_{2}^{{}^{\circ}}$ – 27612_3_
7603.623	40	1	13148.005	13847_2_ – $26995_{3}^{{}^{\circ}}$
7603.249	40	1	13148.652	$19588_{0}^{{}^{\circ}}$ – 32737_5_
7602.795	40	1	13149.437	19713_3_ – $32862_{4}^{{}^{\circ}}$
7602.247	2		13150.385	21575_2_ – $34725_{3}^{{}^{\circ}}$
7601.880	5		13151.020	$19948_{4}^{{}^{\circ}}$ – 33099_3_
7600.637	2		13153.171	$23655_{4}^{{}^{\circ}}$ – 36808_3_
7599.815	2		13154.593	6362_2_ – $19516_{2}^{{}^{\circ}}$
7599.638	5		13154.900	17398_3_ – $30553_{2}^{{}^{\circ}}$
7598.203	200	3	13157.384	16554_6_ – $29711_{0}^{{}^{\circ}}$
7598.203	200	3	13157.384	$11241_{3}^{{}^{\circ}}$ – 24399_3_
7591.999	10		13168.136	$21539_{4}^{{}^{\circ}}$ – 34707_3_
7586.988	8		13176.833	$18069_{3}^{{}^{\circ}}$ – 31245_2_
7586.207	20		13178.190	11802_2_ – $24981_{3}^{{}^{\circ}}$
7585.783	200	10	13178.926	2558_0_ – $15736_{1}^{{}^{\circ}}$
7585.688	200	15	13179.091	16554_6_ – $29733_{0}^{{}^{\circ}}$
7583.574	2		13182.765	$20423_{1}^{{}^{\circ}}$ – 33606_2_
7583.534	2		13182.835	$20423_{1}^{{}^{\circ}}$ – 33606_2_
7583.422	25		13183.029	15493_4_ – $28676_{3}^{{}^{\circ}}$
7581.856	4		13185.752	$18809_{4}^{{}^{\circ}}$ – 31995_4_
7581.088	2		13187.088	$21902_{4}^{{}^{\circ}}$ – 35089_3_
7580.866	4		13187.474	15493_4_ – $28680_{4}^{{}^{\circ}}$
7580.829	5		13187.539	15493_4_ – $28680_{4}^{{}^{\circ}}$
7580.686	40	1	13187.787	15970_3_ – $29157_{3}^{{}^{\circ}}$
7580.340	50	1	13188.389	12847_3_ – $26036_{3}^{{}^{\circ}}$
7579.456	3		13189.928	$17354_{1}^{{}^{\circ}}$ – 30544_2_
7578.274	2		13191.985	$21252_{2}^{{}^{\circ}}$ – 34444_2_
7578.274	2		13191.985	$22248_{2}^{{}^{\circ}}$ – 35440_3_
7578.274	2		13191.985	$22855_{3}^{{}^{\circ}}$ – 36047_2_
7577.550	25		13193.245	$19588_{0}^{{}^{\circ}}$ – 32781_4_
7577.266	20		13193.740	$20214_{3}^{{}^{\circ}}$ – 33408_3_
7574.484	5		13198.585	$20522_{2}^{{}^{\circ}}$ – 33721_2_
7574.116	3		13199.227	$21890_{3}^{{}^{\circ}}$ – 35089_3_
7573.342	50	1	13200.576	12847_3_ – $26048_{4}^{{}^{\circ}}$
7573.155	3		13200.902	$16217_{2}^{{}^{\circ}}$ – 29418_2_
7572.973	4		13201.219	21143_5_ – $34344_{4}^{{}^{\circ}}$
7571.931	3		13203.036	$18809_{4}^{{}^{\circ}}$ – 32012_4_
7571.029	8		13204.609	$16217_{2}^{{}^{\circ}}$ – 29422_1_
7570.636	2		13205.294	$20322_{0}^{{}^{\circ}}$ – 33527_4_
7569.509	50	2	13207.260	$10783_{2}^{{}^{\circ}}$ – 23990_2_
7567.739	300	15	13210.349	9804_5_ – $23015_{0}^{{}^{\circ}}$
7567.596	50	3	13210.599	13297_4_ – $26508_{3}^{{}^{\circ}}$
7566.732	3		13212.107	$22877_{0}^{{}^{\circ}}$ – 36089_4_
7565.850	50	2	13213.647	13847_2_ – $27061_{2}^{{}^{\circ}}$
7563.711	5		13217.384	$18053_{4}^{{}^{\circ}}$ – 31271_5_
7557.751	50	1	13227.807	15970_3_ – $29197_{2}^{{}^{\circ}}$
7557.438	2		13228.355	$19986_{6}^{{}^{\circ}}$ – 33214_5_
7556.646	1		13229.742	$20543_{0}^{{}^{\circ}}$ – 33773_1_
7556.309	2		13230.332	19273_2_ – $32503_{2}^{{}^{\circ}}$
7556.225	5		13230.479	$16783_{4}^{{}^{\circ}}$ – 30014_4_
7553.974	10w	2	13234.421	$22399_{0}^{{}^{\circ}}$ – 35633_4_
7553.974	10w	2	13234.421	$22855_{3}^{{}^{\circ}}$ – 36089_4_
7553.753	2		13234.808	17959_4_ – $31194_{4}^{{}^{\circ}}$
7551.997	4		13237.886	11601_1_ – $24838_{1}^{{}^{\circ}}$
7550.831	1		13239.930	$17411_{3}^{{}^{\circ}}$ – 30651_3_
7550.677	5		13240.200	13847_2_ – $27087_{1}^{{}^{\circ}}$
7549.923	2		13241.522	19713_3_ – $32954_{2}^{{}^{\circ}}$
7549.532	4		13242.208	$22163_{4}^{{}^{\circ}}$ – 35405_3_
7549.439	1		13242.371	20054_2_ – $33297_{2}^{{}^{\circ}}$
7549.316	200	8	13242.587	7280_2_ – $20522_{2}^{{}^{\circ}}$
7547.742	3		13245.348	$20922_{2}^{{}^{\circ}}$ – 34167_3_
7545.657	5		13249.008	12847_3_ – $26096_{3}^{{}^{\circ}}$
7544.592	2		13250.879	$20522_{2}^{{}^{\circ}}$ – 33773_1_
7540.596	15		13257.901	$18069_{3}^{{}^{\circ}}$ – 31326_4_
7539.622	4		13259.613	$18011_{0}^{{}^{\circ}}$ – 31271_5_
7537.429	50	2	13263.471	$15618_{3}^{{}^{\circ}}$ – 28882_2_
7536.407	50	10	13265.270	$20566_{4}^{{}^{\circ}}$ – 33831_4_
7536.244	1		13265.557	$20423_{1}^{{}^{\circ}}$ – 33689_2_
7535.850	5		13266.250	$21165_{3}^{{}^{\circ}}$ – 34431_3_
7531.818	50	2	13273.352	$18053_{4}^{{}^{\circ}}$ – 31326_4_
7531.142	75	2	13274.543	13088_3_ – $26363_{2}^{{}^{\circ}}$
7529.408	2		13277.600	22401_1_ – $35678_{1}^{{}^{\circ}}$
7529.318	3		13277.759	$22163_{4}^{{}^{\circ}}$ – 35440_3_
7528.919	5		13278.463	$19503_{3}^{{}^{\circ}}$ – 32781_4_
7528.490	40	8	13279.220	11601_1_ – $24880_{1}^{{}^{\circ}}$
7526.832	2		13282.145	$23015_{0}^{{}^{\circ}}$ – 36297_4_
7523.335	8		13288.318	16351_0_ – $29640_{1}^{{}^{\circ}}$
7523.133	100	3	13288.675	$14206_{4}^{{}^{\circ}}$ – 27495_4_
7520.694	15		13292.985	$19503_{3}^{{}^{\circ}}$ – 32796_3_
7520.567	2		13293.209	15863_2_ – $29157_{1}^{{}^{\circ}}$
7520.127	8		13293.987	15863_2_ – $29157_{3}^{{}^{\circ}}$
7519.279	1		13295.486	18574_1_ – $31870_{2}^{{}^{\circ}}$
7518.780	50	1	13296.369	13088_3_ – $26384_{4}^{{}^{\circ}}$
7517.963	50	2	13297.814	$20423_{1}^{{}^{\circ}}$ – 33721_2_
7515.504	1		13302.164	19273_2_ – $32575_{2}^{{}^{\circ}}$
7514.681	2		13303.621	21575_2_ – $34878_{1}^{{}^{\circ}}$
7512.237	3		13307.949	$21738_{2}^{{}^{\circ}}$ – 35046_1_
7511.788	100	4	13308.745	9804_5_ – $23113_{4}^{{}^{\circ}}$
7510.144	1		13311.658	$14032_{2}^{{}^{\circ}}$ – 27343_3_
7509.595	2		13312.631	$20214_{3}^{{}^{\circ}}$ – 33527_4_
7508.477	100	4	13314.614	$14206_{4}^{{}^{\circ}}$ – 27521_4_
7507.923	75		13315.596	$18011_{0}^{{}^{\circ}}$ – 31326_4_
7507.737	50		13315.926	$15618_{3}^{{}^{\circ}}$ – 28934_3_
7505.292	50		13320.264	$17224_{2}^{{}^{\circ}}$ – 30544_2_
7505.052	1		13320.690	18549_2_ – $31870_{2}^{{}^{\circ}}$
7503.829	50	8	13322.861	$14243_{1}^{{}^{\circ}}$ – 27566_2_
7503.626	2		13323.221	17959_4_ – $31283_{3}^{{}^{\circ}}$
7501.180	8		13327.566	$22248_{2}^{{}^{\circ}}$ – 35576_3_
7499.786	1		13330.043	19532_4_ – 32862d_4_
7499.579	8	1	13330.411	$21077_{0}^{{}^{\circ}}$ – 34407_6_
7499.000	75	2	13331.440	$8243_{2}^{{}^{\circ}}$ – 21575_2_
7497.552	10	1	13334.015	15863_2_ – $29197_{2}^{{}^{\circ}}$
7497.305	3		13334.454	$21539_{4}^{{}^{\circ}}$ – 34874_3_
7496.988	15	1	13335.018	13962_1_ – $27297_{1}^{{}^{\circ}}$
7495.564	50	2	13337.551	$16346_{4}^{{}^{\circ}}$ – 29684_5_
7494.237	2		13339.913	15970_3_ – $29310_{4}^{{}^{\circ}}$
7493.427	75	2	13341.355	8800_4_ – $22141_{3}^{{}^{\circ}}$
7490.379	5		13346.784	$17411_{3}^{{}^{\circ}}$ – 30758_2_
7489.709	50	2	13347.978	13297_4_ – $26645_{0}^{{}^{\circ}}$
7489.612	50	2	13348.151	$7795_{4}^{{}^{\circ}}$ – 21143_5_
7489.361	1		13348.598	21143_5_ – $34492_{4}^{{}^{\circ}}$
7487.972	100	3	13351.074	$8243_{2}^{{}^{\circ}}$ – 21594_3_
7487.869	3		13351.258	17166_5_ – $30517_{4}^{{}^{\circ}}$
7485.500	5		13355.483	$15490_{0}^{{}^{\circ}}$ – 28845_4_
7483.624	200	10	13358.831	3865_1_ – $17224_{2}^{{}^{\circ}}$
7481.352	200	15	13362.888	8800_4_ – $22163_{4}^{{}^{\circ}}$
7481.110	50	1	13363.320	17398_3_ – $30761_{3}^{{}^{\circ}}$
7480.802	2		13363.870	22098_4_ – $35462_{3}^{{}^{\circ}}$
7477.364	8		13370.015	$21077_{0}^{{}^{\circ}}$ – 34447_4_
7474.559	10	1	13375.032	$19588_{0}^{{}^{\circ}}$ – 32963_5_
7474.043	2		13375.956	$20922_{2}^{{}^{\circ}}$ – 34298_1_
7473.440	20	1	13377.035	$21668_{1}^{{}^{\circ}}$ – 35046_1_
7471.346	5		13380.784	18699_2_ – $32080_{1}^{{}^{\circ}}$
7469.705	4		13383.724	$21077_{0}^{{}^{\circ}}$ – 34460_5_
7469.054	40	1	13384.890	$14206_{4}^{{}^{\circ}}$ – 27591_5_
7466.819	5		13388.897	15863_2_ – $29252_{2}^{{}^{\circ}}$
7465.443	100	3	13391.364	$20214_{3}^{{}^{\circ}}$ – 33606_2_
7465.236	50	1	13391.736	15493_4_ – $28884_{3}^{{}^{\circ}}$
7465.025	15	1	13392.114	$18809_{4}^{{}^{\circ}}$ – 32202_5_
7463.427	1		13394.981	$22399_{0}^{{}^{\circ}}$ – 35794_4_
7462.990	75	1	13395.766	$15166_{3}^{{}^{\circ}}$ – 28562_4_
7461.502	5		13398.437	$13945_{3}^{{}^{\circ}}$ – 27343_3_
7461.385	3		13398.647	$21902_{4}^{{}^{\circ}}$ – 35300_5_
7461.290	8	1	13398.818	$17847_{2}^{{}^{\circ}}$ – 31245_2_
7460.310	10	1	13400.578	$22399_{0}^{{}^{\circ}}$ – 35799_6_
7458.753	50	2	13403.375	$17354_{1}^{{}^{\circ}}$ – 30758_2_
7457.648	25	1	13405.361	$14206_{4}^{{}^{\circ}}$ – 27612_3_
7455.204	100	4	13409.756	$16346_{4}^{{}^{\circ}}$ – 29756_4_
7453.730	20	1	13412.408	13847_2_ – $27260_{3}^{{}^{\circ}}$
7453.188	5		13413.383	$22163_{4}^{{}^{\circ}}$ – 35576_3_
7452.516	20	1	13414.593	17398_3_ – $30812_{2}^{{}^{\circ}}$
7452.432	2		13414.744	21575_2_ – $34989_{1}^{{}^{\circ}}$
7450.306	8		13418.572	$17847_{2}^{{}^{\circ}}$ – 31265_3_
7449.979	3		13419.161	$22163_{4}^{{}^{\circ}}$ – 35582_5_
7449.808	75	2	13419.469	13088_3_ – $26508_{3}^{{}^{\circ}}$
7449.605	75	3	13419.834	7502_3_ – $20922_{2}^{{}^{\circ}}$
7447.848	150	5	13423.000	$11241_{3}^{{}^{\circ}}$ – 24664_3_
7445.697	3		13426.878	$17224_{2}^{{}^{\circ}}$ – 30651_3_
7445.697	3		13426.878	$18614_{1}^{{}^{\circ}}$ – 32041_2_
7444.751	200	8	13428.584	8111_4_ – $21539_{4}^{{}^{\circ}}$
7444.373	8	1	13429.266	$17501_{0}^{{}^{\circ}}$ – 30930_6_
7443.873	50	1	13430.168	$11241_{3}^{{}^{\circ}}$ – 24671_2_
7443.573	1		13430.709	$23655_{4}^{{}^{\circ}}$ – 37085_4_
7442.040	15		13433.476	$16217_{2}^{{}^{\circ}}$ – 29650_2_
7441.125	25	1	13435.128	21645_4_ – $35081_{0}^{{}^{\circ}}$
7439.314	3		13438.398	18431_3_ – $31870_{2}^{{}^{\circ}}$
7437.331	1		13441.981	$22855_{3}^{{}^{\circ}}$ – 36297_4_
7433.375	8		13449.135	15970_3_ – $29419_{2}^{{}^{\circ}}$
7433.005	5		13449.804	13847_2_ – $27297_{1}^{{}^{\circ}}$
7430.255	500	15	13454.782	6362_2_ – $19817_{1}^{{}^{\circ}}$
7429.212	3		13456.671	$19817_{1}^{{}^{\circ}}$ – 33273_1_
7428.943	800	50	13457.159	7280_2_ – $20737_{1}^{{}^{\circ}}$
7427.224	2		13460.273	$19948_{4}^{{}^{\circ}}$ – 33408_3_
7426.906	4		13460.849	18699_2_ – $32160_{2}^{{}^{\circ}}$
7422.013	75	2	13469.724	$19948_{4}^{{}^{\circ}}$ – 33418_4_
7421.465	5		13470.718	$22163_{4}^{{}^{\circ}}$ – 35633_4_
7419.593	10		13474.117	$20214_{3}^{{}^{\circ}}$ – 33689_2_
7418.548	200		13476.015	5563_1_ – $19039_{2}^{{}^{\circ}}$
7417.789	100		13477.394	2869_3_ – $16346_{4}^{{}^{\circ}}$
7416.503	3		13479.731	$19588_{0}^{{}^{\circ}}$ – 33068_6_
7415.923	10		13480.785	$21668_{1}^{{}^{\circ}}$ – 35149_2_
7415.817	5		13480.978	$22396_{1}^{{}^{\circ}}$ – 35877_2_
7415.679	3		13481.228	19273_2_ – $32754_{3}^{{}^{\circ}}$
7414.361	10		13483.625	$18053_{4}^{{}^{\circ}}$ – 31537_3_
7414.139	3		13484.029	$18809_{4}^{{}^{\circ}}$ – 32293_5_
7413.592	25		13485.024	$15618_{3}^{{}^{\circ}}$ – 29104_4_
7413.484	3		13485.220	13962_1_ – $27447_{2}^{{}^{\circ}}$
7413.410	2		13485.355	21645_4_ – $35131_{4}^{{}^{\circ}}$
7411.739	100	4	13488.395	$16346_{4}^{{}^{\circ}}$ – 29835_3_
7411.322	10		13489.154	3865_1_ – $17354_{1}^{{}^{\circ}}$
7409.836	3		13491.859	$19817_{1}^{{}^{\circ}}$ – 33309_2_
7409.618	1		13492.256	$22141_{3}^{{}^{\circ}}$ – 35633_4_
7409.618	1		13492.256	$22338_{3}^{{}^{\circ}}$ – 35831_3_
7409.221	40		13492.979	13297_4_ – $26790_{4}^{{}^{\circ}}$
7404.262	50		13502.016	9804_5_ – $23306_{6}^{{}^{\circ}}$
7403.993	50	1	13502.506	$16346_{4}^{{}^{\circ}}$ – 29849_4_
7403.698	2		13503.044	$21902_{4}^{{}^{\circ}}$ – 35405_3_
7403.273	3		13503.819	11802_2_ – $25306_{2}^{{}^{\circ}}$
7402.248	400	8	13505.689	$10526_{3}^{{}^{\circ}}$ – 24032_4_
7401.872	50		13506.375	$20214_{3}^{{}^{\circ}}$ – 33721_2_
7400.309	2		13509.228	$20922_{2}^{{}^{\circ}}$ – 34431_3_
7400.071	40	2	13509.663	$19227_{6}^{{}^{\circ}}$ – 32737_5_
7399.391	8		13510.904	19532_4_ – $33043_{3}^{{}^{\circ}}$
7398.583	50	1	13512.380	$15166_{3}^{{}^{\circ}}$ – 28679_2_
7398.540	8		13512.458	$19039_{2}^{{}^{\circ}}$ – 32551_3_
7397.074	4		13515.136	12847_3_ – $26363_{2}^{{}^{\circ}}$
7396.895	20	1	13515.463	$14465_{2}^{{}^{\circ}}$ – 27980_3_
7394.970	1		13518.981	11802_2_ – $25321_{3}^{{}^{\circ}}$
7390.097	50		13527.896	$18930_{3}^{{}^{\circ}}$ – 32458_4_
7388.403	5		13530.997	18549_2_ – $32080_{1}^{{}^{\circ}}$
7386.924	50	2	13533.706	$17224_{2}^{{}^{\circ}}$ – 30758_2_
7386.347	50	10	13534.764	$14032_{2}^{{}^{\circ}}$ – 27566_2_
7386.055	8		13535.299	$17354_{1}^{{}^{\circ}}$ – 30889_1_
7385.498	500	15	13536.320	3687_2_ – $17224_{2}^{{}^{\circ}}$
7385.391	8b		13536.516	21594_3_ – $35131_{4}^{{}^{\circ}}$
7385.154	20		13536.950	12847_3_ – $26384_{4}^{{}^{\circ}}$
7384.174	200	5	13538.747	8800_4_ – $22338_{3}^{{}^{\circ}}$
7383.712	150	8	13539.594	$20867_{7}^{{}^{\circ}}$ – 34407_6_
7383.596	50	2	13539.806	17073_1_ – $30613_{0}^{{}^{\circ}}$
7378.165	2		13549.773	$21539_{4}^{{}^{\circ}}$ – 35089_3_
7377.891	40	1	13550.276	$13945_{3}^{{}^{\circ}}$ – 27495_4_
7377.626	2		13550.763	$21890_{3}^{{}^{\circ}}$ – 35440_3_
7376.879	200	8	13552.135	$14481_{6}^{{}^{\circ}}$ – 28034_5_
7376.178	25		13553.423	$17411_{3}^{{}^{\circ}}$ – 30964_3_
7375.142	1		13555.327	15863_2_ – $29419_{2}^{{}^{\circ}}$
7374.483	5		13556.538	$23655_{4}^{{}^{\circ}}$ – 37211_5_
7373.960	1		13557.500	15493_4_ – $29050_{0}^{{}^{\circ}}$
7373.960	1		13557.500	19713_3_ – $33270_{4}^{{}^{\circ}}$
7370.827	50	2	13563.262	13088_3_ – $26651_{2}^{{}^{\circ}}$
7370.409	4		13564.031	17959_4_ – $31523_{3}^{{}^{\circ}}$
7365.427	50	20	13573.206	$18053_{4}^{{}^{\circ}}$ – 31626_5_
7365.233	50	20	13573.564	$19817_{1}^{{}^{\circ}}$ – 33390_1_
7365.166	3h		13573.687	$20724_{0}^{{}^{\circ}}$ – 34298_1_
7363.794	10	1	13576.216	$13945_{3}^{{}^{\circ}}$ – 27521_4_
7362.195	50	1	13579.165	$19948_{4}^{{}^{\circ}}$ – 33527_4_
7361.348	150	5	13580.727	13297_4_ – $26878_{3}^{{}^{\circ}}$
7360.715	2		13581.895	19713_3_ – $33294_{3}^{{}^{\circ}}$
7360.553	2		13582.194	$17847_{2}^{{}^{\circ}}$ – 31429_1_
7360.428	3		13582.425	$19516_{2}^{{}^{\circ}}$ – 33099_3_
7359.530	2		13584.082	19713_3_ – $33297_{2}^{{}^{\circ}}$
7358.738	2		13585.544	18699_2_ – $32285_{3}^{{}^{\circ}}$
7358.568	8		13585.858	18574_1_ – $32160_{2}^{{}^{\circ}}$
7355.179	10		13592.118	17398_3_ – $30990_{3}^{{}^{\circ}}$
7353.644	15		13594.955	21645_4_ – $35240_{3}^{{}^{\circ}}$
7352.937	8		13596.262	$19503_{3}^{{}^{\circ}}$ – 33099_3_
7351.371	1		13599.158	8800_4_ – $22399_{0}^{{}^{\circ}}$
7350.934	5		13599.967	13847_2_ – $27447_{2}^{{}^{\circ}}$
7350.454	50		13600.855	$20566_{4}^{{}^{\circ}}$ – 34167_3_
7346.898	2		13607.438	20054_2_ – $33662_{1}^{{}^{\circ}}$
7346.671	2		13607.858	$22877_{1}^{{}^{\circ}}$ – 36485_2_
7344.939	10		13611.067	18549_2_ – $32160_{2}^{{}^{\circ}}$
7343.396	5h		13613.927	$15490_{0}^{{}^{\circ}}$ – 29104_4_
7342.573	150	5	13615.453	$18011_{0}^{{}^{\circ}}$ – 31626_5_
7342.300	75	2	13615.959	$10783_{2}^{{}^{\circ}}$ – 24399_3_
7342.035	75	2	13616.451	$19948_{4}^{{}^{\circ}}$ – 33564_3_
7341.769	5		13616.944	$20214_{3}^{{}^{\circ}}$ – 33831_4_
7341.433	8		13617.567	$16217_{2}^{{}^{\circ}}$ – 29835_3_
7341.150	500	15	13618.092	$10414_{4}^{{}^{\circ}}$ – 24032_4_
7339.729	3		13620.729	17398_3_ – $31019_{4}^{{}^{\circ}}$
7339.599	150	3	13620.970	$13175_{4}^{{}^{\circ}}$ – 26796_3_
7339.409	50	1	13621.322	$18930_{3}^{{}^{\circ}}$ – 32551_3_
7339.330	2		13621.469	$21252_{2}^{{}^{\circ}}$ – 34874_3_
7339.005	8		13622.072	17166_5_ – $30788_{0}^{{}^{\circ}}$
7336.798	4		13626.170	$19588_{0}^{{}^{\circ}}$ – 33214_5_
7336.134	2		13627.403	$22669_{3}^{{}^{\circ}}$ – 36297_4_
7335.802	5		13628.020	21645_4_ – $35273_{0}^{{}^{\circ}}$
7335.357	1		13628.847	$22248_{2}^{{}^{\circ}}$ – 35877_2_
7334.067	4		13631.244	$22163_{4}^{{}^{\circ}}$ – 35794_4_
7331.906	2		13635.262	22098_4_ – $35733_{4}^{{}^{\circ}}$
7329.488	100	5	13639.760	11802_2_ – $25442_{3}^{{}^{\circ}}$
7328.283	300s	50d	13642.003	7280_2_ – $20922_{2}^{{}^{\circ}}$
7326.801	5		13644.762	$20522_{2}^{{}^{\circ}}$ – 34167_3_
7326.058	3		13646.146	21594_3_ – $35240_{3}^{{}^{\circ}}$
7325.219	8		13647.709	18549_2_ – $32197_{3}^{{}^{\circ}}$
7324.894	20b		13648.314	$23655_{4}^{{}^{\circ}}$ – 37303_4_
7324.808	300	10	13648.475	14204_5_ – $27852_{0}^{{}^{\circ}}$
7323.988	10		13650.003	17073_1_ – $30723_{1}^{{}^{\circ}}$
7323.740	2		13650.465	$22396_{1}^{{}^{\circ}}$ – 36047_2_
7323.207	100	3	13651.458	$15490_{0}^{{}^{\circ}}$ – 29141_5_
7318.595	8		13660.061	12847_3_ – $26508_{3}^{{}^{\circ}}$
7318.272	2h		13660.664	$22855_{3}^{{}^{\circ}}$ – 36515_3_
7317.122	3		13662.811	7502_3_ – $21165_{3}^{{}^{\circ}}$
7316.676	2		13663.644	20054_2_ – $33718_{2}^{{}^{\circ}}$
7316.124	100	1	13664.675	15493_4_ – $29157_{3}^{{}^{\circ}}$
7315.609	150		13665.637	$17224_{2}^{{}^{\circ}}$ – 30889_1_
7315.064	200	3	13666.655	3687_2_ – $17354_{1}^{{}^{\circ}}$
7314.743	2		13667.255	$21738_{2}^{{}^{\circ}}$ – 35405_3_
7311.510	100	3	13673.298	$11241_{3}^{{}^{\circ}}$ – 24915_3_
7311.003	10	2	13674.246	$21902_{4}^{{}^{\circ}}$ – 35576_3_
7309.311	2b		13677.412	$21539_{4}^{{}^{\circ}}$ – 35216_5_
7309.255	100	2	13677.516	17398_3_ – $31075_{2}^{{}^{\circ}}$
7308.640	150	3	13678.667	$15166_{3}^{{}^{\circ}}$ – 28845_4_
7307.933	4		13679.991	$21902_{4}^{{}^{\circ}}$ – 35582_5_
7307.233	4		13681.301	19273_2_ – $32954_{2}^{{}^{\circ}}$
7307.176	5		13681.408	$15736_{1}^{{}^{\circ}}$ – 29418_2_
7305.193	50	1	13685.122	$15736_{1}^{{}^{\circ}}$ – 29422_1_
7304.521	4		13686.381	$21890_{3}^{{}^{\circ}}$ – 35576_3_
7302.496	3b		13690.176	$17847_{2}^{{}^{\circ}}$ – 31537_3_
7302.428	75	2	13690.303	$22399_{0}^{{}^{\circ}}$ – 36089_4_
7298.138	200b	3	13698.351	13297_4_ – $26995_{3}^{{}^{\circ}}$
7296.265	100	3	13701.867	13088_3_ – $26790_{4}^{{}^{\circ}}$
7294.316	15	1	13705.528	$18069_{3}^{{}^{\circ}}$ – 31774_3_
7294.217	5		13705.714	11601_1_ – $25306_{2}^{{}^{\circ}}$
7293.374	15	1	13707.298	$20737_{1}^{{}^{\circ}}$ – 34444_2_
7292.864	2		13708.257	$21165_{3}^{{}^{\circ}}$ – 34873_4_
7292.864	2		13708.257	$22338_{3}^{{}^{\circ}}$ – 36047_2_
7292.494	3		13708.952	$21165_{3}^{{}^{\circ}}$ – 34874_3_
7291.276	8	2	13711.242	17959_4_ – $31671_{4}^{{}^{\circ}}$
7289.812	4		13713.996	19713_3_ – $33427_{2}^{{}^{\circ}}$
7289.659	2		13714.284	22098_4_ – $35812_{3}^{{}^{\circ}}$
7288.990	10	1	13715.543	$15166_{3}^{{}^{\circ}}$ – 28882_2_
7288.610	50	1	13716.258	15970_3_ – $29686_{3}^{{}^{\circ}}$
7286.110	3		13720.964	$18053_{4}^{{}^{\circ}}$ – 31774_3_
7284.905	500	20	13723.233	3687_2_ – $17411_{3}^{{}^{\circ}}$
7284.419	40	1	13724.149	$16783_{4}^{{}^{\circ}}$ – 30508_5_
7284.166	8	1	13724.626	$18069_{3}^{{}^{\circ}}$ – 31793_4_
7281.962	2		13728.780	18431_3_ – $32160_{2}^{{}^{\circ}}$
7280.478	20	1	13731.578	$21902_{4}^{{}^{\circ}}$ – 35633_4_
7279.142	8		13734.098	$22399_{0}^{{}^{\circ}}$ – 36133_5_
7278.767	1		13734.806	$20566_{4}^{{}^{\circ}}$ – 34301_4_
7278.272	1		13735.740	18549_2_ – $32285_{3}^{{}^{\circ}}$
7278.101	3		13736.063	$19227_{6}^{{}^{\circ}}$ – 32963_5_
7278.032	4		13736.193	$22141_{3}^{{}^{\circ}}$ – 35877_2_
7277.013	75	1	13738.116	19532_4_ – $33270_{4}^{{}^{\circ}}$
7276.453	10	1	13739.174	17073_1_ – $30812_{2}^{{}^{\circ}}$
7275.972	2		13740.082	$18053_{4}^{{}^{\circ}}$ – 31793_4_
7275.833	15	1	13740.344	$17224_{2}^{{}^{\circ}}$ – 30964_3_
7274.294	15	1	13743.251	21143_5_ – $34886_{0}^{{}^{\circ}}$
7274.048	3		13743.716	$21890_{3}^{{}^{\circ}}$ – 35633_4_
7273.732	40	1	13744.313	$16217_{2}^{{}^{\circ}}$ – 29961_1_
7270.993	8		13749.491	$23655_{4}^{{}^{\circ}}$ – 37404_3_
7270.558	100	2	13750.313	7502_3_ – $21252_{2}^{{}^{\circ}}$
7268.834	100	2	13753.575	21575_2_ – $35328_{1}^{{}^{\circ}}$
7268.490	5	1	13754.226	$20543_{0}^{{}^{\circ}}$ – 34298_1_
7267.096	50	1	13756.864	$19516_{2}^{{}^{\circ}}$ – 33273_1_
7264.747	2		13761.312	$21539_{4}^{{}^{\circ}}$ – 35300_5_
7264.118	50		13762.504	19532_4_ – $33294_{3}^{{}^{\circ}}$
7262.578	2		13765.422	18431_3_ – $32197_{3}^{{}^{\circ}}$
7262.190	10		13766.157	$23015_{0}^{{}^{\circ}}$ – 36781_5_
7261.217	5		13768.002	$15166_{3}^{{}^{\circ}}$ – 28934_3_
7260.874	5		13768.652	$16783_{4}^{{}^{\circ}}$ – 30552_4_
7260.258	8		13769.821	$17501_{0}^{{}^{\circ}}$ – 31271_5_
7258.177	200	5	13773.769	$14206_{4}^{{}^{\circ}}$ – 27980_3_
7257.334	2		13775.369	$20522_{2}^{{}^{\circ}}$ – 34298_1_
7256.981	150	2	13776.039	$12114_{2}^{{}^{\circ}}$ – 25890_2_
7256.807	75	2	13776.369	15863_2_ – $29640_{1}^{{}^{\circ}}$
7255.351	200	5	13779.133	8111_4_ – $21890_{3}^{{}^{\circ}}$
7253.672	100	3	13782.323	$18011_{0}^{{}^{\circ}}$ – 31793_4_
7251.943	2		13785.609	$20922_{2}^{{}^{\circ}}$ – 34707_3_
7250.100	15		13789.113	$19817_{1}^{{}^{\circ}}$ – 33606_2_
7249.842	75	1	13789.604	13088_3_ – $26878_{3}^{{}^{\circ}}$
7248.964	75	1	13791.274	8111_4_ – $21902_{4}^{{}^{\circ}}$
7248.547	2		13792.067	$19516_{2}^{{}^{\circ}}$ – 33309_2_
7247.843	2		13793.407	$21252_{2}^{{}^{\circ}}$ – 35046_1_
7247.599	40		13793.872	$16217_{2}^{{}^{\circ}}$ – 30011_3_
7246.370	3		13796.211	$21077_{0}^{{}^{\circ}}$ – 34873_4_
7246.318	5h		13796.310	17398_3_ – $31194_{4}^{{}^{\circ}}$
7245.500	8		13797.868	14226_0_ – $28024_{1}^{{}^{\circ}}$
7245.292	2		13798.264	$22855_{3}^{{}^{\circ}}$ – 36653_2_
7244.689	200	8	13799.412	$7795_{4}^{{}^{\circ}}$ – 21594_3_
7244.689	200	8	13799.412	$15618_{3}^{{}^{\circ}}$ – 29418_2_
7242.345	150	2	13803.878	12847_3_ – $26651_{2}^{{}^{\circ}}$
7242.282	20b		13803.998	18699_2_ – $32503_{2}^{{}^{\circ}}$
7242.086	200	10	13804.372	9804_5_ – $23609_{0}^{{}^{\circ}}$
7241.280	2		13805.908	$19503_{3}^{{}^{\circ}}$ – 33309_2_
7240.181	100	2	13808.004	$14465_{2}^{{}^{\circ}}$ – 28273_2_
7238.404	8		13811.394	$22399_{0}^{{}^{\circ}}$ – 36210_5_
7236.257	20		13815.492	$22669_{3}^{{}^{\circ}}$ – 36485_2_
7235.899	50	1	13816.175	21645_4_ – $35462_{3}^{{}^{\circ}}$
7235.551	20		13816.840	15493_4_ – $29310_{4}^{{}^{\circ}}$
7233.390	100		13820.967	17959_4_ – $31780_{3}^{{}^{\circ}}$
7232.946	10		13821.816	13962_1_ – $27784_{2}^{{}^{\circ}}$
7226.246	15		13834.631	$17411_{3}^{{}^{\circ}}$ – 31245_2_
7225.125	20	5	13836.778	$21252_{2}^{{}^{\circ}}$ – 35089_3_
7220.989	100	2	13844.703	$10783_{2}^{{}^{\circ}}$ – 24627_1_
7219.782	25	1	13847.017	19713_3_ – $33560_{4}^{{}^{\circ}}$
7219.156	300	10	13848.218	4961_4_ – $18809_{4}^{{}^{\circ}}$
7218.056	500	75	13850.328	9804_5_ – $23655_{4}^{{}^{\circ}}$
7216.911	8		13852.526	6362_2_ – $20214_{3}^{{}^{\circ}}$
7216.666	40	2	13852.996	17166_5_ – $31019_{4}^{{}^{\circ}}$
7215.933	8		13854.403	$17411_{3}^{{}^{\circ}}$ – 31265_3_
7215.667	10		13854.914	17073_1_ – $30928_{1}^{{}^{\circ}}$
7212.687	500	50	13860.638	$14481_{6}^{{}^{\circ}}$ – 28342_5_
7211.769	2		13862.403	18431_3_ – $32294_{4}^{{}^{\circ}}$
7210.562	2		13864.723	$20566_{4}^{{}^{\circ}}$ – 34431_3_
7210.036	5		13865.735	$21539_{4}^{{}^{\circ}}$ – 35405_3_
7209.981	5		13865.840	$18930_{3}^{{}^{\circ}}$ – 32796_3_
7209.177	5		13867.387	21594_3_ – $35462_{3}^{{}^{\circ}}$
7208.275	100b		13869.122	$13175_{4}^{{}^{\circ}}$ – 27044_3_
7208.000	500	150	13869.651	8800_4_ – $22669_{3}^{{}^{\circ}}$
7207.756	50b	2	13870.121	$23015_{0}^{{}^{\circ}}$ – 36885_4_
7207.392	5		13870.821	$19588_{0}^{{}^{\circ}}$ – 33459_4_
7207.016	50	2	13871.545	$17224_{2}^{{}^{\circ}}$ – 31095_1_
7206.476	400	8	13872.584	$10526_{3}^{{}^{\circ}}$ – 24399_3_
7205.435	2		13874.589	$20423_{1}^{{}^{\circ}}$ – 34298_1_
7204.761	3h		13875.887	$18053_{4}^{{}^{\circ}}$ – 31929_3_
7203.593	4		13878.136	19713_3_ – $33591_{3}^{{}^{\circ}}$
7202.335	5		13880.560	$20566_{4}^{{}^{\circ}}$ – 34447_4_
7201.803	100	2	13881.586	$10783_{2}^{{}^{\circ}}$ – 24664_3_
7201.042	50	1	13883.053	15970_3_ – $29853_{2}^{{}^{\circ}}$
7200.815	2		13883.490	$19948_{4}^{{}^{\circ}}$ – 33831_4_
7200.171	50		13884.732	17398_3_ – $31283_{3}^{{}^{\circ}}$
7200.041	400	10	13884.983	7280_2_ – $21165_{3}^{{}^{\circ}}$
7198.986	5		13887.018	21575_2_ – $35462_{3}^{{}^{\circ}}$
7198.214	4		13888.507	19273_2_ – $33161_{1}^{{}^{\circ}}$
7198.092	20		13888.742	$10783_{2}^{{}^{\circ}}$ – 24671_2_
7196.791	2		13891.253	$17354_{1}^{{}^{\circ}}$ – 31245_2_
7196.569	8		13891.682	$19516_{2}^{{}^{\circ}}$ – 33408_3_
7195.797	4		13893.172	$14465_{2}^{{}^{\circ}}$ – 28358_3_
7195.236	3		13894.255	$20566_{4}^{{}^{\circ}}$ – 34460_5_
7193.357	1		13897.885	$22399_{0}^{{}^{\circ}}$ – 36297_4_
7192.019	40	2	13900.470	11802_2_ – $25703_{2}^{{}^{\circ}}$
7191.585	3		13901.309	$21539_{4}^{{}^{\circ}}$ – 35440_3_
7190.332	2		13903.731	$22877_{1}^{{}^{\circ}}$ – 36781_5_
7190.124	5		13904.134	$19817_{1}^{{}^{\circ}}$ – 33721_2_
7190.050	5		13904.277	$21890_{3}^{{}^{\circ}}$ – 35794_4_
7189.407	10	1	13905.520	$19503_{3}^{{}^{\circ}}$ – 33408_3_
7189.323	1		13905.683	$22141_{3}^{{}^{\circ}}$ – 36047_2_
7188.530	100	3	13907.217	13088_3_ – $26995_{3}^{{}^{\circ}}$
7185.031	3		13913.989	$15736_{1}^{{}^{\circ}}$ – 29650_2_
7184.721	4		13914.590	2869_3_ – $16783_{4}^{{}^{\circ}}$
7184.542	3		13914.936	$19503_{3}^{{}^{\circ}}$ – 33418_4_
7184.426	4		13915.161	$16217_{2}^{{}^{\circ}}$ – 30132_2_
7184.111	8		13915.771	$17411_{3}^{{}^{\circ}}$ – 31326_4_
7183.225	2		13917.488	$17224_{2}^{{}^{\circ}}$ – 31141_3_
7180.959	8		13921.879	$20522_{2}^{{}^{\circ}}$ – 34444_2_
7179.725	150	4	13924.272	$21165_{3}^{{}^{\circ}}$ – 35089_3_
7179.189	1		13925.312	11601_1_ – $25526_{1}^{{}^{\circ}}$
7178.539	10	1	13926.572	$18069_{3}^{{}^{\circ}}$ – 31995_4_
7178.252	8		13927.129	$18809_{4}^{{}^{\circ}}$ – 32737_5_
7177.284	50	2	13929.008	18574_1_ – $32503_{2}^{{}^{\circ}}$
7173.372	500	20	13936.604	13847_2_ – $27784_{2}^{{}^{\circ}}$
7173.372	500	20	13936.604	14204_5_ – $28140_{4}^{{}^{\circ}}$
7173.117	8		13937.099	$15166_{3}^{{}^{\circ}}$ – 29104_4_
7172.867	8		13937.585	21143_5_ – $35081_{0}^{{}^{\circ}}$
7170.575	2		13942.040	$18053_{4}^{{}^{\circ}}$ – 31995_4_
7170.357	100	2	13942.464	12847_3_ – $26790_{4}^{{}^{\circ}}$
7169.621	4		13943.895	$18069_{3}^{{}^{\circ}}$ – 32012_4_
7168.896	800	75	13945.305	0_2_ – $13945_{3}^{{}^{\circ}}$
7167.202	150	4	13948.601	$14032_{2}^{{}^{\circ}}$ – 27980_3_
7165.492	8	1	13951.930	$20922_{2}^{{}^{\circ}}$ – 34874_3_
7165.176	25	1	13952.545	$20214_{3}^{{}^{\circ}}$ – 34167_3_
7164.508	2		13953.846	5563_1_ – $19516_{2}^{{}^{\circ}}$
7164.323	8		13954.206	18549_2_ – $32503_{2}^{{}^{\circ}}$
7163.374	2		13956.055	$19503_{3}^{{}^{\circ}}$ – 33459_4_
7163.180	5		13956.433	$19817_{1}^{{}^{\circ}}$ – 33773_1_
7162.250	8	1	13958.245	21575_2_ – $35533_{2}^{{}^{\circ}}$
7161.689	2		13959.339	$18053_{4}^{{}^{\circ}}$ – 32012_4_
7159:943	300b	8	13962.743	13297_4_ – $27260_{3}^{{}^{\circ}}$
7159.877	100b	2	13962.871	16554_6_ – $30517_{0}^{{}^{\circ}}$
7156.936	300	15	13968.609	4961_4_ – $18930_{3}^{{}^{\circ}}$
7156.936	300	15	13968.609	13297_4_ – $27266_{4}^{{}^{\circ}}$
7155.349	8		13971.707	$18809_{4}^{{}^{\circ}}$ – 32781_4_
7154.952	200	8	13972.482	7280_2_ – $21252_{2}^{{}^{\circ}}$
7154.762	200	20	13972.853	13088_3_ – $27061_{2}^{{}^{\circ}}$
7153.587	200	10	13975.149	17166_5_ – $31141_{0}^{{}^{\circ}}$
7151.525	20	1	13979.178	$20322_{0}^{{}^{\circ}}$ – 34301_4_
7150.285	400	25	13981.602	3865_1_ – $17847_{2}^{{}^{\circ}}$
7149.224	5		13983.677	$22669_{3}^{{}^{\circ}}$ – 36653_2_
7148.923	8	1	13984.266	$18011_{0}^{{}^{\circ}}$ – 31995_4_
7148.728	50	1	13984.647	$21165_{3}^{{}^{\circ}}$ – 35149_2_
7148.557	400	15	13984.982	$10414_{4}^{{}^{\circ}}$ – 24399_3_
7147.913	3		13986.242	$18809_{4}^{{}^{\circ}}$ – 32796_3_
7147.423	15	1	13987.201	$19227_{6}^{{}^{\circ}}$ – 33214_5_
7147.167	2		13987.702	$21890_{3}^{{}^{\circ}}$ – 35877_2_
7146.373	8	1	13989.256	15863_2_ – $29853_{2}^{{}^{\circ}}$
7144.169	8		13993.572	17959_4_ – $31953_{4}^{{}^{\circ}}$
7142.328	300	8	13997.179	$12114_{2}^{{}^{\circ}}$ – 26111_1_
7140.080	4		14001.585	$18011_{0}^{{}^{\circ}}$ – 32012_4_
7138.142	20		14005.387	19713_3_ – $33718_{2}^{{}^{\circ}}$
7137.635	3		14006.382	11802_2_ – $25809_{1}^{{}^{\circ}}$
7137.133	5		14007.367	18431_3_ – $32439_{4}^{{}^{\circ}}$
7136.956	8		14007.714	$22877_{1}^{{}^{\circ}}$ – 36885_4_
7136.841	4		14007.940	$22508_{2}^{{}^{\circ}}$ – 36515_3_
7134.484	8		14012.568	$11877_{1}^{{}^{\circ}}$ – 25890_2_
7132.603	200	4	14016.263	$13175_{4}^{{}^{\circ}}$ – 27191_5_
7130.718	200	5	14019.968	13297_4_ – $27317_{3}^{{}^{\circ}}$
7130.179	100	4	14021.028	$14206_{4}^{{}^{\circ}}$ – 28227_4_
7129.871	20		14021.634	19273_2_ – $33294_{3}^{{}^{\circ}}$
7128.450	8	1	14024.429	$19503_{3}^{{}^{\circ}}$ – 33527_4_
7127.640	10		14026.022	18549_2_ – $32575_{2}^{{}^{\circ}}$
7126.814	100	3	14027.648	19532_4_ – $33560_{4}^{{}^{\circ}}$
7126.324	8		14028.613	17166_5_ – $31194_{4}^{{}^{\circ}}$
7126.009	5		14029.233	$14243_{1}^{{}^{\circ}}$ – 28273_2_
7125.515	150	2	14030.205	12847_3_ – $26878_{3}^{{}^{\circ}}$
7125.313	10	1	14030.603	8111_4_ – $22141_{3}^{{}^{\circ}}$
7124.556	1000	50	14032.094	0_2_ — $14032_{2}^{{}^{\circ}}$
7122.887	3		14035.382	$13945_{3}^{{}^{\circ}}$ – 27980_3_
7122.098	3		14036.937	$21539_{4}^{{}^{\circ}}$ – 35576_3_
7121.908	8		14037.311	7502_3_ – $21539_{4}^{{}^{\circ}}$
7119.870	3		14041.329	$17224_{2}^{{}^{\circ}}$ – 31265_3_
7119.174	2		14042.702	$21539_{4}^{{}^{\circ}}$ – 35582_5_
7116.992	20	1	14047.007	15970_3_ – $30017_{3}^{{}^{\circ}}$
7116.551	8		14047.878	$19516_{2}^{{}^{\circ}}$ – 33564_3_
7114.394	150	3	14052.137	8111_4_ – $22163_{4}^{{}^{\circ}}$
7113.007	5h		14054.877	18699_2_ – $32754_{3}^{{}^{\circ}}$
7112.917	100	4	14055.055	8800_4_ – $22855_{3}^{{}^{\circ}}$
7111.045	75	2	14058.755	19532_4_ – $33591_{3}^{{}^{\circ}}$
7110.278	2		14060.271	$19039_{2}^{{}^{\circ}}$ – 33099_3_
7109.857	150	4	14061.104	6362_2_ – $20423_{1}^{{}^{\circ}}$
7109.550	40	2	14061.711	$19503_{3}^{{}^{\circ}}$ – 33564_3_
7109.320	10		14062.166	13962_1_ – $28024_{1}^{{}^{\circ}}$
7109.175	50		14062.453	$15490_{0}^{{}^{\circ}}$ – 29552_6_
7108.550	2		14063.689	17959_4_ – $32023_{0}^{{}^{\circ}}$
7107.469	10	1	14065.828	$14465_{2}^{{}^{\circ}}$ – 28531_2_
7105.654	10		14069.421	$16217_{2}^{{}^{\circ}}$ – 30286_1_
7104.404	41		14071.896	18431_3_ – $32503_{2}^{{}^{\circ}}$
7103.029	8		14074.620	$17354_{1}^{{}^{\circ}}$ – 31429_1_
7102.185	10		14076.293	18574_1_ – $32650_{1}^{{}^{\circ}}$
7101.660	4		14077.334	8800_4_ – $22877_{0}^{{}^{\circ}}$
7100.391	2		14079.850	22098_4_ – $36178_{4}^{{}^{\circ}}$
7099.094	4		14082.422	$17847_{2}^{{}^{\circ}}$ – 31929_3_
7097.645	5		14085.297	$20322_{0}^{{}^{\circ}}$ – 34407_6_
7097.032	10		14086.513	$20214_{3}^{{}^{\circ}}$ – 34301_4_
7096.665	5		14087.242	$22248_{2}^{{}^{\circ}}$ – 36336_3_
7096.478	4		14087.613	19713_3_ – $33800_{3}^{{}^{\circ}}$
7096.000	3		14088.562	$22396_{1}^{{}^{\circ}}$ – 36485_2_
7095.623	4		14089.311	$19516_{2}^{{}^{\circ}}$ – 33606_2_
7094.803	3		14090.939	18574_1_ – $32665_{1}^{{}^{\circ}}$
7093.128	3		14094.266	$21539_{4}^{{}^{\circ}}$ – 35633_4_
7089.489	8		14101.501	18549_2_ – $32650_{1}^{{}^{\circ}}$
7089.052	8		14102.370	11601_1_ – $25703_{2}^{{}^{\circ}}$
7088.663	1		14103.144	$19503_{3}^{{}^{\circ}}$ – 33606_2_
7088.330	8		14103.807	21575_2_ – $35678_{1}^{{}^{\circ}}$
7087.506	3		14105.446	$22669_{3}^{{}^{\circ}}$ – 36775_3_
7084.165	500	75	14112.099	9804_5_ – $23916_{4}^{{}^{\circ}}$
7082.137	20		14116.140	18549_2_ – $32665_{1}^{{}^{\circ}}$
7077.740	4		14124.909	$20322_{0}^{{}^{\circ}}$ – 34447_4_
7077.422	50b	3	14125.544	17398_3_ – $31523_{3}^{{}^{\circ}}$
7077.380	50b	4	14125.628	$17501_{0}^{{}^{\circ}}$ – 31626_5_
7077.194	4		14125.999	$17411_{3}^{{}^{\circ}}$ – 31537_3_
7074.257	50	1	14131.863	$10783_{2}^{{}^{\circ}}$ – 24915_3_
7073.110	8		14134.155	$22163_{4}^{{}^{\circ}}$ – 36297_4_
7072.394	300	8	14135.586	$14206_{4}^{{}^{\circ}}$ – 28342_5_
7071.482	100		14137.409	$15618_{3}^{{}^{\circ}}$ – 29756_4_
7071.096	150	3	14138.181	$10526_{3}^{{}^{\circ}}$ – 24664_3_
7070.984	10		14138.405	$22669_{3}^{{}^{\circ}}$ – 36808_3_
7070.891	2		14138.591	$20322_{0}^{{}^{\circ}}$ – 34460_5_
7070.793	1		14138.787	21594_3_ – $35733_{4}^{{}^{\circ}}$
7070.279	2		14139.815	$21077_{0}^{{}^{\circ}}$ – 35216_5_
7068.330	2		14143.713	18431_3_ – $32575_{2}^{{}^{\circ}}$
7067.513	75		14145.348	$10526_{3}^{{}^{\circ}}$ – 24671_2_
7067.247	10		14145.881	14226_0_ – $28372_{1}^{{}^{\circ}}$
7066.996	3		14146.383	$22338_{3}^{{}^{\circ}}$ – 36485_2_
7066.287	100	2	14147.803	12847_3_ – $26995_{3}^{{}^{\circ}}$
7064.451	300	10	14151.480	$14206_{4}^{{}^{\circ}}$ – 28358_3_
7063.844	8		14152.696	$21252_{2}^{{}^{\circ}}$ – 35405_3_
7063.585	10		14153.214	15863_2_ – $30017_{3}^{{}^{\circ}}$
7063.314	8		14153.757	19273_2_ – $33427_{2}^{{}^{\circ}}$
7062.418	40	1	14155.553	22401_1_ – $36556_{1}^{{}^{\circ}}$
7061.607	1		14157.179	$21890_{3}^{{}^{\circ}}$ – 36047_2_
7061.392	100	2	14157.610	$8243_{2}^{{}^{\circ}}$ – 22401_1_
7060.651	400	10	14159.096	3687_2_ – $17847_{2}^{{}^{\circ}}$
7060.039	300	8	14160.323	6362_2_ – $20522_{2}^{{}^{\circ}}$
7059.526	150	3	14161.352	$16346_{4}^{{}^{\circ}}$ – 30508_5_
7058.488	150	5	14163.435	$11241_{3}^{{}^{\circ}}$ – 25405_4_
7058.221	2		14163.970	$23655_{4}^{{}^{\circ}}$ – 37819_4_
7056.885	4		14166.652	21645_4_ – $35812_{3}^{{}^{\circ}}$
7056.595	10		14167.234	$20922_{2}^{{}^{\circ}}$ – 35089_3_
7055.900	50		14168.629	$13175_{4}^{{}^{\circ}}$ – 27343_3_
7055.660	1		14169.111	$18930_{3}^{{}^{\circ}}$ – 33099_3_
7054.416	150	2	14171.610	13088_3_ – $27260_{3}^{{}^{\circ}}$
7051.787	50		14176.893	13847_2_ – $28024_{1}^{{}^{\circ}}$
7051.503	8		14177.464	13088_3_ – $27266_{4}^{{}^{\circ}}$
7049.844	20		14180.801	$16783_{4}^{{}^{\circ}}$ – 30964_3_
7047.762	2		14184.990	$20522_{2}^{{}^{\circ}}$ – 34707_3_
7047.319	2		14185.882	$19503_{3}^{{}^{\circ}}$ – 33689_2_
7046.560	40		14187.409	$21902_{4}^{{}^{\circ}}$ – 36089_4_
7046.124	10		14188.287	$21252_{2}^{{}^{\circ}}$ – 35440_3_
7045.332	1		14189.882	15970_3_ – $30160_{4}^{{}^{\circ}}$
7044.969	50	1	14190.613	$18011_{0}^{{}^{\circ}}$ – 32202_5_
7043.714	15		14193.142	15493_4_ – $29686_{3}^{{}^{\circ}}$
7043.228	8		14194.121	$15490_{0}^{{}^{\circ}}$ – 29684_5_
7042.997	8		14194.587	$22141_{3}^{{}^{\circ}}$ – 36336_3_
7042.031	3		14196.534	$23015_{0}^{{}^{\circ}}$ – 37211_5_
7040.530	2		14199.560	$21890_{3}^{{}^{\circ}}$ – 36089_4_
7039.877	5		14200.878	17398_3_ – $31599_{2}^{{}^{\circ}}$
7038.165	40		14204.332	$19516_{2}^{{}^{\circ}}$ – 33721_2_
7037.852	10		14204.964	$17224_{2}^{{}^{\circ}}$ – 31429_1_
7037.781	8		14205.107	18549_2_ – $32754_{3}^{{}^{\circ}}$
7037.410	4		14205.856	$16346_{4}^{{}^{\circ}}$ – 30552_4_
7036.277	400	8	14208.143	$11197_{0}^{{}^{\circ}}$ – 25405_4_
7036.206	50b		14208.287	11601_1_ – $25809_{1}^{{}^{\circ}}$
7033.646	50	1	14213.458	12847_3_ – $27061_{2}^{{}^{\circ}}$
7033.352	100	2	14214.052	$14465_{2}^{{}^{\circ}}$ – 28679_2_
7032.924	100	1	14214.917	8800_4_ – $23015_{0}^{{}^{\circ}}$
7032.677	3		14215.416	$22669_{3}^{{}^{\circ}}$ – 36885_4_
7032.350	3		14216.077	$15618_{3}^{{}^{\circ}}$ – 29835_3_
7032.178	15	1	14216.425	$20214_{3}^{{}^{\circ}}$ – 34431_3_
7031.469	15	1	14217.858	21594_3_ – $35812_{3}^{{}^{\circ}}$
7031.310	50	1	14218.180	$19503_{3}^{{}^{\circ}}$ – 33721_2_
7031.194	10		14218.415	15493_4_ – $29711_{0}^{{}^{\circ}}$
7030.858	50	1	14219.094	$19948_{4}^{{}^{\circ}}$ – 34167_3_
7028.017	100	2	14224.842	$15736_{1}^{{}^{\circ}}$ – 29961_1_
7026.454	100	1	14228.006	8111_4_ – $22338_{3}^{{}^{\circ}}$
7026.041	8		14228.842	13088_3_ – $27317_{3}^{{}^{\circ}}$
7025.379	1		14230.183	$15618_{3}^{{}^{\circ}}$ – 29849_4_
7025.188	4		14230.570	$22855_{3}^{{}^{\circ}}$ – 37085_4_
7023.776	8		14233.431	11802_2_ – $26036_{3}^{{}^{\circ}}$
7023.636	25	1	14233.715	$11877_{1}^{{}^{\circ}}$ – 26111_1_
7023.523	100	3	14233.944	16554_6_ – $30788_{0}^{{}^{\circ}}$
7023.145	100	3	14234.710	$19039_{2}^{{}^{\circ}}$ – 33273_1_
7022.847	2		14235.314	$23655_{4}^{{}^{\circ}}$ – 37890_5_
7022.619	8		14235.776	7502_3_ – $21738_{2}^{{}^{\circ}}$
7021.899	40		14237.236	17959_4_ – $32197_{3}^{{}^{\circ}}$
7021.784	2		14237.469	21575_2_ – $35812_{3}^{{}^{\circ}}$
7021.668	3		14237.704	$23655_{4}^{{}^{\circ}}$ – 37892_4_
7021.279	150	8	14238.493	15970_3_ – $30208_{2}^{{}^{\circ}}$
7020.476	100	1	14240.121	15493_4_ – $29733_{0}^{{}^{\circ}}$
7019.970	4		14241.148	$14032_{2}^{{}^{\circ}}$ – 28273_2_
7018.784	3		14243.554	$19588_{0}^{{}^{\circ}}$ – 33831_4_
7018.563	500	75	14244.003	0_2_ – $14243_{1}^{{}^{\circ}}$
7015.311	100	3	14250.605	$10414_{4}^{{}^{\circ}}$ – 24664_3_
7014.965	100	4	14251.308	15493_4_ – $29744_{3}^{{}^{\circ}}$
7014.873	50	1	14251.495	$15166_{3}^{{}^{\circ}}$ – 29418_2_
7013.617	8		14254.047	5563_1_ – $19817_{1}^{{}^{\circ}}$
7013.171	1		14254.954	18699_2_ – $32954_{2}^{{}^{\circ}}$
7007.092	100	4	14267.321	19532_4_ – $33799_{4}^{{}^{\circ}}$
7006.655	4		14268.210	19532_4_ – $33800_{3}^{{}^{\circ}}$
7006.113	50	2	14269.314	20054_2_ – $34324_{2}^{{}^{\circ}}$
7005.825	8		14269.901	$19039_{2}^{{}^{\circ}}$ – 33309_2_
7002.881	200	8	14275.900	14204_5_ – $28480_{4}^{{}^{\circ}}$
7000.806	500	75	14280.131	9804_5_ – $24084_{6}^{{}^{\circ}}$
6999.624	100	3	14282.543	$18011_{0}^{{}^{\circ}}$ – 32293_5_
6998.247	100	2	14285.353	15970_3_ – $30255_{3}^{{}^{\circ}}$
6997.412	50	1	14287.058	$14243_{1}^{{}^{\circ}}$ – 28531_2_
6996.812	1		14288.283	19713_3_ – $34001_{4}^{{}^{\circ}}$
6996.757	501		14288.395	8111_4_ – $22399_{0}^{{}^{\circ}}$
6996.202	8		14289.528	$18809_{4}^{{}^{\circ}}$ – 33099_3_
6995.141	75	2	14291.696	$21539_{4}^{{}^{\circ}}$ – 35831_3_
6994.736	100	3	14292.523	$17501_{0}^{{}^{\circ}}$ – 31793_4_
6993.988	100	2	14294.052	11802_2_ – $26096_{3}^{{}^{\circ}}$
6989.657	1000	150	14302.909	$7795_{4}^{{}^{\circ}}$ – 22098_4_
6988.871	20	1	14304.517	$16346_{4}^{{}^{\circ}}$ – 30651_3_
6987.790	20	2	14306.730	$20566_{4}^{{}^{\circ}}$ – 34873_4_
6987.444	3		14307.439	$20566_{4}^{{}^{\circ}}$ – 34874_3_
6986.565	3		14309.239	$21738_{2}^{{}^{\circ}}$ – 36047_2_
6986.159	2		14310.070	$22508_{2}^{{}^{\circ}}$ – 36818_2_
6986.031	150	4	14310.332	11802_2_ – $26113_{2}^{{}^{\circ}}$
6985.783	10	1	14310.840	21575_2_ – $35885_{2}^{{}^{\circ}}$
6984.952	3		14312.543	19532_4_ – $33844_{0}^{{}^{\circ}}$
6984.759	5		14312.939	$17224_{2}^{{}^{\circ}}$ – 31537_3_
6984.585	4		14313.295	8800_4_ – $23113_{4}^{{}^{\circ}}$
6983.965	5		14314.566	$22338_{3}^{{}^{\circ}}$ – 36653_2_
6982.752	3		14317.052	20054_2_ – $34371_{2}^{{}^{\circ}}$
6982.608	50	2	14317.348	$23015_{0}^{{}^{\circ}}$ – 37332_6_
6981.082	100	3	14320.477	$13175_{4}^{{}^{\circ}}$ – 27495_4_
6980.543	4	1	14321.583	$20724_{0}^{{}^{\circ}}$ – 35046_1_
6979.944	1		14322.812	18431_3_ – $32754_{3}^{{}^{\circ}}$
6978.753	2		14325.256	17959_4_ – $32285_{3}^{{}^{\circ}}$
6978.241	50	2	14326.307	$14032_{2}^{{}^{\circ}}$ – 28358_3_
6977.875	3		14327.059	$16217_{2}^{{}^{\circ}}$ – 30544_2_
6977.462	25	1	14327.907	$13945_{3}^{{}^{\circ}}$ – 28273_2_
6977.061	3	1	14328.730	$19503_{3}^{{}^{\circ}}$ – 33831_4_
6976.839	2		14329.186	22098_4_ – $36427_{3}^{{}^{\circ}}$
6974.446	10		14334.103	$22877_{1}^{{}^{\circ}}$ – 37211_5_
6974.381	1		14334.236	17959_4_ – $32294_{4}^{{}^{\circ}}$
6970.054	5	1	14343.135	$18069_{3}^{{}^{\circ}}$ – 32412_4_
6969.779	2		14343.701	18699_2_ – $33043_{3}^{{}^{\circ}}$
6969.301	100	3	14344.685	15863_2_ – $30208_{2}^{{}^{\circ}}$
6966.905	3		14349.618	$22399_{0}^{{}^{\circ}}$ – 36749_6_
6966.064	3h		14351.350	$20522_{2}^{{}^{\circ}}$ – 34874_3_
6965.946	5001	4	14351.593	$19039_{2}^{{}^{\circ}}$ – 33390_1_
6965.240	5		14353.048	$19948_{4}^{{}^{\circ}}$ – 34301_4_
6964.271	50	1	14355.045	2869_3_ – $17224_{2}^{{}^{\circ}}$
6963.930	50	1	14355.748	$14206_{4}^{{}^{\circ}}$ – 28562_4_
6962.862	75	2	14357.950	$16783_{4}^{{}^{\circ}}$ – 31141_3_
6962.310	100	4	14359.088	$15490_{0}^{{}^{\circ}}$ – 29849_4_
6960.232	4		14363.375	$17411_{3}^{{}^{\circ}}$ – 31774_3_
6957.265	1		14369.501	$19039_{2}^{{}^{\circ}}$ – 33408_3_
6956.113	1		14371.880	16351_0_ – $30723_{1}^{{}^{\circ}}$
6955.315	100	3	14373.529	13297_4_ – $27670_{3}^{{}^{\circ}}$
6954.655	200	5	14374.893	6362_2_ – $20737_{1}^{{}^{\circ}}$
6954.387	2		14375.447	20054_2_ – $34430_{3}^{{}^{\circ}}$
6952.796	1		14378.737	$18930_{3}^{{}^{\circ}}$ – 33309_2_
6952.189	2		14379.992	18574_1_ – $32954_{2}^{{}^{\circ}}$
6951.654	20	1	14381.099	3687_2_ – $18069_{3}^{{}^{\circ}}$
6951.259	5	1	14381.916	$22399_{0}^{{}^{\circ}}$ – 36781_5_
6950.990	5		14382.473	17398_3_ – $31780_{3}^{{}^{\circ}}$
6950.990	5		14382.473	$17411_{3}^{{}^{\circ}}$ – 31793_4_
6949.754	10		14385.030	13962_1_ – $28347_{2}^{{}^{\circ}}$
6948.391	100	**3**	14387.852	7502_3_ – $21890_{3}^{{}^{\circ}}$
6948.205	300	20	14388.237	15493_4_ – $29881_{4}^{{}^{\circ}}$
6948.090	75	1	14388.475	$10526_{3}^{{}^{\circ}}$ – 24915_3_
6947.914	100	3	14388.840	7280_2_ – $21668_{1}^{{}^{\circ}}$
6947.783	2		14389.111	$18069_{3}^{{}^{\circ}}$ – 32458_4_
6946.599	50	3	14391.564	15863_2_ – $30255_{3}^{{}^{\circ}}$
6946.210	100	3	14392.370	$15618_{3}^{{}^{\circ}}$ – 30011_3_
6945.488	200	8	14393.866	$8243_{2}^{{}^{\circ}}$ – 22637_3_
6944.926	2		14395.031	$21902_{4}^{{}^{\circ}}$ – 36297_4_
6944.773	8		14395.348	$15618_{3}^{{}^{\circ}}$ – 30014_4_
6944.611	8		14395.684	$15736_{1}^{{}^{\circ}}$ – 30132_2_
6943.612	800	200	14397.755	9804_5_ – $24202_{4}^{{}^{\circ}}$
6942.535	150	8	14399.988	7502_3_ – $21902_{4}^{{}^{\circ}}$
6940.327	50	3	14404.569	$18053_{4}^{{}^{\circ}}$ – 32458_4_
6940.037	3		14405.171	18549_2_ – $32954_{2}^{{}^{\circ}}$
6937.618	10		14410.194	13962_1_ – $28372_{1}^{{}^{\circ}}$
6937.033	8		14411.409	$21165_{3}^{{}^{\circ}}$ – 35576_3_
6936.651	150	4	14412.203	12847_3_ – $27260_{3}^{{}^{\circ}}$
6936.223	10		14413.092	$13945_{3}^{{}^{\circ}}$ – 28358_3_
6934.839	4	1	14415.969	$22669_{3}^{{}^{\circ}}$ – 37085_4_
6934.488	15	1	14416.698	$13175_{4}^{{}^{\circ}}$ – 27591_5_
6934.274	100b	5	14417.143	15863_2_ – $30281_{1}^{{}^{\circ}}$
6934.232	100b	3	14417.231	$14465_{2}^{{}^{\circ}}$ – 28882_2_
6933.836	2		14418.054	12847_3_ – $27266_{4}^{{}^{\circ}}$
6932.227	15	1	14421.400	$19986_{6}^{{}^{\circ}}$ – 34407_6_
6931.784	3h		14422.322	14226_0_ – $28649_{1}^{{}^{\circ}}$
6930.094	5		14425.839	$22877_{0}^{{}^{\circ}}$ – 37303_4_
6928.959	3		14428.202	21645_4_ – $36074_{3}^{{}^{\circ}}$
6927.721	2		14430.780	18431_3_ – $32862_{4}^{{}^{\circ}}$
6926.311	2		14433.718	$16217_{2}^{{}^{\circ}}$ – 30651_3_
6925.566	8		14435.271	$14243_{1}^{{}^{\circ}}$ – 28679_2_
6925.471	10	1	14435.469	19532_4_ – $33967_{3}^{{}^{\circ}}$
6925.040	4		14436.367	$22338_{3}^{{}^{\circ}}$ – 36775_3_
6924.657	50	1	14437.166	$13175_{4}^{{}^{\circ}}$ – 27612_3_
6920.834	1		14445.141	19273_2_ – $33718_{2}^{{}^{\circ}}$
6920.391	5		14446.065	$21890_{3}^{{}^{\circ}}$ – 36336_3_
6920.037	150	5	14446.804	$18011_{0}^{{}^{\circ}}$ – 32458_4_
6919.415	1		14448.103	$22855_{3}^{{}^{\circ}}$ – 37303_4_
6916.129	200	10	14454.967	9804_5_ – $24259_{4}^{{}^{\circ}}$
6914.713	100	2	14457.928	7280_2_ – $21738_{2}^{{}^{\circ}}$
6913.173	2		14461.148	$22669_{3}^{{}^{\circ}}$ – 37131_3_
6913.051	4		14461.403	19273_2_ – $33734_{2}^{{}^{\circ}}$
6912.694	2		14462.150	18699_2_ – $33161_{1}^{{}^{\circ}}$
6911.222	800r	200	14465.231	0_2_ – $14465_{2}^{{}^{\circ}}$
6909.222	1001	1	14469.418	12847_3_ – $27317_{3}^{{}^{\circ}}$
6908.989	100	2	14469.906	9804_5_ – $24274_{0}^{{}^{\circ}}$
6908.630	2		14470.658	22401_1_ – $36871_{2}^{{}^{\circ}}$
6908.599	2h		14470.723	22401_1_ – $36871_{2}^{{}^{\circ}}$
6906.698	20	1	14474.705	$19986_{6}^{{}^{\circ}}$ – 34460_5_
6905.856	40	1	14476.470	14204_5_ – $28680_{4}^{{}^{\circ}}$
6904.578	50		14479.150	17959_4_ – $32439_{4}^{{}^{\circ}}$
6904.468	8		14479.380	21594_3_ – $36074_{3}^{{}^{\circ}}$
6903.317	8		14481.795	$16783_{4}^{{}^{\circ}}$ – 31265_3_
6902.957	10		14482.550	$18069_{3}^{{}^{\circ}}$ – 32551_3_
6902.767	2		14482.948	$19948_{4}^{{}^{\circ}}$ – 34431_3_
6902.210	150	3	14484.117	11802_2_ – $26287_{1}^{{}^{\circ}}$
6901.357	10		14485.907	$22399_{0}^{{}^{\circ}}$ – 36885_4_
6901.019	3		14486.617	21645_4_ – $36132_{0}^{{}^{\circ}}$
6900.762	100	5	14487.156	$16783_{4}^{{}^{\circ}}$ – 31271_5_
6900.442	10		14487.828	$18930_{3}^{{}^{\circ}}$ – 33418_4_
6898.073	25		14492.804	$20214_{3}^{{}^{\circ}}$ – 34707_3_
6897.277	20		14494.476	$17501_{0}^{{}^{\circ}}$ – 31995_4_
6895.227	10		14498.786	$19948_{4}^{{}^{\circ}}$ – 34447_4_
6895.116	8		14499.019	21575_2_ – $36074_{3}^{{}^{\circ}}$
6894.229	50	4	14500.884	$10414_{4}^{{}^{\circ}}$ – 24915_3_
6893.626	10	1	14502.153	$20543_{0}^{{}^{\circ}}$ – 35046_1_
6892.247	200	5	14505.054	17166_5_ – $31671_{4}^{{}^{\circ}}$
6889.052	8	1	14511.781	$17501_{0}^{{}^{\circ}}$ – 32012_4_
6888.832	100	3	14512.245	11601_1_ – $26113_{2}^{{}^{\circ}}$
6888.158	50	2	14513.665	$15618_{3}^{{}^{\circ}}$ – 30132_2_
6886.404	200	10	14517.362	3865_1_ – $18382_{0}^{{}^{\circ}}$
6885.964	2		14518.289	$17411_{3}^{{}^{\circ}}$ – 31929_3_
6885.721	1		14518.802	$20922_{2}^{{}^{\circ}}$ – 35440_3_
6883.849	75		14522.750	$20566_{4}^{{}^{\circ}}$ – 35089_3_
6883.311	200	10	14523.885	15493_4_ – $30017_{3}^{{}^{\circ}}$
6883.135	50	4	14524.256	$15490_{0}^{{}^{\circ}}$ – 30014_4_
6882.807	200	8	14524.948	13847_2_ – $28372_{1}^{{}^{\circ}}$
6881.932	2		14526.795	$20322_{0}^{{}^{\circ}}$ – 34849_5_
6881.674	5		14527.340	19273_2_ – $33800_{3}^{{}^{\circ}}$
6881.250	5		14528.235	20054_2_ – $34583_{3}^{{}^{\circ}}$
6880.945	1		14528.879	$18930_{3}^{{}^{\circ}}$ – 33459_4_
6879.376	1		14532.193	21645_4_ – $36178_{4}^{{}^{\circ}}$
6877.469	2		14536.222	20054_2_ – $34591_{1}^{{}^{\circ}}$
6875.440	2		14540.512	$16217_{2}^{{}^{\circ}}$ – 30758_2_
6874.984	10		14541.476	4961_4_ – $19503_{3}^{{}^{\circ}}$
6874.752	200	10	14541.967	2869_3_ – $17411_{3}^{{}^{\circ}}$
6874.192	50b		14543.151	$16783_{4}^{{}^{\circ}}$ – 31326_4_
6872.234	8		14547.295	15970_3_ – $30517_{4}^{{}^{\circ}}$
6871.271	50		14549.334	$22855_{3}^{{}^{\circ}}$ – 37404_3_
6870.988	150	3	14549.933	$15736_{1}^{{}^{\circ}}$ – 30286_1_
6870.911	2		14550.096	$21539_{4}^{{}^{\circ}}$ – 36089_4_
6870.807	2		14550.316	$17224_{2}^{{}^{\circ}}$ – 31774_3_
6870.727	150	3	14550.486	17166_5_ – $31716_{0}^{{}^{\circ}}$
6868.455	100	3	14555.299	13297_4_ – $27852_{0}^{{}^{\circ}}$
6866.756	100s	2	14558.900	8111_4_ – $22669_{3}^{{}^{\circ}}$
6866.368	150	3	14559.723	6362_2_ – $20922_{2}^{{}^{\circ}}$
6866.157	100	2	14560.170	11802_2_ – $26363_{2}^{{}^{\circ}}$
6863.104	8		14566.647	$20522_{2}^{{}^{\circ}}$ – 35089_3_
6862.873	100	2	14567.138	$19039_{2}^{{}^{\circ}}$ – 33606_2_
6858.317	1		14576.814	16351_0_ – $30928_{1}^{{}^{\circ}}$
6857.436	2		14578.687	$21252_{2}^{{}^{\circ}}$ – 35831_3_
6855.689	100	3	14582.402	13088_3_ – $27670_{3}^{{}^{\circ}}$
6855.315	150	4	14583.198	15970_3_ – $30553_{2}^{{}^{\circ}}$
6854.107	400	10	14585.768	13088_3_ – $27674_{2}^{{}^{\circ}}$
6853.511	100	4	14587.036	16554_6_ – $31141_{0}^{{}^{\circ}}$
6852.344	50	2	14589.521	$15166_{3}^{{}^{\circ}}$ – 29756_4_
6852.099	1		14590.042	21143_5_ – $35733_{4}^{{}^{\circ}}$
6850.111	8		14594.276	21594_3_ – $36188_{2}^{{}^{\circ}}$
6849.623	25		14595.316	18699_2_ – $33294_{3}^{{}^{\circ}}$
6848.697	3		14597.290	$18930_{3}^{{}^{\circ}}$ – 33527_4_
6848.279	2		14598.181	$21738_{2}^{{}^{\circ}}$ – 36336_3_
6847.989	3		14598.799	$18809_{4}^{{}^{\circ}}$ – 33408_3_
6847.517	4		14599.805	12847_3_ – $27447_{2}^{{}^{\circ}}$
6846.603	15	1	14601.754	$17411_{3}^{{}^{\circ}}$ – 32012_4_
6843.556	3		14608.255	$18809_{4}^{{}^{\circ}}$ – 33418_4_
6842.724	50		14610.031	7280_2_ – $21890_{3}^{{}^{\circ}}$
6841.962	8		14611.659	18431_3_ – $33043_{3}^{{}^{\circ}}$
6841.690	10		14612.239	$22163_{4}^{{}^{\circ}}$ – 36775_3_
6840.993	2		14613.728	$21902_{4}^{{}^{\circ}}$ – 36515_3_
6840.899	2		14613.929	21575_2_ – $36188_{2}^{{}^{\circ}}$
6839.287	100	2	14617.373	$13945_{3}^{{}^{\circ}}$ – 28562_4_
6838.979	2		14618.032	$16346_{4}^{{}^{\circ}}$ – 30964_3_
6838.902	8		14618.196	$22163_{4}^{{}^{\circ}}$ – 36781_5_
6836.881	50		14622.518	$20423_{1}^{{}^{\circ}}$ – 35046_1_
6836.651	3		14623.009	$22508_{2}^{{}^{\circ}}$ – 37131_3_
6835.613	8		14625.230	$21252_{2}^{{}^{\circ}}$ – 35877_2_
6834.925	400	50	14626.702	4961_4_ – $19588_{0}^{{}^{\circ}}$
6834.780	5b		14627.012	$20522_{2}^{{}^{\circ}}$ – 35149_2_
6834.585	1		14627.430	$19817_{1}^{{}^{\circ}}$ – 34444_2_
6833.697	3		14629.330	21645_4_ – $36275_{0}^{{}^{\circ}}$
6833.373	10		14630.024	$17411_{3}^{{}^{\circ}}$ – 32041_2_
6831.719	3		14633.566	$22669_{3}^{{}^{\circ}}$ – 37303_4_
6831.621	2		14633.776	$22141_{3}^{{}^{\circ}}$ – 36775_3_
6831.249	8		14634.573	$18930_{3}^{{}^{\circ}}$ – 33564_3_
6829.339	100	3	14638.666	$14206_{4}^{{}^{\circ}}$ – 28845_4_
6829.212	8		14638.938	17073_1_ – $31712_{1}^{{}^{\circ}}$
6829.037	500	25	14639.313	7502_3_ – $22141_{3}^{{}^{\circ}}$
6826.296	501	2	14645.191	$22163_{4}^{{}^{\circ}}$ – 36808_3_
6824.678	400	20	14648.663	$11241_{3}^{{}^{\circ}}$ – 25890_2_
6824.380	1		14649.303	$18809_{4}^{{}^{\circ}}$ – 33459_4_
6824.097	8		14649.911	$19039_{2}^{{}^{\circ}}$ – 33689_2_
6823.911	10	1	14650.310	19532_4_ – $34182_{0}^{{}^{\circ}}$
6823.500	150	4	14651.192	13297_4_ – $27948_{4}^{{}^{\circ}}$
6819.796	10		14659.150	$20214_{3}^{{}^{\circ}}$ – 34874_3_
6819.549	5		14659.681	$14481_{6}^{{}^{\circ}}$ – 29141_5_
6818.996	5		14660.870	7502_3_ – $22163_{4}^{{}^{\circ}}$
6817.248	2		14664.629	22098_4_ – $36762_{3}^{{}^{\circ}}$
6816.821	25		14665.547	13847_2_ – $28513_{2}^{{}^{\circ}}$
6816.510	50	2	14666.216	$21165_{3}^{{}^{\circ}}$ – 35831_3_
6816.510	50	2	14666.216	$22855_{3}^{{}^{\circ}}$ – 37521_3_
6816.246	50w		14666.784	15493_4_ – $30160_{4}^{{}^{\circ}}$
6815.604	100	2	14668.166	$15166_{3}^{{}^{\circ}}$ – 29835_3_
6814.184	2		14671.222	$21539_{4}^{{}^{\circ}}$ – 36210_5_
6813.602	10		14672.476	$16217_{2}^{{}^{\circ}}$ – 30889_1_
6811.958	50	1	14676.017	$18930_{3}^{{}^{\circ}}$ – 33606_2_
6811.725	4		14676.519	$22141_{3}^{{}^{\circ}}$ – 36818_2_
6809.504	100	3	14681.306	$11241_{3}^{{}^{\circ}}$ – 25923_4_
6809.311	150	4	14681.722	$12114_{2}^{{}^{\circ}}$ – 26796_3_
6809.103	400	10	14682.170	$19039_{2}^{{}^{\circ}}$ – 33721_2_
6808.530	75	2	14683.406	$18053_{4}^{{}^{\circ}}$ – 32737_5_
6808.373	2		14683.744	$20737_{1}^{{}^{\circ}}$ – 35421_1_
6807.314	100	3	14686.029	11601_1_ – $26287_{1}^{{}^{\circ}}$
6807.108	8	1	14686.473	$22399_{0}^{{}^{\circ}}$ – 37085_4_
6807.035	15	1	14686.631	13962_1_ – $28649_{1}^{{}^{\circ}}$
6805.746	100	2	14689.412	15863_2_ – $30553_{2}^{{}^{\circ}}$
6803.292	10		14694.711	$18614_{1}^{{}^{\circ}}$ – 33309_2_
6802.783	100	3	14695.810	13088_3_ – $27784_{2}^{{}^{\circ}}$
6802.410	3		14696.616	$20724_{0}^{{}^{\circ}}$ – 35421_1_
6801.772	2		14697.995	$22396_{1}^{{}^{\circ}}$ – 37094_2_
6800.461	100	4	14700.828	$17501_{0}^{{}^{\circ}}$ – 32202_5_
6799.707	2		14702.458	22098_4_ – $36800_{4}^{{}^{\circ}}$
6798.743	100	3	14704.543	$17847_{2}^{{}^{\circ}}$ – 32551_3_
6798.483	150	3	14705.105	11802_2_ – $26508_{3}^{{}^{\circ}}$
6795.797	100	2	14710.917	13962_1_ – $28673_{2}^{{}^{\circ}}$
6795.046	75	2	14712.543	$18069_{3}^{{}^{\circ}}$ – 32781_4_
6794.965	25	1	14712.719	$21165_{3}^{{}^{\circ}}$ – 35877_2_
6794.795	8	1	14713.087	$19588_{0}^{{}^{\circ}}$ – 34301_4_
6792.900	15	1	14717.191	19713_3_ – $34430_{3}^{{}^{\circ}}$
6792.674	4		14717.681	$18809_{4}^{{}^{\circ}}$ – 33527_4_
6791.232	400	25	14720.806	8800_4_ – $23521_{3}^{{}^{\circ}}$
6789.000	100		14725.645	$18011_{0}^{{}^{\circ}}$ – 32737_5_
6788.837	300	25	14725.999	$11197_{0}^{{}^{\circ}}$ – 25923_4_
6788.730	50h	1	14726.231	$20423_{1}^{{}^{\circ}}$ – 35149_2_
6788.343	20		14727.071	$18069_{3}^{{}^{\circ}}$ – 32796_3_
6788.180	50		14727.424	18699_2_ – $33427_{2}^{{}^{\circ}}$
6787.913	25		14728.004	$14206_{4}^{{}^{\circ}}$ – 28934_3_
6787.913	25		14728.004	$18053_{4}^{{}^{\circ}}$ – 32781_4_
6787.734	300	25	14728.392	14204_5_ – $28932_{4}^{{}^{\circ}}$
6784.927	10		14734.485	$19039_{2}^{{}^{\circ}}$ – 33773_1_
6784.807	50	2	14734.746	$22669_{3}^{{}^{\circ}}$ – 37404_3_
6782.498	1		14739.762	22098_4_ – $36837_{0}^{{}^{\circ}}$
6781.676	3		14741.549	13847_2_ – $28589_{3}^{{}^{\circ}}$
6781.234	3		14742.509	$18053_{4}^{{}^{\circ}}$ – 32796_3_
6780.415	400	100	14744.290	8111_4_ – $22855_{3}^{{}^{\circ}}$
6780.126	400	1	14744.919	$8243_{2}^{{}^{\circ}}$ – 22988_2_
6779.323	100	1	14746.665	7502_3_ – $22248_{2}^{{}^{\circ}}$
6779.085	4		14747.183	$16217_{2}^{{}^{\circ}}$ – 30964_3_
6778.312	400	40	14748.865	3865_1_ – $18614_{1}^{{}^{\circ}}$
6776.230	100	3	14753.396	$16783_{4}^{{}^{\circ}}$ – 31537_3_
6775.501	2		14754.984	$18809_{4}^{{}^{\circ}}$ – 33564_3_
6775.101	15	2	14755.855	$22338_{3}^{{}^{\circ}}$ – 37094_2_
6774.254	1		14757.700	$21539_{4}^{{}^{\circ}}$ – 36297_4_
6774.086	8		14758.066	22401_1_ – $37159_{1}^{{}^{\circ}}$
6773.770	40	1	14758.754	$18930_{3}^{{}^{\circ}}$ – 33689_2_
6772.237	50b		14762.095	17398_3_ – $32160_{2}^{{}^{\circ}}$
6772.176	400	15	14762.228	15493_4_ – $30255_{3}^{{}^{\circ}}$
6771.623	5		14763.433	$21890_{3}^{{}^{\circ}}$ – 36653_2_
6770.175	5		14766.591	8111_4_ – $22877_{0}^{{}^{\circ}}$
6768.511	3		14770.221	$18011_{0}^{{}^{\circ}}$ – 32781_4_
6765.683	200	100	14776.395	$18614_{1}^{{}^{\circ}}$ – 33390_1_
6764.987	1		14777.915	$21738_{2}^{{}^{\circ}}$ – 36515_3_
6763.532	8	1	14781.094	$19516_{2}^{{}^{\circ}}$ – 34298_1_
6763.340	50	2	14781.514	21645_4_ – $36427_{3}^{{}^{\circ}}$
6760.667	100	3	14787.358	17166_5_ – $31953_{4}^{{}^{\circ}}$
6760.400	2		14787.942	21594_3_ – $36382_{4}^{{}^{\circ}}$
6758.716	2		14791.627	15970_3_ – $30761_{3}^{{}^{\circ}}$
6758.521	5		14792.053	$22338_{3}^{{}^{\circ}}$ – 37131_3_
6758.202	200	8	14792.752	$17501_{0}^{{}^{\circ}}$ – 32293_5_
6757.349	15		14794.619	17959_4_ – $32754_{3}^{{}^{\circ}}$
6757.105	100	5	14795.153	$16346_{4}^{{}^{\circ}}$ – 31141_3_
6756.454	800b	75	14796.579	2558_0_ – $17354_{1}^{{}^{\circ}}$
6756.323	15		14796.866	20054_2_ – $34851_{2}^{{}^{\circ}}$
6756.323	15		14796.866	20054_2_ – $34851_{2}^{{}^{\circ}}$
6755.663	4		14798.311	$19503_{3}^{{}^{\circ}}$ – 34301_4_
6755.475	20	1	14798.723	17398_3_ – $32197_{3}^{{}^{\circ}}$
6755.305	2		14799.096	$22508_{2}^{{}^{\circ}}$ – 37307_3_
6754.265	150	4	14801.374	13847_2_ – $28649_{1}^{{}^{\circ}}$
6753.657	150	4	14802.707	6362_2_ – $21165_{3}^{{}^{\circ}}$
6751.426	100	3	14807.598	$15736_{1}^{{}^{\circ}}$ – 30544_2_
6749.315	50	1	14812.230	19532_4_ – $34344_{4}^{{}^{\circ}}$
6747.402	2		14816.429	$21668_{1}^{{}^{\circ}}$ – 36485_2_
6747.172	5		14816.934	$17224_{2}^{{}^{\circ}}$ – 32041_2_
6746.137	75	2	14819.207	$19588_{0}^{{}^{\circ}}$ – 34407_6_
6743.199	10		14825.664	13847_2_ – $28673_{2}^{{}^{\circ}}$
6742.881	200	8	14826.363	12847_3_ – $27674_{2}^{{}^{\circ}}$
6741.924	100	2	14828.468	13847_2_ – $28676_{3}^{{}^{\circ}}$
6739.963	50h		14832.782	22401_1_ – $37234_{2}^{{}^{\circ}}$
6738.179	200	5	14836.709	7502_3_ – $22338_{3}^{{}^{\circ}}$
6737.643	50	8	14837.889	19273_2_ – $34111_{1}^{{}^{\circ}}$
6737.281	50		14838.687	$20566_{4}^{{}^{\circ}}$ – 35405_3_
6735.686	2		14842.201	$7795_{4}^{{}^{\circ}}$ – 22637_3_
6735.369	100	2	14842.899	15970_3_ – $30812_{2}^{{}^{\circ}}$
6735.128	100	2	14843.430	13297_4_ – $28140_{4}^{{}^{\circ}}$
6734.667	20		14844.446	$15166_{3}^{{}^{\circ}}$ – 30011_3_
6734.003	4		14845.910	$22248_{2}^{{}^{\circ}}$ – 37094_2_
6733.748	150	5	14846.472	14204_5_ – $29050_{0}^{{}^{\circ}}$
6733.314	8		14847.429	$15166_{3}^{{}^{\circ}}$ – 30014_4_
6732.650	50	3	14848.893	11802_2_ – $26651_{2}^{{}^{\circ}}$
6731.047	25		14852.430	18574_1_ – $33427_{2}^{{}^{\circ}}$
6729.932	50	1	14854.890	8800_4_ – $23655_{4}^{{}^{\circ}}$
6728.757	300	5	14857.484	17166_5_ – $32023_{0}^{{}^{\circ}}$
6728.117	150	5	14858.898	$13175_{4}^{{}^{\circ}}$ – 28034_5_
6727.649	100s	2	14859.931	15863_2_ – $30723_{1}^{{}^{\circ}}$
6727.459	500	75	14860.351	5563_1_ – $20423_{1}^{{}^{\circ}}$
6726.946	10	2	14861.484	7280_2_ – $22141_{3}^{{}^{\circ}}$
6726.310	100	2	14862.889	$19986_{6}^{{}^{\circ}}$ – 34849_5_
6725.482	2		14864.719	$22877_{1}^{{}^{\circ}}$ – 37742_6_
6725.128	1		14865.502	18431_3_ – $33297_{2}^{{}^{\circ}}$
6723.120	2		14869.941	19713_3_ – $34583_{3}^{{}^{\circ}}$
6722.852	1		14870.534	$22877_{1}^{{}^{\circ}}$ – 37748_2_
6721.958	5		14872.512	$19588_{0}^{{}^{\circ}}$ – 34460_5_
6721.703	1		14873.076	$21902_{4}^{{}^{\circ}}$ – 36775_3_
6721.089	25		14874.435	$20214_{3}^{{}^{\circ}}$ – 35089_3_
6720.699	1		14875.298	$23015_{0}^{{}^{\circ}}$ – 37890_5_
6719.630	20		14877.664	18549_2_ – $33427_{2}^{{}^{\circ}}$
6719.307	2		14878.380	$16217_{2}^{{}^{\circ}}$ – 31095_1_
6719.198	200	5	14878.621	$10526_{3}^{{}^{\circ}}$ – 25405_4_
6719.011	3		14879.035	$21902_{4}^{{}^{\circ}}$ – 36781_5_
6717.582	1		14882.200	$21165_{3}^{{}^{\circ}}$ – 36047_2_
6717.384	100	3	14882.639	$15490_{0}^{{}^{\circ}}$ – 30372_6_
6716.215	3		14885.229	$21890_{3}^{{}^{\circ}}$ – 36775_3_
6715.515	100	2	14886.781	17398_3_ – $32285_{3}^{{}^{\circ}}$
6713.967	300	8	14890.213	6362_2_ – $21252_{2}^{{}^{\circ}}$
6713.602	100		14891.023	$18382_{0}^{{}^{\circ}}$ – 33273_1_
6712.838	2		14892.717	$22855_{3}^{{}^{\circ}}$ – 37748_2_
6712.194	5		14894.146	21594_3_ – $36488_{2}^{{}^{\circ}}$
6711.495	50		14895.698	17398_3_ – $32294_{4}^{{}^{\circ}}$
6711.249	100	4	14896.243	9804_5_ – $24701_{0}^{{}^{\circ}}$
6710.865	10		14897.096	$14206_{4}^{{}^{\circ}}$ – 29104_4_
6710.545	100	2	14897.806	15863_2_ – $30761_{3}^{{}^{\circ}}$
6710.545	100	2	14897.806	19532_4_ – $34430_{3}^{{}^{\circ}}$
6710.325	3		14898.295	$20522_{2}^{{}^{\circ}}$ – 35421_1_
6709.443	2		14900.253	$13945_{3}^{{}^{\circ}}$ – 28845_4_
6709.248	2		14900.686	$19948_{4}^{{}^{\circ}}$ – 34849_5_
6708.839	50	1	14901.595	$18930_{3}^{{}^{\circ}}$ – 33831_4_
6708.391	5		14902.590	17959_4_ – $32862_{4}^{{}^{\circ}}$
6708.283	5		14902.830	$14032_{2}^{{}^{\circ}}$ – 28934_3_
6707.733	3h		14904.052	$22399_{0}^{{}^{\circ}}$ – 37303_4_
6706.835	15	1	14906.047	$21902_{4}^{{}^{\circ}}$ – 36808_3_
6706.451	2		14906.901	$22877_{1}^{{}^{\circ}}$ – 37784_2_
6705.428	8		14909.175	$20922_{2}^{{}^{\circ}}$ – 35831_3_
6705.151	10		14909.791	$18053_{4}^{{}^{\circ}}$ – 32963_5_
6703.348	5		14913.801	21575_2_ – $36488_{2}^{{}^{\circ}}$
6703.091	2		14914.373	$19516_{2}^{{}^{\circ}}$ – 34431_3_
6702.576	1		14915.519	$21738_{2}^{{}^{\circ}}$ – 36653_2_
6701.373	10	1	14918.196	$20522_{2}^{{}^{\circ}}$ – 35440_3_
6701.373	10	1	14918.196	$21890_{3}^{{}^{\circ}}$ – 36808_3_
6701.010	2		14919.004	$16346_{4}^{{}^{\circ}}$ – 31265_3_
6700.798	8		14919.477	21143_5_ – $36062_{4}^{{}^{\circ}}$
6699.335	2		14922.735	$22163_{4}^{{}^{\circ}}$ – 37085_4_
6698.610	10		14924.350	$16346_{4}^{{}^{\circ}}$ – 31271_5_
6698.516	2		14924.559	$21165_{3}^{{}^{\circ}}$ – 36089_4_
6698.029	200	4	14925.644	$19948_{4}^{{}^{\circ}}$ – 34874_3_
6697.708	200	5	14926.360	3687_2_ – $18614_{1}^{{}^{\circ}}$
6697.139	5		14927.628	$19516_{2}^{{}^{\circ}}$ – 34444_2_
6696.996	1		14927.947	$21890_{3}^{{}^{\circ}}$ – 36818_2_
6696.886	1		14928.192	$19503_{3}^{{}^{\circ}}$ – 34431_3_
6696.140	200	5	14929.855	$12114_{2}^{{}^{\circ}}$ – 27044_3_
6695.175	8	1	14932.007	$20867_{7}^{{}^{\circ}}$ – 35799_6_
6694.492	100	2	14933.530	$15618_{3}^{{}^{\circ}}$ – 30552_4_
6694.002	100	1	14934.623	$14206_{4}^{{}^{\circ}}$ – 29141_5_
6693.205	75		14936.401	12847_3_ – $27784_{2}^{{}^{\circ}}$
6690.906	50		14941.534	$22877_{0}^{{}^{\circ}}$ – 37819_4_
6690.271	4		14942.952	22098_4_ – $37041_{4}^{{}^{\circ}}$
6689.783	5		14944.042	$19503_{3}^{{}^{\circ}}$ – 34447_4_
6687.521	150	3	14949.096	15863_2_ – $30812_{2}^{{}^{\circ}}$
6686.214	10		14952.019	$18011_{0}^{{}^{\circ}}$ – 32963_5_
6685.701	4		14953.166	$14465_{2}^{{}^{\circ}}$ – 29418_2_
6684.687	8		14955.434	13962_1_ – $28917_{2}^{{}^{\circ}}$
6684.049	50	2	14956.862	$14465_{2}^{{}^{\circ}}$ – 29422_1_
6683.991	8b		14956.991	$17501_{0}^{{}^{\circ}}$ – 32458_4_
6683.368	200	3	14958.386	$8243_{2}^{{}^{\circ}}$ – 23201_3_
6682.838	5		14959.572	5563_1_ – $20522_{2}^{{}^{\circ}}$
6681.486	15		14962.599	18699_2_ – $33662_{1}^{{}^{\circ}}$
6680.943	2		14963.815	$22855_{3}^{{}^{\circ}}$ – 37819_4_
6680.664	2		14964.440	$22338_{3}^{{}^{\circ}}$ – 37303_4_
6680.087	50	1	14965.733	$15166_{3}^{{}^{\circ}}$ – 30132_2_
6678.994	1		14968.182	$22338_{3}^{{}^{\circ}}$ – 37307_3_
6678.707	200	8	14968.825	7280_2_ – $22248_{2}^{{}^{\circ}}$
6677.964	15		14970.490	14226_0_ – $29197_{1}^{{}^{\circ}}$
6675.341	4		14976.373	$21539_{4}^{{}^{\circ}}$ – 36515_3_
6675.023	8		14977.086	$23015_{0}^{{}^{\circ}}$ – 37992_4_
6674.698	400	8	14977.816	2869_3_ – $17847_{2}^{{}^{\circ}}$
6674.339	5		14978.621	$20322_{0}^{{}^{\circ}}$ – 35300_5_
6673.590	200	5	14980.302	$16346_{4}^{{}^{\circ}}$ – 31326_4_
6673.428	5		14980.666	5563_1_ – $20543_{0}^{{}^{\circ}}$
6672.971	2		14981.692	21575_2_ – $36556_{1}^{{}^{\circ}}$
6672.383	1		14983.012	$21902_{4}^{{}^{\circ}}$ – 36885_4_
6671.684	3		14984.582	$21668_{1}^{{}^{\circ}}$ – 36653_2_
6670.741	1		14986.700	4961_4_ – $19948_{4}^{{}^{\circ}}$
6669.531	3		14989.419	$22141_{3}^{{}^{\circ}}$ – 37131_3_
6669.463	8		14989.572	$13945_{3}^{{}^{\circ}}$ – 28934_3_
6668.826	100		14991.004	$10414_{4}^{{}^{\circ}}$ – 25405_4_
6668.673	8		14991.348	19713_3_ – $34704_{3}^{{}^{\circ}}$
6668.673	8		14991.348	19713_3_ – $34704_{3}^{{}^{\circ}}$
6668.414	8		14991.930	$18614_{1}^{{}^{\circ}}$ – 33606_2_
6666.987	4		14995.139	$21890_{3}^{{}^{\circ}}$ – 36885_4_
6663.702	50	3	15002.531	8111_4_ – $23113_{4}^{{}^{\circ}}$
6662.270	800	200	15005.755	7502_3_ – $22508_{2}^{{}^{\circ}}$
6661.320	3		15007.895	$18382_{0}^{{}^{\circ}}$ – 33390_1_
6659.331	3		15012.378	19713_3_ – $34725_{3}^{{}^{\circ}}$
6659.266	2		15012.524	$21077_{0}^{{}^{\circ}}$ – 36089_4_
6659.118	10	2	15012.858	$22877_{0}^{{}^{\circ}}$ – 37890_5_
6658.869	2		15013.419	$22508_{2}^{{}^{\circ}}$ – 37521_3_
6658.678	400	15	15013.850	$11241_{3}^{{}^{\circ}}$ – 26255_4_
6658.071	2		15015.219	$22877_{0}^{{}^{\circ}}$ – 37892_4_
6657.879	3		15015.652	$20566_{4}^{{}^{\circ}}$ – 35582_5_
6656.876	50	1	15017.914	$15490_{0}^{{}^{\circ}}$ – 30508_5_
6656.708	4		15018.293	$22669_{3}^{{}^{\circ}}$ – 37688_3_
6656.503	1		15018.756	18699_2_ – $33718_{2}^{{}^{\circ}}$
6655.773	15		15020.403	15970_3_ – $30990_{3}^{{}^{\circ}}$
6655.494	50	2	15021.033	$15736_{1}^{{}^{\circ}}$ – 30758_2_
6655.071	5		15021.988	$18809_{4}^{{}^{\circ}}$ – 33831_4_
6654.236	10		15023.872	15493_4_ – $30517_{0}^{{}^{\circ}}$
6654.110	5		15024.157	15493_4_ – $30517_{4}^{{}^{\circ}}$
6652.225	2		15028.414	$16217_{2}^{{}^{\circ}}$ – 31245_2_
6651.371	5		15030.344	$18069_{3}^{{}^{\circ}}$ – 33099_3_
6650.561	50	1	15032.174	$15618_{3}^{{}^{\circ}}$ – 30651_3_
6649.487	1		15034.602	21143_5_ – $36178_{4}^{{}^{\circ}}$
6649.278	8	1	15035.075	18699_2_ – $33734_{2}^{{}^{\circ}}$
6648.347	5		15037.180	13847_2_ – $28884_{3}^{{}^{\circ}}$
6646.814	8		15040.648	17398_3_ – $32439_{4}^{{}^{\circ}}$
6646.323	20	1	15041.760	18549_2_ – $33591_{3}^{{}^{\circ}}$
6644.839	2		15045.119	$23655_{4}^{{}^{\circ}}$ – 38700_4_
6644.661	200	150	15045.522	9804_5_ – $24850_{6}^{{}^{\circ}}$
6644.550	4	1	15045.773	$18053_{4}^{{}^{\circ}}$ – 33099_3_
6644.029	50	2	15046.953	$17411_{3}^{{}^{\circ}}$ – 32458_4_
6643.501	3		15048.149	$16217_{2}^{{}^{\circ}}$ – 31265_3_
6643.120	8		15049.012	15970_3_ – $31019_{4}^{{}^{\circ}}$
6642.426	40b	1	15050.584	19532_4_ – $34583_{3}^{{}^{\circ}}$
6642.333	50b	4	15050.795	11601_1_ – $26651_{2}^{{}^{\circ}}$
6642.333	50b	4	15050.795	19273_2_ – $34324_{2}^{{}^{\circ}}$
6641.668	4		15052.302	13088_3_ – $28140_{4}^{{}^{\circ}}$
6641.439	4		15052.821	$13175_{4}^{{}^{\circ}}$ – 28227_4_
6641.006	4		15053.802	$20522_{2}^{{}^{\circ}}$ – 35576_3_
6639.726	8		15056.704	$18011_{0}^{{}^{\circ}}$ – 33068_6_
6638.912	150	20	15058.551	$11197_{0}^{{}^{\circ}}$ – 26255_4_
6638.774	8		15058.864	7280_2_ – $22338_{3}^{{}^{\circ}}$
6637.204	15	1	15062.426	$15490_{0}^{{}^{\circ}}$ – 30552_4_
6636.143	2		15064.834	15863_2_ – $30928_{1}^{{}^{\circ}}$
6633.784	50	2	15070.191	13847_2_ – $28917_{2}^{{}^{\circ}}$
6633.581	8		15070.652	$14481_{6}^{{}^{\circ}}$ – 29552_6_
6632.110	5		15073.995	21594_3_ – $36668_{2}^{{}^{\circ}}$
6631.568	40	1	15075.227	11802_2_ – $26878_{3}^{{}^{\circ}}$
6629.447	1		15080.050	$21738_{2}^{{}^{\circ}}$ – 36818_2_
6626.544	2		15086.656	17073_1_ – $32160_{2}^{{}^{\circ}}$
6626.125	50	1	15087.610	18574_1_ – $33662_{1}^{{}^{\circ}}$
6623.482	2		15093.630	21575_2_ – $36668_{2}^{{}^{\circ}}$
6621.329	1		15098.538	19273_2_ – $34371_{2}^{{}^{\circ}}$
6620.408	40	1	15100.639	12847_3_ – $27948_{4}^{{}^{\circ}}$
6620.248	2		15101.004	18699_2_ – $33800_{3}^{{}^{\circ}}$
6618.398	10	1	15105.225	17398_3_ – $32503_{2}^{{}^{\circ}}$
6618.163	200	8	15105.761	15970_3_ – $31075_{2}^{{}^{\circ}}$
6618.163	200	8	15105.761	14204_5_ – $29310_{4}^{{}^{\circ}}$
6617.626	5		15106.987	$18614_{1}^{{}^{\circ}}$ – 33721_2_
6617.512	75	4	15107.247	$10783_{2}^{{}^{\circ}}$ – 25890_2_
6615.071	50	2	15112.822	18549_2_ – $33662_{1}^{{}^{\circ}}$
6613.376	200	8	15116.695	7280_2_ – $22396_{1}^{{}^{\circ}}$
6613.280	4h		15116.914	21645_4_ – $36762_{3}^{{}^{\circ}}$
6609.668	5		15125.175	$20922_{2}^{{}^{\circ}}$ – 36047_2_
6609.031	5	1	15126.633	15863_2_ – $30990_{3}^{{}^{\circ}}$
6608.438	50	2	15127.991	17166_5_ – $32294_{4}^{{}^{\circ}}$
6606.790	25	2	15131.764	21143_5_ – $36275_{0}^{{}^{\circ}}$
6606.597	20	2	15132.206	$21165_{3}^{{}^{\circ}}$ – 36297_4_
6604.528	1		15136.947	$22855_{3}^{{}^{\circ}}$ – 37992_4_
6603.788	5	1	15138.643	19713_3_ – $34851_{2}^{{}^{\circ}}$
6603.618	75	5	15139.033	$15618_{3}^{{}^{\circ}}$ – 30758_2_
6603.026	20	2	15140.390	$17411_{3}^{{}^{\circ}}$ – 32551_3_
6602.771	100	8	15140.975	$19948_{4}^{{}^{\circ}}$ – 35089_3_
6601.452	3	1	15144.000	$22163_{4}^{{}^{\circ}}$ – 37307_3_
6600.730	50	2	15145.656	$16783_{4}^{{}^{\circ}}$ – 31929_3_
6599.183	3		15149.207	$21668_{1}^{{}^{\circ}}$ – 36818_2_
6599.183	3		15149.207	$22669_{3}^{{}^{\circ}}$ – 37819_4_
6597.542	2		15152.975	$15736_{1}^{{}^{\circ}}$ – 30889_1_
6596.750	10	1	15154.794	21645_4_ – $36800_{4}^{{}^{\circ}}$
6594.798	2		15159.280	$18614_{1}^{{}^{\circ}}$ – 33773_1_
6594.442	8	1	15160.098	18574_1_ – $33734_{2}^{{}^{\circ}}$
6593.940	500	150	15161.252	5563_1_ – $20724_{0}^{{}^{\circ}}$
6593.464	100	15	15162.347	16554_6_ – $31716_{0}^{{}^{\circ}}$
6591.485	400	75	15166.899	0_2_ – $15166_{3}^{{}^{\circ}}$
6591.278	8	1	15167.375	$13175_{4}^{{}^{\circ}}$ – 28342_5_
6591.181	100	40	15167.598	7502_3_ – $22669_{3}^{{}^{\circ}}$
6588.540	500r	200	15173.678	3865_1_ – $19039_{2}^{{}^{\circ}}$
6588.340	50	5	15174.139	5563_1_ – $20737_{1}^{{}^{\circ}}$
6588.247	1		15174.353	$14243_{1}^{{}^{\circ}}$ – 29418_2_
6588.063	2		15174.777	$14247_{0}^{{}^{\circ}}$ – 29422_1_
6586.624	4		15178.092	$14243_{1}^{{}^{\circ}}$ – 29422_1_
6585.696	100	5	15180.231	$19227_{6}^{{}^{\circ}}$ – 34407_6_
6584.615	100	8	15182.723	13297_4_ – $28480_{4}^{{}^{\circ}}$
6584.374	50	5	15183.279	$13175_{4}^{{}^{\circ}}$ – 28358_3_
6583.906	400r	200	15184.358	2869_3_ – $18053_{4}^{{}^{\circ}}$
6583.501	8	2	15185.292	18549_2_ – $33734_{2}^{{}^{\circ}}$
6583.177	10	2	15186.039	20054_2_ – $35240_{3}^{{}^{\circ}}$
6582.431	1		15187.760	21575_2_ – $36762_{3}^{{}^{\circ}}$
6581.297	4		15190.377	$20214_{3}^{{}^{\circ}}$ – 35405_3_
6581.135	1		15190.751	$19516_{2}^{{}^{\circ}}$ – 34707_3_
6580.544	3		15192.116	21645_4_ – $36837_{0}^{{}^{\circ}}$
6580.231	75	3	15192.838	11802_2_ – $26995_{3}^{{}^{\circ}}$
6579.482	20	2	15194.568	13962_1_ – $29157_{1}^{{}^{\circ}}$
6578.980	3		15195.727	$21890_{3}^{{}^{\circ}}$ – 37085_4_
6577.216	100	75	15199.803	2869_3_ – $18069_{3}^{{}^{\circ}}$
6576.123	75	8	15202.329	$14481_{6}^{{}^{\circ}}$ – 29684_5_
6575.770	2		15203.145	$18011_{0}^{{}^{\circ}}$ – 33214_5_
6575.148	3		15204.583	$19503_{3}^{{}^{\circ}}$ – 34707_3_
6574.542	4		15205.985	21594_3_ – $36800_{4}^{{}^{\circ}}$
6572.026	10	2	15211.806	$16783_{4}^{{}^{\circ}}$ – 31995_4_
6571.937	5		15212.012	15863_2_ – $31075_{2}^{{}^{\circ}}$
6568.423	3	1	15220.150	$21077_{0}^{{}^{\circ}}$ – 36297_4_
6567.213	2		15222.954	$22669_{3}^{{}^{\circ}}$ – 37892_4_
6565.068	50	5	15227.928	7280_2_ – $22508_{2}^{{}^{\circ}}$
6564.700	2		15228.782	$21902_{4}^{{}^{\circ}}$ – 37131_3_
6564.443	100	8	15229.378	$12114_{2}^{{}^{\circ}}$ – 27343_3_
6562.647	4		15233.546	$19227_{6}^{{}^{\circ}}$ – 34460_5_
6562.105	50		15234.804	13962_1_ – $29197_{1}^{{}^{\circ}}$
6561.854	3		15235.387	13962_1_ – $29197_{2}^{{}^{\circ}}$
6560.849	5		15237.720	$7795_{4}^{{}^{\circ}}$ – 23032_4_
6559.876	5	1	15239.981	$18069_{3}^{{}^{\circ}}$ – 33309_2_
6559.462	1		15240.942	$21890_{3}^{{}^{\circ}}$ – 37131_3_
6559.217	50	8	15241.512	$22163_{4}^{{}^{\circ}}$ – 37404_3_
6559.120	5h		15241.737	$21539_{4}^{{}^{\circ}}$ – 36781_5_
6558.871	100	8	15242.316	3687_2_ – $18930_{3}^{{}^{\circ}}$
6554.563	1		15252.334	$17847_{2}^{{}^{\circ}}$ – 33099_3_
6554.160	150	40	15253.272	4961_4_ – $20214_{3}^{{}^{\circ}}$
6551.917	4		15258.493	11802_2_ – $27061_{2}^{{}^{\circ}}$
6551.703	100	5	15258.992	13088_3_ – $28347_{2}^{{}^{\circ}}$
6551.265	4		15260.012	$20322_{0}^{{}^{\circ}}$ – 35582_5_
6550.335	5		15262.178	21575_2_ – $36837_{1}^{{}^{\circ}}$
6549.820	4		15263.378	$21252_{2}^{{}^{\circ}}$ – 36515_3_
6547.720	2		15268.274	18699_2_ – $33967_{3}^{{}^{\circ}}$
6547.618	1		15268.512	15493_4_ – $30761_{3}^{{}^{\circ}}$
6547.531	3		15268.714	$21539_{4}^{{}^{\circ}}$ – 36808_3_
6545.880	2		15272.566	$22248_{2}^{{}^{\circ}}$ – 37521_3_
6545.719	100	8	15272.941	17166_5_ – $32439_{4}^{{}^{\circ}}$
6545.345	1		15273.814	20054_2_ – $35328_{1}^{{}^{\circ}}$
6543.893	1		15277.203	21594_3_ – $36871_{2}^{{}^{\circ}}$
6542.511	50	3	15280.430	$17501_{0}^{{}^{\circ}}$ – 32781_4_
6542.049	50	3	15281.509	11601_1_ – $26882_{0}^{{}^{\circ}}$
6540.542	15	1	15285.030	11802_2_ – $27087_{1}^{{}^{\circ}}$
6538.301	50	4	15290.269	13962_1_ – $29252_{2}^{{}^{\circ}}$
6537.615	75	5	15291.873	13297_4_ – $28589_{3}^{{}^{\circ}}$
6537.178	50	2	15292.896	12847_3_ – $28140_{4}^{{}^{\circ}}$
6536.295	2		15294.961	15493_4_ – $30788_{0}^{{}^{\circ}}$
6535.486	4		15296.855	21575_2_ – $36871_{2}^{{}^{\circ}}$
6531.342	800r	200	15306.560	6362_2_ – $21668_{1}^{{}^{\circ}}$
6530.500	4	1	15308.534	$20522_{2}^{{}^{\circ}}$ – 35831_3_
6530.500	4	1	15308.534	$22877_{0}^{{}^{\circ}}$ – 38186_4_
6530.157	20	2	15309.338	13847_2_ – $29157_{1}^{{}^{\circ}}$
6530.125	25	3	15309.413	$21902_{4}^{{}^{\circ}}$ – 37211_5_
6529.994	25	2	15309.720	19273_2_ – $34583_{3}^{{}^{\circ}}$
6529.877	3h		15309.994	$20737_{1}^{{}^{\circ}}$ – 36047_2_
6529.597	20	2	15310.651	17959_4_ – $33270_{4}^{{}^{\circ}}$
6529.193	1		15311.598	$20322_{0}^{{}^{\circ}}$ – 35633_4_
6528.592	4		15313.008	15970_3_ – $31283_{3}^{{}^{\circ}}$
6527.858	1		15314.730	$19986_{6}^{{}^{\circ}}$ – 35300_5_
6526.584	20	2	15317.719	19273_2_ – $34591_{1}^{{}^{\circ}}$
6525.714	2		15319.761	$16217_{2}^{{}^{\circ}}$ – 31537_3_
6525.490	3		15320.287	$21165_{3}^{{}^{\circ}}$ – 36485_2_
6522.499	20	5	15327.312	$17224_{2}^{{}^{\circ}}$ – 32551_3_
6522.044	100	15	15328.381	$10783_{2}^{{}^{\circ}}$ – 26111_1_
6520.998	4		15330.840	$22855_{3}^{{}^{\circ}}$ – 38186_4_
6519.224	1		15335.012	17959_4_ – $33294_{3}^{{}^{\circ}}$
6517.277	3		15339.593	$18069_{3}^{{}^{\circ}}$ – 33408_3_
6515.875	5		15342.894	$22399_{0}^{{}^{\circ}}$ – 37742_6_
6514.699	3		15345.663	$15618_{3}^{{}^{\circ}}$ – 30964_3_
6513.200	2		15349.195	$22338_{3}^{{}^{\circ}}$ – 37688_3_
6513.052	20	1	15349.544	13847_2_ – $29197_{1}^{{}^{\circ}}$
6512.802	4		15350.133	13847_2_ – $29197_{2}^{{}^{\circ}}$
6512.366	400	15	15351.161	3687_2_ – $19039_{2}^{{}^{\circ}}$
6511.583	4		15353.007	7502_3_ – $22855_{3}^{{}^{\circ}}$
6511.050	3		15354.263	19532_4_ – $34886_{0}^{{}^{\circ}}$
6510.269	5		15356.105	17398_3_ – $32754_{3}^{{}^{\circ}}$
6509.975	1		15356.799	$21738_{2}^{{}^{\circ}}$ – 37094_2_
6509.857	3		15357.077	$19516_{2}^{{}^{\circ}}$ – 34874_3_
6509.653	2		15357.559	$18809_{4}^{{}^{\circ}}$ – 34167_3_
6509.311	3		15358.365	$22163_{4}^{{}^{\circ}}$ – 37521_3_
6509.053	150	8	15358.974	5563_1_ – $20922_{2}^{{}^{\circ}}$
6508.362	50	1	15360.605	4961_4_ – $20322_{0}^{{}^{\circ}}$
6508.292	2		15360.770	16351_0_ – $31712_{1}^{{}^{\circ}}$
6507.948	4	1	15361.582	$20214_{3}^{{}^{\circ}}$ – 35576_3_
6507.409	1		15362.854	21645_4_ – $37008_{0}^{{}^{\circ}}$
6506.989	100	15	15363.846	$10526_{3}^{{}^{\circ}}$ – 25890_2_
6505.221	1		15368.022	18431_3_ – $33799_{4}^{{}^{\circ}}$
6504.835	2		15368.933	18431_3_ – $33800_{3}^{{}^{\circ}}$
6504.458	5		15369.824	$14465_{2}^{{}^{\circ}}$ – 29835_3_
6504.226	15	2	15370.372	$17411_{3}^{{}^{\circ}}$ – 32781_4_
6501.992	100	5	15375.653	6362_2_ – $21738_{2}^{{}^{\circ}}$
6500.659	75	3	15378.806	13297_4_ – $28676_{3}^{{}^{\circ}}$
6498.769	8	1	15383.279	13297_4_ – $28680_{4}^{{}^{\circ}}$
6497.788	2		15385.601	$15166_{3}^{{}^{\circ}}$ – 30552_4_
6497.490	50	3	15386.307	$14032_{2}^{{}^{\circ}}$ – 29418_2_
6496.965	8	1	15387.550	$13175_{4}^{{}^{\circ}}$ – 28562_4_
6496.028	10	1	15389.770	7280_2_ – $22669_{3}^{{}^{\circ}}$
6495.930	5		15390.002	$14032_{2}^{{}^{\circ}}$ – 29422_1_
6495.595	3		15390.796	$18382_{0}^{{}^{\circ}}$ – 33773_1_
6495.255	20	2	15391.601	13088_3_ – $28480_{4}^{{}^{\circ}}$
6495.004	1		15392.196	$19039_{2}^{{}^{\circ}}$ – 34431_3_
6493.702	4		15395.282	21645_4_ – $37041_{4}^{{}^{\circ}}$
6493.198	150	8	15396.477	$10526_{3}^{{}^{\circ}}$ – 25923_4_
6491.222	2		15401.164	$21902_{4}^{{}^{\circ}}$ – 37303_4_
6490.736	500	50	15402.317	8800_4_ – $24202_{4}^{{}^{\circ}}$
6489.598	10	1	15405.018	13847_2_ – $29252_{2}^{{}^{\circ}}$
6489.362	2		15405.578	$18053_{4}^{{}^{\circ}}$ – 33459_4_
6488.882	50	4	15406.718	$7795_{4}^{{}^{\circ}}$ – 23201_3_
6488.882	50	4	15406.718	$18011_{0}^{{}^{\circ}}$ – 33418_4_
6488.785	2		15406.948	$14243_{1}^{{}^{\circ}}$ – 29650_2_
6487.920	3		15409.002	$22338_{3}^{{}^{\circ}}$ – 37748_2_
6487.479	50	4	15410.050	8111_4_ – $23521_{3}^{{}^{\circ}}$
6486.848	4		15411.549	18699_2_ – $34111_{1}^{{}^{\circ}}$
6484.067	15	3	15418.159	$16783_{4}^{{}^{\circ}}$ – 32202_5_
6484.067	15	3	15418.159	19713_3_ – $35131_{4}^{{}^{\circ}}$
6483.931	2		15418.482	18549_2_ – $33967_{3}^{{}^{\circ}}$
6483.741	3	1	15418.934	$20214_{3}^{{}^{\circ}}$ – 35633_4_
6483.618	20	1	15419.226	15863_2_ – $31283_{3}^{{}^{\circ}}$
6481.299	50	5	15424.743	13088_3_ – $28513_{2}^{{}^{\circ}}$
6480.444	5	1	15426.778	$17847_{2}^{{}^{\circ}}$ – 33273_1_
6479.952	4		15427.950	$16346_{4}^{{}^{\circ}}$ – 31774_3_
6478.616	8	1	15431.131	19273_2_ – $34704_{3}^{{}^{\circ}}$
6474.742	50	5	15440.364	$15490_{0}^{{}^{\circ}}$ – 30930_6_
6472.193	1		15446.445	21594_3_ – $37041_{4}^{{}^{\circ}}$
6471.936	10	1	15447.058	$16346_{4}^{{}^{\circ}}$ – 31793_4_
6467.807	3		15456.919	$19948_{4}^{{}^{\circ}}$ – 35405_3_
6467.671	50	4	15457.244	11802_2_ – $27260_{3}^{{}^{\circ}}$
6467.145	8	1	15458.502	$18069_{3}^{{}^{\circ}}$ – 33527_4_
6466.712	75	4	15459.537	8800_4_ – $24259_{4}^{{}^{\circ}}$
6466.352	50	100	15460.397	11601_1_ – $27061_{2}^{{}^{\circ}}$
6465.692	5	1	15461.976	$17847_{2}^{{}^{\circ}}$ – 33309_2_
6465.585	10	2	15462.231	$17501_{0}^{{}^{\circ}}$ – 32963_5_
6462.606	200b	100	15469.359	16554_6_ – $32023_{0}^{{}^{\circ}}$
6461.454	2		15472.117	$20322_{0}^{{}^{\circ}}$ – 35794_4_
6460.689	2		15473.949	$18053_{4}^{{}^{\circ}}$ – 33527_4_
6460.470	50	3	15474.473	8800_4_ – $24274_{0}^{{}^{\circ}}$
6459.295	5	1	15477.288	$14206_{4}^{{}^{\circ}}$ – 29684_5_
6459.116	5	1	15477.717	$20322_{0}^{{}^{\circ}}$ – 35799_6_
6458.118	2		15480.109	$22338_{3}^{{}^{\circ}}$ – 37819_4_
6457.280	800r	300	15482.118	$7795_{4}^{{}^{\circ}}$ – 23277_5_
6455.987	1		15485.219	$21645_{4}^{{}^{\circ}}$ – 37131_3_
6455.265	50	4	15486.951	11601_1_ – $27087_{1}^{{}^{\circ}}$
6454.629	4		15488.477	$21165_{3}^{{}^{\circ}}$ – 36653_2_
6453.551	3		15491.064	$22399_{0}^{{}^{\circ}}$ – 37890_5_
6453.344	5	1	15491.561	$18809_{4}^{{}^{\circ}}$ – 34301_4_
6452.951	40	2	15492.504	$19948_{4}^{{}^{\circ}}$ – 35440_3_
6452.057	25	2	15494.651	11802_2_ – $27297_{1}^{{}^{\circ}}$
6451.582	2		15495.792	$18069_{3}^{{}^{\circ}}$ – 33564_3_
6451.258	4		15496.570	$14465_{2}^{{}^{\circ}}$ – 29961_1_
6450.956	150	8	15497.295	15493_4_ – $30990_{3}^{{}^{\circ}}$
6450.697	5		15497.917	$12114_{2}^{{}^{\circ}}$ – 27612_3_
6450.593	5		15498.167	8111_4_ – $23609_{0}^{{}^{\circ}}$
6450.007	200	15	15499.575	12847_3_ – $28347_{2}^{{}^{\circ}}$
6449.518	5		15500.751	13088_3_ – $28589_{3}^{{}^{\circ}}$
6449.164	2		15501.601	17073_1_ – $32575_{2}^{{}^{\circ}}$
6448.850	3		15502.356	$21902_{4}^{{}^{\circ}}$ – 37404_3_
6446.771	100	8	15507.355	14204_5_ – $29711_{0}^{{}^{\circ}}$
6446.133	25	1	15508.890	$10414_{4}^{{}^{\circ}}$ – 25923_4_
6445.639	75	2	15510.079	$16783_{4}^{{}^{\circ}}$ – 32293_5_
6444.150	1		15513.663	21645_4_ – $37159_{3}^{{}^{\circ}}$
6444.150	1		15513.663	21645_4_ – $37159_{3}^{{}^{\circ}}$
6443.878	10	1	15514.318	$18930_{3}^{{}^{\circ}}$ – 34444_2_
6443.819	25	1	15514.460	11802_2_ – $27317_{3}^{{}^{\circ}}$
6443.090	4		15516.215	$18011_{0}^{{}^{\circ}}$ – 33527_4_
6443.090	4		15516.215	$22669_{3}^{{}^{\circ}}$ – 38186_4_
6440.353	5	1	15522.809	$15618_{3}^{{}^{\circ}}$ – 31141_3_
6440.245	1		15523.069	$20566_{4}^{{}^{\circ}}$ – 36089_4_
6439.418	4		15525.063	$22163_{4}^{{}^{\circ}}$ – 37688_3_
6438.918	50	8	15526.268	$8243_{2}^{{}^{\circ}}$ – 23769_1_
6438.303	20	2	15527.751	6362_2_ – $21890_{3}^{{}^{\circ}}$
6437.769	200	20	15529.039	14204_5_ – $29733_{0}^{{}^{\circ}}$
6437.769	200	20	15529.039	$19516_{2}^{{}^{\circ}}$ – 35046_1_
6435.108	1		15535.461	$22248_{2}^{{}^{\circ}}$ – 37784_2_
6434.800	2		15536.204	18431_3_ – $33967_{3}^{{}^{\circ}}$
6434.654	3		15536.557	18574_1_ – $34111_{1}^{{}^{\circ}}$
6431.715	5		15543.656	$17847_{2}^{{}^{\circ}}$ – 33390_1_
6431.518	2		15544.132	8111_4_ – $23655_{4}^{{}^{\circ}}$
6430.684	2		15546.148	$14465_{2}^{{}^{\circ}}$ – 30011_3_
6430.499	5	1	15546.596	$22141_{3}^{{}^{\circ}}$ – 37688_3_
6429.673	2		15548.593	19532_4_ – $35081_{0}^{{}^{\circ}}$
6428.773	40	2	15550.770	9804_5_ – $25355_{4}^{{}^{\circ}}$
6427.507	50	4	15553.832	15970_3_ – $31523_{3}^{{}^{\circ}}$
6427.507	50	4	15553.832	$22338_{3}^{{}^{\circ}}$ – 37892_4_
6427.264	3		15554.421	21594_3_ – $37149_{2}^{{}^{\circ}}$
6426.723	1		15555.730	$21252_{2}^{{}^{\circ}}$ – 36808_3_
6426.534	2		15556.187	17398_3_ – $32954_{2}^{{}^{\circ}}$
6426.151	5		15557.115	$16217_{2}^{{}^{\circ}}$ – 31774_3_
6422.948	4		15564.873	21594_3_ – $37159_{3}^{{}^{\circ}}$
6422.683	2		15565.515	$21252_{2}^{{}^{\circ}}$ – 36818_2_
6422.106	100	8	15566.913	$17501_{0}^{{}^{\circ}}$ – 33068_6_
6421.194	2		15569.124	$21738_{2}^{{}^{\circ}}$ – 37307_3_
6420.980	3		15569.643	18431_3_ – $34001_{4}^{{}^{\circ}}$
6420.225	2		15571.474	13847_2_ – $29419_{2}^{{}^{\circ}}$
6420.080	3		15571.826	$17224_{2}^{{}^{\circ}}$ – 32796_3_
6419.849	5	1	15572.386	$19516_{2}^{{}^{\circ}}$ – 35089_3_
6419.160	2		15574.057	21575_2_ – $37149_{2}^{{}^{\circ}}$
6418.697	4		15575.181	7280_2_ – $22855_{3}^{{}^{\circ}}$
6417.908	15	2	15577.096	17073_1_ – $32650_{1}^{{}^{\circ}}$
6416.930	3		15579.470	$20214_{3}^{{}^{\circ}}$ – 35794_4_
6415.536	50	5	15582.855	$16346_{4}^{{}^{\circ}}$ – 31929_3_
6414.703	15	2	15584.878	13088_3_ – $28673_{2}^{{}^{\circ}}$
6414.148	75	10	15586.227	$19503_{3}^{{}^{\circ}}$ – 35089_3_
6413.615	400	100	15587.522	13297_4_ – $28884_{3}^{{}^{\circ}}$
6412.977	1		15589.073	$22508_{2}^{{}^{\circ}}$ – 38097_2_
6412.129	2		15591.134	$15166_{3}^{{}^{\circ}}$ – 30758_2_
6411.899	500	75	15591.694	7502_3_ – $23093_{2}^{{}^{\circ}}$
6411.412	2		15592.878	$22399_{0}^{{}^{\circ}}$ – 37992_4_
6410.091	1		15596.091	$19986_{6}^{{}^{\circ}}$ – 35582_5_
6410.069	2		15596.145	$19986_{6}^{{}^{\circ}}$ – 35582_5_
6409.553	3		15597.401	7280_2_ – $22877_{1}^{{}^{\circ}}$
6408.425	2		15600.146	17959_4_ – $33560_{4}^{{}^{\circ}}$
6406.446	100	5	15604.965	4961_4_ – $20566_{4}^{{}^{\circ}}$
6403.860	4		15611.266	7502_3_ – $23113_{4}^{{}^{\circ}}$
6400.696	100	8	15618.983	0_2_ – $15618_{3}^{{}^{\circ}}$
6399.944	40		15620.819	8800_4_ – $24421_{3}^{{}^{\circ}}$
6399.571	20		15621.729	$19227_{6}^{{}^{\circ}}$ – 34849_5_
6398.602	1		15624.095	20054_2_ – $35678_{1}^{{}^{\circ}}$
6398.451	15	2	15624.463	18699_2_ – $34324_{2}^{{}^{\circ}}$
6397.447	20	2	15626.915	$15618_{3}^{{}^{\circ}}$ – 31245_2_
6396.951	200	8	15628.127	$14206_{4}^{{}^{\circ}}$ – 29835_3_
6396.518	3	1	15629.185	15970_3_ – $31599_{2}^{{}^{\circ}}$
6395.617	3	1	15631.387	$21890_{3}^{{}^{\circ}}$ – 37521_3_
6395.279	3.		15632.213	12847_3_ – $28480_{4}^{{}^{\circ}}$
6395.057	75	4	15632.756	$19516_{2}^{{}^{\circ}}$ – 35149_2_
6394.589	3	1	15633.900	$19948_{4}^{{}^{\circ}}$ – 35582_5_
6394.045	200	8	15635.230	13297_4_ – $28932_{4}^{{}^{\circ}}$
6393.201	5	1	15637.294	$18809_{4}^{{}^{\circ}}$ – 34447_4_
6392.371	75	8	15639.324	21594_3_ – $37234_{2}^{{}^{\circ}}$
6391.173	50	2	15642.256	$14206_{4}^{{}^{\circ}}$ – 29849_4_
6390.388	5	1	15644.177	$20566_{4}^{{}^{\circ}}$ – 36210_5_
6390.126	75	4	15644.819	11802_2_ – $27447_{2}^{{}^{\circ}}$
6389.391	200b	15	15646.618	$15618_{3}^{{}^{\circ}}$ – 31265_3_
6389.391	200b	15	15646.618	$19503_{3}^{{}^{\circ}}$ – 35149_2_
6388.813	100	8	15648.034	15493_4_ – $31141_{0}^{{}^{\circ}}$
6388.416	100	5	15649.006	$16346_{4}^{{}^{\circ}}$ – 31995_4_
6387.595	2		15651.018	$18809_{4}^{{}^{\circ}}$ – 34460_5_
6387.393	200	10	15651.513	3865_1_ – $19516_{2}^{{}^{\circ}}$
6387.096	8	1	15652.240	$18069_{3}^{{}^{\circ}}$ – 33721_2_
6386.780	4	1	15653.015	$21165_{3}^{{}^{\circ}}$ – 36818_2_
6385.059	20	3	15657.234	21143_5_ – $36800_{4}^{{}^{\circ}}$
6383.911	2		15660.049	15863_2_ – $31523_{3}^{{}^{\circ}}$
6381.759	25	2	15665.330	12847_3_ – $28513_{2}^{{}^{\circ}}$
6381.361	50	5	15666.307	$16346_{4}^{{}^{\circ}}$ – 32012_4_
6380.445	3		15668.556	$19039_{2}^{{}^{\circ}}$ – 34707_3_
6379.673	50	8	15670.452	$13175_{4}^{{}^{\circ}}$ – 28845_4_
6379.105	3		15671.848	$21077_{0}^{{}^{\circ}}$ – 36749_6_
6379.005	4		15672.093	$21539_{4}^{{}^{\circ}}$ – 37211_5_
6378.958	1		15672.209	18699_2_ – $34371_{2}^{{}^{\circ}}$
6378.092	1		15674.337	$16783_{4}^{{}^{\circ}}$ – 32458_4_
6376.930	500	200	15677.193	14204_5_ – $29881_{4}^{{}^{\circ}}$
6376.712	50	5	15677.729	13962_1_ – $29640_{1}^{{}^{\circ}}$
6374.269	5	1	15683.737	$18614_{1}^{{}^{\circ}}$ – 34298_1_
6372.460	50	2	15688.190	$17411_{3}^{{}^{\circ}}$ – 33099_3_
6372.125	50	8	15689.014	$11877_{1}^{{}^{\circ}}$ – 27566_2_
6371.944	150	15	15689.460	5563_1_ – $21252_{2}^{{}^{\circ}}$
6370.796	25	2	15692.287	$15736_{1}^{{}^{\circ}}$ – 31429_1_
6369.876	2		15694.554	21143_5_ – $36837_{0}^{{}^{\circ}}$
6369.141	200	25	15696.365	17166_5_ – $32862_{4}^{{}^{\circ}}$
6369.071	50b	2	15696.537	11601_1_ – $27297_{1}^{{}^{\circ}}$
6367.242	15		15701.046	15970_3_ – $31671_{4}^{{}^{\circ}}$
6367.066	3		15701.480	15493_4_ – $31194_{4}^{{}^{\circ}}$
6365.984	2		15704.149	$21077_{0}^{{}^{\circ}}$ – 36781_5_
6364.651	20	3	15707.438	$22399_{0}^{{}^{\circ}}$ – 38106_5_
6364.431	10	2	15707.981	$15618_{3}^{{}^{\circ}}$ – 31326_4_
6364.265	5	1	15708.390	19532_4_ – $35240_{3}^{{}^{\circ}}$
6362.797	2		15712.015	$16217_{2}^{{}^{\circ}}$ – 31929_3_
6362.583	2		15712.543	$19588_{0}^{{}^{\circ}}$ – 35300_5_
6362.256	100	20	15713.351	$17501_{0}^{{}^{\circ}}$ – 33214_5_
6361.796	3		15714.487	$14247_{0}^{{}^{\circ}}$ – 29961_1_
6360.981	2		15716.500	19273_2_ – $34989_{1}^{{}^{\circ}}$
6360.466	5		15717.773	$17847_{2}^{{}^{\circ}}$ – 33564_3_
6359.488	5		15720.190	$21165_{3}^{{}^{\circ}}$ – 36885_4_
6357.363	2		15725.445	$22877_{1}^{{}^{\circ}}$ – 38602_2_
6356.603	2		15727.325	$22163_{4}^{{}^{\circ}}$ – 37890_5_
6356.139	2		15728.473	16351_0_ – $32080_{1}^{{}^{\circ}}$
6355.911	800	200	15729.037	$10526_{3}^{{}^{\circ}}$ – 26255_4_
6355.633	75	8	15729.725	$11241_{3}^{{}^{\circ}}$ – 26971_4_
6355.283	10	1	15730.591	18699_2_ – $34430_{3}^{{}^{\circ}}$
6352.709	4		15736.965	0_2_ – $15736_{1}^{{}^{\circ}}$
6350.893	8	1	15741.465	19532_4_ – $35273_{0}^{{}^{\circ}}$
6348.737	200	15	15746.810	$8243_{2}^{{}^{\circ}}$ – 23990_2_
6348.213	2		15748.110	$20737_{1}^{{}^{\circ}}$ – 36485_2_
6346.963	1		15751.212	$22141_{3}^{{}^{\circ}}$ – 37892_4_
6346.119	100	8	15753.306	13297_4_ – $29050_{0}^{{}^{\circ}}$
6344.331	4		15757.746	20054_2_ – $35812_{3}^{{}^{\circ}}$
6343.728	1		15759.244	$17847_{2}^{{}^{\circ}}$ – 33606_2_
6342.860	500	150	15761.401	8800_4_ – $24561_{3}^{{}^{\circ}}$
6342.292	4	1	15762.812	$18069_{3}^{{}^{\circ}}$ – 33831_4_
6341.873	5h	1	15763.854	$21539_{4}^{{}^{\circ}}$ – 37303_4_
6340.383	5	1	15767.558	$21539_{4}^{{}^{\circ}}$ – 37307_3_
6339.663	100	15	15769.349	7280_2_ – $23049_{1}^{{}^{\circ}}$
6339.297	10	1	15770.259	9804_5_ – $25575_{4}^{{}^{\circ}}$
6337.620	100	4	15774.432	$11197_{0}^{{}^{\circ}}$ – 26971_4_
6337.528	2		15774.661	18549_2_ – $34324_{2}^{{}^{\circ}}$
6336.416	10	2	15777.429	$18930_{3}^{{}^{\circ}}$ – 34707_3_
6336.081	10	2	15778.264	$18053_{4}^{{}^{\circ}}$ – 33831_4_
6335.701	10	1	15779.210	6362_2_ – $22141_{3}^{{}^{\circ}}$
6335.012	8	2	15780.926	$15490_{0}^{{}^{\circ}}$ – 31271_5_
6333.017	1		15785.897	$21902_{4}^{{}^{\circ}}$ – 37688_3_
6332.684	8	1	15786.727	$22399_{0}^{{}^{\circ}}$ – 38186_4_
6331.415	200	15	15789.891	15493_4_ – $31283_{3}^{{}^{\circ}}$
6330.374	10	2	15792.488	13847_2_ – $29640_{1}^{{}^{\circ}}$
6328.807	50	2	15796.398	13088_3_ – $28884_{3}^{{}^{\circ}}$
6328.263	2		15797.756	$15166_{3}^{{}^{\circ}}$ – 30964_3_
6328.115	1		15798.126	$21890_{3}^{{}^{\circ}}$ – 37688_3_
6327.279	500	100	15800.213	$11197_{0}^{{}^{\circ}}$ – 26997_6_
6326.365	200	20	15802.496	$11241_{3}^{{}^{\circ}}$ – 27044_3_
6326.178	8	2	15802.963	$14032_{2}^{{}^{\circ}}$ – 29835_3_
6325.590	81	1	15804.432	$14206_{4}^{{}^{\circ}}$ – 30011_3_
6325.003	5		15805.898	8111_4_ – $23916_{4}^{{}^{\circ}}$
6324.662	2		15806.751	$22338_{3}^{{}^{\circ}}$ – 38145_3_
6324.395	40	2	15807.418	$14206_{4}^{{}^{\circ}}$ – 30014_4_
6324.104	3		15808.145	$21077_{0}^{{}^{\circ}}$ – 36885_4_
6323.055	2		15810.768	15970_3_ – $31780_{3}^{{}^{\circ}}$
6322.866	20	2	15811.240	$20322_{0}^{{}^{\circ}}$ – 36133_5_
6321.965	2		15813.494	$20522_{2}^{{}^{\circ}}$ – 36336_3_
6321.820	75	5	15813.856	7280_2_ – $23093_{2}^{{}^{\circ}}$
6321.298	50	2	15815.162	3687_2_ – $19503_{3}^{{}^{\circ}}$
6319.753	2		15819.029	19532_4_ – $35351_{4}^{{}^{\circ}}$
6319.261	20	3	15820.260	19713_3_ – $35533_{2}^{{}^{\circ}}$
6319.163	40	5	15820.506	$18011_{0}^{{}^{\circ}}$ – 33831_4_
6318.693	8	1	15821.682	$14465_{2}^{{}^{\circ}}$ – 30286_1_
6318.399	1		15822.419	18549_2_ – $34371_{2}^{{}^{\circ}}$
6318.299	8	1	15822.669	$22877_{0}^{{}^{\circ}}$ – 38700_4_
6317.183	150	8	15825.464	12847_3_ – $28673_{2}^{{}^{\circ}}$
6316.061	5	1	15828.276	12847_3_ – $28676_{3}^{{}^{\circ}}$
6315.774	100	8	15828.995	3687_2_ – $19516_{2}^{{}^{\circ}}$
6315.271	2		15830.256	$18614_{1}^{{}^{\circ}}$ – 34444_2_
6314.440	2		15832.339	$20214_{3}^{{}^{\circ}}$ – 36047_2_
6314.269	8	2	15832.768	12847_3_ – $28680_{4}^{{}^{\circ}}$
6312.622	50	4	15836.898	$15490_{0}^{{}^{\circ}}$ – 31326_4_
6311.945	8	2	15838.597	13847_2_ – $29686_{3}^{{}^{\circ}}$
6311.453	10	1	15839.832	17959_4_ – $33799_{4}^{{}^{\circ}}$
6311.097	5	1	15840.725	17959_4_ – $33800_{3}^{{}^{\circ}}$
6310.808	150	8	15841.451	$10414_{4}^{{}^{\circ}}$ – 26255_4_
6309.756	4		15844.092	13088_3_ – $28932_{4}^{{}^{\circ}}$
6308.710	20	2	15846.719	11601_1_ – $27447_{2}^{{}^{\circ}}$
6307.868	25	3	15848.834	15863_2_ – $31712_{1}^{{}^{\circ}}$
6305.276	50	4	15855.349	$16346_{4}^{{}^{\circ}}$ – 32202_5_
6303.251	200	20	15860.443	13297_4_ – $29157_{3}^{{}^{\circ}}$
6301.421	200	20	15865.049	$23655_{4}^{{}^{\circ}}$ – 39520_4_
6300.917	150	10	15866.318	$12114_{2}^{{}^{\circ}}$ – 27980_3_
6298.903	50	5	15871.391	11802_2_ – $27674_{2}^{{}^{\circ}}$
6297.777	10	1	15874.229	$17847_{2}^{{}^{\circ}}$ – 33721_2_
6297.581	1		15874.723	$20214_{3}^{{}^{\circ}}$ – 36089_4_
6297.426	3		15875.113	$17224_{2}^{{}^{\circ}}$ – 33099_3_
6296.426	1		15877.634	22401_1_ – $38278_{1}^{{}^{\circ}}$
6295.170	10	1	15880.802	17073_1_ – $32954_{2}^{{}^{\circ}}$
6295.170	10	1	15880.802	18549_2_ – $34430_{3}^{{}^{\circ}}$
6294.340	2		15882.896	$19948_{4}^{{}^{\circ}}$ – 35831_3_
6294.283	2		15883.040	$22877_{0}^{{}^{\circ}}$ – 38760_4_
6293.480	5		15885.067	17959_4_ – $33844_{0}^{{}^{\circ}}$
6293.241	150	15	15885.670	9804_5_ – $25690_{0}^{{}^{\circ}}$
6293.036	3		15886.188	$20922_{2}^{{}^{\circ}}$ – 36808_3_
6292.891	100	8	15886.554	6362_2_ – $22248_{2}^{{}^{\circ}}$
6292.187	4		15888.331	$19516_{2}^{{}^{\circ}}$ – 35405_3_
6292.061	50	3	15888.649	$14243_{1}^{{}^{\circ}}$ – 30132_2_
6291.629	50	15	15889.740	$13945_{3}^{{}^{\circ}}$ – 29835_3_
6291.192	150	15	15890.844	$14481_{6}^{{}^{\circ}}$ – 30372_6_
6290.576	2		15892.400	18431_3_ – $34324_{2}^{{}^{\circ}}$
6289.159	2		15895.981	$20922_{2}^{{}^{\circ}}$ – 36818_2_
6288.852	3	1	15896.757	13847_2_ – $29744_{3}^{{}^{\circ}}$
6288.424	8	1	15897.839	$17411_{3}^{{}^{\circ}}$ – 33309_2_
6288.074	2		15898.724	17398_3_ – $33297_{2}^{{}^{\circ}}$
6287.570	50	3	15899.998	15970_3_ – $31870_{2}^{{}^{\circ}}$
6287.254	75	4	15900.797	8800_4_ – $24701_{0}^{{}^{\circ}}$
6286.712	3		15902.168	$19503_{3}^{{}^{\circ}}$ – 35405_3_
6286.043	50	4	15903.860	$13945_{3}^{{}^{\circ}}$ – 29849_4_
6285.453	1		15905.353	$22855_{3}^{{}^{\circ}}$ – 38760_4_
6282.449	2		15912.958	18431_3_ – $34344_{4}^{{}^{\circ}}$
6281.543	5		15915.253	$18382_{0}^{{}^{\circ}}$ – 34298_1_
6280.875	2b		15916.946	$17501_{0}^{{}^{\circ}}$ – 33418_4_
6280.359	2		15918.254	$15618_{3}^{{}^{\circ}}$ – 31537_3_
6279.982	75	4	15919.209	$17354_{1}^{{}^{\circ}}$ – 33273_1_
6279.369	2		15920.763	$21165_{3}^{{}^{\circ}}$ – 37085_4_
6278.125	4		15923.918	$19516_{2}^{{}^{\circ}}$ – 35440_3_
6277.512	2		15925.473	21645_4_ – $37571_{0}^{{}^{\circ}}$
6276.162	100	8	15928.899	$13175_{4}^{{}^{\circ}}$ – 29104_4_
6275.849	3		15929.693	$14032_{2}^{{}^{\circ}}$ – 29961_1_
6275.679	2		15930.125	$22877_{1}^{{}^{\circ}}$ – 38807_2_
6274.523	2		15933.059	$22669_{3}^{{}^{\circ}}$ – 38602_2_
6272.676	3		15937.751	$19503_{3}^{{}^{\circ}}$ – 35440_3_
6271.733	1		15940.147	18431_3_ – $34371_{2}^{{}^{\circ}}$
6271.544	75	5	15940.628	2869_3_ – $18809_{4}^{{}^{\circ}}$
6271.131	2		15941.678	$23015_{0}^{{}^{\circ}}$ – 38956_4_
6270.331	8	2	15943.711	$22163_{4}^{{}^{\circ}}$ – 38106_5_
6268.923	2		15947.292	$16346_{4}^{{}^{\circ}}$ – 32293_5_
6268.200	20	2	15949.132	9804_5_ – $25753_{0}^{{}^{\circ}}$
6267.800	2		15950.150	$21738_{2}^{{}^{\circ}}$ – 37688_3_
6267.188	2		15951.707	3865_1_ – $19817_{1}^{{}^{\circ}}$
6266.614	75	5	15953.168	$16783_{4}^{{}^{\circ}}$ – 32737_5_
6265.962	3		15954.828	22098_4_ – $38053_{3}^{{}^{\circ}}$
6265.604	75	10	15955.740	14204_5_ – $30160_{4}^{{}^{\circ}}$
6264.715	100	8	15958.004	$17501_{0}^{{}^{\circ}}$ – 33459_4_
6262.884	3		15962.669	$20522_{2}^{{}^{\circ}}$ – 36485_2_
6261.584	2h		15965.983	$21165_{3}^{{}^{\circ}}$ – 37131_3_
6261.418	400	150	15966.407	$13175_{4}^{{}^{\circ}}$ – 29141_5_
6261.418	400	150	15966.407	$10414_{4}^{{}^{\circ}}$ – 26380_5_
6260.218	8	1	15969.467	8800_4_ – $24769_{3}^{{}^{\circ}}$
6258.097	2		15974.880	$15166_{3}^{{}^{\circ}}$ – 31141_3_
6257.424	200	8	15976.598	6362_2_ – $22338_{3}^{{}^{\circ}}$
6255.531	5	1	15981.432	11802_2_ – $27784_{2}^{{}^{\circ}}$
6255.344	4	1	15981.910	$21539_{4}^{{}^{\circ}}$ – 37521_3_
6254.774	20	2	15983.367	15970_3_ – $31953_{4}^{{}^{\circ}}$
6252.887	4	1	15988.190	$21902_{4}^{{}^{\circ}}$ – 37890_5_
6252.314	8	1	15989.655	$19227_{6}^{{}^{\circ}}$ – 35216_5_
6251.964	8	2	15990.550	$21902_{4}^{{}^{\circ}}$ – 37892_4_
6250.484	50	4	15994.337	$11197_{0}^{{}^{\circ}}$ – 27191_5_
6249.270	3		15997.444	$17411_{3}^{{}^{\circ}}$ – 33408_3_
6249.146	5	1	15997.761	$16783_{4}^{{}^{\circ}}$ – 32781_4_
6247.222	3		16002.688	$21890_{3}^{{}^{\circ}}$ – 37892_4_
6246.664	1		16004.117	$22141_{3}^{{}^{\circ}}$ – 38145_3_
6246.400	4		16004.794	18699_2_ – $34704_{3}^{{}^{\circ}}$
6246.173	75	8	16005.376	13847_2_ – $29853_{2}^{{}^{\circ}}$
6245.848	5	1	16006.208	15863_2_ – $31870_{2}^{{}^{\circ}}$
6245.601	50	15	16006.841	$19039_{2}^{{}^{\circ}}$ – 35046_1_
6245.149	3		16008.000	17959_4_ – $33967_{3}^{{}^{\circ}}$
6244.875	2		16008.702	$21077_{0}^{{}^{\circ}}$ – 37085_4_
6244.381	8h	2	16009.969	$21738_{2}^{{}^{\circ}}$ – 37748_2_
6243.953	2		16011.066	21594_3_ – $37605_{4}^{{}^{\circ}}$
6243.358	50	4	16012.592	13297_4_ – $29310_{4}^{{}^{\circ}}$
6241.873	10	3	16016.402	18574_1_ – $34591_{1}^{{}^{\circ}}$
6240.949	150	8	16018.773	7502_3_ – $23521_{3}^{{}^{\circ}}$
6240.305	15	2	16020.426	19713_3_ – $35733_{4}^{{}^{\circ}}$
6239.742	2		16021.871	$22508_{2}^{{}^{\circ}}$ – 38529_3_
6239.306	5	1	16022.991	$22163_{4}^{{}^{\circ}}$ – 38186_4_
6238.208	5		16025.811	18699_2_ – $34725_{3}^{{}^{\circ}}$
6238.080	20	2	16026.140	$14481_{6}^{{}^{\circ}}$ – 30508_5_
6237.982	5	1	16026.392	$17501_{0}^{{}^{\circ}}$ – 33527_4_
6237.101	5	1	16028.655	17398_3_ – $33427_{2}^{{}^{\circ}}$
6235.176	1		16033.604	18549_2_ – $34583_{3}^{{}^{\circ}}$
6234.856	400	75	16034.427	6362_2_ – $22396_{1}^{{}^{\circ}}$
6234.200	150	10	16036.114	$17354_{1}^{{}^{\circ}}$ – 33390_1_
6233.858	3		16036.994	12847_3_ – $28884_{3}^{{}^{\circ}}$
6233.004	8		16039.191	$23655_{4}^{{}^{\circ}}$ – 39694_5_
6232.845	2		16039.600	$14247_{0}^{{}^{\circ}}$ – 30286_1_
6232.186	8	1	16041.296	21575_2_ – $37616_{1}^{{}^{\circ}}$
6232.129	25	1	16041.443	17959_4_ – $34001_{4}^{{}^{\circ}}$
6232.068	50	4	16041.600	18549_2_ – $34591_{1}^{{}^{\circ}}$
6230.933	3		16044.522	$22141_{3}^{{}^{\circ}}$ – 38186_4_
6230.548	1		16045.514	$19588_{0}^{{}^{\circ}}$ – 35633_4_
6230.221	1		16046.356	$21738_{2}^{{}^{\circ}}$ – 37784_2_
6227.179	8		16054.194	14226_0_ – $30281_{1}^{{}^{\circ}}$
6226.370	100	8	16056.280	2558_0_ – $18614_{1}^{{}^{\circ}}$
6225.105	5	1	16059.543	$19516_{2}^{{}^{\circ}}$ – 35576_3_
6224.678	8	1	16060.645	$19817_{1}^{{}^{\circ}}$ – 35877_2_
6224.527	400	40	16061.034	2869_3_ – $18930_{3}^{{}^{\circ}}$
6224.043	5		16062.283	21594_3_ – $37656_{3}^{{}^{\circ}}$
6223.308	5		16064.180	$18809_{4}^{{}^{\circ}}$ – 34874_3_
6222.769	4h	1	16065.572	$16346_{4}^{{}^{\circ}}$ – 32412_4_
6222.588	3		16066.039	$13945_{3}^{{}^{\circ}}$ – 30011_3_
6221.426	5		16069.040	$13945_{3}^{{}^{\circ}}$ – 30014_4_
6221.320	100	15	16069.313	13088_3_ – $29157_{3}^{{}^{\circ}}$
6221.059	75	3	16069.988	12847_3_ – $28917_{2}^{{}^{\circ}}$
6220.011	100	5	16072.695	9804_5_ – $25877_{4}^{{}^{\circ}}$
6219.775	50	3	16073.305	11601_1_ – $27674_{2}^{{}^{\circ}}$
6219.672	1		16073.571	$19227_{6}^{{}^{\circ}}$ – 35300_5_
6218.862	2		16075.665	$22877_{1}^{{}^{\circ}}$ – 38953_5_
6217.572	10	1	16079.000	$15166_{3}^{{}^{\circ}}$ – 31245_2_
6217.437	1		16079.349	14465_5_ – 30544_2_
6217.437	1		16079.349	$22877_{1}^{{}^{\circ}}$ – 38956_4_
6216.865	15	2	16080.829	$20737_{1}^{{}^{\circ}}$ – 36818_2_
6216.441	4	1	16081.925	21575_2_ – $37656_{3}^{{}^{\circ}}$
6216.270	3		16082.368	$20214_{3}^{{}^{\circ}}$ – 36297_4_
6215.360	1		16084.722	$17224_{2}^{{}^{\circ}}$ – 33309_2_
6214.106	5	1	16087.968	17073_1_ – $33161_{1}^{{}^{\circ}}$
6213.328	3		16089.983	$21902_{4}^{{}^{\circ}}$ – 37992_4_
6212.721	100	5	16091.555	8111_4_ – $24202_{4}^{{}^{\circ}}$
6209.949	8	1	16098.738	$15166_{3}^{{}^{\circ}}$ – 31265_3_
6209.659	2		16099.489	19713_3_ – $35812_{3}^{{}^{\circ}}$
6208.986	8	1	16101.234	7502_3_ – $23603_{2}^{{}^{\circ}}$
6208.861	2		16101.559	$22855_{3}^{{}^{\circ}}$ – 38956_4_
6208.686	100	5	16102.013	$11241_{3}^{{}^{\circ}}$ – 27343_3_
6207.751	150	8	16104.438	17166_5_ – $33270_{4}^{{}^{\circ}}$
6207.220	500	200	16105.815	5563_1_ – $21668_{1}^{{}^{\circ}}$
6205.861	100	8	16109.342	13088_3_ – $29197_{2}^{{}^{\circ}}$
6205.018	50	2	16111.531	$16346_{4}^{{}^{\circ}}$ – 32458_4_
6203.493	500	100	16115.492	4961_4_ – $21077_{0}^{{}^{\circ}}$
6203.169	3	1	16116.333	$17411_{3}^{{}^{\circ}}$ – 33527_4_
6198.223	400	75	16129.193	3687_2_ – $19817_{1}^{{}^{\circ}}$
6197.591	1		16130.838	$20522_{2}^{{}^{\circ}}$ – 36653_2_
6196.311	5		16134.171	20054_2_ – $36188_{2}^{{}^{\circ}}$
6196.172	4		16134.532	$21077_{0}^{{}^{\circ}}$ – 37211_5_
6195.322	25	3	16136.746	$15490_{0}^{{}^{\circ}}$ – 31626_5_
6194.928	1		16137.772	$22669_{3}^{{}^{\circ}}$ – 38807_2_
6193.576	2		16141.295	$19948_{4}^{{}^{\circ}}$ – 36089_4_
6193.290	2		16142.040	$21165_{3}^{{}^{\circ}}$ – 37307_3_
6193.194	5		16142.291	21594_3_ – $37736_{2}^{{}^{\circ}}$
6191.906	400	50	16145.648	6362_2_ – $22508_{2}^{{}^{\circ}}$
6191.261	2		16147.330	$19986_{6}^{{}^{\circ}}$ – 36133_5_
6190.771	10	2	16148.609	$21539_{4}^{{}^{\circ}}$ – 37688_3_
6190.710	10		16148.768	8111_4_ – $24259_{4}^{{}^{\circ}}$
6189.145	50	2	16152.851	7502_3_ – $23655_{4}^{{}^{\circ}}$
6188.863	1		16153.587	$17411_{3}^{{}^{\circ}}$ – 33564_3_
6188.126	200	75	16155.511	$8243_{2}^{{}^{\circ}}$ – 24399_3_
6186.763	2		16159.070	$18930_{3}^{{}^{\circ}}$ – 35089_3_
6186.380	8	1	16160.070	$15166_{3}^{{}^{\circ}}$ – 31326_4_
6185.774	25	2	16161.654	17398_3_ – $33560_{4}^{{}^{\circ}}$
6185.683	1		16161.891	21575_2_ – $37736_{2}^{{}^{\circ}}$
6184.985	4		16163.715	8111_4_ – $24274_{0}^{{}^{\circ}}$
6184.985	4		16163.715	8111_4_ – $24274_{0}^{{}^{\circ}}$
6184.788	150	15	16164.230	13088_3_ – $29252_{2}^{{}^{\circ}}$
6183.942	5		16166.441	$17224_{2}^{{}^{\circ}}$ – 33390_1_
6182.830	50b		16169.349	13847_2_ – $30017_{3}^{{}^{\circ}}$
6182.623	500	1	16169.890	2869_3_ – $19039_{2}^{{}^{\circ}}$
6181.537	10	1	16172.731	$20922_{2}^{{}^{\circ}}$ – 37094_2_
6180.703	200	8	16174.913	5563_1_ – $21738_{2}^{{}^{\circ}}$
6180.285	1		16176.007	18549_2_ – $34725_{3}^{{}^{\circ}}$
6179.548	4		16177.937	15493_4_ – $31671_{4}^{{}^{\circ}}$
6178.930	15	2	16179.555	$16783_{4}^{{}^{\circ}}$ – 32963_5_
6178.432	200	20	16180.859	8800_4_ – $24981_{3}^{{}^{\circ}}$
6177.489	1		16183.329	11601_1_ – $27784_{2}^{{}^{\circ}}$
6177.081	1		16184.398	$17224_{2}^{{}^{\circ}}$ – 33408_3_
6176.490	4		16185.946	$14465_{2}^{{}^{\circ}}$ – 30651_3_
6175.956	10	1	16187.346	$13945_{3}^{{}^{\circ}}$ – 30132_2_
6174.797	4		16190.384	15970_3_ – $32160_{2}^{{}^{\circ}}$
6173.881	4		16192.786	17398_3_ – $33591_{3}^{{}^{\circ}}$
6173.004	2		16195.087	$17411_{3}^{{}^{\circ}}$ – 33606_2_
6170.733	15	2	16201.047	19532_4_ – $35733_{4}^{{}^{\circ}}$
6170.652	15	2	16201.260	7280_2_ – $23481_{1}^{{}^{\circ}}$
6169.822	500	200	16203.439	4961_4_ – $21165_{3}^{{}^{\circ}}$
6169.394	1		16204.563	$21902_{4}^{{}^{\circ}}$ – 38106_5_
6168.837	4	1	16206.026	$19588_{0}^{{}^{\circ}}$ – 35794_4_
6168.482	2		16206.959	$21890_{3}^{{}^{\circ}}$ – 38097_2_
6166.709	4	1	16211.619	$19588_{0}^{{}^{\circ}}$ – 35799_6_
6165.538	4	1	16214.697	$20566_{4}^{{}^{\circ}}$ – 36781_5_
6164.842	8	1	16216.528	15863_2_ – $32080_{1}^{{}^{\circ}}$
6164.480	200	20	16217.480	0_2_ – $16217_{2}^{{}^{\circ}}$
6163.732	100	5	16219.448	$18930_{3}^{{}^{\circ}}$ – 35149_2_
6162.968	40	3	16221.459	13088_3_ – $29310_{4}^{{}^{\circ}}$
6162.854	8	1	16221.759	11802_2_ – $28024_{1}^{{}^{\circ}}$
6162.456	4		16222.807	17959_4_ – $34182_{0}^{{}^{\circ}}$
6162.261	2		16223.320	17073_1_ – $33297_{2}^{{}^{\circ}}$
6161.765	50	4	16224.626	$19986_{6}^{{}^{\circ}}$ – 36210_5_
6161.646	1		16224.939	$22141_{3}^{{}^{\circ}}$ – 38366_4_
6158.829	1		16232.361	$18069_{3}^{{}^{\circ}}$ – 34301_4_
6157.093	100	8	16236.937	$7795_{4}^{{}^{\circ}}$ – 24032_4_
6156.114	8	1	16239.519	$21165_{3}^{{}^{\circ}}$ – 37404_3_
6155.587	200	15	16240.910	7280_2_ – $23521_{3}^{{}^{\circ}}$
6155.299	8	1	16241.670	$20566_{4}^{{}^{\circ}}$ – 36808_3_
6154.627	4	1	16243.443	$21902_{4}^{{}^{\circ}}$ – 38145_3_
6154.515	40	8	16243.738	9804_5_ – $26048_{4}^{{}^{\circ}}$
6154.419	3		16243.992	$12114_{2}^{{}^{\circ}}$ – 28358_3_
6154.068	100	15	16244.918	$14481_{6}^{{}^{\circ}}$ – 30726_7_
6152.950	2		16247.870	$18053_{4}^{{}^{\circ}}$ – 34301_4_
6151.993	150r		16250.398	7502_3_ – $23752_{2}^{{}^{\circ}}$
6151.517	8	2	16251.655	$17354_{1}^{{}^{\circ}}$ – 33606_2_
6151.142	1		16252.646	$20522_{2}^{{}^{\circ}}$ – 36775_3_
6150.683	20	4	16253.859	$11241_{3}^{{}^{\circ}}$ – 27495_4_
6150.323	4	1	16254.810	$14032_{2}^{{}^{\circ}}$ – 30286_1_
6150.120	2		16255.347	$21077_{0}^{{}^{\circ}}$ – 37332_6_
6150.031	1		16255.582	$21890_{3}^{{}^{\circ}}$ – 38145_3_
6148.361	3	1	16259.997	19273_2_ – $35533_{2}^{{}^{\circ}}$
6147.959	2		16261.060	$10783_{2}^{{}^{\circ}}$ – 27044_3_
6145.128	2		16268.551	22401_1_ – $38669_{2}^{{}^{\circ}}$
6145.000	2		16268.890	$21252_{2}^{{}^{\circ}}$ – 37521_3_
6144.760	20	3	16269.526	$10526_{3}^{{}^{\circ}}$ – 26796_3_
6144.414	1		16270.442	$20214_{3}^{{}^{\circ}}$ – 36485_2_
6141.928	1		16277.027	18574_1_ – $34851_{2}^{{}^{\circ}}$
6141.629	2		16277.820	$17411_{3}^{{}^{\circ}}$ – 33689_2_
6140.994	1		16279.503	$18809_{4}^{{}^{\circ}}$ – 35089_3_
6140.994	1		16279.503	$21539_{4}^{{}^{\circ}}$ – 37819_4_
6140.884	4		16279.795	$11241_{3}^{{}^{\circ}}$ – 27521_4_
6140.772	25	4	16280.092	19532_4_ – $35812_{3}^{{}^{\circ}}$
6140.449	1		16280.948	$22248_{2}^{{}^{\circ}}$ – 38529_3_
6139.364	2		16283.825	$21902_{4}^{{}^{\circ}}$ – 38186_4_
6137.025	5	1	16290.031	$18011_{0}^{{}^{\circ}}$ – 34301_4_
6135.996	5	1	16292.763	$14465_{2}^{{}^{\circ}}$ – 30758_2_
6135.628	5	1	16293.740	18431_3_ – $34725_{3}^{{}^{\circ}}$
6135.014	2		16295.371	$20522_{2}^{{}^{\circ}}$ – 36818_2_
6134.563	8	1	16296.569	15863_2_ – $32160_{2}^{{}^{\circ}}$
6133.821	50	2	16298.540	$11197_{0}^{{}^{\circ}}$ – 27495_4_
6133.671	2		16298.939	16351_0_ – $32650_{1}^{{}^{\circ}}$
6133.064	75	4	16300.552	$14243_{1}^{{}^{\circ}}$ – 30544_2_
6132.874	50	4	16301.057	$14206_{4}^{{}^{\circ}}$ – 30508_5_
6132.874	50	4	16301.057	$20214_{3}^{{}^{\circ}}$ – 36515_3_
6132.426	4	1	16302.248	18549_2_ – $34851_{2}^{{}^{\circ}}$
6131.753	3		16304.037	18574_1_ – $34878_{1}^{{}^{\circ}}$
6131.673	5	1	16304.250	$15736_{1}^{{}^{\circ}}$ – 32041_2_
6130.765	25	2	16306.665	20054_2_ – $36361_{1}^{{}^{\circ}}$
6130.459	20	1	16307.479	6362_2_ – $22669_{3}^{{}^{\circ}}$
6129.551	75	8	16309.894	12847_3_ – $29157_{3}^{{}^{\circ}}$
6129.492	15	2	16310.051	8111_4_ – $24421_{3}^{{}^{\circ}}$
6129.322	2		16310.504	$15618_{3}^{{}^{\circ}}$ – 31929_3_
6128.448	25	2	16312.830	14204_5_ – $30517_{0}^{{}^{\circ}}$
6128.344	40	3	16313.107	14204_5_ – $30517_{4}^{{}^{\circ}}$
6128.160	10	2	16313.596	16351_0_ – $32665_{1}^{{}^{\circ}}$
6127.899	3		16314.291	$19516_{2}^{{}^{\circ}}$ – 35831_3_
6127.609	50	2	16315.063	15970_3_ – $32285_{3}^{{}^{\circ}}$
6127.425	50	2	16315.553	$16783_{4}^{{}^{\circ}}$ – 33099_3_
6126.509	1		16317.993	22098_4_ – $38416_{3}^{{}^{\circ}}$
6126.328	2		16318.475	13962_1_ – $30281_{1}^{{}^{\circ}}$
6126.328	2		16318.475	13962_1_ – $30281_{1}^{{}^{\circ}}$
6125.610	4		16320.387	$17847_{2}^{{}^{\circ}}$ – 34167_3_
6124.485	200	15	16323.385	7280_2_ – $23603_{2}^{{}^{\circ}}$
6124.073	50	3	16324.483	$11197_{0}^{{}^{\circ}}$ – 27521_4_
6123.838	75	8	16325.110	$11241_{3}^{{}^{\circ}}$ – 27566_2_
6122.709	3		16328.120	$19503_{3}^{{}^{\circ}}$ – 35831_3_
6121.757	2		16330.659	13088_3_ – $29419_{2}^{{}^{\circ}}$
6120.801	50	4	16333.210	15863_2_ – $32197_{3}^{{}^{\circ}}$
6120.359	8	2	16334.389	$17354_{1}^{{}^{\circ}}$ – 33689_2_
6119.649	25	2	16336.285	17398_3_ – $33734_{2}^{{}^{\circ}}$
6118.056	4	1	16340.538	$17224_{2}^{{}^{\circ}}$ – 33564_3_
6116.172	100	8	16345.572	$14206_{4}^{{}^{\circ}}$ – 30552_4_
6114.931	8	1	16348.889	$19948_{4}^{{}^{\circ}}$ – 36297_4_
6114.546	150	10	16349.918	12847_3_ – $29197_{2}^{{}^{\circ}}$
6113.314	3		16353.213	17073_1_ – $33427_{2}^{{}^{\circ}}$
6111.700	2		16357.532	$20737_{1}^{{}^{\circ}}$ – 37094_2_
6110.677	3		16360.270	21594_3_ – $37954_{3}^{{}^{\circ}}$
6110.476	2		16360.808	13847_2_ – $30208_{2}^{{}^{\circ}}$
6110.476	2		16360.808	$19516_{2}^{{}^{\circ}}$ – 35877_2_
6110.331	51	1	16361.197	$22399_{0}^{{}^{\circ}}$ – 38760_4_
6108.293	100	5	16366.655	$17354_{1}^{{}^{\circ}}$ – 33721_2_
6106.919	5	1	16370.338	$15166_{3}^{{}^{\circ}}$ – 31537_3_
6106.847	10	2	16370.531	$11241_{3}^{{}^{\circ}}$ – 27612_3_
6105.304	4	1	16374.668	$19503_{3}^{{}^{\circ}}$ – 35877_2_
6104.970	2		16375.564	$18069_{3}^{{}^{\circ}}$ – 34444_2_
6104.572	300	200	16376.631	$15618_{3}^{{}^{\circ}}$ – 31995_4_
6104.170	4	1	16377.710	$18053_{4}^{{}^{\circ}}$ – 34431_3_
6104.027	5	1	16378.094	$18069_{3}^{{}^{\circ}}$ – 34447_4_
6103.354	2		16379.899	21575_2_ – $37954_{3}^{{}^{\circ}}$
6102.595	300	50	16381.937	$10414_{4}^{{}^{\circ}}$ – 26796_3_
6101.726	100	10	16384.270	$8243_{2}^{{}^{\circ}}$ – 24627_1_
6101.554	3	1	16384.732	17959_4_ – $34344_{4}^{{}^{\circ}}$
6100.414	3	1	16387.793	$19948_{4}^{{}^{\circ}}$ – 36336_3_
6099.994	50	3	16388.922	13297_4_ – $29686_{3}^{{}^{\circ}}$
6099.458	2		16390.362	$16346_{4}^{{}^{\circ}}$ – 32737_5_
6098.126	200	25	16393.942	17166_5_ – $33560_{4}^{{}^{\circ}}$
6097.821	4	1	16394.762	$11197_{0}^{{}^{\circ}}$ – 27591_5_
6097.592	5		16395.378	$11877_{1}^{{}^{\circ}}$ – 28273_2_
6097.292	4		16396.184	$18011_{0}^{{}^{\circ}}$ – 34407_6_
6095.389	1		16401.303	17398_3_ – $33799_{4}^{{}^{\circ}}$
6095.050	20	4	16402.216	17398_3_ – $33800_{3}^{{}^{\circ}}$
6094.089	10	1	16404.802	12847_3_ – $29252_{2}^{{}^{\circ}}$
6093.813	5	1	16405.545	19273_2_ – $35678_{1}^{{}^{\circ}}$
6093.558	1		16406.232	$22877_{0}^{{}^{\circ}}$ – 39283_4_
6093.240	8	1	16407.088	$18809_{4}^{{}^{\circ}}$ – 35216_5_
6093.027	10	1	16407.661	$21738_{2}^{{}^{\circ}}$ – 38145_3_
6090.607	2		16414.181	13297_4_ – $29711_{0}^{{}^{\circ}}$
6090.451	15	2	16414.601	7502_3_ – $23916_{4}^{{}^{\circ}}$
6090.241	1		16415.167	18574_1_ – $34989_{1}^{{}^{\circ}}$
6088.828	10	2	16418.976	$17354_{1}^{{}^{\circ}}$ – 33773_1_
6088.459	4	1	16419.972	18431_3_ – $34851_{2}^{{}^{\circ}}$
6088.203	10	2	16420.662	$17411_{3}^{{}^{\circ}}$ – 33831_4_
6088.031	400	50	16421.126	$8243_{2}^{{}^{\circ}}$ – 24664_3_
6086.702	8	1	16424.711	$14465_{2}^{{}^{\circ}}$ – 30889_1_
6085.375	100	75	16428.293	$8243_{2}^{{}^{\circ}}$ – 24671_2_
6084.486	2		16430.693	$16783_{4}^{{}^{\circ}}$ – 33214_5_
6084.127	5	1	16431.663	$18614_{1}^{{}^{\circ}}$ – 35046_1_
6083.533	2		16433.267	13847_2_ – $30281_{1}^{{}^{\circ}}$
6083.242	10	2	16434.053	20054_2_ – $36488_{2}^{{}^{\circ}}$
6082.910	2h		16434.950	$16346_{4}^{{}^{\circ}}$ – 32781_4_
6082.669	4		16435.601	$21252_{2}^{{}^{\circ}}$ – 37688_3_
6082.561	10	1	16435.893	13297_4_ – $29733_{0}^{{}^{\circ}}$
6081.299	1		16439.304	22401_1_ – $38840_{1}^{{}^{\circ}}$
6080.900	5	1	16440.382	18549_2_ – $34989_{1}^{{}^{\circ}}$
6079.469	50	4	16444.252	$14206_{4}^{{}^{\circ}}$ – 30651_3_
6079.220	200	25	16444.926	$10526_{3}^{{}^{\circ}}$ – 26971_4_
6078.419	200	40	16447.093	13297_4_ – $29744_{3}^{{}^{\circ}}$
6077.870	150	25	16448.578	$14481_{6}^{{}^{\circ}}$ – 30930_6_
6077.533	4	1	16449.490	$16346_{4}^{{}^{\circ}}$ – 32796_3_
6077.533	4	1	16449.490	$18011_{0}^{{}^{\circ}}$ – 34460_5_
6077.103	100	15	16450.654	8111_4_ – $24561_{3}^{{}^{\circ}}$
6076.984	4	1	16450.977	$17847_{2}^{{}^{\circ}}$ – 34298_1_
6074.001	5		16459.056	$20322_{0}^{{}^{\circ}}$ – 36781_5_
6073.561	3		16460.248	15493_4_ – $31953_{4}^{{}^{\circ}}$
6072.894	5	1	16462.056	12847_3_ – $29310_{4}^{{}^{\circ}}$
6072.799	20	2	16462.313	21143_5_ – $37605_{4}^{{}^{\circ}}$
6072.061	3	1	16464.314	$21902_{4}^{{}^{\circ}}$ – 38366_4_
6071.981	15	2	16464.531	$20867_{7}^{{}^{\circ}}$ – 37332_6_
6071.904	3		16464.740	$17224_{2}^{{}^{\circ}}$ – 33689_2_
6071.802	1		16465.017	19713_3_ – $36178_{4}^{{}^{\circ}}$
6070.458	1		16468.662	$22338_{3}^{{}^{\circ}}$ – 38807_2_
6070.339	25	2	16468.985	15970_3_ – $32439_{4}^{{}^{\circ}}$
6069.844	20	2	16470.328	17959_4_ – $34430_{3}^{{}^{\circ}}$
6069.019	100	5	16472.567	7280_2_ – $23752_{3}^{{}^{\circ}}$
6068.136	1		16474.964	$18930_{3}^{{}^{\circ}}$ – 35405_3_
6064.289	1		16485.415	$22248_{2}^{{}^{\circ}}$ – 38734_3_
6062.846	8		16489.338	$14481_{6}^{{}^{\circ}}$ – 30971_5_
6062.225	1		16491.028	$18809_{4}^{{}^{\circ}}$ – 35300_5_
6061.535	75	3	16492.905	6362_2_ – $22855_{3}^{{}^{\circ}}$
6059.136	10	1	16499.435	$14465_{2}^{{}^{\circ}}$ – 30964_3_
6058.438	4	1	16501.336	$19588_{0}^{{}^{\circ}}$ – 36089_4_
6056.881	5		16505.578	$15490_{0}^{{}^{\circ}}$ – 31995_4_
6055.592	50	10	16509.091	$13175_{4}^{{}^{\circ}}$ – 29684_5_
6055.036	8	1	16510.607	$18930_{3}^{{}^{\circ}}$ – 35440_3_
6054.350	1		16512.478	$14032_{2}^{{}^{\circ}}$ – 30544_2_
6053.790	3		16514.005	$14243_{1}^{{}^{\circ}}$ – 30758_2_
6053.381	300	15	16515.121	6362_2_ – $22877_{1}^{{}^{\circ}}$
6052.444	8	1	16517.678	$10526_{3}^{{}^{\circ}}$ – 27044_3_
6050.981	100	4	16521.671	8800_4_ – $25321_{3}^{{}^{\circ}}$
6050.545	1		16522.862	$15490_{0}^{{}^{\circ}}$ – 32012_4_
6050.457	3		16523.102	$21165_{3}^{{}^{\circ}}$ – 37688_3_
6049.051	800	100	16526.942	3687_2_ – $20214_{3}^{{}^{\circ}}$
6047.794	10s	1	16530.377	15493_4_ – $32023_{0}^{{}^{\circ}}$
6047.273	3		16531.802	$21252_{2}^{{}^{\circ}}$ – 37784_2_
6047.159	1		16532.113	17959_4_ – $34492_{4}^{{}^{\circ}}$
6046.643	10	2	16533.524	15970_3_ – $32503_{2}^{{}^{\circ}}$
6045.246	1		16537.345	$19039_{2}^{{}^{\circ}}$ – 35576_3_
6044.554	8		16539.238	19273_2_ – $35812_{3}^{{}^{\circ}}$
6043.676	2h		16541.641	19532_4_ – $36074_{3}^{{}^{\circ}}$
6042.750	2		16544.175	$19503_{3}^{{}^{\circ}}$ – 36047_2_
6042.593	75	5	16544.605	11802_2_ – $28347_{2}^{{}^{\circ}}$
6042.401	5	1	16545.131	$19588_{0}^{{}^{\circ}}$ – 36133_5_
6040.873	5		16549.316	$17224_{2}^{{}^{\circ}}$ – 33773_1_
6039.228	15	2	16553.824	$22399_{0}^{{}^{\circ}}$ – 38953_5_
6038.681	75	5	16555.323	8800_4_ – $25355_{4}^{{}^{\circ}}$
6037.942	3g		16557.349	$10414_{4}^{{}^{\circ}}$ – 26971_4_
6037.697	400	150	16558.021	3865_1_ – $20423_{1}^{{}^{\circ}}$
6036.765	50	2	16560.578	$10783_{2}^{{}^{\circ}}$ – 27343_3_
6035.365	1		16564.419	$20566_{4}^{{}^{\circ}}$ – 37131_3_
6035.194	75	5	16564.889	$12114_{2}^{{}^{\circ}}$ – 28679_2_
6034.334	3		16567.249	$21539_{4}^{{}^{\circ}}$ – 38106_5_
6034.215	5		16567.576	$19948_{4}^{{}^{\circ}}$ – 36515_3_
6033.515	15	2	16569.498	17398_3_ – $33967_{3}^{{}^{\circ}}$
6033.416	50	5	16569.770	11802_2_ – $28372_{1}^{{}^{\circ}}$
6032.876	75	8	16571.253	12847_3_ – $29419_{2}^{{}^{\circ}}$
6032.555	2		16572.135	$20522_{2}^{{}^{\circ}}$ – 37094_2_
6032.370	50b	8	16572.643	$19227_{6}^{{}^{\circ}}$ – 35799_6_
6030.448	75s	5	16577.925	4961_4_ – $21539_{4}^{{}^{\circ}}$
6030.189	5	1	16578.637	$16217_{2}^{{}^{\circ}}$ – 32796_3_
6029.651	50	2	16580.116	9804_5_ – $26384_{4}^{{}^{\circ}}$
6029.228	50	4	16581.279	$13175_{4}^{{}^{\circ}}$ – 29756_4_
6028.631	3	1	16582.922	$21165_{3}^{{}^{\circ}}$ – 37748_2_
6026.047	50	2	16590.032	8111_4_ – $24701_{0}^{{}^{\circ}}$
6025.784	75b	5	16590.756	13962_1_ – $30553_{2}^{{}^{\circ}}$
6025.049	1		16592.780	$22141_{3}^{{}^{\circ}}$ – 38734_3_
6024.835	2		16593.370	$20214_{3}^{{}^{\circ}}$ – 36808_3_
6024.082	1		16595.444	$18809_{4}^{{}^{\circ}}$ – 35405_3_
6023.332	3b		16597.510	$17847_{2}^{{}^{\circ}}$ – 34444_2_
6023.229	100	8	16597.794	13088_3_ – $29686_{3}^{{}^{\circ}}$
6022.702	5	1	16599.246	$13945_{3}^{{}^{\circ}}$ – 30544_2_
6022.387	5	1	16600.114	19532_4_ – $36132_{0}^{{}^{\circ}}$
6021.036	200s	75	16603.839	$7795_{4}^{{}^{\circ}}$ – 24399_3_
6020.502	25	2	16605.312	15970_3_ – $32575_{2}^{{}^{\circ}}$
6019.821	5	1	16607.190	$13945_{3}^{{}^{\circ}}$ – 30552_4_
6019.642	25	3	16607.684	$15166_{3}^{{}^{\circ}}$ – 31774_3_
6019.411	1		16608.321	$20522_{2}^{{}^{\circ}}$ – 37131_3_
6016.362	50	4	16616.738	$16346_{4}^{{}^{\circ}}$ – 32963_5_
6014.307	50	2	16622.416	$19588_{0}^{{}^{\circ}}$ – 36210_5_
6014.062	75	5	16623.093	17959_4_ – $34583_{3}^{{}^{\circ}}$
6013.442	8	2	16624.807	$16783_{4}^{{}^{\circ}}$ – 33408_3_
6012.410	3	1	16627.661	$21902_{4}^{{}^{\circ}}$ – 38529_3_
6011.537	50	5	16630.075	$10414_{4}^{{}^{\circ}}$ – 27044_3_
6011.203	15	3	16630.999	$18809_{4}^{{}^{\circ}}$ – 35440_3_
6010.253	50b	8	16633.628	17166_5_ – $33799_{4}^{{}^{\circ}}$
6010.163	100	10	16633.877	2869_3_ – $19503_{3}^{{}^{\circ}}$
6010.028	40	3	16634.251	$16783_{4}^{{}^{\circ}}$ – 33418_4_
6008.052	5	1	16639.721	15863_2_ – $32503_{2}^{{}^{\circ}}$
6007.261	10	1	16641.912	21575_2_ – $38216_{3}^{{}^{\circ}}$
6007.073	300s	75	16642.433	8800_4_ – $25442_{3}^{{}^{\circ}}$
6006.294	4		16644.592	17073_1_ – $33718_{2}^{{}^{\circ}}$
6005.921	1		16645.625	19532_4_ – $36178_{4}^{{}^{\circ}}$
6005.600	10	1	16646.515	$21539_{4}^{{}^{\circ}}$ – 38186_4_
6005.174	200	15	16647.696	2869_3_ – $19516_{2}^{{}^{\circ}}$
6003.946	5		16651.101	13962_1_ – $30613_{0}^{{}^{\circ}}$
6002.198	10	1	16655.950	13088_3_ – $29744_{3}^{{}^{\circ}}$
6001.734	10	2	16657.238	3865_1_ – $20522_{2}^{{}^{\circ}}$
6001.207	300	15	16658.701	8111_4_ – $24769_{3}^{{}^{\circ}}$
6000.766	50	8	16659.925	$13175_{4}^{{}^{\circ}}$ – 29835_3_
6000.554	5	1	16660.513	21645_4_ – $38306_{4}^{{}^{\circ}}$
6000.418	8	1	16660.891	17073_1_ – $33734_{2}^{{}^{\circ}}$
5999.594	5	1	16663.179	$18382_{0}^{{}^{\circ}}$ – 35046_1_
5997.788	5		16668.197	$19817_{1}^{{}^{\circ}}$ – 36485_2_
5997.010	8	1	16670.359	$20214_{3}^{{}^{\circ}}$ – 36885_4_
5996.630	50	4	16671.415	$8243_{2}^{{}^{\circ}}$ – 24915_3_
5995.682	10		16674.051	$13175_{4}^{{}^{\circ}}$ – 29849_4_
5995.221	50	4	16675.333	$16783_{4}^{{}^{\circ}}$ – 33459_4_
5994.126	500r	100	16678.380	3865_1_ – $20543_{0}^{{}^{\circ}}$
5993.960	50	8	16678.842	17166_5_ – $33844_{0}^{{}^{\circ}}$
5993.494	40	1	16680.138	7502_3_ – $24182_{2}^{{}^{\circ}}$
5991.457	15		16685.809	5563_1_ – $22248_{2}^{{}^{\circ}}$
5991.004	400s	50	16687.071	6362_2_ – $23049_{1}^{{}^{\circ}}$
5989.445	20	2	16691.414	18549_2_ – $35240_{3}^{{}^{\circ}}$
5986.268	50	3	16700.273	7502_3_ – $24202_{4}^{{}^{\circ}}$
5985.679	20	1	16701.916	14226_0_ – $30928_{1}^{{}^{\circ}}$
5984.998	15	3	16703.816	21575_2_ – $38278_{1}^{{}^{\circ}}$
5984.390	31		16705.513	13847_2_ – $30553_{2}^{{}^{\circ}}$
5984.278	2		16705.826	$13945_{3}^{{}^{\circ}}$ – 30651_3_
5983.163	5		16708.939	$19588_{0}^{{}^{\circ}}$ – 36297_4_
5982.651	10		16710.369	11802_2_ – $28513_{2}^{{}^{\circ}}$
5982.229	5		16711.548	15863_2_ – $32575_{2}^{{}^{\circ}}$
5982.171	5		16711.710	21594_3_ – $38306_{4}^{{}^{\circ}}$
5982.095	50	5	16711.922	$15490_{0}^{{}^{\circ}}$ – 32202_5_
5981.782	1		16712.797	$21890_{3}^{{}^{\circ}}$ – 38602_2_
5981.233	2		16714.331	19713_3_ – $36427_{3}^{{}^{\circ}}$
5979.325	4		16719.664	13297_4_ – $30017_{3}^{{}^{\circ}}$
5977.087	10	2	16725.925	$14032_{2}^{{}^{\circ}}$ – 30758_2_
5975.066	500	150	16731.582	6362_2_ – $23093_{2}^{{}^{\circ}}$
5973.665	500r	150	16735.506	3687_2_ – $20423_{1}^{{}^{\circ}}$
5973.203	10	1	16736.800	$20566_{4}^{{}^{\circ}}$ – 37303_4_
5972.300	3		16739.331	21645_4_ – $38385_{0}^{{}^{\circ}}$
5971.881	3		16740.505	$20566_{4}^{{}^{\circ}}$ – $37307_{3}^{{}^{\circ}}$
5971.369	3		16741.941	$21077_{0}^{{}^{\circ}}$ – 37819_4_
5971.073	4		16742.771	19532_4_ – $36275_{0}^{{}^{\circ}}$
5970.456	50	2	16744.501	17959_4_ – $34704_{3}^{{}^{\circ}}$
5969.742	150	10	16746.504	11601_1_ – $28347_{2}^{{}^{\circ}}$
5967.515	50	2	16752.753	$16346_{4}^{{}^{\circ}}$ – 33099_3_
5966.273	5		16756.240	$17411_{3}^{{}^{\circ}}$ – 34167_3_
5965.832	40	1	16757.479	7502_3_ – $24259_{4}^{{}^{\circ}}$
5965.745	15	1	16757.723	$14206_{4}^{{}^{\circ}}$ – 30964_3_
5964.714	4h		16760.620	21594_3_ – $38355_{2}^{{}^{\circ}}$
5964.481	40	2	16761.275	13962_1_ – $30723_{1}^{{}^{\circ}}$
5964.012	15	1	16762.593	$15166_{3}^{{}^{\circ}}$ – 31929_3_
5963.930	15	1	16762.823	$19986_{6}^{{}^{\circ}}$ – 36749_6_
5963.661	10	1	16763.579	$20322_{0}^{{}^{\circ}}$ – 37085_4_
5963.308	10	1	16764.572	13088_3_ – $29853_{2}^{{}^{\circ}}$
5962.975	3		16765.508	17959_4_ – $34725_{3}^{{}^{\circ}}$
5962.773	4		16766.076	$20922_{2}^{{}^{\circ}}$ – 37688_3_
5962.579	5		16766.621	$18809_{4}^{{}^{\circ}}$ – 35576_3_
5962.060	40	3	16768.081	$12114_{2}^{{}^{\circ}}$ – 28882_2_
5961.249	4		16770.362	21645_4_ – $38416_{3}^{{}^{\circ}}$
5960.785	50		16771.668	11601_1_ – $28372_{1}^{{}^{\circ}}$
5960.529	5		16772.388	$18809_{4}^{{}^{\circ}}$ – 35582_5_
5959.681	50	3	16774.774	8800_4_ – $25575_{4}^{{}^{\circ}}$
5958.811	15	1	16777.223	$10414_{4}^{{}^{\circ}}$ – 27191_5_
5957.592	50	2	16780.656	$14465_{2}^{{}^{\circ}}$ – 31245_2_
5957.472	50	2	16780.994	$16783_{4}^{{}^{\circ}}$ – 33564_3_
5956.514	5		16783.693	$10783_{2}^{{}^{\circ}}$ – 27566_2_
5956.263	100	8	16784.400	15970_3_ – $32754_{3}^{{}^{\circ}}$
5955.566	100	5	16786.365	11802_2_ – $28589_{3}^{{}^{\circ}}$
5954.588	40	2	16789.122	$14481_{6}^{{}^{\circ}}$ – 31271_5_
5953.587	100	4	16791.945	15493_4_ – $32285_{3}^{{}^{\circ}}$
5953.255	50	2	16792.881	13088_3_ – $29881_{4}^{{}^{\circ}}$
5952.807	2		16794.145	$19503_{3}^{{}^{\circ}}$ – 36297_4_
5952.456	2		16795.135	$19986_{6}^{{}^{\circ}}$ – 36781_5_
5950.641	25	2	16800.258	$17501_{0}^{{}^{\circ}}$ – 34301_4_
5950.429	50	2	16800.856	15493_4_ – $32294_{4}^{{}^{\circ}}$
5950.148	15	2	16801.650	15863_2_ – $32665_{1}^{{}^{\circ}}$
5949.373	50	5	16803.838	$15490_{0}^{{}^{\circ}}$ – 32293_5_
5949.218	15	1	16804.276	$18069_{3}^{{}^{\circ}}$ – 34873_4_
5948.965	5		16804.991	$18069_{3}^{{}^{\circ}}$ – 34874_3_
5948.803	100	8	16805.448	7502_3_ – $24307_{2}^{{}^{\circ}}$
5948.393	15	1	16806.607	21143_5_ – $37950_{4}^{{}^{\circ}}$
5947.502	4		16809.124	18431_3_ – $35240_{3}^{{}^{\circ}}$
5947.243	1		16809.857	16351_0_ – $33161_{1}^{{}^{\circ}}$
5946.239	40	2	16812.695	$13945_{3}^{{}^{\circ}}$ – 30758_2_
5946.027	8		16813.294	$21077_{0}^{{}^{\circ}}$ – 37890_5_
5945.480	10	1	16814.841	14204_5_ – $31019_{4}^{{}^{\circ}}$
5945.334	4		16815.254	$22141_{3}^{{}^{\circ}}$ – 38956_4_
5945.191	8	1	16815.658	$21077_{0}^{{}^{\circ}}$ – 37892_4_
5944.806	15	1	16816.747	$22877_{0}^{{}^{\circ}}$ – 39694_5_
5944.648	150	40	16817.194	$10526_{3}^{{}^{\circ}}$ – 27343_3_
5943.934	1		16819.214	$19516_{2}^{{}^{\circ}}$ – 36336_3_
5943.496	25	3	16820.454	$18053_{4}^{{}^{\circ}}$ – 34874_3_
5943.470	50	4	16820.528	$12114_{2}^{{}^{\circ}}$ – 28934_3_
5942.259	8	1	16823.956	$18809_{4}^{{}^{\circ}}$ – 35633_4_
5941.568	2		16825.912	$20922_{2}^{{}^{\circ}}$ – 37748_2_
5941.188	3		16826.988	$19948_{4}^{{}^{\circ}}$ – 36775_3_
5941.188	3		16826.988	$21539_{4}^{{}^{\circ}}$ – 38366_4_
5941.126	5	1	16827.164	$21165_{3}^{{}^{\circ}}$ – 37992_4_
5940.567	50	3	16828.747	$15166_{3}^{{}^{\circ}}$ – 31995_4_
5939.374	5b		16832.127	$21902_{4}^{{}^{\circ}}$ – 38734_3_
5939.044	15		16833.063	$19503_{3}^{{}^{\circ}}$ – 36336_3_
5938.825	400	75	16833.683	5563_1_ – $22396_{1}^{{}^{\circ}}$
5938.459	150	20	16834.721	3687_2_ – $20522_{2}^{{}^{\circ}}$
5938.285	15	1	16835.214	17166_5_ – $34001_{4}^{{}^{\circ}}$
5938.041	1	3	16835.906	$22338_{3}^{{}^{\circ}}$ – 39174_3_
5937.925	50	3	16836.235	$13175_{4}^{{}^{\circ}}$ – 30011_3_
5937.665	100	2	16836.972	$11197_{0}^{{}^{\circ}}$ – 28034_5_
5937.297	25	2	16838.016	$20566_{4}^{{}^{\circ}}$ – 37404_3_
5937.166	100	10	16838.387	12847_3_ – $29686_{3}^{{}^{\circ}}$
5936.876	20	2	16839.210	$13175_{4}^{{}^{\circ}}$ – 30014_4_
5936.876	20	2	16839.210	$15618_{3}^{{}^{\circ}}$ – 32458_4_
5936.387	50	5	16840.597	9804_5_ – $26645_{0}^{{}^{\circ}}$
5934.458	50b	3	16846.071	$15166_{3}^{{}^{\circ}}$ – 32012_4_
5934.412	75b	15	16846.201	11802_2_ – $28649_{1}^{{}^{\circ}}$
5934.103	1		16847.078	$22163_{4}^{{}^{\circ}}$ – 39010_3_
5932.911	15	1	16850.463	13962_1_ – $30812_{2}^{{}^{\circ}}$
5932.426	50	2	16851.841	$14243_{1}^{{}^{\circ}}$ – 31095_1_
5932.056	4		16852.892	$22399_{0}^{{}^{\circ}}$ – 39252_5_
5930.311	10	1	16857.851	$14032_{2}^{{}^{\circ}}$ – 30889_1_
5929.934	100	20	16858.923	3865_1_ – $20724_{0}^{{}^{\circ}}$
5929.586	10	2	16859.912	$19948_{4}^{{}^{\circ}}$ – 36808_3_
5929.328	20	2	16860.646	$17847_{2}^{{}^{\circ}}$ – 34707_3_
5928.861	20	2	16861.974	$18011_{0}^{{}^{\circ}}$ – 34873_4_
5928.755	3		16862.275	$20922_{2}^{{}^{\circ}}$ – 37784_2_
5928.654	15	2	16862.563	13297_4_ – $30160_{4}^{{}^{\circ}}$
5928.123	4	1	16864.073	$18930_{3}^{{}^{\circ}}$ – 35794_4_
5926.545	4		16868.563	$22141_{3}^{{}^{\circ}}$ – 39010_3_
5926.232	150	40	16869.454	$7795_{4}^{{}^{\circ}}$ – 24664_3_
5926.001	50	4	16870.112	8111_4_ – $24981_{3}^{{}^{\circ}}$
5925.719	15	1	16870.914	$20214_{3}^{{}^{\circ}}$ – 37085_4_
5925.404	100	8	16871.811	3865_1_ – $20737_{1}^{{}^{\circ}}$
5924.519	15	2	16874.332	$15166_{3}^{{}^{\circ}}$ – 32041_2_
5924.519	15	2	16874.332	$20867_{7}^{{}^{\circ}}$ – 37742_6_
5923.919	20	1	16876.041	13847_2_ – $30723_{1}^{{}^{\circ}}$
5922.860	50	2	16879.058	7502_3_ – $24381_{2}^{{}^{\circ}}$
5921.102	4		16884.069	$21645_{4}^{{}^{\circ}}$ – 38529_3_
5920.977	15	2	16884.426	$22399_{0}^{{}^{\circ}}$ – 39283_4_
5919.224	15	2	16889.426	$20322_{0}^{{}^{\circ}}$ – 37211_5_
5918.946	100	8	16890.220	8800_4_ – $25690_{0}^{{}^{\circ}}$
5918.946	100	8	16890.220	$17411_{3}^{{}^{\circ}}$ – 34301_4_
5918.189	15	1	16892.380	15970_3_ – $32862_{4}^{{}^{\circ}}$
5917.285	10	1	16894.961	19532_4_ – $36427_{3}^{{}^{\circ}}$
5916.730	100	20	16896.545	12847_3_ – $29744_{3}^{{}^{\circ}}$
5915.168	3		16901.007	$18930_{3}^{{}^{\circ}}$ – 35831_3_
5914.716	50b	7	16902.299	7280_2_ – $24182_{2}^{{}^{\circ}}$
5913.362	50	5	16906.169	$19227_{6}^{{}^{\circ}}$ – 36133_5_
5913.286	8	2	16906.386	$17501_{0}^{{}^{\circ}}$ – 34407_6_
5911.229	50	8	16912.269	11601_1_ – $28513_{2}^{{}^{\circ}}$
5911.097	8	1	16912.647	18549_2_ – $35462_{3}^{{}^{\circ}}$
5910.640	4	1	16913.954	13847_2_ – $30761_{3}^{{}^{\circ}}$
5910.237	15	2	16915.108	$21077_{0}^{{}^{\circ}}$ – 37992_4_
5909.887	1		16916.109	$20214_{3}^{{}^{\circ}}$ – 37131_3_
5908.957	200		16918.772	7502_3_ – $24421_{3}^{{}^{\circ}}$
5906.550	3		16925.666	17398_3_ – $34324_{2}^{{}^{\circ}}$
5906.164	3		16926.773	17959_4_ – $34886_{0}^{{}^{\circ}}$
5905.571	150	20	16928.472	4961_4_ – $21890_{3}^{{}^{\circ}}$
5905.176	50	2	16929.605	$10414_{4}^{{}^{\circ}}$ – 27343_3_
5903.646	2		16933.992	$21668_{1}^{{}^{\circ}}$ – 38602_2_
5903.496	20	2	16934.422	21645_4_ – $38580_{4}^{{}^{\circ}}$
5903.337	15	1	16934.879	$14206_{4}^{{}^{\circ}}$ – 31141_3_
5902.602	50	2	16936.987	14204_5_ – $31141_{0}^{{}^{\circ}}$
5900.448	2		16943.170	$17224_{2}^{{}^{\circ}}$ – 34167_3_
5900.355	5		16943.437	$17354_{1}^{{}^{\circ}}$ – 34298_1_
5899.846	150	5	16944.899	5563_1_ – $22508_{2}^{{}^{\circ}}$
5899.521	50	4	16945.832	15493_4_ – $32439_{4}^{{}^{\circ}}$
5899.466	8b	1	16945.990	$17501_{0}^{{}^{\circ}}$ – 34447_4_
5897.246	1		16952.370	$22399_{0}^{{}^{\circ}}$ – 39351_5_
5896.786	201	1	16953.692	8800_4_ – $25753_{0}^{{}^{\circ}}$
5896.114	2		16955.624	19713_3_ – $36668_{2}^{{}^{\circ}}$
5895.283	150	25	16958.014	13297_4_ – $30255_{3}^{{}^{\circ}}$
5895.053	10	1	16958.676	18574_1_ – $35533_{2}^{{}^{\circ}}$
5894.699	50	25	16959.694	$17501_{0}^{{}^{\circ}}$ – 34460_5_
5893.191	20	2	16964.034	$14465_{2}^{{}^{\circ}}$ – 31429_1_
5892.780	8		16965.217	13847_2_ – $30812_{2}^{{}^{\circ}}$
5892.434	50	5	16966.213	13962_1_ – $30928_{1}^{{}^{\circ}}$
5891.774	5	1	16968.114	$15490_{0}^{{}^{\circ}}$ – 32458_4_
5891.670	5		16968.414	$19516_{2}^{{}^{\circ}}$ – 36485_2_
5891.451	200	25	16969.044	$10526_{3}^{{}^{\circ}}$ – 27495_4_
5887.919	8		16979.223	18699_2_ – $35678_{1}^{{}^{\circ}}$
5887.437	2		16980.613	$21165_{3}^{{}^{\circ}}$ – 38145_3_
5886.851	5		16982.304	$22855_{3}^{{}^{\circ}}$ – 39837_3_
5886.456	15		16983.443	$19227_{6}^{{}^{\circ}}$ – 36210_5_
5886.093	15	1	16984.491	15970_3_ – $32954_{2}^{{}^{\circ}}$
5885.702	200	50	16985.619	9804_5_ – $26790_{4}^{{}^{\circ}}$
5885.502	75	2	16986.196	$11241_{3}^{{}^{\circ}}$ – 28227_4_
5884.033	50	2	16990.437	14204_5_ – $31194_{4}^{{}^{\circ}}$
5882.460	10	1	16994.980	$10526_{3}^{{}^{\circ}}$ – 27521_4_
5881.988	2		16996.344	$21738_{2}^{{}^{\circ}}$ – 38734_3_
5881.065	4		16999.011	$19516_{2}^{{}^{\circ}}$ – 36515_3_
5879.127	50	2	17004.615	$11877_{1}^{{}^{\circ}}$ – 28882_2_
5878.933	100	3	17005.176	12847_3_ – $29853_{2}^{{}^{\circ}}$
5877.186	2		17010.231	$20322_{0}^{{}^{\circ}}$ – 37332_6_
5877.013	25	2	17010.732	$20737_{1}^{{}^{\circ}}$ – 37748_2_
5876.647	3		17011.791	$22163_{4}^{{}^{\circ}}$ – 39174_3_
5874.993	150	8	17016.580	17166_5_ – $34182_{0}^{{}^{\circ}}$
5874.040	50	3	17019.341	$13945_{3}^{{}^{\circ}}$ – 30964_3_
5873.920	4	1	17019.689	21645_4_ – $38665_{0}^{{}^{\circ}}$
5873.774	8	1	17020.112	$17411_{3}^{{}^{\circ}}$ – 34431_3_
5873.462	8		17021.016	$21165_{3}^{{}^{\circ}}$ – 38186_4_
5873.332	10		17021.393	$18809_{4}^{{}^{\circ}}$ – 35831_3_
5871.405	15		17026.979	$17847_{2}^{{}^{\circ}}$ – 34874_3_
5871.186	100	20	17027.614	7280_2_ – $24307_{2}^{{}^{\circ}}$
5870.054	20	1	17030.898	$11197_{0}^{{}^{\circ}}$ – 28227_4_
5869.854	100	5	17031.478	$11241_{3}^{{}^{\circ}}$ – 28273_2_
5869.200	2b		17033.376	$17411_{3}^{{}^{\circ}}$ – 34444_2_
5869.164	8		17033.480	12847_3_ – $29881_{4}^{{}^{\circ}}$
5868.382	150	25	17035.750	$18053_{4}^{{}^{\circ}}$ – 35089_3_
5867.841	20	1	17037.321	17073_1_ – $34111_{1}^{{}^{\circ}}$
5867.542	1		17038.189	$18382_{0}^{{}^{\circ}}$ – 35421_1_
5866.814	50	5	17040.303	$10526_{3}^{{}^{\circ}}$ – 27566_2_
5864.128	4		17048.108	11601_1_ – $28649_{1}^{{}^{\circ}}$
5863.720	100	8	17049.294	3687_2_ – $20737_{1}^{{}^{\circ}}$
5863.562	10		17049.754	19713_3_ – $36762_{3}^{{}^{\circ}}$
5863.146	3	1	17050.963	$21902_{4}^{{}^{\circ}}$ – 38953_5_
5861.894	2		17054.605	$21902_{4}^{{}^{\circ}}$ – 38956_4_
5861.289	8		17056.366	$16217_{2}^{{}^{\circ}}$ – 33273_1_
5861.192	15		17056.648	3865_1_ – $20922_{2}^{{}^{\circ}}$
5860.480	15	1	17058.720	$14206_{4}^{{}^{\circ}}$ – 31265_3_
5860.259	15	1	17059.363	7502_3_ – $24561_{3}^{{}^{\circ}}$
5859.348	15	1	17062.016	$16346_{4}^{{}^{\circ}}$ – 33408_3_
5858.752	50	3	17063.751	$14032_{2}^{{}^{\circ}}$ – 31095_1_
5858.641	50	2	17064.075	$14206_{4}^{{}^{\circ}}$ – 31271_5_
5857.725	2		17066.743	$21890_{3}^{{}^{\circ}}$ – 38956_4_
5856.112	15	1	17071.444	13088_3_ – $30160_{4}^{{}^{\circ}}$
5855.914	5		17072.021	$14465_{2}^{{}^{\circ}}$ – 31537_3_
5855.789	1		17072.385	11601_1_ – $28673_{2}^{{}^{\circ}}$
5854.858	3		17075.100	21594_3_ – $38669_{2}^{{}^{\circ}}$
5854.120	150	8	17077.253	8800_4_ – $25877_{4}^{{}^{\circ}}$
5853.474	100	5	17079.137	2869_3_ – $19948_{4}^{{}^{\circ}}$
5852.945	10		17080.681	$18069_{3}^{{}^{\circ}}$ – 35149_2_
5852.844	15		17080.976	13847_2_ – $30928_{1}^{{}^{\circ}}$
5852.682	200	25	17081.449	$10414_{4}^{{}^{\circ}}$ – 27495_4_
5852.487	100	8	17082.018	11802_2_ – $28884_{3}^{{}^{\circ}}$
5851.214	8	2	17085.734	$10526_{3}^{{}^{\circ}}$ – 27612_3_
5850.566	5		17087.627	19713_3_ – $36800_{4}^{{}^{\circ}}$
5850.378	10	1	17088.176	19273_2_ – $36361_{1}^{{}^{\circ}}$
5850.270	3		17088.491	$20214_{3}^{{}^{\circ}}$ – 37303_4_
5849.765	8	1	17089.966	$17354_{1}^{{}^{\circ}}$ – 34444_2_
5849.514	15	1	17090.700	15863_2_ – $32954_{2}^{{}^{\circ}}$
5849.217	10		17091.567	$16217_{2}^{{}^{\circ}}$ – 33309_2_
5848.554	25	2	17093.505	19532_4_ – $36625_{0}^{{}^{\circ}}$
5848.277	1		17094.315	20054_2_ – $37149_{2}^{{}^{\circ}}$
5848.135	4		17094.729	21575_2_ – $38669_{2}^{{}^{\circ}}$
5845.919	100	5	17101.209	7280_2_ – $24381_{2}^{{}^{\circ}}$
5844.873	5		17104.270	18574_1_ – $35678_{1}^{{}^{\circ}}$
5844.793	75	3	17104.504	20054_2_ – $37159_{1}^{{}^{\circ}}$
5844.704	3		17104.765	20054_2_ – $37159_{3}^{{}^{\circ}}$
5843.805	50	5	17107.396	$10414_{4}^{{}^{\circ}}$ – 27521_4_
5843.267	10	1	17108.971	$21077_{0}^{{}^{\circ}}$ – 38186_4_
5843.012	5		17109.718	$14032_{2}^{{}^{\circ}}$ – 31141_3_
5842.050	50	4	17112.535	$16346_{4}^{{}^{\circ}}$ – 33459_4_
5841.915	15	2	17112.930	18699_2_ – $35812_{3}^{{}^{\circ}}$
5841.758	3		17113.390	13962_1_ – $31075_{2}^{{}^{\circ}}$
5841.281	4		17114.788	$21645_{4}^{{}^{\circ}}$ – 38760_4_
5841.199	10		17115.028	11802_2_ – $28917_{2}^{{}^{\circ}}$
5840.639	50	5	17116.669	$11241_{3}^{{}^{\circ}}$ – 28358_3_
5840.535	8		17116.974	$18930_{3}^{{}^{\circ}}$ – 36047_2_
5839.850	40	2	17118.982	6362_2_ – $23481_{1}^{{}^{\circ}}$
5839.584	40	2	17119.761	$7795_{4}^{{}^{\circ}}$ – 24915_3_
5839.492	50	8	17120.031	13088_3_ – $30208_{2}^{{}^{\circ}}$
5839.266	8		17120.694	$22163_{4}^{{}^{\circ}}$ – 39283_4_
5833.849	8		17136.591	$19516_{2}^{{}^{\circ}}$ – 36653_2_
5833.132	2		17138.697	$21668_{1}^{{}^{\circ}}$ – 38807_2_
5832.371	100	15	17140.934	7280_2_ – $24421_{3}^{{}^{\circ}}$
5831.923	5	1	17142.250	$22141_{3}^{{}^{\circ}}$ – 39283_4_
5831.754	8		17142.747	13847_2_ – $30990_{3}^{{}^{\circ}}$
5831.682	5		17142.959	21645_4_ – $38788_{3}^{{}^{\circ}}$
5831.002	3	1	17144.958	$14481_{6}^{{}^{\circ}}$ – 31626_5_
5830.827	50	5	17145.472	$11197_{0}^{{}^{\circ}}$ – 28342_5_
5827.900	2		17154.083	19273_2_ – $36427_{3}^{{}^{\circ}}$
5826.344	40b	1	17158.665	6362_2_ – $23521_{3}^{{}^{\circ}}$
5826.280	50b	5	17158.853	19713_3_ – $36871_{2}^{{}^{\circ}}$
5826.097	25	2	17159.392	$18930_{3}^{{}^{\circ}}$ – 36089_4_
5825.675	8		17160.635	$19588_{0}^{{}^{\circ}}$ – 36749_6_
5825.003	40	3	17162.615	$15618_{3}^{{}^{\circ}}$ – 32781_4_
5824.895	50	5	17162.933	21143_5_ – $38306_{4}^{{}^{\circ}}$
5823.555	3		17166.882	13088_3_ – $30255_{3}^{{}^{\circ}}$
5823.261	2		17167.749	$22669_{3}^{{}^{\circ}}$ – 39837_3_
5822.796	100	8	17169.120	12847_3_ – $30017_{3}^{{}^{\circ}}$
5822.054	40	1	17171.308	17959_4_ – $35131_{4}^{{}^{\circ}}$
5821.691	3		17172.379	$22508_{2}^{{}^{\circ}}$ – 39680_2_
5820.820	10		17174.948	$20922_{2}^{{}^{\circ}}$ – 38097_2_
5820.081	25	2	17177.129	$15618_{3}^{{}^{\circ}}$ – 32796_3_
5819.902	15	1	17177.657	$10414_{4}^{{}^{\circ}}$ – 27591_5_
5819.607	50	3	17178.528	17166_5_ – $34344_{4}^{{}^{\circ}}$
5819.367	4b		17179.236	20054_2_ – $37234_{2}^{{}^{\circ}}$
5819.302	15	1	17179.428	15863_2_ – $33043_{3}^{{}^{\circ}}$
5819.130	20	1	17179.936	4961_4_ – $22141_{3}^{{}^{\circ}}$
5818.453	50	5	17181.935	$14247_{0}^{{}^{\circ}}$ – 31429_1_
5818.218	2		17182.629	$19948_{4}^{{}^{\circ}}$ – 37131_3_
5817.545	10	1	17184.617	17398_3_ – $34583_{3}^{{}^{\circ}}$
5816.188	15	1	17188.626	$22163_{4}^{{}^{\circ}}$ – 39351_5_
5815.823	2		17189.705	$20214_{3}^{{}^{\circ}}$ – 37404_3_
5814.729	50	2	17192.939	$19588_{0}^{{}^{\circ}}$ – 36781_5_
5814.101	8	2	17194.796	$21539_{4}^{{}^{\circ}}$ – 38734_3_
5813.534	1		17196.473	$13945_{3}^{{}^{\circ}}$ – 31141_3_
5813.181	8		17197.517	$10783_{2}^{{}^{\circ}}$ – 27980_3_
5812.976	100	8	17198.124	$10414_{4}^{{}^{\circ}}$ – 27612_3_
5812.709	50	2	17198.914	$17847_{2}^{{}^{\circ}}$ – 35046_1_
5811.848	40	1	17201.462	4961_4_ – $22163_{4}^{{}^{\circ}}$
5810.453	5		17205.591	$18011_{0}^{{}^{\circ}}$ – 35216_5_
5808.659	50	25	17210.905	8111_4_ – $25321_{3}^{{}^{\circ}}$
5807.932	1		17213.060	22098_4_ – $39311_{4}^{{}^{\circ}}$
5807.685	50	4	17213.792	$14032_{2}^{{}^{\circ}}$ – 31245_2_
5807.685	50	4	17213.792	21575_2_ – $38788_{3}^{{}^{\circ}}$
5806.201	40	2	17218.191	$16346_{4}^{{}^{\circ}}$ – 33564_3_
5805.702	50	3	17219.671	13297_4_ – $30517_{0}^{{}^{\circ}}$
5805.612	1		17219.938	13297_4_ – $30517_{4}^{{}^{\circ}}$
5805.495	25	1	17220.285	$17224_{2}^{{}^{\circ}}$ – 34444_2_
5805.244	2		17221.030	$21539_{4}^{{}^{\circ}}$ – 38760_4_
5804.141	500r	50	17224.302	0_2_ – $17224_{2}^{{}^{\circ}}$
5803.805	5	1	17225.299	$20522_{2}^{{}^{\circ}}$ – 37748_2_
5803.735	8	1	17225.507	$19986_{6}^{{}^{\circ}}$ – 37211_5_
5802.851	501	1	17228.131	13847_2_ – $31075_{2}^{{}^{\circ}}$
5802.097	3		17230.370	19532_4_ – $36762_{3}^{{}^{\circ}}$
5801.027	40	1	17233.548	$14032_{2}^{{}^{\circ}}$ – 31265_3_
5800.830	200	20	17234.133	3687_2_ – $20922_{2}^{{}^{\circ}}$
5800.159	1	1	17236.127	8800_4_ – $26036_{3}^{{}^{\circ}}$
5798.479	50	4	17241.121	6362_2_ – $23603_{2}^{{}^{\circ}}$
5798.260	4		17241.772	21143_5_ – $38385_{0}^{{}^{\circ}}$
5798.088	5		17242.284	$17847_{2}^{{}^{\circ}}$ – 35089_3_
5797.319	75	3	17244.571	8111_4_ – $25355_{4}^{{}^{\circ}}$
5796.521	50	1	17246.945	$15490_{0}^{{}^{\circ}}$ – 32737_5_
5796.070	200	15	17248.287	8800_4_ – $26048_{4}^{{}^{\circ}}$
5795.407	1		17250.260	17073_1_ – $34324_{2}^{{}^{\circ}}$
5792.685	4		17258.366	$19516_{2}^{{}^{\circ}}$ – 36775_3_
5792.431	150	8	17259.123	2558_0_ – $19817_{1}^{{}^{\circ}}$
5791.704	50	2	17261.289	15493_4_ – $32754_{3}^{{}^{\circ}}$
5791.558	5	1	17261.724	$20522_{2}^{{}^{\circ}}$ – 37784_2_
5791.032	5	1	17263.292	$19948_{4}^{{}^{\circ}}$ – 37211_5_
5789.644	200	15	17267.431	7502_3_ – $24769_{3}^{{}^{\circ}}$
5789.374	10	1	17268.236	19532_4_ – $36800_{4}^{{}^{\circ}}$
5788.027	4		17272.255	$19503_{3}^{{}^{\circ}}$ – 36775_3_
5787.896	3		17272.646	$21902_{4}^{{}^{\circ}}$ – 39174_3_
5786.209	3	2	17277.681	$19817_{1}^{{}^{\circ}}$ – 37094_2_
5785.122	8	1	17280.928	17959_4_ – $35240_{3}^{{}^{\circ}}$
5784.913	4		17281.552	7280_2_ – $24561_{3}^{{}^{\circ}}$
5782.307	40b	5	17289.341	$11241_{3}^{{}^{\circ}}$ – 28531_2_
5781.655	50	1	17291.290	$15166_{3}^{{}^{\circ}}$ – 32458_4_
5780.438	2		17294.931	$22399_{0}^{{}^{\circ}}$ – 39694_5_
5779.833	50	2	17296.741	8800_4_ – $26096_{3}^{{}^{\circ}}$
5779.735	8	3	17297.034	$19039_{2}^{{}^{\circ}}$ – 36336_3_
5779.407	20	2	17298.016	17073_1_ – $34371_{2}^{{}^{\circ}}$
5778.594	10	1	17300.450	15970_3_ – $33270_{4}^{{}^{\circ}}$
5778.364	1		17301.138	$19516_{2}^{{}^{\circ}}$ – 36818_2_
5777.856	10	1	17302.659	$17847_{2}^{{}^{\circ}}$ – 35149_2_
5777.398	75	8	17304.031	$12114_{2}^{{}^{\circ}}$ – 29418_2_
5777.023	2		17305.154	$19503_{3}^{{}^{\circ}}$ – 36808_3_
5776.550	2		17306.571	$20214_{3}^{{}^{\circ}}$ – 37521_3_
5776.164	25	2	17307.728	$12114_{2}^{{}^{\circ}}$ – 29422_1_
5775.615	8	1	17309.373	$14465_{2}^{{}^{\circ}}$ – 31774_3_
5775.312	3		17310.281	16351_0_ – $33662_{1}^{{}^{\circ}}$
5774.981	5	1	17311.273	18574_1_ – $35885_{2}^{{}^{\circ}}$
5774.726	10	1	17312.038	12847_3_ – $30160_{4}^{{}^{\circ}}$
5774.073	8	2	17313.995	17959_4_ – $35273_{0}^{{}^{\circ}}$
5773.946	150	10	17314.376	5563_1_ – $22877_{1}^{{}^{\circ}}$
5773.750	10	1	17314.964	$19503_{3}^{{}^{\circ}}$ – 36818_2_
5771.960	15	1	17320.334	$13945_{3}^{{}^{\circ}}$ – 31265_3_
5771.758	75	5	17320.940	$11241_{3}^{{}^{\circ}}$ – 28562_4_
5770.865	10	2	17323.620	$18809_{4}^{{}^{\circ}}$ – 36133_5_
5770.795	5	1	17323.830	$20566_{4}^{{}^{\circ}}$ – 37890_5_
5770.562	4	1	17324.530	$20423_{1}^{{}^{\circ}}$ – 37748_2_
5770.461	10	1	17324.833	15970_3_ – $33294_{3}^{{}^{\circ}}$
5770.100	15b		17325.917	17166_5_ – $34492_{4}^{{}^{\circ}}$
5769.730	8		17327.028	15970_3_ – $33297_{2}^{{}^{\circ}}$
5769.730	8		17327.028	17398_3_ – $34725_{3}^{{}^{\circ}}$
5768.633	10	1	17330.323	$14206_{4}^{{}^{\circ}}$ – 31537_3_
5768.181	200	15	17331.681	8111_4_ – $25442_{3}^{{}^{\circ}}$
5767.779	150	10	17332.889	$13175_{4}^{{}^{\circ}}$ – 30508_5_
5766.662	5		17336.246	$18069_{3}^{{}^{\circ}}$ – 35405_3_
5766.589	1		17336.466	18549_2_ – $35885_{2}^{{}^{\circ}}$
5763.529	200	10	17345.670	2869_3_ – $20214_{3}^{{}^{\circ}}$
5763.312	5	1	17346.323	$19986_{6}^{{}^{\circ}}$ – 37332_6_
5762.959	15	1	17347.386	$16217_{2}^{{}^{\circ}}$ – 33564_3_
5762.796	100	10	17347.876	$17501_{0}^{{}^{\circ}}$ – 34849_5_
5762.078	3b		17350.038	$21902_{4}^{{}^{\circ}}$ – 39252_5_
5761.527	25	2	17351.697	$18053_{4}^{{}^{\circ}}$ – 35405_3_
5760.550	600r	150	17354.640	0_2_ – $17354_{1}^{{}^{\circ}}$
5760.447	75b	15	17354.950	11802_2_ – $29157_{3}^{{}^{\circ}}$
5759.190	5	1	17358.738	$19948_{4}^{{}^{\circ}}$ – 37307_3_
5758.471	5		17360.906	$20423_{1}^{{}^{\circ}}$ – 37784_2_
5757.360	1		17364.256	$21645_{4}^{{}^{\circ}}$ – 39010_3_
5757.336	1		17364.328	$21645_{4}^{{}^{\circ}}$ – 39010_3_
5756.904	4	1	17365.631	$11197_{0}^{{}^{\circ}}$ – 28562_4_
5756.463	1		17366.961	$18930_{3}^{{}^{\circ}}$ – 36297_4_
5756.445	1		17367.016	$18930_{3}^{{}^{\circ}}$ – 36297_4_
5754.844	8	1	17371.847	$18069_{3}^{{}^{\circ}}$ – 35440_3_
5754.734	15	3	17372.179	$17501_{0}^{{}^{\circ}}$ – 34873_4_
5753.984	3		17374.444	18699_2_ – $36074_{3}^{{}^{\circ}}$
5753.026	200	25	17377.337	4961_4_ – $22338_{3}^{{}^{\circ}}$
5752.580	15	1	17378.684	6362_2_ – $23741_{1}^{{}^{\circ}}$
5751.629	4b	1	17381.557	$21902_{4}^{{}^{\circ}}$ – 39283_4_
5751.594	5b	1	17381.663	$13945_{3}^{{}^{\circ}}$ – 31326_4_
5751.432	10	2	17382.153	$19503_{3}^{{}^{\circ}}$ – 36885_4_
5749.782	50b	8	17387.141	3865_1_ – $21252_{2}^{{}^{\circ}}$
5749.733	20b	3	17387.289	$18053_{4}^{{}^{\circ}}$ – 35440_3_
5749.230	15	1	17388.810	$16217_{2}^{{}^{\circ}}$ – 33606_2_
5748.741	150	25	17390.289	6362_2_ – $23752_{2}^{{}^{\circ}}$
5748.323	100	8	17391.554	17959_4_ – $35351_{4}^{{}^{\circ}}$
5747.193	50	4	17394.973	11802_2_ – $29197_{2}^{{}^{\circ}}$
5747.058	2		17395.382	19273_2_ – $36668_{2}^{{}^{\circ}}$
5746.468	4		17397.168	$14032_{2}^{{}^{\circ}}$ – 31429_1_
5745.233	5	1	17400.908	$18809_{4}^{{}^{\circ}}$ – 36210_5_
5743.583	8	1	17405.906	$18930_{3}^{{}^{\circ}}$ – 36336_3_
5743.063	15	1	17407.483	12847_3_ – $30255_{3}^{{}^{\circ}}$
5741.829	50	10	17411.224	0_2_ – $17411_{3}^{{}^{\circ}}$
5741.715	5	1	17411.569	$23015_{0}^{{}^{\circ}}$ – 40426_4_
5740.196	2		17416.177	21645_4_ – $39062_{0}^{{}^{\circ}}$
5739.837	3	1	17417.266	$21539_{4}^{{}^{\circ}}$ – 38956_4_
5738.965	20	2	17419.912	$14206_{4}^{{}^{\circ}}$ – 31626_5_
5737.645	2		17423.920	21575_2_ – $38998_{3}^{{}^{\circ}}$
5736.031	100	8	17428.823	13088_3_ – $30517_{4}^{{}^{\circ}}$
5735.304	8	1	17431.032	15863_2_ – $33294_{3}^{{}^{\circ}}$
5735.146	1		17431.512	$22248_{2}^{{}^{\circ}}$ – 39680_2_
5734.579	8		17433.236	15863_2_ – $33297_{2}^{{}^{\circ}}$
5733.887	20	2	17435.340	13847_2_ – $31283_{3}^{{}^{\circ}}$
5733.651	4	1	17436.057	19713_3_ – $37149_{2}^{{}^{\circ}}$
5733.388	5	1	17436.857	$21738_{2}^{{}^{\circ}}$ – 39174_3_
5733.096	20	2	17437.745	4961_4_ – $22399_{0}^{{}^{\circ}}$
5730.307	2		17446.232	$19039_{2}^{{}^{\circ}}$ – 36485_2_
5729.236	2		17449.493	$21902_{4}^{{}^{\circ}}$ – 39351_5_
5729.119	5		17449.850	11802_2_ – $29252_{2}^{{}^{\circ}}$
5727.997	8		17453.268	17398_3_ – $34851_{2}^{{}^{\circ}}$
5727.711	40	3	17454.139	$10526_{3}^{{}^{\circ}}$ – 27980_3_
5727.021	50	3	17456.242	$19948_{4}^{{}^{\circ}}$ – 37404_3_
5726.792	1		17456.940	15970_3_ – $33427_{2}^{{}^{\circ}}$
5725.390	300	75	17461.215	9804_5_ – $27266_{4}^{{}^{\circ}}$
5724.863	2		17462.822	$17411_{3}^{{}^{\circ}}$ – 34874_3_
5724.463	75	4	17464.043	8111_4_ – $25575_{4}^{{}^{\circ}}$
5724.386	20	2	17464.277	13297_4_ – $30761_{3}^{{}^{\circ}}$
5724.386	20	2	17464.277	$14465_{2}^{{}^{\circ}}$ – 31929_3_
5724.240	100	10	17464.723	13088_3_ – $30553_{2}^{{}^{\circ}}$
5723.532	10	1	17466.883	14204_5_ – $31671_{4}^{{}^{\circ}}$
5722.012	2		17471.523	$16217_{2}^{{}^{\circ}}$ – 33689_2_
5721.425	50	4	17473.316	$15490_{0}^{{}^{\circ}}$ – 32963_5_
5720.185	500r	150	17477.103	3687_2_ – $21165_{3}^{{}^{\circ}}$
5719.625	200	50	17478.815	7502_3_ – $24981_{3}^{{}^{\circ}}$
5719.098	20	2	17480.425	$15618_{3}^{{}^{\circ}}$ – 33099_3_
5718.657	2		17481.773	$21252_{2}^{{}^{\circ}}$ – 38734_3_
5718.118	8	1	17483.421	$17224_{2}^{{}^{\circ}}$ – 34707_3_
5717.526	20	2	17485.231	$16346_{4}^{{}^{\circ}}$ – 33831_4_
5717.172	50	15	17486.314	5563_1_ – $23049_{1}^{{}^{\circ}}$
5716.815	10	1	17487.406	$18809_{4}^{{}^{\circ}}$ – 36297_4_
5716.106	1		17489.575	7280_2_ – $24769_{3}^{{}^{\circ}}$
5715.948	8	1	17490.058	$10783_{2}^{{}^{\circ}}$ – 28273_2_
5715.725	20	2	17490.741	13297_4_ – $30788_{0}^{{}^{\circ}}$
5713.732	2		17496.842	$20322_{0}^{{}^{\circ}}$ – 37819_4_
5711.998	50	5	17502.153	17959_4_ – $35462_{3}^{{}^{\circ}}$
5711.454	1		17503.820	$16217_{2}^{{}^{\circ}}$ – 33721_2_
5711.023	1		17505.141	$14032_{2}^{{}^{\circ}}$ – 31537_3_
5710.270	4		17507.449	$18069_{3}^{{}^{\circ}}$ – 35576_3_
5709.858	1		17508.713	19532_4_ – $37041_{4}^{{}^{\circ}}$
5708.679	50	3	17512.329	14204_5_ – $31716_{0}^{{}^{\circ}}$
5705.866	8	1	17520.962	19713_3_ – $37234_{2}^{{}^{\circ}}$
5705.637	50	4	17521.665	$19227_{6}^{{}^{\circ}}$ – 36749_6_
5705.483	10	1	17522.138	21143_5_ – $38665_{0}^{{}^{\circ}}$
5705.232	4	1	17522.909	$18053_{4}^{{}^{\circ}}$ – 35576_3_
5704.124	8	1	17526.313	$18809_{4}^{{}^{\circ}}$ – 36336_3_
5702.650	50	25	17530.843	5563_1_ – $23093_{2}^{{}^{\circ}}$
5702.543	1		17531.172	$22163_{4}^{{}^{\circ}}$ – 39694_5_
5700.782	50	8	17536.587	$12114_{2}^{{}^{\circ}}$ – 29650_2_
5698.292	50	5	17544.250	$11877_{1}^{{}^{\circ}}$ – 29422_1_
5696.391	50	8	17550.105	15493_4_ – $33043_{3}^{{}^{\circ}}$
5694.434	10	1	17556.136	$16217_{2}^{{}^{\circ}}$ – 33773_1_
5693.759	1		17558.218	$17847_{2}^{{}^{\circ}}$ – 35405_3_
5693.571	8		17558.798	7280_2_ – $24838_{1}^{{}^{\circ}}$
5692.159	10	1	17563.153	15863_2_ – $33427_{2}^{{}^{\circ}}$
5691.906	3		17563.934	19273_2_ – $36837_{1}^{{}^{\circ}}$
5691.681	5		17564.628	3687_2_ – $21252_{2}^{{}^{\circ}}$
5691.060	10b		17566.545	$10414_{4}^{{}^{\circ}}$ – 27980_3_
5690.693	40	3	17567.678	$14206_{4}^{{}^{\circ}}$ – 31774_3_
5690.118	10	3	17569.453	$20214_{3}^{{}^{\circ}}$ – 37784_2_
5689.647	10	1	17570.907	$18011_{0}^{{}^{\circ}}$ – 35582_5_
5689.477	1		17571.432	$22855_{3}^{{}^{\circ}}$ – 40426_4_
5688.527	1		17574.367	$20522_{2}^{{}^{\circ}}$ – 38097_2_
5687.997	2c		17576.004	$14465_{2}^{{}^{\circ}}$ – 32041_2_
5687.350	501	3	17578.004	$15490_{0}^{{}^{\circ}}$ – 33068_6_
5686.876	10	1	17579.469	8111_4_ – $25690_{0}^{{}^{\circ}}$
5685.826	3		17582.715	$19503_{3}^{{}^{\circ}}$ – 37085_4_
5685.192	100	8	17584.676	8800_4_ – $26384_{4}^{{}^{\circ}}$
5684.866	4		17585.684	$18930_{3}^{{}^{\circ}}$ – 36515_3_
5683.486	20	2	17589.954	15970_3_ – $33560_{4}^{{}^{\circ}}$
5682.846	8	1	17591.935	$13945_{3}^{{}^{\circ}}$ – 31537_3_
5682.238	4		17593.818	$17847_{2}^{{}^{\circ}}$ – 35440_3_
5681.441	1		17596.286	11601_1_ – $29197_{1}^{{}^{\circ}}$
5679.535	3		17602.191	20054_2_ – $37656_{3}^{{}^{\circ}}$
5679.004	50	5	17603.836	$11241_{3}^{{}^{\circ}}$ – 28845_4_
5677.729	2h		17607.790	$20922_{2}^{{}^{\circ}}$ – 38529_3_
5677.051	200b	8	17609.892	$7795_{4}^{{}^{\circ}}$ – 25405_4_
5675.701	2		17614.081	$19516_{2}^{{}^{\circ}}$ – 37131_3_
5675.601	2		17614.391	$19039_{2}^{{}^{\circ}}$ – 36653_2_
5675.501	15	2	17614.702	$15166_{3}^{{}^{\circ}}$ – 32781_4_
5674.990	200b	25	17616.288	11802_2_ – $29419_{2}^{{}^{\circ}}$
5674.461	15	2	17617.930	$21902_{4}^{{}^{\circ}}$ – 39520_4_
5674.171	2		17618.830	21645_4_ – $39264_{3}^{{}^{\circ}}$
5673.955	5	1	17619.501	$20566_{4}^{{}^{\circ}}$ – 38186_4_
5673.835	50	5	17619.874	$10414_{4}^{{}^{\circ}}$ – 28034_5_
5673.009	5	1	17622.439	$18011_{0}^{{}^{\circ}}$ – 35633_4_
5672.724	5	1	17623.325	$19588_{0}^{{}^{\circ}}$ – 37211_5_
5671.498	15	2	17627.134	19532_4_ – $37159_{3}^{{}^{\circ}}$
5671.250	10	2	17627.905	$19503_{3}^{{}^{\circ}}$ – 37131_3_
5671.072	40	2	17628.458	16554_6_ – $34182_{0}^{{}^{\circ}}$
5670.827	3		17629.220	$15166_{3}^{{}^{\circ}}$ – 32796_3_
5670.199	1		17631.172	18431_3_ – $36062_{4}^{{}^{\circ}}$
5668.402	3		17636.762	13962_1_ – $31599_{2}^{{}^{\circ}}$
5668.016	1		17637.963	$21645_{4}^{{}^{\circ}}$ – 39283_4_
5667.509	2		17639.541	18549_2_ – $36188_{2}^{{}^{\circ}}$
5667.129	150	15	17640.723	$11241_{3}^{{}^{\circ}}$ – 28882_2_
5666.419	10	1	17642.934	8111_4_ – $25753_{0}^{{}^{\circ}}$
5665.181	200	50	17646.789	$8243_{2}^{{}^{\circ}}$ – 25890_2_
5664.621	75	15	17648.534	$11197_{0}^{{}^{\circ}}$ – 28845_4_
5664.228	40	2	17649.758	$17224_{2}^{{}^{\circ}}$ – 34874_3_
5663.041	50	3	17653.458	2869_3_ – $20522_{2}^{{}^{\circ}}$
5662.949	3		17653.745	$15736_{1}^{{}^{\circ}}$ – 33390_1_
5660.364				18699_2_ – $36361_{1}^{{}^{\circ}}$
5659.878	2		17663.323	$16783_{4}^{{}^{\circ}}$ – 34447_4_
5659.216	5	1	17665.389	21645_4_ – $39311_{4}^{{}^{\circ}}$
5657.926	100	25	17669.417	12847_3_ – $30517_{4}^{{}^{\circ}}$
5656.729	40	2	17673.156	13088_3_ – $30761_{3}^{{}^{\circ}}$
5656.302	10	2	17674.490	$22163_{4}^{{}^{\circ}}$ – 39837_3_
5655.750	10	3	17676.215	13847_2_ – $31523_{3}^{{}^{\circ}}$
5655.490	20	2	17677.028	$16783_{4}^{{}^{\circ}}$ – 34460_5_
5655.213	2		17677.893	$20214_{3}^{{}^{\circ}}$ – 37892_4_
5655.135	2		17678.137	$17411_{3}^{{}^{\circ}}$ – 35089_3_
5653.838	2		17682.193	20054_2_ – $37736_{2}^{{}^{\circ}}$
5653.426	2		17683.481	$21077_{0}^{{}^{\circ}}$ – 38760_4_
5652.570	1		17686.159	$23015_{0}^{{}^{\circ}}$ – 40701_4_
5651.326	5		17690.052	$15618_{3}^{{}^{\circ}}$ – 33309_2_
5650.912	1		17691.348	$17354_{1}^{{}^{\circ}}$ – 35046_1_
5650.354	75	15	17693.095	13297_4_ – $30990_{3}^{{}^{\circ}}$
5649.737	1		17695.028	22401_1_ – $40096_{2}^{{}^{\circ}}$
5648.990	75	10	17697.368	2869_3_ – $20566_{4}^{{}^{\circ}}$
5647.708	20	2	17701.385	$10526_{3}^{{}^{\circ}}$ – 28227_4_
5646.455	50	10	17705.313	12847_3_ – $30553_{2}^{{}^{\circ}}$
5646.255	1		17705.940	$21645_{4}^{{}^{\circ}}$ – 39351_5_
5646.205	1		17706.097	$18809_{4}^{{}^{\circ}}$ – 36515_3_
5645.668	50	20	17707.781	8800_4_ – $26508_{3}^{{}^{\circ}}$
5645.529	50	8	17708.217	4961_4_ – $22669_{3}^{{}^{\circ}}$
5643.346	8	1	17715.067	$19588_{0}^{{}^{\circ}}$ – 37303_4_
5643.110	20	2	17715.808	$17501_{0}^{{}^{\circ}}$ – 35216_5_
5641.735	75	8	17720.125	$14481_{6}^{{}^{\circ}}$ – 32202_5_
5641.562	50	5	17720.669	$12114_{2}^{{}^{\circ}}$ – 29835_3_
5641.242	20	5	17721.674	13297_4_ – $31019_{4}^{{}^{\circ}}$
5640.363	50	2	17724.436	13088_3_ – $30812_{2}^{{}^{\circ}}$
5639.460	10	1	17727.274	15863_2_ – $33591_{3}^{{}^{\circ}}$
5638.767	1		17729.452	$17847_{2}^{{}^{\circ}}$ – 35576_3_
5636.625	1		17736.190	$19039_{2}^{{}^{\circ}}$ – 36775_3_
5635.889	75	15	17738.506	$17411_{3}^{{}^{\circ}}$ – 35149_2_
5635.482	2		17739.787	$19948_{4}^{{}^{\circ}}$ – 37688_3_
5635.175	5	1	17740.753	$18053_{4}^{{}^{\circ}}$ – 35794_4_
5634.619	10	2	17742.504	$14032_{2}^{{}^{\circ}}$ – 31774_3_
5634.097	8s	1	17744.148	$19588_{0}^{{}^{\circ}}$ – 37332_6_
5633.297	75	8	17746.668	$10526_{3}^{{}^{\circ}}$ – 28273_2_
5632.903	2b		17747.909	$10783_{2}^{{}^{\circ}}$ – 28531_2_
5632.776	50	3	17748.309	15970_3_ – $33718_{2}^{{}^{\circ}}$
5632.494	25	1	17749.198	14204_5_ – $31953_{4}^{{}^{\circ}}$
5632.164	5b		17750.238	13962_1_ – $31712_{1}^{{}^{\circ}}$
5630.297	75	10	17756.123	$19986_{6}^{{}^{\circ}}$ – 37742_6_
5629.312	50	3	17759.230	16351_0_ – $34111_{1}^{{}^{\circ}}$
5628.365	8	1	17762.218	$18069_{3}^{{}^{\circ}}$ – 35831_3_
5627.606	3		17764.614	15970_3_ – $33734_{2}^{{}^{\circ}}$
5627.012	3		17766.489	8111_4_ – $25877_{4}^{{}^{\circ}}$
5626.169	1		17769.151	$19039_{2}^{{}^{\circ}}$ – 36808_3_
5624.911	50	3	17773.125	$11877_{1}^{{}^{\circ}}$ – 29650_2_
5624.775	15	2	17773.555	17959_4_ – $35733_{4}^{{}^{\circ}}$
5623.581	25	2	17777.329	15493_4_ – $33270_{4}^{{}^{\circ}}$
5623.474	1		17777.667	$18053_{4}^{{}^{\circ}}$ – 35831_3_
5623.411	2		17777.866	17073_1_ – $34851_{2}^{{}^{\circ}}$
5621.788	20	3	17782.998	$18011_{0}^{{}^{\circ}}$ – 35794_4_
5621.287	8	2	17784.583	$20322_{0}^{{}^{\circ}}$ – 38106_5_
5620.013	15b	5	17788.615	$18011_{0}^{{}^{\circ}}$ – 35799_6_
5619.976	50b	8	17788.732	$14206_{4}^{{}^{\circ}}$ – 31995_4_
5619.719	5b	1	17789.546	$13175_{4}^{{}^{\circ}}$ – 30964_3_
5619.684	4b		17789.656	$15618_{3}^{{}^{\circ}}$ – 33408_3_
5619.526	1		17790.156	$19516_{2}^{{}^{\circ}}$ – 37307_3_
5619.478	1		17790.308	$21890_{3}^{{}^{\circ}}$ – 39680_2_
5619.017	50	4	17791.768	$21165_{3}^{{}^{\circ}}$ – 38956_4_
5618.927	15	1	17792.053	$21902_{4}^{{}^{\circ}}$ – 39694_5_
5617.964	2		17795.103	$17354_{1}^{{}^{\circ}}$ – 35149_2_
5617.291	15	2	17797.235	$14243_{1}^{{}^{\circ}}$ – 32041_2_
5616.945	3	1	17798.331	15863_2_ – $33662_{1}^{{}^{\circ}}$
5616.691	20	2	17799.136	$15618_{3}^{{}^{\circ}}$ – 33418_4_
5616.503	10	2	17799.732	$17501_{0}^{{}^{\circ}}$ – 35300_5_
5616.438	2		17799.938	$20566_{4}^{{}^{\circ}}$ – 38366_4_
5616.328	8	2	17800.286	$19503_{3}^{{}^{\circ}}$ – 37303_4_
5615.879	10	3	17801.709	15493_4_ – $33294_{3}^{{}^{\circ}}$
5615.320	400r	15	17803.482	3865_1_ – $21668_{1}^{{}^{\circ}}$
5614.515	5	1	17806.034	$14206_{4}^{{}^{\circ}}$ – 32012_4_
5613.659				$18069_{3}^{{}^{\circ}}$ – 35877_2_
5612.619	75	3	17812.049	$14481_{6}^{{}^{\circ}}$ – 32293_5_
5612.619	75	3	17812.049	18549_2_ – $36361_{1}^{{}^{\circ}}$
5612.068	150	20	17813.798	$10414_{4}^{{}^{\circ}}$ – 28227_4_
5610.685	200	20	17818.189	11601_1_ – $29419_{2}^{{}^{\circ}}$
5610.329	50	3	17819.320	14204_5_ – $32023_{0}^{{}^{\circ}}$
5610.235	75	5	17819.618	7502_3_ – $25321_{3}^{{}^{\circ}}$
5610.111	75	4	17820.012	6362_2_ – $24182_{2}^{{}^{\circ}}$
5609.857	15	1	17820.819	$16346_{4}^{{}^{\circ}}$ – 34167_3_
5609.585	150	10	17821.683	$17224_{2}^{{}^{\circ}}$ – 35046_1_
5607.197	15	1	17829.273	$13945_{3}^{{}^{\circ}}$ – 31774_3_
5607.087	10	1	17829.623	15970_3_ – $33799_{4}^{{}^{\circ}}$
5606.390	100	10	17831.839	$10526_{3}^{{}^{\circ}}$ – 28358_3_
5604.814				21575_2_ – $39411_{1}^{{}^{\circ}}$
5603.764	20	1	17840.195	$15618_{3}^{{}^{\circ}}$ – 33459_4_
5603.060	8s		17842.437	17398_3_ – $35240_{3}^{{}^{\circ}}$
5602.627	15	1	17843.816	13297_4_ – $31141_{0}^{{}^{\circ}}$
5602.216	2b		17845.125	8800_4_ – $26645_{0}^{{}^{\circ}}$
5601.934	2		17846.023	$22855_{3}^{{}^{\circ}}$ – 40701_4_
5601.604	200	8	17847.074	0_2_ – $17847_{2}^{{}^{\circ}}$
5601.604	200	8	17847.074	5563_1_ – $23410_{0}^{{}^{\circ}}$
5601.506	8b		17847.387	$12114_{2}^{{}^{\circ}}$ – 29961_1_
5601.191	4		17848.390	$13945_{3}^{{}^{\circ}}$ – 31793_4_
5599.865	3		17852.617	17959_4_ – $35812_{3}^{{}^{\circ}}$
5599.655	50	2	17853.286	7502_3_ – $25355_{4}^{{}^{\circ}}$
5599.573	2		17853.548	14226_0_ – $32080_{1}^{{}^{\circ}}$
5599.274	10b		17854.501	15863_2_ – $33718_{2}^{{}^{\circ}}$
5598.445	4		17857.145	18699_2_ – $36556_{1}^{{}^{\circ}}$
5596.836	5		17862.278	$11241_{3}^{{}^{\circ}}$ – 29104_4_
5595.962	75	8	17865.068	$17224_{2}^{{}^{\circ}}$ – 35089_3_
5595.847	50	3	17865.435	2558_0_ – $20423_{1}^{{}^{\circ}}$
5595.778	5		17865.656	$20737_{1}^{{}^{\circ}}$ – 38602_2_
5595.064	300	25	17867.935	$8243_{2}^{{}^{\circ}}$ – 26111_1_
5594.161	15	1	17870.820	15863_2_ – $33734_{2}^{{}^{\circ}}$
5592.597	2		17875.817	19273_2_ – $37149_{2}^{{}^{\circ}}$
5591.907	15b	3	17878.023	18549_2_ – $36427_{3}^{{}^{\circ}}$
5591.907	15b	3	17878.023	$18930_{3}^{{}^{\circ}}$ – 36808_3_
5590.215	20		17883.434	11802_2_ – $29686_{3}^{{}^{\circ}}$
5589.560	1		17885.530	$20922_{2}^{{}^{\circ}}$ – 38807_2_
5589.412	2		17886.003	19273_2_ – $37159_{1}^{{}^{\circ}}$
5589.328	5	1	17886.272	19273_2_ – $37159_{3}^{{}^{\circ}}$
5587.027	500r	50	17893.638	4961_4_ – $22855_{3}^{{}^{\circ}}$
5586.255	2		17896.111	$10783_{2}^{{}^{\circ}}$ – 28679_2_
5585.981	4		17896.989	$12114_{2}^{{}^{\circ}}$ – 30011_3_
5585.884	5b		17897.300	13297_4_ – $31194_{4}^{{}^{\circ}}$
5585.848	151	1	17897.415	$14032_{2}^{{}^{\circ}}$ – 31929_3_
5584.983	2		17900.187	20054_2_ – $37954_{3}^{{}^{\circ}}$
5584.430	4		17901.960	13088_3_ – $30990_{3}^{{}^{\circ}}$
5582.865	50	2	17906.978	$11197_{0}^{{}^{\circ}}$ – 29104_4_
5582.678	50	3	17907.578	13962_1_ – $31870_{2}^{{}^{\circ}}$
5582.367	75	4	17908.575	$15618_{3}^{{}^{\circ}}$ – 33527_4_
5580.756	100	10	17913.745	12847_3_ – $30761_{3}^{{}^{\circ}}$
5580.395	50	10	17914.904	17166_5_ – $35081_{0}^{{}^{\circ}}$
5580.221	3		17915.462	20054_2_ – $37970_{2}^{{}^{\circ}}$
5580.076	50	2	17915.928	4961_4_ – $22877_{0}^{{}^{\circ}}$
5579.990	4		17916.204	21645_4_ – $39562_{4}^{{}^{\circ}}$
5579.359	300	40	17918.230	5563_1_ – $23481_{1}^{{}^{\circ}}$
5578.091	2		17922.303	$21252_{2}^{{}^{\circ}}$ – 39174_3_
5577.602	10		17923.875	$16783_{4}^{{}^{\circ}}$ – 34707_3_
5577.115	5	1	17925.440	$17224_{2}^{{}^{\circ}}$ – 35149_2_
5576.300	40	3	17928.060	$15490_{0}^{{}^{\circ}}$ – 33418_4_
5576.205	100	10	17928.365	$10414_{4}^{{}^{\circ}}$ – 28342_5_
5575.524	3		17930.555	13088_3_ – $31019_{4}^{{}^{\circ}}$
5575.446	4		17930.806	$20214_{3}^{{}^{\circ}}$ – 38145_3_
5574.028	2		17935.367	$21902_{4}^{{}^{\circ}}$ – 39837_3_
5573.599	2		17936.748	15863_2_ – $33800_{3}^{{}^{\circ}}$
5573.355	400	20	17937.533	8111_4_ – $26048_{4}^{{}^{\circ}}$
5572.766	10s	1	17939.429	18549_2_ – $36488_{2}^{{}^{\circ}}$
5572.466	200	8	17940.394	7502_3_ – $25442_{3}^{{}^{\circ}}$
5572.095	20	1	17941.589	11802_2_ – $29744_{3}^{{}^{\circ}}$
5571.253	75b	10	17944.301	$10414_{4}^{{}^{\circ}}$ – 28358_3_
5570.928	50	2	17945.347	6362_2_ – $24307_{2}^{{}^{\circ}}$
5570.762	8	1	17945.882	$15618_{3}^{{}^{\circ}}$ – 33564_3_
5570.265	1		17947.483	$21890_{3}^{{}^{\circ}}$ – 39837_3_
5569.486	2		17949.993	$16217_{2}^{{}^{\circ}}$ – 34167_3_
5568.840	15		17952.076	$15736_{1}^{{}^{\circ}}$ – 33689_2_
5568.542	50	3	17953.036	17398_3_ – $35351_{4}^{{}^{\circ}}$
5568.000	300b	150	17954.784	$16346_{4}^{{}^{\circ}}$ – 34301_4_
5567.942	25b	10	17954.971	$18930_{3}^{{}^{\circ}}$ – 36885_4_
5564.806	25	2	17965.089	17166_5_ – $35131_{4}^{{}^{\circ}}$
5564.691	2		17965.461	$18809_{4}^{{}^{\circ}}$ – 36775_3_
5563.582	4		17969.042	18699_2_ – $36668_{2}^{{}^{\circ}}$
5562.847	5		17971.416	$18809_{4}^{{}^{\circ}}$ – 36781_5_
5560.000	3h		17980.618	$21539_{4}^{{}^{\circ}}$ – 39520_4_
5559.894	150	8	17980.961	3687_2_ – $21668_{1}^{{}^{\circ}}$
5559.526	3		17982.151	18574_1_ – $36556_{1}^{{}^{\circ}}$
5558.882	8b	3	17984.234	$17847_{2}^{{}^{\circ}}$ – 35831_3_
5558.848	75b	3	17984.344	$15736_{1}^{{}^{\circ}}$ – 33721_2_
5558.848	75b	3	17984.344	$19227_{6}^{{}^{\circ}}$ – 37211_5_
5558.433	75b	25b	17985.687	13297_4_ – $31283_{3}^{{}^{\circ}}$
5558.342	400b	50	17985.981	8111_4_ – $26096_{3}^{{}^{\circ}}$
5557.924	25	5	17987.334	13088_3_ – $31075_{2}^{{}^{\circ}}$
5557.045	300	15	17990.179	8800_4_ – $26790_{4}^{{}^{\circ}}$
5555.839	10	1	17994.084	$17411_{3}^{{}^{\circ}}$ – 35405_3_
5555.534	75	4	17995.072	$14206_{4}^{{}^{\circ}}$ – 32202_5_
5554.695	5		17997.790	15970_3_ – $33967_{3}^{{}^{\circ}}$
5554.546	10b	2	17998.273	20054_2_ – $38053_{3}^{{}^{\circ}}$
5554.502	20	4	17998.415	$18809_{4}^{{}^{\circ}}$ – 36808_3_
5552.624	75	4	18004.503	$10526_{3}^{{}^{\circ}}$ – 28531_2_
5551.744	25	5	18007.357	18549_2_ – $36556_{1}^{{}^{\circ}}$
5551.193	40	2	18009.144	$14032_{2}^{{}^{\circ}}$ – 32041_2_
5550.988	2		18009.809	$21165_{3}^{{}^{\circ}}$ – 39174_3_
5550.476	2		18011.470	$21668_{1}^{{}^{\circ}}$ – 39680_2_
5550.456	1		18011.535	$21668_{1}^{{}^{\circ}}$ – 39680_2_
5548.377	15	5	18018.284	$12114_{2}^{{}^{\circ}}$ – 30132_2_
5548.176	400r	40b	18018.937	6362_2_ – $24381_{2}^{{}^{\circ}}$
5547.654	15	1	18020.632	$18069_{3}^{{}^{\circ}}$ – 36089_4_
5547.135	75	3	18022.318	13847_2_ – $31870_{2}^{{}^{\circ}}$
5546.638	4		18023.933	19713_3_ – $37736_{2}^{{}^{\circ}}$
5545.806	15		18026.637	7280_2_ – $25306_{2}^{{}^{\circ}}$
5545.586	2		18027.352	$22399_{0}^{{}^{\circ}}$ – 40426_4_
5544.879	1		18029.651	$17411_{3}^{{}^{\circ}}$ – 35440_3_
5544.543	2		18030.743	$17847_{2}^{{}^{\circ}}$ – 35877_2_
5544.385	3		18031.257	15970_3_ – $34001_{4}^{{}^{\circ}}$
5544.385	3		18031.257	15970_3_ – $34001_{4}^{{}^{\circ}}$
5544.252	4	1	18031.690	$22855_{3}^{{}^{\circ}}$ – 40886_2_
5543.480	10	1	18034.201	20054_2_ – $38088_{2}^{{}^{\circ}}$
5542.890	300	10	18036.121	$10526_{3}^{{}^{\circ}}$ – 28562_4_
5542.027	3		18038.929	19532_4_ – $37571_{0}^{{}^{\circ}}$
5541.936	50	2	18039.225	11601_1_ – $29640_{1}^{{}^{\circ}}$
5541.936	50	2	18039.225	$18614_{1}^{{}^{\circ}}$ – 36653_2_
5541.581	50	2	18040.381	5563_1_ – $23603_{2}^{{}^{\circ}}$
5541.146	50	2	18041.797	7280_2_ – $25321_{3}^{{}^{\circ}}$
5540.521	2		18043.832	$19948_{4}^{{}^{\circ}}$ – 37992_4_
5540.380	1		18044.292	$20322_{0}^{{}^{\circ}}$ – 38366_4_
5539.262	500r	50	18047.933	9804_5_ – $27852_{0}^{{}^{\circ}}$
5538.608	75	4	18050.065	3687_2_ – $21738_{2}^{{}^{\circ}}$
5537.749	1		18052.864	2869_3_ – $20922_{2}^{{}^{\circ}}$
5537.555	50	2	18053.497	4961_4_ – $23015_{0}^{{}^{\circ}}$
5536.436	4		18057.146	18431_3_ – $36488_{2}^{{}^{\circ}}$
5535.970	50	1	18058.666	6362_2_ – $24421_{3}^{{}^{\circ}}$
5534.586	5		18063.181	18699_2_ – $36762_{3}^{{}^{\circ}}$
5534.439	5		18063.661	17398_3_ – $35462_{3}^{{}^{\circ}}$
5533.963	4		18065.215	$16783_{4}^{{}^{\circ}}$ – 34849_5_
5533.608	5		18066.374	$17354_{1}^{{}^{\circ}}$ – 35421_1_
5533.467	5	1	18066.834	15493_4_ – $33560_{4}^{{}^{\circ}}$
5533.219	10b		18067.644	$13945_{3}^{{}^{\circ}}$ – 32012_4_
5532.780	5		18069.077	0_2_ – $18069_{3}^{{}^{\circ}}$
5532.481	1		18070.054	$15618_{3}^{{}^{\circ}}$ – 33689_2_
5531.483	1		18073.314	19532_4_ – $37605_{4}^{{}^{\circ}}$
5530.843	2		18075.405	$18809_{4}^{{}^{\circ}}$ – 36885_4_
5530.075	50	2	18077.916	8800_4_ – $26878_{3}^{{}^{\circ}}$
5529.472	8		18079.887	$18053_{4}^{{}^{\circ}}$ – 36133_5_
5529.096	75	10	18081.117	$17501_{0}^{{}^{\circ}}$ – 35582_5_
5528.226	50b	5	18083.962	$11877_{1}^{{}^{\circ}}$ – 29961_1_
5527.480	10	1	18086.403	$14465_{2}^{{}^{\circ}}$ – 32551_3_
5527.295	200	8	18087.008	$14206_{4}^{{}^{\circ}}$ – 32293_5_
5527.074	2		18087.731	$22338_{3}^{{}^{\circ}}$ – 40426_4_
5526.983	1		18088.029	$20922_{2}^{{}^{\circ}}$ – 39010_3_
5526.528	50	8	18089.518	$16783_{4}^{{}^{\circ}}$ – 34873_4_
5526.434	8	1	18089.826	14204_5_ – $32294_{4}^{{}^{\circ}}$
5526.313	8		18090.222	$16783_{4}^{{}^{\circ}}$ – 34874_3_
5525.799	2		18091.905	$19039_{2}^{{}^{\circ}}$ – 37131_3_
5525.155	20	2	18094.013	18574_1_ – $36668_{2}^{{}^{\circ}}$
5524.583	150b	10b	18095.887	$13175_{4}^{{}^{\circ}}$ – 31271_5_
5523.951	10		18097.957	15493_4_ – $33591_{3}^{{}^{\circ}}$
5523.539	8	2	18099.307	$10783_{2}^{{}^{\circ}}$ – 28882_2_
5523.451	5		18099.596	$21738_{2}^{{}^{\circ}}$ – 39837_3_
5523.164	25	1	18100.536	$16346_{4}^{{}^{\circ}}$ – 34447_4_
5522.616	8		18102.332	$15618_{3}^{{}^{\circ}}$ – 33721_2_
5522.416	3		18102.988	17959_4_ – $36062_{4}^{{}^{\circ}}$
5522.104	8		18104.010	15863_2_ – $33967_{3}^{{}^{\circ}}$
5521.751	200	8	18105.168	$19227_{6}^{{}^{\circ}}$ – 37332_6_
5520.954	5		18107.781	17166_5_ – $35273_{0}^{{}^{\circ}}$
5518.988	200b	50	18114.232	$16346_{4}^{{}^{\circ}}$ – 34460_5_
5517.874	3		18117.889	13962_1_ – $32080_{1}^{{}^{\circ}}$
5517.616	4		18118.736	$21165_{3}^{{}^{\circ}}$ – 39283_4_
5517.455	4		18119.265	18549_2_ – $36668_{2}^{{}^{\circ}}$
5515.845	4		18124.554	19532_4_ – $37656_{3}^{{}^{\circ}}$
5514.873	200b	10	18127.748	$7795_{4}^{{}^{\circ}}$ – 25923_4_
5513.370	2		18132.690	$17501_{0}^{{}^{\circ}}$ – 35633_4_
5513.084	2		18133.630	$20566_{4}^{{}^{\circ}}$ – 38700_4_
5510.495	20	1	18142.150	$15166_{3}^{{}^{\circ}}$ – 33309_2_
5510.372	15	1	18142.555	12847_3_ – $30990_{3}^{{}^{\circ}}$
5509.993	400	20	18143.803	9804_5_ – $27948_{4}^{{}^{\circ}}$
5508.557	75	5	18148.533	$10414_{4}^{{}^{\circ}}$ – 28562_4_
5508.050	8	1	18150.203	$21902_{4}^{{}^{\circ}}$ – 40052_4_
5507.542	1001	2	18151.877	4961_4_ – $23113_{4}^{{}^{\circ}}$
5507.542	1001	2	18151.877	$13175_{4}^{{}^{\circ}}$ – 31326_4_
5507.295	1		18152.691	$10526_{3}^{{}^{\circ}}$ – 28679_2_
5506.919	50	5	18153.931	$19588_{0}^{{}^{\circ}}$ – 37742_6_
5506.676	1		18154.732	$21539_{4}^{{}^{\circ}}$ – 39694_5_
5506.423	3		18155.566	$18930_{3}^{{}^{\circ}}$ – 37085_4_
5506.078	5		18156.703	$21165_{3}^{{}^{\circ}}$ – 39321_3_
5505.934	5		18157.178	$18053_{4}^{{}^{\circ}}$ – 36210_5_
5505.547	2		18158.455	$19948_{4}^{{}^{\circ}}$ – 38106_5_
5504.302	200	20	18162.562	7280_2_ – $25442_{3}^{{}^{\circ}}$
5503.698	2		18164.555	$18930_{3}^{{}^{\circ}}$ – 37094_2_
5503.473	3		18165.298	$17411_{3}^{{}^{\circ}}$ – 35576_3_
5502.718	5		18167.790	$20566_{4}^{{}^{\circ}}$ – 38734_3_
5501.701	8	2	18171.148	12847_3_ – $31019_{4}^{{}^{\circ}}$
5501.467	5		18171.921	22098_4_ – $40270_{4}^{{}^{\circ}}$
5501.281	100	10	18172.536	$12114_{2}^{{}^{\circ}}$ – 30286_1_
5500.032	8		18176.662	$11241_{3}^{{}^{\circ}}$ – 29418_2_
5499.647	75	5	18177.935	5563_1_ – $23741_{1}^{{}^{\circ}}$
5499.255	300	40	18179.230	2558_0_ – $20737_{1}^{{}^{\circ}}$
5498.716	8	1	18181.012	$17224_{2}^{{}^{\circ}}$ – 35405_3_
5497.401	2b		18185.361	17166_5_ – $35351_{4}^{{}^{\circ}}$
5496.138	200	10	18189.540	5563_1_ – $23752_{2}^{{}^{\circ}}$
5494.797	2		18193.979	$20566_{4}^{{}^{\circ}}$ – 38760_4_
5494.619	5		18194.569	13088_3_ – $31283_{3}^{{}^{\circ}}$
5494.330	75	10	18195.526	8800_4_ – $26995_{3}^{{}^{\circ}}$
5493.973	5		18196.708	$17224_{2}^{{}^{\circ}}$ – 35421_1_
5493.594	10	1	18197.963	13962_1_ – $32160_{2}^{{}^{\circ}}$
5493.196	100	8	18199.282	6362_2_ – $24561_{3}^{{}^{\circ}}$
5492.912	15	3	18200.223	$17847_{2}^{{}^{\circ}}$ – 36047_2_
5492.642	100	10	18201.117	7502_3_ – $25703_{2}^{{}^{\circ}}$
5492.332	40	20	18202.145	3687_2_ – $21890_{3}^{{}^{\circ}}$
5489.086	75	5	18212.909	$15618_{3}^{{}^{\circ}}$ – 33831_4_
5488.795	3		18213.874	$16217_{2}^{{}^{\circ}}$ – 34431_3_
5488.207	4		18215.826	$23015_{0}^{{}^{\circ}}$ – 41230_4_
5487.971	15	1	18216.609	$17224_{2}^{{}^{\circ}}$ – 35440_3_
5487.837	15	2	18217.054	$22669_{3}^{{}^{\circ}}$ – 40886_2_
5487.505	15	2	18218.156	17959_4_ – $36178_{4}^{{}^{\circ}}$
5485.714	4		18224.104	20054_2_ – $38278_{1}^{{}^{\circ}}$
5484.988	50	3	18226.516	13297_4_ – $31523_{3}^{{}^{\circ}}$
5484.556	20	1	18227.951	12847_3_ – $31075_{2}^{{}^{\circ}}$
5484.469	8	1	18228.241	$18069_{3}^{{}^{\circ}}$ – 36297_4_
5483.709	2		18230.767	$19588_{0}^{{}^{\circ}}$ – 37819_4_
5483.623	3		18231.053	$19516_{2}^{{}^{\circ}}$ – 37748_2_
5483.143	50	2	18232.649	13847_2_ – $32080_{1}^{{}^{\circ}}$
5482.498	75	8	18234.794	14204_5_ – $32439_{4}^{{}^{\circ}}$
5481.828	8	1	18237.023	19713_3_ – $37950_{4}^{{}^{\circ}}$
5481.612	2		18237.741	$19948_{4}^{{}^{\circ}}$ – 38186_4_
5481.211	5		18239.075	16351_0_ – $34591_{1}^{{}^{\circ}}$
5480.402	4	1	18241.768	$15166_{3}^{{}^{\circ}}$ – 33408_3_
5479.462	8		18244.897	$19503_{3}^{{}^{\circ}}$ – 37748_2_
5479.072	75	75	18246.196	7280_2_ – $25526_{1}^{{}^{\circ}}$
5478.742	50	3	18247.295	15863_2_ – $34111_{1}^{{}^{\circ}}$
5477.550	40	2	18251.266	$14206_{4}^{{}^{\circ}}$ – 32458_4_
5476.482	20	2	18254.825	$11877_{1}^{{}^{\circ}}$ – 30132_2_
5476.482	20	2	18254.825	17073_1_ – $35328_{1}^{{}^{\circ}}$
5475.767	4h		18257.208	19713_3_ – $37970_{2}^{{}^{\circ}}$
5474.145	2		18262.618	18574_1_ – $36837_{1}^{{}^{\circ}}$
5473.849	10	1	18263.606	$22163_{4}^{{}^{\circ}}$ – 40426_4_
5472.701	8		18267.437	$19516_{2}^{{}^{\circ}}$ – 37784_2_
5472.535	25	1	18267.991	$19039_{2}^{{}^{\circ}}$ – 37307_3_
5470.759	150	10	18273.921	8111_4_ – $26384_{4}^{{}^{\circ}}$
5470.145	50	8	18275.972	$18809_{4}^{{}^{\circ}}$ – 37085_4_
5468.569	1		18281.239	$19503_{3}^{{}^{\circ}}$ – 37784_2_
5468.166	2		18282.586	$18053_{4}^{{}^{\circ}}$ – 36336_3_
5467.403	5	1	18285.138	$22141_{3}^{{}^{\circ}}$ – 40426_4_
5467.167	75	4	18285.927	$18011_{0}^{{}^{\circ}}$ – 36297_4_
5466.602	5	1	18287.817	18549_2_ – $36837_{1}^{{}^{\circ}}$
5465.268	5		18292.281	$15166_{3}^{{}^{\circ}}$ – 33459_4_
5464.205	200w	8	18295.839	2869_3_ – $21165_{3}^{{}^{\circ}}$
5463.771	50	4	18297.293	18574_1_ – $36871_{2}^{{}^{\circ}}$
5463.320	40	10h	18298.803	$17501_{0}^{{}^{\circ}}$ – 35799_6_
5462.809	3b		18300.515	20054_2_ – $38355_{2}^{{}^{\circ}}$
5462.336	50	15	18302.099	$19588_{0}^{{}^{\circ}}$ – 37890_5_
5461.636	5	2b	18304.445	$19588_{0}^{{}^{\circ}}$ – 37892_4_
5461.314	3		18305.524	$16783_{4}^{{}^{\circ}}$ – 35089_3_
5461.019	5		18306.513	15493_4_ – $33799_{4}^{{}^{\circ}}$
5459.171	15	3	18312.710	13847_2_ – $32160_{2}^{{}^{\circ}}$
5458.492	1		18314.988	$20214_{3}^{{}^{\circ}}$ – 38529_3_
5457.289	50	3	18319.025	$10526_{3}^{{}^{\circ}}$ – 28845_4_
5456.654	8	1	18321.157	$18809_{4}^{{}^{\circ}}$ – 37131_3_
5456.255	40	2	18322.497	18549_2_ – $36871_{2}^{{}^{\circ}}$
5454.919	10	1	18326.984	22098_4_ – $40425_{4}^{{}^{\circ}}$
5453.757	1		18330.889	$14465_{2}^{{}^{\circ}}$ – 32796_3_
5453.686	4	1	18331.128	18431_3_ – $36762_{3}^{{}^{\circ}}$
5453.297	5		18332.435	16554_6_ – $34886_{0}^{{}^{\circ}}$
5452.219	300	40	18336.060	9804_5_ – $28140_{4}^{{}^{\circ}}$
5451.091	15	2	18339.854	21645_4_ – $39985_{3}^{{}^{\circ}}$
5450.511	25		18341.806	$15490_{0}^{{}^{\circ}}$ – 33831_4_
5450.146	5		18343.034	19273_2_ – $37616_{1}^{{}^{\circ}}$
5449.058	1		18346.697	12847_3_ – $31194_{4}^{{}^{\circ}}$
5448.272	50	3	18349.344	13847_2_ – $32197_{3}^{{}^{\circ}}$
5447.574	2		18351.695	15493_4_ – $33844_{0}^{{}^{\circ}}$
5447.417	2		18352.223	$17224_{2}^{{}^{\circ}}$ – 35576_3_
5447.071	1		18353.389	$22877_{0}^{{}^{\circ}}$ – 41230_4_
5446.894	10		18353.986	15970_3_ – $34324_{2}^{{}^{\circ}}$
5446.321	8		18355.917	$10526_{3}^{{}^{\circ}}$ – 28882_2_
5444.668	2		18361.489	20054_2_ – $38416_{3}^{{}^{\circ}}$
5444.477	5	1	18362.133	$13175_{4}^{{}^{\circ}}$ – 31537_3_
5444.421	1		18362.322	$22338_{3}^{{}^{\circ}}$ – 40701_4_
5443.481	1		18365.493	$19039_{2}^{{}^{\circ}}$ – 37404_3_
5441.213	75	8	18373.148	$18930_{3}^{{}^{\circ}}$ – 37303_4_
5441.042	25	1	18373.726	13297_4_ – $31671_{4}^{{}^{\circ}}$
5440.796	3		18374.556	15970_3_ – $34344_{4}^{{}^{\circ}}$
5440.600	50	2	18375.218	7502_3_ – $25877_{4}^{{}^{\circ}}$
5440.385	4		18375.944	19713_3_ – $38088_{2}^{{}^{\circ}}$
5440.116	4		18376.853	$18930_{3}^{{}^{\circ}}$ – 37307_3_
5439.495	2		18378.951	$22508_{2}^{{}^{\circ}}$ – 40886_2_
5438.250	10	1	18383.159	$17411_{3}^{{}^{\circ}}$ – 35794_4_
5437.958	3		18384.146	$20423_{1}^{{}^{\circ}}$ – 38807_2_
5437.232	10	1	18386.600	$20566_{4}^{{}^{\circ}}$ – 38953_5_
5436.814	2		18388.014	$20214_{3}^{{}^{\circ}}$ – 38602_2_
5436.326	2		18389.665	$19503_{3}^{{}^{\circ}}$ – 37892_4_
5436.146	1		18390.273	$20566_{4}^{{}^{\circ}}$ – 38956_4_
5434.152	100	10	18397.022	8111_4_ – $26508_{3}^{{}^{\circ}}$
5433.876	15	2	18397.956	$15166_{3}^{{}^{\circ}}$ – 33564_3_
5432.127	2		18403.879	$19588_{0}^{{}^{\circ}}$ – 37992_4_
5431.605	15	2	18405.648	11802_2_ – $30208_{2}^{{}^{\circ}}$
5431.112	150	40	18407.319	6362_2_ – $24769_{3}^{{}^{\circ}}$
5430.807	4		18408.353	$10526_{3}^{{}^{\circ}}$ – 28934_3_
5430.593	10		18409.078	$11877_{1}^{{}^{\circ}}$ – 30286_1_
5430.121	3		18410.678	21575_2_ – $39985_{3}^{{}^{\circ}}$
5429.105	100	5	18414.124	17398_3_ – $35812_{3}^{{}^{\circ}}$
5428.069	10		18417.638	19532_4_ – $37950_{4}^{{}^{\circ}}$
5427.767	15	1	18418.663	21143_5_ – $39562_{4}^{{}^{\circ}}$
5427.618	20	2	18419.168	13297_4_ – $31716_{0}^{{}^{\circ}}$
5427.352	2h		18420.071	$17411_{3}^{{}^{\circ}}$ – 35831_3_
5426.621	15b	1	18422.552	19532_4_ – $37954_{3}^{{}^{\circ}}$
5426.574	10b		18422.712	17959_4_ – $36382_{4}^{{}^{\circ}}$
5426.407	50	3	18423.279	7280_2_ – $25703_{2}^{{}^{\circ}}$
5426.170	50	2	18424.084	14226_0_ – $32650_{1}^{{}^{\circ}}$
5424.368	5		18430.204	$12114_{2}^{{}^{\circ}}$ – 30544_2_
5424.008	300	40	18431.427	$10414_{4}^{{}^{\circ}}$ – 28845_4_
5423.501	20b	3	18433.150	$16783_{4}^{{}^{\circ}}$ – 35216_5_
5422.914	15	1	18435.146	12847_3_ – $31283_{3}^{{}^{\circ}}$
5422.847	8b		18435.373	13088_3_ – $31523_{3}^{{}^{\circ}}$
5422.250	2		18437.403	13847_2_ – $32285_{3}^{{}^{\circ}}$
5421.966	5	1	18438.369	$20322_{0}^{{}^{\circ}}$ – 38760_4_
5421.664	100	5	18439.396	$15166_{3}^{{}^{\circ}}$ – 33606_2_
5420.451	4		18443.522	$20566_{4}^{{}^{\circ}}$ – 39010_3_
5419.463	1		18446.884	$18069_{3}^{{}^{\circ}}$ – 36515_3_
5418.702	3		18449.475	18699_2_ – $37149_{2}^{{}^{\circ}}$
5417.808	75	1	18452.520	11802_2_ – $30255_{3}^{{}^{\circ}}$
5417.486	400r	40	18453.616	3687_2_ – $22141_{3}^{{}^{\circ}}$
5415.775	4		18459.446	17073_1_ – $35533_{2}^{{}^{\circ}}$
5415.711	8		18459.664	18699_2_ – $37159_{1}^{{}^{\circ}}$
5415.558	75b		18460.186	15863_2_ – $34324_{2}^{{}^{\circ}}$
5415.528	200b	2	18460.288	$7795_{4}^{{}^{\circ}}$ – 26255_4_
5414.920	5	1	18462.361	$18053_{4}^{{}^{\circ}}$ – 36515_3_
5414.920	5	1	18462.361	$21738_{2}^{{}^{\circ}}$ – 40200_3_
5413.917	10	1	18465.781	8800_4_ – $27266_{4}^{{}^{\circ}}$
5411.406	3		18474.350	$18930_{3}^{{}^{\circ}}$ – 37404_3_
5411.311	5		18474.674	15493_4_ – $33967_{3}^{{}^{\circ}}$
5410.769	400	15	18476.525	6362_2_ – $24838_{1}^{{}^{\circ}}$
5410.313	15b		18478.082	11802_2_ – $30281_{1}^{{}^{\circ}}$
5409.603	2		18480.507	$18614_{1}^{{}^{\circ}}$ – 37094_2_
5409.304	4		18481.528	$14481_{6}^{{}^{\circ}}$ – 32963_5_
5408.749	20	2h	18483.425	13297_4_ – $31780_{3}^{{}^{\circ}}$
5408.192	2		18485.328	$20214_{3}^{{}^{\circ}}$ – 38700_4_
5407.652	400b	40b	18487.175	$11197_{0}^{{}^{\circ}}$ – 29684_5_
5407.567	75b	10b	18487.465	$20522_{2}^{{}^{\circ}}$ – 39010_3_
5406.755	50	4	18490.241	$16217_{2}^{{}^{\circ}}$ – 34707_3_
5405.787	20	1	18493.552	$18809_{4}^{{}^{\circ}}$ – 37303_4_
5404.708	15		18497.244	$18809_{4}^{{}^{\circ}}$ – 37307_3_
5403.200	200	15	18502.407	$16346_{4}^{{}^{\circ}}$ – 34849_5_
5401.582	8b	1u	18507.949	15863_2_ – $34371_{2}^{{}^{\circ}}$
5401.536	10b	1	18508.107	15493_4_ – $34001_{4}^{{}^{\circ}}$
5400.775	8		18510.714	13088_3_ – $31599_{2}^{{}^{\circ}}$
5400.145	150	5	18512.874	$13945_{3}^{{}^{\circ}}$ – 32458_4_
5399.620	50	100	18514.674	$11241_{3}^{{}^{\circ}}$ – 29756_4_
5399.427	8h		18515.336	$21165_{3}^{{}^{\circ}}$ – 39680_2_
5398.920	400	40	18517.075	$16783_{4}^{{}^{\circ}}$ – 35300_5_
5398.704	150	4	18517.815	6362_2_ – $24880_{1}^{{}^{\circ}}$
5398.510	8	1	18518.481	$19588_{0}^{{}^{\circ}}$ – 38106_5_
5398.205	150	4	18519.527	$14032_{2}^{{}^{\circ}}$ – 32551_3_
5397.890	20	1	18520.608	19532_4_ – $38053_{3}^{{}^{\circ}}$
5397.731	1		18521.153	21575_2_ – $40096_{2}^{{}^{\circ}}$
5397.499	2		18521.949	15970_3_ – $34492_{4}^{{}^{\circ}}$
5397.445	5		18522.135	$15166_{3}^{{}^{\circ}}$ – 33689_2_
5397.139	201		18523.185	$17354_{1}^{{}^{\circ}}$ – 35877_2_
5396.109	50	4	18526.720	$16346_{4}^{{}^{\circ}}$ – 34873_4_
5396.109	50	4	18526.720	16351_0_ – $34878_{1}^{{}^{\circ}}$
5394.761	300	20	18531.350	3865_1_ – $22396_{1}^{{}^{\circ}}$
5393.972	150	8	18534.060	7502_3_ – $26036_{3}^{{}^{\circ}}$
5393.873	50b	3	18534.401	8111_4_ – $26645_{0}^{{}^{\circ}}$
5393.873	50b	3	18534.401	18699_2_ – $37234_{2}^{{}^{\circ}}$
5392.768	5		18538.198	$22163_{4}^{{}^{\circ}}$ – 40701_4_
5390.575	2		18545.740	$20214_{3}^{{}^{\circ}}$ – 38760_4_
5390.429	300	75	18546.242	7502_3_ – $26048_{4}^{{}^{\circ}}$
5388.057	300	20	18554.407	$15166_{3}^{{}^{\circ}}$ – 33721_2_
5386.611	500	20	18559.388	4961_4_ – $23521_{3}^{{}^{\circ}}$
5386.611	500	20	18559.388	$11197_{0}^{{}^{\circ}}$ – 29756_4_
5386.149	2b		18560.980	3687_2_ – $22248_{2}^{{}^{\circ}}$
5386.111	201		18561.110	$15736_{1}^{{}^{\circ}}$ – 34298_1_
5384.301	100	15	18567.350	17166_5_ – $35733_{4}^{{}^{\circ}}$
5382.175	15b	1u	18574.684	$14206_{4}^{{}^{\circ}}$ – 32781_4_
5381.368	25b	1	18577.470	$10526_{3}^{{}^{\circ}}$ – 29104_4_
5379.884	8	1	18582.594	13088_3_ – $31671_{4}^{{}^{\circ}}$
5379.325	8b		18584.525	$18069_{3}^{{}^{\circ}}$ – 36653_2_
5379.285	10b	1	18584.663	18574_1_ – $37159_{1}^{{}^{\circ}}$
5379.112	300	20	18585.261	$7795_{4}^{{}^{\circ}}$ – 26380_5_
5378.837	400	40	18586.211	$14481_{6}^{{}^{\circ}}$ – 33068_6_
5378.169	50	4	18588.520	$17501_{0}^{{}^{\circ}}$ – 36089_4_
5377.970	10	1	18589.207	$14206_{4}^{{}^{\circ}}$ – 32796_3_
5376.778	200	50	18593.328	$11241_{3}^{{}^{\circ}}$ – 29835_3_
5376.600	4		18593.944	$19503_{3}^{{}^{\circ}}$ – 38097_2_
5376.366	2b		18594.753	$18809_{4}^{{}^{\circ}}$ – 37404_3_
5375.497	20		18597.759	$19588_{0}^{{}^{\circ}}$ – 38186_4_
5374.998	50	8h	18599.486	$13175_{4}^{{}^{\circ}}$ – 31774_3_
5373.394	40	2	18605.038	17073_1_ – $35678_{1}^{{}^{\circ}}$
5372.703	200	25	18607.431	$11241_{3}^{{}^{\circ}}$ – 29849_4_
5372.230	1		18609.069	21645_4_ – $40254_{3}^{{}^{\circ}}$
5372.117	3		18609.460	18431_3_ – $37041_{4}^{{}^{\circ}}$
5371.999	8		18609.869	18549_2_ – $37159_{1}^{{}^{\circ}}$
5371.678	3		18610.981	7502_3_ – $26113_{2}^{{}^{\circ}}$
5371.124	50	1	18612.901	13962_1_ – $32575_{2}^{{}^{\circ}}$
5371.124	50	1	18612.901	15970_3_ – $34583_{3}^{{}^{\circ}}$
5370.709	150	2	18614.339	0_2_ – $18614_{1}^{{}^{\circ}}$
5370.522	5		18614.987	20054_2_ – $38669_{2}^{{}^{\circ}}$
5369.890	10	1	18617.178	$21077_{0}^{{}^{\circ}}$ – 39694_5_
5369.447	200	5	18618.714	6362_2_ – $24981_{3}^{{}^{\circ}}$
5369.284	200	8	18619.279	5563_1_ – $24182_{2}^{{}^{\circ}}$
5368.902	5		18620.604	20054_2_ – $38675_{3}^{{}^{\circ}}$
5368.654	10	1	18621.464	$16783_{4}^{{}^{\circ}}$ – 35405_3_
5367.849	3		18624.257	21645_4_ – $40270_{4}^{{}^{\circ}}$
5367.234	51		18626.391	22098_4_ – $40724_{0}^{{}^{\circ}}$
5366.557	2		18628.740	$19516_{2}^{{}^{\circ}}$ – 38145_3_
5365.913	2		18630.976	$20322_{0}^{{}^{\circ}}$ – 38953_5_
5365.520	50b	3b	18632.341	$17501_{0}^{{}^{\circ}}$ – 36133_5_
5364.985	4	1	18634.199	$14465_{2}^{{}^{\circ}}$ – 33099_3_
5364.682	8b		18635.251	$10783_{2}^{{}^{\circ}}$ – 29418_2_
5364.447	15	5	18636.067	$17411_{3}^{{}^{\circ}}$ – 36047_2_
5363.931	5		18637.860	16351_0_ – $34989_{1}^{{}^{\circ}}$
5363.880	5		18638.037	$22248_{2}^{{}^{\circ}}$ – 40886_2_
5362.576	200	5	18642.570	3865_1_ – $22508_{2}^{{}^{\circ}}$
5361.156	75	3	18647.507	4961_4_ – $23609_{0}^{{}^{\circ}}$
5360.697	8		18649.104	$19039_{2}^{{}^{\circ}}$ – 37688_3_
5360.150	300	5	18651.007	3687_2_ – $22338_{3}^{{}^{\circ}}$
5359.827	100	4	18652.131	$11197_{0}^{{}^{\circ}}$ – 29849_4_
5359.432	5		18653.506	$17224_{2}^{{}^{\circ}}$ – 35877_2_
5358.713	300	15	18656.009	13297_4_ – $31953_{4}^{{}^{\circ}}$
5358.558	2		18656.548	$16217_{2}^{{}^{\circ}}$ – 34874_3_
5358.415	3		18657.046	$16783_{4}^{{}^{\circ}}$ – 35440_3_
5358.081	5		18658.209	14204_5_ – $32862_{4}^{{}^{\circ}}$
5357.743	8	1	18659.386	18574_1_ – $37234_{2}^{{}^{\circ}}$
5357.338	2		18660.797	$21539_{4}^{{}^{\circ}}$ – 40200_3_
5356.672	10		18663.117	$19227_{6}^{{}^{\circ}}$ – 37890_5_
5356.278	1		18664.490	17398_3_ – $36062_{4}^{{}^{\circ}}$
5356.137	10		18664.981	$15166_{3}^{{}^{\circ}}$ – 33831_4_
5355.638	100	3	18666.720	$11877_{1}^{{}^{\circ}}$ – 30544_2_
5354.602	40	1	18670.332	2869_3_ – $21539_{4}^{{}^{\circ}}$
5353.977	5h		18672.511	$21165_{3}^{{}^{\circ}}$ – 39837_3_
5353.154	2		18675.382	21594_3_ – $40270_{4}^{{}^{\circ}}$
5353.154	2		18675.382	9804_5_ – $28480_{4}^{{}^{\circ}}$
5353.073	50	4	18675.664	17398_3_ – $36074_{3}^{{}^{\circ}}$
5352.992	50b	15	18675.947	12847_3_ – $31523_{3}^{{}^{\circ}}$
5352.304	3b		18678.347	22098_4_ – $40776_{4}^{{}^{\circ}}$
5352.272	5b		18678.459	$17411_{3}^{{}^{\circ}}$ – 36089_4_
5351.996	10		18679.423	8111_4_ – $26790_{4}^{{}^{\circ}}$
5351.839	150	8	18679.970	11601_1_ – $30281_{1}^{{}^{\circ}}$
5351.126	200	25	18682.459	$15618_{3}^{{}^{\circ}}$ – 34301_4_
5350.538	50	4	18684.512	19532_4_ – $38216_{3}^{{}^{\circ}}$
5350.206	50	1	18685.672	$20566_{4}^{{}^{\circ}}$ – 39252_5_
5349.425	3		18688.400	13962_1_ – $32650_{1}^{{}^{\circ}}$
5349.003	75	3	18689.874	$10414_{4}^{{}^{\circ}}$ – 29104_4_
5348.532	4	1	18691.520	$22669_{3}^{{}^{\circ}}$ – 41361_3_
5348.310	50	1	18692.296	13088_3_ – $31780_{3}^{{}^{\circ}}$
5347.973	150	5	18693.474	4961_4_ – $23655_{4}^{{}^{\circ}}$
5346.766	50	2	18697.694	$23015_{0}^{{}^{\circ}}$ – 41712_5_
5345.241	5		18703.028	13962_1_ – $32665_{1}^{{}^{\circ}}$
5344.308	3		18706.293	$18069_{3}^{{}^{\circ}}$ – 36775_3_
5343.581	500r	40	18708.838	3687_2_ – $22396_{1}^{{}^{\circ}}$
5343.361	50b		18709.608	$17501_{0}^{{}^{\circ}}$ – 36210_5_
5341.206	75	3	18717.157	$20566_{4}^{{}^{\circ}}$ – 39283_4_
5340.650	201		18719.106	15863_2_ – $34583_{3}^{{}^{\circ}}$
5340.501	200	10	18719.628	16554_6_ – $35273_{0}^{{}^{\circ}}$
5339.896	50	5	18721.749	$18053_{4}^{{}^{\circ}}$ – 36775_3_
5338.641	40	1	18726.150	13297_4_ – $32023_{0}^{{}^{\circ}}$
5338.366	40	15	18727.114	15863_2_ – $34591_{1}^{{}^{\circ}}$
5338.285	25b	1	18727.399	$10414_{4}^{{}^{\circ}}$ – 29141_5_
5338.215	50b	1	18727.644	13847_2_ – $32575_{2}^{{}^{\circ}}$
5337.502	2		18730.146	21143_5_ – $39873_{0}^{{}^{\circ}}$
5336.785	50	1	18732.662	$14481_{6}^{{}^{\circ}}$ – 33214_5_
5336.315	40	1	18734.312	15970_3_ – $34704_{3}^{{}^{\circ}}$
5335.373	10	3	18737.620	$18011_{0}^{{}^{\circ}}$ – 36749_6_
5334.145	751	1	18741.933	$20214_{3}^{{}^{\circ}}$ – 38956_4_
5333.386	75b	1	18744.601	5563_1_ – $24307_{2}^{{}^{\circ}}$
5333.200	8b		18745.254	$19039_{2}^{{}^{\circ}}$ – 37784_2_
5332.120	2		18749.051	$18069_{3}^{{}^{\circ}}$ – 36818_2_
5331.749	50	2	18750.356	11802_2_ – $30553_{2}^{{}^{\circ}}$
5331.481	8		18751.298	12847_3_ – $31599_{2}^{{}^{\circ}}$
5330.517	8		18754.689	$18053_{4}^{{}^{\circ}}$ – 36808_3_
5330.340	51		18755.312	15970_3_ – $34725_{3}^{{}^{\circ}}$
5330.082	200b	5	18756.220	7280_2_ – $26036_{3}^{{}^{\circ}}$
5330.016	50b		18756.452	$14206_{4}^{{}^{\circ}}$ – 32963_5_
5329.605	2		18757.899	$18930_{3}^{{}^{\circ}}$ – 37688_3_
5328.009	8		18763.517	21143_5_ – $39906_{4}^{{}^{\circ}}$
5327.862	10		18764.035	$14032_{2}^{{}^{\circ}}$ – 32796_3_
5326.976	500r	40	18767.156	8111_4_ – $26878_{3}^{{}^{\circ}}$
5326.278	100b	8	18769.615	$11241_{3}^{{}^{\circ}}$ – 30011_3_
5326.192	50	2	18769.918	$18011_{0}^{{}^{\circ}}$ – 36781_5_
5325.434	100	2	18772.590	$11241_{3}^{{}^{\circ}}$ – 30014_4_
5324.591	10		18775.562	$12114_{2}^{{}^{\circ}}$ – 30889_1_
5323.851	151	3	18778.172	$19588_{0}^{{}^{\circ}}$ – 38366_4_
5323.527	8	1	18779.315	21645_4_ – $40425_{4}^{{}^{\circ}}$
5323.406	150	3	18779.741	19273_2_ – $38053_{3}^{{}^{\circ}}$
5322.901	200	5	18781.523	13088_3_ – $31870_{2}^{{}^{\circ}}$
5321.873	2		18785.151	$20566_{4}^{{}^{\circ}}$ – 39351_5_
5321.691	3		18785.793	$19817_{1}^{{}^{\circ}}$ – 38602_2_
5319.742	8		18792.676	$16783_{4}^{{}^{\circ}}$ – 35576_3_
5319.023	2		18795.216	$20214_{3}^{{}^{\circ}}$ – 39010_3_
5318.110	75	3	18798.443	$16783_{4}^{{}^{\circ}}$ – 35582_5_
5317.912	8		18799.143	22401_1_ – $41200_{2}^{{}^{\circ}}$
5317.494	200	4	18800.620	$8243_{2}^{{}^{\circ}}$ – 27044_3_
5316.783	15		18803.135	13847_2_ – $32650_{1}^{{}^{\circ}}$
5315.834	5		18806.491	$17847_{2}^{{}^{\circ}}$ – 36653_2_
5315.230	150		18808.629	$14465_{2}^{{}^{\circ}}$ – 33273_1_
5314.251	8b	1	18812.093	17073_1_ – $35885_{2}^{{}^{\circ}}$
5314.205	10b	2	18812.256	$19948_{4}^{{}^{\circ}}$ – 38760_4_
5314.178	10b		18812.352	$15618_{3}^{{}^{\circ}}$ – 34431_3_
5313.233	5h		18815.698	19273_2_ – $38088_{2}^{{}^{\circ}}$
5312.904	200	15	18816.863	7280_2_ – $26096_{3}^{{}^{\circ}}$
5312.781	50	3	18817.298	$11197_{0}^{{}^{\circ}}$ – 30014_4_
5312.643	150	15	18817.787	13847_2_ – $32665_{1}^{{}^{\circ}}$
5312.532	400	40	18818.180	5563_1_ – $24381_{2}^{{}^{\circ}}$
5312.001	400r	25	18820.062	3687_2_ – $22508_{2}^{{}^{\circ}}$
5311.870	75b	2	18820.526	$13175_{4}^{{}^{\circ}}$ – 31995_4_
5311.174	20	1	18822.992	$17224_{2}^{{}^{\circ}}$ – 36047_2_
5310.432	5		18825.622	$15618_{3}^{{}^{\circ}}$ – 34444_2_
5309.616	150	5	18828.515	$16217_{2}^{{}^{\circ}}$ – 35046_1_
5309.052	2h		18830.515	21594_3_ – $40425_{4}^{{}^{\circ}}$
5308.808	10	1	18831.381	$22855_{3}^{{}^{\circ}}$ – 41686_3_
5308.726	10	1	18831.672	$18053_{4}^{{}^{\circ}}$ – 36885_4_
5308.310	100	10	18833.147	7280_2_ – $26113_{2}^{{}^{\circ}}$
5306.987	75	4	18837.842	$13175_{4}^{{}^{\circ}}$ – 32012_4_
5306.166	5		18840.757	17959_4_ – $36800_{4}^{{}^{\circ}}$
5305.304	51		18843.818	$14465_{2}^{{}^{\circ}}$ – 33309_2_
5303.557	20	1	18850.025	$16783_{4}^{{}^{\circ}}$ – 35633_4_
5303.483	75	3	18850.288	$12114_{2}^{{}^{\circ}}$ – 30964_3_
5303.334	50	2	18850.818	$13945_{3}^{{}^{\circ}}$ – 32796_3_
5303.162	40	1	18851.429	15493_4_ – $34344_{4}^{{}^{\circ}}$
5302.779	1		18852.791	19532_4_ – $38385_{0}^{{}^{\circ}}$
5302.407	10		18854.114	$18930_{3}^{{}^{\circ}}$ – 37784_2_
5300.524	300	5	18860.811	7502_3_ – $26363_{2}^{{}^{\circ}}$
5300.321	4		18861.534	15863_2_ – $34725_{3}^{{}^{\circ}}$
5299.787	2		18863.434	$19503_{3}^{{}^{\circ}}$ – 38366_4_
5299.376	2		18864.897	13088_3_ – $31953_{4}^{{}^{\circ}}$
5298.714	8	2	18867.254	19713_3_ – $38580_{4}^{{}^{\circ}}$
5298.559	75	2	18867.806	$10783_{2}^{{}^{\circ}}$ – 29650_2_
5298.284	200	4	18868.785	2869_3_ – $21738_{2}^{{}^{\circ}}$
5297.844	100b	5	18870.352	$16346_{4}^{{}^{\circ}}$ – 35216_5_
5297.746	300b	15	18870.701	8800_4_ – $27670_{3}^{{}^{\circ}}$
5296.845	50	2	18873.911	$18011_{0}^{{}^{\circ}}$ – 36885_4_
5296.279	300	5	18875.928	9804_5_ – $28680_{4}^{{}^{\circ}}$
5295.674	50b	4b	18878.085	17959_4_ – $36837_{0}^{{}^{\circ}}$
5295.089	50	1	18880.170	$11877_{1}^{{}^{\circ}}$ – 30758_2_
5294.697	25b	1	18881.568	15970_3_ – $34851_{2}^{{}^{\circ}}$
5294.397	200	3	18882.638	7502_3_ – $26384_{4}^{{}^{\circ}}$
5294.070	50	1	18883.804	19532_4_ – $38416_{3}^{{}^{\circ}}$
5293.435	3	1	18886.070	$17411_{3}^{{}^{\circ}}$ – 36297_4_
5293.137	50	1	18887.133	$21539_{4}^{{}^{\circ}}$ – 40426_4_
5293.077	3		18887.347	$21165_{3}^{{}^{\circ}}$ – 40052_4_
5292.666	3		18888.814	$18930_{3}^{{}^{\circ}}$ – 37819_4_
5292.077	10		18890.916	$11241_{3}^{{}^{\circ}}$ – 30132_2_
5291.818	200	5	18891.840	$10526_{3}^{{}^{\circ}}$ – 29418_2_
5291.638	3		18892.483	$14206_{4}^{{}^{\circ}}$ – 33099_3_
5289.624	75	2	18899.676	13297__4_ – _ $32197_{3}^{{}^{\circ}}$
5287.648	3		18906.739	13847_2_ – $32754_{3}^{{}^{\circ}}$
5285.183	3h		18915.557	$20922_{2}^{{}^{\circ}}$ – 39837_3_
5283.697	50	2	18920.877	11802_2_ – $30723_{1}^{{}^{\circ}}$
5282.552	15		18924.978	$17411_{3}^{{}^{\circ}}$ – 36336_3_
5282.402	200	4	18925.515	$14465_{2}^{{}^{\circ}}$ – 33390_1_
5281.070	300	2	18930.288	0__2_ – _ $18930_{3}^{{}^{\circ}}$
5280.520	50		18932.260	$16217_{2}^{{}^{\circ}}$ – 35149_2_
5280.346	50	2	18932.884	12847_3_ – $31780_{3}^{{}^{\circ}}$
5279.766	2		18934.964	14226_0_ – $33161_{1}^{{}^{\circ}}$
5279.200	50	1	18936.994	15493_4_ – $34430_{3}^{{}^{\circ}}$
5277.404	10		18943.438	$14465_{2}^{{}^{\circ}}$ – 33408_3_
5277.341	3		18943.665	19273_2_ – $38216_{3}^{{}^{\circ}}$
5277.147	75	1	18944.361	6362_2_ – $25306_{2}^{{}^{\circ}}$
5276.198	2		18947.768	$21252_{2}^{{}^{\circ}}$ – 40200_3_
5274.948	8	1	18952.258	11601_1_ – $30553_{2}^{{}^{\circ}}$
5274.581	5		18953.577	$20566_{4}^{{}^{\circ}}$ – 39520_4_
5274.391	75	3	18954.260	$16346_{4}^{{}^{\circ}}$ – 35300_5_
5274.121	200	5	18955.230	4961_4_ – $23916_{4}^{{}^{\circ}}$
5273.595	2		18957.121	$15490_{0}^{{}^{\circ}}$ – 34447_4_
5273.531	40		18957.351	18699_2_ – $37656_{3}^{{}^{\circ}}$
5273.134	100b	10	18958.778	11802_2_ – $30761_{3}^{{}^{\circ}}$
5272.929	100	20	18959.515	6362_2_ – $25321_{3}^{{}^{\circ}}$
5272.092	5		18962.525	$18930_{3}^{{}^{\circ}}$ – 37892_4_
5271.023	20	3	18966.371	17166_5_ – $36132_{0}^{{}^{\circ}}$
5270.832	50	2	18967.058	$19986_{6}^{{}^{\circ}}$ – 38953_5_
5269.794	75	4	18970.794	$15490_{0}^{{}^{\circ}}$ – 34460_5_
5268.539	2		18975.313	$21077_{0}^{{}^{\circ}}$ – 40052_4_
5268.158	2		18976.685	16351_0_ – $35328_{1}^{{}^{\circ}}$
5266.829	2		18981.474	$12114_{2}^{{}^{\circ}}$ – 31095_1_
5266.710	300	15	18981.902	3687_2_ – $22669_{3}^{{}^{\circ}}$
5266.073	1		18984.199	17398_3_ – $36382_{4}^{{}^{\circ}}$
5265.081	10		18987.775	15863_2_ – $34851_{2}^{{}^{\circ}}$
5264.335	20		18990.466	$19817_{1}^{{}^{\circ}}$ – 38807_2_
5263.892	15b	1	18992.064	13962_1_ – $32954_{2}^{{}^{\circ}}$
5262.621	50b	4	18996.651	13297_4_ – $32294_{4}^{{}^{\circ}}$
5262.024	5	1	18998.806	15493_4_ – $34492_{4}^{{}^{\circ}}$
5261.530	50b	1	19000.590	$15166_{3}^{{}^{\circ}}$ – 34167_3_
5261.474	50b	3	19000.792	$7795_{4}^{{}^{\circ}}$ – 26796_3_
5260.106	200	8	19005.734	7502_3_ – $26508_{3}^{{}^{\circ}}$
5259.779	75	2	19006.915	7280_2_ – $26287_{1}^{{}^{\circ}}$
5259.587	20	1	19007.609	$14206_{4}^{{}^{\circ}}$ – 33214_5_
5259.353	3		19008.455	$19948_{4}^{{}^{\circ}}$ – 38956_4_
5258.908	8		19010.063	11802_2_ – $30812_{2}^{{}^{\circ}}$
5258.779	5		19010.530	$16783_{4}^{{}^{\circ}}$ – 35794_4_
5258.361	400r	25	19012.041	3865_1_ – $22877_{1}^{{}^{\circ}}$
5258.212	150b	3	19012.580	11601_1_ – $30613_{0}^{{}^{\circ}}$
5257.607	4		19014.767	15863_2_ – $34878_{1}^{{}^{\circ}}$
5257.051	10	1	19016.778	$18069_{3}^{{}^{\circ}}$ – 37085_4_
5255.921	10b		19020.867	2869_3_ – $21890_{3}^{{}^{\circ}}$
5255.576	100	3	19022.115	12847_3_ – $31870_{2}^{{}^{\circ}}$
5254.262	1501	8	19026.872	$13175_{4}^{{}^{\circ}}$ – 32202_5_
5254.111	40	4	19027.419	$12114_{2}^{{}^{\circ}}$ – 31141_3_
5253.682	50	2	19028.973	17398_3_ – $36427_{3}^{{}^{\circ}}$
5253.534	8		19029.509	$20322_{0}^{{}^{\circ}}$ – 39351_5_
5252.568	15		19033.009	2869_3_ – $21902_{4}^{{}^{\circ}}$
5251.373	501	2	19037.340	18699_2_ – $37736_{2}^{{}^{\circ}}$
5250.874	50	1	19039.149	0_2_ – $19039_{2}^{{}^{\circ}}$
5250.171	10		19041.698	18574_1_ – $37616_{1}^{{}^{\circ}}$
5248.587	10		19047.445	$16783_{4}^{{}^{\circ}}$ – 35831_3_
5248.199	20		19048.853	17959_4_ – $37008_{0}^{{}^{\circ}}$
5247.361	40		19051.895	$10783_{2}^{{}^{\circ}}$ – 29835_3_
5247.200	50b	2	19052.480	8800_4_ – $27852_{0}^{{}^{\circ}}$
5245.698	10		19057.935	$19039_{2}^{{}^{\circ}}$ – 38097_2_
5244.647	8b	1	19061.754	$19948_{4}^{{}^{\circ}}$ – 39010_3_
5244.594	10b	1	19061.947	$18069_{3}^{{}^{\circ}}$ – 37131_3_
5244.594	10b	1	19061.947	$18930_{3}^{{}^{\circ}}$ – 37992_4_
5243.746	50	1	19065.029	$14243_{1}^{{}^{\circ}}$ – 33309_2_
5243.406	8		19066.265	14204_5_ – $33270_{4}^{{}^{\circ}}$
5243.237	3		19066.880	18549_2_ – $37616_{1}^{{}^{\circ}}$
5243.120	3		19067.305	$14032_{2}^{{}^{\circ}}$ – 33099_3_
5242.687	20		19068.880	$20214_{3}^{{}^{\circ}}$ – 39283_4_
5241.858	50	20	19071.896	13088_3_ – $32160_{2}^{{}^{\circ}}$
5241.223	8	1	19074.206	$17411_{3}^{{}^{\circ}}$ – 36485_2_
5241.153	8	1	19074.461	$18011_{0}^{{}^{\circ}}$ – 37085_4_
5240.789	3		19075.786	19713_3_ – $38788_{3}^{{}^{\circ}}$
5240.789	3		19075.786	19713_3_ – $38788_{3}^{{}^{\circ}}$
5240.341	2		19077.417	$18053_{4}^{{}^{\circ}}$ – 37131_3_
5239.984	2		19078.717	21645_4_ – $40724_{0}^{{}^{\circ}}$
5239.550	50	3	19080.297	6362_2_ – $25442_{3}^{{}^{\circ}}$
5239.079	2		19082.012	19273_2_ – $38355_{2}^{{}^{\circ}}$
5238.812	75h	10	19082.985	7280_2_ – $26363_{2}^{{}^{\circ}}$
5237.989	3		19085.983	$19516_{2}^{{}^{\circ}}$ – 38602_2_
5237.229	5	1	19088.753	$15618_{3}^{{}^{\circ}}$ – 34707_3_
5236.766	10	1	19090.440	17398_3_ – $36488_{2}^{{}^{\circ}}$
5235.720	4	1	19094.254	$16346_{4}^{{}^{\circ}}$ – 35440_3_
5234.243	75		19099.642	$14465_{2}^{{}^{\circ}}$ – 33564_3_
5234.107	100	1	19100.138	$8243_{2}^{{}^{\circ}}$ – 27343_3_
5232.841	8		19104.759	$17411_{3}^{{}^{\circ}}$ – 36515_3_
5232.341	40b	2	19106.585	$19039_{2}^{{}^{\circ}}$ – 38145_3_
5232.278	25b	1	19106.815	13847_2_ – $32954_{2}^{{}^{\circ}}$
5231.800	40	1	19108.561	13088_3_ – $32197_{3}^{{}^{\circ}}$
5231.659	3		19109.076	17166_5_ – $36275_{0}^{{}^{\circ}}$
5231.160	1000r	40	19110.899	2558_0_ – $21668_{1}^{{}^{\circ}}$
5230.012	15		19115.093	17073_1_ – $36188_{2}^{{}^{\circ}}$
5228.996	75	1	19118.807	$13175_{4}^{{}^{\circ}}$ – 32293_5_
5227.904	8		19122.801	11601_1_ – $30723_{1}^{{}^{\circ}}$
5227.461	8		19124.421	$10526_{3}^{{}^{\circ}}$ – 29650_2_
5227.078	40		19125.823	11802_2_ – $30928_{1}^{{}^{\circ}}$
5226.527	50		19127.839	9804_5_ – $28932_{4}^{{}^{\circ}}$
5225.755	5	1	19130.665	21645_4_ – $40776_{4}^{{}^{\circ}}$
5225.518	4		19131.532	$12114_{2}^{{}^{\circ}}$ – 31245_2_
5224.928	3		19133.693	$18614_{1}^{{}^{\circ}}$ – 37748_2_
5224.693	5		19134.553	$15166_{3}^{{}^{\circ}}$ – 34301_4_
5222.909	2		19141.089	$14465_{2}^{{}^{\circ}}$ – 33606_2_
5222.765	40	1	19141.617	13297_4_ – $32439_{4}^{{}^{\circ}}$
5222.268	50	1	19143.438	$142478_{0}^{{}^{\circ}}$ – 33390_1_
5220.928	100	1	19148.352	8800_4_ – $27948_{4}^{{}^{\circ}}$
5220.707	100	1	19149.162	8111_4_ – $27260_{3}^{{}^{\circ}}$
5220.131	4		19151.275	$12114_{2}^{{}^{\circ}}$ – 31265_3_
5219.357	20	1	19154.115	$13945_{3}^{{}^{\circ}}$ – 33099_3_
5219.110	400r	15	19155.022	8111_4_ – $27266_{4}^{{}^{\circ}}$
5218.272	2		19158.098	$18053_{4}^{{}^{\circ}}$ – 37211_5_
5217.284	8h		19161.726	$21539_{4}^{{}^{\circ}}$ – 40701_4_
5217.109	2		19162.368	18574_1_ – $37736_{2}^{{}^{\circ}}$
5216.683	20		19163.933	6362_2_ – $25526_{1}^{{}^{\circ}}$
5215.762	5		19167.317	3687_2_ – $22855_{3}^{{}^{\circ}}$
5214.414	4	1	19172.272	$19588_{0}^{{}^{\circ}}$ – 38760_4_
5213.930	2		19174.052	18431_3_ – $37605_{4}^{{}^{\circ}}$
5213.485	75	2	19175.688	$11197_{0}^{{}^{\circ}}$ – 30372_6_
5213.350	400	5	19176.185	$7795_{4}^{{}^{\circ}}$ – 26971_4_
5212.681	15	1	19178.646	$10783_{2}^{{}^{\circ}}$ – 29961_1_
5211.668	4		19182.374	$18809_{4}^{{}^{\circ}}$ – 37992_4_
5211.231	600r	25	19183.982	3865_1_ – $23049_{1}^{{}^{\circ}}$
5210.253	10	1	19187.583	11802_2_ – $30990_{3}^{{}^{\circ}}$
5210.185	8	1	19187.834	$16217_{2}^{{}^{\circ}}$ – 35405_3_
5209.724	300	3	19189.531	3687_2_ – $22877_{1}^{{}^{\circ}}$
5208.087	8		19195.563	13847_2_ – $33043_{3}^{{}^{\circ}}$
5207.803	100	2	19196.610	13088_3_ – $32285_{3}^{{}^{\circ}}$
5207.670	20	1	19197.100	$19503_{3}^{{}^{\circ}}$ – 38700_4_
5207.084	50	1	19199.261	13962_1_ – $33161_{1}^{{}^{\circ}}$
5206.990	3		19199.607	17959_4_ – $37159_{3}^{{}^{\circ}}$
5206.800	50	3	19200.308	$18011_{0}^{{}^{\circ}}$ – 37211_5_
5206.410	5		19201.746	$14206_{4}^{{}^{\circ}}$ – 33408_3_
5205.928	15	2	19203.524	$16217_{2}^{{}^{\circ}}$ – 35421_1_
5205.154	200	2	19206.379	8111_4_ – $27317_{3}^{{}^{\circ}}$
5203.850	300	25	19211.192	$14206_{4}^{{}^{\circ}}$ – 33418_4_
5203.850	300	25	19211.192	15493_4_ – $34704_{3}^{{}^{\circ}}$
5203.641	5		19211.964	11601_1_ – $30812_{2}^{{}^{\circ}}$
5202.410	5		19216.510	17166_5_ – $36382_{4}^{{}^{\circ}}$
5202.007	75	3	19217.998	$11877_{1}^{{}^{\circ}}$ – 31095_1_
5199.323	300b	3	19227.919	7280_2_ – $26508_{3}^{{}^{\circ}}$
5199.164	1000r	25b	19228.507	3865_1_ – $23093_{2}^{{}^{\circ}}$
5198.802	500	100d	19229.846	$10526_{3}^{{}^{\circ}}$ – 29756_4_
5198.162	4		19232.213	15493_4_ – $34725_{3}^{{}^{\circ}}$
5197.577	8		19234.378	$18069_{3}^{{}^{\circ}}$ – 37303_4_
5197.236	200	5	19235.640	$16346_{4}^{{}^{\circ}}$ – 35582_5_
5196.574	5		19238.090	$18069_{3}^{{}^{\circ}}$ – 37307_3_
5195.815	500	8	19240.900	4961_4_ – $24202_{4}^{{}^{\circ}}$
5195.581	15	4	19241.767	$14032_{2}^{{}^{\circ}}$ – 33273_1_
5194.456	400	12	19245.934	9804_5_ – $29050_{0}^{{}^{\circ}}$
5193.946	75	2	19247.824	$17501_{0}^{{}^{\circ}}$ – 36749_6_
5193.636	8b		19248.973	$7795_{4}^{{}^{\circ}}$ – 27044_3_
5193.406	15	1	19249.825	$18053_{4}^{{}^{\circ}}$ – 37303_4_
5192.746	50	1	19252.272	$14206_{4}^{{}^{\circ}}$ – 33459_4_
5192.409	4	1	19253.522	$18053_{4}^{{}^{\circ}}$ – 37307_3_
5192.176	5		19254.385	$15618_{3}^{{}^{\circ}}$ – 34873_4_
5191.986	20	1	19255.090	$15618_{3}^{{}^{\circ}}$ – 34874_3_
5191.919	2		19255.339	18699_2_ – $37954_{3}^{{}^{\circ}}$
5191.715	4	1	19256.095	$14465_{2}^{{}^{\circ}}$ – 33721_2_
5191.487	3		19256.941	$20423_{1}^{{}^{\circ}}$ – 39680_2_
5191.337	15		19257.497	$19503_{3}^{{}^{\circ}}$ – 38760_4_
5190.368	8		19261.093	$17224_{2}^{{}^{\circ}}$ – 36485_2_
5190.219	5		19261.645	$21165_{3}^{{}^{\circ}}$ – 40426_4_
5189.464	3		19264.448	$15166_{3}^{{}^{\circ}}$ – 34431_3_
5189.008	50	1	19266.141	$19986_{6}^{{}^{\circ}}$ – 39252_5_
5187.948	40		19270.077	$10414_{4}^{{}^{\circ}}$ – 29684_5_
5187.773	40	1	19270.727	15970_3_ – $35240_{3}^{{}^{\circ}}$
5187.336	300	200	19272.350	2869_3_ – $22141_{3}^{{}^{\circ}}$
5187.166	100	75b	19272.982	11802_2_ – $31075_{2}^{{}^{\circ}}$
5186.412	100	75	19275.784	5563_1_ – $24838_{1}^{{}^{\circ}}$
5185.895	15	2	19277.706	$15166_{3}^{{}^{\circ}}$ – 34444_2_
5185.215	100		19280.234	$15166_{3}^{{}^{\circ}}$ – 34447_4_
5184.803	10		19281.766	21143_5_ – $40425_{4}^{{}^{\circ}}$
5184.452	100	3	19283.071	$13175_{4}^{{}^{\circ}}$ – 32458_4_
5183.336	50	3	19287.223	$16346_{4}^{{}^{\circ}}$ – 35633_4_
5183.086	50	15	19288.153	7502_3_ – $26790_{4}^{{}^{\circ}}$
5182.405	5		19290.688	$19516_{2}^{{}^{\circ}}$ – 38807_2_
5182.135	3	1	19291.693	$17224_{2}^{{}^{\circ}}$ – 36515_3_
5181.553	8	1	19293.860	2869_3_ – $22163_{4}^{{}^{\circ}}$
5180.718	100	3	19296.969	$18809_{4}^{{}^{\circ}}$ – 38106_5_
5180.406	3		19298.131	4961_4_ – $24259_{4}^{{}^{\circ}}$
5180.184	4b		19298.958	$17354_{1}^{{}^{\circ}}$ – 36653_2_
5179.140	20		19302.849	$11241_{3}^{{}^{\circ}}$ – 30544_2_
5178.852	3		19303.922	$19948_{4}^{{}^{\circ}}$ – 39252_5_
5178.691	5h		19304.522	$19503_{3}^{{}^{\circ}}$ – 38807_2_
5178.486	150	3	19305.286	$20214_{3}^{{}^{\circ}}$ – 39520_4_
5178.338	75	2	19305.838	$16783_{4}^{{}^{\circ}}$ – 36089_4_
5177.621	100	3	19308.512	$10526_{3}^{{}^{\circ}}$ – 29835_3_
5177.482	100	2	19309.030	$15736_{1}^{{}^{\circ}}$ – 35046_1_
5176.961	500	50	19310.973	$11197_{0}^{{}^{\circ}}$ – 30508_5_
5176.546	8	3	19312.521	12847_3_ – $32160_{2}^{{}^{\circ}}$
5176.402	75	1	19313.059	4961_4_ – $24274_{0}^{{}^{\circ}}$
5176.297	5		19313.450	$22399_{0}^{{}^{\circ}}$ – 41712_5_
5176.145	8		19314.017	13847_2_ – $33161_{1}^{{}^{\circ}}$
5175.911	100	3	19314.891	$12114_{2}^{{}^{\circ}}$ – 31429_1_
5175.323	100	2	19317.085	5563_1_ – $24880_{1}^{{}^{\circ}}$
5174.367	100	3	19320.654	$14206_{4}^{{}^{\circ}}$ – 33527_4_
5174.242	100	75	19321.121	$18011_{0}^{{}^{\circ}}$ – 37332_6_
5173.838	50		19322.629	$10526_{3}^{{}^{\circ}}$ – 29849_4_
5173.671	200	2	19323.253	$8243_{2}^{{}^{\circ}}$ – 27566_2_
5172.691	40		19326.914	16351_0_ – $35678_{1}^{{}^{\circ}}$
5172.479	75	3	19327.706	11601_1_ – $30928_{1}^{{}^{\circ}}$
5172.212	10		19328.704	$21902_{4}^{{}^{\circ}}$ – 41230_4_
5170.631	1		19334.614	13962_1_ – $33297_{2}^{{}^{\circ}}$
5170.569	1		19334.846	21645_4_ – $40980_{3}^{{}^{\circ}}$
5170.300	8		19335.852	$18809_{4}^{{}^{\circ}}$ – 38145_3_
5169.019	4		19340.643	8800_4_ – $28140_{4}^{{}^{\circ}}$
5168.920	100	1	19341.014	6362_2_ – $25703_{2}^{{}^{\circ}}$
5168.583	100	2	19342.275	$10414_{4}^{{}^{\circ}}$ – 29756_4_
5166.650	40b	1	19349.511	$10783_{2}^{{}^{\circ}}$ – 30132_2_
5166.618	10b		19349.631	$16783_{4}^{{}^{\circ}}$ – 36133_5_
5166.392	4		19350.477	13088_3_ – $32439_{4}^{{}^{\circ}}$
5166.245	50	1	19351.028	$18053_{4}^{{}^{\circ}}$ – 37404_3_
5165.610	100	5	19353.407	18699_2_ – $38053_{3}^{{}^{\circ}}$
5165.055	20	3	19355.486	$11197_{0}^{{}^{\circ}}$ – 30552_4_
5164.975	20	1	19355.786	14204_5_ – $33560_{4}^{{}^{\circ}}$
5164.789	31	1	19356.483	$22399_{0}^{{}^{\circ}}$ – 41755_4_
5164.400	50	2	19357.941	$14206_{4}^{{}^{\circ}}$ – 33564_3_
5164.206	40	1	19358.669	$14032_{2}^{{}^{\circ}}$ – 33390_1_
5164.108	40	1	19359.036	$16217_{2}^{{}^{\circ}}$ – 35576_3_
5163.459	400	250	19361.469	3687_2_ – $23049_{1}^{{}^{\circ}}$
5163.236	25	1	19362.305	$14243_{1}^{{}^{\circ}}$ – 33606_2_
5162.852	8		19363.745	$13945_{3}^{{}^{\circ}}$ – 33309_2_
5162.740	4		19364.166	$17411_{3}^{{}^{\circ}}$ – 36775_3_
5162.673	15	1	19364.417	17398_3_ – $36762_{3}^{{}^{\circ}}$
5162.553	50	8	19364.867	$19588_{0}^{{}^{\circ}}$ – 38953_5_
5162.356	75	5	19365.606	$19986_{6}^{{}^{\circ}}$ – 39351_5_
5161.540	400	8	19368.667	$8243_{2}^{{}^{\circ}}$ – 27612_3_
5160.718	300	50	19371.752	7280_2_ – $26651_{2}^{{}^{\circ}}$
5160.280	5		19373.396	$19948_{4}^{{}^{\circ}}$ – 39321_3_
5159.621	100	2	19375.871	7502_3_ – $26878_{3}^{{}^{\circ}}$
5159.451	20	2	19376.509	$13175_{4}^{{}^{\circ}}$ – 32551_3_
5159.339	15		19376.930	15863_2_ – $35240_{3}^{{}^{\circ}}$
5158.605	800r	50	19379.687	2869_3_ – $22248_{2}^{{}^{\circ}}$
5157.426	100	2	19384.117	$17501_{0}^{{}^{\circ}}$ – 36885_4_
5154.243	500	40	19396.088	$7795_{4}^{{}^{\circ}}$ – 27191_5_
5153.975	4b		19397.096	$17411_{3}^{{}^{\circ}}$ – 36808_3_
5152.305	4		19403.383	$19948_{4}^{{}^{\circ}}$ – 39351_5_
5151.612	500	15	19405.993	3687_2_ – $23093_{2}^{{}^{\circ}}$
5151.382	50	1	19406.860	$17411_{3}^{{}^{\circ}}$ – 36818_2_
5150.701	50	2	19409.426	$11241_{3}^{{}^{\circ}}$ – 30651_3_
5149.816	100	10	19412.761	$15736_{1}^{{}^{\circ}}$ – 35149_2_
5149.210	300	3	19415.046	13088_3_ – $32503_{2}^{{}^{\circ}}$
5147.135	40	1	19422.873	$12114_{2}^{{}^{\circ}}$ – 31537_3_
5146.059	200	10	19426.934	$16783_{4}^{{}^{\circ}}$ – 36210_5_
5143.916	300b	15	19435.027	$10414_{4}^{{}^{\circ}}$ – 29849_4_
5143.823	150b	4	19435.379	14226_0_ – $33662_{1}^{{}^{\circ}}$
5141.264	50	1	19445.052	$14243_{1}^{{}^{\circ}}$ – 33689_2_
5140.983	3	1	19446.115	12847_3_ – $32294_{4}^{{}^{\circ}}$
5140.771	200b	5	19446.917	6362_2_ – $25809_{1}^{{}^{\circ}}$
5140.716	100b	20b	19447.125	13847_2_ – $33294_{3}^{{}^{\circ}}$
5140.543	50	1	19447.779	$16346_{4}^{{}^{\circ}}$ – 35794_4_
5140.126	40	50	19449.357	13847_2_ – $33297_{2}^{{}^{\circ}}$
5139.314	3		19452.430	$18069_{3}^{{}^{\circ}}$ – 37521_3_
5138.961	15		19453.766	$19503_{3}^{{}^{\circ}}$ – 38956_4_
5138.934	15		19453.868	$21902_{4}^{{}^{\circ}}$ – 41356_5_
5138.086	8	1	19457.079	13297_4_ – $32754_{3}^{{}^{\circ}}$
5137.531	10b		19459.181	$21902_{4}^{{}^{\circ}}$ – 41361_3_
5137.475	150b	1	19459.393	4961_4_ – $24421_{3}^{{}^{\circ}}$
5137.375	2		19459.772	17166_5_ – $36625_{0}^{{}^{\circ}}$
5137.357	1		19459.840	17166_5_ – $36625_{0}^{{}^{\circ}}$
5137.300	3		19460.056	$17847_{2}^{{}^{\circ}}$ – 37307_3_
5136.396	15		19463.481	$17354_{1}^{{}^{\circ}}$ – 36818_2_
5136.121	100	1	19464.523	13962_1_ – $33427_{2}^{{}^{\circ}}$
5136.067	10b		19464.728	15863_2_ – $35328_{1}^{{}^{\circ}}$
5135.856	5	1	19465.527	$20214_{3}^{{}^{\circ}}$ – 39680_2_
5135.585	75	1	19466.554	19532_4_ – $38998_{3}^{{}^{\circ}}$
5134.746	300	3	19469.735	2869_3_ – $22338_{3}^{{}^{\circ}}$
5134.572	50	2	19470.395	$15618_{3}^{{}^{\circ}}$ – 35089_3_
5134.337	15	1	19471.286	$21890_{3}^{{}^{\circ}}$ – 41361_3_
5133.922	3		19472.860	$13945_{3}^{{}^{\circ}}$ – 33418_4_
5133.751	50	1	19473.509	173983_3_ – $36871_{2}^{{}^{\circ}}$
5133.602	5		19474.074	$17411_{3}^{{}^{\circ}}$ – 36885_4_
5133.389	10		19474.882	11601_1_ – $31075_{2}^{{}^{\circ}}$
5132.748	50	1	19477.314	$14243_{1}^{{}^{\circ}}$ – 33721_2_
5131.992	150	1	19480.183	11802_2_ – $31283_{3}^{{}^{\circ}}$
5131.257	5		19482.973	17073_1_ – $36556_{1}^{{}^{\circ}}$
5130.814	20b		19484.656	$16346_{4}^{{}^{\circ}}$ – 35831_3_
5130.777	20b		19484.796	$10526_{3}^{{}^{\circ}}$ – 30011_3_
5130.235	150	2	19486.855	13088_3_ – $32575_{2}^{{}^{\circ}}$
5129.988	8		19487.793	$10526_{3}^{{}^{\circ}}$ – 30014_4_
5129.656	20		19489.054	21645_4_ – $41134_{4}^{{}^{\circ}}$
5129.206	50	1	19490.764	$19039_{2}^{{}^{\circ}}$ – 38529_3_
5128.891	2		19491.961	15970_3_ – $35462_{3}^{{}^{\circ}}$
5128.574	20		19493.166	$19516_{2}^{{}^{\circ}}$ – 39010_3_
5128.489	300	5	19493.489	7502_3_ – $26995_{3}^{{}^{\circ}}$
5125.950	200	3	19503.144	0_2_ – $19503_{3}^{{}^{\circ}}$
5125.827	50b	1	19503.612	18549_2_ – $38053_{3}^{{}^{\circ}}$
5125.797	50b	2	19503.727	$10783_{2}^{{}^{\circ}}$ – 30286_1_
5125.307	5b		19505.591	$21077_{0}^{{}^{\circ}}$ – 40582_5_
5124.925	3		19507.045	$19503_{3}^{{}^{\circ}}$ – 39010_3_
5123.239	50	3	19513.464	$16783_{4}^{{}^{\circ}}$ – 36297_4_
5123.131	50		19513.876	$13945_{3}^{{}^{\circ}}$ – 33459_4_
5123.006	8		19514.352	18574_1_ – $38088_{2}^{{}^{\circ}}$
5122.226	10		19517.323	18699_2_ – $38216_{3}^{{}^{\circ}}$
5121.953	4		19518.364	18431_3_ – $37950_{4}^{{}^{\circ}}$
5121.699	2		19519.332	$22508_{2}^{{}^{\circ}}$ – 42027_2_
5120.593	4		19523.548	$22163_{4}^{{}^{\circ}}$ – 41686_3_
5119.867	20		19526.316	$14247_{0}^{{}^{\circ}}$ – 33773_1_
5119.000	1		19529.623	$14243_{1}^{{}^{\circ}}$ – 33773_1_
5118.176	15		19532.767	$14032_{2}^{{}^{\circ}}$ – 33564_3_
5116.661	2h		19538.551	18431_3_ – $37970_{2}^{{}^{\circ}}$
5115.045	500r	40	19544.724	3865_1_ – $23410_{0}^{{}^{\circ}}$
5114.065	8	1	19548.469	$7795_{4}^{{}^{\circ}}$ – 27343_3_
5113.733	2		19549.738	$22163_{4}^{{}^{\circ}}$ – 41712_5_
5113.050	10	3	19552.349	$16783_{4}^{{}^{\circ}}$ – 36336_3_
5111.911	4		19556.706	$18809_{4}^{{}^{\circ}}$ – 38366_4_
5111.685	25	2	19557.570	$17847_{2}^{{}^{\circ}}$ – 37404_3_
5111.280	150	4	19559.120	7502_3_ – $27061_{2}^{{}^{\circ}}$
5111.063	200	5	19559.951	8111_4_ – $27670_{3}^{{}^{\circ}}$
5110.555	150	2	19561.895	$13175_{4}^{{}^{\circ}}$ – 32737_5_
5110.216	25		19563.192	15970_3_ – $35533_{2}^{{}^{\circ}}$
5110.056	50	2	19563.805	$19039_{2}^{{}^{\circ}}$ – 38602_2_
5109.735	200	3	19565.034	13297_4_ – $32862_{4}^{{}^{\circ}}$
5107.970	3		19571.794	$19948_{4}^{{}^{\circ}}$ – 39520_4_
5107.338	5		19574.216	$14032_{2}^{{}^{\circ}}$ – 33606_2_
5106.023	5		19579.257	13847_2_ – $33427_{2}^{{}^{\circ}}$
5105.242	5		19582.252	$13945_{3}^{{}^{\circ}}$ – 33527_4_
5102.509	3		19592.741	$22163_{4}^{{}^{\circ}}$ – 41755_4_
5102.228	2		19593.820	$17224_{2}^{{}^{\circ}}$ – 36818_2_
5101.799	40	1	19595.468	14204_5_ – $33799_{4}^{{}^{\circ}}$
5101.344	40	1	19597.215	$10414_{4}^{{}^{\circ}}$ – 30011_3_
5101.130	300	4	19598.037	7280_2_ – $26878_{3}^{{}^{\circ}}$
5100.621	300b		19599.993	4961_4_ – $24561_{3}^{{}^{\circ}}$
5100.573	150b	2b	19600.178	$10414_{4}^{{}^{\circ}}$ – 30014_4_
5099.030	50	1	19606.109	$10526_{3}^{{}^{\circ}}$ – 30132_2_
5098.933	150	2	19606.482	$13175_{4}^{{}^{\circ}}$ – 32781_4_
5097.644	8	1	19611.439	17959_4_ – $37571_{0}^{{}^{\circ}}$
5097.039	2		19613.767	$16217_{2}^{{}^{\circ}}$ – 35831_3_
5096.484	500	5	19615.903	3865_1_ – $23481_{1}^{{}^{\circ}}$
5095.644	5	1	19619.137	$18069_{3}^{{}^{\circ}}$ – 37688_3_
5095.539	3		19619.541	$13945_{3}^{{}^{\circ}}$ – 33564_3_
5095.149	20h		19621.043	$13175_{4}^{{}^{\circ}}$ – 32796_3_
5095.074	300	50	19621.331	18431_3_ – $38053_{3}^{{}^{\circ}}$
5094.546	20	3	19623.365	$21738_{2}^{{}^{\circ}}$ – 41361_3_
5094.334	2		19624.182	$21077_{0}^{{}^{\circ}}$ – 40701_4_
5094.127	200	50	19624.979	$14206_{4}^{{}^{\circ}}$ – 33831_4_
5092.023	1		19633.088	21143_5_ – $40776_{4}^{{}^{\circ}}$
5091.641	2h		19634.561	17166_5_ – $36800_{4}^{{}^{\circ}}$
5090.548	300	2	19638.777	2869_3_ – $22508_{2}^{{}^{\circ}}$
5090.052	400	5	19640.690	14204_5_ – $33844_{0}^{{}^{\circ}}$
5086.180	150	3	19655.642	12847_3_ – $32503_{2}^{{}^{\circ}}$
5085.839	15	1	19656.960	$14032_{2}^{{}^{\circ}}$ – 33689_2_
5085.761	4		19657.261	18431_3_ – $38088_{2}^{{}^{\circ}}$
5084.997	200	8	19660.215	$12114_{2}^{{}^{\circ}}$ – 31774_3_
5084.800	150		19660.976	$13945_{3}^{{}^{\circ}}$ – 33606_2_
5084.038	15	1	19663.923	$19588_{0}^{{}^{\circ}}$ – 39252_5_
5083.516	1		19665.942	13088_3_ – $32754_{3}^{{}^{\circ}}$
5082.625	100	8.	19669.390	15863_2_ – $35533_{2}^{{}^{\circ}}$
5082.005	20	8	19671.789	$19503_{3}^{{}^{\circ}}$ – 39174_3_
5081.778	15	2	19672.668	$18930_{3}^{{}^{\circ}}$ – 38602_2_
5081.449	200	4	19673.942	6362_2_ – $26036_{3}^{{}^{\circ}}$
5081.326	2		19674.418	$17847_{2}^{{}^{\circ}}$ – 37521_3_
5080.157	1		19678.945	$18069_{3}^{{}^{\circ}}$ – 37748_2_
5079.911	15	2	19679.898	8800_4_ – $28480_{4}^{{}^{\circ}}$
5078.848	40	1	19684.017	$15736_{1}^{{}^{\circ}}$ – 35421_1_
5077.510	5	1	19689.204	$14032_{2}^{{}^{\circ}}$ – 33721_2_
5075.955	50	1	19695.236	$19039_{2}^{{}^{\circ}}$ – 38734_3_
5074.807	10		19699.691	13962_1_ – $33662_{1}^{{}^{\circ}}$
5074.649	25	1	19700.304	$7795_{4}^{{}^{\circ}}$ – 27495_4_
5072.885	150	1	19707.155	$15166_{3}^{{}^{\circ}}$ – 34874_3_
5070.781	75	2	19715.332	$18069_{3}^{{}^{\circ}}$ – 37784_2_
5070.456	50	1	19716.595	18699_2_ – $38416_{3}^{{}^{\circ}}$
5069.626	1		19719.823	$17411_{3}^{{}^{\circ}}$ – 37131_3_
5069.337	200	4	19720.948	16554_6_ – $36275_{0}^{{}^{\circ}}$
5069.093	20	2	19721.897	$21165_{3}^{{}^{\circ}}$ – 40886_2_
5068.829	25	2	19722.924	$11241_{3}^{{}^{\circ}}$ – 30964_3_
5067.974	1000r	75	19726.251	$7795_{4}^{{}^{\circ}}$ – 27521_4_
5067.807	20		19726.901	$15490_{0}^{{}^{\circ}}$ – 35216_5_
5067.667	5	5	19727.446	12847_3_ – $32575_{2}^{{}^{\circ}}$
5067.138	50	2	19729.506	18549_2_ – $38278_{1}^{{}^{\circ}}$
5066.959	80	20	19730.203	$20322_{0}^{{}^{\circ}}$ – 40052_4_
5066.777	200	40	19730.911	$18011_{0}^{{}^{\circ}}$ – 37742_6_
5066.461	4		19732.142	$16783_{4}^{{}^{\circ}}$ – 36515_3_
5066.134	300	10	19733.416	$11197_{0}^{{}^{\circ}}$ – 30930_6_
5065.190	300	5	19737.093	$8243_{2}^{{}^{\circ}}$ – 27980_3_
5064.945	300r	8	19738.048	3865_1_ – $23603_{2}^{{}^{\circ}}$
5064.601	300	40	19739.389	4961_4_ – $24701_{0}^{{}^{\circ}}$
5064.004	100b	2d	19741.716	8111_4_ – $27852_{0}^{{}^{\circ}}$
5063.658	8	1	19743.065	$16346_{4}^{{}^{\circ}}$ – 36089_4_
5063.565	20h		19743.427	13847_2_ – $33591_{3}^{{}^{\circ}}$
5063.514	2001	501	19743.626	5563_1_ – $25306_{2}^{{}^{\circ}}$
5062.932	300	5	19745.896	13297_4_ – $33043_{3}^{{}^{\circ}}$
5062.494	50	2	19747.604	15493_4_ – $35240_{3}^{{}^{\circ}}$
5061.654	200	5	19750.881	6362_2_ – $26113_{2}^{{}^{\circ}}$
5060.366	3		19755.908	13962_1_ – $33718_{2}^{{}^{\circ}}$
5059.861	400b	75	19757.880	7502_3_ – $27260_{3}^{{}^{\circ}}$
5059.021	8	1	19761.161	17398_3_ – $37159_{3}^{{}^{\circ}}$
5058.446	100	3	19763.407	17073_1_ – $36837_{1}^{{}^{\circ}}$
5058.446	100	3	19763.407	$19588_{0}^{{}^{\circ}}$ – 39351_5_
5058.359	200	3	19763.747	7502_3_ – $27266_{4}^{{}^{\circ}}$
5057.137	5		19768.522	$19039_{2}^{{}^{\circ}}$ – 38807_2_
5056.766	10		19769.973	$18930_{3}^{{}^{\circ}}$ – 38700_4_
5056.194	50	2	19772.209	13962_1_ – $33734_{2}^{{}^{\circ}}$
5055.751	10b		19773.942	13088_3_ – $32862_{4}^{{}^{\circ}}$
5055.225	300	4	19775.999	$13945_{3}^{{}^{\circ}}$ – 33721_2_
5054.493	3		19778.863	19532_4_ – $39311_{4}^{{}^{\circ}}$
5053.067	3		19784.445	$21902_{4}^{{}^{\circ}}$ – 41686_3_
5052.854	8	1	19785.279	18431_3_ – $38216_{3}^{{}^{\circ}}$
5052.448	15		19786.869	$16346_{4}^{{}^{\circ}}$ – 36133_5_
5051.885	500	5	19789.074	8800_4_ – $28589_{3}^{{}^{\circ}}$
5050.780	300	5	19793.403	3687_2_ – $23481_{1}^{{}^{\circ}}$
5050.029	5b		19796.347	11802_2_ – $31599_{2}^{{}^{\circ}}$
5049.976	10b		19796.554	$7795_{4}^{{}^{\circ}}$ – 27591_5_
5049.976	10b		19796.554	$21890_{3}^{{}^{\circ}}$ – 41686_3_
5049.841	50b	1	19797.083	14204_5_ – $34001_{4}^{{}^{\circ}}$
5049.580	40	8	19798.107	17073_1_ – $36871_{2}^{{}^{\circ}}$
5048.937	20	1	19800.628	2869_3_ – $22669_{3}^{{}^{\circ}}$
5047.600	2		19805.873	18549_2_ – $38355_{2}^{{}^{\circ}}$
5047.122	5h	1	19807.748	$18011_{0}^{{}^{\circ}}$ – 37819_4_
5047.047	150b	5	19808.043	4961_4_ – $24769_{3}^{{}^{\circ}}$
5045.417	3		19814.442	13847_2_ – $33662_{1}^{{}^{\circ}}$
5045.252	100	5	19815.090	7502_3_ – $27317_{3}^{{}^{\circ}}$
5044.715	400r	8	19817.199	0_2_ – $19817_{1}^{{}^{\circ}}$
5044.349	5	1	19818.637	$19503_{3}^{{}^{\circ}}$ – 39321_3_
5043.520	75	151	19821.895	$15618_{3}^{{}^{\circ}}$ – 35440_3_
5041.514	2		19829.782	$16217_{2}^{{}^{\circ}}$ – 36047_2_
5041.377	3		19830.320	$18930_{3}^{{}^{\circ}}$ – 38760_4_
5041.126	150	5	19831.308	$17501_{0}^{{}^{\circ}}$ – 37332_6_
5040.683	251	1	19833.051	3687_2_ – $23521_{3}^{{}^{\circ}}$
5040.032	2		19835.612	17398_3_ – $37234_{2}^{{}^{\circ}}$
5039.717	1		19836.852	$18053_{4}^{{}^{\circ}}$ – 37890_5_
5039.529	50	1	19837.592	8111_4_ – $27948_{4}^{{}^{\circ}}$
5039.231	300r	81	19838.765	2558_0_ – $22396_{1}^{{}^{\circ}}$
5038.618	1		19841.179	$17847_{2}^{{}^{\circ}}$ – 37688_3_
5038.306	75	3	19842.407	15970_3_ – $35812_{3}^{{}^{\circ}}$
5036.623	3		19849.038	19713_3_ – $39562_{4}^{{}^{\circ}}$
5035.466	5		19853.598	$21902_{4}^{{}^{\circ}}$ – 41755_4_
5034.297	100	2	19858.209	15493_4_ – $35351_{4}^{{}^{\circ}}$
5033.010	2		19863.286	$19817_{1}^{{}^{\circ}}$ – 39680_2_
5032.786	1		19864.171	$16346_{4}^{{}^{\circ}}$ – 36210_5_
5032.119	4		19866.803	18549_2_ – $38416_{3}^{{}^{\circ}}$
5031.815	10		19868.004	$10783_{2}^{{}^{\circ}}$ – 30651_3_
5031.148	20		19870.638	13847_2_ – $33718_{2}^{{}^{\circ}}$
5030.116	2		19874.714	18431_3_ – $38306_{4}^{{}^{\circ}}$
5030.031	3		19875.050	17166_5_ – $37041_{4}^{{}^{\circ}}$
5029.893	200	3	19875.595	3865_1_ – $23741_{1}^{{}^{\circ}}$
5029.013	75	2	19879.073	$18011_{0}^{{}^{\circ}}$ – 37890_5_
5028.656	300b	15	19880.485	8800_4_ – $28680_{4}^{{}^{\circ}}$
5027.679	5		19884.348	14226_0_ – $34111_{1}^{{}^{\circ}}$
5027.318	2		19885.776	$22141_{3}^{{}^{\circ}}$ – 42027_2_
5027.122	4		19886.551	$13945_{3}^{{}^{\circ}}$ – 33831_4_
5027.024	5		19886.939	13847_2_ – $33734_{2}^{{}^{\circ}}$
5026.956	5		19887.208	3865_1_ – $23752_{2}^{{}^{\circ}}$
5026.159	5		19890.361	$18809_{4}^{{}^{\circ}}$ – 38700_4_
5024.110	8		19898.473	19713_3_ – $39611_{3}^{{}^{\circ}}$
5023.709	50	1	19900.061	$11241_{3}^{{}^{\circ}}$ – 31141_3_
5023.486	50	1	19900.945	$17847_{2}^{{}^{\circ}}$ – 37748_2_
5022.070	5		19906.556	12847_3_ – $32754_{3}^{{}^{\circ}}$
5022.005	75	2	19906.813	9804_5_ – $29711_{0}^{{}^{\circ}}$
5021.254	75	2	19909.791	11802_2_ – $31712_{1}^{{}^{\circ}}$
5019.806	200	25	19915.534	3687_2_ – $23603_{2}^{{}^{\circ}}$
5019.752	10b		19915.748	15970_3_ – $35885_{2}^{{}^{\circ}}$
5018.059	150	5	19922.467	$15166_{3}^{{}^{\circ}}$ – 35089_3_
5017.877	10	2	19923.190	$18069_{3}^{{}^{\circ}}$ – 37992_4_
5017.769	10	2	19923.619	18431_3_ – $38355_{2}^{{}^{\circ}}$
5017.589	5		19924.333	$13175_{4}^{{}^{\circ}}$ – 33099_3_
5017.510	100	15	19924.647	6362_2_ – $26287_{1}^{{}^{\circ}}$
5016.951	8		19926.867	$12114_{2}^{{}^{\circ}}$ – 32041_2_
5016.535	40	2	19928.520	9804_5_ – $29733_{0}^{{}^{\circ}}$
5012.274	5		19945.461	7502_3_ – $27447_{2}^{{}^{\circ}}$
5011.478	75	4	19948.629	15863_2_ – $35812_{3}^{{}^{\circ}}$
5011.478	75	4	19948.629	$21738_{2}^{{}^{\circ}}$ – 41686_3_
5010.951	100	2	19950.727	$18809_{4}^{{}^{\circ}}$ – 38760_4_
5010.417	50	4	19952.853	13847_2_ – $33800_{3}^{{}^{\circ}}$
5009.938	50b		19954.761	13088_3_ – $33043_{3}^{{}^{\circ}}$
5009.240	10	1	19957.541	$15618_{3}^{{}^{\circ}}$ – 35576_3_
5008.481	2		19960.565	$14206_{4}^{{}^{\circ}}$ – 34167_3_
5007.087	5		19966.123	$14465_{2}^{{}^{\circ}}$ – 34431_3_
5006.405	5		19968.842	15493_4_ – $35462_{3}^{{}^{\circ}}$
5006.073	1		19970.167	18699_2_ – $38669_{2}^{{}^{\circ}}$
5005.970	20	5	19970.578	$20522_{2}^{{}^{\circ}}$ – 40493_3_
5005.334	15	1	19973.115	13297_4_ – $33270_{4}^{{}^{\circ}}$
5004.900	2		19974.847	$10783_{2}^{{}^{\circ}}$ – 30758_2_
5004.685	1		19975.705	18699_2_ – $38675_{3}^{{}^{\circ}}$
5004.128	150	10	19977.929	11802_2_ – $31780_{3}^{{}^{\circ}}$
5004.001	50	3	19978.436	14204_5_ – $34182_{0}^{{}^{\circ}}$
5003.857	20	2	19979.011	$14481_{6}^{{}^{\circ}}$ – 34460_5_
5003.767	3		19979.370	$14465_{2}^{{}^{\circ}}$ – 34444_2_
5003.597	75	4	19980.049	7280_2_ – $27260_{3}^{{}^{\circ}}$
5003.385	10s	2	19980.895	$18011_{0}^{{}^{\circ}}$ – 37992_4_
5002.898	15	3	19982.840	$15166_{3}^{{}^{\circ}}$ – 35149_2_
5002.472	3		19984.542	18431_3_ – $38416_{3}^{{}^{\circ}}$
5002.225	3		19985.529	$20214_{3}^{{}^{\circ}}$ – 40200_3_
5002.097	500r	40	19986.040	2869_3_ – $22855_{3}^{{}^{\circ}}$
5001.450	8	1	19988.626	$18614_{1}^{{}^{\circ}}$ – 38602_2_
5001.068	2		19990.152	17959_4_ – $37950_{4}^{{}^{\circ}}$
5001.068	2		19990.152	17959_4_ – $37950_{4}^{{}^{\circ}}$
5000.730	15	1	19991.504	$16783_{4}^{{}^{\circ}}$ – 36775_3_
5000.252	100	25d	19993.414	$17411_{3}^{{}^{\circ}}$ – 37404_3_
4999.841	2		19995.058	17959_4_ – $37954_{3}^{{}^{\circ}}$
4999.239	10	2	19997.466	$16783_{4}^{{}^{\circ}}$ – 36781_5_
4998.419	1		20000.746	6362_2_ – $26363_{2}^{{}^{\circ}}$
4997.566	3		20004.160	$11241_{3}^{{}^{\circ}}$ – 31245_2_
4996.229	5	1	20009.513	16351_0_ – $36361_{1}^{{}^{\circ}}$
4994.982	10		20014.509	12847_3_ – $32862_{4}^{{}^{\circ}}$
4994.891	5		20014.873	$15618_{3}^{{}^{\circ}}$ – 35633_4_
4994.576	10	3	20016.135	$20566_{4}^{{}^{\circ}}$ – 40582_5_
4994.105	50	2	20018.023	$10526_{3}^{{}^{\circ}}$ – 30544_2_
4993.749	50	4	20019.450	4961_4_ – $24981_{3}^{{}^{\circ}}$
4993.111	1		20022.008	15863_2_ – $35885_{2}^{{}^{\circ}}$
4992.638	20	5	20023.905	$11241_{3}^{{}^{\circ}}$ – 31265_3_
4992.375	8	2	20024.960	$19227_{6}^{{}^{\circ}}$ – 39252_5_
4992.125	15	2	20025.963	$10526_{3}^{{}^{\circ}}$ – 30552_4_
4991.603	1		20028.057	$18069_{3}^{{}^{\circ}}$ – 38097_2_
4989.309	150	10	20037.265	7280_2_ – $27317_{3}^{{}^{\circ}}$
4988.772	4		20039.422	$13175_{4}^{{}^{\circ}}$ – 33214_5_
4988.262	1		20041.471	20054_2_ – $40096_{2}^{{}^{\circ}}$
4985.948	50	3	20050.772	$14247_{0}^{{}^{\circ}}$ – 34298_1_
4985.373	300	10	20053.085	3687_2_ – $23741_{1}^{{}^{\circ}}$
4985.125	8		20054.082	$14243_{1}^{{}^{\circ}}$ – 34298_1_
4982.488	150	25h	20064.696	3687_2_ – $23752_{2}^{{}^{\circ}}$
4981.877	41		20067.157	11802_2_ – $31870_{2}^{{}^{\circ}}$
4980.757	20	3	20071.669	16554_6_ – $36625_{0}^{{}^{\circ}}$
4980.186	150	10	20073.970	$11197_{0}^{{}^{\circ}}$ – 31271_5_
4979.521	4b		20076.651	9804_5_ – $29881_{4}^{{}^{\circ}}$
4979.521	4b		20076.651	$18069_{3}^{{}^{\circ}}$ – 38145_3_
4978.727	2		20079.853	$18930_{3}^{{}^{\circ}}$ – 39010_3_
4977.522	5		20084.714	8800_4_ – $28884_{3}^{{}^{\circ}}$
4977.389	8	1	20085.251	$11241_{3}^{{}^{\circ}}$ – 31326_4_
4976.471	1		20088.956	$20522_{2}^{{}^{\circ}}$ – 40611_2_
4976.406	1		20089.218	18699_2_ – $38788_{3}^{{}^{\circ}}$
4975.524	5		20092.779	15970_3_ – $36062_{4}^{{}^{\circ}}$
4975.442	3		20093.110	17959_4_ – $38053_{3}^{{}^{\circ}}$
4975.254	2		20093.869	$10414_{4}^{{}^{\circ}}$ – 30508_5_
4975.088	10	1	20094.540	$14206_{4}^{{}^{\circ}}$ – 34301_4_
4972.754	20	2	20103.971	15970_3_ – $36074_{3}^{{}^{\circ}}$
4972.258	15	1	20105.977	$19588_{0}^{{}^{\circ}}$ – 39694_5_
4972.057	40	1	20106.789	$10783_{2}^{{}^{\circ}}$ – 30889_1_
4971.553	2		20108.828	$21252_{2}^{{}^{\circ}}$ – 41361_3_
4970.074	150b	2	20114.812	$8243_{2}^{{}^{\circ}}$ – 28358_3_
4969.518	2		20117.062	$18069_{3}^{{}^{\circ}}$ – 38186_4_
4968.753	200	20	20120.159	13847_2_ – $33967_{3}^{{}^{\circ}}$
4967.649	5		20124.631	$10526_{3}^{{}^{\circ}}$ – 30651_3_
4967.334	5	1	20125.907	18549_2_ – $38675_{3}^{{}^{\circ}}$
4967.334	5	1	20125.907	21594_3_ – $41720_{2}^{{}^{\circ}}$
4965.731	200	10	20132.404	8800_4_ – $28932_{4}^{{}^{\circ}}$
4964.538	2		20137.242	$21890_{3}^{{}^{\circ}}$ – 42027_2_
4964.255	10	2	20138.390	$10414_{4}^{{}^{\circ}}$ – 30552_4_
4963.763	75	50	20140.386	14204_5_ – $34344_{4}^{{}^{\circ}}$
4963.031	8		20143.356	$18809_{4}^{{}^{\circ}}$ – 38953_5_
4962.466	5		20145.650	6362_2_ – $26508_{3}^{{}^{\circ}}$
4961.724	200	10	20148.662	13962_1_ – $34111_{1}^{{}^{\circ}}$
4958.878	2		20160.226	17073_1_ – $37234_{2}^{{}^{\circ}}$
4958.093	50	2	20163.418	$11877_{1}^{{}^{\circ}}$ – 32041_2_
4957.057	15		20167.632	7280_2_ – $27447_{2}^{{}^{\circ}}$
4956.798	20b		20168.685	7502_3_ – $27670_{3}^{{}^{\circ}}$
4956.637	50	2	20169.340	$16346_{4}^{{}^{\circ}}$ – 36515_3_
4955.303	3		20174.770	$18011_{0}^{{}^{\circ}}$ – 38186_4_
4953.936	25	3	20180.337	$17224_{2}^{{}^{\circ}}$ – 37404_3_
4953.646	2		20181.519	$10783_{2}^{{}^{\circ}}$ – 30964_3_
4953.529	50	10	20181.995	13088_3_ – $33270_{4}^{{}^{\circ}}$
4952.688	40	3	20185.422	$7795_{4}^{{}^{\circ}}$ – 27980_3_
4952.009	2		20188.190	$20423_{1}^{{}^{\circ}}$ – 40611_2_
4950.755	8		20193.303	$18614_{1}^{{}^{\circ}}$ – 38807_2_
4950.250	150	10	20195.363	12847_3_ – $33043_{3}^{{}^{\circ}}$
4949.065	2		20200.199	20054_2_ – $40254_{3}^{{}^{\circ}}$
4948.964	8		20200.611	$14243_{1}^{{}^{\circ}}$ – 34444_2_
4948.565	1		20202.240	$23655_{4}^{{}^{\circ}}$ – 43857_5_
4947.015	5		20208.570	13088_3_ – $33297_{2}^{{}^{\circ}}$
4946.220	2		20211.818	$20214_{3}^{{}^{\circ}}$ – 40426_4_
4945.458	200r	10	20214.932	0_2_ – $20214_{3}^{{}^{\circ}}$
4943.130	50b	2	20224.452	$14206_{4}^{{}^{\circ}}$ – 34431_3_
4943.064	200b	10	20224.722	2869_3_ – $23093_{2}^{{}^{\circ}}$
4941.415	15	1	20231.471	$10526_{3}^{{}^{\circ}}$ – 30758_2_
4940.907	20	2	20233.551	$13175_{4}^{{}^{\circ}}$ – 33408_3_
4940.057	5		20237.033	$10414_{4}^{{}^{\circ}}$ – 30651_3_
4939.642	300r	15	20238.733	$7795_{4}^{{}^{\circ}}$ – 28034_5_
4939.270	75	2	20240.257	$14206_{4}^{{}^{\circ}}$ – 34447_4_
4939.059	10	1	20241.122	$17501_{0}^{{}^{\circ}}$ – 37742_6_
4938.720	3		20242.511	$14465_{2}^{{}^{\circ}}$ – 34707_3_
4938.597	15	1	20243.015	$13175_{4}^{{}^{\circ}}$ – 33418_4_
4938.439	10	1	20243.663	18431_3_ – $38675_{3}^{{}^{\circ}}$
4938.283	15		20244.302	2869_3_ – $23113_{4}^{{}^{\circ}}$
4938.206	20	2	20244.618	$18930_{3}^{{}^{\circ}}$ – 39174_3_
4937.829	300	8	20246.164	5563_1_ – $25809_{1}^{{}^{\circ}}$
4936.889	25	1	20250.019	$17847_{2}^{{}^{\circ}}$ – 38097_2_
4936.775	150	100	20250.486	8800_4_ – $29050_{0}^{{}^{\circ}}$
4935.929	20	1	20253.957	$14206_{4}^{{}^{\circ}}$ – 34460_5_
4935.177	5		20257.043	17959_4_ – $38216_{3}^{{}^{\circ}}$
4934.807	3		20258.562	17398_3_ – $37656_{3}^{{}^{\circ}}$
4934.748	1		20258.804	$15618_{3}^{{}^{\circ}}$ – 35877_2_
4934.337	15	1	20260.492	$20322_{0}^{{}^{\circ}}$ – 40582_5_
4933.825	15b		20262.594	13297_4_ – $33560_{4}^{{}^{\circ}}$
4933.283	3		20264.820	18549_2_ – $38814_{2}^{{}^{\circ}}$
4933.003	20	1	20265.970	$14032_{2}^{{}^{\circ}}$ – 34298_1_
4932.250	5		20269.064	11601_1_ – $31870_{2}^{{}^{\circ}}$
4931.373	10b		20272.669	19713_3_ – $39985_{3}^{{}^{\circ}}$
4931.050	10		20273.997	$15166_{3}^{{}^{\circ}}$ – 35440_3_
4930.322	3		20276.990	$17411_{3}^{{}^{\circ}}$ – 37688_3_
4929.832	5		20279.006	$21077_{0}^{{}^{\circ}}$ – 41356_5_
4929.086	100	3	20282.075	7502_3_ – $27784_{2}^{{}^{\circ}}$
4928.949	5		20282.639	$19039_{2}^{{}^{\circ}}$ – 39321_3_
4928.683	50	2	20283.733	16554_6_ – $36837_{0}^{{}^{\circ}}$
4928.604	8		20284.058	$13175_{4}^{{}^{\circ}}$ – 33459_4_
4927.781	200	5	20287.446	$8243_{2}^{{}^{\circ}}$ – 28531_2_
4927.299	50	1	20289.431	6362_2_ – $26651_{2}^{{}^{\circ}}$
4926.254	40	2	20293.734	13297_4_ – $33591_{3}^{{}^{\circ}}$
4925.822	8b	1b	20295.514	$11241_{3}^{{}^{\circ}}$ – 31537_3_
4925.339	5	2	20297.505	$18069_{3}^{{}^{\circ}}$ – 38366_4_
4925.097	3b		20298.502	$16217_{2}^{{}^{\circ}}$ – 36515_3_
4924.896	8	1	20299.330	18699_2_ – $38998_{3}^{{}^{\circ}}$
4924.246	75b	2	20302.010	$16783_{4}^{{}^{\circ}}$ – 37085_4_
4923.690	5		20304.302	$15490_{0}^{{}^{\circ}}$ – 35794_4_
4922.338	2		20309.879	$15490_{0}^{{}^{\circ}}$ – 35799_6_
4922.231	5		20310.321	$15736_{1}^{{}^{\circ}}$ – 36047_2_
4921.653	40b		20312.706	$10783_{2}^{{}^{\circ}}$ – 31095_1_
4920.624	50	2	20316.954	3865_1_ – $24182_{2}^{{}^{\circ}}$
4920.053	75b	25s	20319.312	15493_4_ – $35812_{3}^{{}^{\circ}}$
4920.018	75b	2	20319.456	2558_0_ – $22877_{1}^{{}^{\circ}}$
4918.659	3		20325.070	15863_2_ – $36188_{2}^{{}^{\circ}}$
4916.383	2h		20334.479	$19503_{3}^{{}^{\circ}}$ – 39837_3_
4915.823	10	1	20336.796	$17411_{3}^{{}^{\circ}}$ – 37748_2_
4915.416	50	1	20338.480	13088_3_ – $33427_{2}^{{}^{\circ}}$
4913.480	5	1	20346.493	17959_4_ – $38306_{4}^{{}^{\circ}}$
4912.043	10	1	20352.445	$13175_{4}^{{}^{\circ}}$ – 33527_4_
4911.784	2		20353.519	$18930_{3}^{{}^{\circ}}$ – 39283_4_
4911.380	200	10	20355.193	9804_5_ – $30160_{4}^{{}^{\circ}}$
4911.380	200	10	20355.193	$18011_{0}^{{}^{\circ}}$ – 38366_4_
4910.807	100b	2	20357.568	11802_2_ – $32160_{2}^{{}^{\circ}}$
4910.792	100b	2	20357.630	8800_4_ – $29157_{3}^{{}^{\circ}}$
4910.549	15	1	20358.637	$10783_{2}^{{}^{\circ}}$ – 31141_3_
4910.158	100	4	20360.259	4961_4_ – $25321_{3}^{{}^{\circ}}$
4909.844	50	3	20361.561	13962_1_ – $34324_{2}^{{}^{\circ}}$
4909.213	15	1	20364.178	14226_0_ – $34591_{1}^{{}^{\circ}}$
4909.010	8	1	20365.020	$18809_{4}^{{}^{\circ}}$ – 39174_3_
4908.478	100	1	20367.227	$14481_{6}^{{}^{\circ}}$ – 34849_5_
4908.012	5		20369.161	8111_4_ – $28480_{4}^{{}^{\circ}}$
4907.044	50	2	20373.179	$17411_{3}^{{}^{\circ}}$ – 37784_2_
4906.731	2		20374.479	19532_4_ – $39906_{4}^{{}^{\circ}}$
4905.631	2		20379.047	$20322_{0}^{{}^{\circ}}$ – 40701_4_
4904.407	1		20384.133	$21902_{4}^{{}^{\circ}}$ – 42286_3_
4903.057	8	1	20389.746	$13175_{4}^{{}^{\circ}}$ – 33564_3_
4902.793	75	5	20390.843	7280_2_ – $27670_{3}^{{}^{\circ}}$
4902.600	50	5	20391.646	$17501_{0}^{{}^{\circ}}$ – 37892_4_
4902.177	2		20393.406	$17354_{1}^{{}^{\circ}}$ – 37748_2_
4902.051	75	5	20393.930	4961_4_ – $25355_{4}^{{}^{\circ}}$
4901.987	50	2	20394.196	7280_2_ – $27674_{2}^{{}^{\circ}}$
4901.987	50	2	20394.196	11802_2_ – $32197_{3}^{{}^{\circ}}$
4901.479	4		20396.310	$21890_{3}^{{}^{\circ}}$ – 42286_3_
4901.371	3		20396.759	$20214_{3}^{{}^{\circ}}$ – 40611_2_
4899.336	50	3	20405.231	17166_5_ – $37571_{0}^{{}^{\circ}}$
4898.697	10	1	20407.893	$17411_{3}^{{}^{\circ}}$ – 37819_4_
4898.358	25b	1	20409.305	13962_1_ – $34371_{2}^{{}^{\circ}}$
4897.586	8	1	20412.522	$14032_{2}^{{}^{\circ}}$ – 34444_2_
4897.586	8	1	20412.522	15970_3_ – $36382_{4}^{{}^{\circ}}$
4895.168	3		20422.605	12847_3_ – $33270_{4}^{{}^{\circ}}$
4894.955	400r	10	20423.494	0_2_ – $20423_{1}^{{}^{\circ}}$
4894.520	4		20425.309	17959_4_ – $38385_{0}^{{}^{\circ}}$
4893.910	4		20427.855	$16783_{4}^{{}^{\circ}}$ – 37211_5_
4893.704	10	1	20428.715	$16346_{4}^{{}^{\circ}}$ – 36775_3_
4893.444	50	2	20429.800	$11197_{0}^{{}^{\circ}}$ – 31626_5_
4892.758	50	2	20432.664	$7795_{4}^{{}^{\circ}}$ – 28227_4_
4892.415	1		20434.097	$21252_{2}^{{}^{\circ}}$ – 41686_3_
4892.276	5	1	20434.677	$16346_{4}^{{}^{\circ}}$ – 36781_5_
4892.038	5		20435.671	$8243_{2}^{{}^{\circ}}$ – 28679_2_
4891.658	15	1	20437.259	$12114_{2}^{{}^{\circ}}$ – 32551_3_
4891.453	4		20438.115	$10526_{3}^{{}^{\circ}}$ – 30964_3_
4890.455	50	8	20442.286	3865_1_ – $24307_{2}^{{}^{\circ}}$
4889.489	50	2	20446.325	7502_3_ – $27948_{4}^{{}^{\circ}}$
4888.811	4		20449.161	12847_3_ – $33297_{2}^{{}^{\circ}}$
4888.714	4		20449.566	18549_2_ – $38998_{3}^{{}^{\circ}}$
4887.822	1		20453.298	19532_4_ – $39985_{3}^{{}^{\circ}}$
4887.538	1		20454.486	16554_6_ – $37008_{0}^{{}^{\circ}}$
4887.096	50	2	20456.336	17959_4_ – $38416_{3}^{{}^{\circ}}$
4886.869	50	2	20457.287	15970_3_ – $36427_{3}^{{}^{\circ}}$
4886.014	3b		20460.867	$18069_{3}^{{}^{\circ}}$ – 38529_3_
4885.825	41		20461.658	$16346_{4}^{{}^{\circ}}$ – 36808_3_
4885.565	5b	1	20462.747	$10783_{2}^{{}^{\circ}}$ – 31245_2_
4885.290	2		20463.899	$17224_{2}^{{}^{\circ}}$ – 37688_3_
4885.241	3		20464.104	$19588_{0}^{{}^{\circ}}$ – 40052_4_
4884.549	50	2	20467.003	$19227_{6}^{{}^{\circ}}$ – 39694_5_
4883.663	2		20470.716	$15618_{3}^{{}^{\circ}}$ – 36089_4_
4883.490	3	1	20471.441	13088_3_ – $33560_{4}^{{}^{\circ}}$
4882.894	5		20473.940	$18809_{4}^{{}^{\circ}}$ – 39283_4_
4882.328	3		20476.313	13847_2_ – $34324_{2}^{{}^{\circ}}$
4882.328	3		20476.313	$18053_{4}^{{}^{\circ}}$ – 38529_3_
4881.853	75	2	20478.306	8111_4_ – $28589_{3}^{{}^{\circ}}$
4881.597	50	2	20479.380	11601_1_ – $32080_{1}^{{}^{\circ}}$
4881.204	75	2	20481.029	4961_4_ – $25442_{3}^{{}^{\circ}}$
4881.070	3		20481.591	$17411_{3}^{{}^{\circ}}$ – 37892_4_
4880.912	50b	2	20482.254	11802_2_ – $32285_{3}^{{}^{\circ}}$
4880.859	25b	2	20482.476	$10783_{2}^{{}^{\circ}}$ – 31265_3_
4880.190	15b	1	20485.284	16351_0_ – $36837_{1}^{{}^{\circ}}$
4880.010	5	1	20486.040	$13945_{3}^{{}^{\circ}}$ – 34431_3_
4878.733	300b	8	20491.402	2558_0_ – $23049_{1}^{{}^{\circ}}$
4878.009	100	3	20494.443	3687_2_ – $24182_{2}^{{}^{\circ}}$
4877.265	20	1	20497.569	15863_2_ – $36361_{1}^{{}^{\circ}}$
4876.856	4		20499.288	$13945_{3}^{{}^{\circ}}$ – 34444_2_
4876.495	100	5	20500.806	$14206_{4}^{{}^{\circ}}$ – 34707_3_
4876.244	50	2	20501.861	$13945_{3}^{{}^{\circ}}$ – 34447_4_
4876.142	3		20502.290	13297_4_ – $33799_{4}^{{}^{\circ}}$
4876.067	4		20502.605	13088_3_ – $33591_{3}^{{}^{\circ}}$
4875.677	4		20504.245	7280_2_ – $27784_{2}^{{}^{\circ}}$
4874.365	150	10	20509.764	8800_4_ – $29310_{4}^{{}^{\circ}}$
4873.855	2		20511.910	$18809_{4}^{{}^{\circ}}$ – 39321_3_
4872.922	300r	25b	20515.838	3865_1_ – $24381_{2}^{{}^{\circ}}$
4872.229	10b	1	20518.756	15970_3_ – $36488_{2}^{{}^{\circ}}$
4872.030	100	4	20519.594	$16783_{4}^{{}^{\circ}}$ – 37303_4_
4871.558	3		20521.582	$21165_{3}^{{}^{\circ}}$ – 41686_3_
4871.286	50	2	20522.728	0_2_ – $20522_{2}^{{}^{\circ}}$
4868.882	150	10	20532.860	$11241_{3}^{{}^{\circ}}$ – 31774_3_
4868.636	8h	1	20533.898	$18069_{3}^{{}^{\circ}}$ – 38602_2_
4867.502	5b	1b	20538.682	$16346_{4}^{{}^{\circ}}$ – 36885_4_
4866.740	2		20541.898	$18809_{4}^{{}^{\circ}}$ – 39351_5_
4865.477	300r	150b	20547.230	$7795_{4}^{{}^{\circ}}$ – 28342_5_
4865.415	75b	15b	20547.492	13297_4_ – $33844_{0}^{{}^{\circ}}$
4864.985	2		20549.308	$19503_{3}^{{}^{\circ}}$ – 40052_4_
4864.789	2		20550.136	5563_1_ – $26113_{2}^{{}^{\circ}}$
4864.433	8	2	20551.640	17398_3_ – $37950_{4}^{{}^{\circ}}$
4864.357	3	1	20551.961	$11241_{3}^{{}^{\circ}}$ – 31793_4_
4862.962	4		20557.856	$16217_{2}^{{}^{\circ}}$ – 36775_3_
4862.597	1		20559.399	11601_1_ – $32160_{2}^{{}^{\circ}}$
4861.720	75b	20h	20563.108	$7795_{4}^{{}^{\circ}}$ – 28358_3_
4861.635	50	2	20563.467	15863_2_ – $36427_{3}^{{}^{\circ}}$
4861.223	300r	50	20565.210	8111_4_ – $28676_{3}^{{}^{\circ}}$
4860.738	3	1	20567.262	18431_3_ – $38998_{3}^{{}^{\circ}}$
4860.173	5	2	20569.653	15493_4_ – $36062_{4}^{{}^{\circ}}$
4857.949	5	1	20579.070	12847_3_ – $33427_{2}^{{}^{\circ}}$
4857.536	100	15	20580.819	$14465_{2}^{{}^{\circ}}$ – 35046_1_
4857.536	100	15	20580.819	15493_4_ – $36074_{3}^{{}^{\circ}}$
4852.868	200	50	20600.616	$16217_{2}^{{}^{\circ}}$ – 36818_2_
4851.676	5	2	20605.677	$17501_{0}^{{}^{\circ}}$ – 38106_5_
4849.861	50	15	20613.389	4961_4_ – $25575_{4}^{{}^{\circ}}$
4849.417	2		20615.276	$10526_{3}^{{}^{\circ}}$ – 31141_3_
4848.363	400r	150	20619.758	3687_2_ – $24307_{2}^{{}^{\circ}}$
4848.216	20	8	20620.383	17959_4_ – $38580_{4}^{{}^{\circ}}$
4847.330	150	15	20624.152	$14465_{2}^{{}^{\circ}}$ – 35089_3_
4847.144	20	2	20624.943	15863_2_ – $36488_{2}^{{}^{\circ}}$
4845.990	1		20629.854	13088_3_ – $33718_{2}^{{}^{\circ}}$
4845.161	100	10	20633.384	6362_2_ – $26995_{3}^{{}^{\circ}}$
4844.929	75	5	20634.372	$19948_{4}^{{}^{\circ}}$ – 40582_5_
4844.612	5b		20635.722	$21077_{0}^{{}^{\circ}}$ – 41712_5_
4843.938	100b	50b	20638.594	7502_3_ – $28140_{4}^{{}^{\circ}}$
4843.876	75b	10b	20638.858	$8243_{2}^{{}^{\circ}}$ – 28882_2_
4843.103	3	1	20642.152	$14206_{4}^{{}^{\circ}}$ – 34849_5_
4840.843	400r	75	20651.789	2869_3_ – $23521_{3}^{{}^{\circ}}$
4839.780	2		20656.324	$20922_{2}^{{}^{\circ}}$ – 41578_2_
4839.674	5	2b	20656.777	$13175_{4}^{{}^{\circ}}$ – 33831_4_
4837.903	1		20664.339	$20566_{4}^{{}^{\circ}}$ – 41230_4_
4837.409	40	4	20666.449	$14206_{4}^{{}^{\circ}}$ – 34873_4_
4837.245	20	3	20667.150	$14206_{4}^{{}^{\circ}}$ – 34874_3_
4836.091	3		20672.081	$20214_{3}^{{}^{\circ}}$ – 40886_2_
4834.632	2		20678.319	$15618_{3}^{{}^{\circ}}$ – 36297_4_
4834.537	3	1	20678.726	$21077_{0}^{{}^{\circ}}$ – 41755_4_
4833.674	20s	4s	20682.418	14204_5_ – $34886_{0}^{{}^{\circ}}$
4833.179	200b	50	20684.536	$14465_{2}^{{}^{\circ}}$ – 35149_2_
4833.112	50b	8	20684.823	15493_4_ – $36178_{4}^{{}^{\circ}}$
4832.423	51	21	20687.772	$11241_{3}^{{}^{\circ}}$ – 31929_3_
4831.767	1		20690.580	17398_3_ – $38088_{2}^{{}^{\circ}}$
4831.597	300r	150	20691.309	$8243_{2}^{{}^{\circ}}$ – 28934_3_
4831.121	500r	200	20693.347	3687_2_ – $24381_{2}^{{}^{\circ}}$
4830.206	1		20697.267	$19503_{3}^{{}^{\circ}}$ – 40200_3_
4829.796	50	8	20699.024	6362_2_ – $27061_{2}^{{}^{\circ}}$
4829.407	5	2	20700.691	11802_2_ – $32503_{2}^{{}^{\circ}}$
4828.658	20	4	20703.902	13297_4_ – $34001_{4}^{{}^{\circ}}$
4828.246	1		20705.669	17959_4_ – $38665_{0}^{{}^{\circ}}$
4826.700	200b	50b	20712.301	$30517_{0}^{{}^{\circ}}$ – 9804_5_
4826.637	50b	15b	20712.571	9804_5_ – $30517_{4}^{{}^{\circ}}$
4826.008	50	4	20715.271	18549_2_ – $39264_{3}^{{}^{\circ}}$
4825.553	5	1	20717.224	$15618_{3}^{{}^{\circ}}$ – 36336_3_
4824.736	1		20720.732	$15490_{0}^{{}^{\circ}}$ – 36210_5_
4823.997	200r	100h	20723.906	5563_1_ – $26287_{1}^{{}^{\circ}}$
4823.604	300r	75	20725.595	6362_2_ – $27087_{1}^{{}^{\circ}}$
4823.122	5b	1	20727.666	$10414_{4}^{{}^{\circ}}$ – 31141_3_
4822.855	300r	75	20728.814	4961_4_ – $25690_{0}^{{}^{\circ}}$
4821.862	15	2	20733.082	3687_2_ – $24421_{3}^{{}^{\circ}}$
4821.587	150	25	20734.265	2869_3_ – $23603_{2}^{{}^{\circ}}$
4821.387	50	15	20735.125	$14481_{6}^{{}^{\circ}}$ – 35216_5_
4820.884	100	200	20737.288	0_2_ – $20737_{1}^{{}^{\circ}}$
4820.788	2h	1	20737.701	$16783_{4}^{{}^{\circ}}$ – 37521_3_
4820.463	150b	40h	20739.099	$10526_{3}^{{}^{\circ}}$ – 31265_3_
4819.508	10	3	20743.209	12847_3_ – $33591_{3}^{{}^{\circ}}$
4819.191	150	15	20744.573	7280_2_ – $28024_{1}^{{}^{\circ}}$
4818.294	5	2	20748.435	$15736_{1}^{{}^{\circ}}$ – 36485_2_
4818.102	4	2	20749.262	$18011_{0}^{{}^{\circ}}$ – 38760_4_
4817.017	75	8	20753.935	$11241_{3}^{{}^{\circ}}$ – 31995_4_
4816.566	1		20755.879	$17847_{2}^{{}^{\circ}}$ – 38602_2_
4815.046	5	2	20762.431	$13945_{3}^{{}^{\circ}}$ – 34707_3_
4814.919	15	3	20762.978	14226_0_ – $34989_{1}^{{}^{\circ}}$
4814.553	2		20764.557	$20922_{2}^{{}^{\circ}}$ – 41686_3_
4813.893	200	100	20767.404	$7795_{4}^{{}^{\circ}}$ – 28562_4_
4813.499	1		20769.103	18699_2_ – $39468_{3}^{{}^{\circ}}$
4813.004	75	15	20771.239	$11241_{3}^{{}^{\circ}}$ – 32012_4_
4812.712	1		20772.500	11802_2_ – $32575_{2}^{{}^{\circ}}$
4812.373	150	25	20773.963	8111_4_ – $28884_{3}^{{}^{\circ}}$
4810.519	8	4	20781.969	15493_4_ – $36275_{0}^{{}^{\circ}}$
4809.615	200	50	20785.875	2869_3_ – $23655_{4}^{{}^{\circ}}$
4808.764	5	2	20789.554	$20566_{4}^{{}^{\circ}}$ – 41356_5_
4808.134	500r	150	20792.278	4961_4_ – $25753_{0}^{{}^{\circ}}$
4807.550	4	2	20794.803	$20566_{4}^{{}^{\circ}}$ – 41361_3_
4806.459	5	2	20799.524	$11241_{3}^{{}^{\circ}}$ – 32041_2_
4806.355	5	1	20799.974	5563_1_ – $26363_{2}^{{}^{\circ}}$
4806.248	3	1	20800.437	$10526_{3}^{{}^{\circ}}$ – 31326_4_
4805.244	15	3	20804.782	15863_2_ – $36668_{2}^{{}^{\circ}}$
4802.670	10	3	20815.933	$11197_{0}^{{}^{\circ}}$ – 32012_4_
4801.951	2		20819.050	$14481_{6}^{{}^{\circ}}$ – 35300_5_
4801.349	4	1	20821.660	8111_4_ – $28932_{4}^{{}^{\circ}}$
4801.054	25	4	20822.939	19273_2_ – $40096_{2}^{{}^{\circ}}$
4799.670	3	1	20828.943	17959_4_ – $38788_{3}^{{}^{\circ}}$
4798.734	4	2	20833.006	18431_3_ – $39264_{3}^{{}^{\circ}}$
4797.497	75	50	20838.378	$19588_{0}^{{}^{\circ}}$ – 40426_4_
4796.667	3	1	20841.983	$14032_{2}^{{}^{\circ}}$ – 34874_3_
4795.913	150	75	20845.260	7502_3_ – $28347_{2}^{{}^{\circ}}$
4795.289	3	1	20847.973	11802_2_ – $32650_{1}^{{}^{\circ}}$
4794.474	2		20851.516	$10414_{4}^{{}^{\circ}}$ – 31265_3_
4793.285	8		20856.689	13847_2_ – $34704_{3}^{{}^{\circ}}$
4793.246	1001	25	20856.858	$10414_{4}^{{}^{\circ}}$ – 31271_5_
4792.007	3		20862.251	$21165_{3}^{{}^{\circ}}$ – 42027_2_
4791.921	5	1	20862.625	11802_2_ – $32665_{1}^{{}^{\circ}}$
4791.362	8	3	20865.059	$16346_{4}^{{}^{\circ}}$ – 37211_5_
4789.387	400r	75	20873.663	3687_2_ – $24561_{3}^{{}^{\circ}}$
4788.678	25	5	20876.754	14204_5_ – $35081_{0}^{{}^{\circ}}$
4788.542	3	1	20877.347	$16217_{2}^{{}^{\circ}}$ – 37094_2_
4788.472	2		20877.652	13847_2_ – $34725_{3}^{{}^{\circ}}$
4788.079	1		20879.366	13088_3_ – $33967_{3}^{{}^{\circ}}$
4787.148	150	25	20883.426	2869_3_ – $23752_{2}^{{}^{\circ}}$
4786.914	5	1	20884.447	$18809_{4}^{{}^{\circ}}$ – 39694_5_
4786.724	75	5	20885.276	13297_4_ – $34182_{0}^{{}^{\circ}}$
4786.532	200	50	20886.114	8800_4_ – $29686_{3}^{{}^{\circ}}$
4785.780	5	2	20889.395	15493_4_ – $36382_{4}^{{}^{\circ}}$
4785.007	1		20892.770	19532_4_ – $40425_{4}^{{}^{\circ}}$
4784.163	2h		20896.456	17073_1_ – $37970_{2}^{{}^{\circ}}$
4784.038	150	20	20897.002	$15618_{3}^{{}^{\circ}}$ – 36515_3_
4783.864	1501	10b	20897.762	6362_2_ – $27260_{3}^{{}^{\circ}}$
4783.605	2b		20898.893	15863_2_ – $36762_{3}^{{}^{\circ}}$
4782.936	5	2	20901.816	15970_3_ – $36871_{2}^{{}^{\circ}}$
4781.671	8	2	20907.346	$18930_{3}^{{}^{\circ}}$ – 39837_3_
4781.527	100	5	20907.976	17398_3_ – $38306_{4}^{{}^{\circ}}$
4781.363	2		20908.693	$20322_{0}^{{}^{\circ}}$ – 41230_4_
4780.751	75	8	20911.369	8800_4_ – $29711_{0}^{{}^{\circ}}$
4780.633	3		20911.885	18699_2_ – $39611_{3}^{{}^{\circ}}$
4780.427	150	25	20912.786	13088_3_ – $34001_{4}^{{}^{\circ}}$
4779.728	50	5	20915.845	4961_4_ – $25877_{4}^{{}^{\circ}}$
4779.661	4	1	20916.138	13962_1_ – $34878_{1}^{{}^{\circ}}$
4779.566	2b		20916.554	$15736_{1}^{{}^{\circ}}$ – 36653_2_
4778.453	5	2	20921.425	$17224_{2}^{{}^{\circ}}$ – 38145_3_
4778.294	400r	50	20922.122	0_2_ – $20922_{2}^{{}^{\circ}}$
4778.021	2		20923.317	2558_0_ – $23481_{1}^{{}^{\circ}}$
4777.421	1		20925.945	20054_2_ – $40980_{3}^{{}^{\circ}}$
4777.192	300	75	20926.948	14204_5_ – $35131_{4}^{{}^{\circ}}$
4775.793	100	15	20933.078	8800_4_ – $29733_{0}^{{}^{\circ}}$
4775.313	1001	5b	20935.182	6362_2_ – $27297_{1}^{{}^{\circ}}$
4774.275	40b		20939.734	8111_4_ – $29050_{0}^{{}^{\circ}}$
4773.241	100	8	20944.270	8800_4_ – $29744_{3}^{{}^{\circ}}$
4772.962	3		20945.494	$18011_{0}^{{}^{\circ}}$ – 38956_4_
4771.533	4	1	20951.767	12847_3_ – $33799_{4}^{{}^{\circ}}$
4771.330	8	2	20952.658	12847_3_ – $33800_{3}^{{}^{\circ}}$
4770.796	15	1	20955.003	6362_2_ – $27317_{3}^{{}^{\circ}}$
4770.440	10b	3	20956.567	$18053_{4}^{{}^{\circ}}$ – 39010_3_
4770.390	15b	3	20956.787	$16346_{4}^{{}^{\circ}}$ – 37303_4_
4769.543	50	3	20960.508	$16346_{4}^{{}^{\circ}}$ – 37307_3_
4766.600	300r	100	20973.450	3865_1_ – $24838_{1}^{{}^{\circ}}$
4766.382	2		20974.409	11601_1_ – $32575_{2}^{{}^{\circ}}$
4766.098	1		20975.659	$14465_{2}^{{}^{\circ}}$ – 35440_3_
4765.593	100b	100	20977.881	7502_3_ – $28480_{4}^{{}^{\circ}}$
4763.963	4	1	20985.059	$12114_{2}^{{}^{\circ}}$ – 33099_3_
4762.805	2		20990.161	$19503_{3}^{{}^{\circ}}$ – 40493_3_
4762.302	8	2	20992.378	$13175_{4}^{{}^{\circ}}$ – 34167_3_
4759.687	75	10	21003.911	13847_2_ – $34851_{2}^{{}^{\circ}}$
4759.441	2		21004.997	$11197_{0}^{{}^{\circ}}$ – 32202_5_
4758.286	50	15	21010.095	$14206_{4}^{{}^{\circ}}$ – 35216_5_
4758.150	100	75	21010.696	$10526_{3}^{{}^{\circ}}$ – 31537_3_
4758.075	50	15	21011.027	7502_3_ – $28513_{2}^{{}^{\circ}}$
4757.415	15	2	21013.942	$14032_{2}^{{}^{\circ}}$ – 35046_1_
4757.133	50	5	21015.187	17073_1_ – $38088_{2}^{{}^{\circ}}$
4756.699	100	25	21017.105	16554_6_ – $37571_{0}^{{}^{\circ}}$
4756.532	75		21017.843	17398_3_ – $38416_{3}^{{}^{\circ}}$
4753.581	21		21030.890	13847_2_ – $34878_{1}^{{}^{\circ}}$
4752.900	10	3	21033.904	$20322_{0}^{{}^{\circ}}$ – 41356_5_
4752.588	50b	10b	21035.284	$16783_{4}^{{}^{\circ}}$ – 37819_4_
4752.199	2		21037.006	18431_3_ – $39468_{3}^{{}^{\circ}}$
4749.968	300b	50	21046.887	8111_4_ – $29157_{3}^{{}^{\circ}}$
4749.892	20	4	21047.224	13297_4_ – $34344_{4}^{{}^{\circ}}$
4749.795	25	3	21047.654	2869_3_ – $23916_{4}^{{}^{\circ}}$
4749.466	3b	1	21049.111	21575_2_ – $42624_{1}^{{}^{\circ}}$
4749.287	5		21049.905	11601_1_ – $32650_{1}^{{}^{\circ}}$
4749.200	400r	150	21050.290	$7795_{4}^{{}^{\circ}}$ – 28845_4_
4747.617	100	4	21057.309	$14032_{2}^{{}^{\circ}}$ – 35089_3_
4745.629	8	2	21066.130	$18614_{1}^{{}^{\circ}}$ – 39680_2_
4745.331	200b	25b	21067.453	7280_2_ – $28347_{2}^{{}^{\circ}}$
4744.838	5	1	21069.642	14204_5_ – $35273_{0}^{{}^{\circ}}$
4744.797	5	2	21069.824	$19817_{1}^{{}^{\circ}}$ – 40886_2_
4744.706	3		21070.228	$13945_{3}^{{}^{\circ}}$ – 35015_2_
4744.516	2		21071.072	15970_3_ – $37041_{4}^{{}^{\circ}}$
4742.114	200	8	21081.745	3687_2_ – $24769_{3}^{{}^{\circ}}$
4741.301	150	40	21085.360	6362_2_ – $27447_{2}^{{}^{\circ}}$
4740.951	200b	15	21086.916	4961_4_ – $26048_{4}^{{}^{\circ}}$
4740.929	100b	8	21087.014	7502_3_ – $28589_{3}^{{}^{\circ}}$
4740.332	15	3	21089.670	$16217_{2}^{{}^{\circ}}$ – 37307_3_
4739.674	300	40	21092.598	7280_2_ – $28372_{1}^{{}^{\circ}}$
4739.353	5	2	21094.026	$14206_{4}^{{}^{\circ}}$ – 35300_5_
4738.839	8	2	21096.314	$21539_{4}^{{}^{\circ}}$ – 42635_5_
4737.915	150	15	21100.428	$14481_{6}^{{}^{\circ}}$ – 35582_5_
4737.604	101	2	21101.813	14226_0_ – $35328_{1}^{{}^{\circ}}$
4737.529	20	3	21102.148	17959_4_ – $39062_{0}^{{}^{\circ}}$
4736.523	10	2	21106.629	$16783_{4}^{{}^{\circ}}$ – 37890_5_
4735.995	200	20	21108.982	$16783_{4}^{{}^{\circ}}$ – 37892_4_
4735.474	4	1	21111.305	$14465_{2}^{{}^{\circ}}$ – 35576_3_
4734.046	200	10	21117.673	$14032_{2}^{{}^{\circ}}$ – 35149_2_
4733.536	2		21119.948	12847_3_ – $33967_{3}^{{}^{\circ}}$
4733.504	1		21120.091	$20566_{4}^{{}^{\circ}}$ – 41686_3_
4733.224	1		21121.340	$18053_{4}^{{}^{\circ}}$ – 39174_3_
4732.826	4	1	21123.116	$10414_{4}^{{}^{\circ}}$ – 31537_3_
4732.103	40	4	21126.344	$13175_{4}^{{}^{\circ}}$ – 34301_4_
4731.192	20	3	21130.411	$15166_{3}^{{}^{\circ}}$ – 36297_4_
4730.657	150	8	21132.801	13297_4_ – $34430_{3}^{{}^{\circ}}$
4730.089	40	5	21135.339	4961_4_ – $26096_{3}^{{}^{\circ}}$
4729.128	400b	100b	21139.634	$7795_{4}^{{}^{\circ}}$ – 28934_3_
4728.985	50	8	21140.273	17166_5_ – $38306_{4}^{{}^{\circ}}$
4728.591	40	3	21142.034	13847_2_ – $34989_{1}^{{}^{\circ}}$
4728.133	300	25d	21144.082	$13945_{3}^{{}^{\circ}}$ – 35089_3_
4727.619	3h		21146.381	$10783_{2}^{{}^{\circ}}$ – 31929_3_
4727.440	75	4	21147.182	14204_5_ – $35351_{4}^{{}^{\circ}}$
4726.598	5	2	21150.949	3687_2_ – $24838_{1}^{{}^{\circ}}$
4726.441	8		21151.651	11802_2_ – $32954_{2}^{{}^{\circ}}$
4726.054	4		21153.383	12847_3_ – $34001_{4}^{{}^{\circ}}$
4725.384	8	2	21156.383	$15618_{3}^{{}^{\circ}}$ – 36775_3_
4724.691	2		21159.486	$12114_{2}^{{}^{\circ}}$ – 33273_1_
4724.292	2		21161.273	$19039_{2}^{{}^{\circ}}$ – 40200_3_
4723.438	800r	200	21165.099	0_2_ – $21165_{3}^{{}^{\circ}}$
4722.499	75	5	21169.307	$15166_{3}^{{}^{\circ}}$ – 36336_3_
4722.089	300	50	21171.145	7502_3_ – $28673_{2}^{{}^{\circ}}$
4721.456	3		21173.983	7502_3_ – $28676_{3}^{{}^{\circ}}$
4721.276	200	15	21174.791	$8243_{2}^{{}^{\circ}}$ – 29418_2_
4720.780	150	15	21177.015	$14243_{1}^{{}^{\circ}}$ – 35421_1_
4720.457	75	8	21178.465	7502_3_ – $28680_{4}^{{}^{\circ}}$
4720.338	3	1	21178.998	15970_3_ – $37149_{2}^{{}^{\circ}}$
4720.160	50	3	21179.797	18431_3_ – $39611_{3}^{{}^{\circ}}$
4719.692	150	5	21181.897	17398_3_ – $38580_{4}^{{}^{\circ}}$
4719.441	20	2	21183.024	2558_0_ – $23741_{1}^{{}^{\circ}}$
4718.051	100	5	21189.264	$20566_{4}^{{}^{\circ}}$ – 41755_4_
4717.487	3	1	21191.798	$17411_{3}^{{}^{\circ}}$ – 38602_2_
4717.390	20	2	21192.233	3687_2_ – $24880_{1}^{{}^{\circ}}$
4716.862	5b		21194.605	13297_4_ – $34492_{4}^{{}^{\circ}}$
4716.849	8b	1	21194.664	$12114_{2}^{{}^{\circ}}$ – 33309_2_
4715.955	5b	1	21198.682	$18053_{4}^{{}^{\circ}}$ – 39252_5_
4715.856	8	1	21199.127	$15618_{3}^{{}^{\circ}}$ – 36818_2_
4714.673	150	8	21204.446	$13945_{3}^{{}^{\circ}}$ – 35149_2_
4713.789	25	4b	21208.422	$16783_{4}^{{}^{\circ}}$ – 37992_4_
4712.839	100	8	21212.698	$10414_{4}^{{}^{\circ}}$ – 31626_5_
4712.481	300b	100	21214.309	9804_5_ – $31019_{4}^{{}^{\circ}}$
4712.004	50	4	21216.456	$11241_{3}^{{}^{\circ}}$ – 32458_4_
4711.917	5	1	21216.848	8800_4_ – $30017_{3}^{{}^{\circ}}$
4711.417	75	5	21219.100	17166_5_ – $38385_{0}^{{}^{\circ}}$
4708.948	3	1	21230.225	$18053_{4}^{{}^{\circ}}$ – 39283_4_
4708.294	200	25	21233.174	7280_2_ – $28513_{2}^{{}^{\circ}}$
4707.772	25b	3	21235.529	13088_3_ – $34324_{2}^{{}^{\circ}}$
4706.691	10	2	21240.406	11802_2_ – $33043_{3}^{{}^{\circ}}$
4706.575	100	5	21240.929	$18011_{0}^{{}^{\circ}}$ – 39252_5_
4705.873	8	1	21244.098	19532_4_ – $40776_{4}^{{}^{\circ}}$
4704.997	5	1	21248.053	$10526_{3}^{{}^{\circ}}$ – 31774_3_
4704.935	8	2	21248.333	$17354_{1}^{{}^{\circ}}$ – 38602_2_
4703.990	500r	200	21252.602	0_2_ – $21252_{2}^{{}^{\circ}}$
4703.213	50	2	21256.112	13088_3_ – $34344_{4}^{{}^{\circ}}$
4703.190	50	5	21256.216	$13175_{4}^{{}^{\circ}}$ – 34431_3_
4702.778	15	2	21258.079	$10783_{2}^{{}^{\circ}}$ – 32041_2_
4702.591	25	3	21258.924	$15490_{0}^{{}^{\circ}}$ – 36749_6_
4702.475	25	3	21259.449	$17501_{0}^{{}^{\circ}}$ – 38760_4_
4701.388	20	4	21264.364	16351_0_ – $37616_{1}^{{}^{\circ}}$
4700.959	50	4	21266.304	$15618_{3}^{{}^{\circ}}$ – 36885_4_
4700.772	200	8	21267.150	$10526_{3}^{{}^{\circ}}$ – 31793_4_
4700.655	2		21267.679	19713_3_ – $40980_{3}^{{}^{\circ}}$
4700.234	8	1	21269.584	15493_4_ – $36762_{3}^{{}^{\circ}}$
4699.602	10	2	21272.445	$18011_{0}^{{}^{\circ}}$ – 39283_4_
4698.732	25b	3	21276.383	$12114_{2}^{{}^{\circ}}$ – 33390_1_
4697.600	10	2	21281.510	17073_1_ – $38355_{2}^{{}^{\circ}}$
4697.359	2		21282.602	$19948_{4}^{{}^{\circ}}$ – 41230_4_
4697.212	10	3	21283.268	13088_3_ – $34371_{2}^{{}^{\circ}}$
4696.697	15b	4b	21285.602	13297_4_ – $34583_{3}^{{}^{\circ}}$
4696.667	25b	8b	21285.738	$13175_{4}^{{}^{\circ}}$ – 34460_5_
4696.588	2		21286.096	18699_2_ – $39985_{3}^{{}^{\circ}}$
4695.705	1		21290.099	$20737_{1}^{{}^{\circ}}$ – 42027_2_
4695.456	200	50	21291.228	$15490_{0}^{{}^{\circ}}$ – 36781_5_
4695.039	300r	100	21293.119	3687_2_ – $24981_{3}^{{}^{\circ}}$
4694.537	5	1	21295.396	15863_2_ – $37159_{1}^{{}^{\circ}}$
4694.478	15	3b	21295.663	15863_2_ – $37159_{3}^{{}^{\circ}}$
4692.465	3	1	21304.798	17959_4_ – $39264_{3}^{{}^{\circ}}$
4691.883	100	8	21307.441	15493_4_ – $36800_{4}^{{}^{\circ}}$
4691.631	300b	50b	21308.586	6362_2_ – $27670_{3}^{{}^{\circ}}$
4691.603	50b	8b	21308.713	$7795_{4}^{{}^{\circ}}$ – 29104_4_
4691.500	100	15	21309.181	7280_2_ – $28589_{3}^{{}^{\circ}}$
4691.346	100	15	21309.880	$11241_{3}^{{}^{\circ}}$ – 32551_3_
4690.894	15	2	21311.934	6362_2_ – $27674_{2}^{{}^{\circ}}$
4690.623	300	40b	21313.165	2869_3_ – $24182_{2}^{{}^{\circ}}$
4689.532	4		21318.123	$14481_{6}^{{}^{\circ}}$ – 35799_6_
4689.451	50	5	21318.491	$15166_{3}^{{}^{\circ}}$ – 36485_2_
4689.252	2001	50b	21319.396	5563_1_ – $26882_{0}^{{}^{\circ}}$
4688.458	5h	2	21323.006	$16783_{4}^{{}^{\circ}}$ – 38106_5_
4686.195	500r	50	21333.303	2869_3_ – $24202_{4}^{{}^{\circ}}$
4684.634	40	3	21340.412	$18011_{0}^{{}^{\circ}}$ – 39351_5_
4684.362	50b	3	21341.651	13088_3_ – $34430_{3}^{{}^{\circ}}$
4683.349	200	20	21346.267	$7795_{4}^{{}^{\circ}}$ – 29141_5_
4682.728	75	5	21349.098	$15166_{3}^{{}^{\circ}}$ – 36515_3_
4682.662	3b		21349.399	$17411_{3}^{{}^{\circ}}$ – 38760_4_
4682.229	150	15	21351.373	17959_4_ – $3931_{4}^{{}^{\circ}}$
4681.743	1		21353.590	11601_1_ – $32954_{2}^{{}^{\circ}}$
4680.588	50b	10b	21358.859	11802_2_ – $33161_{1}^{{}^{\circ}}$
4680.387	100	8	21359.776	8800_4_ – $30160_{4}^{{}^{\circ}}$
4680.235	200	15	21360.470	$10414_{4}^{{}^{\circ}}$ – 31774_3_
4679.919	3		21361.912	$16783_{4}^{{}^{\circ}}$ – 38145_3_
4679.575	4	2	21363.482	$20214_{3}^{{}^{\circ}}$ – 41578_2_
4679.390	3		21364.327	$20922_{2}^{{}^{\circ}}$ – 42286_3_
4679.001	20	1	21366.103	13962_1_ – $35328_{1}^{{}^{\circ}}$
4678.358	5	2	21369.040	7280_2_ – $28649_{1}^{{}^{\circ}}$
4678.233	150	20b	21369.611	$14206_{4}^{{}^{\circ}}$ – 35576_3_
4678.145	8	2	21370.012	$19516_{2}^{{}^{\circ}}$ – 40886_2_
4676.971	150	25b	21375.377	$14206_{4}^{{}^{\circ}}$ – 35582_5_
4676.248	2		21378.681	$17224_{2}^{{}^{\circ}}$ – 38602_2_
4676.248	2		21378.681	$17224_{2}^{{}^{\circ}}$ – 38602_2_
4676.056	500r	150	21379.559	$10414_{4}^{{}^{\circ}}$ – 31793_4_
4675.375	200	15	21382.673	7502_3_ – $28884_{3}^{{}^{\circ}}$
4675.115	3	2	21383.862	$19503_{3}^{{}^{\circ}}$ – 40886_2_
4674.006	3		21388.936	$14032_{2}^{{}^{\circ}}$ – 35421_1_
4673.805	2		21389.856	9804_5_ – $31194_{4}^{{}^{\circ}}$
4673.661	800r	150	21390.515	2869_3_ – $24259_{4}^{{}^{\circ}}$
4673.042	200	25	21393.348	7280_2_ – $28673_{2}^{{}^{\circ}}$
4672.632	8	1	21395.225	$15490_{0}^{{}^{\circ}}$ – 36885_4_
4672.436	50	20	21396.123	7280_2_ – $28676_{3}^{{}^{\circ}}$
4672.370	2		21396.425	$17411_{3}^{{}^{\circ}}$ – 38807_2_
4672.332	5	1	21396.599	18699_2_ – $40096_{2}^{{}^{\circ}}$
4671.091	2		21402.284	$16783_{4}^{{}^{\circ}}$ – 38186_4_
4670.942	50	3	21402.966	$10526_{3}^{{}^{\circ}}$ – 31929_3_
4670.832	10	1	21403.471	13088_3_ – $34492_{4}^{{}^{\circ}}$
4670.059	15b	1	21407.013	13297_4_ – $34704_{3}^{{}^{\circ}}$
4669.984	500r	100	21407.357	$8243_{2}^{{}^{\circ}}$ – 29650_2_
4669.665	75	4	21408.819	$14032_{2}^{{}^{\circ}}$ – 35440_3_
4668.492	3		21414.198	17166_5_ – $38580_{4}^{{}^{\circ}}$
4668.172	800r	150	21415.666	7502_3_ – $28917_{2}^{{}^{\circ}}$
4666.797	500r	150	21421.976	6362_2_ – $27784_{2}^{{}^{\circ}}$
4666.515	200	8	21423.270	4961_4_ – $26384_{4}^{{}^{\circ}}$
4665.713	40	3	21426.953	$14206_{4}^{{}^{\circ}}$ – 35633_4_
4665.483	5	1	21428.009	13297_4_ – $34725_{3}^{{}^{\circ}}$
4664.968	100	4	21430.375	7502_3_ – $28932_{4}^{{}^{\circ}}$
4664.784	25	2	21431.220	$11877_{1}^{{}^{\circ}}$ – 33309_2_
4663.670	5	2	21436.339	18549_2_ – $39985_{3}^{{}^{\circ}}$
4663.202	300r	10	21438.490	2869_3_ – $24307_{2}^{{}^{\circ}}$
4662.590	20	2	21441.304	3865_1_ – $25306_{2}^{{}^{\circ}}$
4660.254	20	2	21452.052	14226_0_ – $35678_{1}^{{}^{\circ}}$
4660.254	20	2	21452.052	$17501_{0}^{{}^{\circ}}$ – 38953_5_
4660.039	4	1	21453.042	$17354_{1}^{{}^{\circ}}$ – 38807_2_
4659.568	200	40	21455.210	8800_4_ – $30255_{3}^{{}^{\circ}}$
4659.458	5		21455.717	$17501_{0}^{{}^{\circ}}$ – 38956_4_
4657.033	1		21466.889	$15618_{3}^{{}^{\circ}}$ – 37085_4_
4656.548	50	4	21469.125	$10526_{3}^{{}^{\circ}}$ – 31995_4_
4655.974	3		21471.771	$20214_{3}^{{}^{\circ}}$ – 41686_3_
4655.818	4	1	21472.491	$16346_{4}^{{}^{\circ}}$ – 37819_4_
4655.328	1		21474.751	$17847_{2}^{{}^{\circ}}$ – 39321_3_
4655.210	100	20	21475.295	18431_3_ – $39906_{4}^{{}^{\circ}}$
4655.030	75	10	21476.126	12847_3_ – $34324_{2}^{{}^{\circ}}$
4654.002	50	3	21480.869	13847_2_ – $35328_{1}^{{}^{\circ}}$
4652.743	20	2	21486.682	$15166_{3}^{{}^{\circ}}$ – 36653_2_
4651.584	75b		21492.036	11802_2_ – $33294_{3}^{{}^{\circ}}$
4651.112	75	5	21494.216	11802_2_ – $33297_{2}^{{}^{\circ}}$
4651.062	75b	4	21494.447	13088_3_ – $34583_{3}^{{}^{\circ}}$
4650.628	50	4	21496.453	$18930_{3}^{{}^{\circ}}$ – 40426_4_
4650.233	100	5	21498.279	5563_1_ – $27061_{2}^{{}^{\circ}}$
4649.975	75	8	21499.472	17166_5_ – $38665_{0}^{{}^{\circ}}$
4647.953	100	5	21508.825	$18011_{0}^{{}^{\circ}}$ – 39520_4_
4647.249	150	20	21512.083	2869_3_ – $24381_{2}^{{}^{\circ}}$
4647.249	150	20	21512.083	$15618_{3}^{{}^{\circ}}$ – 37131_3_
4647.068	50	2	21512.921	$11877_{1}^{{}^{\circ}}$ – 33390_1_
4646.685	150	15	21514.694	$10526_{3}^{{}^{\circ}}$ – 32041_2_
4646.538	75b	4	21515.375	$10414_{4}^{{}^{\circ}}$ – 31929_3_
4646.505	8b	1	21515.528	15493_4_ – $37008_{0}^{{}^{\circ}}$
4645.186	3		21521.637	18574_1_ – $40096_{2}^{{}^{\circ}}$
4644.707	150	50	21523.856	12847_3_ – $34371_{2}^{{}^{\circ}}$
4644.495	4		21524.839	5563_1_ – $27087_{1}^{{}^{\circ}}$
4643.557	100	10	21529.187	14204_5_ – $35733_{4}^{{}^{\circ}}$
4643.266	1		21530.536	$16217_{2}^{{}^{\circ}}$ – 37748_2_
4642.816	3		21532.623	$13175_{4}^{{}^{\circ}}$ – 34707_3_
4641.687	5	1	21537.860	$22399_{0}^{{}^{\circ}}$ – 43937_6_
4641.244	300b	150b	21539.916	$11241_{3}^{{}^{\circ}}$ – 32781_4_
4641.024	2		21540.937	$20214_{3}^{{}^{\circ}}$ – 41755_4_
4640.396	1		21543.852	$16346_{4}^{{}^{\circ}}$ – 37890_5_
4639.896	75	25	21546.174	$16346_{4}^{{}^{\circ}}$ – 37892_4_
4639.857	40b	5	21546.355	4961_4_ – $26508_{3}^{{}^{\circ}}$
4639.755	5		21546.828	18549_2_ – $40096_{2}^{{}^{\circ}}$
4639.520	200	50	21547.920	15493_4_ – $37041_{4}^{{}^{\circ}}$
4638.686	150	10	21551.794	2869_3_ – $24421_{3}^{{}^{\circ}}$
4638.127	150	10	21554.391	$11241_{3}^{{}^{\circ}}$ – 32796_3_
4637.191	50	2	21558.742	$21077_{0}^{{}^{\circ}}$ – 42635_5_
4636.758	4	1	21560.755	11601_1_ – $33161_{1}^{{}^{\circ}}$
4634.605	8	2	21570.771	13962_1_ – $35533_{2}^{{}^{\circ}}$
4634.227	3		21572.530	$19039_{2}^{{}^{\circ}}$ – 40611_2_
4633.765	300b	75b	21574.681	$12114_{2}^{{}^{\circ}}$ – 33689_2_
4633.620	150	8	21575.356	8111_4_ – $29686_{3}^{{}^{\circ}}$
4632.294	3		21581.532	$10414_{4}^{{}^{\circ}}$ – 31995_4_
4632.173	5b	1	21582.096	$14465_{2}^{{}^{\circ}}$ – 36047_2_
4632.140	8b	2	21582.250	12847_3_ – $34430_{3}^{{}^{\circ}}$
4632.038	5	1	21582.725	$16783_{4}^{{}^{\circ}}$ – 38366_4_
4631.901	2		21583.363	$17224_{2}^{{}^{\circ}}$ – 38807_2_
4631.644	100	50	21584.561	$11197_{0}^{{}^{\circ}}$ – 32781_4_
4631.021	50	3	21587.464	$14206_{4}^{{}^{\circ}}$ – 35794_4_
4630.640	8	1	21589.241	13297_4_ – $34886_{0}^{{}^{\circ}}$
4630.165	5		21591.455	$8243_{2}^{{}^{\circ}}$ – 29835_3_
29.199	15		21595.961	17073_1_ – $38669_{2}^{{}^{\circ}}$
4628.579	15b		21598.854	$10414_{4}^{{}^{\circ}}$ – 32012_4_
4628.558	75b	40	21598.952	$17411_{3}^{{}^{\circ}}$ – 39010_3_
4628.202	200	4	21600.613	8111_4_ – $29711_{0}^{{}^{\circ}}$
4627.503	3		21603.876	$20423_{1}^{{}^{\circ}}$ – 42027_2_
4627.298	200	20	21604.833	7280_2_ – $28884_{3}^{{}^{\circ}}$
4626.521	5	1	21608.461	$15166_{3}^{{}^{\circ}}$ – 36775_3_
4625.893	2		21611.395	$18069_{3}^{{}^{\circ}}$ – 39680_2_
4625.274	100	5	21614.287	13847_2_ – $35462_{3}^{{}^{\circ}}$
4624.939	5		21615.852	13088_3_ – $34704_{3}^{{}^{\circ}}$
4624.314	75	4	21618.774	3687_2_ – $25306_{2}^{{}^{\circ}}$
4623.553	8b	1	21622.332	8111_4_ – $29733_{0}^{{}^{\circ}}$
4623.169	10b	1	21624.128	11802_2_ – $33427_{2}^{{}^{\circ}}$
4623.118	50b	5	21624.367	$14206_{4}^{{}^{\circ}}$ – 35831_3_
4621.657	50	2	21631.203	$13945_{3}^{{}^{\circ}}$ – 35576_3_
4621.165	150	40	21633.506	8111_4_ – $29744_{3}^{{}^{\circ}}$
4621.076	50	2	21633.922	3687_2_ – $25321_{3}^{{}^{\circ}}$
4620.705	3b		21635.659	15970_3_ – $37605_{4}^{{}^{\circ}}$
4620.448	150	10	21636.862	13088_3_ – $34725_{3}^{{}^{\circ}}$
4620.241	200	15	21637.832	7280_2_ – $28917_{2}^{{}^{\circ}}$
4619.624	50	4	21640.722	$18053_{4}^{{}^{\circ}}$ – 39694_5_
4619.223	2		21642.600	$19588_{0}^{{}^{\circ}}$ – 41230_4_
4617.384	3		21651.220	$15166_{3}^{{}^{\circ}}$ – 36818_2_
4617.295	25	2	21651.637	$14481_{6}^{{}^{\circ}}$ – 36133_5_
4617.295	25	2	21651.637	17959_4_ – $39611_{3}^{{}^{\circ}}$
4616.451	75	100	21655.596	7502_3_ – $29157_{3}^{{}^{\circ}}$
4615.669	50	4	21659.265	$12114_{2}^{{}^{\circ}}$ – 33773_1_
4615.334	150	15	21660.837	3865_1_ – $25526_{1}^{{}^{\circ}}$
4615.025	200	40	21662.287	6362_2_ – $28024_{1}^{{}^{\circ}}$
4614.538	2		21664.573	18431_3_ – $40096_{2}^{{}^{\circ}}$
4614.278	8	2	21665.794	20054_2_ – $41720_{2}^{{}^{\circ}}$
4614.162	50	4	21666.339	15493_4_ – $37159_{3}^{{}^{\circ}}$
4613.605	150	15	21668.954	0_2_ – $21668_{1}^{{}^{\circ}}$
4610.628	300b	50	21682.945	$18011_{0}^{{}^{\circ}}$ – 39694_5_
4610.532	10	1	21683.397	$18809_{4}^{{}^{\circ}}$ – 40493_3_
4610.456	2		21683.754	4961_4_ – $26645_{0}^{{}^{\circ}}$
4610.307	5	1	21684.455	$15618_{3}^{{}^{\circ}}$ – 37303_4_
4610.078	100	4	21685.532	13847_2_ – $35533_{2}^{{}^{\circ}}$
4609.796	2		21686.859	15970_3_ – $37656_{3}^{{}^{\circ}}$
4609.516	2		21688.176	$15618_{3}^{{}^{\circ}}$ – 37307_3_
4609.439	2		21688.538	$13945_{3}^{{}^{\circ}}$ – 35633_4_
4608.620	150	8	21692.392	2869_3_ – $24561_{3}^{{}^{\circ}}$
4607.935	300	20	21695.617	7502_3_ – $29197_{2}^{{}^{\circ}}$
4607.834	50	5	21696.093	11601_1_ – $33297_{2}^{{}^{\circ}}$
4607.377	150	50	21698.245	$13175_{4}^{{}^{\circ}}$ – 34873_4_
4607.224	3		21698.965	$13175_{4}^{{}^{\circ}}$ – 34874_3_
4605.825	40	2	21705.556	18549_2_ – $40254_{3}^{{}^{\circ}}$
4605.425	2		21707.441	19273_2_ – $40980_{3}^{{}^{\circ}}$
4603.537	2		21716.344	13962_1_ – $35678_{1}^{{}^{\circ}}$
4603.434	1		21716.830	8800_4_ – $30517_{0}^{{}^{\circ}}$
4603.143	300b	100b	21718.202	$8243_{2}^{{}^{\circ}}$ – 29961_1_
4603.113	50b	8b	21718.344	$15166_{3}^{{}^{\circ}}$ – 36885_4_
4602.795	2		21719.845	$20566_{4}^{{}^{\circ}}$ – 42286_3_
4602.420	20	2	21721.614	$15490_{0}^{{}^{\circ}}$ – 37211_5_
4601.347	40	4	21726.680	$19986_{6}^{{}^{\circ}}$ – 41712_5_
4601.104	4	1	21727.827	$19503_{3}^{{}^{\circ}}$ – 41230_4_
4600.969	3		21728.464	$11877_{1}^{{}^{\circ}}$ – 33606_2_
4600.872	100	8	21728.922	$14481_{6}^{{}^{\circ}}$ – 36210_5_
4599.704	100	10	21734.440	5563_1_ – $27297_{1}^{{}^{\circ}}$
4598.939	40	2	21738.055	0_2_ – $21738_{2}^{{}^{\circ}}$
4598.888	4	1	21738.296	$19948_{4}^{{}^{\circ}}$ – 41686_3_
4598.434	50	4	21740.442	17073_1_ – $38814_{2}^{{}^{\circ}}$
4597.243	4		21746.075	$16783_{4}^{{}^{\circ}}$ – 38529_3_
4596.308	200	50	21750.498	7502_3_ – $29252_{2}^{{}^{\circ}}$
4596.176	100	8	21751.123	$17501_{0}^{{}^{\circ}}$ – 39252_5_
4595.902	3		21752.420	15863_2_ – $37616_{1}^{{}^{\circ}}$
4595.420	800r	200	21754.701	3687_2_ – $25442_{3}^{{}^{\circ}}$
4594.260	2		21760.194	$16346_{4}^{{}^{\circ}}$ – 38106_5_
4594.046	2		21761.208	$19817_{1}^{{}^{\circ}}$ – 41578_2_
4593.645	300	150	21763.107	13088_3_ – $34851_{2}^{{}^{\circ}}$
4593.524	4	2	21763.681	$17411_{3}^{{}^{\circ}}$ – 39174_3_
4593.362	8	1	21764.448	$19948_{4}^{{}^{\circ}}$ – 41712_5_
4592.958	100	5	21766.362	$11197_{0}^{{}^{\circ}}$ – 32963_5_
4592.877	3		21766.746	17073_1_ – $38840_{1}^{{}^{\circ}}$
4592.851	1		21766.869	15970_3_ – $37736_{2}^{{}^{\circ}}$
4592.668	500r	150	21767.737	$8243_{2}^{{}^{\circ}}$ – 30011_3_
4592.519	15	2	21768.443	$10783_{2}^{{}^{\circ}}$ – 32551_3_
4592.097	8		21770.444	8111_4_ – $29881_{4}^{{}^{\circ}}$
4591.972	1		21771.036	$18930_{3}^{{}^{\circ}}$ – 40701_4_
4591.581	8	1	21772.890	$18809_{4}^{{}^{\circ}}$ – 40582_5_
4589.526	3		21782.639	$17501_{0}^{{}^{\circ}}$ – 39283_4_
4589.328	5		21783.579	13297_4_ – $35081_{0}^{{}^{\circ}}$
4588.846	8	1	21785.867	$17224_{2}^{{}^{\circ}}$ – 39010_3_
4588.426	500r	200	21787.861	$10414_{4}^{{}^{\circ}}$ – 32202_5_
4588.350	100b	25	21788.222	11802_2_ – $33591_{3}^{{}^{\circ}}$
4587.332	3		21793.057	15863_2_ – $37656_{3}^{{}^{\circ}}$
4586.047	50b	2	21799.163	$14032_{2}^{{}^{\circ}}$ – 35831_3_
4585.175	2		21803.309	$14243_{1}^{{}^{\circ}}$ – 36047_2_
4584.300	5	2	21807.470	$19948_{4}^{{}^{\circ}}$ – 41755_4_
4584.247	10	2	21807.722	7502_3_ – $29310_{4}^{{}^{\circ}}$
4583.514	1		21811.210	$11877_{1}^{{}^{\circ}}$ – 33689_2_
4583.063	5		21813.356	$15490_{0}^{{}^{\circ}}$ – 37303_4_
4580.981	3		21823.270	18431_3_ – $40254_{3}^{{}^{\circ}}$
4580.405	15	2	21826.014	11601_1_ – $33427_{2}^{{}^{\circ}}$
4579.829	200	15	21828.759	4961_4_ – $26790_{4}^{{}^{\circ}}$
4579.367	75	3	21830.961	16554_6_ – $38385_{0}^{{}^{\circ}}$
4578.778	5b		21833.769	13297_4_ – $35131_{4}^{{}^{\circ}}$
4577.905	100	3	21837.933	3865_1_ – $25703_{2}^{{}^{\circ}}$
4577.823	100s	4	21838.324	3687_2_ – $25526_{1}^{{}^{\circ}}$
4577.582	150	5	21839.474	$16346_{4}^{{}^{\circ}}$ – 38186_4_
4576.964	4		21842.423	$15490_{0}^{{}^{\circ}}$ – 37332_6_
4576.742	100	2	21843.482	$11877_{1}^{{}^{\circ}}$ – 33721_2_
4576.542	20	2	21844.437	$19516_{2}^{{}^{\circ}}$ – 41361_3_
4576.272	8	2	21845.726	$14032_{2}^{{}^{\circ}}$ – 35877_2_
4575.821	1		21847.879	$19039_{2}^{{}^{\circ}}$ – 40886_2_
4575.247	200	50	21850.620	$17501_{0}^{{}^{\circ}}$ – 39351_5_
4574.028	200	20	21856.443	12847_3_ – $34704_{3}^{{}^{\circ}}$
4573.574	75	3	21858.613	14204_5_ – $36062_{4}^{{}^{\circ}}$
4573.433	3		21859.286	11802_2_ – $33662_{1}^{{}^{\circ}}$
4571.959	100	20	21866.334	9804_5_ – $31671_{4}^{{}^{\circ}}$
4570.972	500r	200	21871.055	$11197_{0}^{{}^{\circ}}$ – 33068_6_
4570.655	20h	3	21872.572	$17411_{3}^{{}^{\circ}}$ – 39283_4_
4570.551	2		21873.070	15863_2_ – $37736_{2}^{{}^{\circ}}$
4569.736	100	5	21876.971	7280_2_ – $29157_{1}^{{}^{\circ}}$
4569.636	20	2	21877.449	12847_3_ – $34725_{3}^{{}^{\circ}}$
4569.574	5	2	21877.746	7280_2_ – $29157_{3}^{{}^{\circ}}$
4569.148	150	20	21879.786	$10414_{4}^{{}^{\circ}}$ – 32293_5_
4568.523	100	10	21882.779	$14206_{4}^{{}^{\circ}}$ – 36089_4_
4568.143	200	40	21884.599	5563_1_ – $27447_{2}^{{}^{\circ}}$
4567.856	4	1	21885.974	$13945_{3}^{{}^{\circ}}$ – 35831_3_
4567.240	300	100	21888.926	$7795_{4}^{{}^{\circ}}$ – 29684_5_
4566.988	10		21890.134	0_2_ – $21890_{3}^{{}^{\circ}}$
4566.713	15	4	21891.452	$18809_{4}^{{}^{\circ}}$ – 40701_4_
4565.805	40b	5	21895.806	$11877_{1}^{{}^{\circ}}$ – 33773_1_
4565.783	75b	10	21895.911	17166_5_ – $39062_{0}^{{}^{\circ}}$
4564.835	150	10	21900.458	2869_3_ – $24769_{3}^{{}^{\circ}}$
4563.658	400r	200b	21906.106	8111_4_ – $30017_{3}^{{}^{\circ}}$
4562.727	8	1	21910.576	$17411_{3}^{{}^{\circ}}$ – 39321_3_
4562.476	75	5	21911.782	9804_5_ – $31716_{0}^{{}^{\circ}}$
4562.081	200	50	21913.679	17959_4_ – $39873_{0}^{{}^{\circ}}$
4561.704	5	2	21915.490	11802_2_ – $33718_{2}^{{}^{\circ}}$
4561.495	200	25	21916.494	4961_4_ – $26878_{3}^{{}^{\circ}}$
4561.397	100b	10	21916.965	7502_3_ – $29419_{2}^{{}^{\circ}}$
4561.348	500r	200	21917.200	7280_2_ – $29197_{1}^{{}^{\circ}}$
4561.228	150	10	21917.777	7280_2_ – $29197_{2}^{{}^{\circ}}$
4560.981	2		21918.964	$15166_{3}^{{}^{\circ}}$ – 37085_4_
4560.067	3		21923.357	13962_1_ – $35885_{2}^{{}^{\circ}}$
4559.396	2		21926.583	$14206_{4}^{{}^{\circ}}$ – 36133_5_
4559.312	50	5	21926.987	16351_0_ – $38278_{1}^{{}^{\circ}}$
4559.108	1		21927.969	$15166_{3}^{{}^{\circ}}$ – 37094_2_
4559.058	100	3	21928.209	$16217_{2}^{{}^{\circ}}$ – 38145_3_
4558.346	300	40	21931.634	$10526_{3}^{{}^{\circ}}$ – 32458_4_
4555.903	300	150	21943.394	13297_4_ – $35240_{3}^{{}^{\circ}}$
4555.809	500r	100	21943.847	3865_1_ – $25809_{1}^{{}^{\circ}}$
4555.143	100	5	21947.055	17959_4_ – $39906_{4}^{{}^{\circ}}$
4554.415	100	3	21950.563	$16783_{4}^{{}^{\circ}}$ – 38734_3_
4553.474	3		21955.099	$21902_{4}^{{}^{\circ}}$ – 43857_5_
4552.222	100b	8	21961.138	$7795_{4}^{{}^{\circ}}$ – 29756_4_
4552.154	300r	150	21961.466	8800_4_ – $30761_{3}^{{}^{\circ}}$
4551.597	25	5	21964.153	$15166_{3}^{{}^{\circ}}$ – 37131_3_
4551.473	200	75	21964.751	13847_2_ – $35812_{3}^{{}^{\circ}}$
4549.833	40	2	21972.669	7280_2_ – $29252_{2}^{{}^{\circ}}$
4549.606	5b		21973.765	14204_5_ – $36178_{4}^{{}^{\circ}}$
4549.052	2		21976.441	13297_4_ – $35273_{0}^{{}^{\circ}}$
4548.979	20	4	21976.794	$16783_{4}^{{}^{\circ}}$ – 38760_4_
4548.326	100b	10	21979.949	15970_3_ – $37950_{4}^{{}^{\circ}}$
4547.611	5	1	21983.404	$18069_{3}^{{}^{\circ}}$ – 40052_4_
4547.248	200	25	21985.160	6362_2_ – $28347_{2}^{{}^{\circ}}$
4546.675	75		21987.930	8800_4_ – $30788_{0}^{{}^{\circ}}$
4544.729	3b		21997.345	$18614_{1}^{{}^{\circ}}$ – 40611_2_
4544.657	20	4	21997.694	11802_2_ – $33800_{3}^{{}^{\circ}}$
4544.422	2		21998.831	$18053_{4}^{{}^{\circ}}$ – 40052_4_
4543.412	100b	8b	22003.721	12847_3_ – $34851_{2}^{{}^{\circ}}$
4543.384	20b	5	22003.857	$14206_{4}^{{}^{\circ}}$ – 36210_5_
4542.049	8	1	22010.324	6362_2_ – $28372_{1}^{{}^{\circ}}$
4541.501	10	2	22012.980	$10783_{2}^{{}^{\circ}}$ – 32796_3_
4541.039	8b		22015.219	$14032_{2}^{{}^{\circ}}$ – 36047_2_
4540.999	300r	75	22015.413	3687_2_ – $25703_{2}^{{}^{\circ}}$
4540.566	75	5	22017.513	$11197_{0}^{{}^{\circ}}$ – 33214_5_
4540.255	50b	15	22019.021	$17501_{0}^{{}^{\circ}}$ – 39520_4_
4540.070	4		22019.918	$16346_{4}^{{}^{\circ}}$ – 38366_4_
4537.143	100	3	22034.123	4961_4_ – $26995_{3}^{{}^{\circ}}$
4536.322	2		22038.111	13847_2_ – $35885_{2}^{{}^{\circ}}$
4535.979	100	8	22039.778	$7795_{4}^{{}^{\circ}}$ – 29835_3_
4535.546	100	8	22041.882	$13175_{4}^{{}^{\circ}}$ – 35216_5_
4535.388	10	3	22042.650	13088_3_ – $35131_{4}^{{}^{\circ}}$
4535.255	300r	150	22043.296	$8243_{2}^{{}^{\circ}}$ – 30286_1_
4535.103	2		22044.035	$104414_{4}^{{}^{\circ}}$ – 32458_4_
4534.402	50	4	22047.443	$15736_{1}^{{}^{\circ}}$ – 37784_2_
4533.717	4		22050.774	$14465_{2}^{{}^{\circ}}$ – 36515_3_
4533.071	300	75	22053.916	$7795_{4}^{{}^{\circ}}$ – 29849_4_
4531.574	3		22061.202	11601_1_ – $33662_{1}^{{}^{\circ}}$
4531.531	1		22061.411	$19516_{2}^{{}^{\circ}}$ – 41578_2_
4530.319	200	100	22067.313	$11241_{3}^{{}^{\circ}}$ – 33309_2_
4529.922	40	5	22069.247	$15618_{3}^{{}^{\circ}}$ – 37688_3_
4527.115	21		22082.930	15970_3_ – $38053_{3}^{{}^{\circ}}$
4525.447	40	3	22091.069	15863_2_ – $37954_{3}^{{}^{\circ}}$
4524.129	100	10	22097.505	$17224_{2}^{{}^{\circ}}$ – 39321_3_
4523.211	2		22101.990	$13945_{3}^{{}^{\circ}}$ – 36047_2_
4522.315	50	4	22106.369	15863_2_ – $37970_{2}^{{}^{\circ}}$
4521.332	15b	4	22111.175	5563_1_ – $27674_{2}^{{}^{\circ}}$
4521.305	1501	20	22111.307	16554_6_ – $38665_{0}^{{}^{\circ}}$
4521.196	500r	75	22111.840	2869_3_ – $24981_{3}^{{}^{\circ}}$
4521.059	50	5	22112.510	15493_4_ – $37605_{4}^{{}^{\circ}}$
4519.259	300	75	22121.317	3687_2_ – $25809_{1}^{{}^{\circ}}$
4518.609	5b		22124.499	$19588_{0}^{{}^{\circ}}$ – 41712_5_
4518.342	50		22125.807	$13175_{4}^{{}^{\circ}}$ – 35300_5_
4517.725	4b	2	22128.829	$19227_{6}^{{}^{\circ}}$ – 41356_5_
4517.680	4b		22129.049	$15618_{3}^{{}^{\circ}}$ – 37748_2_
4517.629	8b	3	22129.299	$14206_{4}^{{}^{\circ}}$ – 36336_3_
4516.736	3		22133.674	11601_1_ – $33734_{2}^{{}^{\circ}}$
4516.540	40	4	22134.634	14226_0_ – $36361_{1}^{{}^{\circ}}$
4515.629	10	2	22139.100	7280_2_ – $29419_{2}^{{}^{\circ}}$
4515.401	10	2	22140.218	$15166_{3}^{{}^{\circ}}$ – 37307_3_
4515.118	300r	50	22141.605	0_2_ – $22141_{3}^{{}^{\circ}}$
4514.548	10	2	22144.401	$13945_{3}^{{}^{\circ}}$ – 36089_4_
4514.398	3	1	22145.137	17166_5_ – $39311_{4}^{{}^{\circ}}$
4514.059	75	5	22146.800	$18053_{4}^{{}^{\circ}}$ – 40200_3_
4513.681	400r	150	22148.654	9804_5_ – $31953_{4}^{{}^{\circ}}$
4513.224	200	50	22150.897	6362_2_ – $28513_{2}^{{}^{\circ}}$
4512.950	2		22152.242	13088_3_ – $35240_{3}^{{}^{\circ}}$
4510.611	75		22163.729	15493_4_ – $37656_{3}^{{}^{\circ}}$
4510.611	75		22163.729	17398_3_ – $39562_{4}^{{}^{\circ}}$
4510.433	4		22164.604	13297_4_ – $35462_{3}^{{}^{\circ}}$
4509.960	100	8	22166.928	$11241_{3}^{{}^{\circ}}$ – 33408_3_
4509.844	20	4	22167.498	$19588_{0}^{{}^{\circ}}$ – 41755_4_
4509.461	2		22169.381	$16783_{4}^{{}^{\circ}}$ – 38953_5_
4509.390	2		22169.730	$19516_{2}^{{}^{\circ}}$ – 41686_3_
4508.715	2		22173.049	$16783_{4}^{{}^{\circ}}$ – 38956_4_
4508.035	100	20	22176.394	$11241_{3}^{{}^{\circ}}$ – 33418_4_
4507.639	2		22178.342	14204_5_ – $36382_{4}^{{}^{\circ}}$
4506.549	150	40	22183.706	$12114_{2}^{{}^{\circ}}$ – 34298_1_
4506.474	200	75	22184.075	7502_3_ – $29686_{3}^{{}^{\circ}}$
4505.607	8	3	22188.344	$14465_{2}^{{}^{\circ}}$ – 36653_2_
4505.448	2		22189.127	15863_2_ – $38053_{3}^{{}^{\circ}}$
4505.216	500r	100	22190.269	8800_4_ – $30990_{3}^{{}^{\circ}}$
4504.630	100	20	22193.156	$17501_{0}^{{}^{\circ}}$ – 39694_5_
4503.214	15	3	22200.135	$15618_{3}^{{}^{\circ}}$ – 37819_4_
4501.177	4	2	22210.181	$19817_{1}^{{}^{\circ}}$ – 42027_2_
4500.581	20	3	22213.122	17398_3_ – $39611_{3}^{{}^{\circ}}$
4499.984	500	200	22216.069	$7795_{4}^{{}^{\circ}}$ – 30011_3_
4499.705	50	5	22217.446	$11241_{3}^{{}^{\circ}}$ – 33459_4_
4499.431	15	3	22218.799	9804_5_ – $32023_{3}^{{}^{\circ}}$
4499.383	20	8	22219.037	$7795_{4}^{{}^{\circ}}$ – 30014_4_
4498.947	800r	200	22221.190	5563_1_ – $27784_{2}^{{}^{\circ}}$
4498.156	40	3	22225.097	15863_2_ – $38088_{2}^{{}^{\circ}}$
4497.908	150b	50	22226.323	$16783_{4}^{{}^{\circ}}$ – 39010_3_
4497.908	150b	50	22226.323	13847_2_ – $36074_{3}^{{}^{\circ}}$
4497.888	50b		22226.421	13962_1_ – $36188_{2}^{{}^{\circ}}$
4497.780	3	1	22226.955	6362_2_ – $28589_{3}^{{}^{\circ}}$
4497.125	4	2	22230.192	$13175_{4}^{{}^{\circ}}$ – 35405_3_
4495.598	15	4	22237.743	$15166_{3}^{{}^{\circ}}$ – 37404_3_
4494.863	2		22241.380	$14243_{1}^{{}^{\circ}}$ – 36485_2_
4494.692	100	8	22242.225	7502_3_ – $29744_{3}^{{}^{\circ}}$
4493.763	2		22246.824	15970_3_ – $38216_{3}^{{}^{\circ}}$
4493.568	100	20	22247.789	3865_1_ – $26113_{2}^{{}^{\circ}}$
4493.334	1000r	300	22248.948	0_2_ – $22248_{2}^{{}^{\circ}}$
4492.677	20	5	22252.201	$15490_{0}^{{}^{\circ}}$ – 37742_6_
4492.573	8	3	22252.716	$19503_{3}^{{}^{\circ}}$ – 41755_4_
4492.102	200	20	22255.050	$10526_{3}^{{}^{\circ}}$ – 32781_4_
4490.673	200	4	22262.131	$11197_{0}^{{}^{\circ}}$ – 33459_4_
4490.524	2		22262.870	13088_3_ – $35351_{4}^{{}^{\circ}}$
4489.664	200	100	22267.134	$14481_{6}^{{}^{\circ}}$ – 36749_6_
4489.245	2		22269.213	$17411_{3}^{{}^{\circ}}$ – 39680_2_
4488.554	5	2	22272.641	$18614_{1}^{{}^{\circ}}$ – 40886_2_
4488.312	200	100	22273.842	$15618_{3}^{{}^{\circ}}$ – 37892_4_
4486.898	300	100	22280.861	2558_0_ – $24838_{1}^{{}^{\circ}}$
4486.419	8	2	22283.240	12847_3_ – $35131_{4}^{{}^{\circ}}$
4485.898	5	2	22285.828	$11241_{3}^{{}^{\circ}}$ – 33527_4_
4485.713	150	40	22286.747	6362_2_ – $28649_{1}^{{}^{\circ}}$
4484.038	200		22295.072	17959_4_ – $40254_{3}^{{}^{\circ}}$
4483.346	300	1	22298.513	4961_4_ – $27260_{3}^{{}^{\circ}}$
4482.911	5	2	22300.677	$18930_{3}^{{}^{\circ}}$ – 41230_4_
4482.855	3	1	22300.955	$8243_{2}^{{}^{\circ}}$ – 30544_2_
4482.169	400r	100	22304.368	4961_4_ – $27266_{4}^{{}^{\circ}}$
4481.391	20	3	22308.241	11802_2_ – $34111_{1}^{{}^{\circ}}$
4481.226	10	8	22309.062	$14206_{4}^{{}^{\circ}}$ – 36515_3_
4481.010	8	3	22310.137	$14465_{2}^{{}^{\circ}}$ – 36775_3_
4480.547	251	10	22312.443	$16217_{2}^{{}^{\circ}}$ – 38529_3_
4480.308	1		22313.633	$20322_{0}^{{}^{\circ}}$ – 42635_5_
4480.267	25	3	22313.837	6362_2_ – $28676_{3}^{{}^{\circ}}$
4479.781	15	4	22316.258	$10783_{2}^{{}^{\circ}}$ – 33099_3_
4479.637	200	75	22316.975	$12114_{2}^{{}^{\circ}}$ – 34431_3_
4478.594	150	40	22322.172	2558_0_ – $24880_{1}^{{}^{\circ}}$
4478.402	150	40	22323.130	$11241_{3}^{{}^{\circ}}$ – 33564_3_
4477.865	3		22325.806	$17354_{1}^{{}^{\circ}}$ – 39680_2_
4477.216	50	8	22329.043	$15490_{0}^{{}^{\circ}}$ – 37819_4_
4477.037	8		22329.935	14226_0_ – $36556_{1}^{{}^{\circ}}$
4476.977	5	2	22330.235	$12114_{2}^{{}^{\circ}}$ – 34444_2_
4475.765	75	8	22336.281	15970_3_ – $38306_{4}^{{}^{\circ}}$
4475.410	2		22338.053	17073_1_ – $39411_{1}^{{}^{\circ}}$
4475.410	2		22338.053	$19948_{4}^{{}^{\circ}}$ – 42286_3_
4475.221	200	40	22338.996	0_2_ – $22338_{3}^{{}^{\circ}}$
4474.819	50	8	22341.003	8800_4_ – $31141_{0}^{{}^{\circ}}$
4474.789	10		22341.153	13847_2_ – $36188_{2}^{{}^{\circ}}$
4474.403	5		22343.080	$14465_{2}^{{}^{\circ}}$ – 36808_3_
4473.337	2		22348.405	3687_2_ – $26036_{3}^{{}^{\circ}}$
4472.844	200	50	22350.868	7502_3_ – $29853_{2}^{{}^{\circ}}$
4472.620	20	5	22351.987	$13945_{3}^{{}^{\circ}}$ – 36297_4_
4472.348	3		22353.347	$17847_{2}^{{}^{\circ}}$ – 40200_3_
4471.871	25	2	22355.731	4961_4_ – $27317_{3}^{{}^{\circ}}$
4471.483	3		22357.671	$18069_{3}^{{}^{\circ}}$ – 40426_4_
4470.990	200	25	22360.136	7280_2_ – $29640_{1}^{{}^{\circ}}$
4470.990	200	25	22360.136	$15736_{1}^{{}^{\circ}}$ – 38097_2_
4470.105	5	2	22364.563	$11241_{3}^{{}^{\circ}}$ – 33606_2_
4469.526	400r	150	22367.460	$10414_{4}^{{}^{\circ}}$ – 32781_4_
4468.320	200	50	22373.497	13088_3_ – $35462_{3}^{{}^{\circ}}$
4467.188	2		22379.166	7502_3_ – $29881_{4}^{{}^{\circ}}$
4465.975	150	2	22385.245	15970_3_ – $38355_{2}^{{}^{\circ}}$
4465.471	3		22387.771	$16346_{4}^{{}^{\circ}}$ – 38734_3_
4464.848	50	20	22390.895	$13945_{3}^{{}^{\circ}}$ – 36336_3_
4464.140	10	3	22394.446	8800_4_ – $31194_{4}^{{}^{\circ}}$
4463.834	200	100	22395.981	17166_5_ – $39562_{4}^{{}^{\circ}}$
4463.668	200	75	22396.814	0_2_ – $22396_{1}^{{}^{\circ}}$
4463.245	4	2	22398.937	13962_1_ – $36361_{1}^{{}^{\circ}}$
4462.757	3		22401.386	$13175_{4}^{{}^{\circ}}$ – 35576_3_
4461.815	15b		22406.115	8111_4_ – $30517_{0}^{{}^{\circ}}$
4461.789	200b	100	22406.246	7280_2_ – $29686_{3}^{{}^{\circ}}$
4461.602	15		22407.185	$13175_{4}^{{}^{\circ}}$ – 35582_5_
4461.241	200	100	22408.998	3687_2_ – $26096_{3}^{{}^{\circ}}$
4459.976	15	4	22415.354	$18011_{0}^{{}^{\circ}}$ – 40426_4_
4458.736	150	75	22421.588	3865_1_ – $26287_{1}^{{}^{\circ}}$
4458.002	600r	200	22425.279	3687_2_ – $26113_{2}^{{}^{\circ}}$
4457.782	2		22426.386	$17411_{3}^{{}^{\circ}}$ – 39837_3_
4456.809	2		22431.282	18549_2_ – $40980_{3}^{{}^{\circ}}$
4455.867	150	25	22436.024	13297_4_ – $35733_{4}^{{}^{\circ}}$
4455.148	2		22439.645	$18053_{4}^{{}^{\circ}}$ – 40493_3_
4454.139	75	10	22444.728	13088_3_ – $35533_{2}^{{}^{\circ}}$
4453.860	8	3	22446.134	15970_3_ – $38416_{3}^{{}^{\circ}}$
4452.565	200	100	22452.662	2869_3_ – $25321_{3}^{{}^{\circ}}$
4452.437	50	4	22453.308	$14032_{2}^{{}^{\circ}}$ – 36485_2_
4451.871	1		22456.162	$17224_{2}^{{}^{\circ}}$ – 39680_2_
4451.740	20	4	22456.823	15493_4_ – $37950_{4}^{{}^{\circ}}$
4451.359	40	15	22458.745	$13175_{4}^{{}^{\circ}}$ – 35633_4_
4450.802	100	40	22461.556	5563_1_ – $28024_{1}^{{}^{\circ}}$
4450.238	5		22464.402	7280_2_ – $29744_{3}^{{}^{\circ}}$
4450.066	2		22465.271	17959_4_ – $40425_{4}^{{}^{\circ}}$
4449.437	5	1	22468.446	$16783_{4}^{{}^{\circ}}$ – 39252_5_
4447.230	150	25	22479.597	$11241_{3}^{{}^{\circ}}$ – 33721_2_
4446.584	100	15	22482.862	8800_4_ – $31283_{3}^{{}^{\circ}}$
4445.901	200	150	22486.316	2869_3_ – $25355_{4}^{{}^{\circ}}$
4445.447	3	1	22488.612	16351_0_ – $38840_{1}^{{}^{\circ}}$
4445.309	200b	40b	22489.310	9804_5_ – $32294_{4}^{{}^{\circ}}$
4445.032	200	75	22490.712	$10783_{2}^{{}^{\circ}}$ – 33273_1_
4444.901	1		22491.375	15863_2_ – $38355_{2}^{{}^{\circ}}$
4443.665	150	100	22497.631	3865_1_ – $26363_{2}^{{}^{\circ}}$
4443.203	4	2	22499.970	$16783_{4}^{{}^{\circ}}$ – 39283_4_
4442.513	20	5	22503.465	12847_3_ – $35351_{4}^{{}^{\circ}}$
4441.659	50	4	22507.791	16554_6_ – $39062_{0}^{{}^{\circ}}$
4441.609	100	5	22508.045	0_2_ – $22508_{2}^{{}^{\circ}}$
4441.512	25	3	22508.536	17398_3_ – $39906_{4}^{{}^{\circ}}$
4441.198	40	5	22510.128	11601_1_ – $34111_{1}^{{}^{\circ}}$
4441.149	15	3	22510.376	$19516_{2}^{{}^{\circ}}$ – 42027_2_
4440.353	300	75	22514.411	$8243_{2}^{{}^{\circ}}$ – 30758_2_
4440.276	300	75	22514.802	7502_3_ – $30017_{3}^{{}^{\circ}}$
4440.230	2		22515.035	13297_4_ – $35812_{3}^{{}^{\circ}}$
4439.858	100	4	22516.921	$16217_{2}^{{}^{\circ}}$ – 38734_3_
4439.000	8	2	22521.273	$15166_{3}^{{}^{\circ}}$ – 37688_3_
4438.746	400	200	22522.562	6362_2_ – $28884_{3}^{{}^{\circ}}$
4438.092	150	8	22525.881	$10783_{2}^{{}^{\circ}}$ – 33309_2_
4437.446	300	20	22529.160	$18053_{4}^{{}^{\circ}}$ – 40582_5_
4435.705	4	1	22538.003	$16783_{4}^{{}^{\circ}}$ – 39321_3_
4435.296	100	10	22540.081	$13945_{3}^{{}^{\circ}}$ – 36485_2_
4434.075	150	40	22546.288	$18809_{4}^{{}^{\circ}}$ – 41356_5_
4433.490	150	50	22549.263	$10414_{4}^{{}^{\circ}}$ – 32963_5_
4432.252	400	150	22555.561	6362_2_ – $28917_{2}^{{}^{\circ}}$
4431.416	100	8	22559.816	15493_4_ – $38053_{3}^{{}^{\circ}}$
4429.979	100	8	22567.134	$15618_{3}^{{}^{\circ}}$ – 38186_4_
4429.822	200	20	22567.934	$16783_{4}^{{}^{\circ}}$ – 39351_5_
4429.722	8	2	22568.443	$14206_{4}^{{}^{\circ}}$ – 36775_3_
4429.633	200	20h	22568.897	11802_2_ – $34371_{2}^{{}^{\circ}}$
4428.828	5	2	22572.999	7280_2_ – $29853_{2}^{{}^{\circ}}$
4428.744	100	5	22573.427	2869_3_ – $25442_{3}^{{}^{\circ}}$
4428.553	200	40	22574.400	$14206_{4}^{{}^{\circ}}$ – 36781_5_
4427.530	100b	8	22579.616	13847_2_ – $36427_{3}^{{}^{\circ}}$
4427.232	100	15	22581.136	$15166_{3}^{{}^{\circ}}$ – 37748_2_
4426.019	1		22587.325	17398_3_ – $39985_{3}^{{}^{\circ}}$
4425.460	200	20	22590.177	$16217_{2}^{{}^{\circ}}$ – 38807_2_
4424.836	200b	50	22593.363	$12114_{2}^{{}^{\circ}}$ – 34707_3_
4424.661	4	2	22594.257	13962_1_ – $36556_{1}^{{}^{\circ}}$
4424.243	75	5	22596.392	14204_5_ – $36800_{4}^{{}^{\circ}}$
4423.719	400	150	22599.068	3687_2_ – $26287_{1}^{{}^{\circ}}$
4423.263	100	10	22601.398	$14206_{4}^{{}^{\circ}}$ – 36808_3_
4422.048	600	200	22607.608	$10783_{2}^{{}^{\circ}}$ – 33390_1_
4420.925	100	8	22613.350	$17224_{2}^{{}^{\circ}}$ – 39837_3_
4420.782	300b	100b	22614.082	12847_3_ – $35462_{3}^{{}^{\circ}}$
4420.256	200	20	22616.773	$15490_{0}^{{}^{\circ}}$ – 38106_5_
4420.112	100	10	22617.509	$15166_{3}^{{}^{\circ}}$ – 37784_2_
4419.768	100	20	22619.270	$13175_{4}^{{}^{\circ}}$ – 35794_4_
4418.554	75	3	22625.484	$10783_{2}^{{}^{\circ}}$ – 33408_3_
4418.201	15	2	22627.292	11802_2_ – $34430_{3}^{{}^{\circ}}$
4417.229	10	3	22632.271	$18069_{3}^{{}^{\circ}}$ – 40701_4_
4416.946	25	3	22633.721	14204_5_ – $36837_{0}^{{}^{\circ}}$
4416.844	500	100	22634.244	9804_5_ – $32439_{4}^{{}^{\circ}}$
4414.767	75	10	22644.892	13088_3_ – $35733_{4}^{{}^{\circ}}$
4414.486	500d	150	22646.334	$8243_{2}^{{}^{\circ}}$ – 30889_1_
4414.216	75	5	22647.719	$18053_{4}^{{}^{\circ}}$ – 40701_4_
4414.142	15	3	22648.098	$18930_{3}^{{}^{\circ}}$ – 41578_2_
4413.632	200	25	22650.715	8111_4_ – $30761_{3}^{{}^{\circ}}$
4413.589	1		22650.936	18549_2_ – $41200_{2}^{{}^{\circ}}$
4413.343	10b	3	22652.199	$15166_{3}^{{}^{\circ}}$ – 37819_4_
4412.565	300b	50b	22656.193	$13175_{4}^{{}^{\circ}}$ – 35831_3_
4412.270	25s	2	22657.707	7502_3_ – $30160_{4}^{{}^{\circ}}$
4411.139	50	5	22663.517	$16346_{4}^{{}^{\circ}}$ – 39010_3_
4409.899	100	5	22669.889	0_2_ – $22669_{3}^{{}^{\circ}}$
4408.883	800r	200	22675.113	3687_2_ – $26363_{2}^{{}^{\circ}}$
4408.481	300	150	22677.181	8111_4_ – $30788_{0}^{{}^{\circ}}$
4408.251	20	2	22678.364	$14206_{4}^{{}^{\circ}}$ – 36885_4_
4406.472	5	2	22687.520	$19948_{4}^{{}^{\circ}}$ – 42635_5_
4405.999	15	3	22689.955	$18011_{0}^{{}^{\circ}}$ – 40701_4_
4404.820	15	3	22696.028	$15490_{0}^{{}^{\circ}}$ – 38186_4_
4404.474	1		22697.811	17398_3_ – $40096_{2}^{{}^{\circ}}$
4404.113	300	40	22699.672	15970_3_ – $38669_{2}^{{}^{\circ}}$
4403.425	50	3	22703.218	18431_3_ – $41134_{4}^{{}^{\circ}}$
4402.927	500	150	22705.786	2869_3_ – $25575_{4}^{{}^{\circ}}$
4402.601	75	10	22707.467	17166_5_ – $39873_{0}^{{}^{\circ}}$
4402.448	50	5	22708.256	$13945_{3}^{{}^{\circ}}$ – 36653_2_
4402.246	200	50	22709.299	4961_4_ – $27670_{3}^{{}^{\circ}}$
4401.581	500	200	22712.729	$7795_{4}^{{}^{\circ}}$ – 30508_5_
4399.968	150	50	22721.055	$8243_{2}^{{}^{\circ}}$ – 30964_3_
4399.583	8	2	22723.044	11601_1_ – $34324_{2}^{{}^{\circ}}$
4399.453	15	3	22723.715	15493_4_ – $38216_{3}^{{}^{\circ}}$
4399.027	50	15	22725.916	$15166_{3}^{{}^{\circ}}$ – 37892_4_
4398.272	15	3	22729.817	$14481_{6}^{{}^{\circ}}$ – 37211_5_
4397.009	200w	40	22736.346	$16783_{4}^{{}^{\circ}}$ – 39520_4_
4396.140	100	10	22740.840	17166_5_ – $39906_{4}^{{}^{\circ}}$
4395.674	10	2	22743.251	$14032_{2}^{{}^{\circ}}$ – 36775_3_
4394.838	50	15	22747.577	$15618_{3}^{{}^{\circ}}$ – 38366_4_
4393.759	400	150	22753.163	7502_3_ – $30255_{3}^{{}^{\circ}}$
4393.131	4	2	22756.416	$18930_{3}^{{}^{\circ}}$ – 41686_3_
4392.974	400	100	22757.229	$7795_{4}^{{}^{\circ}}$ – 30552_4_
4392.495	100b	10	22759.710	$12114_{2}^{{}^{\circ}}$ – 34874_3_
4391.531	100	25	22764.706	17959_4_ – $40724_{0}^{{}^{\circ}}$
4391.390	150	50	22765.437	13297_4_ – $36062_{4}^{{}^{\circ}}$
4390.613	15	3	22769.466	$19516_{2}^{{}^{\circ}}$ – 42286_3_
4389.308	3	1	22776.236	$14032_{2}^{{}^{\circ}}$ – 36808_3_
4389.234	8	2	22776.620	13297_4_ – $36074_{3}^{{}^{\circ}}$
4388.569	10	3	22780.071	11802_2_ – $34583_{3}^{{}^{\circ}}$
4388.541	1		22780.216	$21077_{0}^{{}^{\circ}}$ – 43857_5_
4388.101	50	5	22782.500	$10526_{3}^{{}^{\circ}}$ – 33309_2_
4387.734	150	40	22784.406	5563_1_ – $28347_{2}^{{}^{\circ}}$
4387.028	100b	40	22788.072	11802_2_ – $34591_{1}^{{}^{\circ}}$
4386.138	3		22792.696	$16217_{2}^{{}^{\circ}}$ – 39010_3_
4385.753	25	5	22794.697	6362_2_ – $29157_{1}^{{}^{\circ}}$
4384.656	150b	50b	22800.400	$10414_{4}^{{}^{\circ}}$ – 33214_5_
4383.872	100	40	22804.478	14204_5_ – $37008_{0}^{{}^{\circ}}$
4383.602	15	3	22805.882	15863_2_ – $38669_{2}^{{}^{\circ}}$
4382.893	100	15	22809.571	5563_1_ – $28372_{1}^{{}^{\circ}}$
4382.526	3		22811.481	15863_2_ – $38675_{3}^{{}^{\circ}}$
4382.205	20	5	22813.152	15493_4_ – $38306_{4}^{{}^{\circ}}$
4381.130	2		22818.750	15970_3_ – $38788_{3}^{{}^{\circ}}$
4380.879	4	1	22820.057	3687_2_ – $26508_{3}^{{}^{\circ}}$
4380.716	15		22820.906	13847_2_ – $36668_{2}^{{}^{\circ}}$
4380.289	200	25	22823.131	$10783_{2}^{{}^{\circ}}$ – 33606_2_
4379.860	20	4	22825.366	$15166_{3}^{{}^{\circ}}$ – 37992_4_
4378.177	500r	100	22834.140	2869_3_ – $25703_{2}^{{}^{\circ}}$
4378.026	100	3	22834.928	6362_2_ – $29197_{1}^{{}^{\circ}}$
4377.650	8		22836.889	14204_5_ – $37041_{4}^{{}^{\circ}}$
4376.682	8	2	22841.940	$14465_{2}^{{}^{\circ}}$ – 37307_3_
4376.257	25	5	22844.158	15970_3_ – $38814_{2}^{{}^{\circ}}$
4374.710	8	3	22852.236	$8243_{2}^{{}^{\circ}}$ – 31095_1_
4374.124	600	150	22855.298	0_2_ – $22855_{3}^{{}^{\circ}}$
4374.013	150	40	22855.878	$7795_{4}^{{}^{\circ}}$ – 30651_3_
4373.206	40	8	22860.095	$21077_{0}^{{}^{\circ}}$ – 43937_6_
4372.067	1		22866.051	$15736_{1}^{{}^{\circ}}$ – 38602_2_
4371.140	75	8	22870.900	8800_4_ – $31671_{4}^{{}^{\circ}}$
4370.980	10	3	22871.737	17398_3_ – $40270_{4}^{{}^{\circ}}$
4370.068	5	2	22876.510	$15490_{0}^{{}^{\circ}}$ – 38366_4_
4369.876	200	50	22877.515	0_2_ – $22877_{1}^{{}^{\circ}}$
4369.602	50	25	22878.950	$14206_{4}^{{}^{\circ}}$ – 37085_4_
4369.496	100	25	22879.505	8111_4_ – $30990_{3}^{{}^{\circ}}$
4369.288	50b	25	22880.594	13297_4_ – $36178_{4}^{{}^{\circ}}$
4368.995	20	4	22882.128	$10526_{3}^{{}^{\circ}}$ – 33408_3_
4368.352	5	2	22885.497	12847_3_ – $35733_{4}^{{}^{\circ}}$
4367.418	100	15	22890.391	6362_2_ – $29252_{2}^{{}^{\circ}}$
4367.287	50	15	22891.077	4961_4_ – $27852_{0}^{{}^{\circ}}$
4367.191	5	2	22891.581	$10526_{3}^{{}^{\circ}}$ – 33418_4_
4367.113	3	1	22891.989	15493_4_ – $38385_{0}^{{}^{\circ}}$
4365.930	600	150	22898.192	$8243_{2}^{{}^{\circ}}$ – 31141_3_
4365.302	4	1	22901.486	11802_2_ – $34704_{3}^{{}^{\circ}}$
4365.020	25	8	22902.966	$18809_{4}^{{}^{\circ}}$ – 41712_5_
4364.506	8	3	22905.663	$16346_{4}^{{}^{\circ}}$ – 39252_5_
4364.461	5	2	22905.899	$10783_{2}^{{}^{\circ}}$ – 33689_2_
4364.040	25	4	22908.109	8111_4_ – $31019_{4}^{{}^{\circ}}$
4363.798	2		22909.379	13962_1_ – $36871_{2}^{{}^{\circ}}$
4363.589	1		22910.476	$16783_{4}^{{}^{\circ}}$ – 39694_5_
4363.500	8	2	22910.944	$15618_{3}^{{}^{\circ}}$ – 38529_3_
4362.720	15	3	22915.040	13847_2_ – $36762_{3}^{{}^{\circ}}$
4362.472	50	8	22916.343	8800_4_ – $31716_{0}^{{}^{\circ}}$
4361.201	4	1	22923.021	15493_4_ – $38416_{3}^{{}^{\circ}}$
4360.991	5	1	22924.125	$14206_{4}^{{}^{\circ}}$ – 37131_3_
4360.825	3	1	22924.997	15863_2_ – $38788_{3}^{{}^{\circ}}$
4360.167	100	15	22928.457	7280_2_ – $30208_{2}^{{}^{\circ}}$
4359.836	10	2	22930.198	$15166_{3}^{{}^{\circ}}$ – 38097_2_
4359.558	15	4	22931.660	$12114_{2}^{{}^{\circ}}$ – 35046_1_
4359.372	600r	100	22932.638	$10526_{3}^{{}^{\circ}}$ – 33459_4_
4358.321	500	150	22938.168	$10783_{2}^{{}^{\circ}}$ – 33721_2_
4358.082	15	5	22939.426	$14465_{2}^{{}^{\circ}}$ – 37404_3_
4357.974	8	2	22939.995	$13945_{3}^{{}^{\circ}}$ – 36885_4_
4356.835	3	1	22945.992	$18809_{4}^{{}^{\circ}}$ – 41755_4_
4356.045	75	20	22950.153	5563_1_ – $28513_{2}^{{}^{\circ}}$
4354.660	5	2	22957.452	$16217_{2}^{{}^{\circ}}$ – 39174_3_
4354.483	400	100	22958.386	$13175_{4}^{{}^{\circ}}$ – 36133_5_
4353.449	300b	100b	22963.838	3687_2_ – $26651_{2}^{{}^{\circ}}$
4352.613	150	25b	22968.249	2558_0_ – $25526_{1}^{{}^{\circ}}$
4351.466	100	50	22974.303	13088_3_ – $36062_{4}^{{}^{\circ}}$
4351.274	200	50	22975.317	7280_2_ – $30255_{3}^{{}^{\circ}}$
4351.120	8	3	22976.130	$17224_{2}^{{}^{\circ}}$ – 40200_3_
4351.017	50	10	22976.673	15863_2_ – $38840_{1}^{{}^{\circ}}$
4350.272	100	10	22980.609	8800_4_ – $31780_{3}^{{}^{\circ}}$
4349.630	40	5	22984.000	$15618_{3}^{{}^{\circ}}$ – 38602_2_
4349.346	20	4	22985.501	13088_3_ – $36074_{3}^{{}^{\circ}}$
4349.072	400	40	22986.949	4961_4_ – $27948_{4}^{{}^{\circ}}$
4348.828	3	1	22988.239	$19039_{2}^{{}^{\circ}}$ – 42027_2_
4348.599	400	75	22989.449	13847_2_ – $36837_{1}^{{}^{\circ}}$
4348.498	4	1	22989.983	11601_1_ – $34591_{1}^{{}^{\circ}}$
4348.403	3	1	22990.485	$10783_{2}^{{}^{\circ}}$ – 33773_1_
4347.638	100	15	22994.531	$10414_{4}^{{}^{\circ}}$ – 33408_3_
4346.432	500r	200	23000.911	7280_2_ – $30281_{1}^{{}^{\circ}}$
4346.410	15b		23001.028	$10526_{3}^{{}^{\circ}}$ – 33527_4_
4345.851	150	50	23003.986	$10414_{4}^{{}^{\circ}}$ – 33418_4_
4345.702	25	10	23004.775	$14206_{4}^{{}^{\circ}}$ – 37211_5_
4343.744	5		23015.144	7502_3_ – $30517_{4}^{{}^{\circ}}$
4343.381	100	25	23017.068	3865_1_ – $26882_{0}^{{}^{\circ}}$
4342.973	15	4	23019.230	$15166_{3}^{{}^{\circ}}$ – 38186_4_
4342.674	100	20	23020.815	17959_4_ – $40980_{3}^{{}^{\circ}}$
4342.445	500r	100	23022.029	$8243_{2}^{{}^{\circ}}$ – 31265_3_
4342.049	50	4	23024.128	13847_2_ – $36871_{2}^{{}^{\circ}}$
4340.896	200	40	23030.244	8111_4_ – $31141_{0}^{{}^{\circ}}$
4339.924	10	2	23035.402	$12114_{2}^{{}^{\circ}}$ – 35149_2_
4339.449	8	2	23037.923	12847_3_ – $35885_{2}^{{}^{\circ}}$
4339.374	15	3	23038.321	$10526_{3}^{{}^{\circ}}$ – 33564_3_
4339.069	3	1	23039.941	$17847_{2}^{{}^{\circ}}$ – 40886_2_
4338.108	400	100	23045.045	$10414_{4}^{{}^{\circ}}$ – 33459_4_
4337.639	15	4	23047.536	$19588_{0}^{{}^{\circ}}$ – 42635_5_
4337.277	800r	100	23049.460	0_2_ – $23049_{1}^{{}^{\circ}}$
4336.986	50	10	23051.006	7502_3_ – $30553_{2}^{{}^{\circ}}$
4335.992	5	2	23056.291	$14465_{2}^{{}^{\circ}}$ – 37521_3_
4335.731	200b	50b	23057.678	9804_5_ – $32862_{4}^{{}^{\circ}}$
4335.347	25b	8	23059.721	$11241_{3}^{{}^{\circ}}$ – 34301_4_
4333.552	10	3	23069.272	$20867_{7}^{{}^{\circ}}$ – 43937_6_
4333.284	75	4	23070.699	$15736_{1}^{{}^{\circ}}$ – 38807_2_
4332.339	150	40	23075.731	11802_2_ – $34878_{1}^{{}^{\circ}}$
4331.582	5	1	23079.764	$10526_{3}^{{}^{\circ}}$ – 33606_2_
4331.291	4b	2b	23081.314	$15618_{3}^{{}^{\circ}}$ – 38700_4_
4330.844	300	25	23083.697	8111_4_ – $31194_{4}^{{}^{\circ}}$
4330.565	8b	3	23085.184	13297_4_ – $36382_{4}^{{}^{\circ}}$
4330.412	50	20	23086.000	5563_1_ – $28649_{1}^{{}^{\circ}}$
4328.915	300r	25	23093.983	0_2_ – $23093_{2}^{{}^{\circ}}$
4328.436	3	1	23096.539	$14206_{4}^{{}^{\circ}}$ – 37303_4_
4327.981	15	3	23098.966	$14032_{2}^{{}^{\circ}}$ – 37131_3_
4327.715	150	25	23100.386	13088_3_ – $36188_{2}^{{}^{\circ}}$
4327.036	100	25	23104.011	17166_5_ – $40270_{4}^{{}^{\circ}}$
4325.860	150	8	23110.292	5563_1_ – $28673_{2}^{{}^{\circ}}$
4325.274	200	50	23113.423	$10414_{4}^{{}^{\circ}}$ – 33527_4_
4324.901	20	4	23115.416	$15618_{3}^{{}^{\circ}}$ – 38734_3_
4323.634	20	5	23122.190	$13175_{4}^{{}^{\circ}}$ – 36297_4_
4322.181	3		23129.963	13297_4_ – $36427_{3}^{{}^{\circ}}$
4321.224	5	2	23135.085	15863_2_ – $38998_{3}^{{}^{\circ}}$
4318.523	20	8	23149.555	$13945_{3}^{{}^{\circ}}$ – 37094_2_
4317.838	100	5	23153.227	8800_4_ – $31953_{4}^{{}^{\circ}}$
4316.371	25	5	23161.096	$13175_{4}^{{}^{\circ}}$ – 36336_3_
4316.218	10	3	23161.917	$18069_{3}^{{}^{\circ}}$ – 41230_4_
4316.109	200	50	23162.502	$10526_{3}^{{}^{\circ}}$ – 33689_2_
4315.254	500r	150	23167.091	2869_3_ – $26036_{3}^{{}^{\circ}}$
4315.052	100	15	23168.176	$11877_{1}^{{}^{\circ}}$ – 35046_1_
4314.491	8	3	23171.188	18549_2_ – $41720_{2}^{{}^{\circ}}$
4314.319	300	50	23172.112	8111_4_ – $31283_{3}^{{}^{\circ}}$
4314.279	2		23172.327	15493_4_ – $38665_{0}^{{}^{\circ}}$
4314.053	20	8	23173.541	$16346_{4}^{{}^{\circ}}$ – 39520_4_
4313.778	25	8	23175.018	17959_4_ – $41134_{4}^{{}^{\circ}}$
4313.335	2		23177.398	$18053_{4}^{{}^{\circ}}$ – 41230_4_
4312.992	800r	200	23179.241	2869_3_ – $26048_{4}^{{}^{\circ}}$
4312.992	800r	200	23179.241	4961_4_ – $28140_{4}^{{}^{\circ}}$
4311.798	300	100	23185.660	$8243_{2}^{{}^{\circ}}$ – 31429_1_
4311.625	150b	25	23186.590	13962_1_ – $37149_{2}^{{}^{\circ}}$
4311.576	100b	25	23186.854	11802_2_ – $34989_{1}^{{}^{\circ}}$
4311.062	150	15	23189.618	$11241_{3}^{{}^{\circ}}$ – 34431_3_
4310.957	75	5	23190.183	3687_2_ – $26878_{3}^{{}^{\circ}}$
4310.101	15	5	23194.788	$10526_{3}^{{}^{\circ}}$ – 33721_2_
4309.886	150	50	23195.945	3865_1_ – $27061_{2}^{{}^{\circ}}$
4309.557	25	4	23197.716	$14206_{4}^{{}^{\circ}}$ – 37404_3_
4308.599	100b	20	23202.874	$11241_{3}^{{}^{\circ}}$ – 34444_2_
4308.121	300	100	23205.449	$11241_{3}^{{}^{\circ}}$ – 34447_4_
4307.176	600r	200	23210.540	$11197_{0}^{{}^{\circ}}$ – 34407_6_
4305.495	5	2	23219.602	$18011_{0}^{{}^{\circ}}$ – 41230_4_
4304.955	150	10	23222.514	3865_1_ – $27087_{1}^{{}^{\circ}}$
4304.868	8	3	23222.984	$14465_{2}^{{}^{\circ}}$ – 37688_3_
4304.801	8	3	23223.345	8800_4_ – $32023_{0}^{{}^{\circ}}$
4304.290	8	3	23226.102	12847_3_ – $36074_{3}^{{}^{\circ}}$
4303.988	75	5	23227.732	2869_3_ – $26096_{3}^{{}^{\circ}}$
4300.370	3	1	23247.273	$19039_{2}^{{}^{\circ}}$ – 42286_3_
4299.839	500r	100	23250.144	$11197_{0}^{{}^{\circ}}$ – 34447_4_
4299.633	200	50	23251.258	2558_0_ – $25809_{1}^{{}^{\circ}}$
4298.121	75	5	23259.437	7502_3_ – $30761_{3}^{{}^{\circ}}$
4297.938	100	20	23260.428	$14481_{6}^{{}^{\circ}}$ – 37742_6_
4297.307	300b	75	23263.843	$11197_{0}^{{}^{\circ}}$ – 34460_5_
4295.902	2	1	23271.451	13962_1_ – $37234_{2}^{{}^{\circ}}$
4295.816	100	40	23271.917	$11877_{1}^{{}^{\circ}}$ – 35149_2_
4295.584	200	25	23273.174	7280_2_ – $30553_{2}^{{}^{\circ}}$
4295.236	25	5	23275.060	$14032_{2}^{{}^{\circ}}$ – 37307_3_
4294.756	75b	25b	23277.661	11601_1_ – $34878_{1}^{{}^{\circ}}$
4294.720	100b	25	23277.856	6362_2_ – $29640_{1}^{{}^{\circ}}$
4293.800	8b	3	23282.844	$14465_{2}^{{}^{\circ}}$ – 37748_2_
4292.681	4	1	23288.913	18431_3_ – $41720_{2}^{{}^{\circ}}$
4292.305	50	10	23290.953	$12114_{2}^{{}^{\circ}}$ – 35405_3_
4291.810	400	100	23293.639	$8243_{2}^{{}^{\circ}}$ – 31537_3_
4291.635	50	10	23294.589	15970_3_ – $39264_{3}^{{}^{\circ}}$
4290.394	200	50	23301.327	13847_2_ – $37149_{2}^{{}^{\circ}}$
4289.655	100	40	23305.341	$10526_{3}^{{}^{\circ}}$ – 33831_4_
4289.414	8	3	23306.650	$12114_{2}^{{}^{\circ}}$ – 35421_1_
4289.204	8	2	23307.791	3687_2_ – $26995_{3}^{{}^{\circ}}$
4289.204	8	2	23307.791	$18053_{4}^{{}^{\circ}}$ – 41361_3_
4288.669	200	75	23310.699	7502_3_ – $30812_{2}^{{}^{\circ}}$
4288.471	200b	50b	23311.775	13847_2_ – $37159_{3}^{{}^{\circ}}$
4287.082	200	40	23319.328	16554_6_ – $39873_{0}^{{}^{\circ}}$
4286.229	200b	75b	23323.969	6362_2_ – $29686_{3}^{{}^{\circ}}$
4285.757	3	1	23326.537	$12114_{2}^{{}^{\circ}}$ – 35440_3_
4285.398	10	3	23328.491	13297_4_ – $36625_{0}^{{}^{\circ}}$
4283.103	200b	50b	23340.991	12847_3_ – $36188_{2}^{{}^{\circ}}$
4283.073	50b	20b	23341.155	15970_3_ – $39311_{4}^{{}^{\circ}}$
4282.084	150b	50b	23346.546	$7795_{4}^{{}^{\circ}}$ – 31141_3_
4280.568	300r	100	23354.814	5563_1_ – $28917_{2}^{{}^{\circ}}$
4280.322	8	3	23356.156	$18930_{3}^{{}^{\circ}}$ – 42286_3_
4279.281	25	3	23361.837	$13945_{3}^{{}^{\circ}}$ – 37307_3_
4279.064	300	40	23363.022	$15166_{3}^{{}^{\circ}}$ – 38529_3_
4278.324	500r	200	23367.063	14204_5_ – $37571_{0}^{{}^{\circ}}$
4275.567	50	4	23382.130	6362_2_ – $29744_{3}^{{}^{\circ}}$
4275.165	15	2	23384.329	$10783_{2}^{{}^{\circ}}$ – 34167_3_
4274.817	200	25	23386.233	13847_2_ – $37234_{2}^{{}^{\circ}}$
4273.911	15	3	23391.190	$15618_{3}^{{}^{\circ}}$ – 39010_3_
4272.875	400r	50	23396.862	8800_4_ – $32197_{3}^{{}^{\circ}}$
4272.303	300r	100	23399.994	3687_2_ – $27087_{1}^{{}^{\circ}}$
4270.734	100	15	23408.591	$14481_{6}^{{}^{\circ}}$ – 37890_5_
4269.943	300	75	23412.927	8111_4_ – $31523_{3}^{{}^{\circ}}$
4269.272	3	1	23416.607	$16783_{4}^{{}^{\circ}}$ – 40200_3_
4269.063	100	5	23417.753	$10414_{4}^{{}^{\circ}}$ – 33831_4_
4266.447	15	3	23432.111	3865_1_ – $27297_{1}^{{}^{\circ}}$
4264.338	2001	20	23443.700	7280_2_ – $30723_{1}^{{}^{\circ}}$
4261.492	150	4	23459.356	$13945_{3}^{{}^{\circ}}$ – 37404_3_
4260.984	100	2	23462.153	$12114_{2}^{{}^{\circ}}$ – 35576_3_
4260.802	150	1	23463.155	$15490_{0}^{{}^{\circ}}$ – 38953_5_
4260.395	15	1	23465.397	13297_4_ – $36762_{3}^{{}^{\circ}}$
4260.332	800r	5	23465.744	9804_5_ – $33270_{4}^{{}^{\circ}}$
4260.288	75b	1	23465.986	$11241_{3}^{{}^{\circ}}$ – 34707_3_
4260.138	20		23466.812	$15490_{0}^{{}^{\circ}}$ – 38956_4_
4259.494	200	1	23470.360	$7795_{4}^{{}^{\circ}}$ – 31265_3_
4258.521	400	2	23475.723	$7795_{4}^{{}^{\circ}}$ – 31271_5_
4257.496	600r	4	23481.375	0_2_ – $23481_{1}^{{}^{\circ}}$
4257.459	10h		23481.579	7280_2_ – $30761_{3}^{{}^{\circ}}$
4256.254	500r	8	23488.226	7502_3_ – $30990_{3}^{{}^{\circ}}$
4256.039	10		23489.413	$14032_{2}^{{}^{\circ}}$ – 37521_3_
4255.795	150b	4	23490.760	6362_2_ – $29853_{2}^{{}^{\circ}}$
4255.759	50b	3	23490.958	$16346_{4}^{{}^{\circ}}$ – 39837_3_
4255.237	200	3	23493.840	2869_3_ – $26363_{2}^{{}^{\circ}}$
4254.380	100	1	23498.572	15970_3_ – $39468_{3}^{{}^{\circ}}$
4253.539	600r	20	23503.218	13297_4_ – $36800_{4}^{{}^{\circ}}$
4253.388	10b		23504.053	$14243_{1}^{{}^{\circ}}$ – 37748_2_
4251.421	5		23514.927	$10783_{2}^{{}^{\circ}}$ – 34298_1_
4251.286	50		23515.674	2869_3_ – $26384_{4}^{{}^{\circ}}$
4251.077	4		23516.830	7502_3_ – $31019_{4}^{{}^{\circ}}$
4250.775	100	2	23518.501	4961_4_ – $28480_{4}^{{}^{\circ}}$
4250.315	800r	75	23521.046	0_2_ – $23521_{3}^{{}^{\circ}}$
4249.476	150	1	23525.690	11802_2_ – $35328_{1}^{{}^{\circ}}$
4248.518	50	1	23530.995	$8243_{2}^{{}^{\circ}}$ – 31774_3_
4248.391	200	3	23531.698	$7795_{4}^{{}^{\circ}}$ – 31326_4_
4248.274	4		23532.346	$17354_{1}^{{}^{\circ}}$ – 40886_2_
4248.181	5		23532.861	7280_2_ – $30812_{2}^{{}^{\circ}}$
4247.861	150	2	23534.634	12847_3_ – $36382_{4}^{{}^{\circ}}$
4247.777	10		23535.099	$20322_{0}^{{}^{\circ}}$ – 43857_5_
4246.796	10b		23540.536	13297_4_ – $36837_{0}^{{}^{\circ}}$
4246.324	20	1	23543.153	$11877_{1}^{{}^{\circ}}$ – 35421_1_
4245.451	100	3	23547.994	15863_2_ – $39411_{1}^{{}^{\circ}}$
4244.022	25	1	23555.922	$15618_{3}^{{}^{\circ}}$ – 39174_3_
4243.261	100	2	23560.147	8111_4_ – $31671_{4}^{{}^{\circ}}$
4241.697	4		23568.834	15493_4_ – $39062_{0}^{{}^{\circ}}$
4241.096	100	2	23572.173	3687_2_ – $27260_{3}^{{}^{\circ}}$
4239.791	15		23579.429	12847_3_ – $36427_{3}^{{}^{\circ}}$
4239.276	4		23582.293	3865_1_ – $27447_{2}^{{}^{\circ}}$
4239.276	4		23582.293	17398_3_ – $40980_{3}^{{}^{\circ}}$
4237.535	10		23591.982	15970_3_ – $39562_{4}^{{}^{\circ}}$
4237.182	20		23593.947	5563_1_ – $29157_{1}^{{}^{\circ}}$
4236.049	50	1	23600.258	$13175_{4}^{{}^{\circ}}$ – 36775_3_
4235.463	600r	5	23603.523	0_2_ – $23603_{2}^{{}^{\circ}}$
4235.238	20	1	23604.777	15863_2_ – $39468_{3}^{{}^{\circ}}$
4235.092	50	1	23605.591	8111_4_ – $31716_{0}^{{}^{\circ}}$
4234.231	50	1	23610.390	17166_5_ – $40776_{4}^{{}^{\circ}}$
4233.907	50	1	23612.197	$14206_{4}^{{}^{\circ}}$ – 37819_4_
4232.478	1		23620.169	$16217_{2}^{{}^{\circ}}$ – 39837_3_
4231.617	10		23624.975	$14481_{6}^{{}^{\circ}}$ – 38106_5_
4231.136	4		23627.661	4961_4_ – $28589_{3}^{{}^{\circ}}$
4230.823	150	1	23629.409	3687_2_ – $27317_{3}^{{}^{\circ}}$
4230.427	500	5	23631.621	$11241_{3}^{{}^{\circ}}$ – 34873_4_
4230.299	15		23632.336	$11241_{3}^{{}^{\circ}}$ – 34874_3_
4229.961	10		23634.224	5563_1_ – $29197_{1}^{{}^{\circ}}$
4229.864	20		23634.766	5563_1_ – $29197_{2}^{{}^{\circ}}$
4229.148	300	4	23638.767	2869_3_ – $26508_{3}^{{}^{\circ}}$
4228.761	150b	3	23640.931	$10526_{3}^{{}^{\circ}}$ – 34167_3_
4228.677	25b	2	23641.400	15970_3_ – $39611_{3}^{{}^{\circ}}$
4228.418	100	3	23642.848	$16783_{4}^{{}^{\circ}}$ – 40426_4_
4227.387	400	8	23648.614	7280_2_ – $30928_{1}^{{}^{\circ}}$
4226.775	100	2	23652.038	$11197_{0}^{{}^{\circ}}$ – 34849_5_
4226.298	200		23654.708	6362_2_ – $30017_{3}^{{}^{\circ}}$
4226.047	10		23656.113	$14032_{2}^{{}^{\circ}}$ – 37688_3_
4225.489	501	2	23659.236	$18053_{4}^{{}^{\circ}}$ – 41712_5_
4225.095	3		23661.443	$10783_{2}^{{}^{\circ}}$ – 34444_2_
4224.871	3		23662.697	$17224_{2}^{{}^{\circ}}$ – 40886_2_
4224.488	3		23664.843	$15618_{3}^{{}^{\circ}}$ – 39283_4_
4223.589	150b	3	23669.880	8111_4_ – $31780_{3}^{{}^{\circ}}$
4222.439	20		23676.326	$11197_{0}^{{}^{\circ}}$ – 34873_4_
4221.691	75	2	23680.521	$14465_{2}^{{}^{\circ}}$ – 38145_3_
4221.151	100	2	23683.550	$14206_{4}^{{}^{\circ}}$ – 37890_5_
4220.730	150	2	23685.913	$8243_{2}^{{}^{\circ}}$ – 31929_3_
4220.730	150	2	23685.913	$14206_{4}^{{}^{\circ}}$ – 37892_4_
4220.570	3		23686.810	$18069_{3}^{{}^{\circ}}$ – 41755_4_
4220.064	200	15	23689.650	5563_1_ – $29252_{2}^{{}^{\circ}}$
4219.569	20	1	23692.430	7502_3_ – $31194_{4}^{{}^{\circ}}$
4217.185	100b	2b	23705.823	$16346_{4}^{{}^{\circ}}$ – 40052_4_
4216.543	3		23709.432	$16783_{4}^{{}^{\circ}}$ – 40493_3_
4216.371	2001	3	23710.399	7280_2_ – $30990_{3}^{{}^{\circ}}$
4216.205	75	1	23711.333	13297_4_ – $37008_{0}^{{}^{\circ}}$
4216.068	300s	5	23712.103	13088_3_ – $36800_{4}^{{}^{\circ}}$
4215.625	150	1	23714.595	4961_4_ – $28676_{3}^{{}^{\circ}}$
4215.383	3		23715.956	$14032_{2}^{{}^{\circ}}$ – 37748_2_
4215.210	4		23716.930	$12114_{2}^{{}^{\circ}}$ – 35831_3_
4214.828	200	4	23719.079	4961_4_ – $28680_{4}^{{}^{\circ}}$
4213.315	15	1	23727.596	11601_1_ – $35328_{1}^{{}^{\circ}}$
4213.067	400	5	23728.993	2558_0_ – $26287_{1}^{{}^{\circ}}$
4212.923	50	1	23729.804	$17501_{0}^{{}^{\circ}}$ – 41230_4_
4212.653	4		23731.325	$17847_{2}^{{}^{\circ}}$ – 41578_2_
4210.923	900r	10	23741.075	0_2_ – $23741_{1}^{{}^{\circ}}$
4210.765	400	10	23741.965	$7795_{4}^{{}^{\circ}}$ – 31537_3_
4210.600	2		23742.896	$13945_{3}^{{}^{\circ}}$ – 37688_3_
4210.455	75	2	23743.713	13297_4_ – $37041_{4}^{{}^{\circ}}$
4210.089	75	3	23745.778	14204_5_ – $37950_{4}^{{}^{\circ}}$
4208.921	100b	10b	23752.367	$14032_{2}^{{}^{\circ}}$ – 37784_2_
4208.867	200b		23752.672	0_2_ – $23752_{2}^{{}^{\circ}}$
4208.750	100b	5	23753.332	$10414_{4}^{{}^{\circ}}$ – 34167_3_
4208.412	150	4	23755.240	9804_5_ – $33560_{4}^{{}^{\circ}}$
4207.612	25	1	23759.756	3687_2_ – $27447_{2}^{{}^{\circ}}$
4207.176	20	1	23762.218	$15490_{0}^{{}^{\circ}}$ – 39252_5_
4206.058	10	1	23768.535	13847_2_ – $37616_{1}^{{}^{\circ}}$
4205.540	10		23771.462	15493_4_ – $39264_{3}^{{}^{\circ}}$
4205.012	4		23774.447	13962_1_ – $37736_{2}^{{}^{\circ}}$
4204.930	3		23774.911	$10526_{3}^{{}^{\circ}}$ – 34301_4_
4203.884	75	2	23780.826	7502_3_ – $31283_{3}^{{}^{\circ}}$
4203.575	15	1	23782.574	2869_3_ – $26651_{2}^{{}^{\circ}}$
4203.437	4	1	23783.355	13088_3_ – $36871_{2}^{{}^{\circ}}$
4203.085	15	1	23785.346	$14206_{4}^{{}^{\circ}}$ – 37992_4_
4202.266	50	2	23789.982	$15166_{3}^{{}^{\circ}}$ – 38956_4_
4200.686	2		23798.930	$16783_{4}^{{}^{\circ}}$ – 40582_5_
4200.149	2		23801.973	17398_3_ – $41200_{2}^{{}^{\circ}}$
4200.017	2		23802.721	$13945_{3}^{{}^{\circ}}$ – 37748_2_
4198.936	20	1	23808.849	3865_1_ – $27674_{2}^{{}^{\circ}}$
4198.876	15	1	23809.189	13847_2_ – $37656_{3}^{{}^{\circ}}$
4197.317	2		23818.032	15493_4_ – $39311_{4}^{{}^{\circ}}$
4197.016	2		23819.740	$17411_{3}^{{}^{\circ}}$ – 41230_4_
4196.846	10s		23820.705	12847_3_ – $36668_{2}^{{}^{\circ}}$
4194.936	200	5	23831.551	$7795_{4}^{{}^{\circ}}$ – 31626_5_
4193.601	1		23839.137	$13945_{3}^{{}^{\circ}}$ – 37784_2_
4193.518	15	2	23839.609	$17847_{2}^{{}^{\circ}}$ – 41686_3_
4193.017	600	15	23842.457	8111_4_ – $31953_{4}^{{}^{\circ}}$
4192.877	2		23843.253	$15166_{3}^{{}^{\circ}}$ – 39010_3_
4192.362	150	3	23846.182	6362_2_ – $30208_{2}^{{}^{\circ}}$
4191.146	15	1	23853.101	$14243_{1}^{{}^{\circ}}$ – 38097_2_
4191.028	3		23853.772	$16346_{4}^{{}^{\circ}}$ – 40200_3_
4190.813	5	1	23854.996	$17501_{0}^{{}^{\circ}}$ – 41356_5_
4190.620	15	1	23856.095	5563_1_ – $29419_{2}^{{}^{\circ}}$
4189.633	5	1	23861.715	$15490_{0}^{{}^{\circ}}$ – 39351_5_
4189.563	100	3	23862.113	13297_4_ – $37159_{3}^{{}^{\circ}}$
4187.512	1		23873.800	$13945_{3}^{{}^{\circ}}$ – 37819_4_
4187.142	50	3	23875.910	11802_2_ – $35678_{1}^{{}^{\circ}}$
4185.143	50	2	23887.314	$10414_{4}^{{}^{\circ}}$ – 34301_4_
4184.138	200	4	23893.052	6362_2_ – $30255_{3}^{{}^{\circ}}$
4182.933	20	2	23899.934	$14206_{4}^{{}^{\circ}}$ – 38106_5_
4182.707	4		23901.226	$15618_{3}^{{}^{\circ}}$ – 39520_4_
4182.080	50	2	23904.809	$10526_{3}^{{}^{\circ}}$ – 34431_3_
4181.518	2		23908.022	$11241_{3}^{{}^{\circ}}$ – 35149_2_
4180.720	10	1	23912.585	8111_4_ – $32023_{0}^{{}^{\circ}}$
4180.326	20	2	23914.839	12847_3_ – $36762_{3}^{{}^{\circ}}$
4179.861	1		23917.499	$16783_{4}^{{}^{\circ}}$ – 4071_4_
4179.760	10	2	23918.077	$10526_{3}^{{}^{\circ}}$ – 34444_2_
4179.620	200b	5	23918.878	3865_1_ – $27784_{2}^{{}^{\circ}}$
4179.314	100	8	23920.630	$10526_{3}^{{}^{\circ}}$ – 34447_4_
4179.220	150	3	23921.168	2869_3_ – $26790_{4}^{{}^{\circ}}$
4178.848	200	3	23923.297	4961_4_ – $28884_{3}^{{}^{\circ}}$
4178.624	2		23924.579	$10783_{2}^{{}^{\circ}}$ – 34707_3_
4177.281	4	1	23932.271	11601_1_ – $35533_{2}^{{}^{\circ}}$
4177.165	100	4	23932.936	$12114_{2}^{{}^{\circ}}$ – 36047_2_
4176.480	10	1	23936.861	15970_3_ – $39906_{4}^{{}^{\circ}}$
4175.322	4		23943.499	$15736_{1}^{{}^{\circ}}$ – 39680_2_
4174.620	2		23947.526	$13945_{3}^{{}^{\circ}}$ – 37892_4_
4173.720	25	1	23952.690	12847_3_ – $36800_{4}^{{}^{\circ}}$
4173.448	50	4	23954.251	8800_4_ – $32754_{3}^{{}^{\circ}}$
4170.533	500b	25	23970.993	4961_4_ – $28932_{4}^{{}^{\circ}}$
4169.762	4		23975.425	15493_4_ – $39468_{3}^{{}^{\circ}}$
4169.085	15	1	23979.319	$7795_{4}^{{}^{\circ}}$ – 31774_3_
4168.455	50	4	23982.943	$16217_{2}^{{}^{\circ}}$ – 40200_3_
4167.864	20	1	23986.343	3687_2_ – $27674_{2}^{{}^{\circ}}$
4165.766	800r	15	23998.423	$7795_{4}^{{}^{\circ}}$ – 31793_4_
4165.496	3		23999.979	$11877_{1}^{{}^{\circ}}$ – 35877_2_
4164.972	50	3	24002.998	7280_2_ – $31283_{3}^{{}^{\circ}}$
4163.947	100	2	24008.907	2869_3_ – $26878_{3}^{{}^{\circ}}$
4163.827	10	1	24009.599	11802_2_ – $35812_{3}^{{}^{\circ}}$
4162.782	75	5	24015.626	15970_3_ – $39985_{3}^{{}^{\circ}}$
4162.509	400	15	24017.201	$10414_{4}^{{}^{\circ}}$ – 34431_3_
4162.029	3		24019.971	$11197_{0}^{{}^{\circ}}$ – 35216_5_
4161.738	150	5	24021.650	7502_3_ – $31523_{3}^{{}^{\circ}}$
4161.344	2		24023.925	12847_3_ – $36871_{2}^{{}^{\circ}}$
4160.272	2b		24030.115	$15490_{0}^{{}^{\circ}}$ – 39520_4_
4159.764	20	1	24033.049	$10414_{4}^{{}^{\circ}}$ – 34447_4_
4159.152	75	2	24036.586	$13175_{4}^{{}^{\circ}}$ – 37211_5_
4158.535	800	20	24040.152	9804_5_ – $33844_{0}^{{}^{\circ}}$
4157.391	150b	3	24046.767	$10414_{4}^{{}^{\circ}}$ – 34460_5_
4157.363	25b	2b	24046.929	$13945_{3}^{{}^{\circ}}$ – 37992_4_
4156.481	50b	10b	24052.031	14226_0_ – $38278_{1}^{{}^{\circ}}$
4155.010	5		24060.546	13088_3_ – $37149_{2}^{{}^{\circ}}$
4154.719	200	5	24062.232	8800_4_ – $32862_{4}^{{}^{\circ}}$
4153.573	8	1	24068.870	15493_4_ – $39562_{4}^{{}^{\circ}}$
4153.204	5		24071.009	13088_3_ – $37159_{3}^{{}^{\circ}}$
4152.561	2		24074.736	18549_2_ – $42624_{1}^{{}^{\circ}}$
4152.147	15		24077.137	5563_1_ – $29640_{1}^{{}^{\circ}}$
4152.032	3		24077.803	11601_1_ – $35678_{1}^{{}^{\circ}}$
4151.144	50b	2	24082.954	11802_2_ – $35885_{2}^{{}^{\circ}}$
4150.598	5		24086.122	8111_4_ – $32197_{3}^{{}^{\circ}}$
4150.087	200b	5	24089.088	4961_4_ – $29050_{0}^{{}^{\circ}}$
4148.829	50	3	24096.392	3687_2_ – $27784_{2}^{{}^{\circ}}$
4148.724	100	3	24097.001	7502_3_ – $31599_{2}^{{}^{\circ}}$
4147.538	100	3	24103.892	$11197_{0}^{{}^{\circ}}$ – 35300_5_
4146.968	15	1	24107.205	13847_2_ – $37954_{3}^{{}^{\circ}}$
4145.858	5b		24113.659	$14032_{2}^{{}^{\circ}}$ – 38145_3_
4145.296	20	2	24116.928	$15166_{3}^{{}^{\circ}}$ – 39283_4_
4144.342	40	3	24122.480	13847_2_ – $37970_{2}^{{}^{\circ}}$
4143.711	5		24126.153	15970_3_ – $40096_{2}^{{}^{\circ}}$
4143.648	100	2	24126.520	2869_3_ – $26995_{3}^{{}^{\circ}}$
4143.337	5	1	24128.331	$13175_{4}^{{}^{\circ}}$ – 37303_4_
4142.325	100	3	24134.225	$7795_{4}^{{}^{\circ}}$ – 31929_3_
4141.719	100	3	24137.757	$14465_{2}^{{}^{\circ}}$ – 38602_2_
4138.040	200	3	24159.216	3865_1_ – $28024_{1}^{{}^{\circ}}$
4136.286	300	8	24169.461	$11877_{1}^{{}^{\circ}}$ – 36047_2_
4136.132	8	1	24170.361	16554_6_ – $40724_{0}^{{}^{\circ}}$
4135.480	300	3	24174.171	8111_4_ – $32285_{3}^{{}^{\circ}}$
4134.323	50	3	24180.936	14204_5_ – $38385_{0}^{{}^{\circ}}$
4134.062	200b	10b	24182.463	0_2_ – $24182_{2}^{{}^{\circ}}$
4133.958	200	10	24183.071	8111_4_ – $32294_{4}^{{}^{\circ}}$
4132.620	150	3	24190.901	6362_2_ – $30553_{2}^{{}^{\circ}}$
4131.710	150	3	24196.229	4961_4_ – $29157_{3}^{{}^{\circ}}$
4131.662	200b	3	24196.510	9804_5_ – $34001_{4}^{{}^{\circ}}$
4131.207	25	2	24199.175	$11241_{3}^{{}^{\circ}}$ – 35440_3_
4131.002	800	15	24200.375	$7795_{4}^{{}^{\circ}}$ – 31995_4_
4130.168	10	1	24205.262	13847_2_ – $38053_{3}^{{}^{\circ}}$
4129.073	3		24211.681	$17501_{0}^{{}^{\circ}}$ – 41712_5_
4128.050	100	2	24217.681	$7795_{4}^{{}^{\circ}}$ – 32012_4_
4127.345	150b	10	24221.818	$12114_{2}^{{}^{\circ}}$ – 36336_3_
4126.032	10	1	24229.526	$13175_{4}^{{}^{\circ}}$ – 37404_3_
4124.105	5b		24240.847	$13945_{3}^{{}^{\circ}}$ – 38186_4_
4124.043	150	5	24241.211	13847_2_ – $38088_{2}^{{}^{\circ}}$
4123.726	100	3	24243.074	8800_4_ – $33043_{3}^{{}^{\circ}}$
4123.601	150	3	24243.809	7280_2_ – $31523_{3}^{{}^{\circ}}$
4121.744	3		24254.732	$17501_{0}^{{}^{\circ}}$ – 41755_4_
4120.364	15	1	24262.855	$10783_{2}^{{}^{\circ}}$ – 35046_1_
4118.490	200	4	24273.895	13297_4_ – $37571_{0}^{{}^{\circ}}$
4118.225	5	1	24275.457	$17411_{3}^{{}^{\circ}}$ – 41686_3_
4117.698	4b	1	24278.564	7502_3_ – $31780_{3}^{{}^{\circ}}$
4116.635	25b	4b	24284.833	15970_3_ – $40254_{3}^{{}^{\circ}}$
4116.635	25b	4b	24284.833	11601_1_ – $35885_{2}^{{}^{\circ}}$
4115.759	500r	50	24290.002	5563_1_ – $29853_{2}^{{}^{\circ}}$
4113.873	20	1	24301.137	12847_3_ – $37149_{2}^{{}^{\circ}}$
4112.753	600b	15	24307.755	0_2_ – $24307_{2}^{{}^{\circ}}$
4112.713	300b		24307.991	$8243_{2}^{{}^{\circ}}$ – 32551_3_
4112.665	40b	2	24308.275	13297_4_ – $37605_{4}^{{}^{\circ}}$
4112.104	10	1	24311.591	12847_3_ – $37159_{3}^{{}^{\circ}}$
4110.829	200b	10b	24319.131	7280_2_ – $31599_{2}^{{}^{\circ}}$
4110.317	2		24322.160	17398_3_ – $41720_{2}^{{}^{\circ}}$
4110.174	4		24323.007	$14206_{4}^{{}^{\circ}}$ – 38529_3_
4109.323	400r	5	24328.044	8111_4_ — $32439_{4}^{{}^{\circ}}$
4108.183	4		24334.794	$11241_{3}^{{}^{\circ}}$ – 35576_3_
4107.861	100	3	24336.702	3687_2_ – $28024_{1}^{{}^{\circ}}$
4106.522	4		24344.637	$17411_{3}^{{}^{\circ}}$ – 41755_4_
4106.154	15b	2b	24346.819	$10526_{3}^{{}^{\circ}}$ – 34873_4_
4106.034	10	1	24347.530	$10526_{3}^{{}^{\circ}}$ – 34874_3_
4105.894	25b		24348.361	4961_4_ – $29310_{4}^{{}^{\circ}}$
4105.062	25	2	24353.295	$15166_{3}^{{}^{\circ}}$ – 39520_4_
4103.692	20b	2	24361.425	6362_2_ – $30723_{1}^{{}^{\circ}}$
4102.820	20		24366.603	$10783_{2}^{{}^{\circ}}$ – 35149_2_
4102.617	400r	3	24367.809	7502_3_ – $31870_{2}^{{}^{\circ}}$
4101.234	5		24376.026	14204_5_ – $38580_{4}^{{}^{\circ}}$
4100.919	40	2	24377.898	9804_5_ – $34182_{0}^{{}^{\circ}}$
4100.341	1000r	20	24381.334	0_2_ – $24381_{2}^{{}^{\circ}}$
4099.553	100	4	24386.021	11802_2_ – $36188_{2}^{{}^{\circ}}$
4099.553	100	4	24386.021	12847_3_ – $37234_{2}^{{}^{\circ}}$
4098.730	100s	2	24390.917	2869_3_ – $27260_{3}^{{}^{\circ}}$
4097.747	500b	8	24396.768	2869_3_ – $27266_{4}^{{}^{\circ}}$
4097.321	200	8	24399.305	6362_2_ – $30761_{3}^{{}^{\circ}}$
4096.077	150	4	24406.714	$7795_{4}^{{}^{\circ}}$ – 32202_5_
4094.901	50	3	24413.724	15493_4_ – $39906_{4}^{{}^{\circ}}$
4093.672	40	2	24421.053	0_2_ – $24421_{3}^{{}^{\circ}}$
4091.988	8	1	24431.103	13847_2_ – $38278_{1}^{{}^{\circ}}$
4091.737	40	2	24432.602	7280_2_ – $31712_{1}^{{}^{\circ}}$
4091.590	10	1	24433.479	$15618_{3}^{{}^{\circ}}$ – 40052_4_
4089.138	400	8	24448.130	2869_3_ – $27317_{3}^{{}^{\circ}}$
4088.728	500	8	24450.582	6362_2_ – $30812_{2}^{{}^{\circ}}$
4088.629	100	3	24451.174	7502_3_ – $31953_{4}^{{}^{\circ}}$
4087.285	200	5	24459.214	$10414_{4}^{{}^{\circ}}$ – 34873_4_
4087.166	501	2	24459.926	$10414_{4}^{{}^{\circ}}$ – 34874_3_
4086.936	15	1	24461.303	14204_5_ – $38665_{0}^{{}^{\circ}}$
4085.434	500	10	24470.296	8800_4_ – $33270_{4}^{{}^{\circ}}$
4085.257	200	8	24471.356	$14481_{6}^{{}^{\circ}}$ – 38953_5_
4083.469	500	8	24482.071	3865_1_ – $28347_{2}^{{}^{\circ}}$
4081.592	50	2	24493.329	$14206_{4}^{{}^{\circ}}$ – 38700_4_
4081.368	800	8	24494.673	8800_4_ – $33294_{3}^{{}^{\circ}}$
4080.707	400	20	24498.641	$7795_{4}^{{}^{\circ}}$ – 32293_5_
4080.359	150	4	24500.730	7280_2_ – $31780_{3}^{{}^{\circ}}$
4079.273	25b	1	24507.253	3865_1_ – $28372_{1}^{{}^{\circ}}$
4078.302	100	2	24513.087	$13175_{4}^{{}^{\circ}}$ – 37688_3_
4077.623	15	1	24517.169	13088_3_ – $37605_{4}^{{}^{\circ}}$
4075.907	150	5	24527.491	$14206_{4}^{{}^{\circ}}$ – 38734_3_
4075.503	400r	10	24529.922	2558_0_ – $27087_{1}^{{}^{\circ}}$
4073.960	25	1	24539.213	$12114_{2}^{{}^{\circ}}$ – 36653_2_
4073.856	200	3	24539.839	9804_5_ – $34344_{4}^{{}^{\circ}}$
4073.007	20	1	24544.954	$14465_{2}^{{}^{\circ}}$ – 39010_3_
4071.750	200b	15	24552.532	$8243_{2}^{{}^{\circ}}$ – 32796_3_
4071.731	100b		24552.646	$11241_{3}^{{}^{\circ}}$ – 35794_4_
4071.553	100	1	24553.719	$14206_{4}^{{}^{\circ}}$ – 38760_4_
4070.756	100	2	24558.527	11802_2_ – $36361_{1}^{{}^{\circ}}$
4070.238	150	3	24561.652	0_2_ – $24561_{3}^{{}^{\circ}}$
4070.115	25	1	24562.394	$15490_{0}^{{}^{\circ}}$ – 40052_4_
4070.041	75	1	24562.841	$10526_{3}^{{}^{\circ}}$ – 35089_3_
4069.901	5		24563.686	$14243_{1}^{{}^{\circ}}$ – 38807_2_
4069.461	500r	15	24566.342	6362_2_ – $30928_{1}^{{}^{\circ}}$
4069.112	50	2	24568.449	13847_2_ – $38416_{3}^{{}^{\circ}}$
4068.705	75		24570.906	$14032_{2}^{{}^{\circ}}$ – 38602_2_
4068.470	50	2	24572.325	$16783_{4}^{{}^{\circ}}$ – 41356_5_
4067.451	400r	5	24578.481	2869_3_ – $27447_{2}^{{}^{\circ}}$
4066.961	50	1	24581.442	$15618_{3}^{{}^{\circ}}$ – 40200_3_
4066.822	100	2	24582.283	$18053_{4}^{{}^{\circ}}$ – 42635_5_
4066.435	25		24584.622	$13945_{3}^{{}^{\circ}}$ – 38529_3_
4065.889	100	3	24587.923	11601_1_ – $36188_{2}^{{}^{\circ}}$
4065.618	100	4	24589.562	$11241_{3}^{{}^{\circ}}$ – 35831_3_
4065.551	50	1	24589.968	7280_2_ – $31870_{2}^{{}^{\circ}}$
4064.332	400	10	24597.343	$11197_{0}^{{}^{\circ}}$ – 35794_4_
4063.407	1500r	75	24602.942	$11197_{0}^{{}^{\circ}}$ – 35799_6_
4061.625	300	3	24613.736	14226_0_ – $38840_{1}^{{}^{\circ}}$
4060.241	5		24622.126	$10783_{2}^{{}^{\circ}}$ – 35405_3_
4060.062	200	1	24623.211	$10526_{3}^{{}^{\circ}}$ – 35149_2_
4059.847	200	10	24624.515	11802_2_ – $36427_{3}^{{}^{\circ}}$
4059.253	1500r	20	24628.119	6362_2_ – $30990_{3}^{{}^{\circ}}$
4058.936	75	8	24630.042	$19227_{6}^{{}^{\circ}}$ – 43857_5_
4057.940	200	2	24636.087	$11241_{3}^{{}^{\circ}}$ – 35877_2_
4056.718	10		24643.508	8111_4_ – $32754_{3}^{{}^{\circ}}$
4056.636	200	2	24644.006	$13175_{4}^{{}^{\circ}}$ – 37819_4_
4056.399	100	2	24645.446	5563_1_ – $30208_{2}^{{}^{\circ}}$
4056.007	150	1	24647.828	3865_1_ – $28513_{2}^{{}^{\circ}}$
4055.908	200	1	24648.430	13088_3_ – $37736_{2}^{{}^{\circ}}$
4054.387	200	8	24657.676	$13945_{3}^{{}^{\circ}}$ – 38602_2_
4054.302	300	8	24658.193	7502_3_ – $32160_{2}^{{}^{\circ}}$
4054.080	20		24659.543	3687_2_ – $28347_{2}^{{}^{\circ}}$
4053.841	40		24660.997	$12114_{2}^{{}^{\circ}}$ – 36775_3_
4053.527	1000r	75	24662.907	$7795_{4}^{{}^{\circ}}$ – 32458_4_
4052.240	100b		24670.740	$15166_{3}^{{}^{\circ}}$ – 39837_3_
4051.913	40		24672.731	$17354_{1}^{{}^{\circ}}$ – 42027_2_
4051.500	500	15	24675.246	$10414_{4}^{{}^{\circ}}$ – 35089_3_
4049.944	500	40	24684.726	3687_2_ – $28372_{1}^{{}^{\circ}}$
4049.535	200	25	24687.219	9804_5_ – $34492_{4}^{{}^{\circ}}$
4048.432	800	50	24693.945	$12114_{2}^{{}^{\circ}}$ – 36808_3_
4048.288	800	40	24694.824	7502_3_ – $32197_{3}^{{}^{\circ}}$
4047.058	50	1	24702.329	$14032_{2}^{{}^{\circ}}$ – 38734_3_
4046.824	200	15	24703.757	$12114_{2}^{{}^{\circ}}$ – 36818_2_
4046.252	200	8	24707.249	13962_1_ – $38669_{2}^{{}^{\circ}}$
4045.848	40b	2	24709.717	$14465_{2}^{{}^{\circ}}$ – 39174_3_
4045.818	50b	3	24709.900	$19227_{6}^{{}^{\circ}}$ – 43937_6_
4045.226	200	2	24713.516	6362_2_ – $31075_{2}^{{}^{\circ}}$
4044.926	300	8	24715.349	$13175_{4}^{{}^{\circ}}$ – 37890_5_
4044.515	200b	2	24717.860	5563_1_ – $30281_{1}^{{}^{\circ}}$
4043.394	1000r	50	24724.713	4961_4_ – $29686_{3}^{{}^{\circ}}$
4040.971	100		24739.538	2558_0_ – $27297_{1}^{{}^{\circ}}$
4039.865	800	20	24746.311	$14206_{4}^{{}^{\circ}}$ – 38953_5_
4039.268	100	2	24749.968	4961_4_ – $29711_{0}^{{}^{\circ}}$
4039.268	100	2	24749.968	$14206_{4}^{{}^{\circ}}$ – 38956_4_
4039.022	150	1	24751.475	8111_4_ – $32862_{4}^{{}^{\circ}}$
4038.635	100		24753.847	11802_2_ – $36556_{1}^{{}^{\circ}}$
4038.456	300	2	24754.944	$13945_{3}^{{}^{\circ}}$ – 38700_4_
4038.228	300	10	24756.342	$7795_{4}^{{}^{\circ}}$ – 32551_3_
4037.993	15		24757.783	12847_3_ – $37605_{4}^{{}^{\circ}}$
4037.663	100	2	24759.806	8800_4_ – $33560_{4}^{{}^{\circ}}$
4037.562	500	15	24760.425	11601_1_ – $36361_{1}^{{}^{\circ}}$
4036.047	2000r	50	24769.719	0_2_ – $24769_{3}^{{}^{\circ}}$
4035.931	200	8	24770.431	$14481_{6}^{{}^{\circ}}$ – 39252_5_
4035.730	300	1	24771.665	4961_4_ – $29733_{0}^{{}^{\circ}}$
4035.089	100		24775.600	$14032_{2}^{{}^{\circ}}$ – 38807_2_
4034.878	200	8	24776.896	15493_4_ – $40270_{4}^{{}^{\circ}}$
4033.907	600r	8	24782.860	4961_4_ – $29744_{3}^{{}^{\circ}}$
4033.776	500	8	24783.664	3865_1_ – $28649_{1}^{{}^{\circ}}$
4032.596	600r	200	24790.916	8800_4_ – $33591_{3}^{{}^{\circ}}$
4032.199	20		24793.357	$10783_{2}^{{}^{\circ}}$ – 35576_3_
4030.842	2000r	50	24801.704	2869_3_ – $27670_{3}^{{}^{\circ}}$
4030.590	20		24803.254	$14206_{4}^{{}^{\circ}}$ – 39010_3_
4030.292	300	40	24805.088	2869_3_ – $27674_{2}^{{}^{\circ}}$
4030.214	100	1	24805.568	$11241_{3}^{{}^{\circ}}$ – 36047_2_
4030.078	25		24806.405	15970_3_ – $40776_{4}^{{}^{\circ}}$
4029.827	400r	15	24807.951	3865_1_ – $28673_{2}^{{}^{\circ}}$
4029.658	400	15	24808.991	12847_3_ – $37656_{3}^{{}^{\circ}}$
4028.334	200	10	24817.145	$13175_{4}^{{}^{\circ}}$ – 37992_4_
4027.546	1501	20	24822.000	13847_2_ – $38669_{2}^{{}^{\circ}}$
4027.007	500r	10	24825.322	3687_2_ – $28513_{2}^{{}^{\circ}}$
4026.644	4		24827.560	13847_2_ – $38675_{3}^{{}^{\circ}}$
4024.801	300r	8	24838.929	0_2_ – $24838_{1}^{{}^{\circ}}$
4023.338	400	10	24847.961	$11241_{3}^{{}^{\circ}}$ – 36089_4_
4022.728	75	1	24851.729	13962_1_ – $38814_{2}^{{}^{\circ}}$
4022.067	500b		24855.813	$8243_{2}^{{}^{\circ}}$ – 33099_3_
4021.940	200b	4	24856.598	$14465_{2}^{{}^{\circ}}$ – 39321_3_
4021.750	200	8	24857.772	14204_5_ – $39062_{0}^{{}^{\circ}}$
4021.149	300	10	24861.487	13088_3_ – $37950_{4}^{{}^{\circ}}$
4021.007	75	2	24862.365	$13945_{3}^{{}^{\circ}}$ – 38807_2_
4020.462	100		24865.735	11802_2_ – $36668_{2}^{{}^{\circ}}$
4020.354	500	20	24866.403	13088_3_ – $37954_{3}^{{}^{\circ}}$
4019.787	200	5	24869.911	$14481_{6}^{{}^{\circ}}$ – 39351_5_
4018.932	25	2	24875.201	$17411_{3}^{{}^{\circ}}$ – 42286_3_
4018.354	200	15	24878.779	$10526_{3}^{{}^{\circ}}$ – 35405_3_
4018.118	50b		24880.241	0_2_ – $24880_{1}^{{}^{\circ}}$
4018.099	400b	15	24880.358	7280_2_ – $32160_{2}^{{}^{\circ}}$
4017.461	8		24884.309	$16346_{4}^{{}^{\circ}}$ – 41230_4_
4017.257	100	2	24885.573	$15166_{3}^{{}^{\circ}}$ – 40052_4_
4017.062	400	5	24886.781	$10414_{4}^{{}^{\circ}}$ – 35300_5_
4016.896	10		24887.809	11601_1_ – $36488_{2}^{{}^{\circ}}$
4016.112	100	2	24892.668	$11197_{0}^{{}^{\circ}}$ – 36089_4_
4014.716	400	10	24901.323	3687_2_ – $28589_{3}^{{}^{\circ}}$
4014.472	40	2	24902.837	$16783_{4}^{{}^{\circ}}$ – 41686_3_
4012.610	25	2	24914.392	$10526_{3}^{{}^{\circ}}$ – 35440_3_
4012.495	2000r	150	24915.106	2869_3_ – $27784_{2}^{{}^{\circ}}$
4012.190	150	2	24917.000	7280_2_ – $32197_{3}^{{}^{\circ}}$
4011.739	800r	5b	24919.801	4961_4_ – $29881_{4}^{{}^{\circ}}$
4011.591	500r	40	24920.721	6362_2_ – $31283_{3}^{{}^{\circ}}$
4009.816	400b	10	24931.752	$13175_{4}^{{}^{\circ}}$ – 38106_5_
4009.783	200b	2	24931.957	15493_4_ – $40425_{4}^{{}^{\circ}}$
4009.724	400	10	24932.324	8111_4_ – $33043_{3}^{{}^{\circ}}$
4009.057	1500r	100	24936.472	$11197_{0}^{{}^{\circ}}$ – 36133_5_
4009.012	300r	50	24936.752	7502_3_ – $32439_{4}^{{}^{\circ}}$
4008.443	10		24940.292	$11877_{1}^{{}^{\circ}}$ – 36818_2_
4008.312	100		24941.107	13847_2_ – $38788_{3}^{{}^{\circ}}$
4008.210	1500	100	24941.741	$7795_{4}^{{}^{\circ}}$ – 32737_5_
4005.960	300	8	24955.750	11601_1_ – $36556_{1}^{{}^{\circ}}$
4005.297	75	1	24959.881	11802_2_ – $36762_{3}^{{}^{\circ}}$
4005.091	500r	10	24961.165	3687_2_ – $28649_{1}^{{}^{\circ}}$
4004.560	20		24964.474	13088_3_ – $38053_{3}^{{}^{\circ}}$
4004.238	8	1	24966.482	13847_2_ – $38814_{2}^{{}^{\circ}}$
4003.993	4		24968.009	$14206_{4}^{{}^{\circ}}$ – 39174_3_
4003.574	400	25	24970.622	$13175_{4}^{{}^{\circ}}$ – 38145_3_
4002.376	75	3	24978.096	$14032_{2}^{{}^{\circ}}$ – 39010_3_
4001.992	50		24980.493	$12114_{2}^{{}^{\circ}}$ – 37094_2_
4001.894	300	2	24981.105	0_2_ – $24981_{3}^{{}^{\circ}}$
4001.198	100h		24985.450	3687_2_ – $28673_{2}^{{}^{\circ}}$
4001.058	800r	40	24986.324	$7795_{4}^{{}^{\circ}}$ – 32781_4_
4000.748	200		24988.261	3687_2_ – $28676_{3}^{{}^{\circ}}$
4000.445	200	3	24990.153	5563_1_ – $30553_{2}^{{}^{\circ}}$
4000.280	800	20	24991.184	$10414_{4}^{{}^{\circ}}$ – 35405_3_
4000.041	200b		24992.677	$15618_{3}^{{}^{\circ}}$ – 40611_2_
4000.024	100b		24992.783	13847_2_ – $38840_{1}^{{}^{\circ}}$
3998.953	600	20	24999.477	8800_4_ – $33799_{4}^{{}^{\circ}}$
3998.800	100b	5	25000.433	13088_3_ – $38088_{2}^{{}^{\circ}}$
3998.733	400b	5	25000.852	$7795_{4}^{{}^{\circ}}$ – 32796_3_
3998.657	300s	2	25001.327	7502_3_ – $32503_{2}^{{}^{\circ}}$
3998.061	300	5	25005.054	7280_2_ – $32285_{3}^{{}^{\circ}}$
3997.443	15b		25008.920	13297_4_ – $38306_{4}^{{}^{\circ}}$
3997.347	200	4	25009.521	$16346_{4}^{{}^{\circ}}$ – 41356_5_
3997.172	50	1	25010.615	15970_3_ – $40980_{3}^{{}^{\circ}}$
3997.109	300	5	25011.009	$13175_{4}^{{}^{\circ}}$ – 38186_4_
3997.019	300	8	25011.573	$13945_{3}^{{}^{\circ}}$ – 38956_4_
3996.670	500	15	25013.757	$11197_{0}^{{}^{\circ}}$ – 36210_5_
3996.508	100	2	25014.771	$16346_{4}^{{}^{\circ}}$ – 41361_3_
3996.203	300	25	25016.680	$12114_{2}^{{}^{\circ}}$ – 37131_3_
3994.038	5		25030.240	$8243_{2}^{{}^{\circ}}$ – 33273_1_
3993.515	200	15	25033.518	$15166_{3}^{{}^{\circ}}$ – 40200_3_
3993.397	100		25034.258	11802_2_ – $36837_{1}^{{}^{\circ}}$
3991.731	800r	40	25044.706	8800_4_ – $33844_{0}^{{}^{\circ}}$
3991.624	300b	40	25045.377	$14206_{4}^{{}^{\circ}}$ – 39252_5_
3991.289	15		25047.479	$18809_{4}^{{}^{\circ}}$ – 43857_5_
3990.892	300	15	25049.971	$10526_{3}^{{}^{\circ}}$ – 35576_3_
3990.809	300	25	25050.492	5563_1_ – $30613_{0}^{{}^{\circ}}$
3990.492	800r	10	25052.482	3865_1_ – $28917_{2}^{{}^{\circ}}$
3990.020	150b	40	25055.445	4961_4_ – $30017_{3}^{{}^{\circ}}$
3990.002	20b	3	25055.558	$11241_{3}^{{}^{\circ}}$ – 36297_4_
3988.953	25		25062.147	$17224_{2}^{{}^{\circ}}$ – 42286_3_
3988.519	25	4	25064.874	$13945_{3}^{{}^{\circ}}$ – 39010_3_
3988.429	75		25065.440	$8243_{2}^{{}^{\circ}}$ – 33309_2_
3988.078	10b	8b	25067.646	11601_1_ – $36668_{2}^{{}^{\circ}}$
3987.867	150	2	25068.972	11802_2_ – $36871_{2}^{{}^{\circ}}$
3987.206	200b		25073.128	7502_3_ – $32575_{2}^{{}^{\circ}}$
3986.604	200	25	25076.914	$14206_{4}^{{}^{\circ}}$ – 39283_4_
3986.215	25		25079.361	2869_3_ – $27948_{4}^{{}^{\circ}}$
3985.815	8		25081.878	9804_5_ – $34886_{0}^{{}^{\circ}}$
3985.737	4		25082.369	$15618_{3}^{{}^{\circ}}$ – 40701_4_
3984.879	300	40	25087.769	13297_4_ – $38385_{0}^{{}^{\circ}}$
3984.095	200		25092.706	$15490_{0}^{{}^{\circ}}$ – 40582_5_
3983.815	2		25094.470	$11241_{3}^{{}^{\circ}}$ – 36336_3_
3983.783	10		25094.671	$10783_{2}^{{}^{\circ}}$ – 35877_2_
3982.894	200	8	25100.272	$11197_{0}^{{}^{\circ}}$ – 36297_4_
3982.607	100	2	25102.081	12847_3_ – $37950_{4}^{{}^{\circ}}$
3981.827	300	100	25106.998	12847_3_ – $37954_{3}^{{}^{\circ}}$
3980.575	15		25114.895	$14206_{4}^{{}^{\circ}}$ – 39321_3_
3979.956	200	8	25118.801	13297_4_ – $38416_{3}^{{}^{\circ}}$
3979.402	75	2	25122.298	12847_3_ – $37970_{2}^{{}^{\circ}}$
3978.438	50	1	25128.385	13088_3_ – $38216_{3}^{{}^{\circ}}$
3977.434	15		25134.728	$17501_{0}^{{}^{\circ}}$ – 42635_5_
3976.152	50b	1	25142.831	$14032_{2}^{{}^{\circ}}$ – 39174_3_
3975.976	20		25143.944	$16217_{2}^{{}^{\circ}}$ – 41361_3_
3975.831	150	5	25144.862	$14206_{4}^{{}^{\circ}}$ – 39351_5_
3975.468	200	20	25147.157	$8243_{2}^{{}^{\circ}}$ – 33390_1_
3975.008	5s		25150.067	$15736_{1}^{{}^{\circ}}$ – 40886_2_
3974.829	200	4	25151.200	13847_2_ – $38998_{3}^{{}^{\circ}}$
3973.507	25	2	25159.568	8111_4_ – $33270_{4}^{{}^{\circ}}$
3973.333	500	10	25160.669	5563_1_ – $30723_{1}^{{}^{\circ}}$
3973.196	1000r	50	25161.537	6362_2_ – $31523_{3}^{{}^{\circ}}$
3973.063	40b	1	25162.379	$10414_{4}^{{}^{\circ}}$ – 35576_3_
3972.640	500	75	25165.058	$8243_{2}^{{}^{\circ}}$ – 33408_3_
3972.230	75	2	25167.656	8800_4_ – $33967_{3}^{{}^{\circ}}$
3972.153	1500r	300	25168.144	$7795_{4}^{{}^{\circ}}$ – 32963_5_
3972.153	1500r	300	25168.144	$10414_{4}^{{}^{\circ}}$ – 35582_5_
3969.665	300	15	25183.918	8111_4_ – $33294_{3}^{{}^{\circ}}$
3967.610	200	2	25196.961	3687_2_ – $28884_{3}^{{}^{\circ}}$
3967.392	2000r	300	25198.346	4961_4_ – $30160_{4}^{{}^{\circ}}$
3966.334	200	4	25205.067	12847_3_ – $38053_{3}^{{}^{\circ}}$
3965.483	100	3	25210.476	$19227_{6}^{{}^{\circ}}$ – 44437_6_
3964.455	75	2	25217.013	$11877_{1}^{{}^{\circ}}$ – 37094_2_
3964.329	200	3	25217.814	13088_3_ – $38306_{4}^{{}^{\circ}}$
3964.030	300	15	25219.717	$10414_{4}^{{}^{\circ}}$ – 35633_4_
3962.475	100	3	25229.613	$13945_{3}^{{}^{\circ}}$ – 39174_3_
3962.420	400r	15	25229.964	3687_2_ – $28917_{2}^{{}^{\circ}}$
3962.371	75	5b	25230.276	15970_3_ – $41200_{2}^{{}^{\circ}}$
3962.197	200	8	25231.384	15493_4_ – $40724_{0}^{{}^{\circ}}$
3960.685	10		25241.015	12847_3_ – $38088_{2}^{{}^{\circ}}$
3960.270	500	50	25243.661	$11241_{3}^{{}^{\circ}}$ – 36485_2_
3959.300	1000r	100	25249.845	5563_1_ – $30812_{2}^{{}^{\circ}}$
3958.928	200	3	25252.217	7502_3_ – $32754_{3}^{{}^{\circ}}$
3957.736	40		25259.823	$15166_{3}^{{}^{\circ}}$ – 40426_4_
3957.059	100	20	25264.144	$10783_{2}^{{}^{\circ}}$ – 36047_2_
3956.481	150	20	25267.835	$10526_{3}^{{}^{\circ}}$ – 35794_4_
3956.005	400	10	25270.875	11601_1_ – $36871_{2}^{{}^{\circ}}$
3955.891	400	50b	25271.604	2869_3_ – $28140_{4}^{{}^{\circ}}$
3955.477	15		25274.249	$11241_{3}^{{}^{\circ}}$ – 36515_3_
3955.170	800	40	25276.210	9804_5_ – $35081_{0}^{{}^{\circ}}$
3954.130	100	20	25282.858	13297_4_ – $38580_{4}^{{}^{\circ}}$
3954.068	100	10	25283.255	15493_4_ – $40776_{4}^{{}^{\circ}}$
3953.056	4		25289.727	$14032_{2}^{{}^{\circ}}$ – 39321_3_
3952.967	75	1	25290.296	$12114_{2}^{{}^{\circ}}$ – 37404_3_
3952.761	500r	10	25291.614	3865_1_ – $29157_{1}^{{}^{\circ}}$
3952.421	25		25293.790	4961_4_ – $30255_{3}^{{}^{\circ}}$
3950.804	300	8	25304.142	$7795_{4}^{{}^{\circ}}$ – 33099_3_
3950.707	100s	3	25304.763	$10526_{3}^{{}^{\circ}}$ – 35831_3_
3950.393	1000r	200	25306.775	0_2_ – $25306_{2}^{{}^{\circ}}$
3948.133	500	40	25321.261	$8243_{2}^{{}^{\circ}}$ – 33564_3_
3948.029	600r	15	25321.928	0_2_ – $25321_{3}^{{}^{\circ}}$
3947.329	1000r	100	25326.418	9804_5_ – $35131_{4}^{{}^{\circ}}$
3947.136	500	50	25327.656	13088_3_ – $38416_{3}^{{}^{\circ}}$
3946.481	400	10	25331.860	3865_1_ – $29197_{1}^{{}^{\circ}}$
3946.391	300	25	25332.438	3865_1_ – $29197_{2}^{{}^{\circ}}$
3945.206	200	8	25340.046	$16346_{4}^{{}^{\circ}}$ – 41686_3_
3944.253	500	20	25346.169	11802_2_ – $37149_{2}^{{}^{\circ}}$
3943.605	300	25	25350.333	6362_2_ – $31712_{1}^{{}^{\circ}}$
3943.459	300	40	25351.272	$10526_{3}^{{}^{\circ}}$ – 35877_2_
3942.907	300	20	25354.821	$13175_{4}^{{}^{\circ}}$ – 38529_3_
3942.628	100		25356.615	11802_2_ – $37159_{3}^{{}^{\circ}}$
3942.072	500b	50	25360.192	7502_3_ – $32862_{4}^{{}^{\circ}}$
3941.962	10	1	25360.899	$16217_{2}^{{}^{\circ}}$ – 41578_2_
3941.682	10		25362.701	$8243_{2}^{{}^{\circ}}$ – 33606_2_
3941.232	100	2	25365.597	5563_1_ – $30928_{1}^{{}^{\circ}}$
3941.137	100	3	25366.208	$16346_{4}^{{}^{\circ}}$ – 41712_5_
3940.837	200	5	25368.139	13297_4_ – $38665_{0}^{{}^{\circ}}$
3940.705	200	4	25368.989	12847_3_ – $38216_{3}^{{}^{\circ}}$
3940.422	5		25370.811	7280_2_ – $32650_{1}^{{}^{\circ}}$
3939.538	75	2	25376.503	$13945_{3}^{{}^{\circ}}$ – 39321_3_
3939.318	150	8	25377.921	13297_4_ – $38675_{3}^{{}^{\circ}}$
3938.956	75	2	25380.253	$10414_{4}^{{}^{\circ}}$ – 35794_4_
3938.614	400b	150	25382.457	8800_4_ – $34182_{0}^{{}^{\circ}}$
3938.152	200	8	25385.434	7280_2_ – $32665_{1}^{{}^{\circ}}$
3937.861	100	2b	25387.310	3865_1_ – $29252_{2}^{{}^{\circ}}$
3934.465	15	1	25409.223	$16346_{4}^{{}^{\circ}}$ – 41755_4_
3934.060	150	3	25411.838	$11241_{3}^{{}^{\circ}}$ – 36653_2_
3933.238	400	40	25417.149	$10414_{4}^{{}^{\circ}}$ – 35831_3_
3933.034	200	5	25418.467	6362_2_ – $31780_{3}^{{}^{\circ}}$
3932.911	1500r	200	25419.262	$7795_{4}^{{}^{\circ}}$ – 33214_5_
3931.085	150	3	25431.069	11802_2_ – $37234_{2}^{{}^{\circ}}$
3930.252	150	4	25436.459	$14243_{1}^{{}^{\circ}}$ – 39680_2_
3929.291	300	8	25442.680	0_2_ – $25442_{3}^{{}^{\circ}}$
3928.967	200b	40b	25444.778	$15166_{3}^{{}^{\circ}}$ – 40611_2_
3928.865	300	50	25445.439	$8243_{2}^{{}^{\circ}}$ – 33689_2_
3928.308	100	10	25449.046	8111_4_ – $33560_{4}^{{}^{\circ}}$
3927.806	400	10	25452.299	7502_3_ – $32954_{2}^{{}^{\circ}}$
3926.864	200	8	25458.404	12847_3_ – $38306_{4}^{{}^{\circ}}$
3925.596	300	8	25466.628	2558_0_ – $28024_{1}^{{}^{\circ}}$
3925.219	400	50	25469.074	9804_5_ – $35273_{0}^{{}^{\circ}}$
3925.093	1000r	200b	25469.891	3687_2_ – $29157_{3}^{{}^{\circ}}$
3924.404	5001	10	25474.363	7280_2_ – $32754_{3}^{{}^{\circ}}$
3923.799	500r	20	25478.290	2869_3_ – $28347_{2}^{{}^{\circ}}$
3923.510	100	2	25480.167	8111_4_ – $33591_{3}^{{}^{\circ}}$
3922.394	100	8	25487.416	$14206_{4}^{{}^{\circ}}$ – 39694_5_
3921.782	25	4	25491.394	13297_4_ – $38788_{3}^{{}^{\circ}}$
3921.731	15		25491.725	13088_3_ – $38580_{4}^{{}^{\circ}}$
3920.058	300	10	25502.604	$16783_{4}^{{}^{\circ}}$ – 42286_3_
3919.275	200	5	25507.699	6362_2_ – $31870_{2}^{{}^{\circ}}$
3919.023	1000r	100	25509.339	3687_2_ – $29197_{1}^{{}^{\circ}}$
3918.934	300	8	25509.919	3687_2_ – $29197_{2}^{{}^{\circ}}$
3917.270	500	50	25520.755	$10526_{3}^{{}^{\circ}}$ – 36047_2_
3916.597	200	8	25525.140	$13175_{4}^{{}^{\circ}}$ – 38700_4_
3916.417	500r	25	25526.313	0_2_ – $25526_{1}^{{}^{\circ}}$
3915.848	200	15	25530.022	$8243_{2}^{{}^{\circ}}$ – 33773_1_
3915.295	300	8	25533.628	$11241_{3}^{{}^{\circ}}$ – 36775_3_
3915.172	5		25534.430	$15166_{3}^{{}^{\circ}}$ – 40701_4_
3914.163	200s	8	25541.012	7502_3_ – $33043_{3}^{{}^{\circ}}$
3913.645	2001	5	25544.393	8800_4_ – $34344_{4}^{{}^{\circ}}$
3913.302	75	2	25546.632	9804_5_ – $35351_{4}^{{}^{\circ}}$
3913.082	300	25	25548.068	11601_1_ – $37149_{2}^{{}^{\circ}}$
3912.738	75	3	25550.314	17073_1_ – $42624_{1}^{{}^{\circ}}$
3912.485	751	1	25551.966	$11197_{0}^{{}^{\circ}}$ – 36749_6_
3912.211	75	2	25553.756	3865_1_ – $29419_{2}^{{}^{\circ}}$
3911.950	300b	25	25555.460	4961_4_ – $30517_{0}^{{}^{\circ}}$
3911.910	500r	40	25555.722	4961_4_ – $30517_{4}^{{}^{\circ}}$
3911.523	200	3	25558.250	11601_1_ – $37159_{1}^{{}^{\circ}}$
3911.364	15		25559.289	$13175_{4}^{{}^{\circ}}$ – 38734_3_
3910.773	300	8	25563.152	$10526_{3}^{{}^{\circ}}$ – 36089_4_
3910.622	50	2	25564.139	13847_2_ – $39411_{1}^{{}^{\circ}}$
3910.521	100	2	25564.799	3687_2_ – $29252_{2}^{{}^{\circ}}$
3910.251	50	2	25566.564	$11241_{3}^{{}^{\circ}}$ – 36808_3_
3909.991	100	8	25568.264	12847_3_ – $38416_{3}^{{}^{\circ}}$
3909.139	300	20	25573.837	$12114_{2}^{{}^{\circ}}$ – 37688_3_
3908.979	300	25	25574.883	$13945_{3}^{{}^{\circ}}$ – 39520_4_
3908.749	500	15	25576.388	$11241_{3}^{{}^{\circ}}$ – 36818_2_
3908.014	75	1	25581.198	13088_3_ – $38669_{2}^{{}^{\circ}}$
3907.543	50	5	25584.282	$11197_{0}^{{}^{\circ}}$ – 36781_5_
3907.352	2		25585.532	$13175_{4}^{{}^{\circ}}$ – 38760_4_
3907.160	200	10	25586.790	13088_3_ – $38675_{3}^{{}^{\circ}}$
3903.481	300	3	25610.905	2869_3_ – $28480_{4}^{{}^{\circ}}$
3903.315	200	5	25611.994	$15618_{3}^{{}^{\circ}}$ – 41230_4_
3903.103	1000r	150	25613.385	$7795_{4}^{{}^{\circ}}$- 33408_3_
3901.957	20	2	25620.907	13847_2_ – $39468_{3}^{{}^{\circ}}$
3901.662	300	15	25622.844	$7795_{4}^{{}^{\circ}}$ – 33418_4_
3900.577	600r	50	25629.971	8800_4_ – $34430_{3}^{{}^{\circ}}$
3900.464	40	1	25630.714	$14206_{4}^{{}^{\circ}}$ – 39837_3_
3898.795	300	100	25641.686	15493_4_ – $41134_{4}^{{}^{\circ}}$
3898.509	300b	100	25643.567	$11241_{3}^{{}^{\circ}}$ – 36885_4_
3898.438	500r	25	25644.034	2869_3_ – $28513_{2}^{{}^{\circ}}$
3897.778	200	5	25648.376	$14032_{2}^{{}^{\circ}}$ – 39680_2_
3895.418	2000r	200	25663.914	$7795_{4}^{{}^{\circ}}$ – 33459_4_
3894.601	8		25669.298	14204_5_ – $39873_{0}^{{}^{\circ}}$
3893.817	200	8	25674.466	7280_2_ – $32954_{2}^{{}^{\circ}}$
3893.651	300	40	25675.561	$10414_{4}^{{}^{\circ}}$ – 36089_4_
3891.725	400	10	25688.267	$11197_{0}^{{}^{\circ}}$ – 36885_4_
3891.657	75	1	25688.716	8111_4_ – $33799_{4}^{{}^{\circ}}$
3891.520	300	3	25689.621	8111_4_ – $33800_{3}^{{}^{\circ}}$
3889.906	300	4	25700.279	13088_3_ – $38788_{3}^{{}^{\circ}}$
3889.715	100	2	25701.541	13297_4_ – $38998_{3}^{{}^{\circ}}$
3889.609	150	3	25702.242	$10783_{2}^{{}^{\circ}}$ – 36485_2_
3889.542	200	8	25702.685	14204_5_ – $39906_{4}^{{}^{\circ}}$
3889.432	15		25703.411	0_2_ – $25703_{2}^{{}^{\circ}}$
3887.220	40		25718.037	6362_2_ – $32080_{1}^{{}^{\circ}}$
3887.019	800r	100	25719.367	$10414_{4}^{{}^{\circ}}$ – 36133_5_
3886.917	800r	500	25720.042	2869_3_ – $28589_{3}^{{}^{\circ}}$
3886.064	75	25	25725.688	13088_3_ – $38814_{2}^{{}^{\circ}}$
3885.224	200	5	25731.250	3687_2_ – $29419_{2}^{{}^{\circ}}$
3885.066	200	10	25732.296	$7795_{4}^{{}^{\circ}}$ – 33527_4_
3884.985	25		25732.833	$10783_{2}^{{}^{\circ}}$ – 36515_3_
3884.629	200	8	25735.191	$13945_{3}^{{}^{\circ}}$ – 39680_2_
3884.629	200	8	25735.191	$14465_{2}^{{}^{\circ}}$ – 40200_3_
3883.767	200	15	25740.902	$15490_{0}^{{}^{\circ}}$ – 41230_4_
3883.536	200	5	25742.433	$15618_{3}^{{}^{\circ}}$ – 41361_3_
3882.324	200	8	25750.470	15970_3_ – $41720_{2}^{{}^{\circ}}$
3880.404	50	2	25763.211	7280_2_ – $33043_{3}^{{}^{\circ}}$
3880.323	100	3	25763.748	13847_2_ – $39611_{3}^{{}^{\circ}}$
3880.194	300	15	25764.605	13297_4_ – $39062_{0}^{{}^{\circ}}$
3879.644	1000r	200	25768.257	7502_3_ – $33270_{4}^{{}^{\circ}}$
3879.445	400b	10	25769.579	$7795_{4}^{{}^{\circ}}$ – 33564_3_
3879.269	400	25	25770.748	$10526_{3}^{{}^{\circ}}$ – 36297_4_
3878.662	400	150	25774.781	3865_1_ – $29640_{1}^{{}^{\circ}}$
3878.160	100	3	25778.117	$13175_{4}^{{}^{\circ}}$ – 38953_5_
3877.610	500b	15	25781.774	$13175_{4}^{{}^{\circ}}$ – 38956_4_
3877.462	500	20	25782.758	8800_4_ – $34583_{3}^{{}^{\circ}}$
3875.646	500r	25	25794.838	7502_3_ – $33297_{2}^{{}^{\circ}}$
3875.374	1500r	200	25796.649	$10414_{4}^{{}^{\circ}}$ – 36210_5_
3875.157	300	2	25798.093	6362_2_ – $32160_{2}^{{}^{\circ}}$
3874.862	1500r	200	25800.057	4961_4_ – $30761_{3}^{{}^{\circ}}$
3874.244	1000r	50	25804.173	2869_3_ – $28673_{2}^{{}^{\circ}}$
3873.821	2000r	500	25806.990	2869_3_ – $28676_{3}^{{}^{\circ}}$
3873.473	500r	50	25809.309	0_2_ – $25809_{1}^{{}^{\circ}}$
3873.421	400b	20	25809.655	$10526_{3}^{{}^{\circ}}$ – 36336_3_
3873.147	400	10	25811.481	2869_3_ – $28680_{4}^{{}^{\circ}}$
3872.861	200	4	25813.387	11802_2_ – $37616_{1}^{{}^{\circ}}$
3872.672	200b	5	25814.647	2558_0_ – $28372_{1}^{{}^{\circ}}$
3870.891	75		25826.524	4961_4_ – $30788_{0}^{{}^{\circ}}$
3870.763	75	2	25827.378	12847_3_ – $38675_{3}^{{}^{\circ}}$
3869.663	800r	50	25834.720	6362_2_ – $32197_{3}^{{}^{\circ}}$
3868.254	400	10	25844.130	$11241_{3}^{{}^{\circ}}$ – 37085_4_
3868.040	100	3	25845.559	$14206_{4}^{{}^{\circ}}$ – 40052_4_
3867.974	100	200	25846.001	$18011_{0}^{{}^{\circ}}$ – 43857_5_
3867.069	40	10	25852.049	$16783_{4}^{{}^{\circ}}$ – 42635_5_
3866.907	500b	50	25853.132	$11241_{3}^{{}^{\circ}}$ – 37094_2_
3866.771	200	2	25854.041	11802_2_ – $37656_{3}^{{}^{\circ}}$
3866.375	100	2	25856.689	15863_2_ – $41720_{2}^{{}^{\circ}}$
3866.338	40	1	25856.937	8111_4_ – $33967_{3}^{{}^{\circ}}$
3864.970	200b	40b	25866.089	$15490_{0}^{{}^{\circ}}$ – 41356_5_
3862.654	200b	200	25881.597	7280_2_ – $33161_{1}^{{}^{\circ}}$
3862.419	500b	250b	25883.172	$10414_{4}^{{}^{\circ}}$ – 36297_4_
3861.574	300b	40	25888.835	$11197_{0}^{{}^{\circ}}$ – 37085_4_
3861.502	300b	40	25889.318	$11241_{3}^{{}^{\circ}}$ – 37131_3_
3861.351	300	10	25890.331	8111_4_ – $34001_{4}^{{}^{\circ}}$
3861.051	200	2	25892.342	$13945_{3}^{{}^{\circ}}$ – 39837_3_
3859.289	20		25904.163	8800_4_ – $34704_{3}^{{}^{\circ}}$
3858.927	100b	10	25906.593	$11877_{1}^{{}^{\circ}}$ – 37784_2_
3858.358	200	150	25910.414	13088_3_ – $38998_{3}^{{}^{\circ}}$
3856.622	300	15	25922.077	$10414_{4}^{{}^{\circ}}$ – 36336_3_
3856.516	300	50	25922.789	6362_2_ – $32285_{3}^{{}^{\circ}}$
3856.354	400r	40	25923.878	$8243_{2}^{{}^{\circ}}$ – 34167_3_
3856.222	100	8b	25924.766	7502_3_ – $33427_{2}^{{}^{\circ}}$
3853.985	100	8	25939.813	$16346_{4}^{{}^{\circ}}$ – 42286_3_
3853.825	200b	15	25940.890	12847_3_ – $38788_{3}^{{}^{\circ}}$
3852.135	800r	50	25952.270	3687_2_ – $29640_{1}^{{}^{\circ}}$
3851.155	300	15	25958.874	$10526_{3}^{{}^{\circ}}$ – 36485_2_
3851.072	300	20	25959.434	$15618_{3}^{{}^{\circ}}$ – 41578_2_
3850.058	20		25966.270	12847_3_ – $38814_{2}^{{}^{\circ}}$
3849.910	400	15	25967.269	13297_4_ – $39264_{3}^{{}^{\circ}}$
3847.617	500	10	25982.743	$12114_{2}^{{}^{\circ}}$ – 38097_2_
3846.886	1000r	100	25987.681	3865_1_ – $29853_{2}^{{}^{\circ}}$
3846.625	400	50	25989.444	$10526_{3}^{{}^{\circ}}$ – 36515_3_
3846.024	15		25993.505	$14206_{4}^{{}^{\circ}}$ – 40200_3_
3845.302	100	2	25998.386	3687_2_ – $29686_{3}^{{}^{\circ}}$
3845.092	400	10	25999.805	$13175_{4}^{{}^{\circ}}$ – 39174_3_
3843.019	300	40	26013.830	13297_4_ – $39311_{4}^{{}^{\circ}}$
3842.898	400r	75b	26014.649	$11197_{0}^{{}^{\circ}}$ – 37211_5_
3842.803	200	5	26015.292	11601_1_ – $37616_{1}^{{}^{\circ}}$
3842.742	200	4	26015.705	2869_3_ – $28884_{3}^{{}^{\circ}}$
3842.549	400	40	26017.012	7280_2_ – $33297_{2}^{{}^{\circ}}$
3840.918	25	10	26028.059	$14465_{2}^{{}^{\circ}}$ – 40493_3_
3840.799	600r	40	26028.866	4961_4_ – $30990_{3}^{{}^{\circ}}$
3840.430	300	250	26031.367	$12114_{2}^{{}^{\circ}}$ – 38145_3_
3839.898	50	10	26034.973	$10783_{2}^{{}^{\circ}}$ – 36818_2_
3839.695	800r	200	26036.349	0_2_ – $26036_{3}^{{}^{\circ}}$
3837.875	1500r	200	26048.696	2869_3_ – $28917_{2}^{{}^{\circ}}$
3837.024	200	3	26054.473	$8243_{2}^{{}^{\circ}}$ – 34298_1_
3836.721	300	20	26056.531	3687_2_ – $29744_{3}^{{}^{\circ}}$
3836.584	800r	100	26057.461	4961_4_ – $31019_{4}^{{}^{\circ}}$
3836.542	1000r	150b	26057.747	7502_3_ – $33560_{4}^{{}^{\circ}}$
3835.960	75	8	26061.700	$11241_{3}^{{}^{\circ}}$ – 37303_4_
3835.711	200	5	26063.392	2869_3_ – $28932_{4}^{{}^{\circ}}$
3835.414	200	10	26065.410	$11241_{3}^{{}^{\circ}}$ – 37307_3_
3835.349	200	10	26065.852	14204_5_ – $40270_{4}^{{}^{\circ}}$
3835.077	100	2	26067.700	$15618_{3}^{{}^{\circ}}$ – 41686_3_
3834.890	100	5	26068.971	$16217_{2}^{{}^{\circ}}$ – 42286_3_
3834.488	100	75	26071.704	8111_4_ – $34182_{0}^{{}^{\circ}}$
3832.323	75	3b	26086.433	8800_4_ – $34886_{6}^{{}^{\circ}}$
3831.962	100	2	26088.890	7502_3_ – $33591_{3}^{{}^{\circ}}$
3831.640	300	50	26091.083	2558_0_ – $28649_{1}^{{}^{\circ}}$
3830.774	800r	150	26096.981	0_2_ – $26096_{3}^{{}^{\circ}}$
3830.198	50	2	26100.905	$14481_{6}^{{}^{\circ}}$ – 40582_5_
3830.061	500r	50	26101.839	$10414_{4}^{{}^{\circ}}$ – 36515_3_
3829.393	50b		26106.392	$11197_{0}^{{}^{\circ}}$ – 37303_4_
3829.283	150b	4b	26107.142	$13945_{3}^{{}^{\circ}}$ – 40052_4_
3828.385	3000r	300	26113.265	0_2_ – $26113_{2}^{{}^{\circ}}$
3826.368	400r	75	26127.030	$10526_{3}^{{}^{\circ}}$ – 36653_2_
3825.132	500r	200	26135.472	$11197_{0}^{{}^{\circ}}$ – 37332_6_
3824.927	20	2	26136.873	$15618_{3}^{{}^{\circ}}$ – 41755_4_
3823.527	10		26146.443	$14465_{2}^{{}^{\circ}}$ – 40611_2_
3823.458	300	25	26146.915	7280_2_ – $33427_{2}^{{}^{\circ}}$
3823.067	500r	100	26149.589	5563_1_ – $31712_{1}^{{}^{\circ}}$
3822.863	300	40	26150.984	12847_3_ – $38998_{3}^{{}^{\circ}}$
3822.709	300	25	26152.038	11802_2_ – $37954_{3}^{{}^{\circ}}$
3821.119	15		26162.920	$11241_{3}^{{}^{\circ}}$ – 37404_3_
3820.792	600r	75	26165.159	3687_2_ – $29853_{2}^{{}^{\circ}}$
3820.474	300b	10	26167.337	11802_2_ – $37970_{2}^{{}^{\circ}}$
3820.327	200	2	26168.343	$14032_{2}^{{}^{\circ}}$ – 40200_3_
3819.904	500	100b	26171.241	13297_4_ – $39468_{3}^{{}^{\circ}}$
3819.189	200	10	26176.141	13088_3_ – $39264_{3}^{{}^{\circ}}$
3819.112	300	10	26176.668	$13175_{4}^{{}^{\circ}}$ – 39351_5_
3818.685	400r	15	26179.595	4961_4_ – $31141_{0}^{{}^{\circ}}$
3817.495	100		26187.756	$8243_{2}^{{}^{\circ}}$ – 34431_3_
3816.510	15	1	26194.514	$15166_{3}^{{}^{\circ}}$ – 41361_3_
3815.566	500	50	26200.995	$8243_{2}^{{}^{\circ}}$ – 34444_2_
3813.815	500r	50	26213.024	6362_2_ – $32575_{2}^{{}^{\circ}}$
3812.905	75	2	26219.280	$11877_{1}^{{}^{\circ}}$ – 38097_2_
3812.827	15		26219.816	$14206_{4}^{{}^{\circ}}$ – 40426_4_
3812.667	75	5	26220.917	14204_5_ – $40425_{4}^{{}^{\circ}}$
3812.398	400	40	26222.767	$15490_{0}^{{}^{\circ}}$ – 41712_5_
3810.995	600r	150	26232.420	7502_3_ – $33734_{2}^{{}^{\circ}}$
3810.906	75	1	26233.033	4961_4_ – $31194_{4}^{{}^{\circ}}$
3808.614	500	20	26248.819	$10526_{3}^{{}^{\circ}}$ – 36775_3_
3808.430	15		26250.088	11802_2_ – $38053_{3}^{{}^{\circ}}$
3807.700	200	5b	26255.120	$13945_{3}^{{}^{\circ}}$ – 40200_3_
3807.273	400	50	26258.065	9804_5_ – $36062_{4}^{{}^{\circ}}$
3806.317	100	1	26264.660	13297_4_ – $39562_{4}^{{}^{\circ}}$
3806.153	200	8	26265.791	$15490_{0}^{{}^{\circ}}$ – 41755_4_
3805.227	200	10	26272.183	16351_0_ – $42624_{1}^{{}^{\circ}}$
3804.127	400	20	26279.779	$11241_{3}^{{}^{\circ}}$ – 37521_3_
3803.985	600r	150	26280.761	8800_4_ – $35081_{0}^{{}^{\circ}}$
3803.839	400	25	26281.769	$10526_{3}^{{}^{\circ}}$ – 36808_3_
3803.173	5b	3	26286.371	$14206_{4}^{{}^{\circ}}$ – 40493_3_
3803.075	3000r	200	26287.049	0_2_ – $26287_{1}^{{}^{\circ}}$
3802.864	500	10	26288.507	6362_2_ – $32650_{1}^{{}^{\circ}}$
3802.589	3		26290.408	$15736_{1}^{{}^{\circ}}$ – 42027_2_
3802.420	400	25	26291.577	$10526_{3}^{{}^{\circ}}$ – 36818_2_
3801.571	400r	100	26297.448	7502_3_ – $33799_{4}^{{}^{\circ}}$
3801.443	800r	100	26298.334	7502_3_ – $33800_{3}^{{}^{\circ}}$
3800.745	2		26303.163	6362_2_ – $32665_{1}^{{}^{\circ}}$
3800.197	400	15	26306.956	5563_1_ – $31870_{2}^{{}^{\circ}}$
3799.166	75	1	26314.095	13297_4_ – $39611_{3}^{{}^{\circ}}$
3798.432	200	75	26319.180	8111_4_ – $34430_{3}^{{}^{\circ}}$
3798.103	500r	150	26321.460	4961_4_ – $31283_{3}^{{}^{\circ}}$
3797.205	200	10	26327.684	9804_5_ – $36132_{0}^{{}^{\circ}}$
3796.997	200	10	26329.126	3687_2_ – $30017_{3}^{{}^{\circ}}$
3796.731	500b	20	26330.971	8800_4_ – $35131_{4}^{{}^{\circ}}$
3794.981	200	3	26343.113	3865_1_ – $30208_{2}^{{}^{\circ}}$
3794.697	1500r	100	26345.084	$13175_{4}^{{}^{\circ}}$ – 39520_4_
3793.097	300	10	26356.197	$17501_{0}^{{}^{\circ}}$ – 43857_5_
3792.730	4001	75	26358.747	$10526_{3}^{{}^{\circ}}$ – 36885_4_
3792.371	1000r	100	26361.242	$10414_{4}^{{}^{\circ}}$ – 36775_3_
3792.104	150	3	26363.098	0_2_ – $26363_{2}^{{}^{\circ}}$
3791.517	300	10	26367.180	$10414_{4}^{{}^{\circ}}$ – 36781_5_
3791.221	150	8	26369.238	11601_1_ – $37970_{2}^{{}^{\circ}}$
3790.794	1500r	200	26372.209	$7795_{4}^{{}^{\circ}}$ – 34167_3_
3790.646	100	5	26373.238	9804_5_ – $36178_{4}^{{}^{\circ}}$
3790.269	300	10	26375.861	$14206_{4}^{{}^{\circ}}$ – 40582_5_
3789.659	50	1	26380.107	13088_3_ – $39468_{3}^{{}^{\circ}}$
3789.529	75	2	26381.012	8111_4_ – $34492_{4}^{{}^{\circ}}$
3789.372	300	10	26382.105	7280_2_ – $33662_{1}^{{}^{\circ}}$
3789.167	500r	100	26383.532	2869_3_ – $29252_{2}^{{}^{\circ}}$
3787.934	300	3	26392.120	6362_2_ – $32754_{3}^{{}^{\circ}}$
3787.638	300	40	26394.182	$10414_{4}^{{}^{\circ}}$ – 36808_3_
3785.771	100	2	26407.199	13847_2_ – $40254_{3}^{{}^{\circ}}$
3785.154	200	2	26411.503	$15166_{3}^{{}^{\circ}}$ – 41578_2_
3784.793	300	15	26414.022	11802_2_ – $38216_{3}^{{}^{\circ}}$
3784.574	400	20	26415.551	3865_1_ – $30281_{1}^{{}^{\circ}}$
3784.574	400	20	26415.551	$12114_{2}^{{}^{\circ}}$ – 38529_3_
3784.406	100	3	26416.723	12847_3_ – $39264_{3}^{{}^{\circ}}$
3783.680	50b		26421.792	$14465_{2}^{{}^{\circ}}$ – 40886_2_
3781.638	10		26436.059	$17501_{0}^{{}^{\circ}}$ – 43937_6_
3781.318	300	75	26438.296	7280_2_ – $33718_{2}^{{}^{\circ}}$
3780.966	300r	20b	26440.757	2869_3_ – $29310_{4}^{{}^{\circ}}$
3780.147	200	8	26446.486	$11241_{3}^{{}^{\circ}}$ – 37688_3_
3778.987	200	3	26454.603	7280_2_ – $33734_{2}^{{}^{\circ}}$
3778.045	300	15	26461.199	$14032_{2}^{{}^{\circ}}$ – 40493_3_
3777.745	200	40	26463.301	12847_3_ – $39311_{4}^{{}^{\circ}}$
3777.626	50b		26464.134	$8243_{2}^{{}^{\circ}}$ – 34707_3_
3777.415	300	40	26465.612	7502_3_ – $33967_{3}^{{}^{\circ}}$
3776.732	10		26470.399	9804_5_ – $36275_{0}^{{}^{\circ}}$
3776.622	300	40b	26471.169	$10414_{4}^{{}^{\circ}}$ – 36885_4_
3776.502	300	8	26472.010	8111_4_ – $34583_{3}^{{}^{\circ}}$
3776.271	600r	150	26473.630	8800_4_ – $35273_{0}^{{}^{\circ}}$
3775.937	15		26475.971	11802_2_ – $38278_{1}^{{}^{\circ}}$
3775.157	150	15	26481.442	$13945_{3}^{{}^{\circ}}$ – 40426_4_
3774.223	50b		26487.995	11601_1_ – $38088_{2}^{{}^{\circ}}$
3774.133	100	8	26488.627	$12114_{2}^{{}^{\circ}}$ – 38602_2_
3773.306	200	8	26494.432	$14206_{4}^{{}^{\circ}}$ – 40701_4_
3772.649	400	10	26499.046	7502_3_ – $34001_{4}^{{}^{\circ}}$
3771.629	100b	5	26506.212	$7795_{4}^{{}^{\circ}}$ – 34301_4_
3771.616	300b	10	26506.303	$11241_{3}^{{}^{\circ}}$ – 37748_2_
3771.370	1500r	100	26508.032	0_2_ – $26508_{3}^{{}^{\circ}}$
3770.056	1500r	200	26517.271	5563_1_ – $32080_{1}^{{}^{\circ}}$
3769.778	50	1	26519.226	$13175_{4}^{{}^{\circ}}$ – 39694_5_
3769.697	75	10	26519.796	$15166_{3}^{{}^{\circ}}$ – 41686_3_
3769.615	200b	40	26520.373	14204_5_ – $40724_{0}^{{}^{\circ}}$
3769.592	400b	50	26520.535	7280_2_ – $33800_{3}^{{}^{\circ}}$
3769.101	75	1	26523.990	$10783_{2}^{{}^{\circ}}$ – 37307_3_
3766.447	300	10	26542.679	$11241_{3}^{{}^{\circ}}$ – 37784_2_
3766.083	300b	50	26545.244	$11197_{0}^{{}^{\circ}}$ – 37742_6_
3765.696	200	4	26547.972	$13945_{3}^{{}^{\circ}}$ – 40493_3_
3765.412	400r	50	26549.975	2869_3_ – $29419_{2}^{{}^{\circ}}$
3765.241	800r	200	26551.181	8800_4_ – $35351_{4}^{{}^{\circ}}$
3765.067	150	2	26552.408	11802_2_ – $38355_{2}^{{}^{\circ}}$
3764.089	4		26559.306	$10526_{3}^{{}^{\circ}}$ – 37085_4_
3763.668	300	10	26562.277	4961_4_ – $31523_{3}^{{}^{\circ}}$
3762.934	500r	100	26567.458	3687_2_ – $30255_{3}^{{}^{\circ}}$
3762.815	300b	25	26568.298	$10526_{3}^{{}^{\circ}}$ – 37094_2_
3762.257	200	8	26572.239	14204_5_ – $40776_{4}^{{}^{\circ}}$
3761.704	200	20	26576.145	13297_4_ – $39873_{0}^{{}^{\circ}}$
3761.527	200	20	26577.396	$11241_{3}^{{}^{\circ}}$ – 37819_4_
3761.470	200	25	26577.798	9804_5_ – $36382_{4}^{{}^{\circ}}$
3761.217	200	8	26579.586	$14032_{2}^{{}^{\circ}}$ – 40611_2_
3759.886	50		26588.995	$15166_{3}^{{}^{\circ}}$ – 41755_4_
3759.433	500r	75	26592.199	6362_2_ – $32954_{2}^{{}^{\circ}}$
3759.314	200r	100	26593.041	3687_2_ – $30281_{1}^{{}^{\circ}}$
3759.262	300r	100	26593.408	8111_4_ – $34704_{3}^{{}^{\circ}}$
3758.706	400	20	26597.342	5563_1_ – $32160_{2}^{{}^{\circ}}$
3758.467	400r	25	26599.033	2558_0_ – $29157_{1}^{{}^{\circ}}$
3757.694	1000r	200	26604.505	$10526_{3}^{{}^{\circ}}$ – 37131_3_
3756.451	200	8	26613.308	11802_2_ – $38416_{3}^{{}^{\circ}}$
3756.293	400	25	26614.428	8111_4_ – $34725_{3}^{{}^{\circ}}$
3755.498	25s	3	26620.061	$12114_{2}^{{}^{\circ}}$ – 38734_3_
3755.211	800r	250	26622.096	$11197_{0}^{{}^{\circ}}$ – 37819_4_
3754.030	500r	50	26630.471	$8243_{2}^{{}^{\circ}}$ – 34874_3_
3753.241	300	40	26636.069	$7795_{4}^{{}^{\circ}}$ – 34431_3_
3752.789	300	8	26639.277	2558_0_ – $29197_{1}^{{}^{\circ}}$
3752.265	10	1	26642.997	$14243_{1}^{{}^{\circ}}$ – 40886_2_
3751.121	300	25	26651.122	$11241_{3}^{{}^{\circ}}$ – 37892_4_
3751.021	300r	10	26651.833	0_2_ – $26651_{2}^{{}^{\circ}}$
3749.617	300	20	26661.812	8800_4_ – $35462_{3}^{{}^{\circ}}$
3749.514	300	10	26662.544	$13175_{4}^{{}^{\circ}}$ – 39837_3_
3749.083	200	25	26665.609	$7795_{4}^{{}^{\circ}}$ – 34460_5_
3748.820	100	8	26667.480	$15618_{3}^{{}^{\circ}}$ – 42286_3_
3748.225	20b	5	26671.713	$10414_{4}^{{}^{\circ}}$ – 37085_4_
3746.929	4		26680.938	6362_2_ – $33043_{3}^{{}^{\circ}}$
3745.176	4001	20	26693.427	$11197_{0}^{{}^{\circ}}$ – 37890_5_
3744.847	100	5	26695.772	$11197_{0}^{{}^{\circ}}$ – 37892_4_
3742.923	1000r	100	26709.494	4961_4_ – $31671_{4}^{{}^{\circ}}$
3742.275	200	5	26714.119	12847_3_ – $39562_{4}^{{}^{\circ}}$
3741.884	200	8	26716.910	$10414_{4}^{{}^{\circ}}$ – 37131_3_
3740.731	40	2	26725.145	$11877_{1}^{{}^{\circ}}$ – 38602_2_
3737.514	600r	75	26748.147	3865_1_ – $30613_{0}^{{}^{\circ}}$
3736.655	100	5	26754.296	11601_1_ – $38355_{2}^{{}^{\circ}}$
3736.566	50	1	26754.933	4961_4_ – $31716_{0}^{{}^{\circ}}$
3736.411	5		26756.043	$13945_{3}^{{}^{\circ}}$ – 40701_4_
3735.827	50b	2	26760.226	15863_2_ – $42624_{1}^{{}^{\circ}}$
3735.363	100	3	26763.550	12847_3_ – $39611_{3}^{{}^{\circ}}$
3733.672	300r	25	26775.671	8111_4_ – $34886_{0}^{{}^{\circ}}$
3733.501	10	1	26776.897	$10526_{3}^{{}^{\circ}}$ – 37303_4_
3732.985	400r	40	26780.599	$10526_{3}^{{}^{\circ}}$ – 37307_3_
3730.946	50	3	26795.234	$11197_{0}^{{}^{\circ}}$ – 37992_4_
3730.623	75	4	26797.554	$10414_{4}^{{}^{\circ}}$ – 37211_5_
3730.368	800r	2001	26799.386	6362_2_ – $33161_{1}^{{}^{\circ}}$
3729.945	400	40	26802.425	$8243_{2}^{{}^{\circ}}$ – 35046_1_
3727.903	1000r	150	26817.106	2869_3_ – $29686_{3}^{{}^{\circ}}$
3727.725	75	2	26818.386	13088_3_ – $39906_{4}^{{}^{\circ}}$
3727.612	400r	40	26819.199	4961_4_ – $31780_{3}^{{}^{\circ}}$
3727.346	300	40	26821.113	9804_5_ – $36625_{0}^{{}^{\circ}}$
3727.251	300	40	26821.797	7502_3_ – $34324_{2}^{{}^{\circ}}$
3725.966	75	2	26831.047	7280_2_ – $34111_{1}^{{}^{\circ}}$
3724.397	100	2	26842.350	7502_3_ – $34344_{4}^{{}^{\circ}}$
3723.921	300s	8	26845.781	$8243_{2}^{{}^{\circ}}$ – 35089_3_
3722.655	75	2	26854.910	$14032_{2}^{{}^{\circ}}$ – 40886_2_
3722.591	100b	5	26855.372	$11241_{3}^{{}^{\circ}}$ – 38097_2_
3722.179	300b	25b	26858.344	3865_1_ – $30723_{1}^{{}^{\circ}}$
3721.881	10b		26860.495	$15166_{3}^{{}^{\circ}}$ – 42027_2_
3721.219	500r	50	26865.273	3687_2_ – $30553_{2}^{{}^{\circ}}$
3721.003	100	10	26866.833	11802_2_ – $38669_{2}^{{}^{\circ}}$
3720.628	20	15	26869.540	7502_3_ – $34371_{2}^{{}^{\circ}}$
3719.837	400r	40	26875.254	2869_3_ – $29744_{3}^{{}^{\circ}}$
3719.546	300	15	26877.356	$13175_{4}^{{}^{\circ}}$ – 40052_4_
3719.435	3000	200	26878.158	0_2_ – $26878_{3}^{{}^{\circ}}$
3717.895	50	2	26889.291	$10414_{4}^{{}^{\circ}}$ – 37303_4_
3717.384	100	3	26892.988	$10414_{4}^{{}^{\circ}}$ – 37307_3_
3716.992	100b	4	26895.824	$12114_{2}^{{}^{\circ}}$ – 39010_3_
3716.942	100	2	26896.186	$14465_{2}^{{}^{\circ}}$ – 41361_3_
3716.808	100	2	26897.155	13088_3_ – $39985_{3}^{{}^{\circ}}$
3715.862	300	20	26904.003	$11241_{3}^{{}^{\circ}}$ – 38145_3_
3715.561	400	50	26906.182	$8243_{2}^{{}^{\circ}}$ – 35149_2_
3715.060	100	2	26909.810	$11197_{0}^{{}^{\circ}}$ – 38106_5_
3714.696	100	3	26912.447	$7795_{4}^{{}^{\circ}}$ – 34707_3_
3712.299	40	2	26929.824	$11877_{1}^{{}^{\circ}}$ – 38807_2_
3712.187	25	8	26930.636	14204_5_ – $41134_{4}^{{}^{\circ}}$
3711.926	200	5	26932.530	6362_2_ – $33294_{3}^{{}^{\circ}}$
3711.833	200	15	26933.205	8800_4_ – $35733_{4}^{{}^{\circ}}$
3711.623	600r	50	26934.729	6362_2_ – $33297_{2}^{{}^{\circ}}$
3711.357	75	4b	26936.659	$17501_{0}^{{}^{\circ}}$ – 44437_6_
3710.830	100	1	26940.484	5563_1_ – $32503_{2}^{{}^{\circ}}$
3710.665	1001	2	26941.682	$13945_{3}^{{}^{\circ}}$ – 40886_2_
3710.292	300	8	26944.391	$11241_{3}^{{}^{\circ}}$ – 38186_4_
3709.861	300	25	26947.521	3865_1_ – $30812_{2}^{{}^{\circ}}$
3708.483	75	2	26957.534	13297_4_ – $40254_{3}^{{}^{\circ}}$
3707.465	200	8	26964.936	$10783_{2}^{{}^{\circ}}$ – 37748_2_
3706.767	2000r	300	26970.013	8111_4_ – $35081_{0}^{{}^{\circ}}$
3706.402	200	75	26972.669	13297_4_ – $40270_{4}^{{}^{\circ}}$
3704.862	500r	50	26983.880	2869_3_ – $29853_{2}^{{}^{\circ}}$
3704.584	100	5	26985.905	11802_2_ – $38788_{3}^{{}^{\circ}}$
3704.147	200	50	26989.089	$11197_{0}^{{}^{\circ}}$ – 38186_4_
3703.951	75b		26990.517	$10414_{4}^{{}^{\circ}}$ – 37404_3_
3703.775	500r	50	26991.800	4961_4_ – $31953_{4}^{{}^{\circ}}$
3703.342	200	2	26994.955	$10526_{3}^{{}^{\circ}}$ – 37521_3_
3703.230	400r	25	26995.772	0_2_ – $26995_{3}^{{}^{\circ}}$
3702.479	100	2	27001.247	$10783_{2}^{{}^{\circ}}$ – 37784_2_
3701.594	150b	3	27007.703	13088_3_ – $40096_{2}^{{}^{\circ}}$
3701.099	200	2	27011.315	11802_2_ – $38814_{2}^{{}^{\circ}}$
3700.978	500r	100	27012.198	2869_3_ – $29881_{4}^{{}^{\circ}}$
3699.881	500r	50	27020.207	8111_4_ – $35131_{4}^{{}^{\circ}}$
3699.355	300	5	27024.048	$14206_{4}^{{}^{\circ}}$ – 41230_4_
3699.182	500	75	27025.312	$13175_{4}^{{}^{\circ}}$ – 40200_3_
3698.106	1000r	150	27033.176	9804_5_ – $36837_{0}^{{}^{\circ}}$
3697.743	300	10	27035.829	3687_2_ – $30723_{1}^{{}^{\circ}}$
3697.497	300	3	27037.628	11802_2_ – $38840_{1}^{{}^{\circ}}$
3695.288	500r	75	27053.790	$7795_{4}^{{}^{\circ}}$ – 34849_5_
3694.579	50	2	27058.982	12847_3_ – $39906_{4}^{{}^{\circ}}$
3694.365	300r	50	27060.549	$12114_{2}^{{}^{\circ}}$ – 39174_3_
3694.246	300r	10	27061.421	0_2_ – $27061_{2}^{{}^{\circ}}$
3694.177	400r	75	27061.926	4961_4_ – $32023_{0}^{{}^{\circ}}$
3693.993	400r	200	27063.274	3865_1_ – $30928_{1}^{{}^{\circ}}$
3693.804	200b	25	27064.659	6362_2_ – $33427_{2}^{{}^{\circ}}$
3693.247	200	10	27068.741	11601_1_ – $38669_{2}^{{}^{\circ}}$
3692.566	1000r	100	27073.733	3687_2_ – $30761_{3}^{{}^{\circ}}$
3691.972	300b	5	27078.088	$7795_{4}^{{}^{\circ}}$ – 34873_4_
3691.876	400r	100	27078.792	$7795_{4}^{{}^{\circ}}$ – 34874_3_
3691.613	500r	200	27080.722	7502_3_ – $34583_{3}^{{}^{\circ}}$
3691.411	500r	50	27082.203	2558_0_ – $29640_{1}^{{}^{\circ}}$
3690.649	100b	8b	27087.795	5563_1_ – $32650_{1}^{{}^{\circ}}$
3690.624	600r	4	27087.978	0_2_ – $27087_{1}^{{}^{\circ}}$
3690.115	200	300	27091.715	7280_2_ – $34371_{2}^{{}^{\circ}}$
3688.658	300	20	27102.416	5563_1_ – $32665_{1}^{{}^{\circ}}$
3687.983	400r	75	27107.376	$10414_{4}^{{}^{\circ}}$ – 37521_3_
3687.193	4001	20	27113.184	$14465_{2}^{{}^{\circ}}$ – 41578_2_
3686.326	2		27119.560	$15166_{3}^{{}^{\circ}}$ – 42286_3_
3685.586	300b	101	27125.005	3687_2_ – $30812_{2}^{{}^{\circ}}$
3685.214	200	4	27127.743	13297_4_ – $40425_{4}^{{}^{\circ}}$
3684.932	300	20	27129.819	8111_4_ – $35240_{3}^{{}^{\circ}}$
3683.856	20		27137.743	12847_3_ – $39985_{3}^{{}^{\circ}}$
3682.759	10b		27145.827	$15490_{0}^{{}^{\circ}}$ – 42635_5_
3682.486	800r	50	27147.839	2869_3_ – $30017_{3}^{{}^{\circ}}$
3682.295	300	20	27149.247	$14206_{4}^{{}^{\circ}}$ – 41356_5_
3682.179	300	50	27150.103	7280_2_ – $34430_{3}^{{}^{\circ}}$
3680.605	400r	40	27161.713	$8243_{2}^{{}^{\circ}}$ – 35405_3_
3680.448	500r	50	27162.871	8111_4_ – $35273_{0}^{{}^{\circ}}$
3679.970	75	2	27166.400	13088_3_ – $40254_{3}^{{}^{\circ}}$
3679.544	400r	150	27169.545	$11197_{0}^{{}^{\circ}}$ – 38366_4_
3678.478	400r	50b	27177.418	$8243_{2}^{{}^{\circ}}$ – 35421_1_
3675.960	400	50	27196.034	11802_2_ – $38998_{3}^{{}^{\circ}}$
3675.790	400r	50	27197.292	$8243_{2}^{{}^{\circ}}$ – 35440_3_
3675.137	500r	50	27202.124	7502_3_ – $34704_{3}^{{}^{\circ}}$
3674.891	300r	50	27203.945	9804_5_ – $37008_{0}^{{}^{\circ}}$
3674.420	50	5	27207.432	$12114_{2}^{{}^{\circ}}$ – 39321_3_
3674.013	300r	50b	27210.446	3865_1_ – $31075_{2}^{{}^{\circ}}$
3673.639	50	1	27213.216	11601_1_ – $38814_{2}^{{}^{\circ}}$
3672.522	500	25	27221.493	$10526_{3}^{{}^{\circ}}$ – 37748_2_
3672.522	500	25	27221.493	$14465_{2}^{{}^{\circ}}$ – 41686_3_
3672.300	500r	50	27223.138	7502_3_ – $34725_{3}^{{}^{\circ}}$
3671.540	600r	150	27228.773	6362_2_ – $33591_{3}^{{}^{\circ}}$
3671.242	75	2	27230.983	$14481_{6}^{{}^{\circ}}$ – 41712_5_
3670.520	200	4	27236.340	9804_5_ – $37041_{4}^{{}^{\circ}}$
3669.968	2000r	100	27240.436	8111_4_ – $35351_{4}^{{}^{\circ}}$
3668.914	100	4	27248.261	12847_3_ – $40096_{2}^{{}^{\circ}}$
3667.621	500	40	27257.867	$10526_{3}^{{}^{\circ}}$ – 37784_2_
3667.311	5	1	27260.171	0_2_ – $27260_{3}^{{}^{\circ}}$
3666.981	800r	75	27262.625	8800_4_ – $36062_{4}^{{}^{\circ}}$
3665.476	100b	10b	27273.818	8800_4_ – $36074_{3}^{{}^{\circ}}$
3665.443	150b	500	27274.064	$10414_{4}^{{}^{\circ}}$ – 37688_3_
3663.543	300	15	27288.208	$11241_{3}^{{}^{\circ}}$ – 38529_3_
3663.201	1500r	100	27290.756	2869_3_ – $30160_{4}^{{}^{\circ}}$
3662.955	150	15	27292.589	$10526_{3}^{{}^{\circ}}$ – 37819_4_
3662.750	300r	20	27294.116	$7795_{4}^{{}^{\circ}}$ – 35089_3_
3662.283	100	3	27297.596	0_2_ – $27297_{1}^{{}^{\circ}}$
3661.982	150	15	27299.840	6362_2_ – $33662_{1}^{{}^{\circ}}$
3661.622	500r	75	27302.524	3687_2_ – $30990_{3}^{{}^{\circ}}$
3661.575	500r	200	27302.874	7280_2_ – $34583_{3}^{{}^{\circ}}$
3660.500	400	25	27310.892	7280_2_ – $34591_{1}^{{}^{\circ}}$
3660.091	200b	4	27313.944	$10783_{2}^{{}^{\circ}}$ – 38097_2_
3659.629	600r	150	27317.392	0_2_ – $27317_{3}^{{}^{\circ}}$
3658.808	400r	25	27323.522	4961_4_ – $32285_{3}^{{}^{\circ}}$
3657.641	200s	10	27332.240	8800_4_ – $36132_{0}^{{}^{\circ}}$
3657.054	300	20	27336.627	13088_3_ – $40425_{4}^{{}^{\circ}}$
3656.693	500r	50	27339.325	2869_3_ – $30208_{2}^{{}^{\circ}}$
3655.125	300	50	27351.053	8111_4_ – $35462_{3}^{{}^{\circ}}$
3654.915	3		27352.624	13847_2_ – $41200_{2}^{{}^{\circ}}$
3654.461	500r	50	27356.023	6362_2_ – $33718_{2}^{{}^{\circ}}$
3653.586	40		27362.574	$10783_{2}^{{}^{\circ}}$ – 38145_3_
3653.090	100	2	27366.289	$10526_{3}^{{}^{\circ}}$ – 37892_4_
365.9284	20	1	27372.328	6362_2_ – $33734_{2}^{{}^{\circ}}$
3650.436	200	50	27386.185	2869_3_ – $30255_{3}^{{}^{\circ}}$
3650.204	3001	2	27387.925	3687_2_ – $31075_{2}^{{}^{\circ}}$
3649.735	1000r	100	27391.445	5563_1_ – $32954_{2}^{{}^{\circ}}$
3647.932	300	50	27404.982	$10414_{4}^{{}^{\circ}}$ – 37819_4_
3647.662	25		27407.011	12847_3_ – $40254_{3}^{{}^{\circ}}$
3647.575	300	15	27407.665	$13175_{4}^{{}^{\circ}}$ – 40582_5_
3646.452	15		27416.105	$13945_{3}^{{}^{\circ}}$ – 41361_3_
3645.706	300	15	27421.715	$7795_{4}^{{}^{\circ}}$ – 35216_5_
3645.648	200	8	27422.151	12847_3_ – $40270_{4}^{{}^{\circ}}$
3645.365	200b	25b	27424.280	7280_2_ – $34704_{3}^{{}^{\circ}}$
3644.981	200	3	27427.169	13297_4_ – $40724_{0}^{{}^{\circ}}$
3643.512	600r	50	27438.227	6362_2_ – $33800_{3}^{{}^{\circ}}$
3642.573	500r	50	27445.300	7280_2_ – $34725_{3}^{{}^{\circ}}$
3642.249	2000r	100	27447.741	0_2_ – $27447_{2}^{{}^{\circ}}$
3640.820	10		27458.514	$11241_{3}^{{}^{\circ}}$ – 38700_4_
3640.390	100	3	27461.757	11802_2_ – $39264_{3}^{{}^{\circ}}$
3639.867	4001	40	27465.703	$10526_{3}^{{}^{\circ}}$ – 37992_4_
3638.644	500r	50	27474.935	8800_4_ – $36275_{0}^{{}^{\circ}}$
3638.460	40	2	27476.324	$10414_{4}^{{}^{\circ}}$ – 37890_5_
3638.319	400r	25	27477.389	4961_4_ – $32439_{4}^{{}^{\circ}}$
3638.146	200	4	27478.695	$10414_{4}^{{}^{\circ}}$ – 37892_4_
3638.097	150	4	27479.066	13297_4_ – $40776_{4}^{{}^{\circ}}$
3638.004	75	4	27479.768	$14206_{4}^{{}^{\circ}}$ – 41686_3_
3636.295	150	3	27492.682	$11241_{3}^{{}^{\circ}}$ – 38734_3_
3634.902	200	4	27503.218	$11197_{0}^{{}^{\circ}}$ – 38700_4_
3634.582	600r	100b	27505.640	$7795_{4}^{{}^{\circ}}$ – 35300_5_
3634.546	500b	50b	27505.912	$14206_{4}^{{}^{\circ}}$ – 41712_5_
3633.910	100	3	27510.726	$16346_{4}^{{}^{\circ}}$ – 43857_5_
3632.830	1000r	100	27518.905	$11241_{3}^{{}^{\circ}}$ – 38760_4_
3631.863	150	8	27526.231	$13175_{4}^{{}^{\circ}}$ – 40701_4_
3629.214	15		27546.323	$14032_{2}^{{}^{\circ}}$ – 41578_2_
3628.867	100	4	27548.956	$14206_{4}^{{}^{\circ}}$ – 41755_4_
3626.939	300	50	27563.600	$11197_{0}^{{}^{\circ}}$ – 38760_4_
3626.631	200b	10	27565.941	$11241_{3}^{{}^{\circ}}$ – 38807_2_
3626.613	50b		27566.078	$12114_{2}^{{}^{\circ}}$ – 39680_2_
3626.025	50	3	27570.548	$10526_{3}^{{}^{\circ}}$ – 38097_2_
3625.895	500r	400b	27571.537	7280_2_ – $34851_{2}^{{}^{\circ}}$
3625.150	300	75	27577.203	12847_3_ – $40425_{4}^{{}^{\circ}}$
3625.026	300r	100	27578.146	$10414_{4}^{{}^{\circ}}$ – 37992_4_
3624.472	300	50	27582.361	8800_4_ – $36382_{4}^{{}^{\circ}}$
3623.773	300	25	27587.681	$8243_{2}^{{}^{\circ}}$ – 35831_3_
3622.795	600r	50	27595.129	3687_2_ – $31283_{3}^{{}^{\circ}}$
3622.338	500r	20b	27598.610	5563_1_ – $33161_{1}^{{}^{\circ}}$
3621.435	50		27605.492	6362_2_ – $33967_{3}^{{}^{\circ}}$
3620.977	5		27608.983	11802_2_ – $39411_{1}^{{}^{\circ}}$
3620.839	300r	50	27610.035	$7795_{4}^{{}^{\circ}}$ – 35405_3_
3619.633	100	20	27619.234	$10526_{3}^{{}^{\circ}}$ – 38145_3_
3619.211	200	40	27622.455	8111_4_ – $35733_{4}^{{}^{\circ}}$
3618.597	200	10	27627.142	8800_4_ – $36427_{3}^{{}^{\circ}}$
3618.363	800r	50	27628.928	7502_3_ – $35131_{4}^{{}^{\circ}}$
3617.818	150	10	27633.090	$13945_{3}^{{}^{\circ}}$ – 41578_2_
3617.671	300	8	27634.213	$8243_{2}^{{}^{\circ}}$ – 35877_2_
3616.177	200	8	27645.629	$7795_{4}^{{}^{\circ}}$ – 35440_3_
3615.849	200	4	27648.137	2869_3_ – $30517_{4}^{{}^{\circ}}$
3615.004	100	5	27654.600	$14032_{2}^{{}^{\circ}}$ – 41686_3_
3614.352	500r	75	27659.588	$10526_{3}^{{}^{\circ}}$ – 38186_4_
3613.547	200	5	27665.750	11802_2_ – $39468_{3}^{{}^{\circ}}$
3612.864	600r	5	27670.980	0_2_ – $27670_{3}^{{}^{\circ}}$
3612.427	1500r	75	27674.327	0_2_ – $27674_{2}^{{}^{\circ}}$
3611.257	25	5	27683.293	13297_4_ – $40980_{3}^{{}^{\circ}}$
3611.158	200s	10	27684.052	2869_3_ – $30553_{2}^{{}^{\circ}}$
3610.650	300	20	27687.947	13088_3_ – $40776_{4}^{{}^{\circ}}$
3608.880	300	4	27701.526	8111_4_ – $35812_{3}^{{}^{\circ}}$
3607.819	75		27709.673	7280_2_ – $34989_{1}^{{}^{\circ}}$
3606.090	200	3	27722.958	2558_0_ – $30281_{1}^{{}^{\circ}}$
3604.965	20		27731.609	$10414_{4}^{{}^{\circ}}$ – 38145_3_
3604.680	600r	40	27733.802	3865_1_ – $31599_{2}^{{}^{\circ}}$
3604.657	100		27733.979	5563_1_ – $33297_{2}^{{}^{\circ}}$
3604.064	200b		27738.542	7502_3_ – $35240_{3}^{{}^{\circ}}$
3603.694	150	4	27741.390	$13945_{3}^{{}^{\circ}}$ – 41686_3_
3601.770	200	10	27756.208	$11197_{0}^{{}^{\circ}}$ – 38953_5_
3601.295	300	10	27759.869	$11197_{0}^{{}^{\circ}}$ – 38956_4_
3600.431	300	50	27766.531	9804_5_ – $37571_{0}^{{}^{\circ}}$
3600.182	100	25	27768.451	$11241_{3}^{{}^{\circ}}$ – 39010_3_
3599.723	300	20	27771.992	$10414_{4}^{{}^{\circ}}$ – 38186_4_
3598.523	150	10	27781.252	$7795_{4}^{{}^{\circ}}$ – 35576_3_
3598.245	75	10	27783.399	$14243_{1}^{{}^{\circ}}$ – 42027_2_
3598.120	2000r	50	27784.364	0_2_ – $27784_{2}^{{}^{\circ}}$
3597.776	10		27787.020	$7795_{4}^{{}^{\circ}}$ – 35582_5_
3597.021	75	2	27792.853	4961_4_ – $32754_{3}^{{}^{\circ}}$
3595.975	150	5	27800.937	9804_5_ – $37605_{4}^{{}^{\circ}}$
3595.756	100	10	27802.630	$11877_{1}^{{}^{\circ}}$ – 39680_2_
3595.617	500r	100	27803.705	$8243_{2}^{{}^{\circ}}$ – 36047_2_
3594.985	300	20	27808.593	11802_2_ – $39611_{3}^{{}^{\circ}}$
3593.535	15		27819.813	$10783_{2}^{{}^{\circ}}$ – 38602_2_
3593.352	5		27821.230	$14465_{2}^{{}^{\circ}}$ – 42286_3_
3592.777	800r	100	27825.682	8800_4_ – $36625_{0}^{{}^{\circ}}$
3591.452	800r	50	27835.948	3687_2_ – $31523_{3}^{{}^{\circ}}$
3591.253	300	50	27837.490	13297_4_ – $41134_{4}^{{}^{\circ}}$
3590.924	400		27840.041	$10526_{3}^{{}^{\circ}}$ – 38366_4_
3589.992	300	20	27847.268	3865_1_ – $31712_{1}^{{}^{\circ}}$
3589.748	1000r	75	27849.161	7502_3_ – $35351_{4}^{{}^{\circ}}$
3586.703	100		27872.803	13847_2_ – $41720_{2}^{{}^{\circ}}$
3584.175	800r	50	27892.462	2869_3_ – $30761_{3}^{{}^{\circ}}$
3583.100	600r	25	27900.830	4961_4_ – $32862_{4}^{{}^{\circ}}$
3581.754	200	10	27911.315	3687_2_ – $31599_{2}^{{}^{\circ}}$
3579.543	300	50	27928.554	12847_3_ – $40776_{4}^{{}^{\circ}}$
3578.947	400	10	27933.205	$11241_{3}^{{}^{\circ}}$ – 39174_3_
3577.596	200	10	27943.753	2869_3_ – $30812_{2}^{{}^{\circ}}$
3576.633	300	40	27951.277	$10783_{2}^{{}^{\circ}}$ – 38734_3_
3576.557	400r	150	27951.871	8111_4_ – $36062_{4}^{{}^{\circ}}$
3576.481	300		27952.465	$10414_{4}^{{}^{\circ}}$ – 38366_4_
3575.546	50		27959.774	7502_3_ – $35462_{3}^{{}^{\circ}}$
3575.427	100	10	27960.705	7280_2_ – $35240_{3}^{{}^{\circ}}$
3575.301	50b		27961.690	6362_2_ – $34324_{2}^{{}^{\circ}}$
3575.121	500r	50	27963.098	8111_4_ – $36074_{3}^{{}^{\circ}}$
3571.008	300	10	27995.304	$14032_{2}^{{}^{\circ}}$ – 42027_2_
3570.523	300	10	27999.107	$7795_{4}^{{}^{\circ}}$ – 35794_4_
3570.357	500	10	28000.408	8800_4_ – $36800_{4}^{{}^{\circ}}$
3569.977	100	5	28003.389	$10526_{3}^{{}^{\circ}}$ – 38529_3_
3569.820	1000r	25	28004.620	3865_1_ – $31870_{2}^{{}^{\circ}}$
3569.207	75		28009.430	6362_2_ – $34371_{2}^{{}^{\circ}}$
3567.672	200	20	28021.481	8111_4_ – $36132_{0}^{{}^{\circ}}$
3567.264	1000r	50	28024.686	0_2_ – $28024_{1}^{{}^{\circ}}$
3566.459	20	1	28031.011	7502_3_ – $35533_{2}^{{}^{\circ}}$
3565.822	300	15	28036.018	$7795_{4}^{{}^{\circ}}$ – 35831_3_
3565.604	200	10	28037.732	8800_4_ – $36837_{0}^{{}^{\circ}}$
3565.049	40		28042.097	$11241_{3}^{{}^{\circ}}$ – 39283_4_
3564.508	100	10	28046.353	13088_3_ – $41134_{4}^{{}^{\circ}}$
3564.235	200	8	28048.501	7280_2_ – $35328_{1}^{{}^{\circ}}$
3563.375	600r	75b	28055.270	$11197_{0}^{{}^{\circ}}$ – 39252_5_
3563.299	300	25	28055.869	$13175_{4}^{{}^{\circ}}$ – 41230_4_
3561.879	40		28067.053	8111_4_ – $36178_{4}^{{}^{\circ}}$
3561.780	500r	75	28067.833	6362_2_ – $34430_{3}^{{}^{\circ}}$
3560.688	200	5	28076.441	$10526_{3}^{{}^{\circ}}$ – 38602_2_
3560.295	20		28079.540	$14206_{4}^{{}^{\circ}}$ – 42286_3_
3560.226	40	5	28080.084	$11241_{3}^{{}^{\circ}}$ – 39321_3_
3560.021	300r	50	28081.701	4961_4_ – $33043_{3}^{{}^{\circ}}$
3559.378	200b		28086.774	$11197_{0}^{{}^{\circ}}$ – 39283_4_
3558.638	100		28092.614	$8243_{2}^{{}^{\circ}}$ – 36336_3_
3558.599	8		28092.922	3687_2_ – $31780_{3}^{{}^{\circ}}$
3557.818	150	5	28099.089	5563_1_ – $33662_{1}^{{}^{\circ}}$
3556.206	8		28111.826	13088_3_ – $41200_{2}^{{}^{\circ}}$
3555.705	500r	50	28115.787	$10414_{4}^{{}^{\circ}}$ – 38529_3_
3555.013	1000r	50	28121.259	2869_3_ – $30990_{3}^{{}^{\circ}}$
3551.984	20		28145.239	9804_5_ – $37950_{4}^{{}^{\circ}}$
3551.401	1000r	150	28149.860	2869_3_ – $31019_{4}^{{}^{\circ}}$
3550.878	300	40	28154.006	$14481_{6}^{{}^{\circ}}$ – 42635_5_
3550.783	300	40	28154.759	$11197_{0}^{{}^{\circ}}$ – 39351_5_
3550.718	100s		28155.274	5563_1_ – $33718_{2}^{{}^{\circ}}$
3549.596	1000r	75	28164.174	8111_4_ – $36275_{0}^{{}^{\circ}}$
3549.396	50	25	28165.761	2558_0_ – $30723_{1}^{{}^{\circ}}$
3548.664	300	8	28171.570	5563_1_ – $33734_{2}^{{}^{\circ}}$
3547.469	1501		28181.060	$13175_{4}^{{}^{\circ}}$ – 41356_5_
3547.364	300b		28181.894	7280_2_ – $35462_{3}^{{}^{\circ}}$
3547.338	200b		28182.100	3687_2_ – $31870_{2}^{{}^{\circ}}$
3547.252	100	5	28182.784	11802_2_ – $39985_{3}^{{}^{\circ}}$
3546.809	75		28186.304	$13175_{4}^{{}^{\circ}}$ – 41361_3_
3544.250	150	3	28206.654	2869_3_ – $31075_{2}^{{}^{\circ}}$
3544.097	200		28207.872	$10526_{3}^{{}^{\circ}}$ – 38734_3_
3544.018	800r	50	28208.500	8800_4_ – $37008_{0}^{{}^{\circ}}$
3543.208	150	3	28214.949	3865_1_ – $32080_{1}^{{}^{\circ}}$
3542.498	500r	50	28220.604	6362_2_ – $34583_{3}^{{}^{\circ}}$
3541.692	40		28227.026	$10783_{2}^{{}^{\circ}}$ – 39010_3_
3541.493	150		28228.612	6362_2_ – $34591_{1}^{{}^{\circ}}$
3540.807	2		28234.081	$10526_{3}^{{}^{\circ}}$ – 38760_4_
3539.951	8b		28240.908	8800_4_ – $37041_{4}^{{}^{\circ}}$
3539.843	300	25	28241.770	$8243_{2}^{{}^{\circ}}$ – 36485_2_
3538.415	3		28253.167	7280_2_ – $35533_{2}^{{}^{\circ}}$
3538.264	15	2	28254.372	$14032_{2}^{{}^{\circ}}$ – 42286_3_
3536.009	500r	20	28272.390	$8243_{2}^{{}^{\circ}}$ – 36515_3_
3535.250	150b		28278.460	$11241_{3}^{{}^{\circ}}$ – 39520_4_
3534.916	15		28281.132	$10526_{3}^{{}^{\circ}}$ – 38807_2_
3533.396	40	1	28293.298	11802_2_ – $40096_{2}^{{}^{\circ}}$
3533.181	200b	10	28295.019	3865_1_ – $32160_{2}^{{}^{\circ}}$
3531.450	1000r	50	28308.888	4961_4_ – $33270_{4}^{{}^{\circ}}$
3531.282	200	8	28310.235	7502_3_ – $35812_{3}^{{}^{\circ}}$
3530.514	800r	50	28316.393	8111_4_ – $36427_{3}^{{}^{\circ}}$
3530.030	50	3	28320.276	$10414_{4}^{{}^{\circ}}$ – 38734_3_
3529.670	200b	15	28323.164	$11197_{0}^{{}^{\circ}}$ – 39520_4_
3529.385	3001	40	28325.451	2869_3_ – $31194_{4}^{{}^{\circ}}$
3528.411	300	50	28333.270	4961_4_ – $33294_{3}^{{}^{\circ}}$
3527.793	200	10	28338.233	$7795_{4}^{{}^{\circ}}$ – 36133_5_
3527.434	4s		28341.117	$13945_{3}^{{}^{\circ}}$ – 42286_3_
3527.322	200	50	28342.017	6362_2_ – $34704_{3}^{{}^{\circ}}$
3526.764	300		28346.501	$10414_{4}^{{}^{\circ}}$ – 38760_4_
3526.634	500r	10	28347.546	0_2_ – $28347_{2}^{{}^{\circ}}$
3526.029	151	1	28352.410	12847_3_ – $41200_{2}^{{}^{\circ}}$
3525.171	8		28359.310	8800_4_ – $37159_{3}^{{}^{\circ}}$
3524.708	300	8	28363.035	6362_2_ – $34725_{3}^{{}^{\circ}}$
3524.178	300	25	28367.301	$15490_{0}^{{}^{\circ}}$ – 43857_5_
3523.758	300	8	28370.682	2558_0_ – $30928_{1}^{{}^{\circ}}$
3523.505	300		28372.719	0_2_ – $28372_{1}^{{}^{\circ}}$
3522.154	100	2	28383.602	7502_3_ – $35885_{2}^{{}^{\circ}}$
3521.141	100	2	28391.767	$10783_{2}^{{}^{\circ}}$ – 39174_3_
3521.059	500r	25	28392.428	3687_2_ – $32080_{1}^{{}^{\circ}}$
3520.453	5		28397.315	14226_0_ – $42624_{1}^{{}^{\circ}}$
3520.277	15		28398.735	7280_2_ – $35678_{1}^{{}^{\circ}}$
3518.885	4001	50	28409.969	$8243_{2}^{{}^{\circ}}$ – 36653_2_
3518.404	1000r	40	28413.853	2869_3_ – $31283_{3}^{{}^{\circ}}$
3518.197	100	5	28415.524	$7795_{4}^{{}^{\circ}}$ – 36210_5_
3516.530	3		28428.994	$14206_{4}^{{}^{\circ}}$ – 42635_5_
3515.327	100	40	28438.723	$11241_{3}^{{}^{\circ}}$ – 39680_2_
3514.285	200	10	28447.155	$15490_{0}^{{}^{\circ}}$ – 43937_6_
3513.682	75s	4	28452.037	11802_2_ – $40254_{3}^{{}^{\circ}}$
3511.157	800r	25	28472.497	3687_2_ – $32160_{2}^{{}^{\circ}}$
3509.784	10		28483.635	$10526_{3}^{{}^{\circ}}$ – 39010_3_
3509.089	500	25	28489.276	6362_2_ – $34851_{2}^{{}^{\circ}}$
3508.360	300	3	28495.196	11601_1_ – $40096_{2}^{{}^{\circ}}$
3508.100	300b		28497.307	$11197_{0}^{{}^{\circ}}$ – 39694_5_
3507.520	100		28502.020	$7795_{4}^{{}^{\circ}}$ – 36297_4_
3506.645	300	5	28509.131	3687_2_ – $32197_{3}^{{}^{\circ}}$
3506.345	150	10	28511.571	$13175_{4}^{{}^{\circ}}$ – 41686_3_
3506.132	300	3	28513.303	0_2_ – $28513_{2}^{{}^{\circ}}$
3505.934	10		28514.913	8111_4_ – $36625_{0}^{{}^{\circ}}$
3505.767	150	5	28516.271	6362_2_ – $34878_{1}^{{}^{\circ}}$
3503.863	75		28531.766	$8243_{2}^{{}^{\circ}}$ – 36775_3_
3503.786	500r	40	28532.393	7280_2_ – $35812_{3}^{{}^{\circ}}$
3503.130	200	15	28537.736	$13175_{4}^{{}^{\circ}}$ – 41712_5_
3503.017	100	5	28538.657	$10783_{2}^{{}^{\circ}}$ – 39321_3_
3502.963	200	10	28539.097	$10414_{4}^{{}^{\circ}}$ – 38953_5_
3502.739	200b		28540.922	$7795_{4}^{{}^{\circ}}$ – 36336_3_
3502.514	100	2	28542.755	$10414_{4}^{{}^{\circ}}$ – 38956_4_
3501.866	150b	25	28548.037	5563_1_ – $34111_{1}^{{}^{\circ}}$
3500.326	50	10	28560.596	7502_3_ – $36062_{4}^{{}^{\circ}}$
3499.821	200	5	28564.717	$8243_{2}^{{}^{\circ}}$ – 36808_3_
3498.956	200b	20b	28571.779	7502_3_ – $36074_{3}^{{}^{\circ}}$
3498.621	800r	75	28574.514	$8243_{2}^{{}^{\circ}}$ – 36818_2_
3497.901	50	10	28580.396	9804_5_ – $38385_{0}^{{}^{\circ}}$
3497.856	50	3	28580.764	$13175_{4}^{{}^{\circ}}$ – 41755_4_
3496.811	500r	15	28589.305	0_2_ – $28589_{3}^{{}^{\circ}}$
3496.003	200	10	28595.912	$11241_{3}^{{}^{\circ}}$ – 39837_3_
3495.847	15		28597.188	3687_2_ – $32285_{3}^{{}^{\circ}}$
3495.700	800r	25	28598.390	4961_4_ – $33560_{4}^{{}^{\circ}}$
3494.801	100		28605.747	7280_2_ – $35885_{2}^{{}^{\circ}}$
3492.158	25		28627.396	6362_2_ – $34989_{1}^{{}^{\circ}}$
3491.900	500	2	28629.511	4961_4_ – $33591_{3}^{{}^{\circ}}$
3490.848	20	1	28638.139	3865_1_ – $32503_{2}^{{}^{\circ}}$
3489.602	1001	1	28648.364	$10526_{3}^{{}^{\circ}}$ – 39174_3_
3489.509	400	2	28649.128	0_2_ – $28649_{1}^{{}^{\circ}}$
3489.185	500	4	28651.788	8111_4_ – $36762_{3}^{{}^{\circ}}$
3488.834	500	3	28654.670	2869_3_ – $31523_{3}^{{}^{\circ}}$
3487.991	1501	4	28661.596	13962_1_ – $42624_{1}^{{}^{\circ}}$
3486.551	1000r	10b	28673.433	0_2_ – $28673_{2}^{{}^{\circ}}$
3486.269	50	1	28675.752	7502_3_ – $36178_{4}^{{}^{\circ}}$
3486.210	50	1	28676.238	0_2_ – $28676_{3}^{{}^{\circ}}$
3484.943	100	1	28686.663	7502_3_ – $36188_{2}^{{}^{\circ}}$
3484.580	8		28689.651	8111_4_ – $36800_{4}^{{}^{\circ}}$
3480.810	100		28720.723	$7795_{4}^{{}^{\circ}}$ – 36515_3_
3480.052	500	3	28726.979	8111_4_ – $36837_{0}^{{}^{\circ}}$
3479.684	300	2	28730.017	2869_3_ – $31599_{2}^{{}^{\circ}}$
3479.221	300b	2	28733.840	$11877_{1}^{{}^{\circ}}$ – 40611_2_
3476.385	200	3	28757.280	$10526_{3}^{{}^{\circ}}$ – 39283_4_
3475.961	100b		28760.788	$10414_{4}^{{}^{\circ}}$ – 39174_3_
3475.943	100b		28760.937	5563_1_ – $34324_{2}^{{}^{\circ}}$
3474.717	200	10	28771.084	8800_4_ – $37571_{0}^{{}^{\circ}}$
3474.530	200	3	28772.633	$12114_{2}^{{}^{\circ}}$ – 40886_2_
3474.188	3		28775.465	9804_5_ – $38580_{4}^{{}^{\circ}}$
3472.989	15b		28785.399	3865_1_ – $32650_{1}^{{}^{\circ}}$
3471.959	500r	501	28793.938	7280_2_ – $36074_{3}^{{}^{\circ}}$
3471.800	50b		28795.257	$10526_{3}^{{}^{\circ}}$ – 39321_3_
3471.216	1000r	50b	28800.102	3865_1_ – $32665_{1}^{{}^{\circ}}$
3471.001	300	15	28801.885	2869_3_ – $31671_{4}^{{}^{\circ}}$
3470.568	300	20	28805.479	8800_4_ – $37605_{4}^{{}^{\circ}}$
3470.181	5	2	28808.691	5563_1_ – $34371_{2}^{{}^{\circ}}$
3469.345	1000r	150	28815.633	3687_2_ – $32503_{2}^{{}^{\circ}}$
3466.644	600r	100	28838.083	4961_4_ – $33799_{4}^{{}^{\circ}}$
3466.538	600r	40	28838.965	4961_4_ – $33800_{3}^{{}^{\circ}}$
3465.061	200b	25	28851.258	$8243_{2}^{{}^{\circ}}$ – 37094_2_
3464.560	25	2	28855.430	$11197_{0}^{{}^{\circ}}$ – 40052_4_
3463.921	50	2	28860.753	9804_5_ – $38665_{0}^{{}^{\circ}}$
3461.801	8		28878.426	6362_2_ – $35240_{3}^{{}^{\circ}}$
3461.574	200	3	28880.320	7502_3_ – $36382_{4}^{{}^{\circ}}$
3461.215	2000r	75	28883.315	4961_4_ – $33844_{0}^{{}^{\circ}}$
3461.018	1500r	50	28884.959	0_2_ – $28884_{3}^{{}^{\circ}}$
3460.724	75	2	28887.413	3687_2_ – $32575_{2}^{{}^{\circ}}$
3460.724	75	2	28887.413	$8243_{2}^{{}^{\circ}}$ – 37131_3_
3459.536	50b		28897.333	$10783_{2}^{{}^{\circ}}$ – 39680_2_
3459.488	75	8	28897.734	8111_4_ – $37008_{0}^{{}^{\circ}}$
3458.299	20		28907.669	$10414_{4}^{{}^{\circ}}$ – 39321_3_
3457.829	75		28911.598	2869_3_ – $31780_{3}^{{}^{\circ}}$
3457.069	400r	25	28917.954	0_2_ – $28917_{2}^{{}^{\circ}}$
3456.217	2		28925.082	7502_3_ – $36427_{3}^{{}^{\circ}}$
3455.612	200b	10	28930.146	8111_4_ – $37041_{4}^{{}^{\circ}}$
3453.513	300b	10	28947.729	$15490_{0}^{{}^{\circ}}$ – 44437_6_
3452.207	75	1	28958.680	$11241_{3}^{{}^{\circ}}$ – 40200_3_
3451.702	800r	100	28962.916	3687_2_ – $32650_{1}^{{}^{\circ}}$
3451.308	100		28966.223	6362_2_ – $35328_{1}^{{}^{\circ}}$
3449.949	200	5	28977.623	3687_2_ – 32665_1_
3448.949	300	10	28986.034	$7795_{4}^{{}^{\circ}}$ – 36781_5_
3448.891	15h		28986.522	7502_3_ – $36488_{2}^{{}^{\circ}}$
3447.189	40		29000.833	2869_3_ – $31870_{2}^{{}^{\circ}}$
3446.546	300	8	29006.243	4961_4_ – $33967_{3}^{{}^{\circ}}$
3446.200	15		29009.155	$11877_{1}^{{}^{\circ}}$ – 40886_2_
3443.980	100	8	29027.854	5563_1_ – $34591_{1}^{{}^{\circ}}$
3442.578	800r	20	29039.676	4961_4_ – $34001_{4}^{{}^{\circ}}$
3441.527	200	5	29048.544	8111_4_ – $37159_{3}^{{}^{\circ}}$
3440.827	8		29054.453	$10783_{2}^{{}^{\circ}}$ – 39837_3_
3439.748	100b		29063.567	$8243_{2}^{{}^{\circ}}$ – 37307_3_
3439.399	400b	8	29066.516	3687_2_ – $32754_{3}^{{}^{\circ}}$
3437.645	50b	5b	29081.346	7280_2_ – $36361_{1}^{{}^{\circ}}$
3437.307	2000r	75	29084.206	2869_3_ – $31953_{4}^{{}^{\circ}}$
3436.726	1500r	50	29089.122	3865_1_ – $32954_{2}^{{}^{\circ}}$
3436.621	200	4	29090.011	$7795_{4}^{{}^{\circ}}$ – 36885_4_
3435.487	251		29099.613	6362_2_ – $35462_{3}^{{}^{\circ}}$
3434.727	200b	2	29106.052	$10414_{4}^{{}^{\circ}}$ – 39520_4_
3434.103	50b		29111.340	$13175_{4}^{{}^{\circ}}$ – 42286_3_
3429.871	100b		29147.259	7280_2_ – $36427_{3}^{{}^{\circ}}$
3429.574	200	10	29149.783	8800_4_ – $37950_{4}^{{}^{\circ}}$
3429.086	50		29153.931	$10526_{3}^{{}^{\circ}}$ – 39680_2_
3428.998	1500r	50	29154.679	2558_0_ – $31712_{1}^{{}^{\circ}}$
3428.714	300r	8	29157.094	0_2_ – $29157_{1}^{{}^{\circ}}$
3428.621	300r	4	29157.885	0_2_ – $29157_{3}^{{}^{\circ}}$
3428.248	50	3	29161.057	$8243_{2}^{{}^{\circ}}$ – 37404_3_
3427.619	5		29166.408	7502_3_ – $36668_{2}^{{}^{\circ}}$
3427.089	200s		29170.919	6362_2_ – $35533_{2}^{{}^{\circ}}$
3426.285	2		29177.764	11802_2_ – $40980_{3}^{{}^{\circ}}$
3425.435	150	15	29185.004	$11241_{3}^{{}^{\circ}}$ – 40426_4_
3423.990	1500r	50	29197.320	0_2_ – $29197_{1}^{{}^{\circ}}$
3423.923	200b		29197.891	0_2_ – $29197_{2}^{{}^{\circ}}$
3422.655	1000r	40	29208.708	7280_2_ – $36488_{2}^{{}^{\circ}}$
3421.210	2000r		29221.044	4961_4_ – $34182_{0}^{{}^{\circ}}$
3420.196	100	3	29229.707	$11197_{0}^{{}^{\circ}}$ – 40426_4_
3418.170	15		29247.032	$12114_{2}^{{}^{\circ}}$ – 41361_3_
3417.642	100		29251.550	$11241_{3}^{{}^{\circ}}$ – 40493_3_
3417.498	400r	10	29252.783	0_2_ – $29252_{2}^{{}^{\circ}}$
3416.595	150	3	29260.514	7502_3_ – $36762_{3}^{{}^{\circ}}$
3415.885	500r	75	29266.595	3687_2_ – $32954_{2}^{{}^{\circ}}$
3414.712	200	2	29276.649	7280_2_ – $36556_{1}^{{}^{\circ}}$
3414.565	100b	1	29277.909	$8243_{2}^{{}^{\circ}}$ – 37521_3_
3414.299	75		29280.190	$10414_{4}^{{}^{\circ}}$ – 39694_5_
3413.327	100	3	29288.528	5563_1_ – $34851_{2}^{{}^{\circ}}$
3413.086	300b	25	29290.596	$7795_{4}^{{}^{\circ}}$ – 37085_4_
3413.013	1000r	25	29291.222	2869_3_ – $32160_{2}^{{}^{\circ}}$
3412.422	15		29296.295	3865_1_ – $33161_{1}^{{}^{\circ}}$
3412.181	200		29298.364	7502_3_ – $36800_{4}^{{}^{\circ}}$
3410.699	25		29311.094	$10526_{3}^{{}^{\circ}}$ – 39837_3_
3410.184	150	3	29315.521	5563_1_ – $34878_{1}^{{}^{\circ}}$
3410.076	300	50	29316.449	6362_2_ – $35678_{1}^{{}^{\circ}}$
3408.750	1000r	25	29327.853	2869_3_ – $32197_{3}^{{}^{\circ}}$
3405.558	1500r	50	29355.341	3687_2_ – $33043_{3}^{{}^{\circ}}$
3403.903	300	8	29369.613	7502_3_ – $36871_{2}^{{}^{\circ}}$
3403.222	15		29375.490	$14481_{6}^{{}^{\circ}}$ – 43857_5_
3402.354	15		29382.984	4961_4_ – $34344_{4}^{{}^{\circ}}$
3401.711	500r	25	29388.538	7280_2_ – $36668_{2}^{{}^{\circ}}$
3400.680	25		29397.447	11802_2_ – $41200_{2}^{{}^{\circ}}$
3398.545	1500r	50	29415.914	2869_3_ – $32285_{3}^{{}^{\circ}}$
3398.490	150b	4	29416.390	$7795_{4}^{{}^{\circ}}$ – 37211_5_
3398.457	100b	4	29416.676	8800_4_ – $38216_{3}^{{}^{\circ}}$
3398.162	25		29419.230	0_2_ – $29419_{2}^{{}^{\circ}}$
3397.516	1500r	50b	29424.823	2869_3_ – $32294_{4}^{{}^{\circ}}$
3397.306	50	1	29426.642	5563_1_ – $34989_{1}^{{}^{\circ}}$
3396.727	800r	50	29431.658	3865_1_ – $33297_{2}^{{}^{\circ}}$
3395.234	20	1	29444.600	$8243_{2}^{{}^{\circ}}$ – 37688_3_
3393.993	200	2	29455.365	$14481_{6}^{{}^{\circ}}$ – 43937_6_
3393.421	150	10	29460.331	8111_4_ – $37571_{0}^{{}^{\circ}}$
3393.373	4b		29460.747	$13175_{4}^{{}^{\circ}}$ – 42635_5_
3392.993	25		29464.046	$12114_{2}^{{}^{\circ}}$ – 41578_2_
3392.473	100	2	29468.563	4961_4_ – $34430_{3}^{{}^{\circ}}$
3391.872	400r	40	29473.784	3687_2_ – $33161_{1}^{{}^{\circ}}$
3390.849	200	8	29482.676	7280_2_ – $36762_{3}^{{}^{\circ}}$
3389.463	800r	40	29494.731	8111_4_ – $37605_{4}^{{}^{\circ}}$
3388.348	50		29504.437	$8243_{2}^{{}^{\circ}}$ – 37748_2_
3388.154	150b	5	29506.126	8800_4_ – $38306_{4}^{{}^{\circ}}$
3388.117	40b	2	29506.448	9804_5_ – $39311_{4}^{{}^{\circ}}$
3387.920	600r	15	29508.164	$7795_{4}^{{}^{\circ}}$ – 37303_4_
3387.493	50b		29511.883	$7795_{4}^{{}^{\circ}}$ – 37307_3_
3386.291	5		29522.359	2558_0_ – $32080_{1}^{{}^{\circ}}$
3386.159	15	5	29523.509	6362_2_ – $35885_{2}^{{}^{\circ}}$
3385.373	4		29530.364	4961_4_ – $34492_{4}^{{}^{\circ}}$
3384.403	200	4	29538.827	7502_3_ – $37041_{4}^{{}^{\circ}}$
3384.177	15		29540.800	$8243_{2}^{{}^{\circ}}$ – 37784_2_
3383.586	200	8	29545.959	8111_4_ – $37656_{3}^{{}^{\circ}}$
3382.310	4		29557.106	7280_2_ – $36837_{1}^{{}^{\circ}}$
3381.800	2		29561.563	3865_1_ – $33427_{2}^{{}^{\circ}}$
3380.859	600r	20	29569.790	2869_3_ – $32439_{4}^{{}^{\circ}}$
3380.571	3		29572.309	$12114_{2}^{{}^{\circ}}$ – 41686_3_
3379.124	10b		29584.973	8800_4_ – $38385_{0}^{{}^{\circ}}$
3378.348	2		29591.768	7280_2_ – $36871_{2}^{{}^{\circ}}$
3377.483	100	15	29599.346	11601_1_ – $41200_{2}^{{}^{\circ}}$
3376.617	25		29606.937	3687_2_ – $33294_{3}^{{}^{\circ}}$
3376.367	300	1	29609.130	3687_2_ – $33297_{2}^{{}^{\circ}}$
3375.587	200b		29615.971	8800_4_ – $38416_{3}^{{}^{\circ}}$
3374.975	1500r	75	29621.341	4961_4_ – $34583_{3}^{{}^{\circ}}$
3373.493	800r	75	29634.354	2869_3_ – $32503_{2}^{{}^{\circ}}$
3372.822	400r	100	29640.249	0_2_ – $29640_{1}^{{}^{\circ}}$
3372.253	10		29645.250	$11241_{3}^{{}^{\circ}}$ – 40886_2_
3372.077	8		29646.798	7502_3_ – $37149_{2}^{{}^{\circ}}$
3371.664	10		29650.429	$14206_{4}^{{}^{\circ}}$ – 43857_5_
3370.887	200	5	29657.263	7502_3_ – $37159_{3}^{{}^{\circ}}$
3369.000	200	4	29673.874	$10526_{3}^{{}^{\circ}}$ – 40200_3_
3367.582	400r	10	29686.368	0_2_ – $29686_{3}^{{}^{\circ}}$
3365.337	400r	50	29706.171	2869_3_ – $32575_{2}^{{}^{\circ}}$
3364.888	25		29710.135	$10783_{2}^{{}^{\circ}}$ – 40493_3_
3362.443	10b		29731.738	7502_3_ – $37234_{2}^{{}^{\circ}}$
3361.196	300r	25	29742.768	4961_4_ – $34704_{3}^{{}^{\circ}}$
3360.997	400r	150	29744.529	0_2_ – $29744_{3}^{{}^{\circ}}$
3356.986	8		29780.068	8800_4_ – $38580_{4}^{{}^{\circ}}$
3355.106	500r	10	29796.754	3865_1_ – $33662_{1}^{{}^{\circ}}$
3351.753	3		29826.561	6362_2_ – $36188_{2}^{{}^{\circ}}$
3350.352	200	5	29839.033	8111_4_ – $37950_{4}^{{}^{\circ}}$
3349.800	40b		29843.950	8111_4_ – $37954_{3}^{{}^{\circ}}$
3348.790	400b		29852.950	3865_1_ – $33718_{2}^{{}^{\circ}}$
3348.770	1500r	20	29853.129	0_2_ – $29853_{2}^{{}^{\circ}}$
3347.403	150		29865.319	8800_4_ – $38665_{0}^{{}^{\circ}}$
3346.992	100	3	29868.987	7280_2_ – $37149_{2}^{{}^{\circ}}$
3346.966	300	3	29869.219	3865_1_ – $33734_{2}^{{}^{\circ}}$
3345.851	200r	8b	29879.172	7280_2_ – $37159_{1}^{{}^{\circ}}$
3345.822	200r	8	29879.431	7280_2_ – $37159_{3}^{{}^{\circ}}$
3345.170	150	3	29885.255	2869_3_ – $32754_{3}^{{}^{\circ}}$
3344.310	20	2	29892.940	$7795_{4}^{{}^{\circ}}$ – 37688_3_
3343.499	150	5	29900.190	$10526_{3}^{{}^{\circ}}$ – 40426_4_
3343.281	100b		29902.140	$8243_{2}^{{}^{\circ}}$ – 38145_3_
3343.164	300	5	29903.186	3687_2_ – $33591_{3}^{{}^{\circ}}$
3342.069	200	10	29912.984	$12114_{2}^{{}^{\circ}}$ – 42027_2_
3341.550	10	2	29917.630	11802_2_ – $41720_{2}^{{}^{\circ}}$
3340.724	300r	5	29925.026	4961_4_ – $34886_{0}^{{}^{\circ}}$
3337.505	10		29953.888	7280_2_ – $37234_{2}^{{}^{\circ}}$
3337.275	200	5	29955.952	$14481_{6}^{{}^{\circ}}$ – 44437_6_
3336.074	150		29966.736	$10526_{3}^{{}^{\circ}}$ – 40493_3_
3335.694	150	4	29970.150	5563_1_ – $35533_{2}^{{}^{\circ}}$
3335.238	300	5	29974.247	3687_2_ – $33662_{1}^{{}^{\circ}}$
3333.571	10	1	29989.236	$11241_{3}^{{}^{\circ}}$ – 41230_4_
3333.128	1000r	20b	29993.222	2869_3_ – $32862_{4}^{{}^{\circ}}$
3332.479	100b	1	29999.062	6362_2_ – $36361_{1}^{{}^{\circ}}$
3330.976	150	4	30012.598	$10414_{4}^{{}^{\circ}}$ – 40426_4_
3330.477	1500r	40	30017.095	0_2_ – $30017_{3}^{{}^{\circ}}$
3329.728	400r	75	30023.847	$7795_{4}^{{}^{\circ}}$ – 37819_4_
3328.997	10s		30030.439	3687_2_ – $33718_{2}^{{}^{\circ}}$
3328.608	4		30033.949	$11197_{0}^{{}^{\circ}}$ – 41230_4_
3327.193	800r	25	30046.721	3687_2_ – $33734_{2}^{{}^{\circ}}$
3322.945	200b		30085.131	$10526_{3}^{{}^{\circ}}$ – 40611_2_
3322.924	10		30085.321	2869_3_ – $32954_{2}^{{}^{\circ}}$
3322.093	400r	10	30092.847	2558_0_ – $32650_{1}^{{}^{\circ}}$
3321.835	5	1	30095.184	$7795_{4}^{{}^{\circ}}$ – 37890_5_
3321.574	300r	40	30097.549	$7795_{4}^{{}^{\circ}}$ – 37892_4_
3321.067	3		30102.143	9804_5_ – $39906_{4}^{{}^{\circ}}$
3320.879	20		30103.847	$10783_{2}^{{}^{\circ}}$ – 40886_2_
3320.476	400r	25	30107.501	2558_0_ – $32665_{1}^{{}^{\circ}}$
3319.910	400r	75b	30112.634	3687_2_ – $33800_{3}^{{}^{\circ}}$
3319.572	50		30115.700	5563_1_ – $35678_{1}^{{}^{\circ}}$
3319.149	25b		30119.538	11601_1_ – $41720_{2}^{{}^{\circ}}$
3319.133	300b	40	30119.683	$11241_{3}^{{}^{\circ}}$ – 41361_3_
3318.390	200	5	30126.426	6362_2_ – $36488_{2}^{{}^{\circ}}$
3315.848	200	2	30149.521	$11877_{1}^{{}^{\circ}}$ – 42027_2_
3314.793	400r		30159.117	$11197_{0}^{{}^{\circ}}$ – 41356_5_
3313.744	20b		30168.663	$10414_{4}^{{}^{\circ}}$ – 40582_5_
3313.647	500r		30169.546	4961_4_ – $35131_{4}^{{}^{\circ}}$
3313.367	25b		30172.096	$12114_{2}^{{}^{\circ}}$ – 42286_3_
3313.151	5	1	30174.063	2869_3_ – $33043_{3}^{{}^{\circ}}$
3313.073	3001	40	30174.773	$10526_{3}^{{}^{\circ}}$ – 40701_4_
3310.922	8	2	30194.376	6362_2_ – $36556_{1}^{{}^{\circ}}$
3310.814	25	4	30195.361	8111_4_ – $38306_{4}^{{}^{\circ}}$
3310.637	150d	15b	30196.975	$7795_{4}^{{}^{\circ}}$ – 37992_4_
3310.446	200	10b	30198.717	8800_4_ – $38998_{3}^{{}^{\circ}}$
3309.365	1000r	40	30208.582	0_2_ – $30208_{2}^{{}^{\circ}}$
3306.506	2b		30234.701	7502_3_ – $37736_{2}^{{}^{\circ}}$
3305.303	400r	40	30245.705	3865_1_ – $34111_{1}^{{}^{\circ}}$
3304.238	3000r	200	30255.453	0_2_ – $30255_{3}^{{}^{\circ}}$
3303.546	20		30261.791	8800_4_ – $39062_{0}^{{}^{\circ}}$
3302.192	400r	5	30274.198	8111_4_ – $38385_{0}^{{}^{\circ}}$
3301.651	1500r	150	30279.159	4961_4_ – $35240_{3}^{{}^{\circ}}$
3301.570	200	8	30279.902	3687_2_ – $33967_{3}^{{}^{\circ}}$
3301.449	150	3	30281.011	0_2_ – $30281_{1}^{{}^{\circ}}$
3300.870	20	4	30286.323	$8243_{2}^{{}^{\circ}}$ – 38529_3_
3300.775	150	4	30287.194	$10414_{4}^{{}^{\circ}}$ – 40701_4_
3298.811	300	8	30305.226	8111_4_ – $38416_{3}^{{}^{\circ}}$
3298.697	75	4	30306.273	6362_2_ – $36668_{2}^{{}^{\circ}}$
3298.119	75	3	30311.584	$7795_{4}^{{}^{\circ}}$ – 38106_5_
3298.050	500r	40	30312.218	4961_4_ – $35273_{0}^{{}^{\circ}}$
3296.901	30h	2	30322.782	5563_1_ – $35885_{2}^{{}^{\circ}}$
3295.444	25		30336.188	7280_2_ – $37616_{1}^{{}^{\circ}}$
3292.926	300	4	30359.384	$8243_{2}^{{}^{\circ}}$ – 38602_2_
3292.810	50b	4	30360.454	$10526_{3}^{{}^{\circ}}$ – 40886_2_
3291.037	401	1	30376.810	7280_2_ – $37656_{3}^{{}^{\circ}}$
3289.631	1501	10	30389.792	4961_4_ – $35351_{4}^{{}^{\circ}}$
3289.517	150	10	30390.845	$7795_{4}^{{}^{\circ}}$ – 38186_4_
3288.478	2		30400.447	6362_2_ – $36762_{3}^{{}^{\circ}}$
3288.387	300r	8	30401.288	2869_3_ – $33270_{4}^{{}^{\circ}}$
3286.019	20	3	30423.196	3687_2_ – $34111_{1}^{{}^{\circ}}$
3285.752	800r	20	30425.668	2869_3_ – $33294_{3}^{{}^{\circ}}$
3285.514	200r	5	30427.872	2869_3_ – $33297_{2}^{{}^{\circ}}$
3283.671	100	4	30444.949	$11241_{3}^{{}^{\circ}}$ – 41686_3_
3283.369	8	2	30447.749	7502_3_ – $37950_{4}^{{}^{\circ}}$
3282.837	40	2	30452.683	7502_3_ – $37954_{3}^{{}^{\circ}}$
3282.385	50	3	30456.877	7280_2_ – $37736_{2}^{{}^{\circ}}$
3282.198	8	1	30458.612	3865_1_ – $34324_{2}^{{}^{\circ}}$
3281.189	300	5	30467.978	7502_3_ – $37970_{2}^{{}^{\circ}}$
3281.049	400r	25	30469.278	8111_4_ – $38580_{4}^{{}^{\circ}}$
3280.451	20		30474.832	6362_2_ – $36837_{1}^{{}^{\circ}}$
3278.733	200	25	30490.800	$8243_{2}^{{}^{\circ}}$ – 38734_3_
3277.702	50	3	30500.390	4961_4_ – $35462_{3}^{{}^{\circ}}$
3277.062	200	4	30506.347	3865_1_ – $34371_{2}^{{}^{\circ}}$
3276.721	300	5	30509.521	6362_2_ – $36871_{2}^{{}^{\circ}}$
3276.562	25	3	30511.002	8800_4_ – $39311_{4}^{{}^{\circ}}$
3276.225	200	8	30514.140	$11241_{3}^{{}^{\circ}}$ – 41755_4_
3276.045	50	5	30515.817	$11197_{0}^{{}^{\circ}}$ – 41712_5_
3272.301	5		30550.730	7502_3_ – $38053_{3}^{{}^{\circ}}$
3272.027	800r	75	30553.288	0_2_ – $30553_{2}^{{}^{\circ}}$
3271.892	300r	100	30554.549	8111_4_ – $38665_{0}^{{}^{\circ}}$
3271.546	50	3	30557.780	2869_3_ – $33427_{2}^{{}^{\circ}}$
3271.433	2		30558.836	$11197_{0}^{{}^{\circ}}$ – 41755_4_
3270.874	150	10	30564.058	$8243_{2}^{{}^{\circ}}$ – 38807_2_
3270.100	100	5	30571.292	$7795_{4}^{{}^{\circ}}$ – 38366_4_
3269.352	3		30578.286	$10783_{2}^{{}^{\circ}}$ – 41361_3_
3268.453	300	75	30586.697	7502_3_ – $38088_{2}^{{}^{\circ}}$
3266.635	500r	50	30603.719	2558_0_ – $33161_{1}^{{}^{\circ}}$
3264.859	40	3	30620.366	9804_5_ – $40425_{4}^{{}^{\circ}}$
3263.183	400r	20	30636.092	3687_2_ – $34324_{2}^{{}^{\circ}}$
3259.744	25	2	30668.412	8800_4_ – $39468_{3}^{{}^{\circ}}$
3259.061	600r	50	30674.839	7280_2_ – $37954_{3}^{{}^{\circ}}$
3258.744	100	3	30677.823	8111_4_ – $38788_{3}^{{}^{\circ}}$
3258.274	15	2	30682.248	$13175_{4}^{{}^{\circ}}$ – 43857_5_
3258.103	400r		30683.858	3687_2_ – $34371_{2}^{{}^{\circ}}$
3257.435	200	75	30690.150	7280_2_ – $37970_{2}^{{}^{\circ}}$
3257.366	1000r	50	30690.800	2869_3_ – $33560_{4}^{{}^{\circ}}$
3255.920	200	8	30704.430	$10526_{3}^{{}^{\circ}}$ – 41230_4_
3254.834	8b		30714.674	7502_3_ – $38216_{3}^{{}^{\circ}}$
3254.067	300r	20	30721.914	2869_3_ – $33591_{3}^{{}^{\circ}}$
3253.866	300r	50	30723.811	0_2_ – $30723_{1}^{{}^{\circ}}$
3253.684	300r	40	30725.530	3865_1_ – $34591_{1}^{{}^{\circ}}$
3251.916	2000r	200	30742.234	3687_2_ – $34430_{3}^{{}^{\circ}}$
3249.855	800r	25	30761.730	0_2_ – $30761_{3}^{{}^{\circ}}$
3249.344	50	3	30766.567	$8243_{2}^{{}^{\circ}}$ – 39010_3_
3248.790	3		30771.813	4961_4_ – $35733_{4}^{{}^{\circ}}$
3247.331	15		30785.639	$11241_{3}^{{}^{\circ}}$ – 42027_2_
3247.218	75	4	30786.710	6362_2_ – $37149_{2}^{{}^{\circ}}$
3246.319	5	1	30795.235	$10783_{2}^{{}^{\circ}}$ – 41578_2_
3246.144	75	4	30796.895	6362_2_ – $37159_{1}^{{}^{\circ}}$
3246.116	20	2	30797.161	6362_2_ – $37159_{3}^{{}^{\circ}}$
3245.994	200	8	30798.319	5563_1_ – $36361_{1}^{{}^{\circ}}$
3245.386	3	1	30804.088	7502_3_ – $38306_{4}^{{}^{\circ}}$
3244.883	100	5	30808.863	7280_2_ – $38088_{2}^{{}^{\circ}}$
3244.628	4		30811.284	8800_4_ – $39611_{3}^{{}^{\circ}}$
3244.448	1000r	50	30812.994	0_2_ – $30812_{2}^{{}^{\circ}}$
3244.043	150	10	30816.840	$10414_{4}^{{}^{\circ}}$ –41230_4_
3243.585	40	2	30821.192	11802_2_ – $42624_{1}^{{}^{\circ}}$
3242.145	50	15b	30834.880	$10526_{3}^{{}^{\circ}}$ – 41361_3_
3240.644	400r	40b	30849.162	2869_3_ – $33718_{2}^{{}^{\circ}}$
3240.236	40	3	30853.046	7502_3_ – $38355_{2}^{{}^{\circ}}$
3238.934	400r	20	30865.448	2869_3_ – $33734_{2}^{{}^{\circ}}$
3238.287	25	3	30871.615	6362_2_ – $37234_{2}^{{}^{\circ}}$
3236.573	100	8b	30887.963	8111_4_ – $38998_{3}^{{}^{\circ}}$
3234.996	400r	20	30903.020	3687_2_ – $34591_{1}^{{}^{\circ}}$
3234.791	10	2	30904.978	$7795_{4}^{{}^{\circ}}$ – 38700_4_
3233.853	5	2	30913.942	7502_3_ – $38416_{3}^{{}^{\circ}}$
3233.240	15	2	30919.803	9804_5_ – $40724_{0}^{{}^{\circ}}$
3232.305	500r	25	30928.747	0_2_ – $30928_{1}^{{}^{\circ}}$
3232.124	300r		30930.479	2869_3_ – $33799_{4}^{{}^{\circ}}$
3232.031	200r		30931.368	2869_3_ – $33800_{3}^{{}^{\circ}}$
3231.461	75		30936.824	7280_2_ – $38216_{3}^{{}^{\circ}}$
3231.221	5		30939.122	$7795_{4}^{{}^{\circ}}$ – 38734_3_
3230.368	25	2	30947.291	$10414_{4}^{{}^{\circ}}$ – 41361_3_
3228.485	25	3	30965.341	$7795_{4}^{{}^{\circ}}$ – 38760_4_
3227.820	10		30971.720	9804_5_ – $40776_{4}^{{}^{\circ}}$
3225.863	50	8	30990.509	0_2_ – $30990_{3}^{{}^{\circ}}$
3225.539	100	8	30993.621	5563_1_ – $36556_{1}^{{}^{\circ}}$
3225.007	100	5	30998.734	7280_2_ – $38278_{1}^{{}^{\circ}}$
3223.504	8	2	31013.187	3865_1_ – $34878_{1}^{{}^{\circ}}$
3223.168	200r	5	31016.420	3687_2_ – $34704_{3}^{{}^{\circ}}$
3222.475	101		31023.090	11601_1_ – $42624_{1}^{{}^{\circ}}$
3220.986	75b		31037.431	3687_2_ – $34725_{3}^{{}^{\circ}}$
3219.490	20h	4	31051.852	$10526_{3}^{{}^{\circ}}$ – 41578_2_
3217.265	40	2	31073.326	8800_4_ – $39873_{0}^{{}^{\circ}}$
3216.998	75	3	31075.905	0_2_ – $31075_{2}^{{}^{\circ}}$
3216.761	40		31078.195	$8243_{2}^{{}^{\circ}}$ – 39321_3_
3214.648	150	5	31098.622	2869_3_ – $33967_{3}^{{}^{\circ}}$
3214.381	400r	25	31101.205	4961_4_ – $36062_{4}^{{}^{\circ}}$
3214.076	400r	50	31104.156	2558_0_ – $33662_{1}^{{}^{\circ}}$
3213.937	8	1	31105.501	5563_1_ – $36668_{2}^{{}^{\circ}}$
3213.818	3		31106.653	8800_4_ – $39906_{4}^{{}^{\circ}}$
3211.997	200r	5	31124.288	3865_1_ – $34989_{1}^{{}^{\circ}}$
3211.195	400r	25	31132.061	2869_3_ – $34001_{4}^{{}^{\circ}}$
3210.779	200	5	31136.095	7280_2_ – $38416_{3}^{{}^{\circ}}$
3208.971	15		31153.636	8111_4_ – $39264_{3}^{{}^{\circ}}$
3208.527	100	3	31157.947	$7795_{4}^{{}^{\circ}}$ – 38953_5_
3208.152	25		31161.589	$7795_{4}^{{}^{\circ}}$ – 38956_4_
3207.938	100	5	31163.668	3687_2_ – $34851_{2}^{{}^{\circ}}$
3207.548	4		31167.457	7502_3_ – $38669_{2}^{{}^{\circ}}$
3205.696	50	3	31185.462	8800_4_ – $39985_{3}^{{}^{\circ}}$
3205.162	3		31190.658	3687_2_ – $34878_{1}^{{}^{\circ}}$
3202.521	200r	8	31216.379	4961_4_ – $36178_{4}^{{}^{\circ}}$
3199.669	1		31244.202	$10783_{2}^{{}^{\circ}}$ – 42027_2_
3196.611	20	2	31274.091	5563_1_ – $36837_{1}^{{}^{\circ}}$
3195.688	400r	50	31283.123	0_2_ – $31283_{3}^{{}^{\circ}}$
3194.518	5		31294.580	6362_2_ – $37656_{3}^{{}^{\circ}}$
3194.096	200	5	31298.715	$10414_{4}^{{}^{\circ}}$ – 41712_5_
3193.781	200r	5	31301.802	3687_2_ – $34989_{1}^{{}^{\circ}}$
3192.586	400r	20	31313.518	4961_4_ – $36275_{0}^{{}^{\circ}}$
3190.895	150b	2	31330.112	9804_5_ – $41134_{4}^{{}^{\circ}}$
3189.711	200	8	31341.741	$10414_{4}^{{}^{\circ}}$ – 41755_4_
3188.092	8b		31357.656	8111_4_ – $39468_{3}^{{}^{\circ}}$
3186.371	75	2	31374.592	6362_2_ – $37736_{2}^{{}^{\circ}}$
3185.857	1		31379.654	$7795_{4}^{{}^{\circ}}$ – 39174_3_
3184.843	300	8	31389.644	7280_2_ – $38669_{2}^{{}^{\circ}}$
3184.277	300	15b	31395.223	7280_2_ – $38675_{3}^{{}^{\circ}}$
3181.670	500r	20	31420.947	4961_4_ – $36382_{4}^{{}^{\circ}}$
3180.062	100	5	31436.835	$8243_{2}^{{}^{\circ}}$ – 39680_2_
3179.858	25	2	31438.851	$11197_{0}^{{}^{\circ}}$ – 42635_5_
3178.622	200	4	31451.076	8111_4_ – $39562_{4}^{{}^{\circ}}$
3178.244	600r	100	31454.816	2869_3_ – $34324_{2}^{{}^{\circ}}$
3178.018	3		31457.053	$7795_{4}^{{}^{\circ}}$ – 39252_5_
3177.402	100	2	31463.151	3865_1_ – $35328_{1}^{{}^{\circ}}$
3176.723	4		31469.876	8800_4_ – $40270_{4}^{{}^{\circ}}$
3176.167	200r	5	31475.385	2869_3_ – $34344_{4}^{{}^{\circ}}$
3174.840	200	4	31488.540	$7795_{4}^{{}^{\circ}}$ – 39283_4_
3174.020	300	8	31496.675	7502_3_ – $38998_{3}^{{}^{\circ}}$
3173.634	2		31500.506	8111_4_ – $39611_{3}^{{}^{\circ}}$
3173.427	500r	50	31502.560	2869_3_ – $34371_{2}^{{}^{\circ}}$
3172.808	8		31508.706	7280_2_ – $38788_{3}^{{}^{\circ}}$
3171.276	500r	75	31523.927	0_2_ – $31523_{3}^{{}^{\circ}}$
3171.015	10	3	31526.521	$7795_{4}^{{}^{\circ}}$ – 39321_3_
3170.250	5	1	31534.129	7280_2_ – $38814_{2}^{{}^{\circ}}$
3168.342	100	2	31553.118	2558_0_ – $34111_{1}^{{}^{\circ}}$
3165.048	150	3	31585.956	5563_1_ – $37149_{2}^{{}^{\circ}}$
3164.386	25	3	31592.564	6362_2_ – $37954_{3}^{{}^{\circ}}$
3164.239	200	3	31594.031	$8243_{2}^{{}^{\circ}}$ – 39837_3_
3163.715	200	3	31599.264	0_2_ – $31599_{2}^{{}^{\circ}}$
3162.856	100b		31607.846	6362_2_ – $37970_{2}^{{}^{\circ}}$
3161.364	200	5	31622.762	2869_3_ – $34492_{4}^{{}^{\circ}}$
3161.148	100		31624.923	8800_4_ – $40425_{4}^{{}^{\circ}}$
3159.577	10		31640.647	3687_2_ – $35328_{1}^{{}^{\circ}}$
3157.221	300r	8	31664.257	4961_4_ – $36625_{0}^{{}^{\circ}}$
3156.866	300		31667.818	3865_1_ – $35533_{2}^{{}^{\circ}}$
3152.395	100	3	31712.730	0_2_ – $31712_{1}^{{}^{\circ}}$
3152.295	100	3	31713.736	2869_3_ – $34583_{3}^{{}^{\circ}}$
3151.184	300	5	31724.917	$7795_{4}^{{}^{\circ}}$ – 39520_4_
3151.017	200		31726.598	6362_2_ – $38088_{2}^{{}^{\circ}}$
3147.449	200	25	31762.562	8111_4_ – $39873_{0}^{{}^{\circ}}$
3146.310	200r	8	31774.060	3687_2_ – $35462_{3}^{{}^{\circ}}$
3145.637	400r	25	31780.858	0_2_ – $31780_{3}^{{}^{\circ}}$
3144.145	100	3	31795.939	8111_4_ – $39906_{4}^{{}^{\circ}}$
3143.632	1		31801.127	4961_4_ – $36762_{3}^{{}^{\circ}}$
3142.422	100	5	31813.372	3865_1_ – $35678_{1}^{{}^{\circ}}$
3140.272	600r	50	31835.152	2869_3_ – $34704_{3}^{{}^{\circ}}$
3139.892	150	100	31839.005	4961_4_ – $36800_{4}^{{}^{\circ}}$
3139.273	150		31845.282	3687_2_ – $35533_{2}^{{}^{\circ}}$
3138.359	40	2	31854.556	6362_2_ – $38216_{3}^{{}^{\circ}}$
3138.200	100	10	31856.170	2869_3_ – $34725_{3}^{{}^{\circ}}$
3136.829	400r	8	31870.093	0_2_ – $31870_{2}^{{}^{\circ}}$
3136.612	10		31872.298	$10414_{4}^{{}^{\circ}}$ – 42286_3_
3136.216	800r	100	31876.322	4961_4_ – $36837_{0}^{{}^{\circ}}$
3133.980	200	5	31899.064	$7795_{4}^{{}^{\circ}}$ – 39694_5_
3131.496	20	2	31924.366	8800_4_ – $40724_{0}^{{}^{\circ}}$
3128.314	100	3	31956.838	$8243_{2}^{{}^{\circ}}$ – 40200_3_
3126.414	200	2	31976.258	8800_4_ – $40776_{4}^{{}^{\circ}}$
3125.812	150	2	31982.416	2869_3_ – $34851_{2}^{{}^{\circ}}$
3125.601	500r	20	31984.575	7280_2_ – $39264_{3}^{{}^{\circ}}$
3124.986	75	2	31990.869	3687_2_ – $35678_{1}^{{}^{\circ}}$
3122.102	15b		32020.419	3865_1_ – $35885_{2}^{{}^{\circ}}$
3120.881	400r	25	32032.946	2558_0_ – $34591_{1}^{{}^{\circ}}$
3119.963	100	20	32042.371	$7795_{4}^{{}^{\circ}}$ – 39837_3_
3119.504	200r		32047.085	4961_4_ – $37008_{0}^{{}^{\circ}}$
3118.848	15	1	32053.826	6362_2_ – $38416_{3}^{{}^{\circ}}$
3118.266	4		32059.808	7502_3_ – $39562_{4}^{{}^{\circ}}$
3116.352	300r	10	32079.498	4961_4_ – $37041_{4}^{{}^{\circ}}$
3116.263	500r		32080.414	0_2_ – $32080_{1}^{{}^{\circ}}$
3111.982	50	2	32124.544	3687_2_ – $35812_{3}^{{}^{\circ}}$
3111.280	300	8	32131.792	7280_2_ – $39411_{1}^{{}^{\circ}}$
3110.102	200		32143.962	8111_4_ – $40254_{3}^{{}^{\circ}}$
3108.636	200	3	32159.120	8111_4_ – $40270_{4}^{{}^{\circ}}$
3108.504	300r	10	32160.485	0_2_ – $32160_{2}^{{}^{\circ}}$
3107.214	3		32173.837	5563_1_ – $37736_{2}^{{}^{\circ}}$
3105.794	200b		32188.546	7280_2_ – $39468_{3}^{{}^{\circ}}$
3104.966	200r	3	32197.130	0_2_ – $32197_{3}^{{}^{\circ}}$
3104.892	200r	25	32197.897	4961_4_ – $37159_{3}^{{}^{\circ}}$
3102.595	20		32221.734	$10414_{4}^{{}^{\circ}}$ – 42635_5_
3099.902	201		32249.725	$8243_{2}^{{}^{\circ}}$ – 40493_3_
3099.184	100	2	32257.196	$7795_{4}^{{}^{\circ}}$ – 40052_4_
3098.726	300r	5	32261.963	2869_3_ – $35131_{4}^{{}^{\circ}}$
3096.498	25		32285.176	0_2_ – $32285_{3}^{{}^{\circ}}$
3094.373	2		32307.346	6362_2_ – $38669_{2}^{{}^{\circ}}$
3093.832	4		32312.995	6362_2_ – $38675_{3}^{{}^{\circ}}$
3093.719	200	3	32314.176	8111_4_ — $40425_{4}^{{}^{\circ}}$
3093.102	15		32320.621	2558_0_ – $34878_{1}^{{}^{\circ}}$
3092.828	300r	8	32323.484	3865_1_ – $36188_{2}^{{}^{\circ}}$
3088.567	200r	10	32368.076	$8243_{2}^{{}^{\circ}}$ – 40611_2_
3088.235	15	1	32371.556	2869_3_ – $35240_{3}^{{}^{\circ}}$
3086.852	50	1	32386.059	3687_2_ – $36074_{3}^{{}^{\circ}}$
3085.084	3b		32404.618	7502_3_ – $39906_{4}^{{}^{\circ}}$
3085.038	4		32405.101	$7795_{4}^{{}^{\circ}}$ – 40200_3_
3083.003	75b		32426.490	6362_2_ – $38788_{3}^{{}^{\circ}}$
3082.507	150r	3	32431.707	2558_0_ – $34989_{1}^{{}^{\circ}}$
3080.593	3		32451.857	6362_2_ – $38814_{2}^{{}^{\circ}}$
3078.100	3		32478.139	6362_2_ – $38840_{1}^{{}^{\circ}}$
3077.718	300r	5	32482.170	2869_3_ – $35351_{4}^{{}^{\circ}}$
3077.600	25	1	32483.415	7502_3_ – $39985_{3}^{{}^{\circ}}$
3076.410	150	30	32495.980	3865_1_ – $36361_{1}^{{}^{\circ}}$
3075.938	5		32500.966	3687_2_ – $36188_{2}^{{}^{\circ}}$
3075.687	100	3	32503.618	0_2_ – $32503_{2}^{{}^{\circ}}$
3073.589	2		32525.804	5563_1_ – $38088_{2}^{{}^{\circ}}$
3068.908	300r	5	32575.414	0_2_ – $32575_{2}^{{}^{\circ}}$
3067.270	25		32592.809	2869_3_ – $35462_{3}^{{}^{\circ}}$
3067.163	100b		32593.946	7502_3_ – $40096_{2}^{{}^{\circ}}$
3065.683	200r	2	32609.680	4961_4_ – $37571_{0}^{{}^{\circ}}$
3065.314	15b	4	32613.606	8111_4_ – $40724_{0}^{{}^{\circ}}$
3064.396	2		32623.375	3865_1_ – $36488_{2}^{{}^{\circ}}$
3063.637	8b		32631.457	$7795_{4}^{{}^{\circ}}$ – 40426_4_
3063.157	75b		32636.570	6362_2_ – $38998_{3}^{{}^{\circ}}$
3062.517	100	10	32643.391	$8243_{2}^{{}^{\circ}}$ – 40886_2_
3062.454	200r	2	32644.062	4961_4_ – $37605_{4}^{{}^{\circ}}$
3061.812	400r	3	32650.907	0_2_ – $32650_{1}^{{}^{\circ}}$
3060.928	100		32660.336	$11197_{0}^{{}^{\circ}}$ – 43857_5_
3060.582	75		32664.028	2869_3_ – $35533_{2}^{{}^{\circ}}$
3060.440	500r	10	32665.543	0_2_ – $32665_{1}^{{}^{\circ}}$
3059.698	200	1	32673.465	3687_2_ – $36361_{1}^{{}^{\circ}}$
3058.029	200r	2	32691.297	3865_1_ – $36556_{1}^{{}^{\circ}}$
3057.651	100		32695.338	4961_4_ – $37656_{3}^{{}^{\circ}}$
3056.693	150	1	32705.585	7280_2_ – $39985_{3}^{{}^{\circ}}$
3053.536	200	1	32739.397	3687_2_ – $36427_{3}^{{}^{\circ}}$
3053.462	150	1	32740.190	$11197_{0}^{{}^{\circ}}$ – 43937_6_
3052.296	3		32752.697	7502_3_ – $40254_{3}^{{}^{\circ}}$
3052.128	100	1	32754.500	0_2_ – $32754_{3}^{{}^{\circ}}$
3050.892	4b		32767.769	7502_3_ – $40270_{4}^{{}^{\circ}}$
3050.631	200r	2	32770.572	2558_0_ – $35328_{1}^{{}^{\circ}}$
3049.054	100b		32787.521	$7795_{4}^{{}^{\circ}}$ – 40582_5_
3048.621	10		32792.177	5563_1_ – $38355_{2}^{{}^{\circ}}$
3047.816	500r	2	32800.838	3687_2_ – $36488_{2}^{{}^{\circ}}$
3046.401	4		32816.073	7280_2_ – $40096_{2}^{{}^{\circ}}$
3041.941	100		32864.185	2869_3_ – $35733_{4}^{{}^{\circ}}$
3041.515	300r	100	32868.788	3687_2_ – $36556_{1}^{{}^{\circ}}$
3041.433	40b		32869.674	8111_4_ – $40980_{3}^{{}^{\circ}}$
3038.417	1001		32902.300	6362_2_ – $39264_{3}^{{}^{\circ}}$
3038.070	100		32906.057	$7795_{4}^{{}^{\circ}}$ – 40701_4_
3036.518	10b	2	32922.875	7502_3_ – $40425_{4}^{{}^{\circ}}$
3034.639	400r	2	32943.260	2869_3_ – $35812_{3}^{{}^{\circ}}$
3033.595	15		32954.597	0_2_ – $32954_{2}^{{}^{\circ}}$
3032.016	100r	2	32971.758	3865_1_ – $36837_{1}^{{}^{\circ}}$
3031.733	75b		32974.836	7280_2_ – $40254_{3}^{{}^{\circ}}$
3031.195	100r	2	32980.688	3687_2_ – $36668_{2}^{{}^{\circ}}$
3030.487	400r	8	32988.393	4961_4_ – $37950_{4}^{{}^{\circ}}$
3030.035	75		32993.314	4961_4_ – $37954_{3}^{{}^{\circ}}$
3028.830	200	2	33006.439	3865_1_ – $36871_{2}^{{}^{\circ}}$
3027.896	150	3	33016.620	2869_3_ – $35885_{2}^{{}^{\circ}}$
3027.228	200b	15	33023.906	8111_4_ – $41134_{4}^{{}^{\circ}}$
3025.448	200r	2	33043.334	0_2_ – $33043_{3}^{{}^{\circ}}$
3024.883	150		33049.506	6362_2_ – $39411_{1}^{{}^{\circ}}$
3022.567	3	1	33074.829	3687_2_ – $36762_{3}^{{}^{\circ}}$
3021.056	300	1	33091.370	4961_4_ – $38053_{3}^{{}^{\circ}}$
3018.644	100		33117.810	$8243_{2}^{{}^{\circ}}$ – 41361_3_
3015.785	40	3	33149.205	3687_2_ – $36837_{1}^{{}^{\circ}}$
3014.641	150r	1	33161.784	0_2_ – $33161_{1}^{{}^{\circ}}$
3012.630	150		33183.919	3687_2_ – $36871_{2}^{{}^{\circ}}$
3010.736	200r	2	33204.794	2869_3_ – $36074_{3}^{{}^{\circ}}$
3007.476	100	1	33240.785	$11197_{0}^{{}^{\circ}}$ – 44437_6_
3006.720	3		33249.143	6362_2_ – $39611_{3}^{{}^{\circ}}$
3006.164	300r	2	33255.292	4961_4_ – $38216_{3}^{{}^{\circ}}$
3004.163	3b		33277.442	5563_1_ – $38840_{1}^{{}^{\circ}}$
3003.605	200	1	33283.624	3865_1_ – $37149_{2}^{{}^{\circ}}$
3002.687	200r	2	33293.799	3865_1_ – $37159_{1}^{{}^{\circ}}$
3002.390	100b	10	33297.092	0_2_ – $33297_{2}^{{}^{\circ}}$
3001.336	50r		33308.785	2869_3_ – $36178_{4}^{{}^{\circ}}$
3000.353	100b		33319.697	2869_3_ – $36188_{2}^{{}^{\circ}}$
2998.103	150b		33344.702	4961_4_ – $38306_{4}^{{}^{\circ}}$
2995.962	100	1	33368.530	3865_1_ – $37234_{2}^{{}^{\circ}}$
2991.032	75b		33423.528	4961_4_ – $38385_{0}^{{}^{\circ}}$
2990.717	75		33427.048	0_2_ – $33427_{2}^{{}^{\circ}}$
2989.272	401		33443.206	$10414_{4}^{{}^{\circ}}$ – 43857_5_
2988.255	300r		33454.587	4961_4_ – $38416_{3}^{{}^{\circ}}$
2987.672	300r	2	33461.115	3687_2_ – $37149_{2}^{{}^{\circ}}$
2986.740	100r	2	33471.556	3687_2_ – $37159_{3}^{{}^{\circ}}$
2986.127	2		33478.427	7502_3_ – $40980_{3}^{{}^{\circ}}$
2980.112	200r	2	33545.996	3687_2_ – $37234_{2}^{{}^{\circ}}$
2979.035	100		33558.123	2869_3_ – $36427_{3}^{{}^{\circ}}$
2978.791	75	2	33560.872	$7795_{4}^{{}^{\circ}}$ – 41356_5_
2978.325	100	1	33566.122	$7795_{4}^{{}^{\circ}}$ – 41361_3_
2976.103	200r	3	33591.182	0_2_ – $33591_{3}^{{}^{\circ}}$
2973.675	100	1	33618.608	4961_4_ – $38580_{4}^{{}^{\circ}}$
2973.590	15		33619.569	2869_3_ – $36488_{2}^{{}^{\circ}}$
2972.438	10		33632.598	7502_3_ – $41134_{4}^{{}^{\circ}}$
2969.822	200r	4	33662.223	0_2_ – $33662_{1}^{{}^{\circ}}$
2966.662	5		33698.077	7502_3_ – $41200_{2}^{{}^{\circ}}$
2966.441	8		33700.587	7280_2_ – $40980_{3}^{{}^{\circ}}$
2966.150	1001	5	33703.894	4961_4_ – $38665_{0}^{{}^{\circ}}$
2965.290	15		33713.668	4961_4_ – $38675_{3}^{{}^{\circ}}$
2963.521	2		33733.792	6362_2_ – $40096_{2}^{{}^{\circ}}$
2963.442	75		33734.691	0_2_ – $33734_{2}^{{}^{\circ}}$
2959.137	200	2	33783.767	$8243_{2}^{{}^{\circ}}$ – 42027_2_
2957.767	75	3	33799.414	2869_3_ – $36668_{2}^{{}^{\circ}}$
2957.660	100r	5	33800.637	0_2_ – $33800_{3}^{{}^{\circ}}$
2957.421	10b		33803.368	2558_0_ – $36361_{1}^{{}^{\circ}}$
2955.339	10		33827.181	4961_4_ – $38788_{3}^{{}^{\circ}}$
2953.454	25	1	33848.770	5563_1_ – $39411_{1}^{{}^{\circ}}$
2951.471	50	1	33871.511	3865_1_ – $37736_{2}^{{}^{\circ}}$
2949.736	20	2	33891.433	$7795_{4}^{{}^{\circ}}$ – 41686_3_
2949.639	50	2	33892.547	6362_2_ – $40254_{3}^{{}^{\circ}}$
2949.551	100r	15b	33893.559	2869_3_ – $36762_{3}^{{}^{\circ}}$
2947.462	100	3	33917.579	$7795_{4}^{{}^{\circ}}$ – 41712_5_
2947.229	200	25	33920.261	7280_2_ – $41200_{2}^{{}^{\circ}}$
2946.528	100	20	33928.330	3687_2_ – $37616_{1}^{{}^{\circ}}$
2946.258	200r	50	33931.439	2869_3_ – $36800_{4}^{{}^{\circ}}$
2943.729	400	3	33960.589	$7795_{4}^{{}^{\circ}}$ – 41755_4_
2943.095	300r	2	33967.904	0_2_ – $33967_{3}^{{}^{\circ}}$
2943.000	300r	20	33969.001	3687_2_ – $37656_{3}^{{}^{\circ}}$
2940.432	40h		33998.666	2558_0_ – $36556_{1}^{{}^{\circ}}$
2940.088	800r	50	34002.644	2869_3_ – $36871_{2}^{{}^{\circ}}$
2937.095	5		34037.292	4961_4_ – $38998_{3}^{{}^{\circ}}$
2936.617	150h	3	34042.832	$8243_{2}^{{}^{\circ}}$ – 42286_3_
2936.089	500	5	34048.954	3687_2_ – $37736_{2}^{{}^{\circ}}$
2931.663	150b	25	34100.356	4961_4_ – $39062_{0}^{{}^{\circ}}$
2931.281	300r	2	34104.800	3865_1_ – $37970_{2}^{{}^{\circ}}$
2930.735	4		34111.153	0_2_ – $34111_{1}^{{}^{\circ}}$
2925.526	500r	5	34171.887	2869_3_ – $37041_{4}^{{}^{\circ}}$
2921.560	15b		34218.273	7502_3_ – $41720_{2}^{{}^{\circ}}$
2916.369	500r	10	34279.177	2558_0_ – $36837_{1}^{{}^{\circ}}$
2916.313	75b	2	34279.835	2869_3_ – $37149_{2}^{{}^{\circ}}$
2916.105	100	2	34282.280	3687_2_ – $37970_{2}^{{}^{\circ}}$
2915.424	100	2	34290.287	2869_3_ – $37159_{3}^{{}^{\circ}}$
2912.553	100	3	34324.087	0_2_ – $34324_{2}^{{}^{\circ}}$
2910.390	150	2	34349.595	4961_4_ – $39311_{4}^{{}^{\circ}}$
2909.103	300d	8d	34364.791	2869_3_ – $37234_{2}^{{}^{\circ}}$
2909.080	50b	5b	34365.063	3687_2_ – $38053_{3}^{{}^{\circ}}$
2908.507	200r	20	34371.833	0_2_ – $34371_{2}^{{}^{\circ}}$
2902.714	300	2	34440.426	7280_2_ – $41720_{2}^{{}^{\circ}}$
2898.556	100	2	34489.829	3865_1_ – $38355_{2}^{{}^{\circ}}$
2898.443	8b		34491.174	$7795_{4}^{{}^{\circ}}$ – 42286_3_
2897.112	40b	20b	34507.019	4961_4_ – $39468_{3}^{{}^{\circ}}$
2895.271	150	3	34528.960	3687_2_ – $38216_{3}^{{}^{\circ}}$
2890.746	40		34583.007	0_2_ – $34583_{3}^{{}^{\circ}}$
2890.078	150r	5	34591.000	0_2_ – $34591_{1}^{{}^{\circ}}$
2889.290	150r	15	34600.433	4961_4_ – $39562_{4}^{{}^{\circ}}$
2885.169	75		34649.852	4961_4_ – $39611_{3}^{{}^{\circ}}$
2883.713	200w	2	34667.346	3687_2_ – $38355_{2}^{{}^{\circ}}$
2880.632	200r	3	34704.423	0_2_ – $34704_{3}^{{}^{\circ}}$
2878.657	150b		34728.232	3687_2_ – $38416_{3}^{{}^{\circ}}$
2877.974	5		34736.473	2869_3_ – $37605_{4}^{{}^{\circ}}$
2873.734	75b		34787.722	2869_3_ – $37656_{3}^{{}^{\circ}}$
2869.589	2		34837.969	6362_2_ – $41200_{2}^{{}^{\circ}}$
2869.374	5		34840.580	$7795_{4}^{{}^{\circ}}$ – 42635_5_
2868.460	300r	10	34851.681	0_2_ – $34851_{2}^{{}^{\circ}}$
2867.141	40b		34867.713	2869_3_ – $37736_{2}^{{}^{\circ}}$
2866.241	40		34878.661	0_2_ – $34878_{1}^{{}^{\circ}}$
2863.510	10		34911.924	4961_4_ – $39873_{0}^{{}^{\circ}}$
2860.776	100	2	34945.287	4961_4_ – $39906_{4}^{{}^{\circ}}$
2860.491	150	3	34948.769	3865_1_ – $38814_{2}^{{}^{\circ}}$
2857.336	10		34987.356	3687_2_ – $38675_{3}^{{}^{\circ}}$
2857.138	100	2	34989.781	0_2_ – $34989_{1}^{{}^{\circ}}$
2854.342	300	3	35024.054	4961_4_ – $39985_{3}^{{}^{\circ}}$
2851.558	50r	4	35058.246	2558_0_ – $37616_{1}^{{}^{\circ}}$
2849.725	10	1	35080.796	2869_3_ – $37950_{4}^{{}^{\circ}}$
2849.326	100	25	35085.708	2869_3_ – $37954_{3}^{{}^{\circ}}$
2848.084	300r	2	35101.007	2869_3_ – $37970_{2}^{{}^{\circ}}$
2846.036	150	2	35126.265	3687_2_ – $38814_{2}^{{}^{\circ}}$
2843.906	300r	2	35152.572	3687_2_ – $38840_{1}^{{}^{\circ}}$
2841.383	150h	2	35183.784	2869_3_ – $38053_{3}^{{}^{\circ}}$
2838.483	300r	2	35219.729	2869_3_ – $38088_{2}^{{}^{\circ}}$
2836.783	3		35240.834	0_2_ – $35240_{3}^{{}^{\circ}}$
2832.568	100		35293.271	4961_4_ – $40254_{3}^{{}^{\circ}}$
2831.146	20	4	35310.997	3687_2_ – $38998_{3}^{{}^{\circ}}$
2829.732	150r	2	35328.641	0_2_ – $35328_{1}^{{}^{\circ}}$
2828.502	2001	4	35344.003	7280_2_ – $42624_{1}^{{}^{\circ}}$
2828.206	150r	4	35347.702	2869_3_ – $38216_{3}^{{}^{\circ}}$
2821.072	100s	2	35437.086	2869_3_ – $38306_{4}^{{}^{\circ}}$
2818.971	15		35463.496	4961_4_ – $40425_{4}^{{}^{\circ}}$
2817.180	5		35486.041	2869_3_ – $38355_{2}^{{}^{\circ}}$
2813.434	100		35533.287	0_2_ – $35533_{2}^{{}^{\circ}}$
2812.395	8b		35546.414	3865_1_ – $39411_{1}^{{}^{\circ}}$
2810.002	100		35576.683	3687_2_ – $39264_{3}^{{}^{\circ}}$
2805.228	4	1	35637.226	5563_1_ – $41200_{2}^{{}^{\circ}}$
2801.957	25		35678.826	0_2_ – $35678_{1}^{{}^{\circ}}$
2799.432	100	1	35711.006	2869_3_ – $38580_{4}^{{}^{\circ}}$
2798.421	75		35723.907	3687_2_ – $39411_{1}^{{}^{\circ}}$
2795.368	100		35762.921	4961_4_ – $40724_{0}^{{}^{\circ}}$
2792.433	15	1	35800.508	2869_3_ – $38669_{2}^{{}^{\circ}}$
2791.999	50	1	35806.073	2869_3_ – $38675_{3}^{{}^{\circ}}$
2791.499	100b		35812.486	0_2_ – $35812_{3}^{{}^{\circ}}$
2782.871	100		35923.513	3687_2_ – $39611_{3}^{{}^{\circ}}$
2781.209	50		35944.979	2869_3_ – $38814_{2}^{{}^{\circ}}$
2775.488	100		36019.067	4961_4_ – $40980_{3}^{{}^{\circ}}$
2772.176	4		36062.098	$7795_{4}^{{}^{\circ}}$ – 43857_5_
2771.257	75		36074.056	0_2_ – $36074_{3}^{{}^{\circ}}$
2766.988	50		36129.709	2869_3_ – $38998_{3}^{{}^{\circ}}$
2763.655	40b		36173.280	4961_4_ – $41134_{4}^{{}^{\circ}}$
2762.458	100r		36188.953	0_2_ – $36188_{2}^{{}^{\circ}}$
2759.272	8		36230.737	3865_1_ – $40096_{2}^{{}^{\circ}}$
2756.912	3		36261.750	6362_2_ – $42624_{1}^{{}^{\circ}}$
2755.336	75		36282.490	2558_0_ – $38840_{1}^{{}^{\circ}}$
2754.179	8		36297.731	3687_2_ – $39985_{3}^{{}^{\circ}}$
2749.351	50	2	36361.468	0_2_ – $36361_{1}^{{}^{\circ}}$
2746.786	100		36395.421	2869_3_ – $39264_{3}^{{}^{\circ}}$
2744.376	20		36427.381	0_2_ – $36427_{3}^{{}^{\circ}}$
2743.274	3		36442.013	2869_3_ – $39311_{4}^{{}^{\circ}}$
2739.753	50		36488.844	0_2_ – $36488_{2}^{{}^{\circ}}$
2734.661	40	2	36556.783	0_2_ – $36556_{1}^{{}^{\circ}}$
2733.900	50		36566.959	3687_2_ – $40254_{3}^{{}^{\circ}}$
2726.317	201		36668.661	0_2_ – $36668_{2}^{{}^{\circ}}$
2724.522	25		36692.818	2869_3_ – $39562_{4}^{{}^{\circ}}$
2720.857	50		36742.241	2869_3_ – $39611_{3}^{{}^{\circ}}$
2719.333	15b		36762.831	0_2_ – $36762_{3}^{{}^{\circ}}$
2713.843	75		36837.197	0_2_ – $36837_{1}^{{}^{\circ}}$
2712.620	2		36853.804	2558_0_ – $39411_{1}^{{}^{\circ}}$
2711.288	5		36871.909	0_2_ – $36871_{2}^{{}^{\circ}}$
2699.153	2		37037.670	2869_3_ – $39906_{4}^{{}^{\circ}}$
2693.425	50h		37116.433	2869_3_ – $39985_{3}^{{}^{\circ}}$
2691.057	200r		37149.091	0_2_ – $37149_{2}^{{}^{\circ}}$
2690.319	10		37159.281	0_2_ – $37159_{1}^{{}^{\circ}}$
2685.426	3		37226.984	2869_3_ – $40096_{2}^{{}^{\circ}}$
2680.688	100r		37292.777	3687_2_ – $40980_{3}^{{}^{\circ}}$
2677.666	10		37334.863	3865_1_ – $41200_{2}^{{}^{\circ}}$
2674.029	3		37385.640	2869_3_ – $40254_{3}^{{}^{\circ}}$
2664.996	8		37512.351	3687_2_ – $41200_{2}^{{}^{\circ}}$
2657.633	3		37616.274	0_2_ – $37616_{1}^{{}^{\circ}}$
2654.762	4		37656.952	0_2_ – $37656_{3}^{{}^{\circ}}$
2640.865	10		37855.102	3865_1_ – $41720_{2}^{{}^{\circ}}$
2633.914	2		37954.997	0_2_ – $37954_{3}^{{}^{\circ}}$
2632.854	20	1	37970.277	0_2_ – $37970_{2}^{{}^{\circ}}$
2627.128	5		38053.031	0_2_ – $38053_{3}^{{}^{\circ}}$
2624.647	100r		38088.999	0_2_ – $38088_{2}^{{}^{\circ}}$
2623.100	100r		38111.461	2869_3_ – $40980_{3}^{{}^{\circ}}$
2615.858	5		38216.966	0_2_ – $38216_{3}^{{}^{\circ}}$
2611.626	15	2	38278.891	0_2_ – $38278_{1}^{{}^{\circ}}$
2606.425	20		38355.270	0_2_ – $38355_{2}^{{}^{\circ}}$
2602.290	4		38416.212	0_2_ – $38416_{3}^{{}^{\circ}}$
2585.224	3		38669.796	0_2_ – $38669_{2}^{{}^{\circ}}$
2584.851	2		38675.375	0_2_ – $38675_{3}^{{}^{\circ}}$
2577.288	75r	3	38788.861	0_2_ – $38788_{3}^{{}^{\circ}}$
2575.599	3		38814.295	0_2_ – $38814_{2}^{{}^{\circ}}$
2573.854	3		38840.609	0_2_ – $38840_{1}^{{}^{\circ}}$
2563.402	2		38998.967	0_2_ – $38998_{3}^{{}^{\circ}}$
2546.052	100		39264.707	0_2_ – $39264_{3}^{{}^{\circ}}$
2536.540	25		39411.940	0_2_ – $39411_{1}^{{}^{\circ}}$
2523.760	25		39611.503	0_2_ – $39611_{3}^{{}^{\circ}}$
2500.142	10		39985.674	0_2_ – $39985_{3}^{{}^{\circ}}$
2495.126	10		40066.053	2558_0_ – $42624_{1}^{{}^{\circ}}$
2493.250	100		40096.198	0_2_ – $40096_{2}^{{}^{\circ}}$
2483.418	50		40254.930	0_2_ – $40254_{3}^{{}^{\circ}}$
2439.433	150r		40980.707	0_2_ – $40980_{3}^{{}^{\circ}}$
2345.372	50		42624.104	0_2_ – $42624_{1}^{{}^{\circ}}$

## Footnotes

1 Dates enclosed in brackets indicate literature references at the end of this paper.
